# Improved Metabolic Health Alters Host Metabolism in Parallel with Changes in Systemic Xeno-Metabolites of Gut Origin

**DOI:** 10.1371/journal.pone.0084260

**Published:** 2014-01-08

**Authors:** Caitlin Campbell, Dmitry Grapov, Oliver Fiehn, Carol J. Chandler, Dustin J. Burnett, Elaine C. Souza, Gretchen A. Casazza, Mary B. Gustafson, Nancy L. Keim, John W. Newman, Gary R. Hunter, Jose R. Fernandez, W. Timothy Garvey, Mary-Ellen Harper, Charles L. Hoppel, John K. Meissen, Kohei Take, Sean H. Adams

**Affiliations:** 1 USDA-ARS Western Human Nutrition Research Center, Davis, California, United States of America; 2 West Coast Metabolomics Center, University of California Davis, Davis, California, United States of America; 3 Genome Center, University of California Davis, Davis, California, United States of America; 4 Sports Medicine Program, University of California, Davis School of Medicine, Sacramento, California, United States of America; 5 Department of Nutrition, University of California Davis, Davis, California, United States of America; 6 Department of Nutrition Sciences, University of Alabama, Birmingham, Alabama, United States of America; 7 Human Studies Department, University of Alabama, Birmingham, Alabama, United States of America; 8 Department of Biochemistry, Microbiology and Immunology, University of Ottawa, Ottawa, Ontario, Canada; 9 Pharmacology Department, Case Western Reserve University, Cleveland, Ohio, United States of America; Institut d'Investigacions Biomèdiques August Pi i Sunyer, Spain

## Abstract

Novel plasma metabolite patterns reflective of improved metabolic health (insulin sensitivity, fitness, reduced body weight) were identified before and after a 14–17 wk weight loss and exercise intervention in sedentary, obese insulin-resistant women. To control for potential confounding effects of diet- or microbiome-derived molecules on the systemic metabolome, sampling was during a tightly-controlled feeding test week paradigm. Pairwise and multivariate analysis revealed intervention- and insulin-sensitivity associated: (1) Changes in plasma xeno-metabolites (“non-self” metabolites of dietary or gut microbial origin) following an oral glucose tolerance test (e.g. higher post-OGTT propane-1,2,3-tricarboxylate [tricarballylic acid]) or in the overnight-fasted state (e.g., lower γ-tocopherol); (2) Increased indices of saturated very long chain fatty acid elongation capacity; (3) Increased post-OGTT α-ketoglutaric acid (α-KG), fasting α-KG inversely correlated with Matsuda index, and altered patterns of malate, pyruvate and glutamine hypothesized to stem from improved mitochondrial efficiency and more robust oxidation of glucose. The results support a working model in which improved metabolic health modifies host metabolism in parallel with altering systemic exposure to xeno-metabolites. This highlights that interpretations regarding the origins of peripheral blood or urinary “signatures” of insulin resistance and metabolic health must consider the potentially important contribution of gut-derived metabolites toward the host's metabolome.

## Introduction

Pre-diabetes and type 2 diabetes mellitus (T2DM) are defined by elevated blood glucose following an overnight fast or at 2 hr following an oral glucose tolerance test (OGTT) [1]; however, a clinically-significant increase in blood sugar is a late event in disease progression and is not an optimal prognostic. Identifying more sensitive T2DM risk markers or those that track deteriorating insulin sensitivity would have potential value as clinical diagnostics and would help elucidate the underlying pathophysiology. Advancements in metabolomics technologies to interrogate hundreds of metabolites in human blood or urine hold promise in this regard. Recent metabolomics studies have highlighted that human insulin resistance, T2DM, and T2DM risk involve significant perturbations in lipid and amino acid metabolism in addition to glucose, as reflected in altered phosphatidylcholine derivatives, positive associations with blood branched-chain amino acids (BCAAs), 2-hydroxybutyrate (2-HB), long- and medium-chain acylcarnitines, and negative associations with blood glycine and linoleoyl-glycerophosphocholine (L-GPC)[2]–[16].

Measurement of blood metabolites in the overnight-fasted state, while valuable, may not unmask subtle phenotypes associated with insulin resistance or pre-diabetes that manifest when the body's metabolic machinery is challenged. Since insulin resistance involves impairment of normal glucose and insulin homeostasis, metabolomics analyses following an OGTT are an attractive means to identify biochemical pathways associated with individual variability in insulin action and blood sugar control. To our knowledge, only five studies have reported post-OGTT blood metabolite profiling in humans [17]–[21]. These reports highlighted that in healthy individuals a glucose challenge with attendant increases in blood insulin and glucose is accompanied by expected reductions in plasma indices of lipolysis (e.g., glycerol, long-chain fatty acids [LCFA]) and LCFA β-oxidation (e.g., chain-shortened fatty acylcarnitines, β-hydroxybutyrate), and increases in markers of tissue amino acid utilization (e.g., reductions in blood amino acids) and glycolysis (e.g. lactate). Interestingly, the OGTT significantly increased blood bile acid and lysophosphotidylcholine metabolites, although the etiology of this was not identified [17], [19]. Post-OGTT metabolite patterns have also been compared between normal glucose tolerant (NGT) and impaired glucose tolerant (IGT) subjects: IGT persons showed blunted post-OGTT changes in lactate, glycerol and glycerol-3-phosphate, and leucine/isoleucine [17], [20].

We are not aware of any reports examining OGTT-associated metabolomics to determine longitudinal changes in circulating metabolite patterns, to test the hypothesis that improvements in insulin sensitivity or metabolic fitness alter comprehensive metabolite responses. For instance, one would expect that plasma markers that positively correlate with insulin resistance or T2DM in cross-sectional studies (see above) would be reduced by interventions that increase insulin sensitivity. We examined this issue in obese sedentary women with modest hyperinsulinemia, tested before and after a 14–17 week weight loss and fitness intervention and while fed a controlled diet. A subset of the 321 detected plasma metabolites were altered in the fasted state or following an OGTT when comparing pre- vs. post-intervention. Variance in both expected and novel markers, including several putative xeno-metabolites (i.e., of non-endogenous origin, from diet and/or gut microbial metabolism), discriminated the pre- vs. post-intervention condition. One or more of these metabolites may be useful to track improvements in or deterioration of metabolic health, and the results point to alterations in gut metabolism or microbial ecology that occur in response to improvements in host metabolic health.

## Methods

### Human Subjects

All protocols were approved by the University of California at Davis Institutional Review Board, and all subjects provided informed written consent. The study is listed in ClinicalTrials.gov (NCT01494025). An important goal of the research was to identify metabolites that are responsive to changes in insulin sensitivity due to improvements in fitness and body mass following a diet and exercise intervention. To this end, obese modestly hyperinsulinemic 30–50 year old females were recruited from the greater Davis and Sacramento, California communities. All participants were eumenorrheic, non-smoking, and sedentary (typical planned exercise <30 min. per week). Body mass index (BMI) was between 30–37.5 kg/m^2^ and participants reported that they were weight stable as defined <5% change in body mass over the past 6 months. Participants were insulin-resistant at the time of screening as determined by an abbreviated oral glucose tolerance test (OGTT) that consisted of an initial blood draw (following an overnight fast) and an additional blood draw 2 hours after consuming a standard 75 g glucose drink (Fisher Scientific, catalog #s 401025-FB, 401526-FB, 401223-FB). Insulin resistance was defined as one or more of the following: (a) as per the American Diabetes Association guidelines for pre-diabetes, fasting glucose ≥100 and <126 mg/dL or 2-hour OGTT glucose ≥140 and <199 mg/dL; and/or (b) a target Quantitative Insulin Sensitivity Check Index (QUICKI) score <0.315, Homeostasis Model Assessment (HOMA) >3.67, or logHOMA >0.085. The latter criteria were derived using the upper limit of normal fasting glucose (100 mg/dL) and a serum insulin (15 µU/mL) that approximates the upper value of the third quartile of 72 normoglycemic men and women tested by the UC Davis Medical Center (UCDMC) Pathology Laboratory in the course of establishing their normal ranges for insulin using the ADVIA Centaur instrument. One subject displayed normal plasma glucose and a borderline QUICKI score of 0.316, but was included in the study because she met all other inclusion criteria, had a fasting insulin of 17 µU/mL and high lipids (cholesterol 266 mg/dL; triglycerides 440 mg/dL), consistent with an insulin resistance phenotype. Exclusion criteria included any clinical signs of infection, chronic disease, personal history of cardiovascular disease, elevated blood pressure (>130/85), diabetes, regular medications other than oral contraceptives and pregnancy or lactation. Out of 511 initial phone screens, a total of 18 subjects were enrolled, 2 dropped prior to testing, 16 participated through the first phase of the study, and 1 subject was not compliant with the prescribed diet provided during either Test Week and was therefore excluded (**Figure S1**). Three subjects dropped following Test Week 1, prior to or during weight loss/exercise intervention, leaving 12 of 15 starting subjects available for re-examination in Test Week 2.

### Pre- and Post- Intervention Test Week Diet and OGTT Protocol

Participants completed testing before (“Test Week 1”) and after (“Test Week 2”) an exercise and weight loss intervention (14–17 weeks) designed to improve fitness and insulin sensitivity (described below). To minimize variability in metabolomics that could be influenced by differences in diet composition, during Test Week 1 and Test Week 2 the participants were provided standardized meals and snacks, with foods lot-matched within-subject. Diets were prescribed to maintain body mass during the specific Test Week using the DRI equation. Mass was determined daily during Test Weeks and small changes in prescription calories were made to maintain body mass within 5%. Study menus were designed by a registered dietitian and prepared by the WHNRC Metabolic Food Lab using the University of Minnesota's Nutrient Data System for Research (NDS-R) version 2009 and ProNutra software (Viocare Technologies, Inc.), guided by the 2005 Dietary Guidelines for Americans (DGs). Menus consisted of primarily shelf-stable (e.g., frozen, dried, canned) foods (see **File S1**: Supplemental Materials 1). For the first 3 d, each subject was fed Menu 1; for the subsequent 3–5 d, subjects were fed Menu 2. The average calculated nutrient composition based on the actual menus provided to the study participants, as well as the targets for meeting the DG's 2000-kcal goals are provided in **File S1** (Supplemental Materials 1); these targets were confirmed by *post hoc* composite analysis of the diet (Covance) During Test Week 1 and Test Week 2, participants were instructed to eat and drink only what was provided to them by the study team, and encouraged to eat study meals on site; however, for logistical reasons (e.g., work schedules) many meals were packed “to go.” Self-reported compliance was determined with daily food diaries (**File S1**: Supplemental Materials 1). On specific test days when participants ate a meal in-house (e.g., following the OGTT completion), meals were monitored by the Metabolic Food Lab staff.

A primary aim of the study was to examine fasting plasma metabolomics patterns following the exercise and weight loss intervention. A secondary aim (OGTT metabolomics) was initiated after the first two enrolled subjects (a completer and a non-completer in terms of the entire weight loss/fitness intervention) finished Test Week 1. Thus, Test Week 1 OGTT metabolomics were only available for 13 subjects. After an overnight fast, during Day 3 or 4 of the Test Week period, participants reported to the WHNRC at which time most women had a catheter placed in an antecubital vein (others for which catheterization was difficult had samples collected by venipuncture during the OGTT). Approximately 15–30 min. later, a standard 75 g glucose OGTT was administered. EDTA-treated blood samples were taken prior to the glucose drink (“overnight fasted”) and at 30, 60, 90, and 120 minutes after ingestion. Plasma glucose was determined at the UCDMC using a Beckman Coulter clinical analyzer DXC800, and serum insulin was analyzed using standard methods as per manufacturer's protocols (ADVIA Centaur, Siemans). The insulin sensitivity index (“Matsuda index”) was calculated per Matsuda and DeFronzo [22]. HbA1c was measured by UCDMC using a Trinity Biotech Ultra 2 HbA1c Analyzer.

### Weight Loss and Fitness Regimen

Subjects were prescribed a self-selected calorie-restricted diet based on the DGs and using the DRI equation to target a 10% body mass loss over 14 weeks (ca. 500–600 kcal/day reduction). A Baecke physical activity questionnaire [23] was administered to assess self-reported physical activity level with a score of 5 for the lowest activity and 15 for the highest activity related to work, sport/exercise, and non-sport leisure categories; we used a score of 7 for calculating maintenance calories. Participants recorded daily food intake in diaries and received weekly counseling from a registered dietitian. Subjects were provided with a daily nutritional supplement (Bayer One-a-Day for Women) during the intervention to assure adequate intake of essential vitamins and minerals. Body mass was measured weekly on an electronic scale (Scale-Tronic model 6002; Wheaton, IL) to the nearest 0.1 kg with participants in light clothing, all jewelry removed, pockets emptied, and without shoes. Height was measured to the nearest 0.1 cm using a wall-mounted stadiometer (Ayrton Stadiometer model S100; Prior Lake, MN), and body mass index (BMI, kg/m^2^) calculated. Body fat mass and fat-free mass were measured by dual energy X-ray absorptiometry (DEXA, GE Lunar Prodigy Encore v10.5, Madison, WI). During Test Week 1, a graded cycle ergometer test (SRM ergometer, Colorado Springs, CO) was preformed to determine peak oxygen consumption (VO_2peak_). Participants arrived at the UC Davis Sports Medicine Clinic after consuming a standard breakfast (Menu 2) 2–3 hours prior to exercise. During Test Week 1, the participants received a resting ECG, a spirometry test, and a medical clearance exam by a Sports Medicine Clinic physician. For the exercise test, participants completed a 5 min warm up, followed by a graded exercise test to exhaustion (initial workload of 50 W, increased by 20 W every 2 min until volitional fatigue). A metabolic cart (Parvo Medics TrueOne 2400, Sandy, UT) was used to take continuous indirect calorimetry measurements. At the end of every 2-min stage, the following data were recorded: HR, blood pressure (BP) by auscultation, and rating of perceived exertion (RPE) using a 0–10 scale [24]. VO_2peak_ was determined as the highest VO_2_ (ml/kg/min) over a 30 s period. The VO_2peak_ measurement was replicated during Test Week 2.

Total length of time for the weight loss and fitness intervention phase of the study varied from 14–17 wk, necessitated to match Test Weeks with respect to menstrual phase within each subject to avoid any potential cycle-associated changes in glucose and insulin homeostasis [25], [26] or metabolomics outcomes. If a follicular phase (by self-report of menses) could not be achieved for an individual for Test Week 1, that individual's Test Week 2 was targeted to coincide with the same reported menstrual phase they were in for Test Week 1. Serum luteinizing hormone (LH) and follicle stimulating hormone (FSH) were analyzed at UCDMC (Siemens ADVIA Centaur chemiluminometric immunoassay) to evaluate cycle stage *post hoc*.

Participants engaged in a prescribed exercise regimen a minimum of 4 times/wk for the duration of the intervention as directed by WHNRC Physiology Support Lab (PSL) exercise physiologists. Over the first 4 intervention weeks, participants exercised aerobically 4 days/wk for 30 minutes each (treadmill or cycle ergometer) at an intensity of 60–70% of their maximal HR as determined in the VO_2peak_ test. During intervention weeks 5–8, exercise sessions were increased to 40 minutes/session, 4 days/wk and during intervention weeks 9 onward the intensity was increased to a HR of 75% of maximal. Participants wore HR monitors during all exercise sessions to ensure that they were exercising at the appropriate intensity for the prescribed amount of time, with digital information downloaded by PSL staff weekly to ensure compliance.

### Metabolite Analysis

The details of sample handling, metabolite detection and analysis have been presented in detail elsewhere [8], [27]. In brief, EDTA plasma aliquots (15 µL) were extracted and a set of 13 C8–C30 fatty acid methyl ester internal standards were added, followed by methoximation/trimethylsilylation derivitization with 10 µL methoxyamine hydrochloride in pyridine followed by 90 µL MSTFA. Analytes in a 0.5 µL sample injection were separated using an Agilent 6890 gas chromatograph (Santa Clara, CA) equipped with a 30 m×0.25 mm i.d., Rtx5Sil-MS column with 0.25 µm 5% diphenyl film and a 10 m integrated guard column (Restek, Bellefonte PA). Chromatography was performed with constant flow of 1 mL/min while ramping the oven temperature from 50°C to 330°C with 22 min total run time. Mass spectra were acquired on a Leco Pegasus IV time of flight mass spectrometer (St. Joseph, MI) with a 280°C transfer line, a 250°C ion source, and −70eV electron ionization impact. Mass spectra were acquired from m/z 85–500 at 17 spectra s−1 and 1850 V detector voltage. Result files were exported to servers and processed by the Fiehn lab metabolomics BinBase database. Database entries in BinBase were matched against the Fiehn mass spectral library of 1,200 authentic metabolite spectra using retention index and mass spectrum information or the NIST05 commercial library. Identified metabolites were reported if present in at least 50% of the samples. Peak heights of quantifier ions for each reported metabolite were normalized to the sum intensities of all reported metabolites and these relative abundances were used for statistical investigation. External 5-point calibration curves established with mixtures of 30 metabolites allowed for the routine assessment of instrument sensitivity and analyte intensity with respect to the instrument dynamic range. Each chromatogram was further controlled with respect to the total number of identified metabolites and total peak intensities to ensure that outliers did not confound the statistical analysis.

### Statistical Analysis & Modeling

#### Linear mixed effects models

Models were used to identify significant intervention-associated changes in fasting and AUC metabolite values. Variables were transformed to normality, as evaluated by the Anderson Darling test for normality, using variable-wise log and power transformations, implemented in the R statistical language v2.15.1 [28]. Mixed effects models, treating the intervention (pre- vs. post-) as the main effect and individual subjects as random effects, were constructed on transformed variables using the MIXED procedure in SAS for Windows Release 9.3 (Cary, NC). A false discovery rate associated with the multiple hypotheses testing was adjusted to allow for a maximum 5% probability (q = 0.05) of false positives [29]. Fasting and analyte AUC changes with intervention were also compared using paired t-test with subject data from completers for which data were available from both Test Week 1 and Test Week 2, and conclusions drawn did not differ from the mixed model approach that included all participants; thus, the latter is reported herein. Using the group differences and variance in the concentration of the plasma metabolite 2-HB derived from our prior studies in obese women [8], and assuming an 80% power to detect a mean group difference equal to the SD, a power calculation indicated that our study design of 12 completing subjects was acceptable.

#### Partial least squares discriminant analysis

PLS-DA was implemented in imDEV 1.4.2 [30] and R, package pls [31]. PLS-DA modeling was used to identify optimal metabolic fasting and AUC classifiers between pre- or post-intervention conditions. An independent PLS model was developed on intervention adjusted data to identify metabolic changes correlated with time during the OGTT. Models were calculated using autoscaled data, leave-one-out cross-validation and their parameters used to identify intervention-associated and OGTT-responsive multivariate metabolic effects. A PLS-DA model (“combined model”) was also constructed using factors derived from the overnight-fasted model and the OGTT-AUC model. Metabolites were retained if their model weights differed significantly from the mean parameter weight at p<0.05 using a one-sample t-Test. Positive and negative coefficients were tested separately. Permutation tests (n = 100) were used to compare the optimized (n = 48) and all parameter (n = 648) models' performance statistics to their respective random NULL distributions. Distributions for the model cross-validated fit on training data (Q2), root mean squared error of prediction on test data (RMSEP), and area under the receiver operator characteristic curve (AUROC) (n = 100) by randomly splitting the samples between (2/3) training and (1/3) test sets. Statistical distributions for permuted models' performance measures were calculated using identical procedures, in addition to permutation of the dependent variable or samples pre- or post-intervention assignment.

#### OGTT-responsive metabolic chemical similarity network

A chemical similarity network was used to map and visualize OGTT-associated changes in metabolite levels within a biological context. In this network, vertices represent metabolites that are connected by edges (lines) based on chemical similarity (Tanimoto similarity >0.7) calculated in R using the package ChemmineR [32]. In this network vertex size is used to encode the absolute value of the PLS-DA latent variable 1 (LV1) model loading (importance/magnitude of metabolic change during the OGTT) and vertex color the sign of the LV 1 loading or direction of metabolic change during the OGTT (decrease, blue; increase, red).

## Results

### Body Mass loss and improvements in metabolic health indices following a fitness and dieting intervention

**Figure 1:**
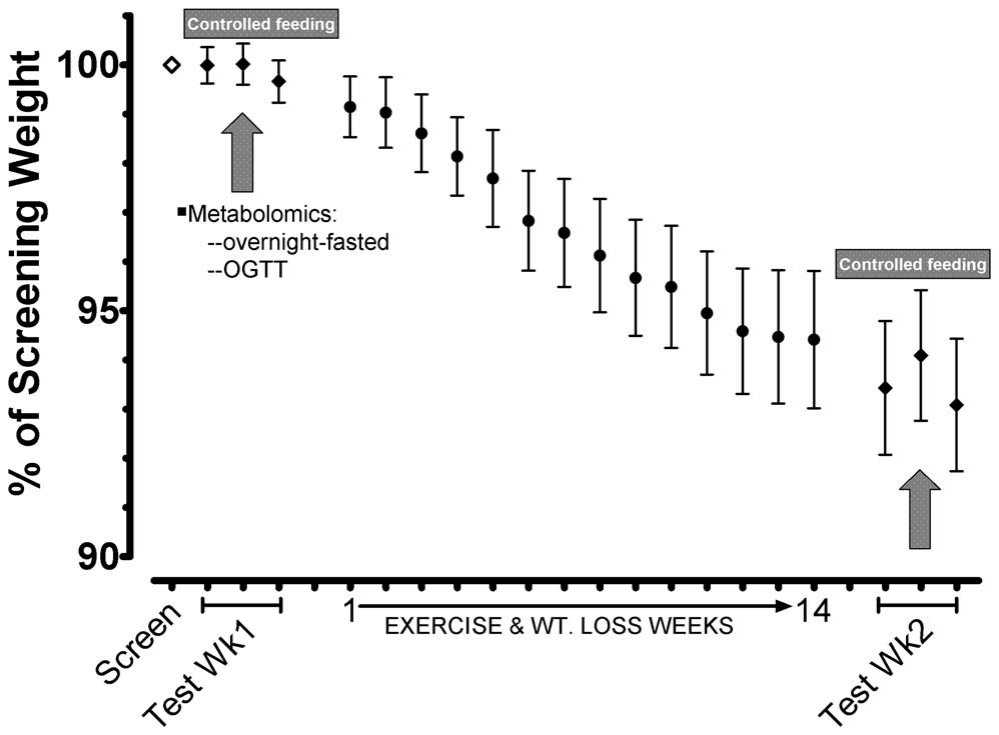
Study schematic and weight loss patterns in obese, sedentary women undergoing a fitness and weight loss intervention to assess changes in the plasma metabolome. Blood was drawn in the overnight-fasted and post-OGTT conditions, during each of pre-intervention and post-intervention Test Weeks identically controlled for dietary intake, weight maintenance, and physical activity. To match menstrual cycle stage within individuals, Test Week 2 was after an intervention period ranging from 14-17 weeks (14 week time point mean is depicted for clarity). Of 15 women completing Test Week 1, 12 remained throughout the study to finish Test Week 2.

Of the 55 overweight to obese sedentary women initially screened using clinical blood lab results, 18 (36%) presented with insulin resistance as defined by our criteria (**Figure S1**). **Figure 1** highlights the successful body mass maintenance achieved in Test Weeks 1 and 2 that preceded and followed, respectively, the weight loss intervention phase. Results for body mass, body fat, insulin resistance indices, and other phenotype variables for the 15 compliant subjects enrolled in the study are provided in **Table 1**. As expected, there was a significant reduction in body mass and body fat during the intervention, and markedly improved insulin sensitivity as indicated by increased Matsuda index. Although there is no consensus Matsuda index (a.k.a. “Composite Index”) indicative of insulin resistance or diabetic risk, a value of <2.5 has been used to identify insulin-resistant subjects in a cross-sectional cohort of normal glucose tolerant adults (representing the lowest ∼20–33% of insulin sensitivity in the population; Ref. [33]). Similarly, obese subjects considered “at risk” for metabolic disease had a Matsuda index <2.5 [34], [35]. Fitness was significantly increased by intervention as indicated by higher VO_2peak_ and increased maximal work on a cycle ergometer (**Table 1**). Directional changes in fitness and glucose sensitivity parameters were the same even if just examining the subset of 12 women completing both Test Week 1 and Test Week 2 (**File S2**: Supplemental Materials 2). There were no significant differences in pre- vs. post-intervention serum FSH or LH concentrations in subjects completing both Tests Weeks in the protocol and neither hormone was correlated with QUICKI or the Matsuda index at either time point (data not shown). As anticipated, FSH and LH concentrations were tightly correlated (e.g., r = 0.816 and r = 0.705 in Test Weeks 1 and 2, respectively, p≤0.01).

**Table 1:** Body mass, fitness, and glucose homeostasis indices in previously sedentary obese women following a weight loss and fitness intervention.

	Pre-intervention^a^	Post-intervention^b^	p-value Mixed Model^c^
Age	40.4±5.2 (median, 38.8)	–	–
Body Mass (kg)	88.6±2.1	83.0±3.1	<0.0001
BMI, kg/m^2^	33.5±0.6	31.0±0.9	0.0007
Body Fat%	47.5±1.0	43.4±1.5	<0.0001
Fat Mass (kg)	41.9±1.6	36.2±2.4	0.0001
Fat Free Mass (FFM, kg)	43.1±0.8	43.3±0.9	NS
VO_2_peak (mL/kg/min)	21.1±1.1	25.6±1.1	<0.0001
VO_2_peak (mL/kg FFM/min)	43.7±1.6	49.4±1.4	<0.0001
Maximal Power (Watts at VO_2_peak)	142.0±3.8	166.7±6.4	<0.0001
Fasting Glucose (mg/dL)	89.1±1.5	84.6±1.5	0.0159
Fasting Insulin (µU/mL)	18.5±2.1	13.7±1.7	0.0092
Matsuda Index	1.97±0.17	2.91±0.36	0.0076
QUICKI	0.315±0.004	0.332±0.006	0.0046

^a^ pre-diet and exercise intervention, n = 15 (self-reported ethnicity, n: White, 9; Hispanic, 3; Black, 1; Asian, 1; Other, 1).

^b^ post-diet and exercise intervention, n = 12.

^c^ comparisons using paired t-tests from subjects completing both Test Week 1 (pre-intervention) and Test Week 2 (post-intervention) yielded identical statistical patterns.

Values are means ± SEM; NS  =  not statistically significant.

### OGTT-associated changes in plasma metabolites

**Figure 2:**
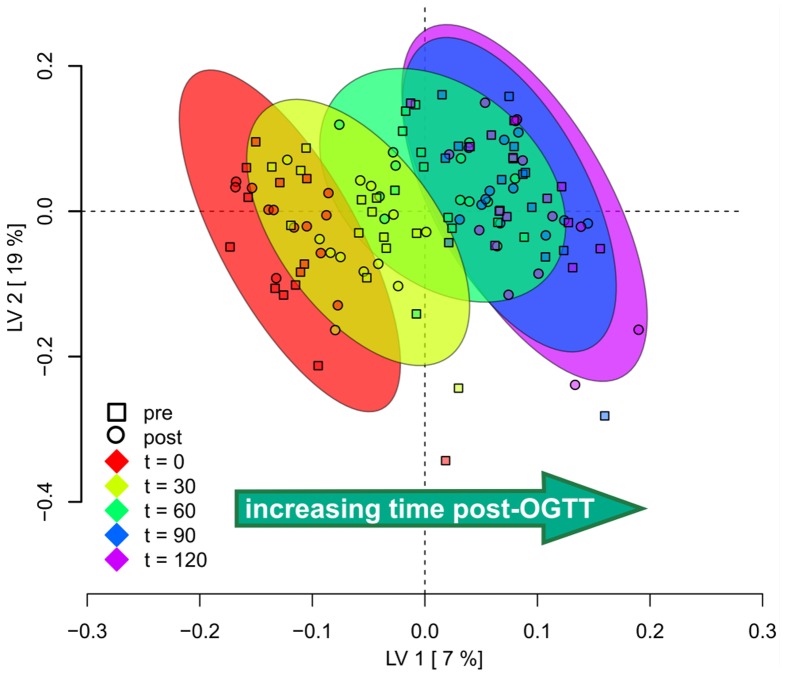
Subject Scores Plot derived from a partial least squares discriminate analysis (PLS-DA) model using temporal variance in identified plasma metabolites over the course of an OGTT in women. Each time point is represented by a different colored oval surrounding subject-time point groupings (see legend), and the score of each subject is represented as a symbol within time point cluster. Both pre- and post-intervention data were used to generate this plot (see Results). Note that variation in metabolite levels at each of the time points led to separation (discrimination) of clusters from one another, with the exception of the final 2 time points for which subject scores were similar.

Application of PLS-DA modeling using all identified metabolites (named with PubChem Compound Identifier [CID]) enabled discrimination of time-associated metabolic phenotypes in subjects as illustrated in **Figure 2**. A clear left-to-right shift along the X axis (latent variable dimension 1, LV1) was evident for all subjects, indicating a metabolic trajectory common to all subjects following the OGTT. Covariate adjustment for the effect of the weight loss and fitness intervention, done prior to modeling, enabled visualization of post-OGTT-associated time course metabolite patterns that were shared in both the pre-intervention and post-intervention states. Put differently, OGTT impacted general metabolite patterns in a qualitatively similar way under both pre- and post-intervention conditions for the majority of metabolites. Variance in plasma metabolite levels did not discriminate the 90 and 120 min post-OGTT time points (see overlapping ovals) indicating that the most dynamic metabolite changes took place between 0–90 min.

**Figure 3:**
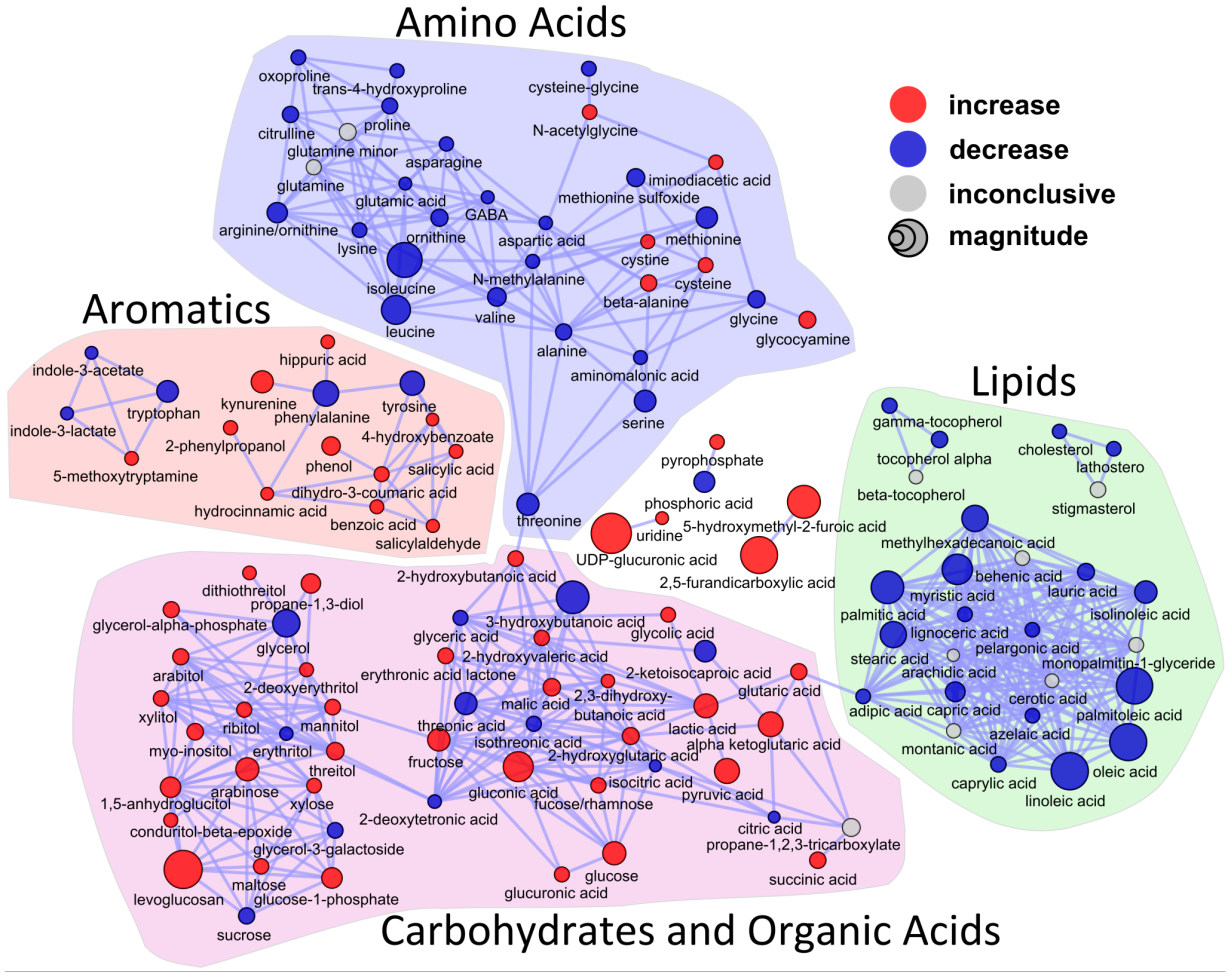
A chemical similarity network of identified metabolites was used to visualize OGTT-associated changes in metabolite levels following an OGTT in women. Vertices represent metabolites that are connected by edges (lines) based on chemical similarity (Tanimoto similarity >0.7). Loadings on the first latent variable in the PLS model for metabolic changes correlated with time during the OGTT are mapped to vertex size (absolute loading) and color is used to display the direction of the change (sign of loading: red, increase; blue, decrease; gray, unclear change or differential change in pre- vs. post-intervention conditions).

To gain insight into OGTT-associated trends in broad classes of plasma metabolites, a chemical similarity network of identified metabolites was constructed using data from both the pre- and post-intervention phases and all OGTT time points (**Figure 3**). From this map, it is clear that the OGTT triggered the following general shifts in blood.

#### Lipid Metabolism

A reduction in most lipid class members was observed, including free fatty acids, glycerol, and 3-hydroxybutyrate (β-hydroxybutyrate). This is consistent with an expected reductions in lipolysis and ketogenesis following the post-OGTT insulin surge. Medium-chain length dicarboxylic acids adipic (di-C6) and azaelic (di-C9), and medium-chain fatty acids caprylic (C8∶0) and pelargonic (C9∶0) were decreased in plasma with the OGTT, likely due to reductions in peroxisomal ω-oxidation and β-oxidation. A suite of very long chain fatty acids (arachidic [C20∶0], behenic [C22∶0], lignoceric [C24∶0], cerotic [C26∶0], montanic [C28∶0]) displayed mixed patterns of plasma concentrations post-OGTT, depending upon intervention phase. In the pre-intervention phase, post-OGTT plasma concentrations of these VLCFAs were stable or fell whereas levels tended to rise in the post-intervention phase (see results below). These results suggest that intervention led to alterations saturated long-chain fatty acid (LCFA) elongation in the course of the OGTT.

#### Amino Acid Metabolism

With OGTT there was a decrease in plasma amino acid concentrations, with a concurrent increase in several measured downstream catabolism products, e.g. cysteine-cystine (Met derivatives), 2-HB (Met or Thr derivative), 2-ketoisocaproic acid (Leu metabolite), kynurenine and 5-methoxytryptamine (Trp products). These patterns are consistent with an effect of insulin to promote amino acid uptake and tissue utilization.

#### Carbohydrate Metabolism

As expected, there were OGTT-associated increases in glucose and glucose-derived metabolites, e.g. glucose-1-phosphate, fructose, lactate, pyruvate, UDP-glucuronic acid (UDP-glucose derivative) and a rise in organic acids including TCA cycle intermediates. This highlights the anticipated rise in glucose oxidative catabolism and increased engagement of minor pathways of glucose conversion (e.g., to fructose via the sorbitol pathway).

#### Splanchnic Metabolites

We observed a modest decrease in cholesterol and the cholesterol precursor lathosterol with the OGTT. In addition, in the post-intervention phase the OGTT elicited an increase in plasma gut microbial metabolite propane-1,2,3-tricarboxylate (a.k.a. tricarballylic acid; discussed in more detail below).

**Figure 3** also illustrates post-OGTT increases in plasma concentrations of several adulterants we subsequently identified in the commercial OGTT solutions. For instance, there was a large increase in levoglucosan, a molecule produced upon heating of carbohydrates including glucose. There was no levoglucosan detected following injection of a derivatized pure glucose standard into the GC-TOF instrument (data not shown), indicating that this molecule was not a by-product of the heating step of sample analysis. However, levoglucosan was readily detected in *post-hoc* analyses of all OGTT solutions, likely present due to heating of the dextrose syrup ingredient that occurs prior to compounding of the OGTT drinks. Post-OGTT increases in plasma 5-hydroxymethylfurfural (5-HMF) derivatives (5-hydroxymethyl-2-furoic acid [HMFA] and the HMFA metabolite 2,5-furandicarboxylic acid [FDCA]) were also detected. 5-HMF was detected in all of the OGTT solutions and is a molecule commonly found in heat-processed foods, emanating from dehydration of sugars. However, FDCA was not detected and HMFA was only observed in one of the OGTT solutions. The explanation for the relatively large increase in plasma HMFA and FDCA post-OGTT (**Figure 3**) is likely that these metabolites represent terminal products of 5-HMF, a molecule rapidly and completely metabolized (endogenous and microbial) following ingestion [36]–[39]. Notably, tricarballylic acid was not detected in the OGTT solutions, indicating it was not an adulterant of these preparations.

### Plasma metabolites altered in the overnight-fasted state by the fitness and weight loss regimen

We next sought to identify metabolites with systemic concentrations impacted by weight loss, improved insulin sensitivity and fitness (defined here as “metabolic health”). Metabolites with significant concentration changes comparing the overnight-fasted pre- and post-intervention states are presented in **Table 2**. The list includes equal numbers of known and as-yet unidentified metabolites and represents just 5.6% of all metabolites detected. Thus, the weight loss and fitness regimen resulted in only modest alterations in overnight-fasted concentrations in the metabolite classes detected by this analytical platform. Some trends observed from these overnight-fasted plasma metabolite data include: (1) Modest but statistically significant increases in the 5-HMF derivatives HMFA and FDCA, as well as a modest but significant decrease in food-derived 1,5-anhydroglucitol. Thus, even in the overnight-fasted state and under highly-controlled dietary conditions, there were subtle intervention-associated shifts in levels of blood metabolites that originate from diet and microbial metabolism. (2) A 40% reduction in the concentration of the low-abundance metabolite α-ketoglutarate, ∼32% lower level of the glutamate derivative gamma-aminobutyric acid (GABA), and significantly reduced pyruvic acid and uric acid. (3) A ∼30% reduced concentration of behenic (C22∶0) fatty acid. Concentrations and pre- vs. post-intervention comparisons for all detected metabolites are provided in **Table S1**.

**Table 2:** Overnight-fasted concentrations of identified and as-yet unidentified plasma metabolites^a^ that were significantly altered by a weight loss and fitness intervention in adult women.

	Pre-intervention^b^	Post-intervention^c^	Fold of Pre-intervention	p-value^d^
5-hydroxymethyl-2-furoic acid (HMFA)	172±21	200±11	1.2	0.0409
glycine	181000±11361	207000±13856	1.1	0.0321
2,5-furandicarboxylic acid (FDCA)	153±22	168±12	1.1	0.0341
1,5-anhydroglucitol	115000±8004	94800±5196	0.83	0.0281
BB369638	1260±83	1040±84	0.83	0.0398
uric acid	86500±5422	69500±4619	0.80	0.0005
BB314770	15800±1110	12300±779	0.78	0.0435
BB225396	2040±527	1580±254	0.77	0.0230
BB402237	3210±181	2380±687	0.74	0.0045
γ-tocopherol	2110±219	1540±254	0.73	0.0196
behenic acid	1470±207	1060±95	0.72	0.0407
BB226851	1260±139	912±104	0.72	0.0054
BB288808	727±300	518±38	0.71	0.0372
GABA	5180±620	3520±520	0.68	0.0143
pyruvic acid	5040±775	3400±866	0.67	0.0458
BB223513	2990±310	1970±225	0.66	0.0012
BB228147	947±108	603±58	0.64	0.0175
α-ketoglutaric acid	804±88	480±75	0.60	0.0010
BB206309	32800±21430	7620±808	0.23	0.0429

^a^ metabolites detected by GC-TOF, values are in quantion peak heights; unidentified metabolites indicated with their BinBase (BB) nomenclatures; values are mean ± SEM; values for glucose and insulin are provided in Table 1.

^b^ pre-diet and exercise intervention, n = 15.

^c^ post-diet and exercise intervention, n = 12.

^d^ p-value for a mixed effects linear model (*no comparison met the 5% significance criteria after false discovery rate correction at a q = 0.05).

### Plasma metabolites with post-OGTT concentration excursions (AUCs) altered by the fitness and weight loss regimen

**Figure 4:**
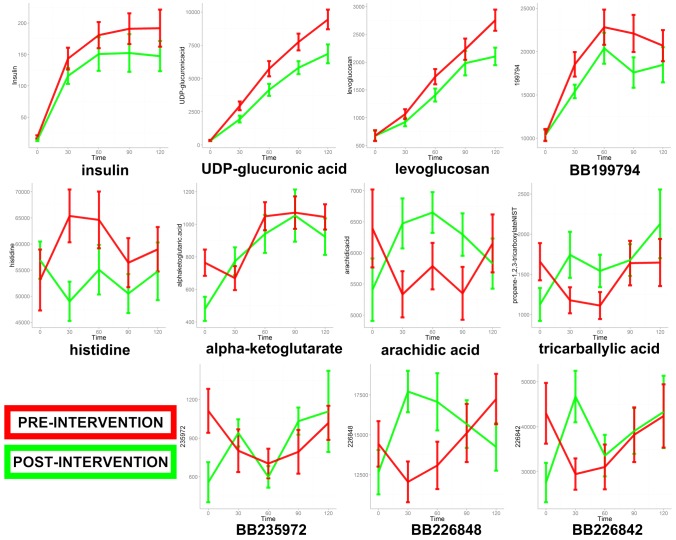
Post-OGTT excursions in plasma concentrations of metabolites that displayed a significant difference in area-under-the-curve (AUC) when comparing the pre- and post-intervention phases (red and green lines, respectively) of a weight loss and fitness regimen in women. Values are quantion peak heights for each of the individual metabolites.

**Figure 5:**
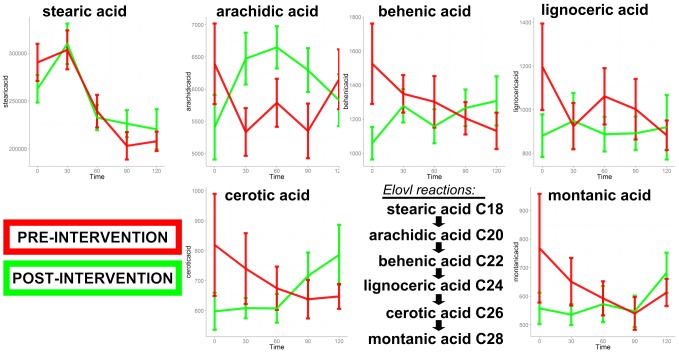
Post-OGTT excursions in plasma concentrations of very long chain fatty acid (VLCFA) metabolites that are products of the Elongation of very long chain fatty acid (Elovl) enzymes. Illustrated are temporal changes in metabolites when comparing the pre- and post-intervention phases (red and green lines, respectively) of a weight loss and fitness regimen in women. Values are quantion peak heights for each of the individual metabolites. Also shown is the set of reactions catalyzed by ELOVL including metabolites detected in this study.

Post-OGTT plasma AUCs of 95 metabolites differed significantly from zero at one or both intervention time points (one-sample t-test, p<0.05; equivalent to 95% confidence interval not overlapping with zero) (**Table S2**). Some metabolite AUCs were significantly different from zero in both pre- and post-intervention (38 metabolites), whereas changes in others were significantly altered by the OGTT only in the pre- (14 metabolites) or only in the post-intervention (43 metabolites) phases. Of these metabolites, 11 displayed statistically-significant differences comparing the pre- vs. post-intervention phases (**Table 3**). The glucose AUC was reduced following the intervention (**File S2**: Supplemental Materials 2, first metabolite AUC panel in series) but this did not reach statistical significance (p = 0.06). From this analysis, it is clear that the OGTT challenge successfully unmasked metabolic health-associated patterns that were not detected using fasting metabolite comparisons alone. Excursion plots of the Table 3 metabolites are depicted in **Figure 4**. From this figure it is clear that the AUC of α-ketoglutarate was significantly enhanced following the fitness and weight loss intervention, partly explained by a significantly lower time zero (overnight-fasted) concentration. Other unique patterns illustrated in **Figure 4** included an unexpected post-intervention rise in AUCs for arichidic acid (C20∶0) and the gut microbe-derived propane-1,2,3-tricarboxylate (tricarballylic acid). Arachidic acid is derived from Elongation of very long chain fatty acids (Elovl) enzyme activity using stearic acid (C18∶0) as substrate, and differential pre- and post-intervention AUC shifts patterns of other VLCFA Elovl products were qualitatively similar to arachidic (**Figure 5**). All detected metabolites' AUCs are provided in **Table S2**, and graphs for all metabolites' post-OGTT excursions are provided in **File S2** (Supplemental Materials 2).

**Table 3:** Post-OGTT excursions (AUC) of plasma identified and as-yet unidentified metabolites^a^ and serum insulin significantly changed by a weight loss and fitness intervention in adult women.

	Pre-intervention^b^	Post-intervention^c^	p-value^d^	AUC change
insulin	†16400±1969	†13300±2165	0.0006	↓
UDP-glucuronic acid	†599000±52696	†426000±43301	0.0006	↓
levoglucosan	†121000±11926	†90000±13279	0.0049	↓
BB199794	†1120000±141449	†793000±106810	0.0478	↓
histidine	902000±554700	†−516000±207846	0.0342	↓
α-ketoglutaric acid	†19200±7488	†46500±10681	0.0456	↑
arachidic acid	−84800±63790	†102000±43301	0.0088	↑
propane-1,2,3-tricarboxylate (tricarballylic acid)	−31400±27735	†62600±20207	0.0179	↑
BB235972	−32500±19414	†35200±15877	0.0197	↑
BB226848	−49000±188598	†398000±106810	0.0262	↑
BB226842	−914000±748845	†1330000±433012	0.0447	↑

^a^ metabolites measured by GC-TOF, AUC values are derived from quantion peak heights; unidentified metabolites indicated with their BinBase (BB) nomenclatures; values are mean ± SEM.

^b^ pre-diet and exercise intervention, n = 13.

^c^ post-diet and exercise intervention, n = 12.

^d^ p-value for a mixed effects linear model (no comparison met the 5% significance criteria after false discovery rate correction at a q = 0.05).

AUC significantly different from zero; not shown are metabolite AUCs that were significantly different by intervention period but that were not different from zero at either time point due to high variance (see **Results**).

### PLS-DA Model using overnight-fasted metabolite levels and post-OGTT AUCs to identify the most robust factors discriminating the pre- vs. post- fitness and weight loss intervention phenotypes

**Figure 6:**
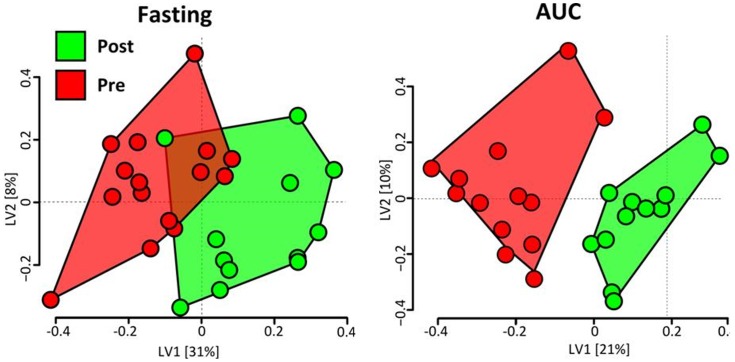
Subject Scores Plots derived from PLS-DA models using either overnight-fasted or post-OGTT (area-under-the-curve, AUC) metabolite variances, illustrating that differences in select metabolic features successfully discriminate subjects based on intervention phase. Each symbol represents the score for a single subject during the pre- or post-intervention phases (red and green, respectively). For both models, the best discrimination of phases was evident along the latent variable 1 axis (LV1). Note that one subject's score from the post-intervention phase overlapped with the scores cluster of the pre-intervention subjects, indicating similarity in fasted metabolite pattern with pre-intervention phase women (also see Results).

To determine the best combination of metabolic parameters indicative of an improvement in metabolic health, a combined model was generated that utilized fasting metabolite levels, AUCs, and clinical indices of insulin sensitivity (Matsuda index, QUICKI, fasted insulin and glucose). First, to build this combined PLS-DA model, feature selection was used to optimize fasting metabolites that best differentiate pre- and post-diet and fitness intervention (**Table 4**, 1^st^ column). Features were selected based on a filter approach [40] on variable coefficient weights [41](see **Methods**). PLS scores based on a model constructed from discriminating overnight-fasting analyte variables reasonably separated pre- and post-intervention subject groups as shown in the subject scores plot in **Figure 6A**. Second, AUCs for all analytes were similarly modeled, and feature selection was used to optimize independent AUC values (**Table 4**, 2^nd^ column) that best differentiated pre- and post-diet and fitness intervention phenotypes, as illustrated in the subject scores plot in **Figure 6B**.

**Table 4:** Plasma metabolite and endocrine parameters used to generate a combined PLS-DA model that best discriminates the pre- and post- weight loss and fitness intervention states in adult women.

Parameters found in the overnight-fasted PLS-DA model^a^	Parameters found in the AUC PLS-DA model^b^	Parameters found in both fasting & AUC models^c^	Factors added back for final combined model^d^
malic acid	arachidic acid	213961	oleamide AUC
288808	oleamide	281268	226851
228147	propane-1,2,3-tricarboxylate (tricarballylic acid)	α-ketoglutaric acid	HMFA
uridine	235972	γ-tocopherol	insulin
223513	295002	cystine	serine
402237	threonic acid	uric acid	glucose (clinical)
insulin	glutamine	211972	
217893	UDP-glucuronic acid	N-methylalanine	
glycine	stigmasterol		
lactic acid	*levoglucosan		
5-hydroxymethyl-2-furoic acid (HMFA)	226848		
serine			
226851			
arginine/ornithine			
glutamic acid			
pyruvic acid			
glucose (clinical)			

^a^ Parameters selected in the overnight-fasted plasma metabolite PLS-DA model.

^b^ Parameters selected in the post-OGTT plasma metabolite AUC PLS-DA model.

^c^ Parameters observed in both the overnight-fasted metabolite PLS-DA model and the AUC-only PLS-DA model.

^d^ Parameters not found initially in the combined model, but added back because of their presence in either the the overnight-fasted metabolite PLS-DA model or AUC-only model.

adulterant in OGTT solution (see **Results**).

**Figure 7:**
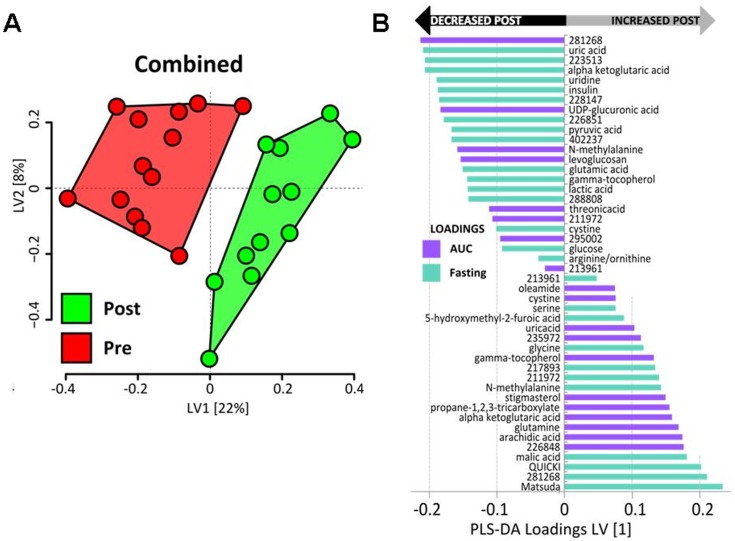
Subject Scores Plot (A) and Variable Loadings Plot (B) derived from a combined PLS-DA model using both overnight-fasted and post-OGTT (area-under-the-curve, AUC) metabolite variances. The model was calculated using a combination of the most robust metabolic features included in separate PLS-DA modeling of the fasted and post-OGTT states (Table 4). Each symbol in the Scores Plot represents the score for a single subject during the pre- or post-intervention phases (red and green, respectively). The best discrimination of phases was evident along the latent variable 1 axis (LV1), and the contribution of individual metabolic phenotype factor variance toward separation of the groups along LV1 is depicted in the Loadings Plot.

Finally, a combined model utilized both fasting and post-OGTT AUC parameters to identify all factors that discriminate the subjects based on pre- and post-intervention status (**Table 4**). **Figure 7A** depicts the PLS-DA subject scores plot output from this model showing separation of groups using this approach, and **Figure 7B** is a loadings plot indicating the most important features that best defined the pre- vs. post-intervention metabolic phenotype differences. These results indicate that improved metabolic health was defined by a subset of metabolites, including (as examples): (1) Changes in the AUC and/or fasting plasma levels of diet or gut microbe-derived molecules such as stigmasterol, propane-1,2,3-tricarboxylate (tricarballylic acid), furoic acid derivatives, and γ-tocopherol; (2) Shifts in plasma α-ketoglutarate, UDP-glucuronic acid, malate and pyruvate suggestive of changes in mitochondrial function and glucose oxidative and non-oxidative metabolisms; (3) Alterations in AUC of oleamide, an endocannabinoid; (4) Reduction in post-OGTT purine and pyrimidine metabolites uric acid and uridine.

### Metabolite and phenotype correlations in the pre- and post-intervention periods

An important aim of the current work was to identify metabolites that correlate with insulin sensitivity and blood sugar control. To this end, a Spearman's cross-correlation plot (CCP) for phenotype and metabolite variables was constructed to identify factors that associate with one another in the pre- and post-intervention phases (**Tables S3 and S4**). For clarity, this analysis was only conducted with factors having PLS-DA loadings values > +/−1 SD from the mean loadings values. As expected, the Matsuda insulin sensitivity index was strongly and negatively correlated with fasting plasma insulin and the QUICKI score (derived from fasting insulin and glucose), regardless of intervention phase (**Table 5**). In addition, 8 and 10 metabolites, respectively, were correlated with the Matsuda index in the pre- and post-intervention phases (**Table 5**). Of note, the only factor that correlated significantly in both phases was fasting plasma α-ketoglutaric acid (α-KG), as this metabolite's fasting plasma concentration was inversely correlated with insulin sensitivity. Xeno-metabolites directly or indirectly from diet also correlated with Matsuda index: e.g., FDCA and γ-tocopherol. Altogether, these results and other correlation patterns in **Tables S3 and S4** highlight that relationships between metabolite factors and insulin sensitivity are malleable with changes in fitness, weight, or other aspects that were altered by the study intervention.

**Table 5:** Correlations of interest between Matsuda insulin sensitivity index and fasting plasma or post-OGTT metabolite area-under-the-curve (AUC) in adult women either pre- or post-weight loss and fitness intervention.^a^

Pre-intervention^b^	Post-intervention^c^
insulin (−0.872)***	insulin (−0.804)**
QUICKI (0.923)***	QUICKI (0.839)***
α-ketoglutaric acid (−0.549)*	α-ketoglutaric acid (−0.776)**
BB199777 AUC (−0.654)*	BB235972 (0.769)**
BB199794 AUC (−0.560)*	BB299159 (0.573)*
BB213961 AUC (0.560)*	2,5-furandicarboxylic acid (0.651)*
cystine AUC (0.599)*	BB295002 AUC (0.811)*
γ-tocopherol AUC (0.654)*	BB299211 AUC (0.650)*
isolinoleic acid AUC (0.692)**	levoglucosan AUC (−0.720)**
salicylaldehyde AUC (0.637)*	palmitoleic acid AUC (0.650)*
	lysine (0.615)*
	pyruvic acid (−0.601)*

^a^ Contrasts and values are from **Tables S3 and S4**; Spearman's correlation r values are in parentheses; *p≤0.05, **p<0.01, ***p<0.001.

^b^ pre-diet and exercise intervention, n = 13.

^c^ post-diet and exercise intervention, n = 12.

## Discussion

A better understanding of the systemic metabolite changes that track insulin resistance and pre-diabetes could help lead to development of new prognostic biomarkers, and will inform on the basic physiological processes and tissue cross-talk events that are associated with metabolic status. Comprehensive metabolite profiling in the insulin-resistant and T2D states has highlighted that in addition to compromised glucose homeostasis, these conditions are associated with perturbations in fatty acid, amino acid, and bile acid metabolism [2]–[16]. Most metabolomics studies in this arena have focused on the overnight-fasted state or under non-controlled dietary conditions, but metabolic phenotypes could manifest more robustly under dynamic conditions that assess metabolic flexibility rather than single static measures [17]–[21]. The current study assessed, for the first time, changes in post-OGTT comprehensive metabolite patterns following a diet and fitness intervention that markedly improved metabolic health indices (weight loss, increased fitness, and improved insulin sensitivity). The three major findings are outlined below.

First, we made the novel observation that intervention led to alterations in circulating concentrations of gut-derived xeno-metabolites both in the fasted state and during a glucose challenge, suggesting that gut function and/or microbial metabolism were responsive to host metabolic health status. That gastrointestinal microbes generate gut and systemic short-chain fatty acids (SCFAs) has been long-appreciated, but it is increasingly clear from rodent and human models that many other microbe-derived metabolites make their way into the bloodstream (e.g. [42]–[49]). Current perspectives tend to emphasize the importance of the gut microbiome on driving obesity and dysfunctional metabolism in the host (reviewed in [50], [51]). However, the degree to which host insulin sensitivity, fitness, or adiposity impacts microbial ecology and metabolism of gut-derived xeno-metabolites is not as well established. Provision of oral glucose was associated with a significant post-OGTT change in plasma concentration of the citrate analog propane-1,2,3-tricarboxylate (tricarballylic acid), a pattern altered following the weight loss and fitness regimen. Tricarballylic acid is a product of gut microbial metabolism of food-derived trans-aconitate (or to a smaller degree cis-aconitate, citrate or isocitrate from host or diet), classically described in ruminants [52], [53]. Rat cecal bacteria, but not liver preparations, have been shown to produce tricarballylic acid from aconitate, and rat cecal bacteria showed no ability to degrade tricarballylic acid [54]. Altogether, the results in rats indicate that tricarballylic acid is a terminal xeno-metabolite made by mammalian gut microbes. Supporting a microbial origin of tricarballylic acid was the observation that its AUC displayed a strong inverse correlation with fasting plasma concentration of 2,3,5-trihydroxypyrazine (r = −0.643; p = 0.02 [pre] and r = −0.895; p<0.0001 [post]), a metabolite that has not been reported to be synthesized by mammals and is structurally related to plant- or pathogen-derived hydroxypyrazine metabolites. Thus, we speculate that fasting plasma 2,3,5-trihydroxypyrazine concentrations marked prevalence or metabolic activity of specific gut microbes that are associated with tricarballylic acid production. In addition to aconitate as precursor, we cannot exclude the possibility that microbes utilized the citrate additive contained in the OGTT solutions to produce tricarballylic acid. The mechanisms underlying the shift in tricarballylic acid post-OGTT plasma excursion upon improvement in metabolic health remain to be evaluated experimentally, but changes in gut tricarballylic acid production concomitant with altered microbial ecology may have occurred. Tricarballylic acid uptake transporters in the gut brush border, described as a Na^+^-dependent transporter shared with citrate, have been described for cattle [55] and likely played a role in glucose-responsive uptake of this organic acid in our human cohort. It remains to be seen if changes in blood levels of tricarballylic acid impact human host biology, but in an extreme example, rumen over-production of tricarballylic acid in cattle eating grasses high in trans-aconitate is implicated in inhibition of host tissue aconitase thought to contribute to grass tetany [52], [53]. However, results were equivocal in rat liver slices treated with tricarballylic acid at a concentration of as-yet unknown physiological relevance [54]. Microbial transformation of aconitate to tricarballylic acid strongly modified short-chain fatty acid (SCFA) production in artificial cultures [52], suggestive of another means by which host physiology could be impacted by changes in accumulation of this metabolite or the activities of its microbial producers.

Other examples of blood xeno-metabolites altered by the weight loss and fitness intervention included increased post-OGTT AUCs for γ-tocopherol and stigmasterol, and modest increases in fasting concentrations of FDCA and 5-HMFA, the terminal metabolites of the food-derived 5-HMF [36]–[39]. Also notable were intervention-associated reductions in fasting plasma γ-tocopherol and 1,5-anhydroglucitol. Recently, in an epidemiology study assessing environmental and dietary factors that increase the odds of developing T2D, fasting blood γ-tocopherol was one of the strongest and most consistent risk factors, although the reason for this observation is unknown [56]. In light of this, we propose that circulating γ-tocopherol in part reflects changes in gut biology that are influenced by metabolic health. With respect to the food-derived 1,5-anhydroglucitol, this metabolite fluctuates inversely with large increases in glucose concentration or with diet [57], yet these parameters were essentially stable when comparing the pre- and post-intervention phases herein. Since a highly-controlled feeding paradigm ensured that subjects in the current study ate identical foods during the pre- and post-intervention testing weeks, changes in tricarballylic acid and other xeno-metabolites may reflect innate shifts in the subjects' gut microbe ecology and host metabolism/uptake of gut-derived metabolites. Gut permeability is increased by high fat feeding, Metabolic Syndrome, obesity and diabetes in humans, and these conditions are often associated with an altered gut microbiome (reviewed in [50], [51]). Consistent with our perspective that host metabolic health can regulate xeno-metabolite exposure, Zhao et al. reported that hippuric acid, methylxanthine, methyluric acid, and 3-hydroxyhippuric acid were reduced in urine of impaired glucose tolerant (IGT) subjects [7]. In retrospect, we have also previously observed alterations in fasting plasma levels of putative xeno-metabolites or co-metabolites (those involving both host and microbial processing) in obese T2D compared to weight-matched non-diabetic African-American women (e.g., 3,6-anhydrogalactose, benzoic acid, benzylalcohol, cis-3,4-methylene-nonanoylcarnitine, cis-3,4-methylene-heptanoylcarnitine)[3], [8]. Thus, a major implication of our findings and those of Zhao et al. [7] is that changes in the host's metabolic health (insulin sensitivity, fitness, or adiposity) significantly regulates meal-associated (transient) and fasting (chronic) systemic exposure to xeno-metabolites.

A second major finding was that weight loss and fitness intervention increased post-OGTT plasma indices of saturated very long chain fatty acid (VLCFA) elongation, with adipose tissue ELOVL enzyme activity as a likely contributor. To our knowledge, such a pattern has not been described previously. The OGTT elicited an expected drop in the plasma concentration of a variety of LCFAs, including ELOVL substrate stearic acid and some VLCFAs, consistent with repression of lipolysis and promotion of net tissue fatty acid uptake in response to insulin. Unexpectedly, following intervention the post-OGTT plasma excursions of VLCFAs were generally increased, especially apparent for arachidic acid (C20∶0), suggesting that saturated fatty acid elongation was enhanced in response to insulin and glucose. Products of the ELOVL 1 and 3 enzyme reactions include the saturated VLCFAs found to be changed in the current study [58], making these enzymes potential candidates underlying the phenotype. One hint regarding a site of elongation of saturated VLCFA post-OGTT emerged from correlation analysis: Plasma arachidic acid AUC (and, to an extent, that of its downstream ELOVL product behenic acid) was strongly correlated with body fat (pre- and post-intervention r values ≥0.7, p≤0.01; **Tables S3–S4**). This points to adipose tissue as the likely site of fatty acid elongation to generate arachidic and behenic acids following OGTT in humans, and suggests that improved metabolic health enhances these pathways. This speculation will, of course, require experimental assessment. One possible explanation is that improved insulin sensitivity promoted adipose tissue glucose uptake, *de novo* fatty acid synthesis, and fatty acid sequestration (reduced lipolysis) thus promoting ELOVL substrate availability during the OGTT. Alternatively, it is worth considering that training/fitness-derived signals could have increased adipose ELOVL(s) expression: the Spiegelman lab recently described that a potential training-induced myokine, FNDC5 or irisin, can strongly trigger cultured murine adipocyte gene expression of ELOVL3 (a.k.a. Cig30, a classic marker of activated brown adipocytes) [59].

A third important finding was that the post-weight loss and fitness intervention phenotype was marked by significant alterations in blood organic acids, most notably α-ketoglutaric acid (α-KG), malate, and pyruvate. One possible explanation is that improved metabolic health altered mitochondrial function (e.g., anaplerotic/cataplerotic balance) and/or transamination reactions. A novel finding was that fasting and post-OGTT plasma α-KG concentrations were major discriminating variables when comparing the pre-intervention phase (insulin-resistant, sedentary obesity) and post-intervention phase (improved insulin sensitivity and physical fitness). Fasting plasma α-KG was strongly and inversely correlated with the Matsuda index, indicating that improved insulin sensitivity reduced α-KG production and transport to plasma, and/or increased α-KG clearance in the basal state. One intriguing possibility is that fasting plasma α-KG was reflective of improved post-intervention LCFA β-oxidation efficiency and TCA cycle activity. Mitochondrial α-KG export (cataplerosis) is coincident with high mitochondrial LCFA availability accompanied by incomplete β-oxidation [60], [61] and at least for muscle, exercise interventions improve mitochondrial oxidative capacity [62] and reduce incomplete LCFA β-oxidation [63]. We observed increased fasting plasma malic acid (malate) and decreased pyruvic acid (pyruvate) as discriminating variables for the post-intervention phenotype, and post-intervention plasma malic acid was inversely correlated with pyruvate (r = −0.78, p<0.01). Such a pattern is consistent with the idea that TCA cycle capacity and more efficient glucose/pyruvate oxidation are associated with improved metabolic health. Supportive of these views was our observation that post-OGTT UDP-glucuronic acid AUC was significantly reduced by the fitness and weight loss intervention, suggestive of diminished non-oxidative metabolism of glucose. Furthermore, following the weight loss and fitness intervention, the post-OGTT α-KG excursion was significantly increased, and post-OGTT AUC trajectories for other TCA intermediates (e.g., isocitrate, fumarate, malate) also appeared higher post-intervention (see **File S2**: Supplemental Materials 2). Such results would be anticipated with greater post-OGTT glucose carbon flux into the TCA cycle upon improvements of insulin sensitivity and mitochondrial function, and may explain why a higher glutamine excursion post-intervention was also a discriminating factor in the PLS-DA model. Mitochondrial α-KG/glutamate/glutamine are in equilibrium through bi-directional mitochondrial conversions [64], [65]. Considering the central role of α-KG in aminotransferase reactions or metabolite synthesis (i.e., carnitine synthesis from butyrobetaine), we cannot exclude that pre- vs. post-intervention patterns of this metabolite were impacted by these routes. Notably, fasting glutamic acid (the transaminase partner with α-KG) was also reduced following intervention and was an important discriminating factor in the PLS-DA model. Also, one cannot ignore the potential effect of gut microbial metabolism of organic acids [48], which may have impacted intervention-associated blood metabolites. These possibilities will require experimental evaluation of organic acid kinetics and microbial metabolism across a range of insulin sensitivities and fitness levels. Regardless, our results indicate that measurement of blood α-KG or its ratio with other discriminating variables could be used to track insulin sensitivity and metabolic health status.

As a final point, we anticipated that improvements in insulin resistance, weight loss and fitness would alter recently-described metabolite biomarkers of insulin sensitivity and diabetes risk including branched chain amino acids (BCAAs), phenylalanine, tyrosine, and the methionine/threonine derivative 2-HB (reviewed in [66]). However, in the current study plasma concentrations of these amino acids, their derivatives, and their post-OGTT reductions (AUCs) did not correlate with marked improvements of insulin sensitivity. Similarly, in a small cross-sectional cohort, Ramos-Roman et al. did not detect associations between post-meal insulin sensitivity indices and patterns of BCAA-associated carnitine derivatives [67]. In contrast, using multivariate analysis, fasting blood BCAA or derivatives contributed to principal components that correlated with insulin sensivity in a dieting trial [68], although changes in BCAA were less apparent in a separate study comparing against bariatric surgery weight loss [11]. In a cross-sectional study comparing a large cohort with broader differences in metabolic phenotypes, Shaham et al. demonstrated that a higher fasting insulin concentration was associated with blunted post-OGTT reductions in blood leucine/isoleucine, suggestive of lower insulin-stimulated tissue BCAA utilization with insulin resistance [17]. LaFerrere and colleagues reported that blood BCAA tracked insulin resistance indices regardless of weight loss [11]. Differences in outcomes between those reports and ours may stem from our use of a controlled feeding paradigm prior to blood sampling, and fitness improvement coupled to weight loss. Regardless, we conclude that blood BCAA and 2-HB do not always track changes in insulin resistance status in humans, and the associations may be context-specific.

Other reported markers of insulin resistance or T2D primarily from cross-sectional studies include increased fasting blood or urine stearoyl-CoA desaturase-derived fatty acids, glucose derivatives and gluconeogenic precursors, bile acids or derivatives, cystine-cysteine (e.g., [5], [7], [8], [15], [16], [69], [70]), and decreased glycine, glutamine, L-GPC, and arachidonic acid (e.g., [5], [7]–[9], [13], [15], [16], [71]). Under conditions of a glucose challenge, individuals with insulin resistance had a blunted response in terms of blood lactate, bile acid or TCA-associated organic acid increases and glycerol and β-hydroxybutyrate decreases [17], [20]). Consistent with these studies, we observed that improved metabolic health and weight loss led to lower fasting lactate and pyruvate (possibly reflective of more efficient oxidative metabolism of glucose), reduced cystine (potentially indicating lower oxidative stress), and higher fasting plasma glycine (unknown etiology, but possibly related to reduced glycine conjugation load with more efficient metabolism). Previously, we reported uridine (a component of RNA metabolism, minor sugar derivatives, or diet) was elevated in T2D [8], and in the current study fasting plasma levels of this metabolite were reduced post-intervention and helped discriminate the pre- vs. post-intervention conditions. Thus, the directionalities of many metabolites in the current study are consistent with expectations from prior reports. The specific origins of these patterns and their connections with metabolic health phenotypes require further study.

### Strengths, Limitations and Future Directions

There were several limitations of the study. A select number of plasma metabolite patterns were shifted with improved fitness, weight loss, and insulin sensitivity, but this cannot confirm phenotype cause-and-effect or metabolite site(s) of origin, especially since fecal samples were not collected. Furthermore, in the current study design, one cannot deconvolute the main driver(s) of the blood metabolite changes, e.g. weight loss and fitness vs. insulin sensitivity. Strengths of the study included minimization of variance through the use of a narrowly-defined cohort of women, dynamic measurement of metabolites (post-OGTT, pre- and post-intervention), and a blood sampling regimen comparing pre- and post-intervention test weeks that were precisely-matched for diet, physical activity levels, weight-maintenance and menstrual cycle phase. The results highlight the importance of using uniform dietary and challenge test conditions when interrogating biological models with highly-sensitive metabolite analysis platforms. We do acknowledge, however, that interpretations are limited to the current subject population and conditions, and whether or not they apply to the broader population will require further validation studies. With respect to the observed xeno-metabolite patterns, one may ask, “What are the host signals emanating from improved insulin sensitivity, fitness or adiposity that influence gut function, microbial ecology, and concomitant changes in xeno-metabolite kinetics?” That we observed significant blood fasting and post-OGTT xeno-metabolite changes following a weight loss and fitness regimen under well-matched diet sampling conditions emphasizes the critical importance of signals sensitive to host metabolic physiology that direct the gut environment.

## Supporting Information

Figure S1 **Schematic of screening and recruitment of sedentary, obese insulin-resistant women for the weight loss and fitness intervention study.**
(PDF)

Table S1 **Pre- vs. post-intervention overnight-fasting plasma metabolite simple comparisons, ranked by magnitude of change.**
(XLS)

Table S2 **Pre- vs. Post-intervention area-under-the-curve (AUC) following an oral glucose tolerance test.**
(XLS)

Table S3 **Pre-intervention Spearman's correlation coefficient comparisons across analytes and phenotype variables.**
(XLS)

Table S4 **Post-intervention Spearman's correlation coefficient comparisons across analytes and phenotype variables.**
(XLS)

File S1 **Diet nutrient summary, menu components used in each of the Test Weeks, and food diary example.**
(PDF)

File S2 **Plasma concentration excursions for plasma analytes derived from metabolomics studies.** Concentrations were tracked following a standard 75 g oral glucose tolerance test prior to (Pre-) and after (Post-) weight loss and fitness intervention in sedentary obese insulin-resistant womens. Mean±SEM are shown for n = 15 (Pre) and n = 12 (Post).
(PDF)
