# Correction: Improved Metabolic Health Alters Host Metabolism in Parallel with Changes in Systemic Xeno-Metabolites of Gut Origin

**DOI:** 10.1371/journal.pone.0091258

**Published:** 2014-02-27

**Authors:** The PLOS ONE Staff

The name of the 17th author is incorrect. The correct name is: Kohei Takeuchi.
