# Supplementary material for: Improved Metabolic Health Alters Host Metabolism in Parallel with Changes in Systemic Xeno-Metabolites of Gut Origin
Source: PLoS One. 2014 Jan 8;9(1):e84260. doi: 10.1371/journal.pone.0084260 (PMC3885560; doi:10.1371/journal.pone.0084260)
Supplement: Figure S1 — Schematic of screening and recruitment of sedentary, obese insulin-resistant women for the weight loss and fitness intervention study. (PDF) [file pone.0084260.s001.pdf]

SUPPLEMENTAL FIGURE 1 (Campbell et al.)**.** Schematic of screening and recruitment of sedentary, obese, insulin-resistant women for the weight loss and fitness intervention study.

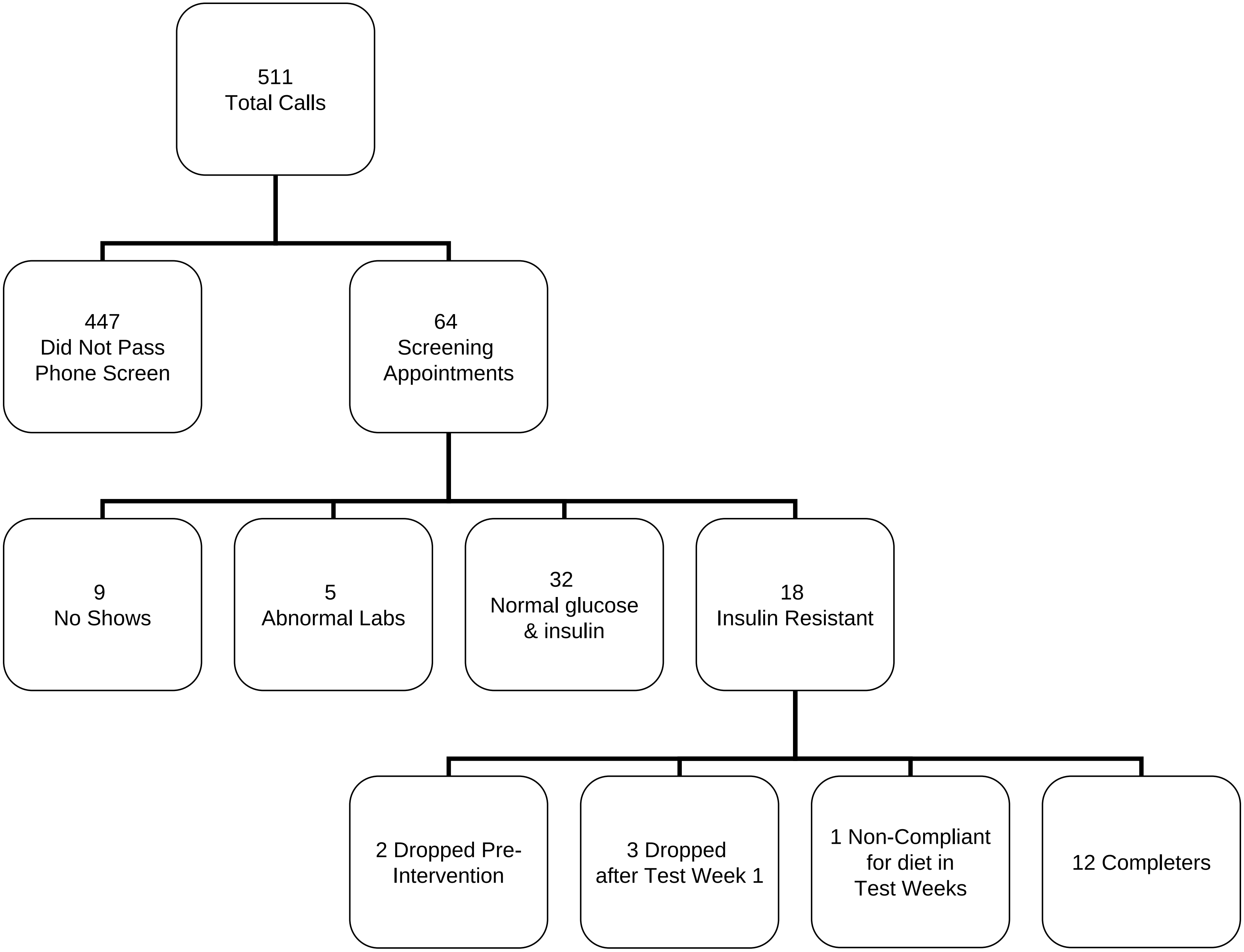

SUPPLEMENTAL FIGURE 1
