# Supplementary material for: Improved Metabolic Health Alters Host Metabolism in Parallel with Changes in Systemic Xeno-Metabolites of Gut Origin
Source: PLoS One. 2014 Jan 8;9(1):e84260. doi: 10.1371/journal.pone.0084260 (PMC3885560; doi:10.1371/journal.pone.0084260)
Supplement: File S1 — Diet nutrient summary, menu components used in each of the Test Weeks, and food diary example. (PDF) [file pone.0084260.s006.pdf]

SUPPLEMENTAL MATERIALS 1 (Campbell et al.). Diet nutrient summary, menu components used in each of the Test Weeks, and food diary example.

SUPPLEMENTAL MATERIALS:

Calculated Nutrient Values of Provided Menus During Test Weeks Coinciding with Metabolomics Measures

		Calculated			
**Analyte**	**Unit**	**Weeks1+2**	**Week1**	**Week2**	**Recommendations****
			**Mean**	**Mean**	
Days of feeding	n	162	81	81	
Energy	kcals	2088	2109	2066	2000
Fat	grams	69.5	70.2	68.8	
Carbohydrate	grams	301.4	304.1	298.5	130 (RDA/AI)
Protein	grams	82.9	84	81.8	46 (RDA/AI)
Fat	% of kcals	28.8%	28.8%	28.8%	25%-35% of total kcals (AMDR)
Carbohydrate	% of kcals	55.8%	55.8%	55.9%	45%-65% of total kcals (AMDR)
Protein	% of kcals	15.3%	15.4%	15.2%	10%-35% of total kcals (AMDR)
Cholesterol	milligrams	80.3	81.1	79.5	<300 (DG)
SFA	grams	17.9	18.1	17.6	
SFA	% of kcals	7.4%	7.4%	7.3%	<10% of total kcals
MUFA	grams	28.3	28.4	28.3	
MUFA	% of kcals	11.8%	11.7%	11.9%	
PUFA	grams	18.7	18.9	18.4	
PUFA	% of kcals	7.7%	7.8%	7.7%	
PUFA 18:2 (linoleic)	grams	15.7	15.9	15.4	12
PUFA 18:2 (linoleic)	% of kcals	6.8%	6.8%	6.7%	5%-10% of total kcals
PUFA 18:3 (alpha-linoleic)	grams	2.7	2.8	2.7	1.1
PUFA 18:3 (alpha-linoleic)	% of kcals	1.2%	1.2%	1.2%	0.6%-1.2% of total kcals
TFA	grams	0.8	0.8	0.8	
TFA	% of kcals	0.3%	0.3%	0.3%	
Fiber_total	grams	31.2	31.5	30.9	25
Fiber total	g/1,000 kcals	14.9	14.9	15.0	14
Fiber_Sol	grams	7.6	7.7	7.6	
Fiber_Insol	grams	23.5	23.8	23.3	
Sodium	milligrams	2931.5	2972.4	2888.6	<2,300
Potassium	milligrams	3519.4	3567.5	3468.9	4,700

Note: The recommendations set forth by the Dietary Guidelines for Americans are adapted from the Dietary Reference Intake (DRI) reports that are established by the Institute of Medicine (IOM). ** Abbreviations: RDA/AI = Recommended Dietary Allowances / Adequate Intake; AMDR = Acceptable Macronutrient Distribution Ranges

FL68 / Menu 1K (2100) Weigh Back

MENU 1 COMPONENTS, TEST WEEKS 1&2

Name: Composites - Set 1, FL68 **Date: 01/23/13**

SUPPLEMENTS

Food Item Description	Weight (g)	Amount	Measure	Prepared	Amt. Left	
Multivitamin (Bayer; One-a-Day for Women; Production Lot # = 238977-01; Expiration Date = 09/11)/FL68	5.00	1.00	each			

BREAKFAST

Food Item Description	Weight (g)	Amount	Measure	Prepared	Amt. Left	
Oatmeal, instant, dry (Quaker; CA-SUPC=6286025; AL-SUPC=6286025)/FL68	28.35	1.00	pc			
Oatmeal, instant, dry (Quaker; CA-SUPC=6286025; AL-SUPC=6286025)/FL68	28.35	1.00	pc			
Wheat Germ, toasted (Quaker/Mother's; www.quakeroats.elsstore.com; UPC=03000021638)/FL68	19.70	19.70	gram			
Milk, nonfat, lactose-free (CA-Dairy Ease; CA-SUPC=9016759; AL-Lactaid; AL-SUPC=9582875)/FL68	246.00	1.00	pc			
Raisins, seedless (Sysco Imperial; CA-SUPC=9387317; AL-SUPC=9387317)/FL68	43.00	43.00	gram			
Margarine, (Smart Balance Omega; CA-SUPC=6631347; AL-SUPC=6631347)/FL68	5.00	1.00	pc			
Margarine, (Smart Balance Omega; CA-SUPC=6631347; AL-SUPC=6631347)/FL68	5.00	1.00	pc			
Margarine, (Smart Balance Omega; CA-SUPC=6631347; AL-SUPC=6631347)/FL68	5.00	1.00	pc			
Margarine, (Smart Balance Omega; CA-SUPC=6631347; AL-SUPC=6631347)/FL68	5.00	1.00	pc			
Honey (CA-House Recipe; CA-SUPC=3530128; AL-FlavorFresh; AL-SUPC=6892495)/FL68	12.00	1.00	pc			
Bread, 100% whole-wheat, (CA-Hi-Vibe; CA-SUPC=7819846; AL-local vendor; Brand Name=???????)/FL68	14.18	14.18	gram			
Water, bottled (CA-Crystal Geyser; CA-SUPC=3199056; AL-AlpineSprings; AL-SUPC=5179569)/FL68	500.00	1.00	pc			
Water, bottled (CA-Crystal Geyser; CA-SUPC=3199056; AL-AlpineSprings; AL-SUPC=5179569)/FL68	500.00	1.00	pc			

LUNCH

Food Item Description	Weight (g)	Amount	Measure	Prepared	Amt. Left	
Soup, chicken noodle, low sodium, canned, heat serve (Campbell's; CA-SUPC=4104469; AL-SUPC=4104469)/FL68	205.50	1.00	pc			
Soup, chicken noodle, low sodium, canned, heat serve (Campbell's; CA-SUPC=4104469; AL-SUPC=4104469)/FL68	205.50	1.00	pc			
Bread, 100% whole-wheat, (CA-Hi-Vibe; CA-SUPC=7819846; AL-local vendor; Brand Name=???????)/FL68	42.00	42.00	gram			
Margarine, (Smart Balance Omega; CA-SUPC=6631347; AL-SUPC=6631347)/FL68	5.00	1.00	pc			
Salt (CA-Sysco Classic; CA-SUPC=4002994; AL-Diacrys; AL-SUPC=4582508)/FL68	0.10	1.00	pc			
Salt (CA-Sysco Classic; CA-SUPC=4002994; AL-Diacrys; AL-SUPC=4582508)/FL68	0.10	1.00	pc			
Pepper, black, ground (CA-ImpSysco/McCormick; CA-SUPC=5229299; AL-Diacrys; AL-SUPC=4903787)/FL68	0.10	1.00	pc			
Pepper, black, ground (CA-ImpSysco/McCormick; CA-SUPC=5229299; AL-Diacrys; AL-SUPC=4903787)/FL68	0.10	1.00	pc			
Lettuce, green leaf, raw, washed, dried, brown spots and core sections removed (Local Supermarket)/FL68	54.00	54.00	gram			
Tomatoes, red, ripe, raw, year round average (Local Supermarket)/FL68	55.35	55.35	gram			
Dressing, italian, fat-free (CA-Marzetti; CA-SUPC=5397252; AL-Marzetti; AL-SUPC=5397252)/FL68	42.50	1.00	pc			
Water, bottled (CA-Crystal Geyser; CA-SUPC=3199056; AL-AlpineSprings; AL-SUPC=5179569)/FL68	500.00	1.00	pc			
Water, bottled (CA-Crystal Geyser; CA-SUPC=3199056; AL-AlpineSprings; AL-SUPC=5179569)/FL68	500.00	1.00	pc			

DINNER

Food Item Description	Weight (g)	Amount	Measure	Prepared	Amt. Left	
Lean Cuisine, Sweet Sour Chicken (CA-SUPC=9148867; AL-Local Supermarket)/FL68	283.50	1.00	pc			
Spinach, frozen, chopped, raw (Sys Imp; CA-SUPC=1025857; AL-SUPC=1025857)/FL68	75.00	75.00	gram			
Mushrooms, canned, drained (Arrezio; CA-SUPC=6289130; AL-SUPC=6289130)/FL68	40.00	40.00	gram			
Margarine, (Smart Balance Omega; CA-SUPC=6631347; AL-SUPC=6631347)/FL68	5.00	1.00	pc			
Margarine, (Smart Balance Omega; CA-SUPC=6631347; AL-SUPC=6631347)/FL68	5.00	1.00	pc			

DINNER (continued)

Food Item Description	Weight (g)	Amount	Measure	Prepared	Amt. Left	
Juice, orange, frozen, 4-fl. oz (Ardmore; CA-SUPC=2200319; AL-SUPC=9548942)/FL68	124.50	1.00	pc			
Juice, orange, frozen, 4-fl. oz (Ardmore; CA-SUPC=2200319; AL-SUPC=9548942)/FL68	124.50	1.00	pc			
Water, bottled (CA-Crystal Geyser; CA-SUPC=3199056; AL-AlpineSprings; AL-SUPC=5179569)/FL68	500.00	1.00	pc			
Water, bottled (CA-Crystal Geyser; CA-SUPC=3199056; AL-AlpineSprings; AL-SUPC=5179569)/FL68	500.00	1.00	pc			

SNACK 1

Food Item Description	Weight (g)	Amount	Measure	Prepared	Amt. Left	
Peanut butter, smooth, with salt (House Recipe; CA-SUPC=6132377; AL-SUPC=6132377)/FL68	14.20	1.00	pc			
Carrots, baby, raw, washed, ends trimmed (Local Supermarket)/FL68	75.00	75.00	gram			
Water, bottled (CA-Crystal Geyser; CA-SUPC=3199056; AL-AlpineSprings; AL-SUPC=5179569)/FL68	500.00	1.00	pc			
Water, bottled (CA-Crystal Geyser; CA-SUPC=3199056; AL-AlpineSprings; AL-SUPC=5179569)/FL68	500.00	1.00	pc			

TRAIL MIX

Food Item Description	Weight (g)	Amount	Measure	Prepared	Amt. Left	
Jellybeans, Sunkist Tangerine (Jelly Belly; www.jellybelly.com)/FL68	27.50	27.50	gram			
Chocolate Chips, semisweet (CA-Hershey; CA-SUPC=4015137; AL-SUPC=4015137)/FL68	9.50	9.50	gram			
Soy Nuts, organic, dry roasted, unsalted, whole (www.nuts.com)	13.50	13.50	gram			
Nuts, macadamia, dry roasted, unalted (Sys Imp; CA-SUPC=5835376; AL-SUPC=5685904)/FL68	4.00	4.00	gram			
Kashi GOLEAN Crunch (Kellogg's; CA-SUPC=7677770; AL-Purchase locally)/FL68	3.00	3.00	gram			

TRAIL MIX ADD-ONS

Food Item Description	Weight (g)	Amount	Measure	Prepared	Amt. Left	
Coconut, dried, sweetened, shredded (Bakers Classic; CA-SUPC=4510871; AL-SUPC=4510467)/FL68	10.00	10.00	gram			
Almonds, blanched, slivered (CA-Sysco Classic; CA-SUPC=6814594; AL-Azar; AL-SUPC=5206051)/FL68	14.00	14.00	gram			

FL68: Participant Checklist / Menus 1G to 1L

For questions about Test Week Foods and/or Meal Schedule issues, please call the Metabolic Kitchen at 530-752-6109 (Mon-Fri)

Participant ID#: ________ - ________

Menu Date (MM/DD/YY): ____/____/____

Menu Day of Week (Circle One): Mo Tu We Th Fr Sa Su

Packed By	Food Items	Ate All	Notes	Prep Instructions
**Breakfast**	☐ In-House (Serve Time: ______ am pm)			☐ Pack-Out (Pick-Up Time: ______ am pm)
	Multivitamin (one)			**Step 1:** Combine oatmeal, wheat germ, milk, and raisins in a microwave-safe bowl. Use a microwave-safe plate as a lid.
	Oatmeal (2 packets)			**Step 2:** Microwave on high for 1 minute; stir. Repeat until oatmeal is hot. Be careful not to overheat as oatmeal may overflow. Use pot holder to remove bowl.
	Wheat Germ (19.7 g)			**Step 3:** Add margarine and honey.
	Milk (1 carton)			**Step 4:** Use bread to mop up any remaining cereal.
	Raisins (43 g)			
	Margarine (4 pats)			
	Honey (1 packet)			
	Bread (14.2 g)			
	☐ Water (1)* ☐ Water (2)*	←		
	☐ Water (3) * ☐ Water (4)*			
**Lunch**	☐ In-House (Serve Time: ______ am pm)			☐ Pack-Out (Pick-Up Time: ______ am pm)
	Chicken Soup (2 cans)			**Step 1:** Place soup in a microwave-safe bowl. Add salt and pepper. Use a microwave-safe plate as a lid.
	Salt (2 packets)			**Step 2:** Microwave on high for 1 minute; stir. Repeat until soup is hot. Be careful not to overheat as soup may overflow. Use pot holder to remove bowl.
	Pepper (2 packets)			**Step 3:** Wash lettuce and tomato under cold running water. Tear lettuce into bite-size pieces; and measure out 2 cups (not packed). Remove tomato core; cut in half. Use half with today's salad; save the other half for tomorrow's menu. Add dressing.
	Lettuce (2 cups, pieces)			**Step 4:** Spread margarine on bread, and use to mop up any remaining soup and/or salad dressing.
	Tomato (1/2 each)			
	Italian Dressing (1 packet)			
	Bread (42 g)			
	Margarine (1 pat)			
	☐ Water (5)* ☐ Water (6)*	←		
	☐ Water (7) * ☐ Water (8)*			
**Dinner**	☐ In-House (Serve Time: ______ am pm)			☐ Pack-Out (Pick-Up Time: ______ am pm)
	Sweet/Sour Chicken (1 box)			**Step 1:** Microwave Sweet/Sour Chicken following manufacturer's instructions.
	Spinach (75 g)			**Step 2:** Combine spinach, mushrooms, and margarine in a microwave-safe bowl. Use a microwave-safe plate as a lid.
	Mushrooms (40 g)			**Step 3:** Microwave spinach/mushrooms on high for 1 minute; stir. Repeat until hot. Use pot holder to remove bowl.
	Margarine (2 pats)			**Step 4:** Peel off Orange Juice lid, and drink.
	Orange Juice (2 containers)			
	☐ Water (9)* ☐ Water (10)*	←		
	☐ Water (11) * ☐ Water (12)*			
**Snacks**	☐ In-House (Serve Time: ______ am pm)			☐ Pack-Out (Pick-Up Time: ______ am pm)
	Peanut Butter (1 packet)			**Snack 1:** Dip carrots in peanut butter.
	Carrots (75 g)			**Snack 2:** Trail Mix: eat *ad lib* throughout the day.
	Trail Mix (individualized)			

* It is not essential for you to drink **all** the water we provide; but it is essential that you drink **only** the water that we provide to you during the test weeks. If you would like more water, please inform the Metabolic Kitchen staff.

Did you experience any adverse events (e.g., headache)? ☐ No ☐ Yes: ______________________________

Please record anything you ate and/or drank (including the amounts/servings of food and/or medication) that was not provided by the study team (if nothing, then write "X"): ______________________________

FL68 / Menu 2K (2100) Weigh Back

MENU 2 COMPONENTS, TEST WEEKS 1&2

Name: Composites - Set 1, FL68

Date: 01/24/13

SUPPLEMENTS

Food Item Description	Weight (g)	Amount	Measure	Prepared	Amt. Left	
Multivitamin (Bayer; One-a-Day for Women; Production Lot # = 238977-01; Expiration Date = 09/11)/FL68	5.00	1.00	each			

BREAKFAST

Food Item Description	Weight (g)	Amount	Measure	Prepared	Amt. Left	
Cheerios, 0.688 oz bowl (General Mills; CA-SUPC=4212221; AL-SUPC=4212221)/FL68	19.50	1.00	pc			
Cheerios, 0.688 oz bowl (General Mills; CA-SUPC=4212221; AL-SUPC=4212221)/FL68	19.50	1.00	pc			
Wheat Germ, toasted (Quaker/Mother's; www.quakeroats.elsstore.com; UPC=03000021638)/FL68	19.00	19.00	gram			
Milk, nonfat, lactose-free (CA-Dairy Ease; CA-SUPC=9016759; AL-Lactaid; AL-SUPC=9582875)/FL68	246.00	1.00	pc			
Applesauce, canned (Sahara Burst; CA-SUPC=6818579; AL-SUPC=6818579)/FL68	113.40	1.00	pc			
Applesauce, canned (Sahara Burst; CA-SUPC=6818579; AL-SUPC=6818579)/FL68	113.40	1.00	pc			
Juice, cranberry, 5.5 fl. oz. can (Oceanspray; CA-SUPC=4036315; AL-SUPC=4036315)/FL68	170.50	1.00	pc			
Water, bottled (CA-Crystal Geyser; CA-SUPC=3199056; AL-AlpineSprings; AL-SUPC=5179569)/FL68	500.00	1.00	pc			
Water, bottled (CA-Crystal Geyser; CA-SUPC=3199056; AL-AlpineSprings; AL-SUPC=5179569)/FL68	500.00	1.00	pc			

LUNCH

Food Item Description	Weight (g)	Amount	Measure	Prepared	Amt. Left	
Bread, white, (Sysco Classic; CA-SUPC=9843830; AL-SUPC=8386765)/FL68	28.35	28.35	gram			
Mayonnaise, Light (CA-Best Foods/Hellman's; CA-SUPC=6260509; AL-Best Foods/Hellman's; AL-Woodfruitter UPC=508713)/FL68	12.48	1.00	pc			
Mayonnaise, Light (CA-Best Foods/Hellman's; CA-SUPC=6260509; AL-Best Foods/Hellman's; AL-Woodfruitter UPC=508713)/FL68	12.48	1.00	pc			
Mustard, yellow (CA-Sysco Classic; CA-SUPC=5106059; AL-French's; AL-SYSCO=6391124)/FL68	4.20	1.00	pc			
Turkey, breast, slices (Hormel; CA-SUPC=2477081; AL-SUPC=2477081) / FL68	25.50	25.50	gram			
Cheese, cheddar, reduced-fat, sliced (CA-Safeway; Lucerne; AL-Local Supermarket; Brand Name??????)/FL68	60.00	60.00	gram			
Lettuce, green leaf, raw, washed, dried, brown spots and core sections removed (Local Supermarket)/FL68	54.00	54.00	gram			
Tomatoes, red, ripe, raw, year round average (Local Supermarket)/FL68	55.35	55.35	gram			
Carrots, baby, raw, washed, ends trimmed (Local Supermarket)/FL68	90.00	90.00	gram			
Broccoli, raw, washed, florets, yellow trimmed off (Local Supermarket)/FL68	75.00	75.00	gram			
Dressing, Ranch, regular (Marzetti; CA-SUPC=5274899; AL-SUPC=5274899)/FL68	42.53	1.00	pc			
Water, bottled (CA-Crystal Geyser; CA-SUPC=3199056; AL-AlpineSprings; AL-SUPC=5179569)/FL68	500.00	1.00	pc			
Water, bottled (CA-Crystal Geyser; CA-SUPC=3199056; AL-AlpineSprings; AL-SUPC=5179569)/FL68	500.00	1.00	pc			

DINNER

Food Item Description	Weight (g)	Amount	Measure	Prepared	Amt. Left	
Lean Cuisine, Chicken with Basil Cream Sauce (Stouffers; CA-SUPC=1426345; AL-Local Supermarket)/FL68	240.97	1.00	pc			
Peas, green, frozen, unprepared (CA-Sysco Imperial; CA-SUPC=3959210; AL-Sysco Classic; AL-SUPC=3608353)/FL68	65.00	65.00	gram			
Margarine, (Smart Balance Omega; CA-SUPC=6631347; AL-SUPC=6631347)/FL68	5.00	1.00	pc			
Margarine, (Smart Balance Omega; CA-SUPC=6631347; AL-SUPC=6631347)/FL68	5.00	1.00	pc			
Peaches, canned, light syrup pack, solids and liquids (CA-Dole; CA-SUPC=4714671; AL-Sahara Burst; AL-SUPC=9704917)/FL68	113.40	1.00	pc			
Juice, V8, low-sodium (Campbell's; CA-SUPC=5142187; AL-SUPC=5142187)/FL68	167.10	1.00	pc			
Juice, V8, low-sodium (Campbell's; CA-SUPC=5142187; AL-SUPC=5142187)/FL68	167.10	1.00	pc			
Water, bottled (CA-Crystal Geyser; CA-SUPC=3199056; AL-AlpineSprings; AL-SUPC=5179569)/FL68	500.00	1.00	pc			
Water, bottled (CA-Crystal Geyser; CA-SUPC=3199056; AL-AlpineSprings; AL-SUPC=5179569)/FL68	500.00	1.00	pc			

SNACK 1

Food Item Description	Weight (g)	Amount	Measure	Prepared	Amt. Left	
Cranberries, dried, craisins (Sysco Imperial; CA-SUPC=7102403; AL-Oceanspray; AL-SUPC=7415460)/FL68	61.50	61.50	gram			
Water, bottled (CA-Crystal Geyser; CA-SUPC=3199056; AL-AlpineSprings; AL-SUPC=5179569)/FL68	500.00	1.00	pc			
Water, bottled (CA-Crystal Geyser; CA-SUPC=3199056; AL-AlpineSprings; AL-SUPC=5179569)/FL68	500.00	1.00	pc			

TRAIL MIX

Food Item Description	Weight (g)	Amount	Measure	Prepared	Amt. Left	
Jellybeans, Very Cherry (Jelly Belly; www.jellybelly.com)/FL68	27.50	27.50	gram			
Chocolate Chips, semisweet (CA-Hershey; CA-SUPC=4015137; AL-SUPC=4015137)/FL68	9.50	9.50	gram			
Soy Nuts, organic, dry roasted, unsalted, whole (www.nuts.com)	13.50	13.50	gram			
Nuts, macadamia, dry roasted, unalted (Sys Imp; CA-SUPC=5835376; AL-SUPC=5685904)/FL68	4.00	4.00	gram			
Kashi GOLEAN Crunch (Kellogg's; CA-SUPC=7677770; AL-Purchase locally)/FL68	3.00	3.00	gram			

TRAIL MIX ADD-ONS

Food Item Description	Weight (g)	Amount	Measure	Prepared	Amt. Left	
Coconut, dried, sweetened, shredded (Bakers Classic; CA-SUPC=4510871; AL-SUPC=4510467)/FL68	11.00	11.00	gram			
Almonds, blanched, slivered (CA-Sysco Classic; CA-SUPC=6814594; AL-Azar; AL-SUPC=5206051)/FL68	10.00	10.00	gram			

For questions about Test Week Foods and/or Meal Schedule issues, please call the Metabolic Kitchen at 530-752-6109 (Mon-Fri)

FL68: Participant Checklist / Menus 2G to 2L

Participant ID#: ________ - ________

Menu Date (MM/DD/YY): ____/____/____ Menu Day of Week (Circle One): Mo Tu We Th Fr Sa Su

Packed By	Food Items	Ate All	Notes	Prep Instructions
Breakfast	*☐ In-House (Serve Time: ______ am pm)*			*☐ Pack-Out (Pick-Up Time: ______ am pm)*
	Multivitamin (one)			***Step 1:*** Combine Cheerios, wheat germ, and milk. (Note: if you wish, Trail Mix may be added to cereal). Be sure to drink any remaining milk.
	Cheerios (2 bowls)			
	Wheat Germ (19 g)			
	Milk (1 carton)			
	Applesauce (2 containers)			
	Cranberry Juice (1 can)			
	☐ Water (1)* ☐ Water (2)*	←		
	☐ Water (3) * ☐ Water (4)*			
Lunch	*☐ In-House (Serve Time: ______ am pm)*			*☐ Pack-Out (Pick-Up Time: ______ am pm)*
	Bread (28.4 g)			***Step 1:*** For sandwich, spread mayonnaise and mustard onto bread. Add turkey slices and half of the cheese. Set aside the remaining half of cheese.
	Mayonnaise (2 packets)			
	Mustard (1 packet)			
	Turkey (25.5 g)			
	Cheese (60 g)			***Step 2:*** Wash lettuce and tomato under cold running water. Tear lettuce into bite-size pieces; and measure out 2 cups (not packed). Remove tomato core; cut in half. Use half with today's salad; save the other half for tomorrow's menu. Add carrots and dressing. (Note: some of the lettuce for the salad may be added to the sandwich). Use sandwich to mop up any remaining salad dressing.
	Lettuce (2 cups, pieces)			
	Tomato (1/2 each)			
	Carrots (90 g)			
	Broccoli (75 g)			
	Ranch Dressing (1 packet)			
	☐ Water (5)* ☐ Water (6)*	←		
	☐ Water (7) * ☐ Water (8)*			
				Step 3: Place broccoli in a microwave-safe bowl. Microwave on high for 1 minute. Repeat until hot. Add remaining half of cheese to the top. Microwave on high for 15 seconds or until cheese melts. Use pot holder to remove bowl.
Dinner	*☐ In-House (Serve Time: ______ am pm)*			*☐ Pack-Out (Pick-Up Time: ______ am pm)*
	Chicken with Basil (1 box)			***Step 1:*** Microwave Chicken with Basil following manufacturer's instructions.
	Peas (65 g)			***Step 2:*** Combine peas and margarine in a microwave-safe bowl. Use a microwave-safe plate as a lid.
	Margarine (2 pats)			
	Peaches (1 container)			
	V8 (2 cans)			***Step 3:*** Microwave peas and margarine on high for 1 minute; stir. Repeat until hot. Use pot holder to remove bowl. Use chicken to mop up remaining margarine from peas.
	☐ Water (9)* ☐ Water (10)*			
	☐ Water (11) * ☐ Water (12)*	←		
Snacks	*☐ In-House (Serve Time: ______ am pm)*			*☐ Pack-Out (Pick-Up Time: ______ am pm)*
	Cranberries (61.5 g)			***Snack 1:*** Cranberries.
	Trail Mix (individualized)			***Snack 2:*** Trail Mix: eat *ad lib* throughout the day.

* It is not essential for you to drink **all** the water we provide; but it is essential that you drink **only** the water that we provide to you during the test weeks. If you would like more water, please inform the Metabolic Kitchen staff.

Did you experience any adverse events (e.g., headache)? ☐ No ☐ Yes: ______________________________

Please record anything you ate and/or drank (including the amounts/servings of food and/or medication) that was not provided by the study team (if nothing, then write "X"): ____

SUBJECT FOOD DIARY, TEST WEEKS & INTERVENTION PERIOD

Beta-Oxidation Study

Participant Food Tracking Booklet

Participant ID: ____________________

Week of __/__/__ - __/__/__

USDA ARS Western Human Nutrition Research Center

Tips for Keeping a Successful Food Record

- Remember, recording is a **motivational tool** that will help you be successful at identifying problematic foods. It will also help you develop better self-discipline, and will force you to think twice about what you put into your mouth.
- **Be honest!** Record what you actually eat so the dietitian can better help you reach both your goals and the study goals.
- Keep this booklet with you **AT ALL TIMES** and write down **EVERYTHING** you eat and/or drink. A piece of gum, a small handful of chips, a can of regular soda or a small cookie may not seem like much, but all these calories count during the study!
- **Write it when you eat and/or drink it.** Don't wait until the end of the day, as our memories are not good at remembering what we ate and/or drank earlier in the day.
- **Be specific.** Include "extras" (e.g., gravy on your chicken, light mayonnaise or tomato on your sandwich, butter on your vegetables or bread, and low fat salad dressings).
- **Estimate amounts.** If you had a cup of soup, measure or estimate (see How to Estimate Portion Sizes below) the actual amount (e.g., 12 oz. Campbell's chicken noodle soup) instead of writing "cup" of soup. If you ate at a cafeteria or dining hall, estimate as closely as you can (or write down how many servings you ate).
- **Write legibly.** Illegible records will take longer to review when you meet with the dietitian.

Adapted from http://www.ksu.ksu.edu/lafene/foodrecords.htm

(Inches)

How Do I Estimate Portion Sizes?

Portion Size	Everyday Object		Portion Size	Everyday Object
**1 cup =** 1 baseball (hard)	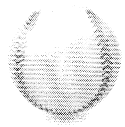		**1 medium fruit =** 1 baseball (hard)	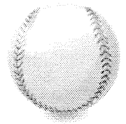
**1/2 cup =** 1/2 baseball (hard)	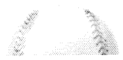		**1 1/2 oz cheese =** 4 stacked dice	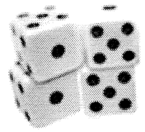
**1 teaspoon =** 1 thumb tip	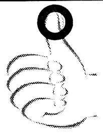		**3 oz. meat =** 1 deck of cards	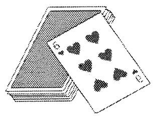
**1/4 cup =** 2 ping-pong balls	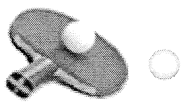		**2 Tablespoons =** 1 ping-pong ball	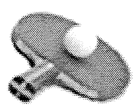

References and for more information: www.win.niddk.nih.gov/publications/just_enough.htm#howdoi; www.k-state.edu/lafene/foodrecords.htm

Helpful Recording Tips

Household Measurements
3 tsp = 1 T
1 T = 1/2 fl. oz.
1/4 c = 2 fl. oz. or 4 T
1/2 c = 4 fl. oz or 8 T
1 c = 8 fl. oz. or 16 T
2 c = 1 pint
2 pints = 1 quart

Common Abbreviations	
Cup(s) = c	Packet(s) = pkt
Diameter = dia	Slice(s) = sl
Fluid Ounce(s) = fl. oz.	Square(s) = sq
Gram(s) = g	Tablespoon(s) = T or Tbsp
Large = lg	Teaspoon(s) = t or tsp
Medium = med	Whole = whl
Milligram(s) = mg	With = w/
Ounce(s) = oz	Without = w/o
Piece(s) = pc	

How Do I Estimate 1-Ounce Equivalents (oz. eq.)?

Grains	
Bagels	One 2 1/2”-dia “mini” bagel (4 1/2”-dia bagel = 4-oz. eq.)
Breads	One “regular” slice
Cornbread	One small piece (2 1/2” x 1 1/4” x 1 1/4”)
Crackers	5 whole wheat; 7 square/round
English Muffins	1/2 regular-sized muffin
Muffins	1 small (2 1/2”-dia)
Cooked Cereal	1/2 c cooked (1 oz. dry)
Cold Cereal	1 c unsweetened flakes/ rounds (3/4 c. sweetened); 1 1/4 c puffs
Rice and Pasta	1/2 cup cooked (1 oz dry)
Tortillas	One 6”-dia flour or corn

Meat & Beans	
Beef	1 oz. cooked lean
Pork	1 oz. cooked lean (incl. ham)
Chicken or Turkey	1 oz. cooked (no skin)
Fish and Shellfish	1 oz. cooked
Eggs	1 egg
Nuts and Seeds	1/2 oz. nuts, 1/2 oz. of seeds; 1 Tablsepoon of pea-nut butter or almond butter
Dry Beans & Peas	1/4 cup cooked dry beans; 1/4 cup cooked dry peas; 1.4 cup baked or refried beans

References and for more information: http://www.mypyramid.gov/pyramid/grains_counts.html and http://www.mypyramid.gov/pyramid/meat_counts.html

Today's Date : *09 / 09 / 2009* **Day of Week**: Mon Tue (Wed) Thu Fri Sat Sun

Time	Food/Beverage	Brand/Restaurant	Portion
[X]	**One Multivitamin (One-a-Day for Women)**		
8 am	*Oatmeal, instant, plain*	*Quaker*	*1 pkt*
	Banana	——	*1/2 med*
	Almonds, raw, sliced	*Diamond*	*2 T*
	Blueberries, unsweetened, fresh	——	*1/2 c*
	Milk, skim	*Berkeley Farms*	*1 c*
12 pm	*Tuna, unsalted, packed in water*	*Bumble Bee*	*2 oz*
	Bread, whole wheat	*Orowheat*	*2 sl*
	Mayonnaise, light	*Best Foods*	*1 T*
	Tomato	——	*1 med sl*
	Lettuce, green leaf	——	*1 med leaf*
	Water, bottled	*Crystal Geyser*	*16.9 fl. oz.*
3 pm	*Broccoli, fresh*	——	*1/2 c*
	Hummus	*Trader Joe's*	*2 T*
7pm	*Mexican "Stir Fry"*		
	Mixed bell peppers, sautéed	——	*1 c*
	Red onion, sautéed	——	*1/2 c*
	Olive oil (for sautéeing)	*Pompei*	*1 t*
	Black Beans, canned, drained	*Progresso*	*3/4 c*
	Rice, white, cooked	*Uncle Ben's*	*1 c*
	Salsa, medium	*Pace*	*2 T*
	Sour cream, low fat	*Berkeley Farms*	*2 T*
9 pm	*Yogurt, low fat vanilla*	*Yoplait*	*6 oz*

Food Group	My Goals
Grain (1-oz. eq) [Whole Grains]	**5** [2.5]
Vegetable (cups)	**2**
Fruit (cups)	**1.5**
Dairy (cups)	**2.5**
Meat & Beans (1-oz eq)	**4.5**
Oils (tsp)	**4**
Extras (calories)	**152**
Fluids (8-fl. oz.)	**≥6**

Adverse Events: ☐ No ☒ **Yes**: *Headache*
Medications: ☐ No ☒ **Yes**: *Aspirin—325 mg x2*
Thoughts:

My Mood/Feelings Today:

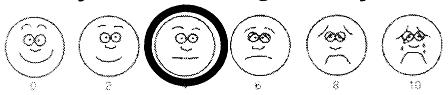

EXAMPLE

Today's Date : _____/_____/_____ **Day of Week**: Mon Tue Wed Thu Fri Sat Sun

Time	Food/Beverage	Brand/Restaurant	Portion
[ ]	One Multivitamin (One-a-Day for Women)		

Food Group	My Goals
Grain (1-oz. eq) [Whole Grains]	5 [2.5]
Vegetable (cups)	2
Fruit (cups)	1.5
Dairy (cups)	2.5
Meat & Beans (1-oz eq)	4.5
Oils (tsp)	4
Extras (calories)	152
Water (8-fl. oz.)	≥6

Adverse Events: ☐ No ☐ Yes: ____________

Medications: ☐ No ☐ Yes: ____________

Thoughts:

My Mood/Feelings Today:

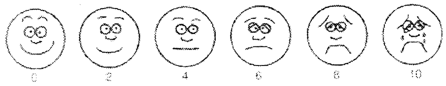

Use this space for writing down additional notes and/or recipes; collecting food labels; or noting any other information you believe may be helpful for communicating with the dietitian.

If found, please return to the

USDA ARS Western Human Nutrition Research Center

430 West Health Sciences Drive
Davis, CA 95616

Contact Information:

530-754-6854
(Research Dietitian)

This booklet was assembled by Dustin J. Burnett, MS, RD and Elaine Souza, MPH, RD; and revised by the BOx Study Team as an informational and tracking tool used by the study participants of the Box Study.
