# Supplementary material for: Improved Metabolic Health Alters Host Metabolism in Parallel with Changes in Systemic Xeno-Metabolites of Gut Origin
Source: PLoS One. 2014 Jan 8;9(1):e84260. doi: 10.1371/journal.pone.0084260 (PMC3885560; doi:10.1371/journal.pone.0084260)
Supplement: File S2 — Plasma concentration excursions for plasma analytes derived from metabolomics studies. Concentrations were tracked following a standard 75 g oral glucose tolerance test prior to (Pre-) and after (Post-) weight loss and fitness intervention in sedentary obese insulin-resistant womens. Mean±SEM are shown for n = 15 (Pre) and n = 12 (Post). (PDF) [file pone.0084260.s007.pdf]

SUPPLEMENTAL MATERIALS 2 (Campbell et al.). **Table:** Clinical parameters from the subset of subject that completed both Test Week 1 (Pre-weight loss and fitness intervention) and Test Week 2 (Post-weight loss and fitness intervention) regimens. See manuscript Table 1 for complete cohort results. **Figures:** Plasma concentration excursions for plasma analytes derived from metabolomics studies. Concentrations were tracked following a standard 75 g oral glucose tolerance test prior to (Pre-) and after (Post-) a weight loss and fitness intervention in sedentary obese hyperinsulinemic women. Mean ± SEM are shown for n=15 (Pre) and n=12 (Post).

**Supplemental Table.** Body mass, fitness, and glucose homeostasis indices in previously sedentary obese women following a weight loss and fitness intervention (subset completing both Test Week 1 and Test Week 2).			
	Pre-intervention[a]	Post-intervention[b]	p-value Mixed Model[c]
Body Mass (kg)	89.2 ± 2.6	83.0 ± 3.1	<0.0001
BMI, kg/m^2	33 ± 0.8	31.0 ± 0.9	<0.0001
Body Fat%	47.4 ± 1.1	43.4 ± 1.5	<0.0001
Fat Mass (kg)	42.07 ± 2.8	36.2 ± 2.4	0.0009
Fat Free Mass (FFM, kg)	43.4 ± 0.8	43.3 ± 0.9	NS
VO_2peak (mL/kg/min)	21.3 ± 1.1	25.6 ± 1.1	<0.0001
VO_2peak (mL/kg FFM/min)	43.5 ± 1.8	49.4 ± 1.4	<0.0001
Maximal Power (Watts)	141.7 ± 4.6	166.7 ± 6.4	<0.0001
Fasting Glucose (mg/dL)	87.6 ± 1.4	84.6 ± 1.5	0.038
Fasting Insulin (µU/mL)	18.7 ± 2.6	13.7 ± 1.7	<0.0001
Matsuda Index	1.97 ± 0.20	2.91 ± 0.36	0.0083
QUICKI	0.315 ± 0.005	0.332 ± 0.006	0.0048

[a]pre-diet and exercise intervention, n=12

[b]post-diet and exercise intervention, n=12

[c]comparisons using paired t-tests from subjects completing both Test Week 1 (pre-intervention) and Test Week 2 (post-intervention) yielded identical statistical patterns

Values are means ± SEM; NS = not statistically significant

* mean ± SEM subject age was 41 ± 1.6 yrs and the median age was 40.5 yrs

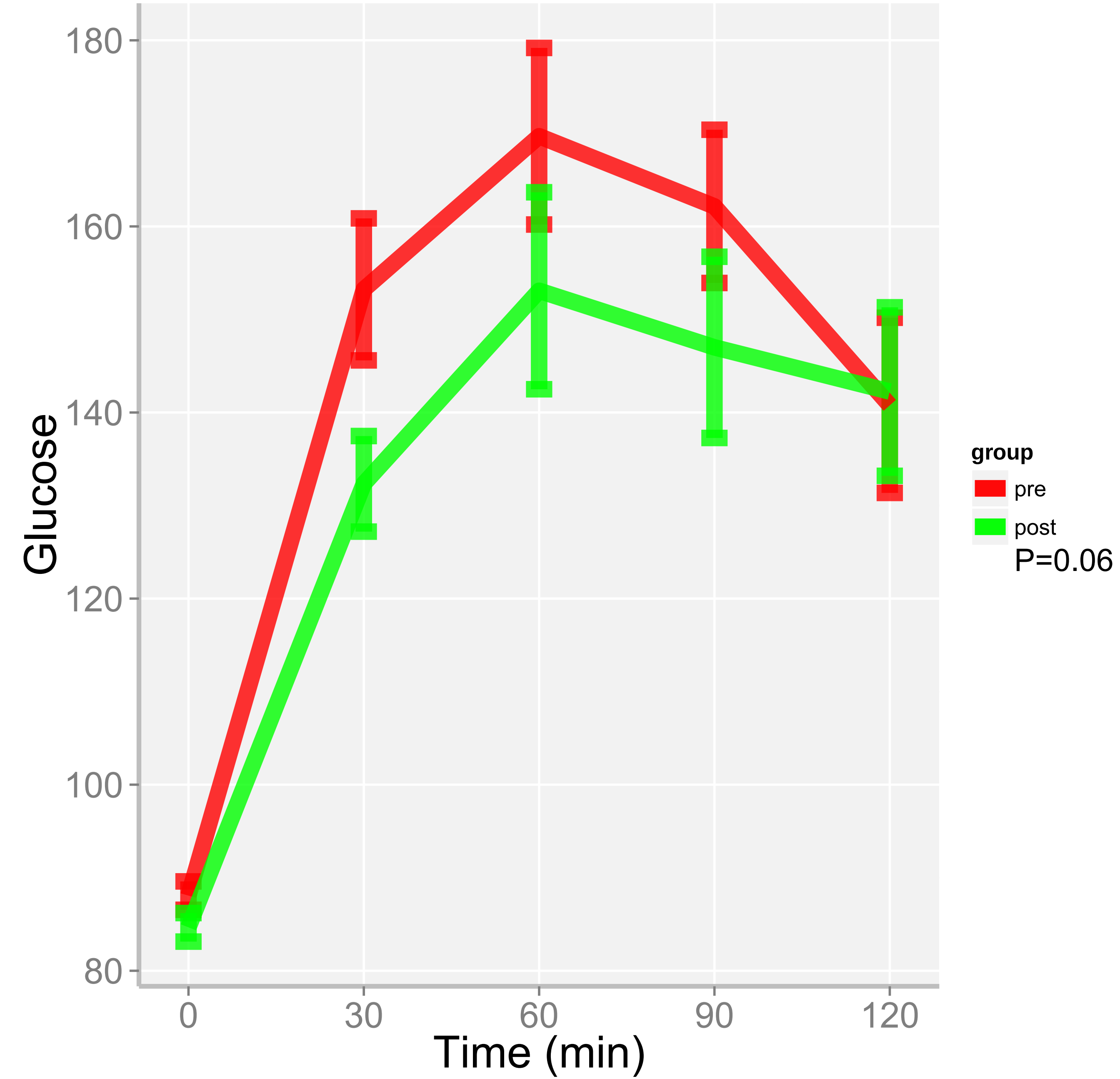

Glucose
180
160
140
120
100
80
0
30
60
90
120
Time (min)
group
pre
post
P=0.06

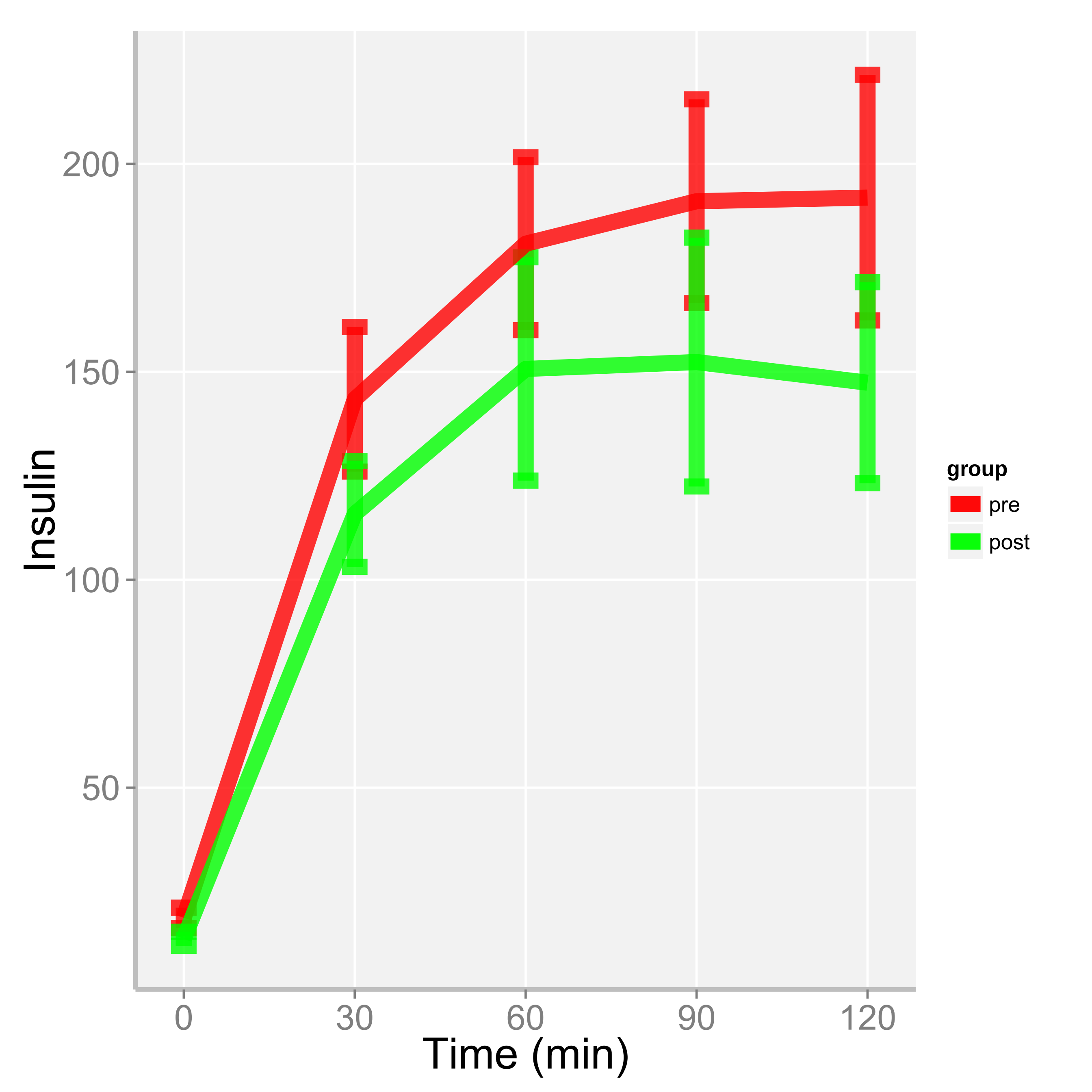

Insulin
200
150
100
50
0
30
60
90
120
Time (min)
group
pre
post

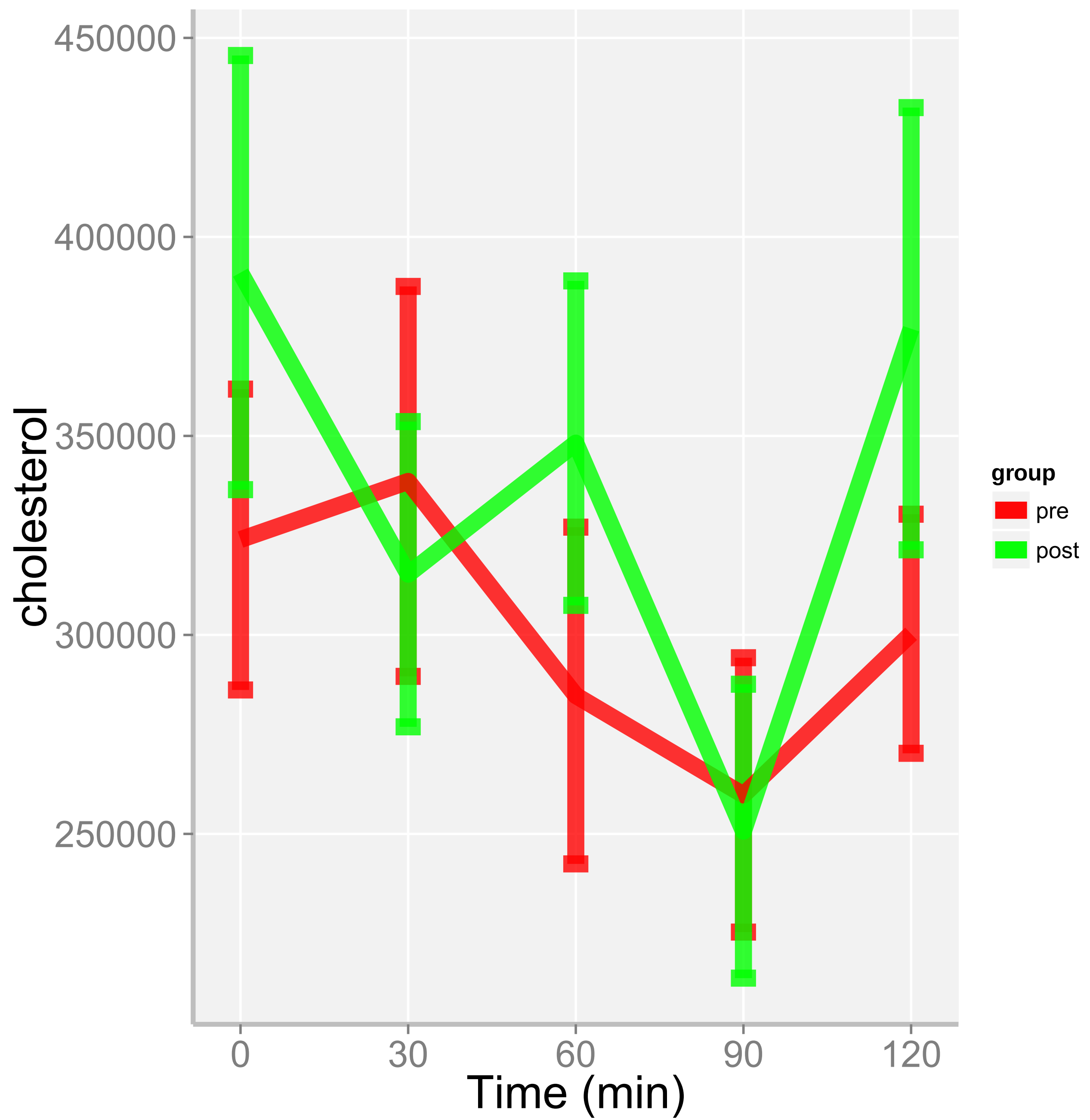

450000
400000
350000
300000
250000
cholesterol
0
30
60
90
120
Time (min)
group
pre
post

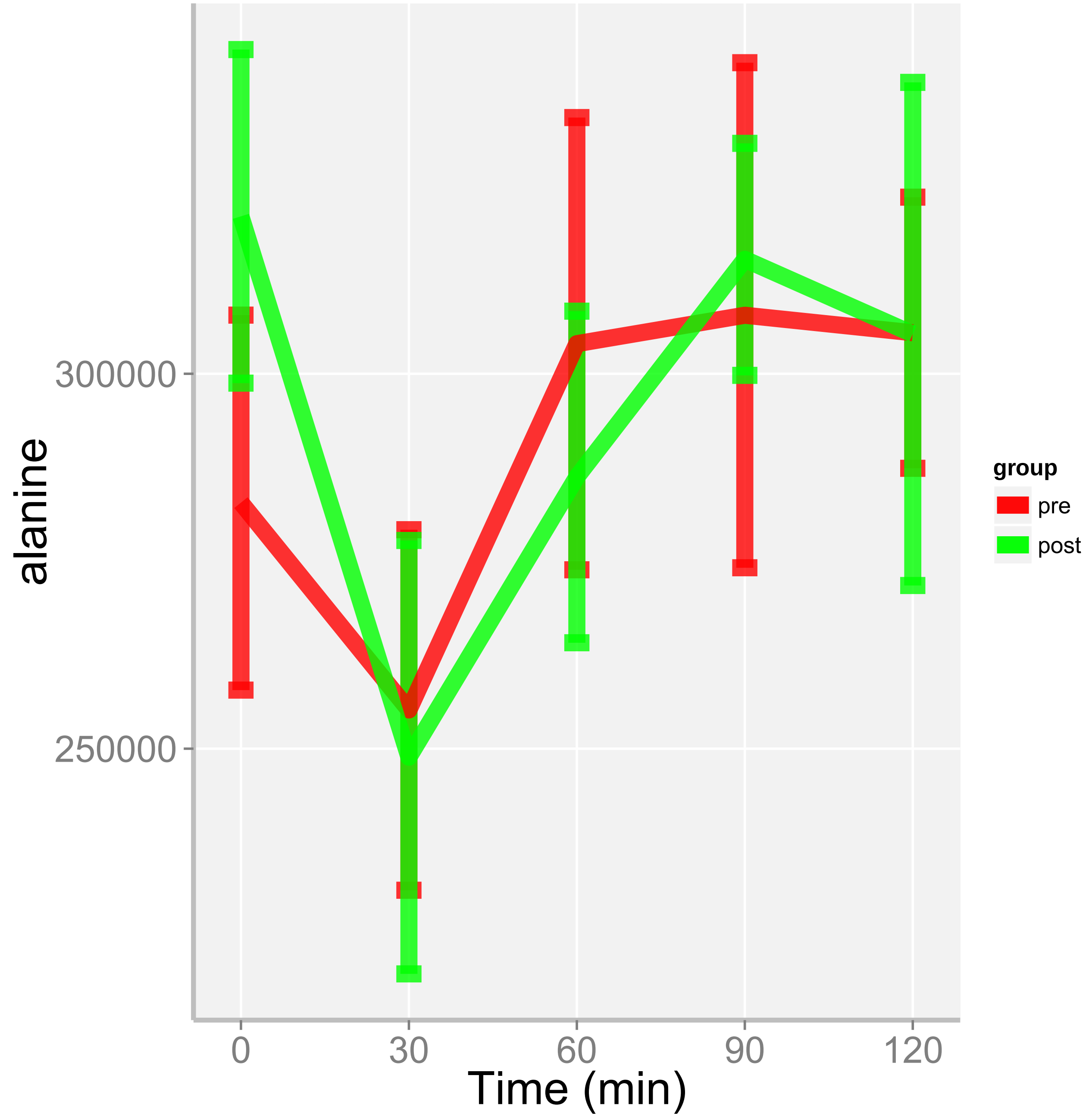

alanine
300000
250000
0
30
60
90
120
Time (min)
group
pre
post

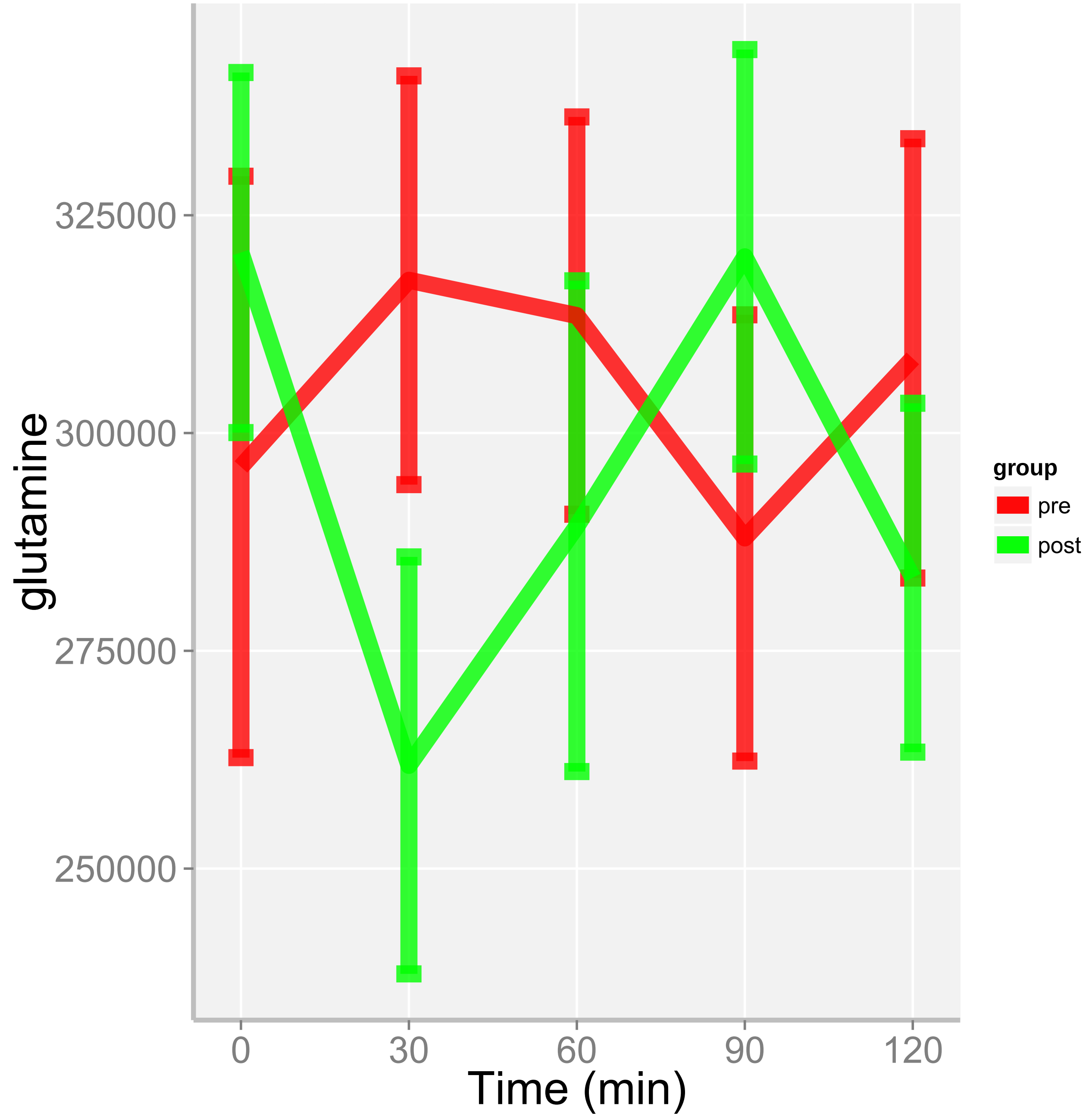

glutamine
325000
300000
275000
250000
0
30
60
90
120
Time (min)
group
pre
post

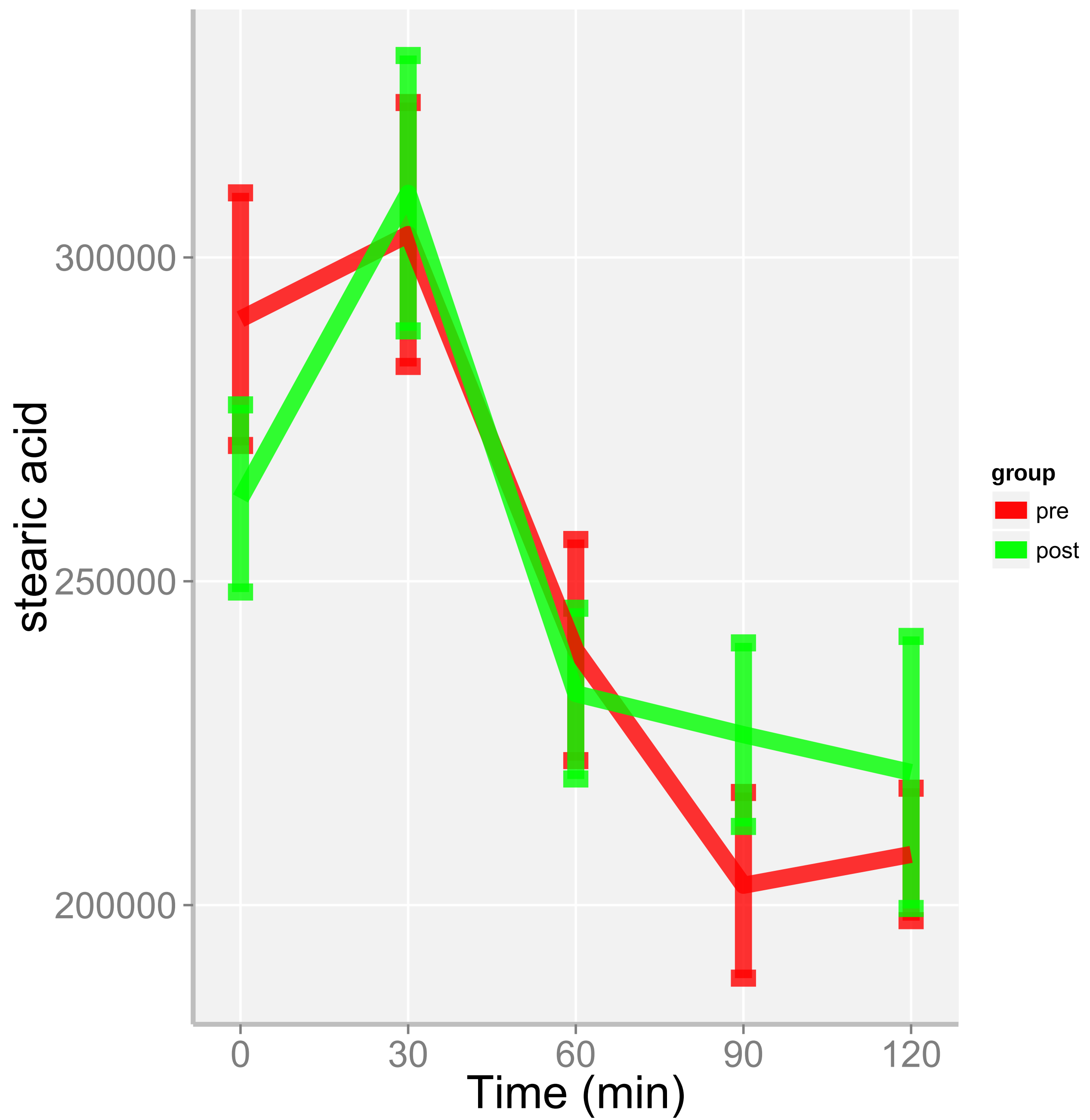

stearic acid
300000
250000
200000
0
30
60
90
120
Time (min)
group
pre
post

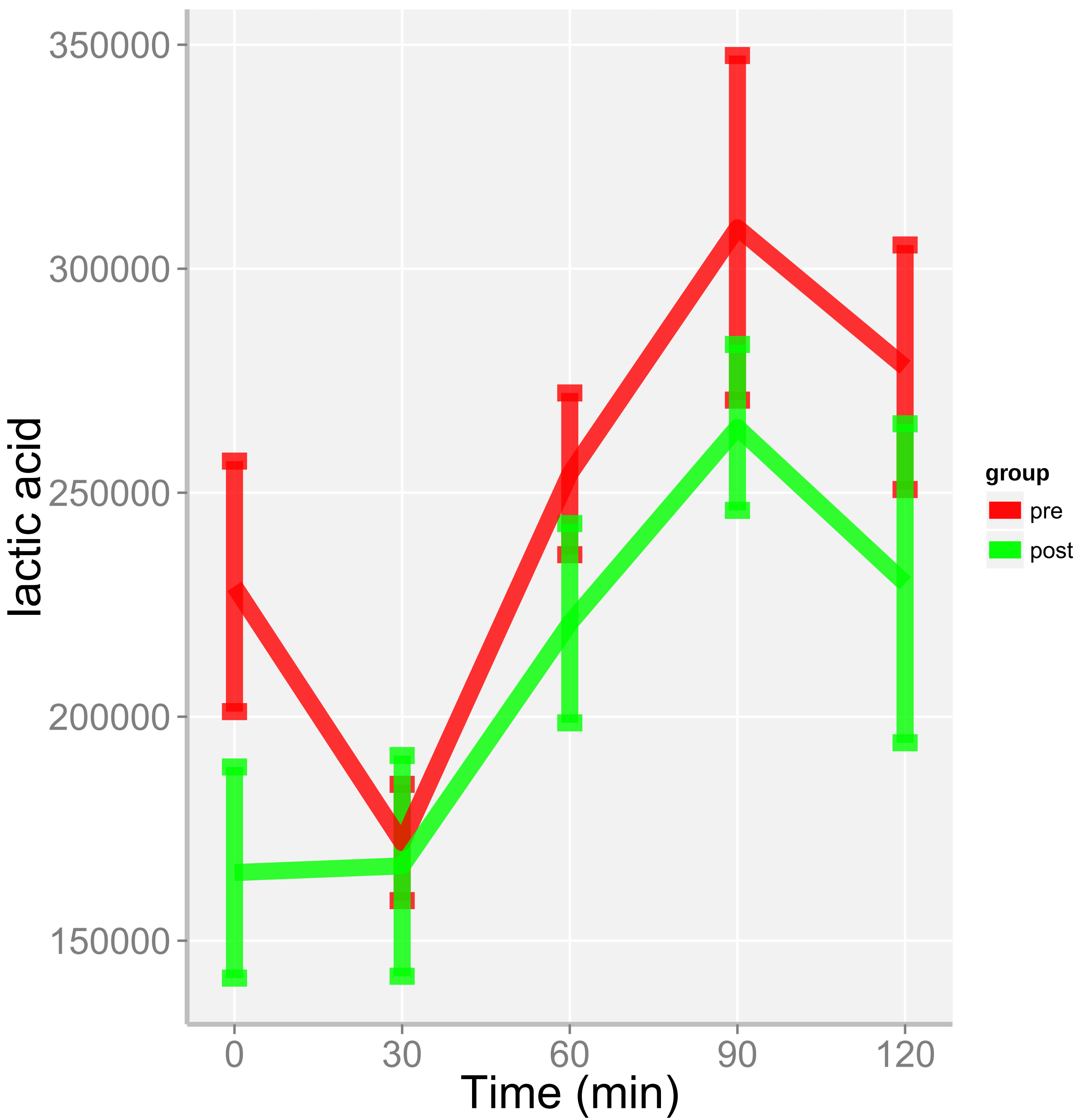
lactic acid
350000
300000
250000
200000
150000
0
30
60
90
120
Time (min)
group
pre
post

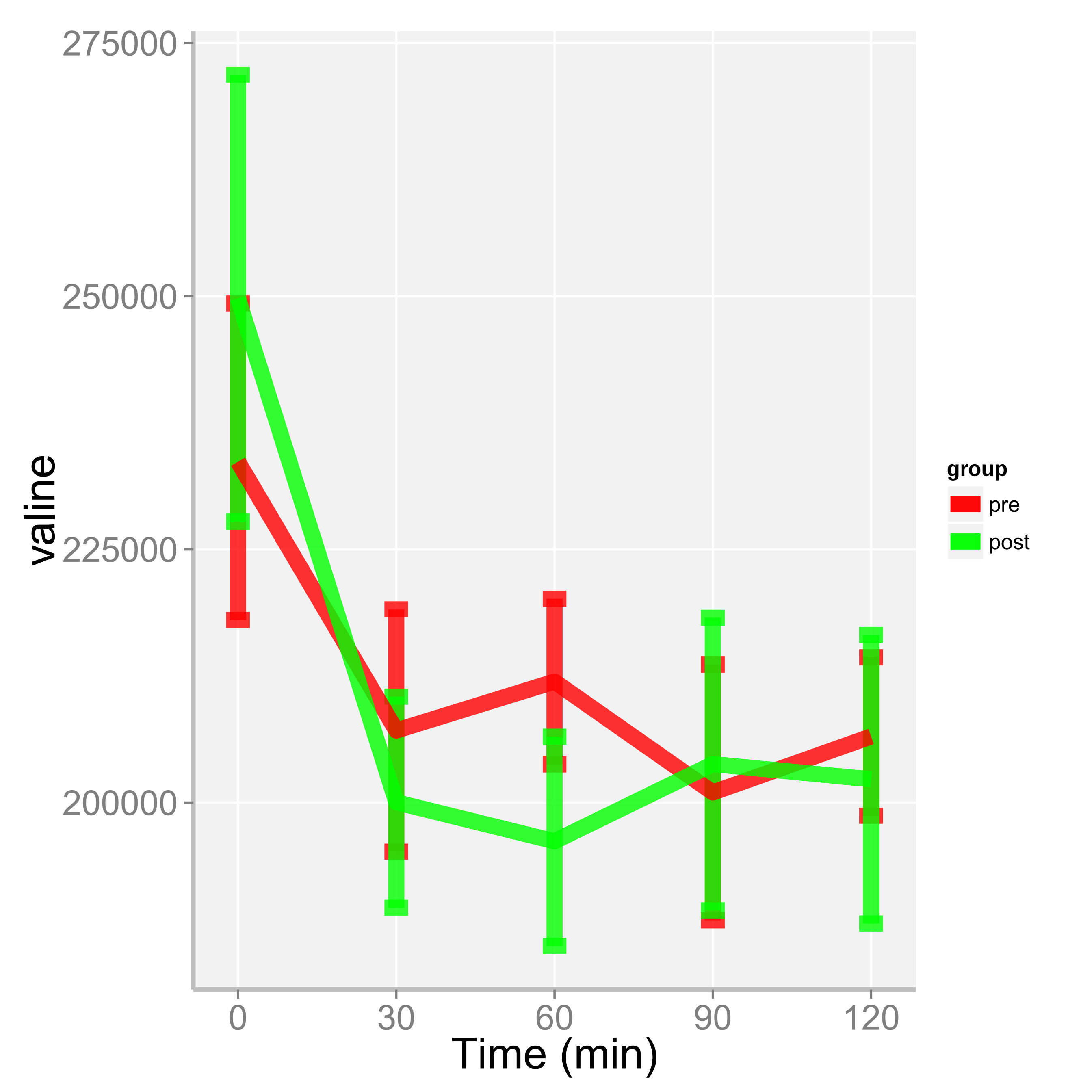

275000
250000
225000
200000
valine
0
30
60
90
120
Time (min)
group
pre
post

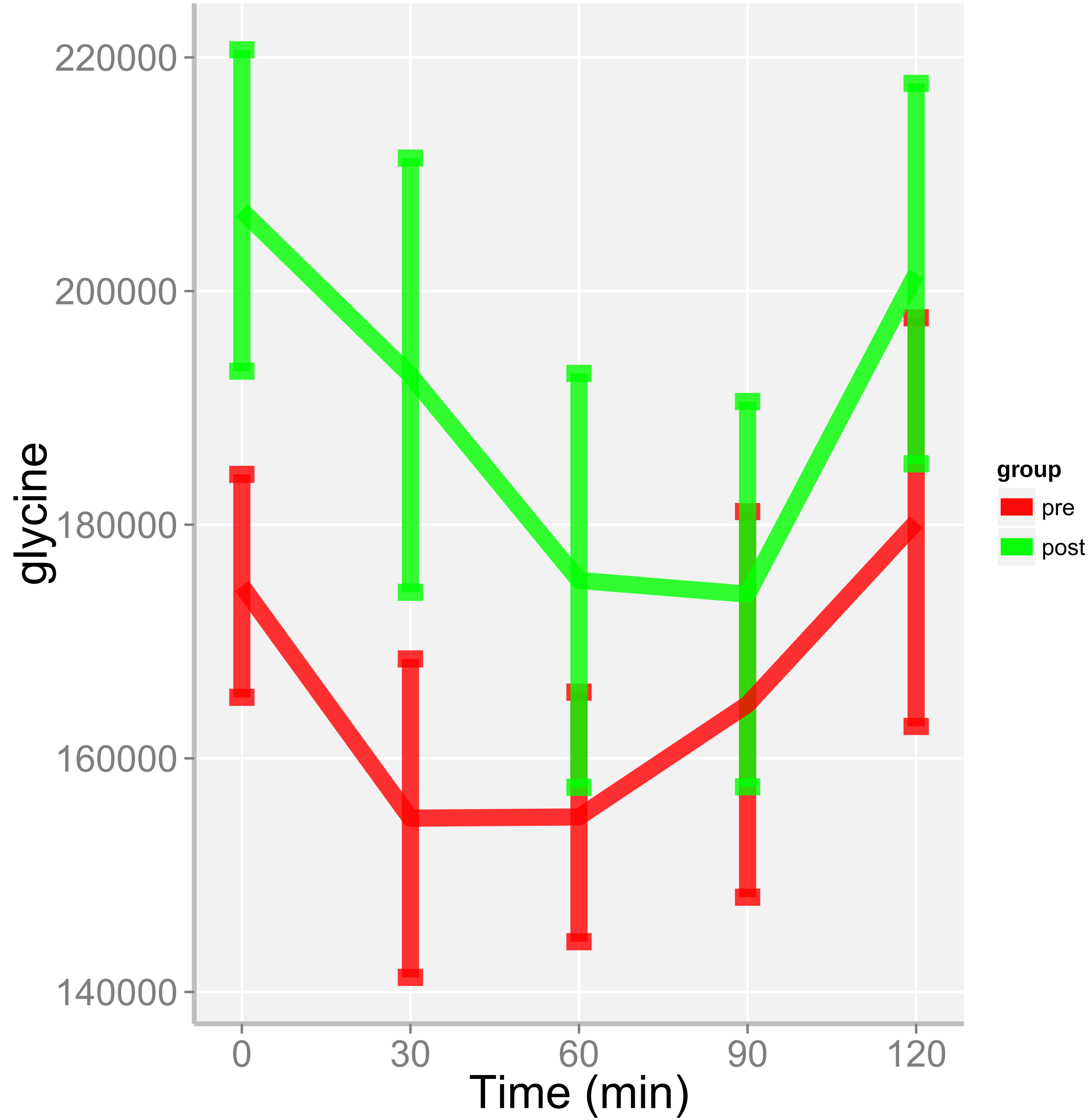

glycine
220000
200000
180000
160000
140000
0
30
60
90
120
Time (min)
group
pre
post

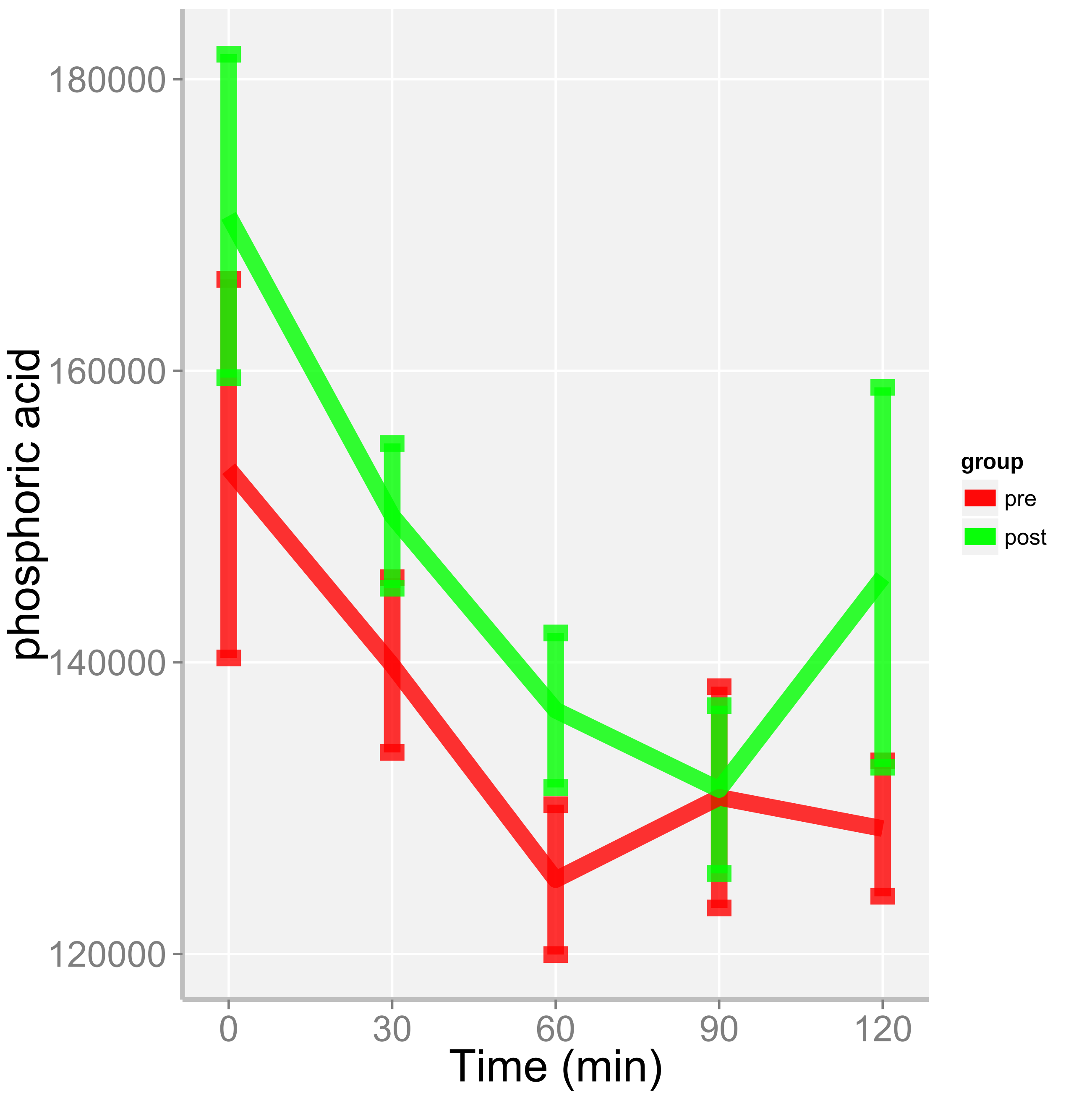

phosphoric acid
180000
160000
140000
120000
0
30
60
90
120
Time (min)
group
pre
post

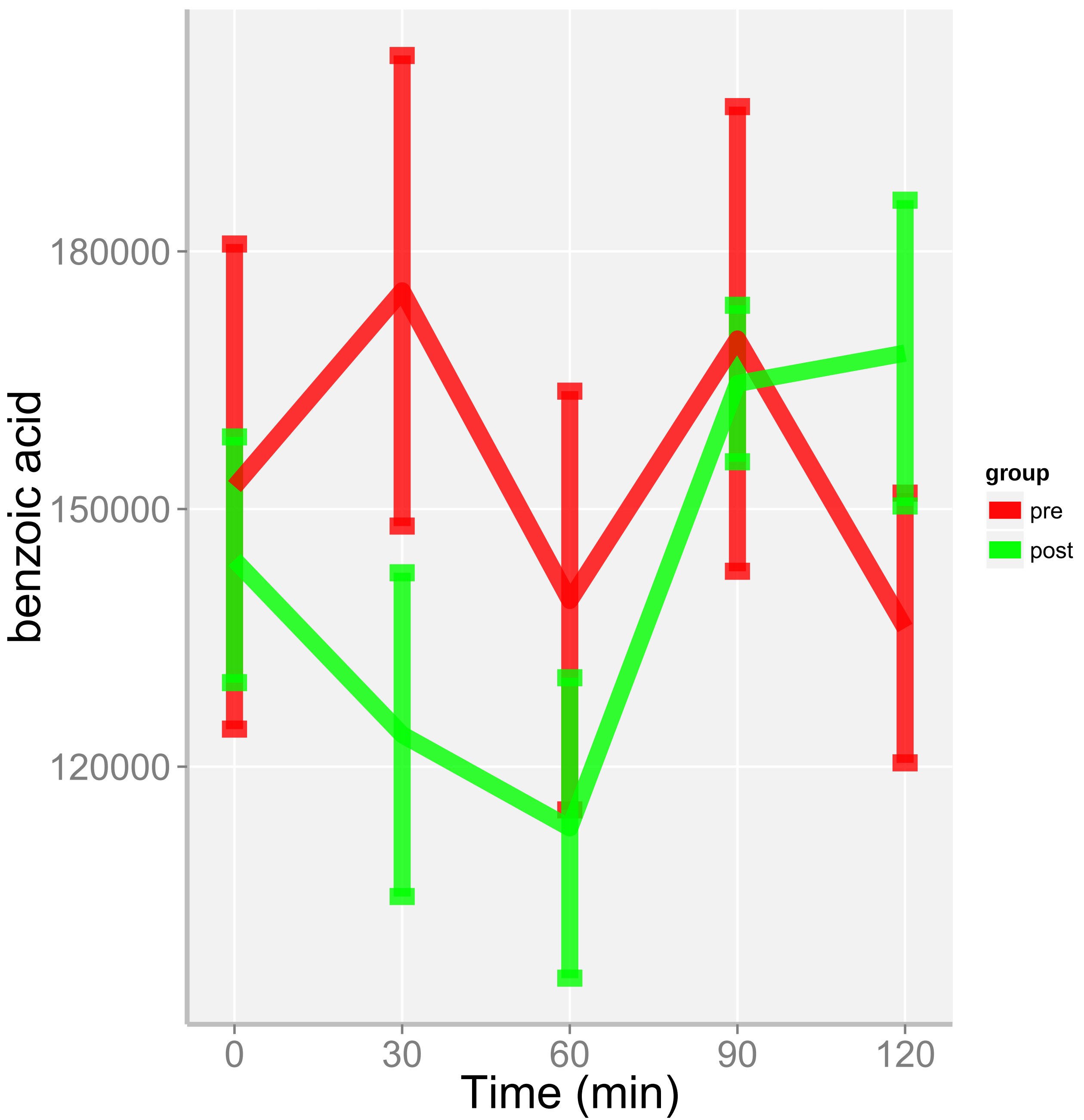

benzoic acid
180000
150000
120000
0
30
60
90
120
Time (min)
group
pre
post

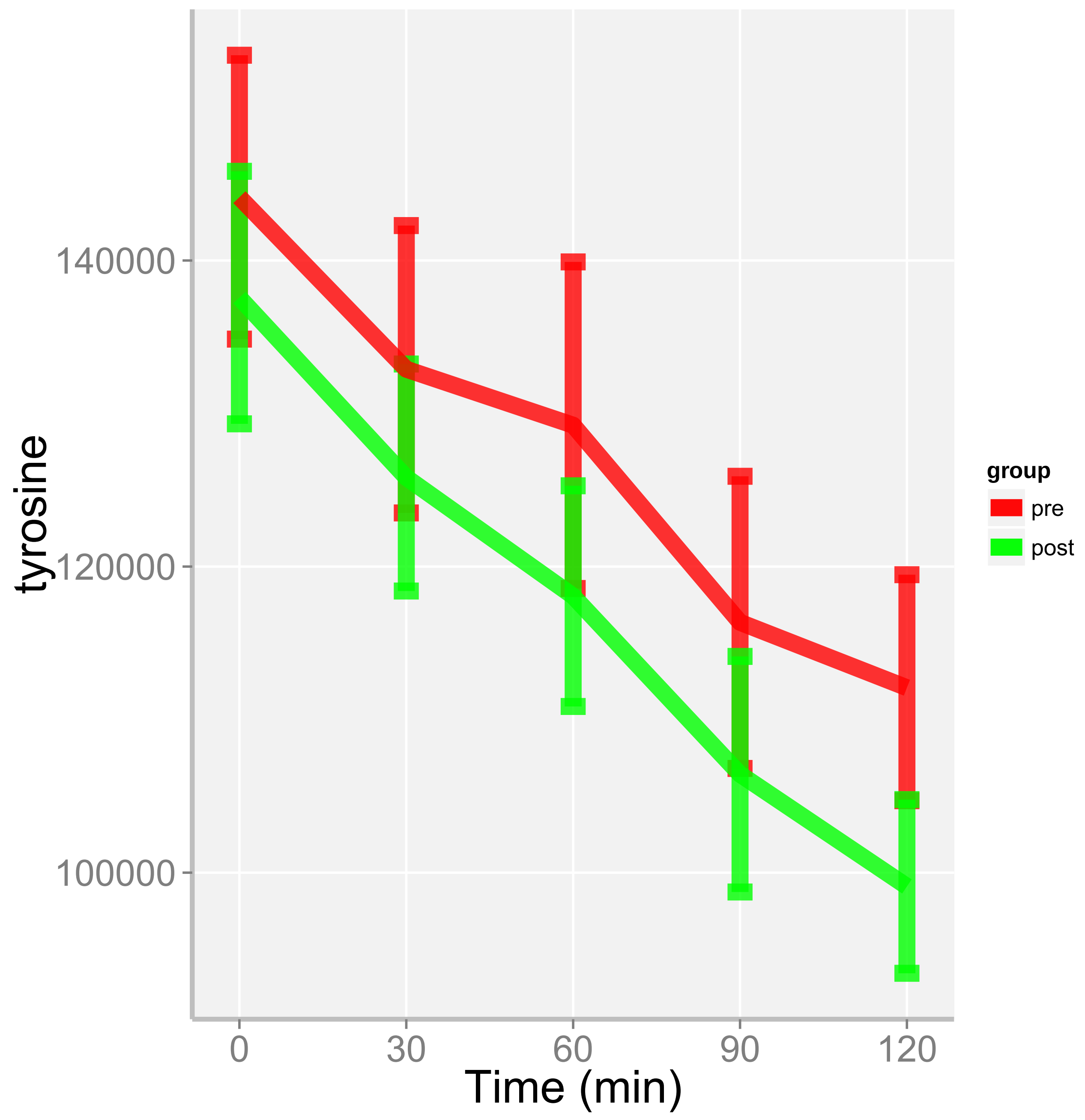

tyrosine
140000
120000
100000
0
30
60
90
120
Time (min)
group
pre
post

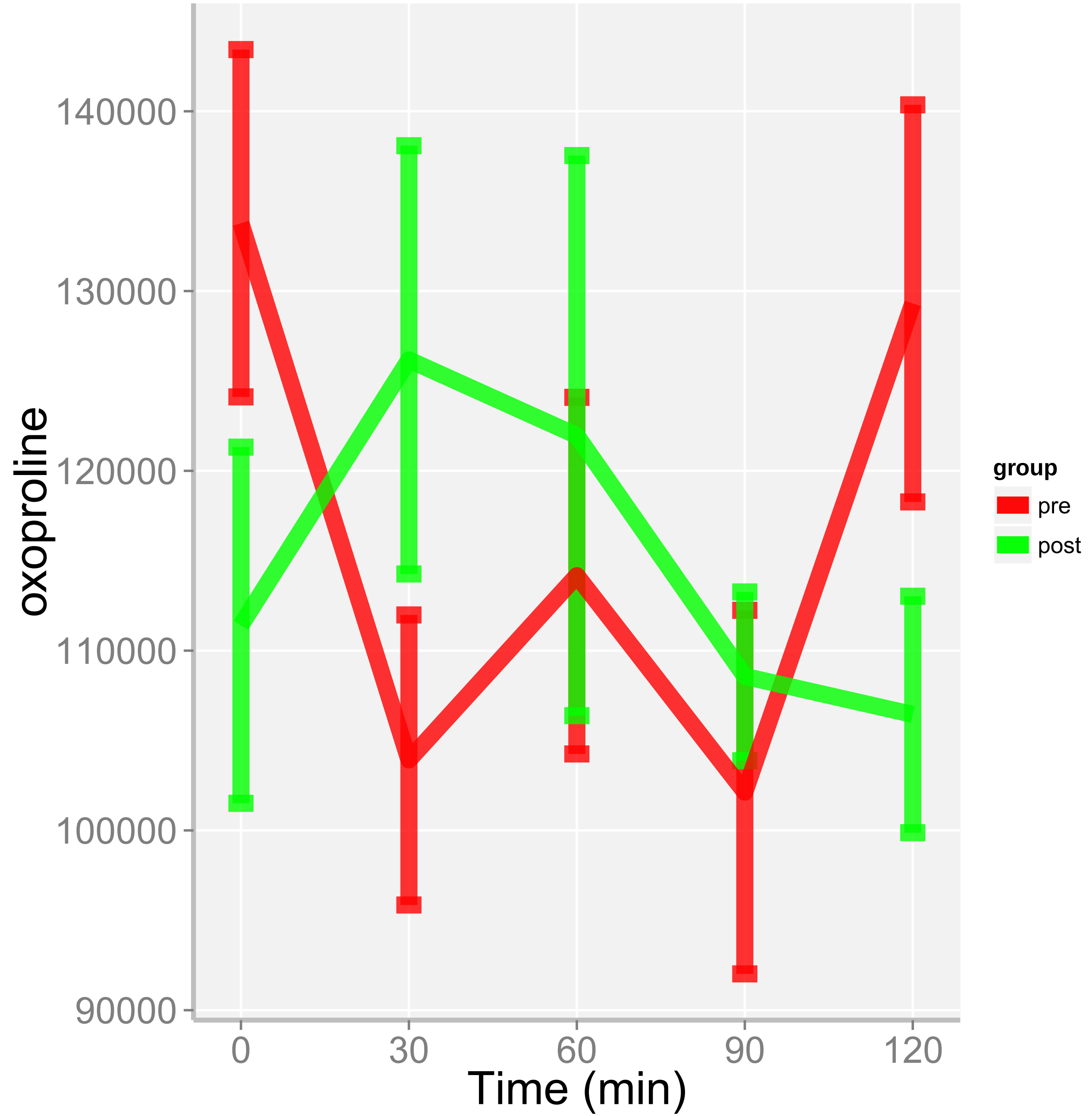

140000
130000
120000
110000
100000
90000
oxoproline
0
30
60
90
120
Time (min)
group
pre
post

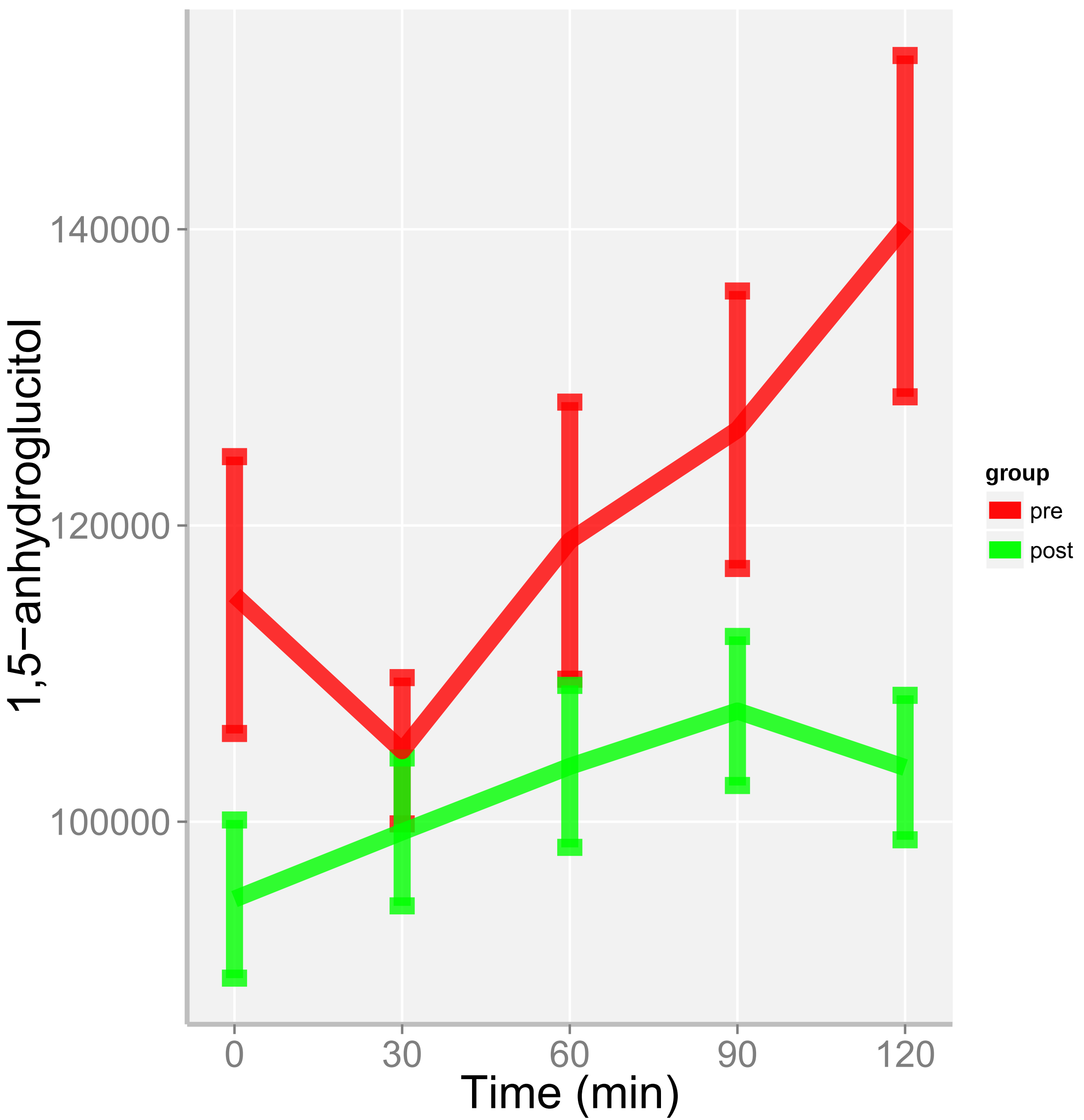

1,5−anhydroglucitol
140000
120000
100000
0
30
60
90
120
Time (min)
group
pre
post

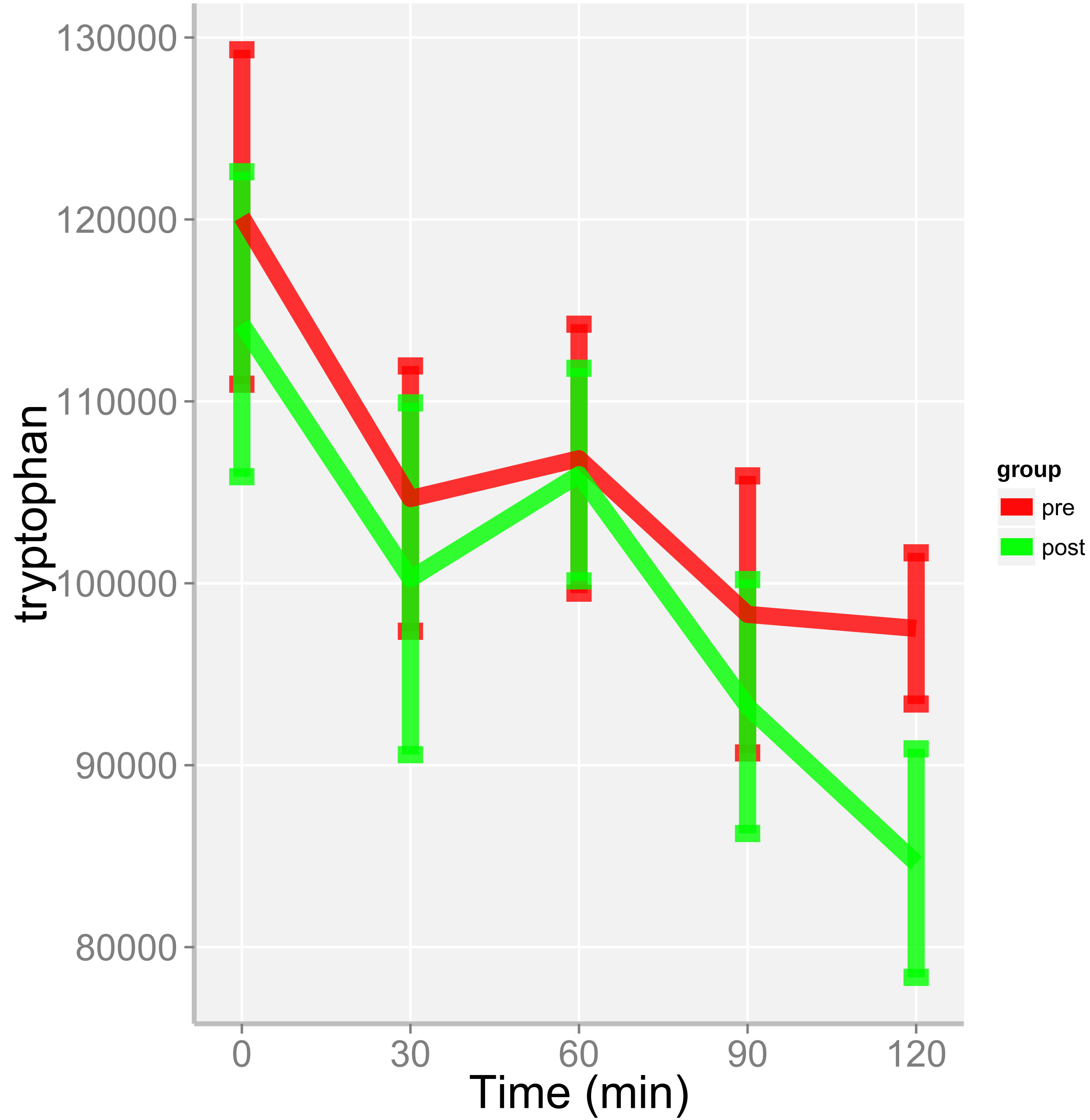

130000
120000
110000
100000
90000
80000
tryptophan
0
30
60
90
120
Time (min)
group
pre
post

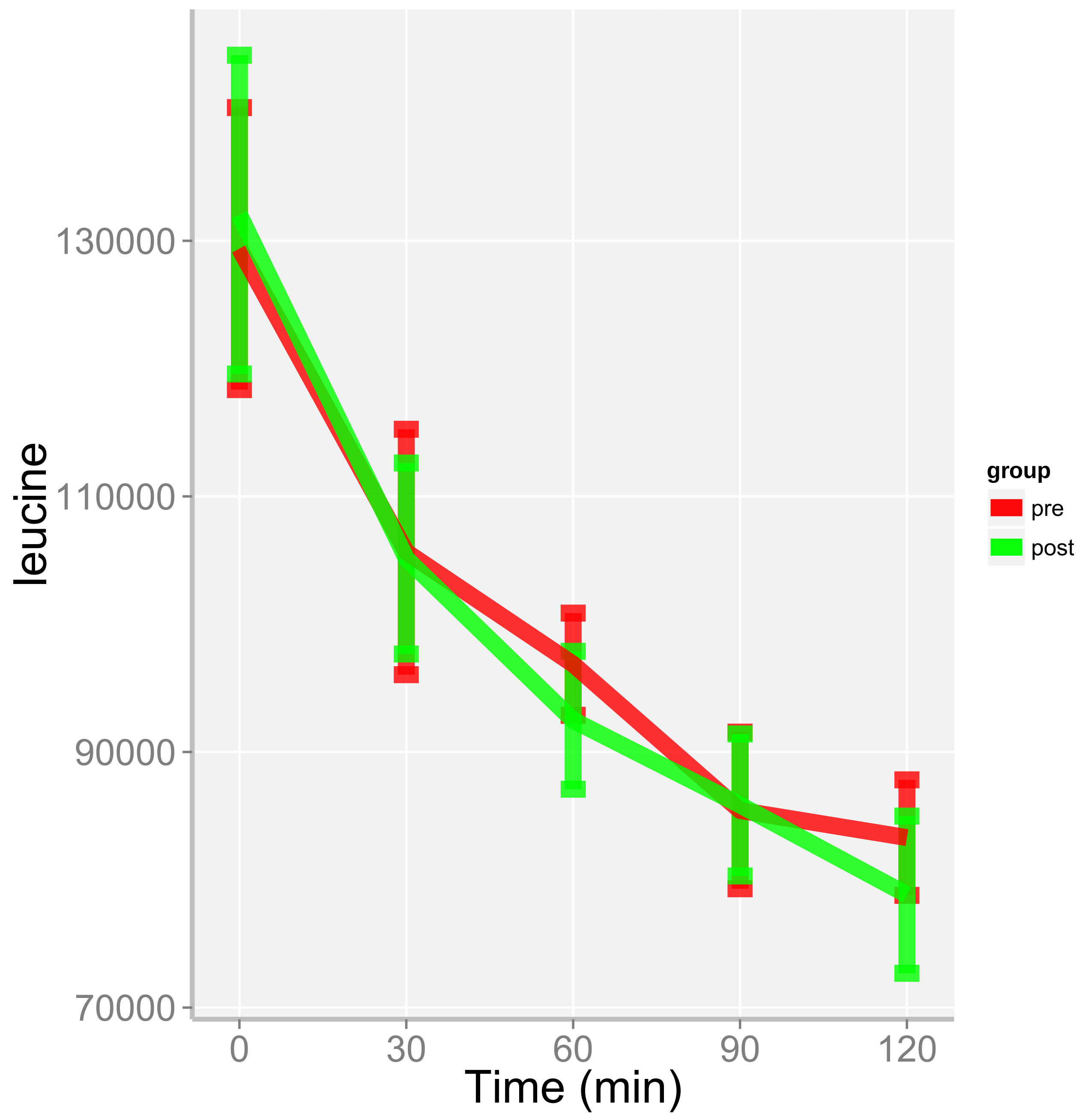

leucine
130000
110000
90000
70000
0
30
60
90
120
Time (min)
group
pre
post

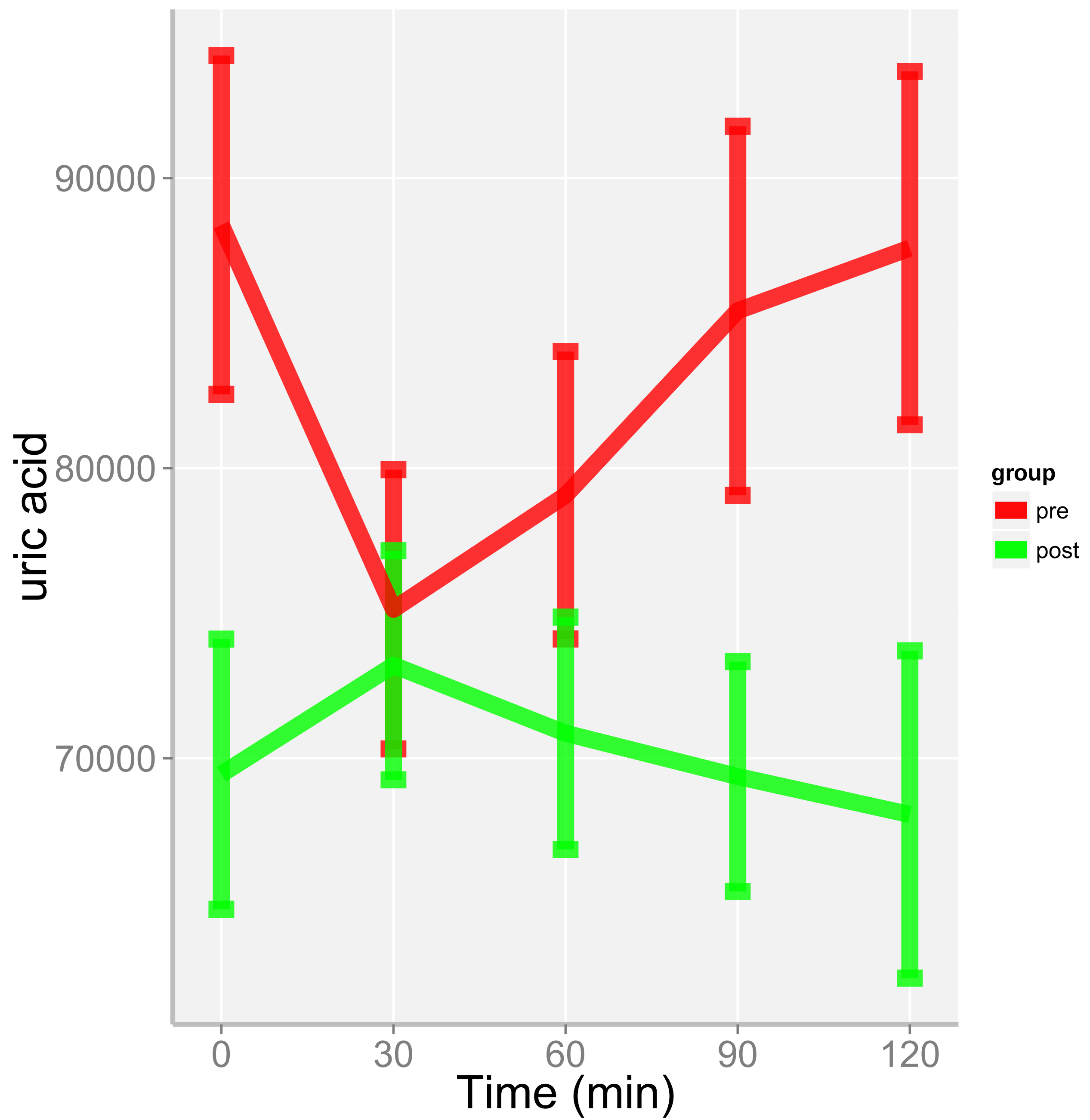
uric acid
90000
80000
70000
0
30
60
90
120
Time (min)
group
pre
post

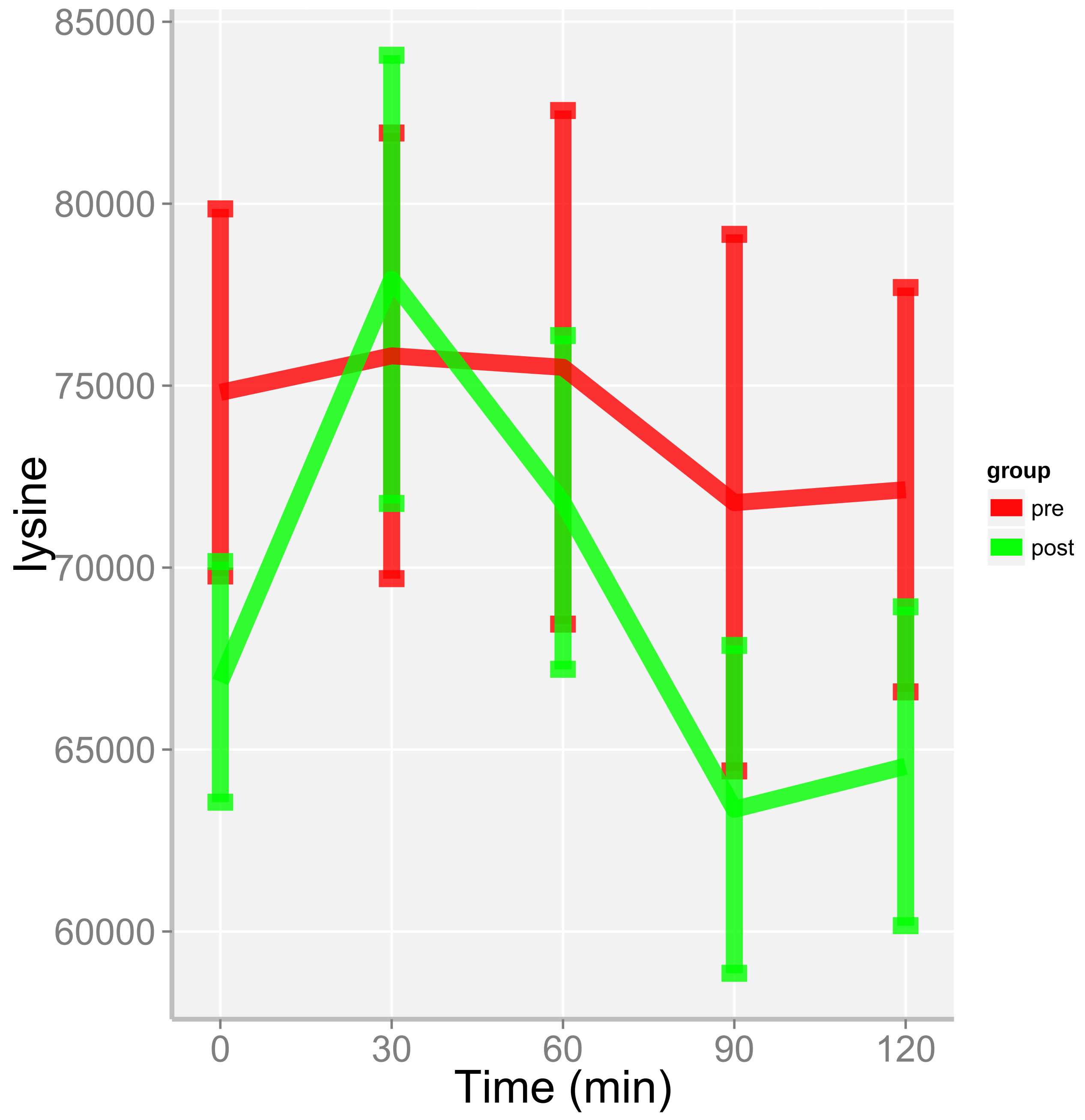

85000
80000
75000
70000
65000
60000
lysine
0
30
60
90
120
Time (min)
group
pre
post

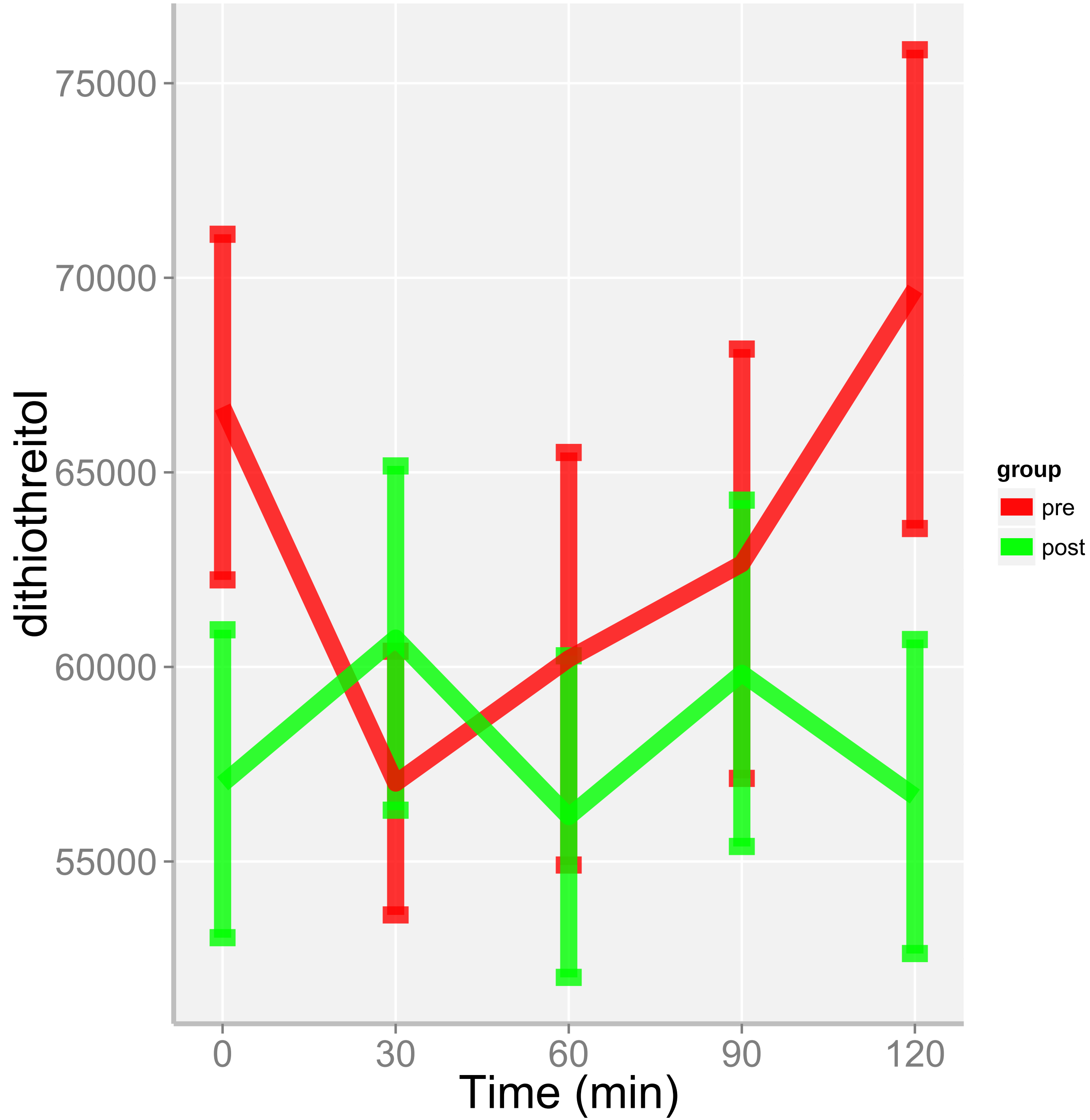

75000
70000
65000
60000
55000
dithiothreitol
0
30
60
90
120
Time (min)
group
pre
post

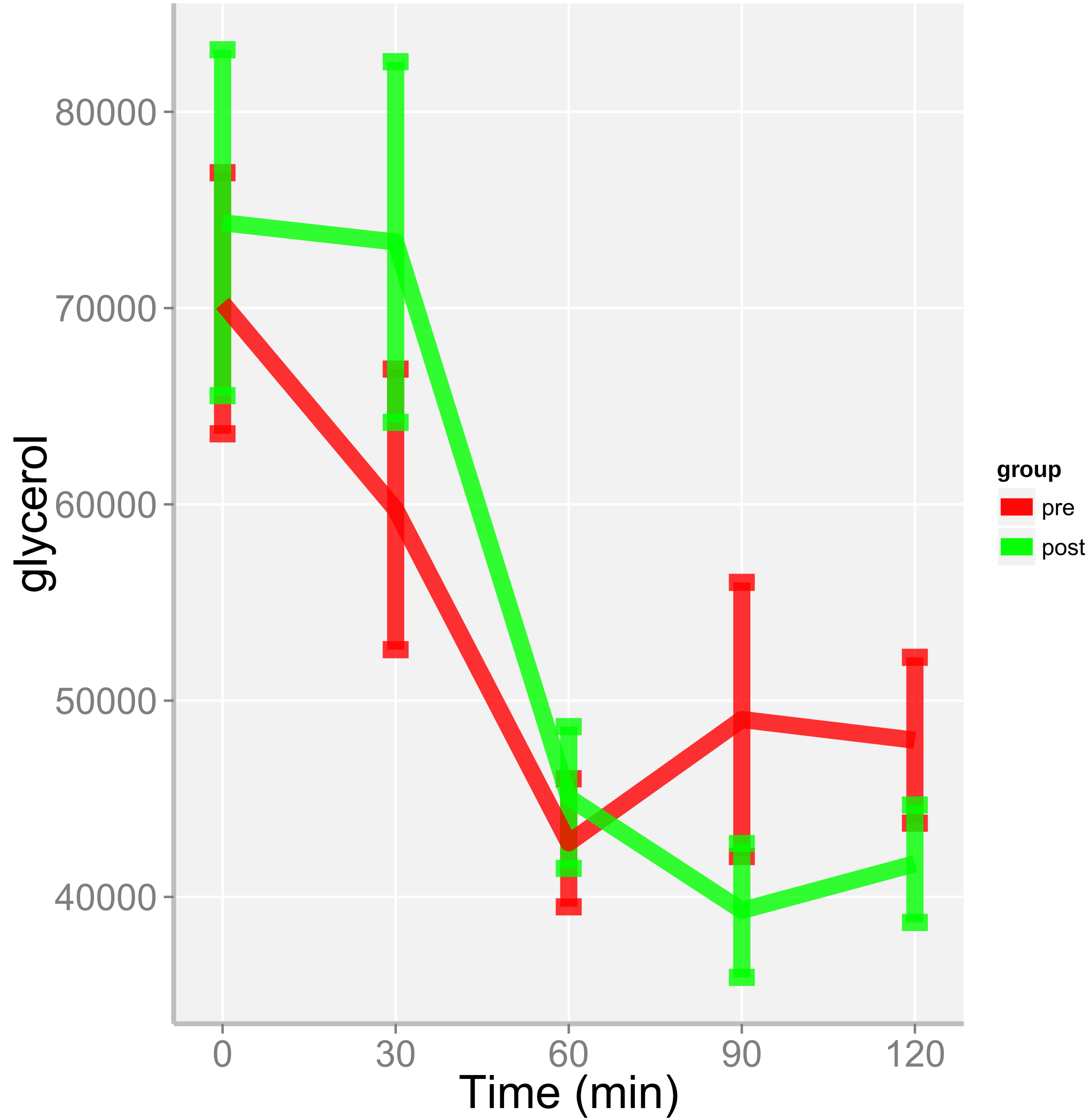

80000
70000
60000
50000
40000
glycerol
0
30
60
90
120
Time (min)
group
pre
post

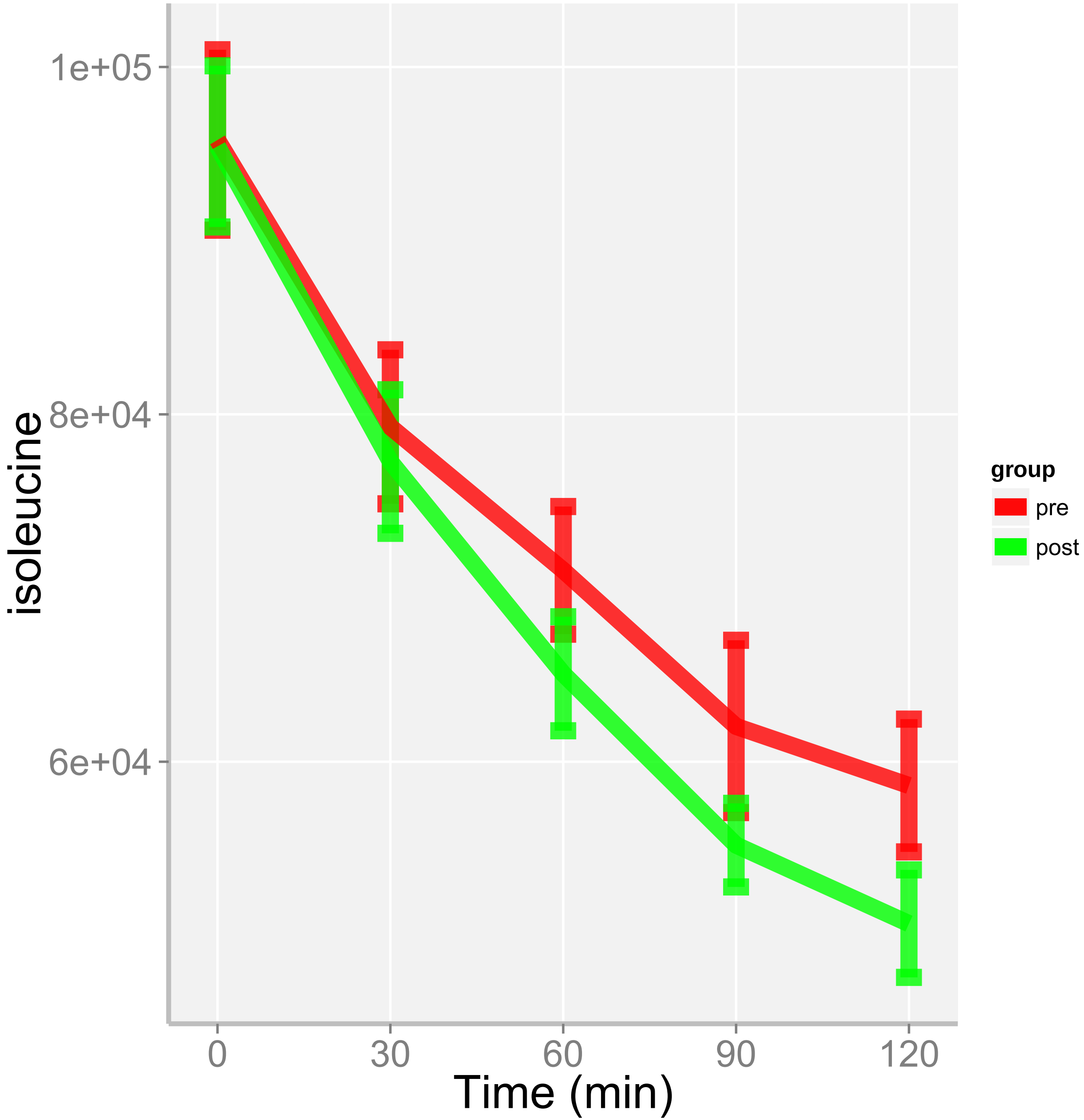

isoleucine
1e+05
8e+04
6e+04
0
30
60
90
120
Time (min)
group
pre
post

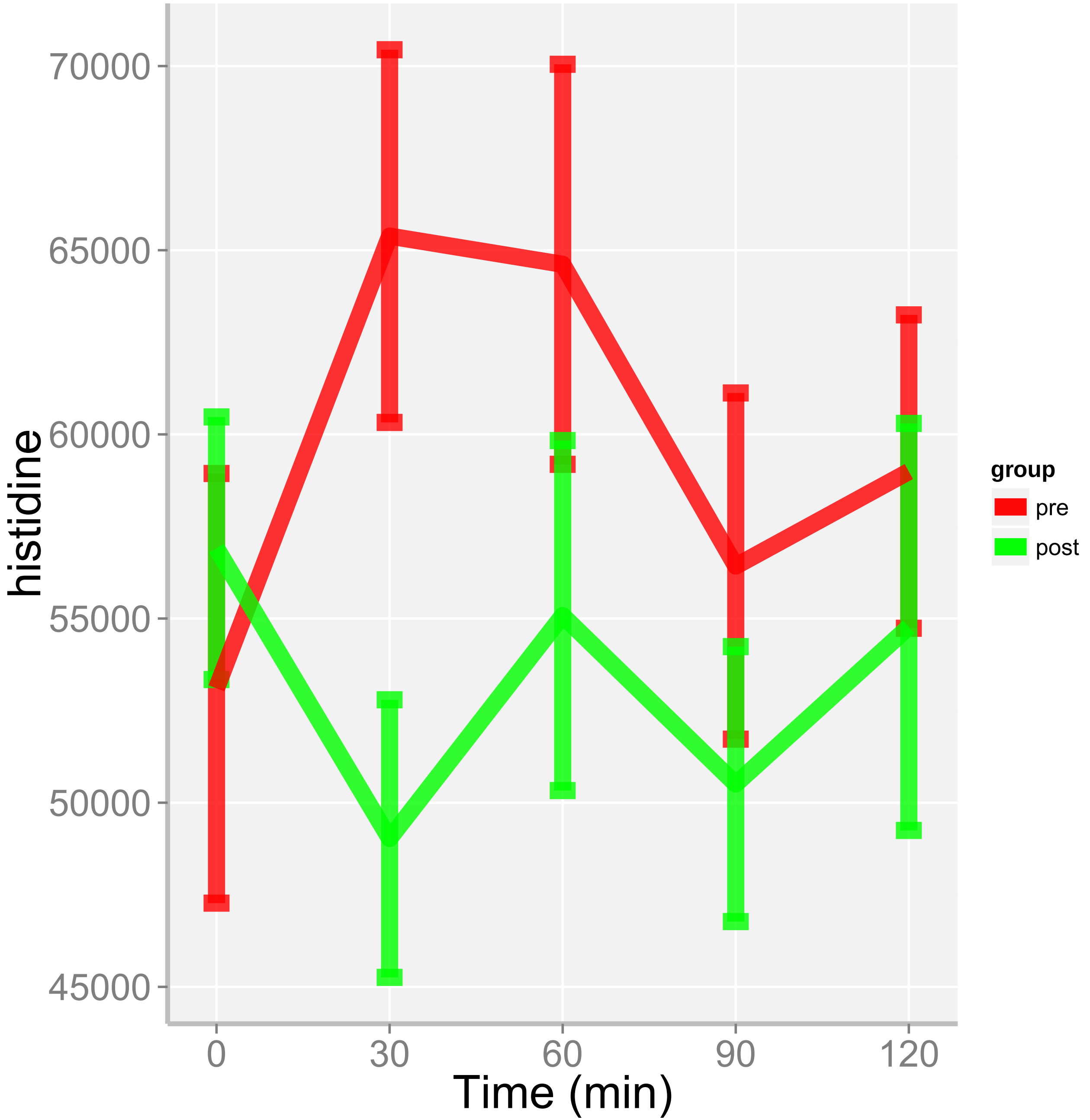

histidine
70000
65000
60000
55000
50000
45000
0
30
60
90
120
Time (min)
group
pre
post

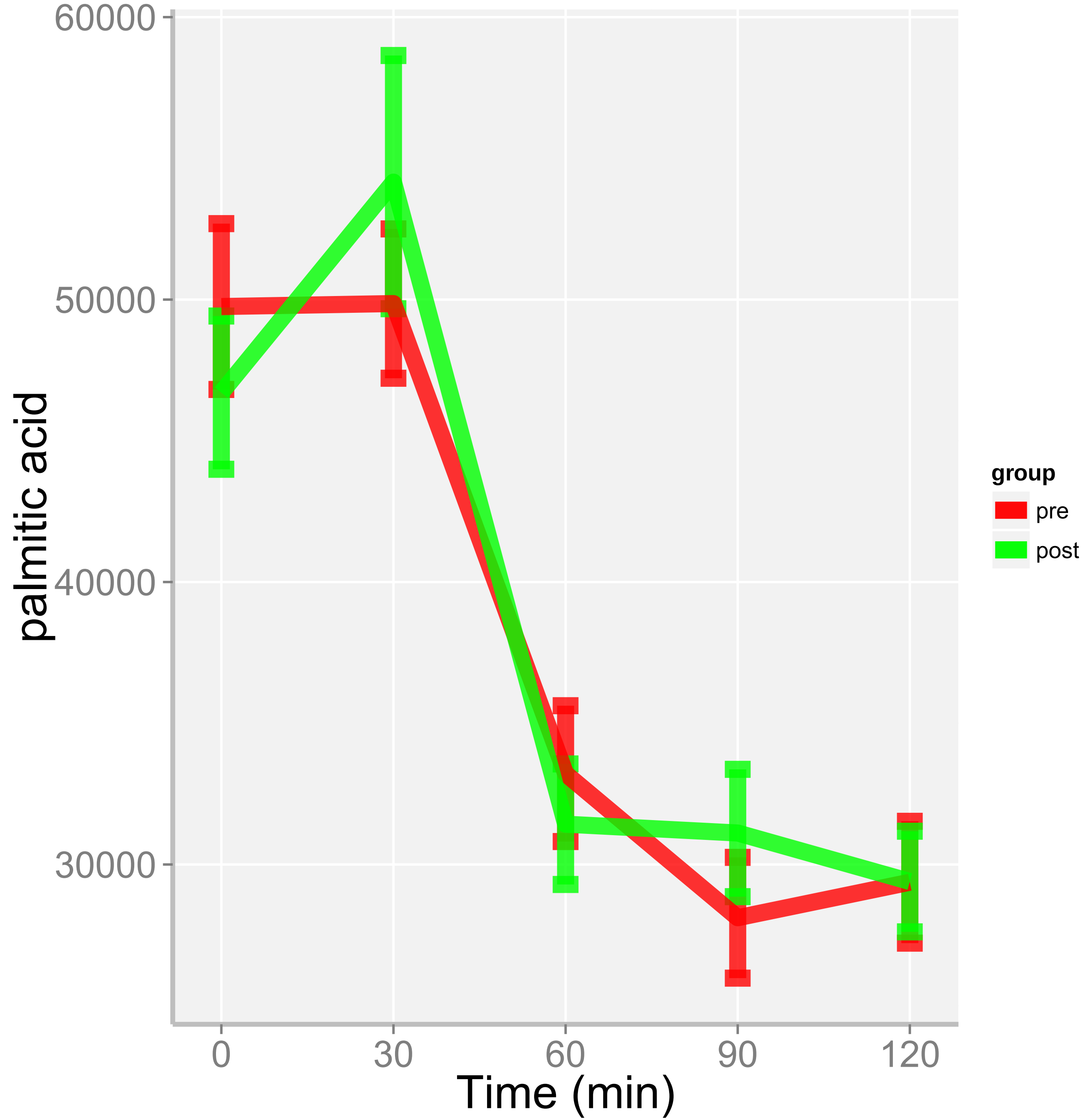

palmitic acid
60000
50000
40000
30000
0
30
60
90
120
Time (min)
group
pre
post

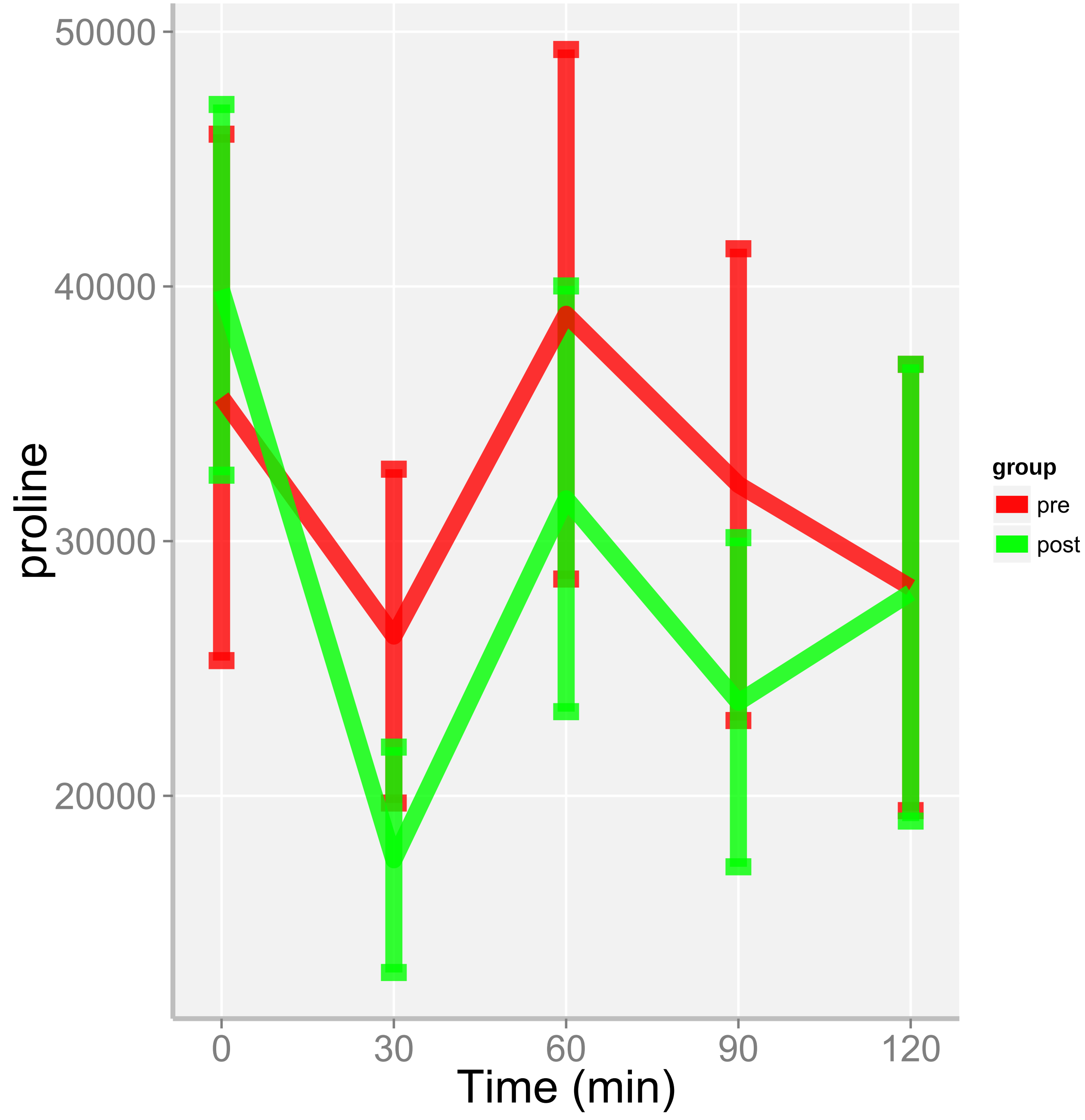

50000
40000
30000
20000
proline
0
30
60
90
120
Time (min)
group
pre
post

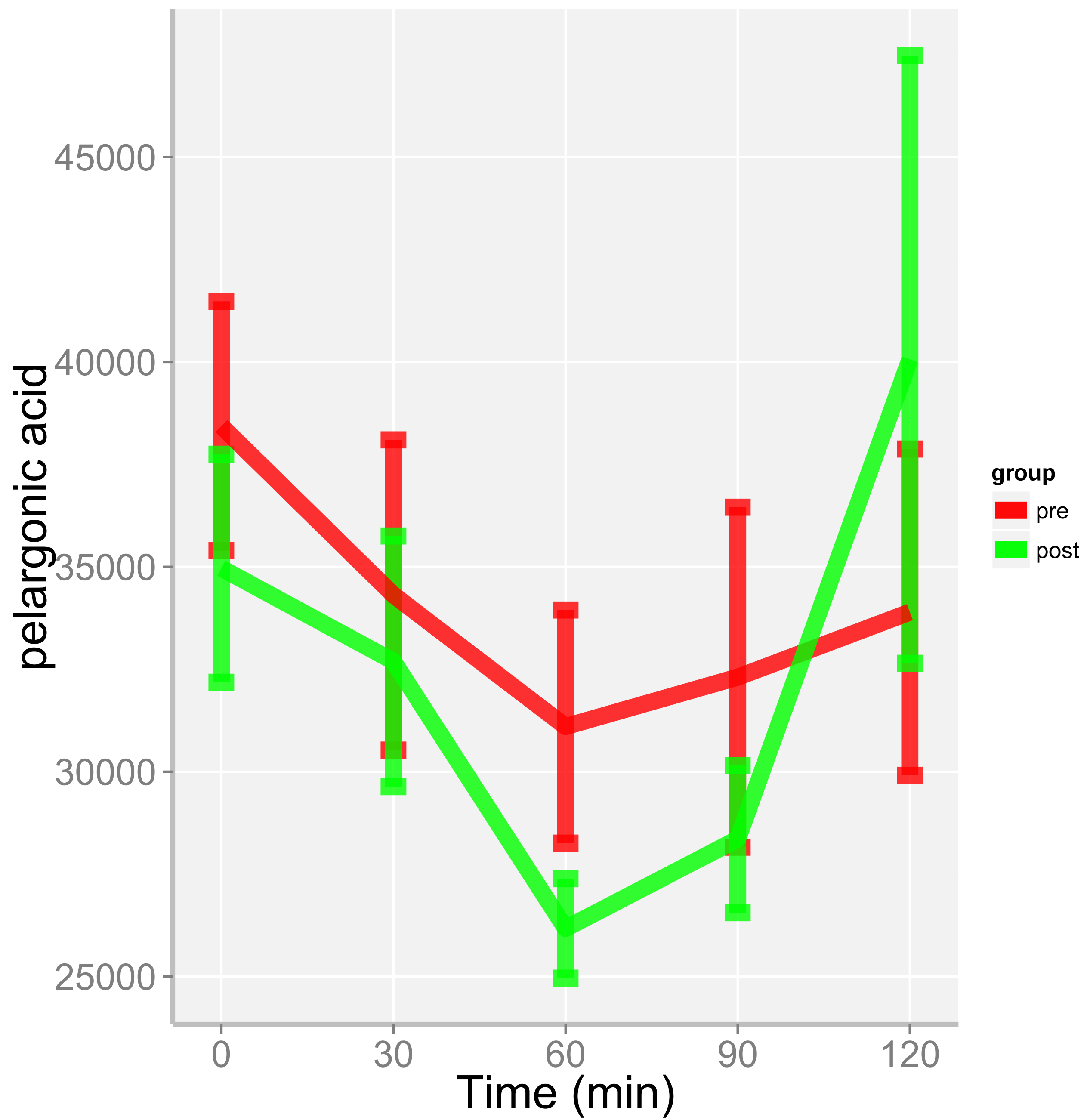

pelargonic acid
45000
40000
35000
30000
25000
0
30
60
90
120
Time (min)
group
pre
post

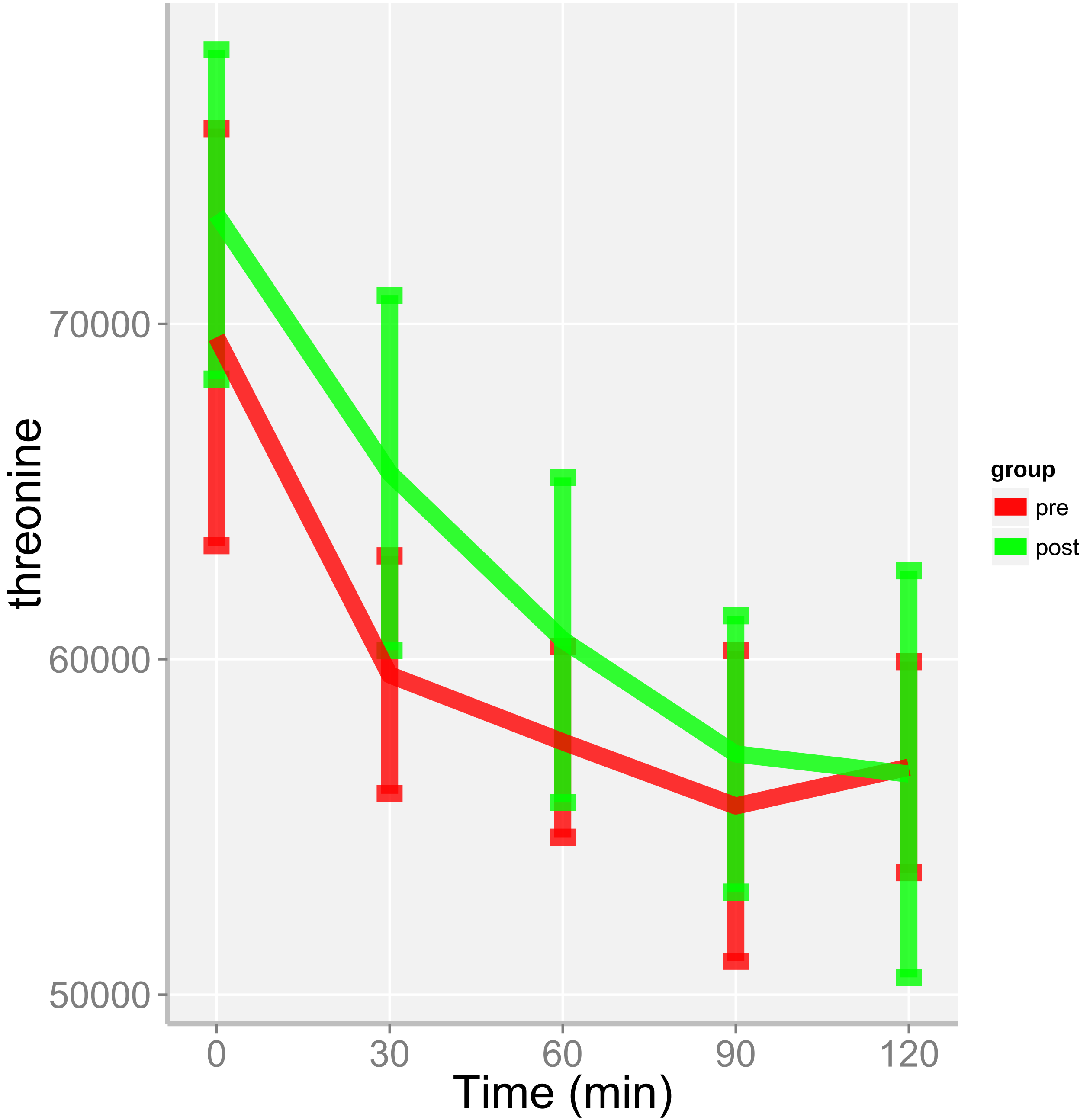
threonine
70000
60000
50000
0
30
60
90
120
Time (min)
group
pre
post

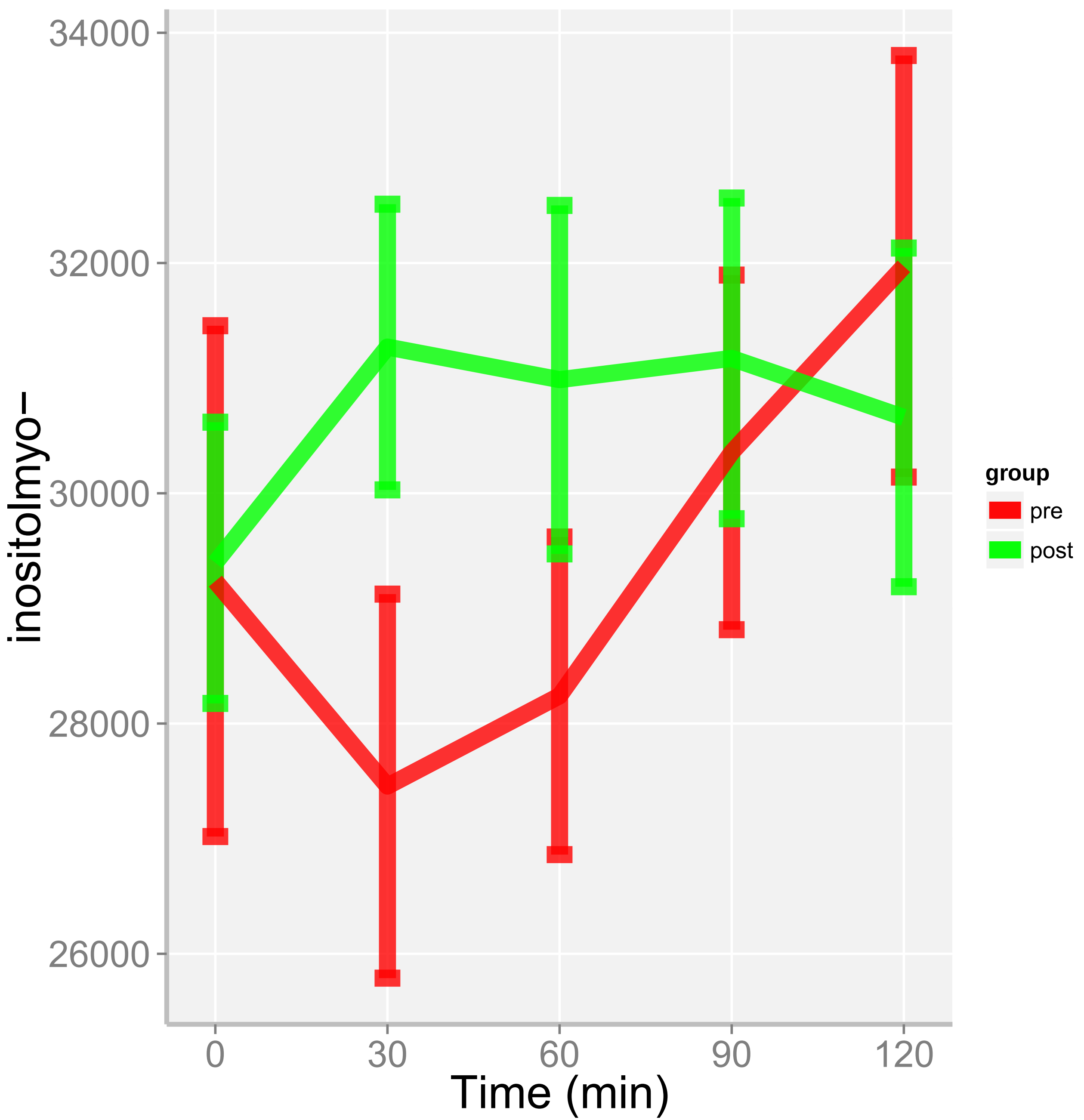
34000
32000
30000
28000
26000
inositolmyo−
0
30
60
90
120
Time (min)
group
pre
post

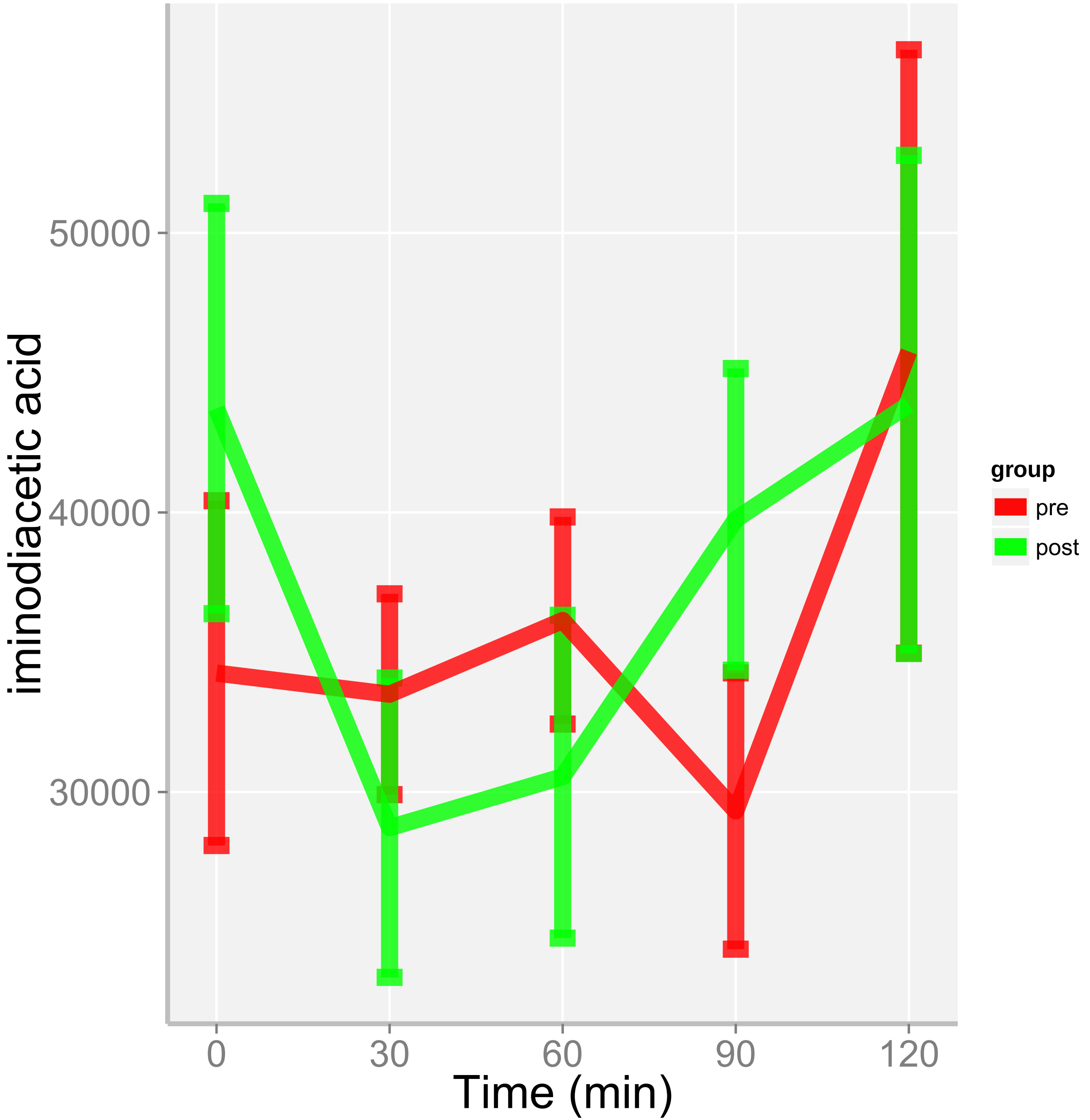

iminodiacetic acid
50000
40000
30000
0
30
60
90
120
Time (min)
group
pre
post

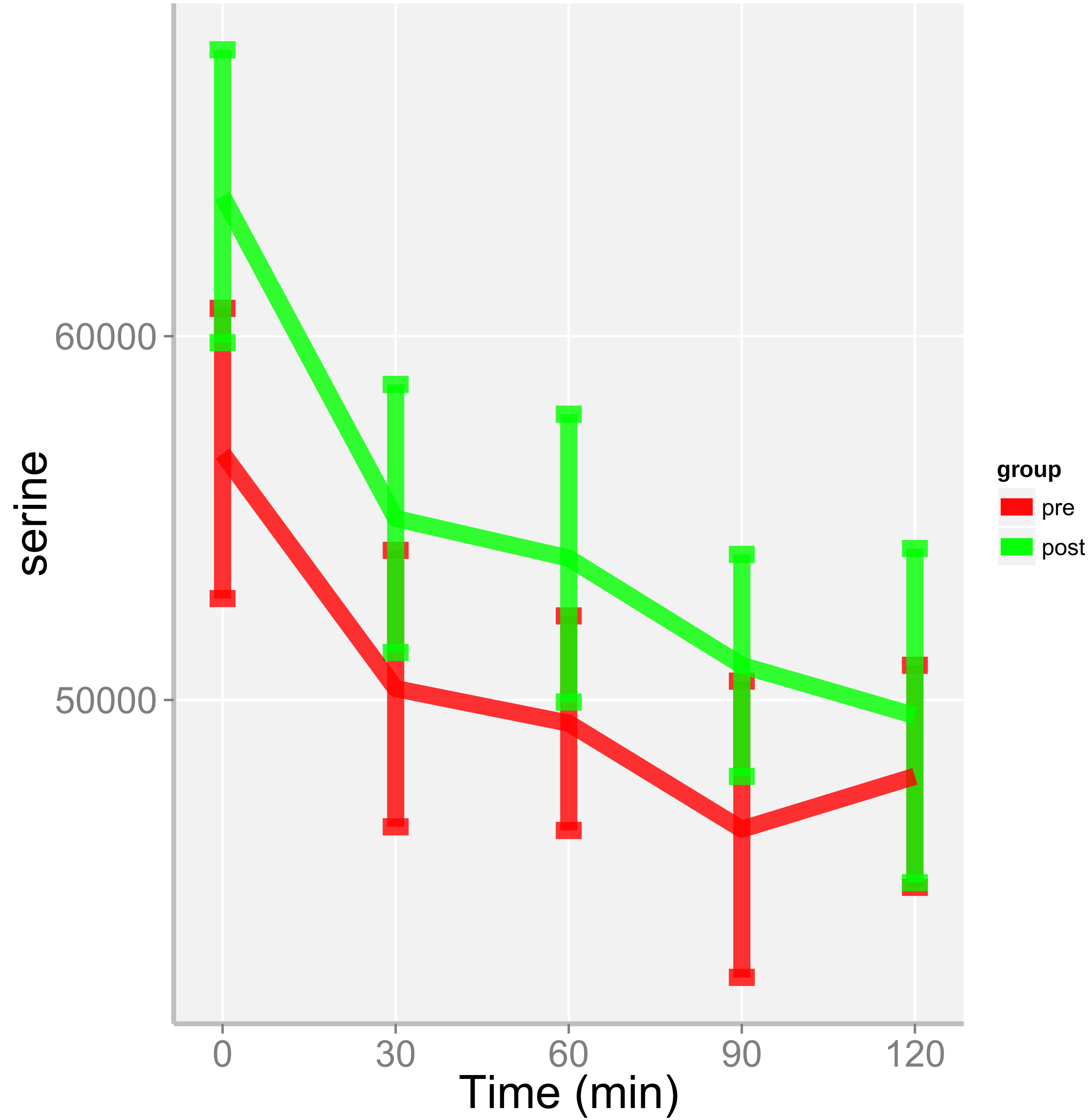

serine
60000
50000
0
30
60
90
120
Time (min)
group
pre
post

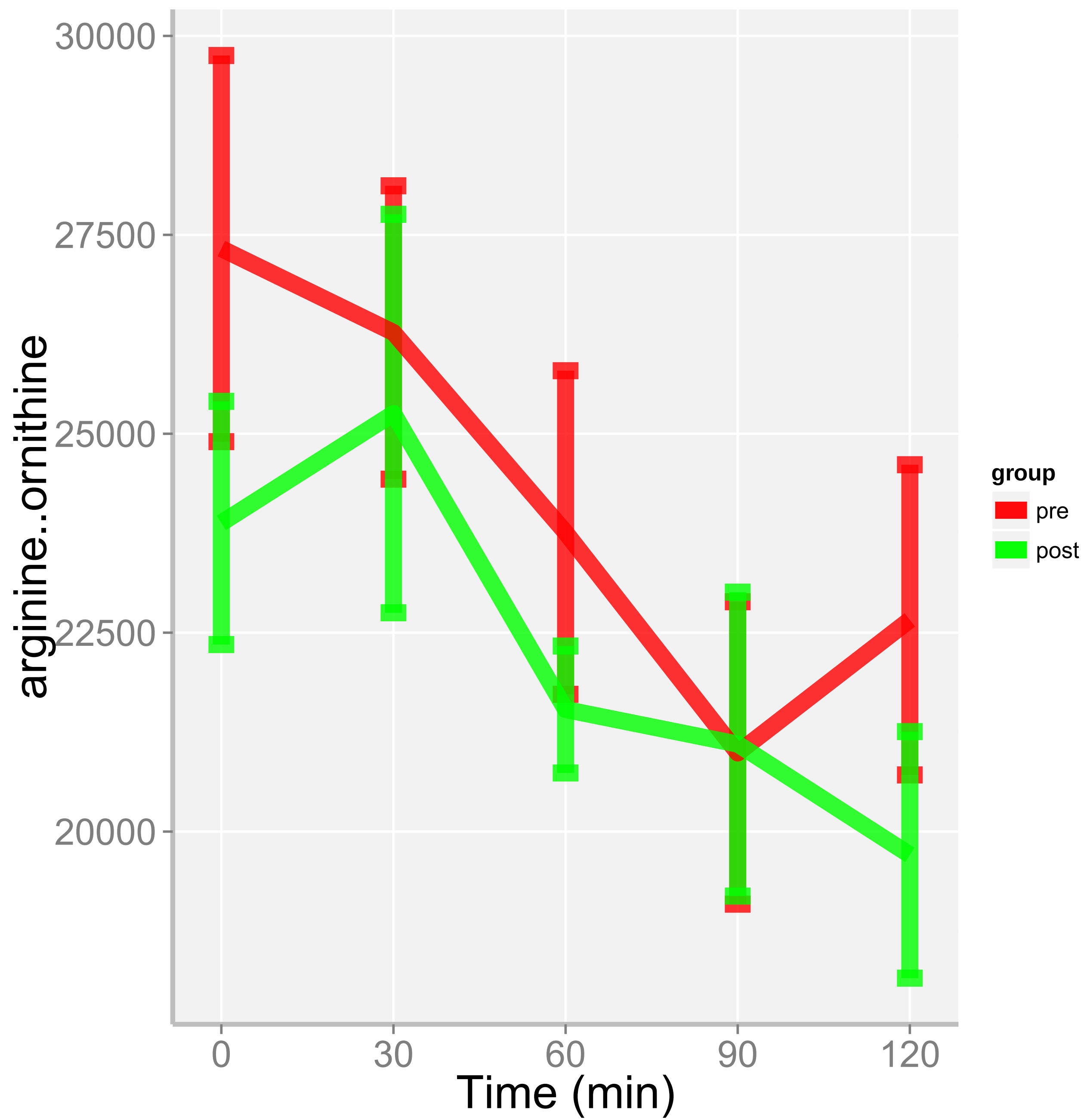

30000
27500
25000
22500
20000
arginine..ornithine
0
30
60
90
120
Time (min)
group
pre
post

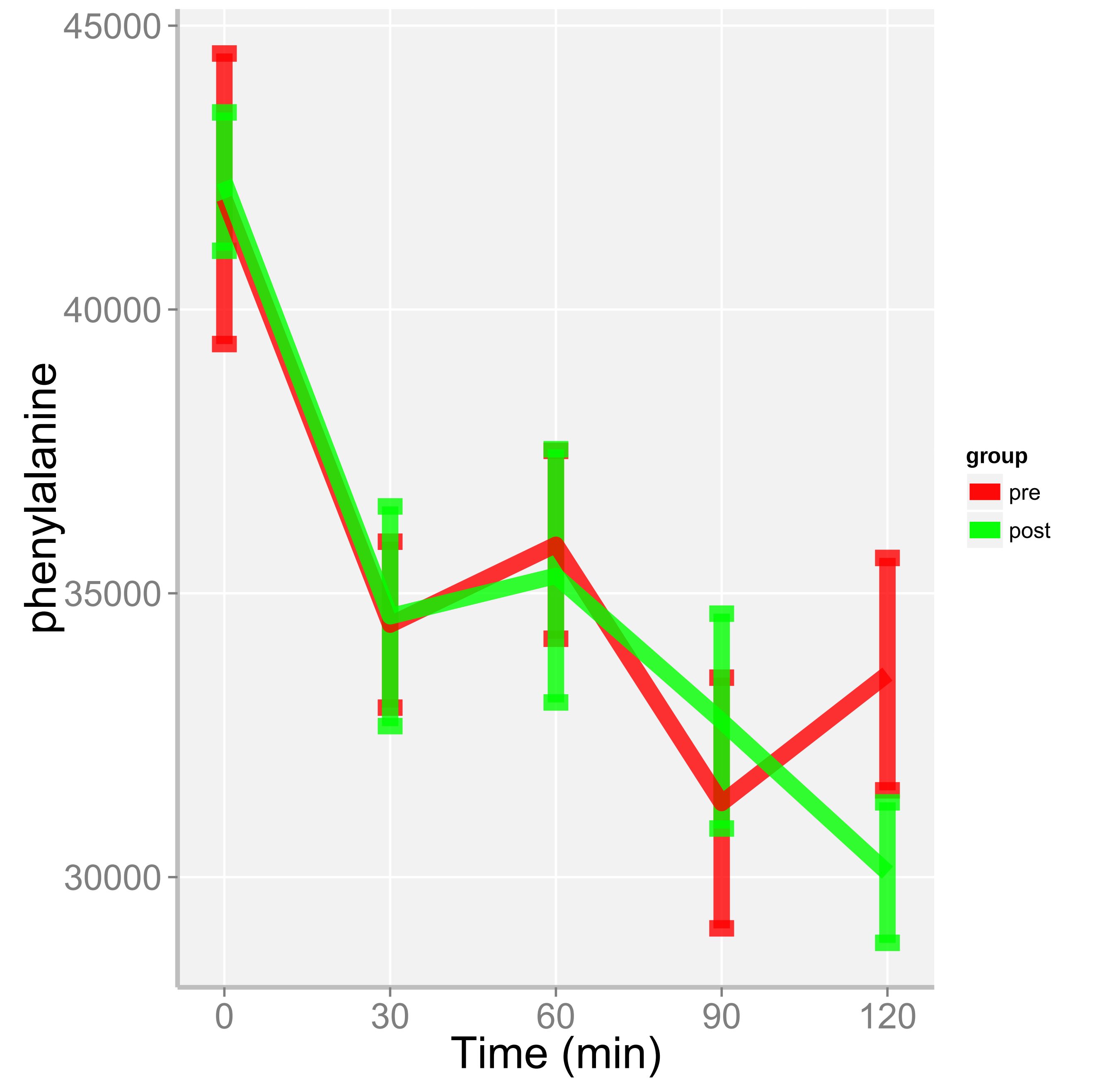

45000
40000
35000
30000
phenylalanine
0
30
60
90
120
Time (min)
group
pre
post

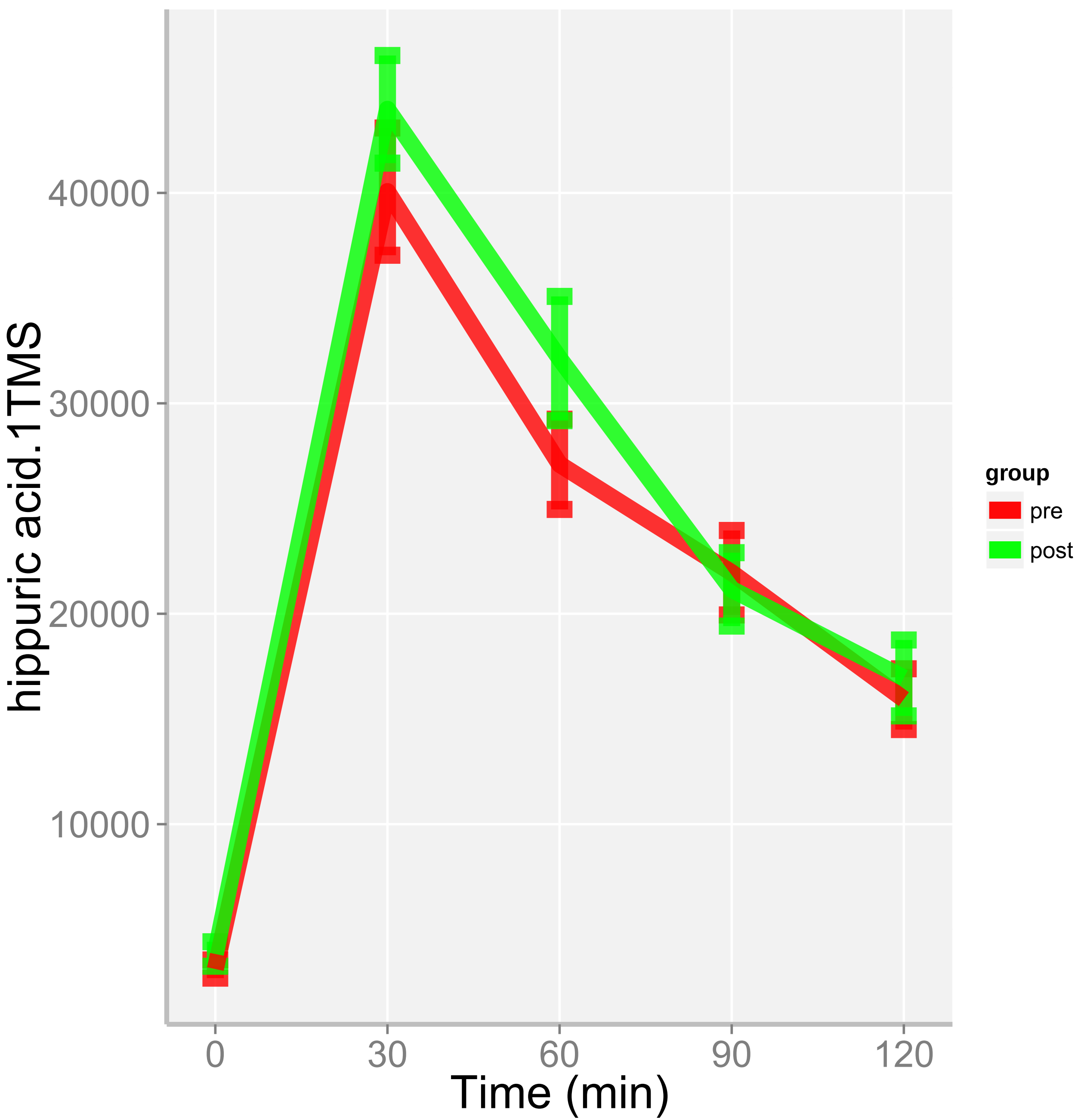

hippuric acid.1TMS
40000
30000
20000
10000
0
30
60
90
120
Time (min)
group
pre
post

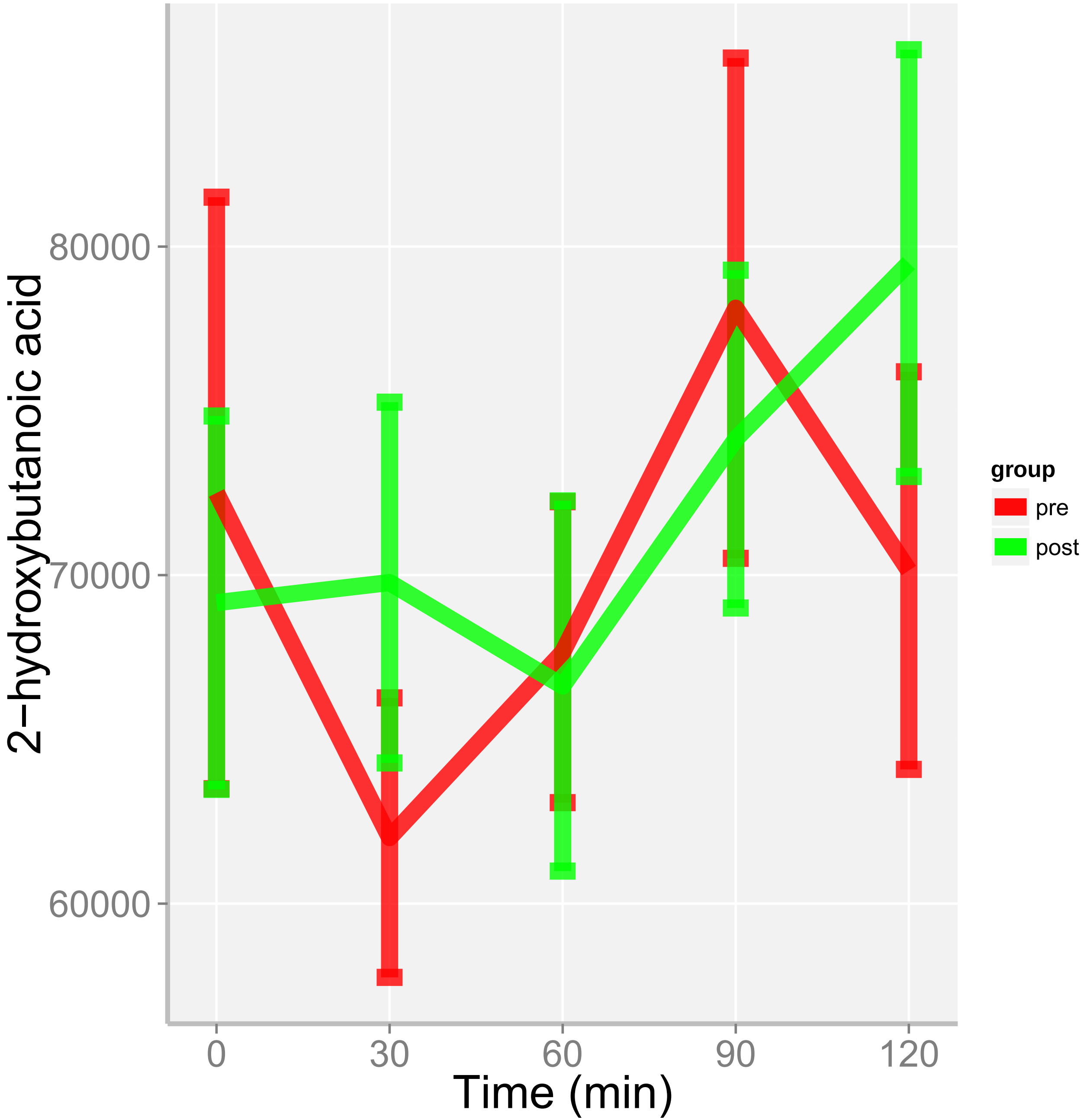
2−hydroxybutanoic acid
80000
70000
60000
0
30
60
90
120
Time (min)
group
pre
post

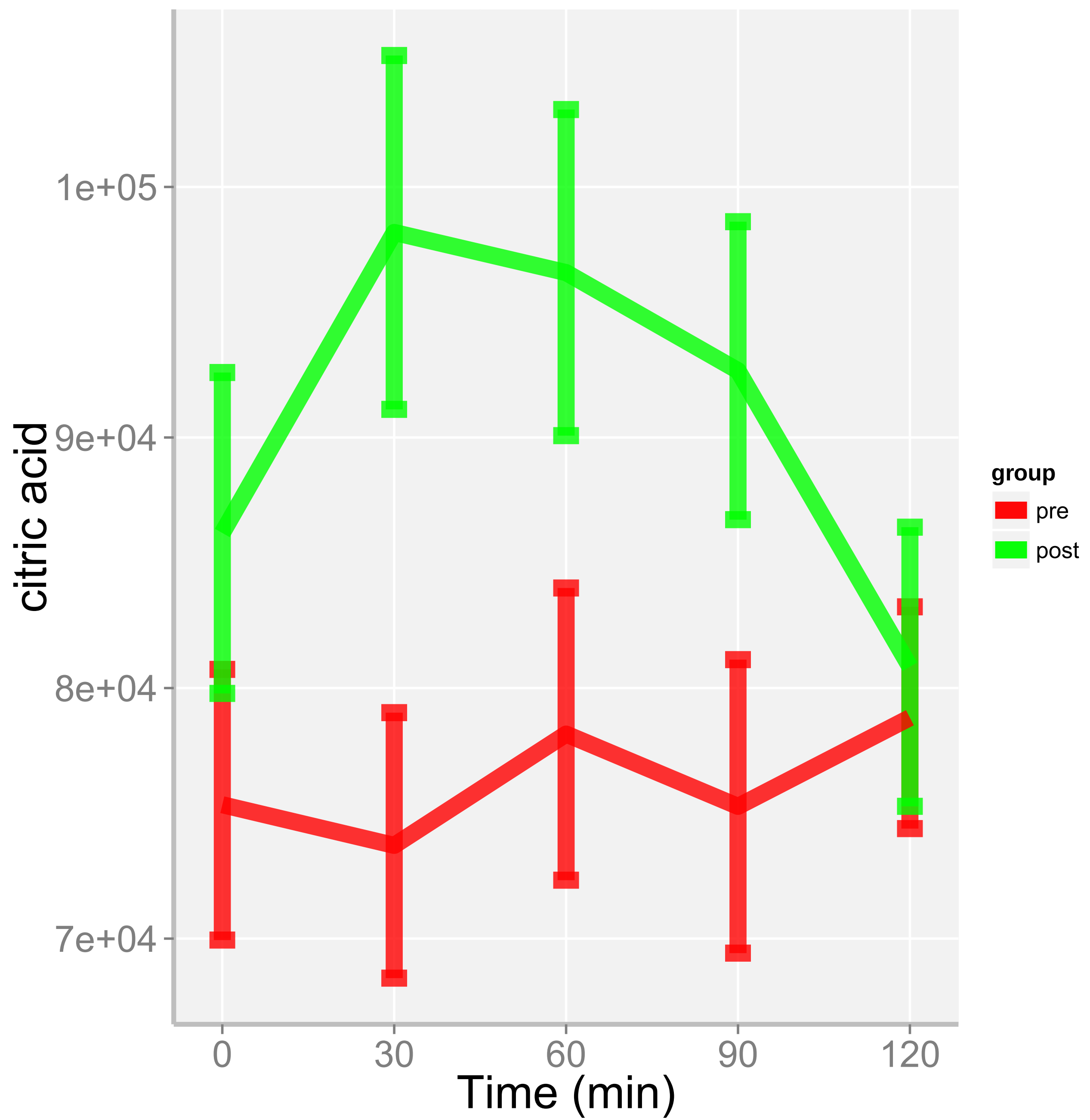

citric acid
1e+05
9e+04
8e+04
7e+04
0
30
60
90
120
Time (min)
group
pre
post

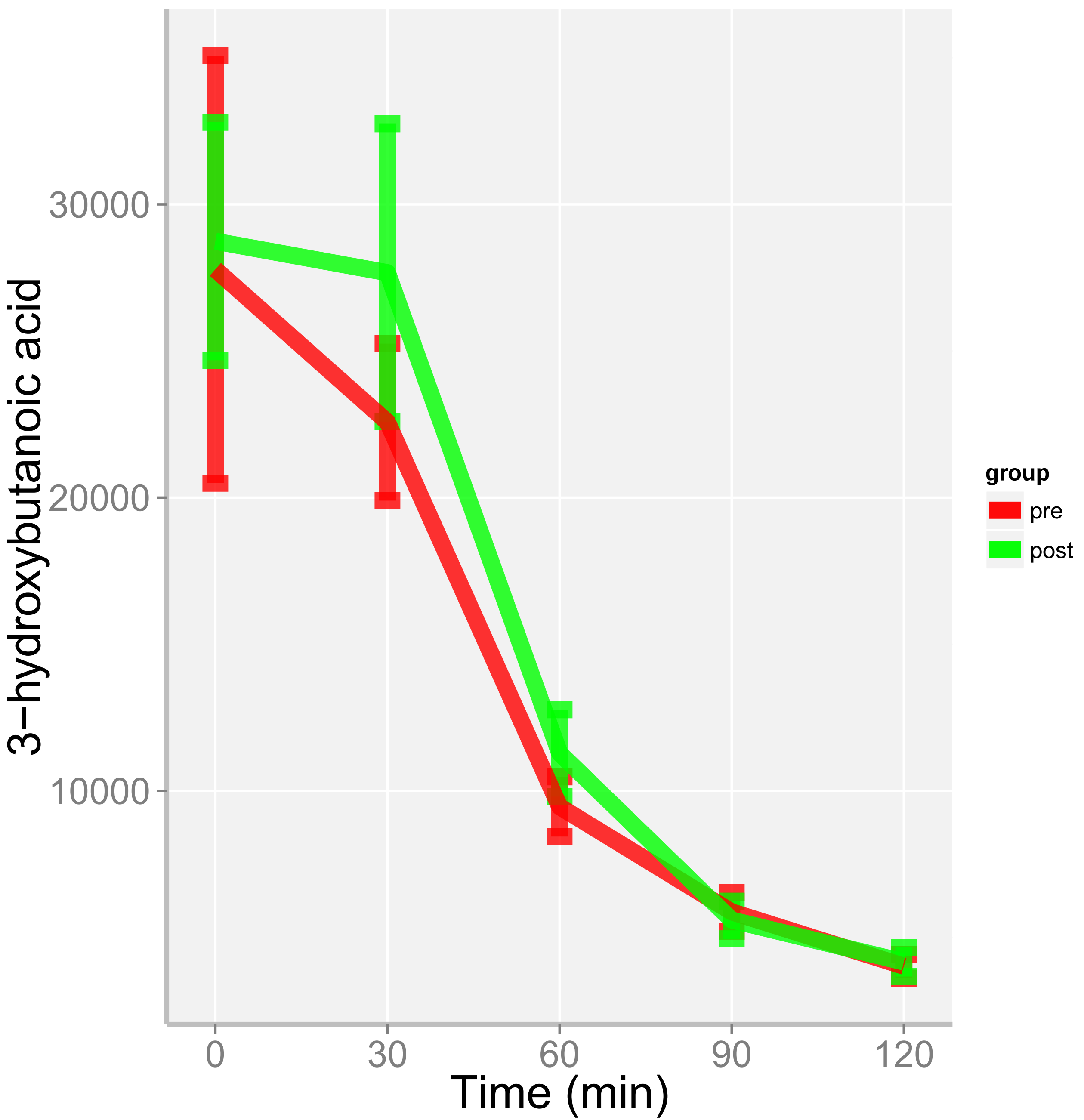

3−hydroxybutanoic acid
30000
20000
10000
0
30
60
90
120
Time (min)
group
pre
post

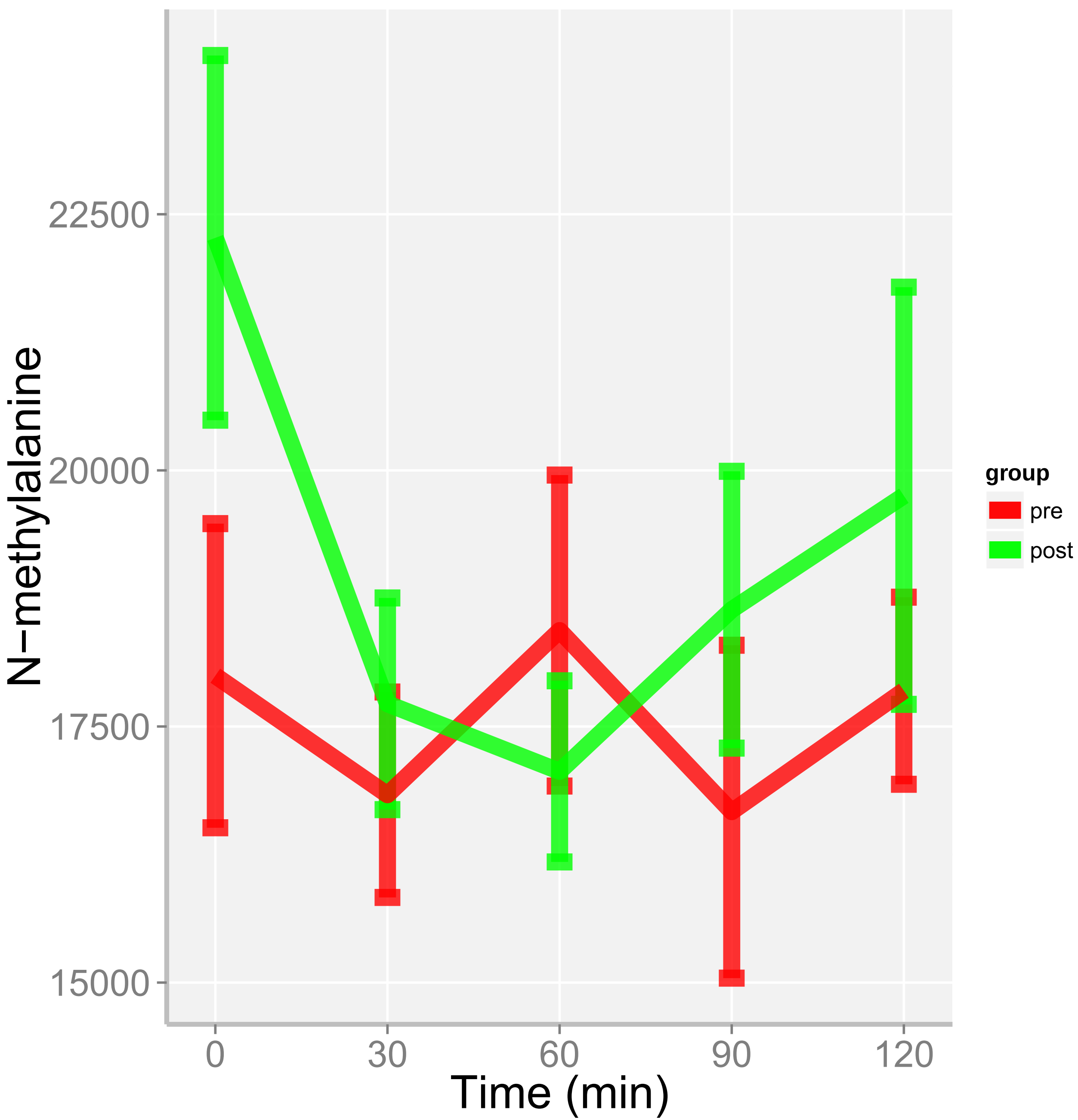
N−methylalanine
22500
20000
17500
15000
0
30
60
90
120
Time (min)
group
pre
post

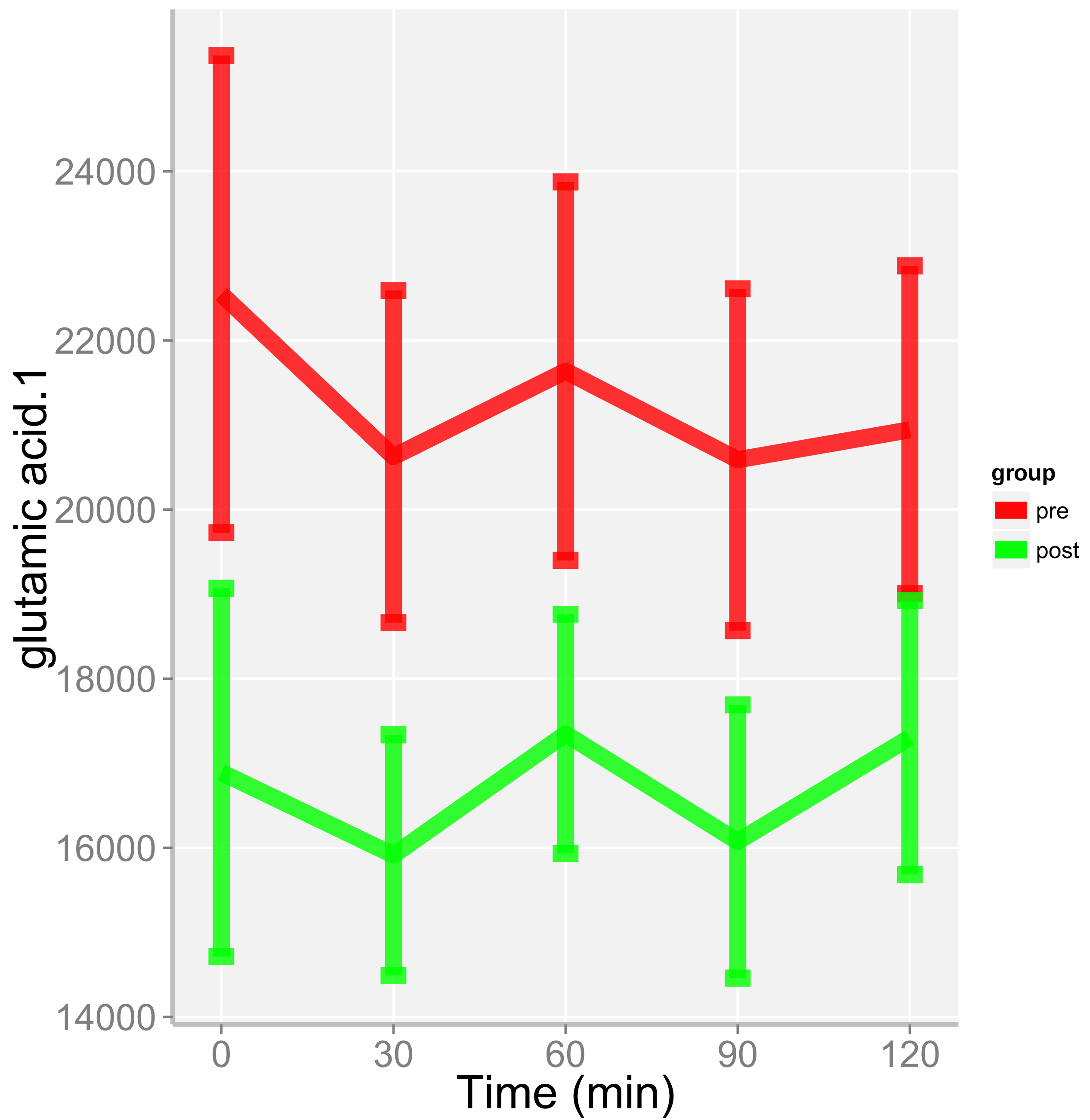

glutamic acid.1
24000
22000
20000
18000
16000
14000
0
30
60
90
120
Time (min)
group
pre
post

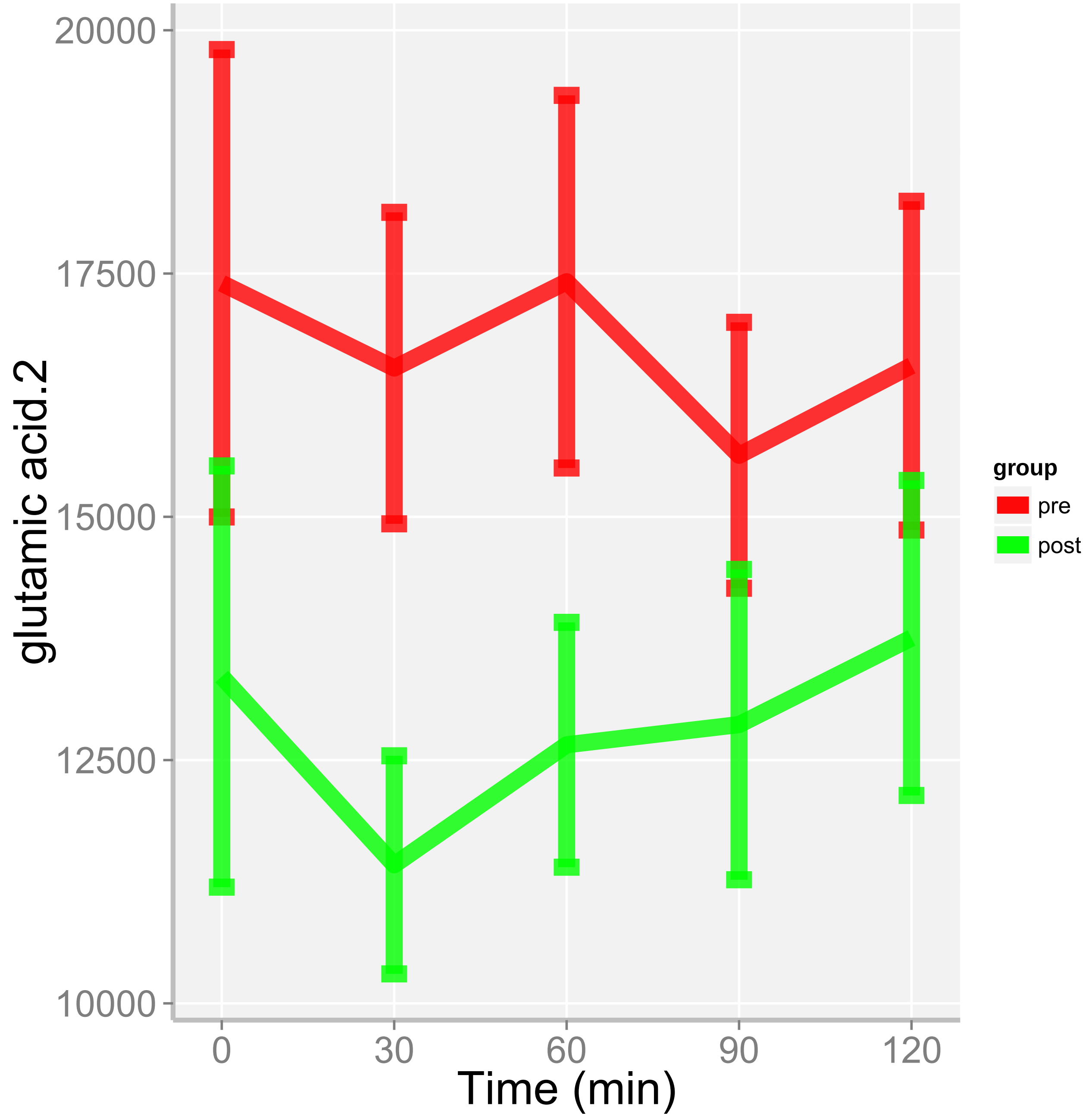

glutamic acid.2
20000
17500
15000
12500
10000
0
30
60
90
120
Time (min)
group
pre
post

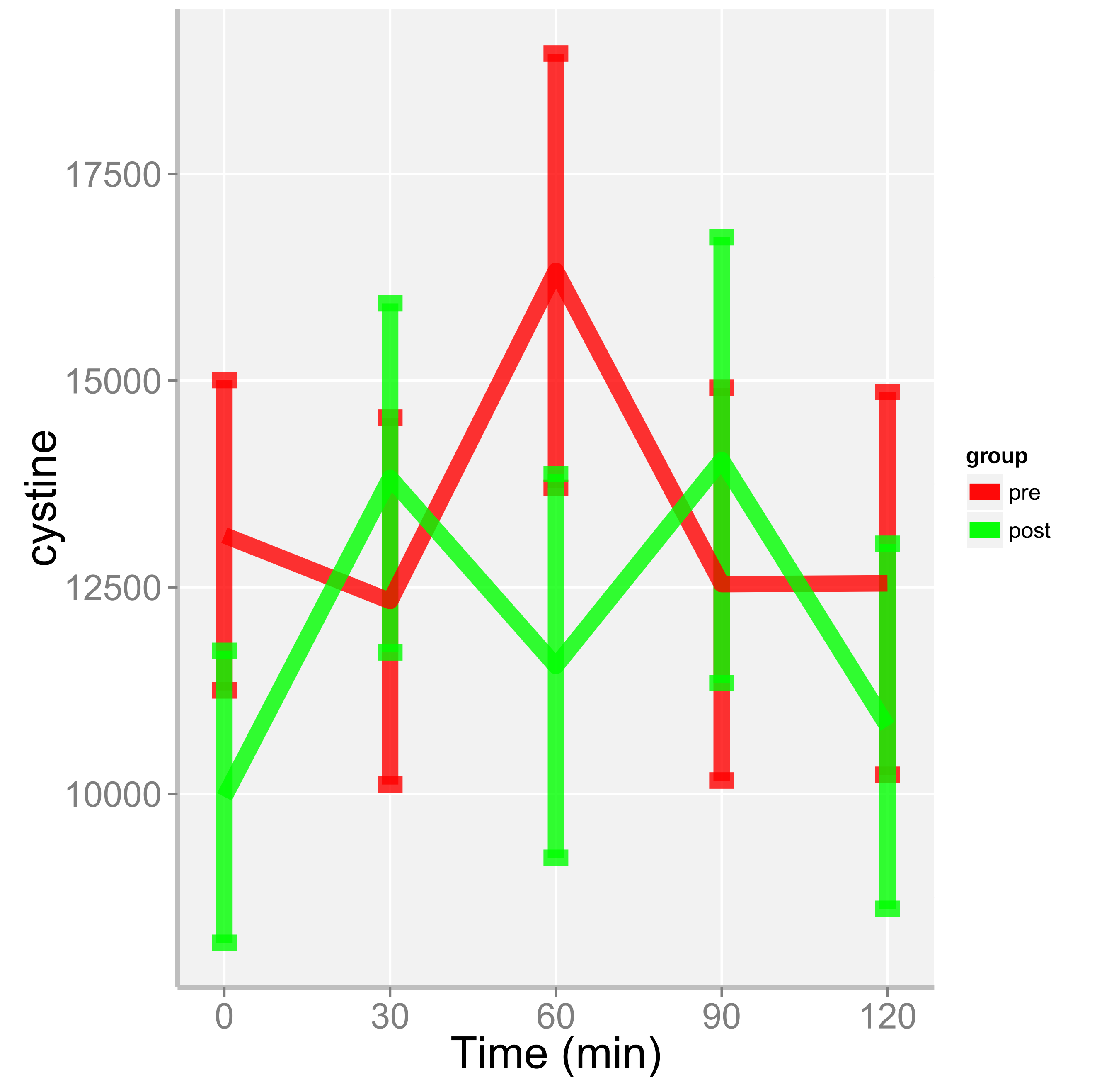

cystine
17500
15000
12500
10000
0
30
60
90
120
Time (min)
group
pre
post

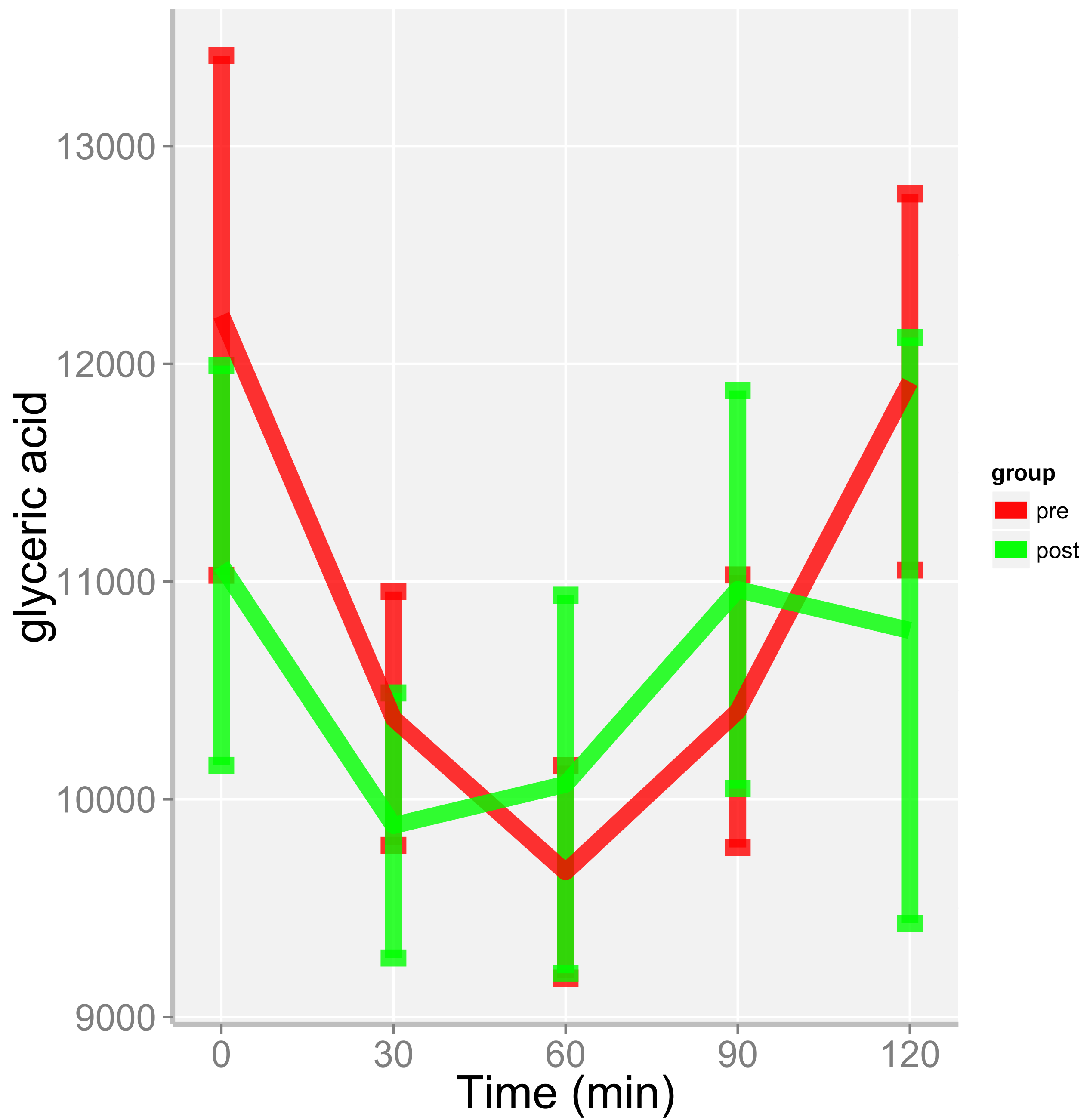

glyceric acid
13000
12000
11000
10000
9000
0
30
60
90
120
Time (min)
group
pre
post

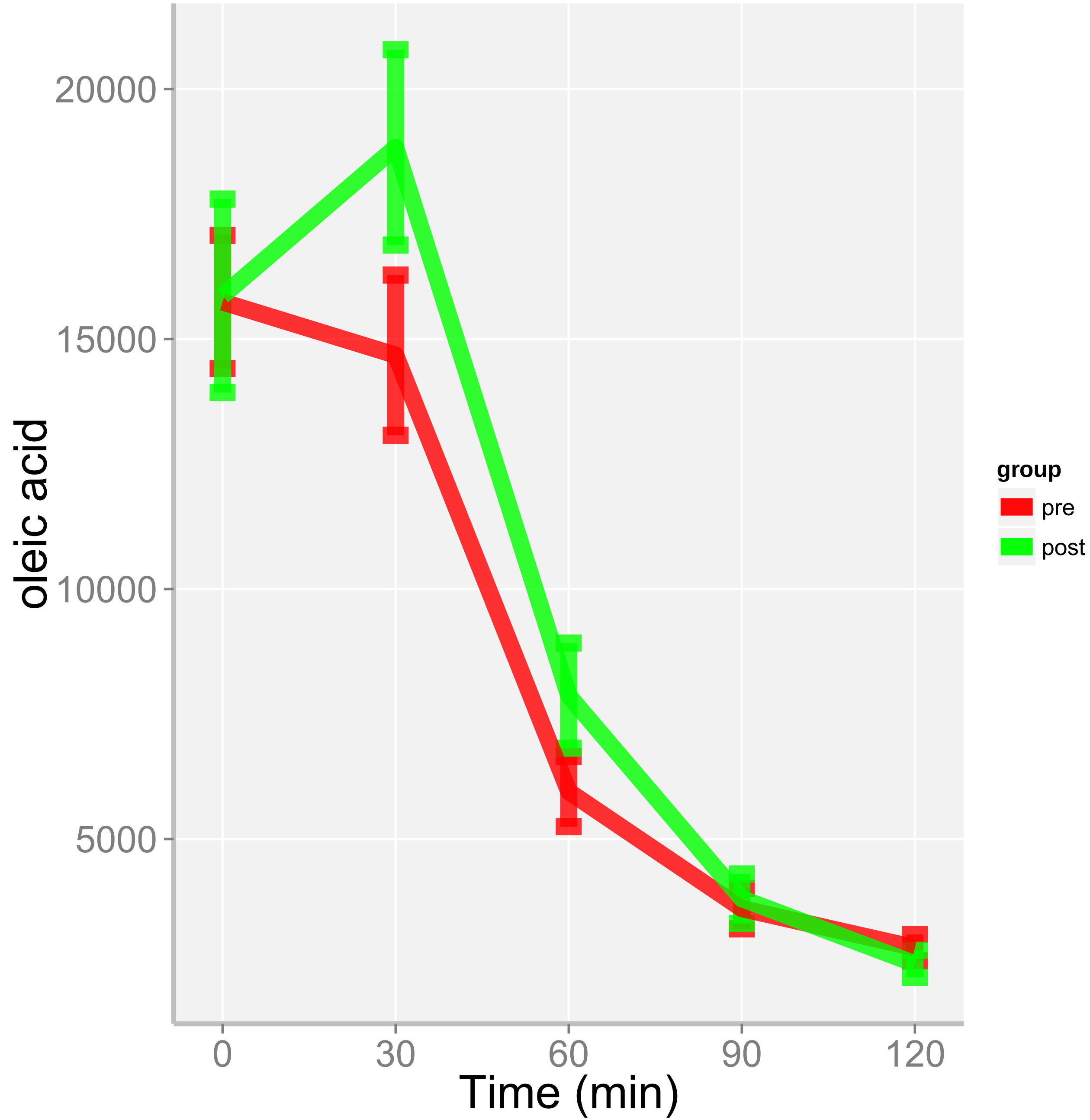
oleic acid
20000
15000
10000
5000
0
30
60
90
120
Time (min)
group
pre
post

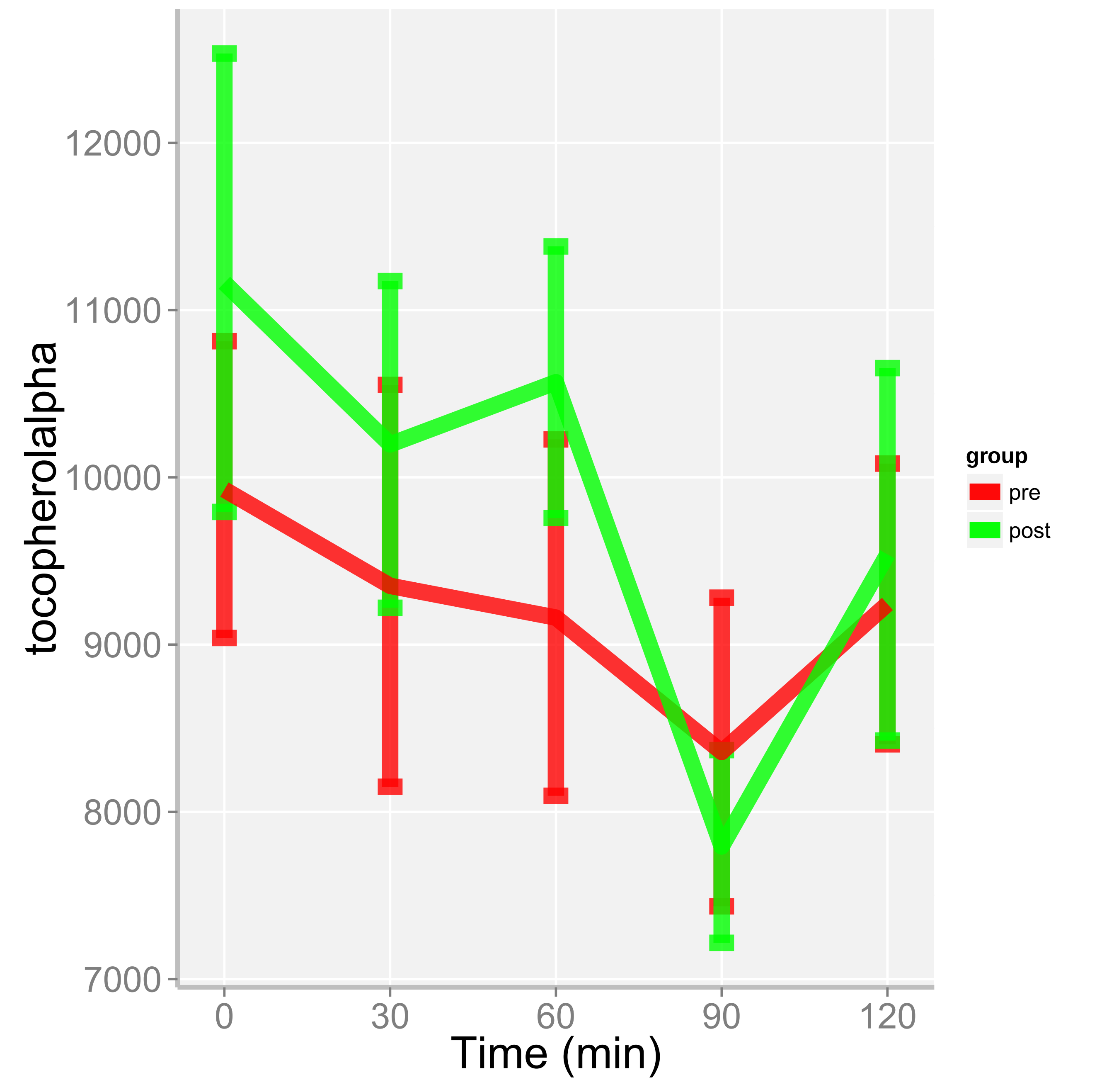

tocopherolalpha
12000
11000
10000
9000
8000
7000
0
30
60
90
120
Time (min)
group
pre
post

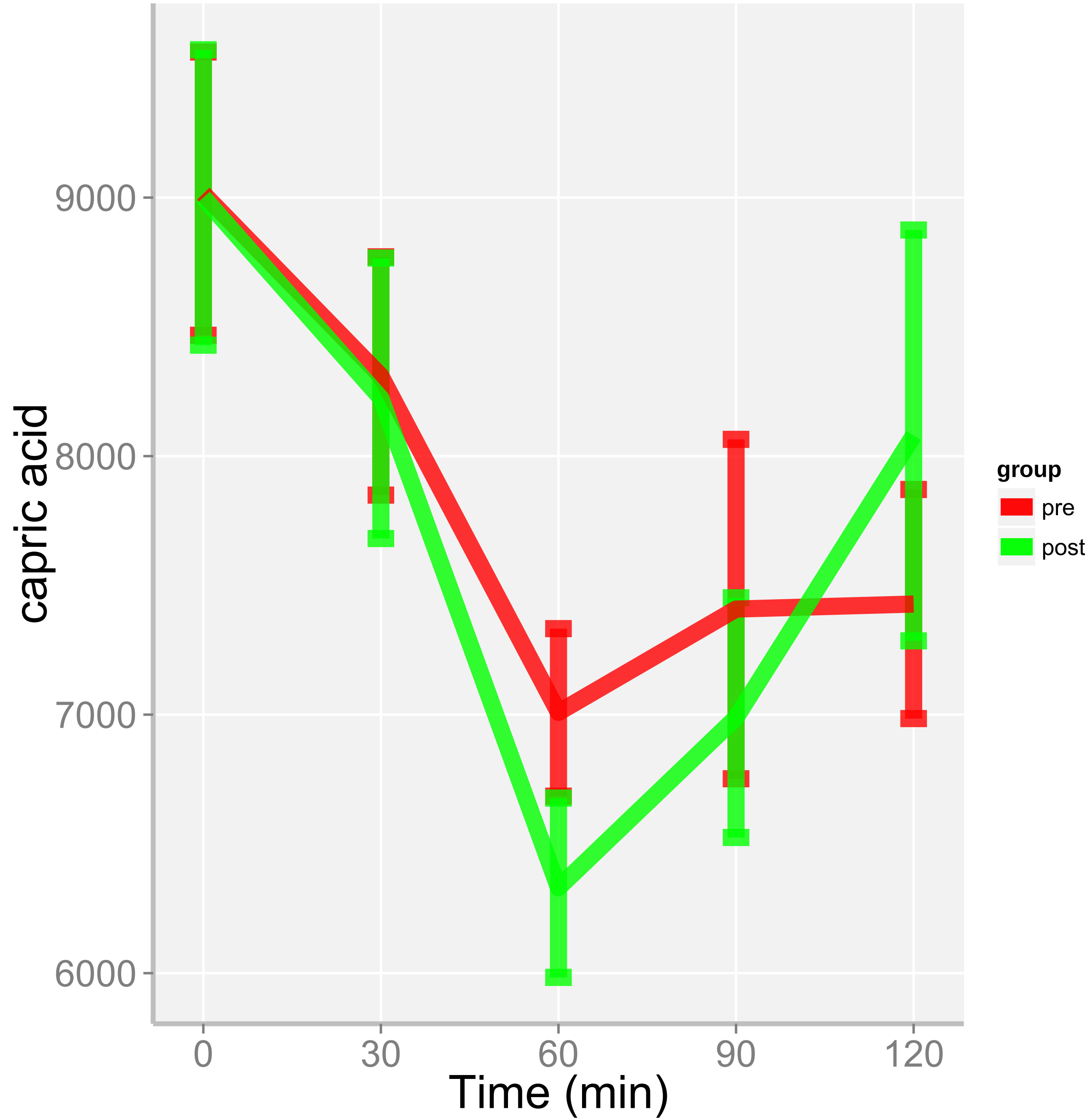
capric acid
9000
8000
7000
6000
0
30
60
90
120
Time (min)
group
pre
post

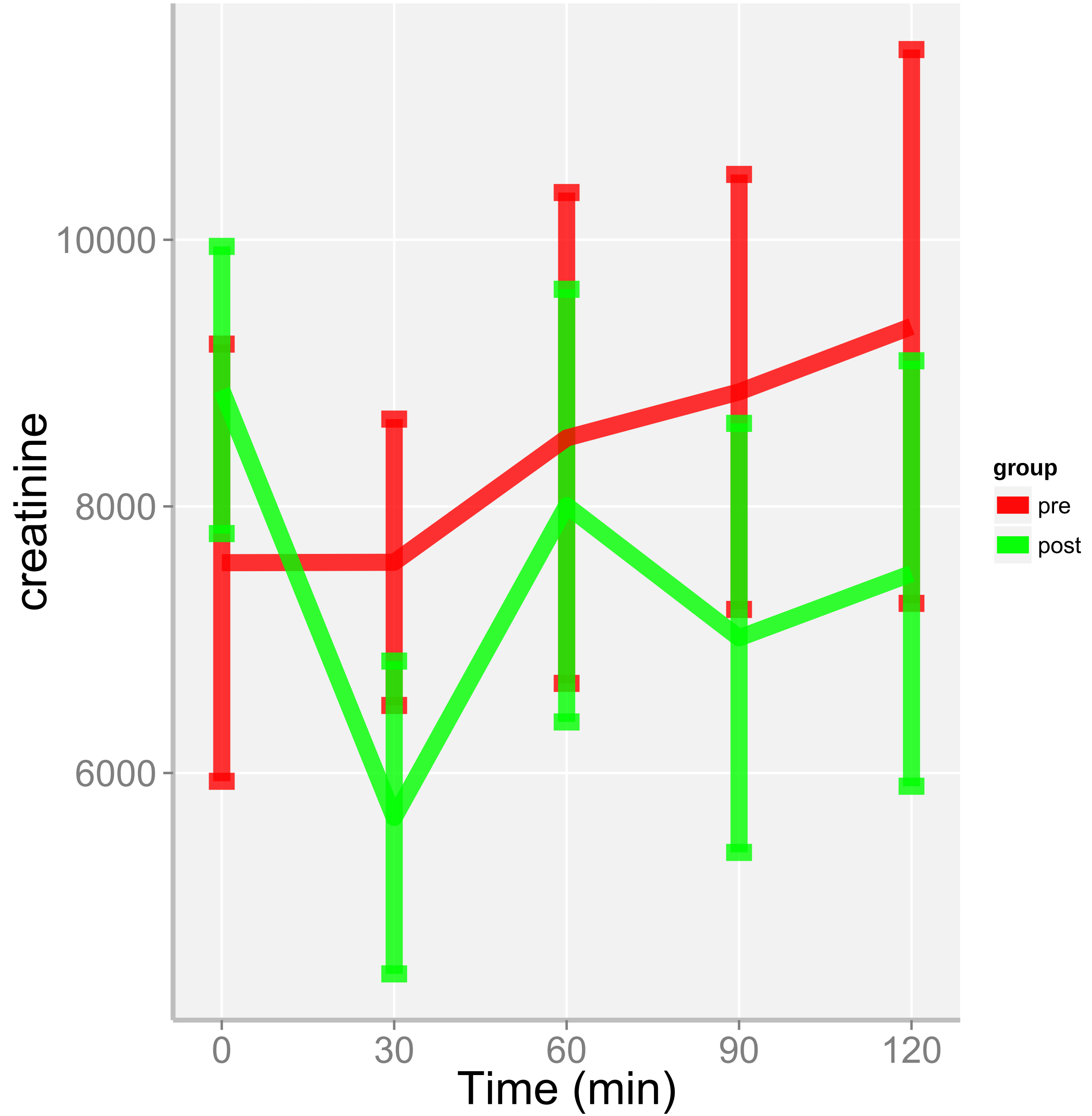

10000
8000
6000
creatinine
0
30
60
90
120
Time (min)
group
pre
post

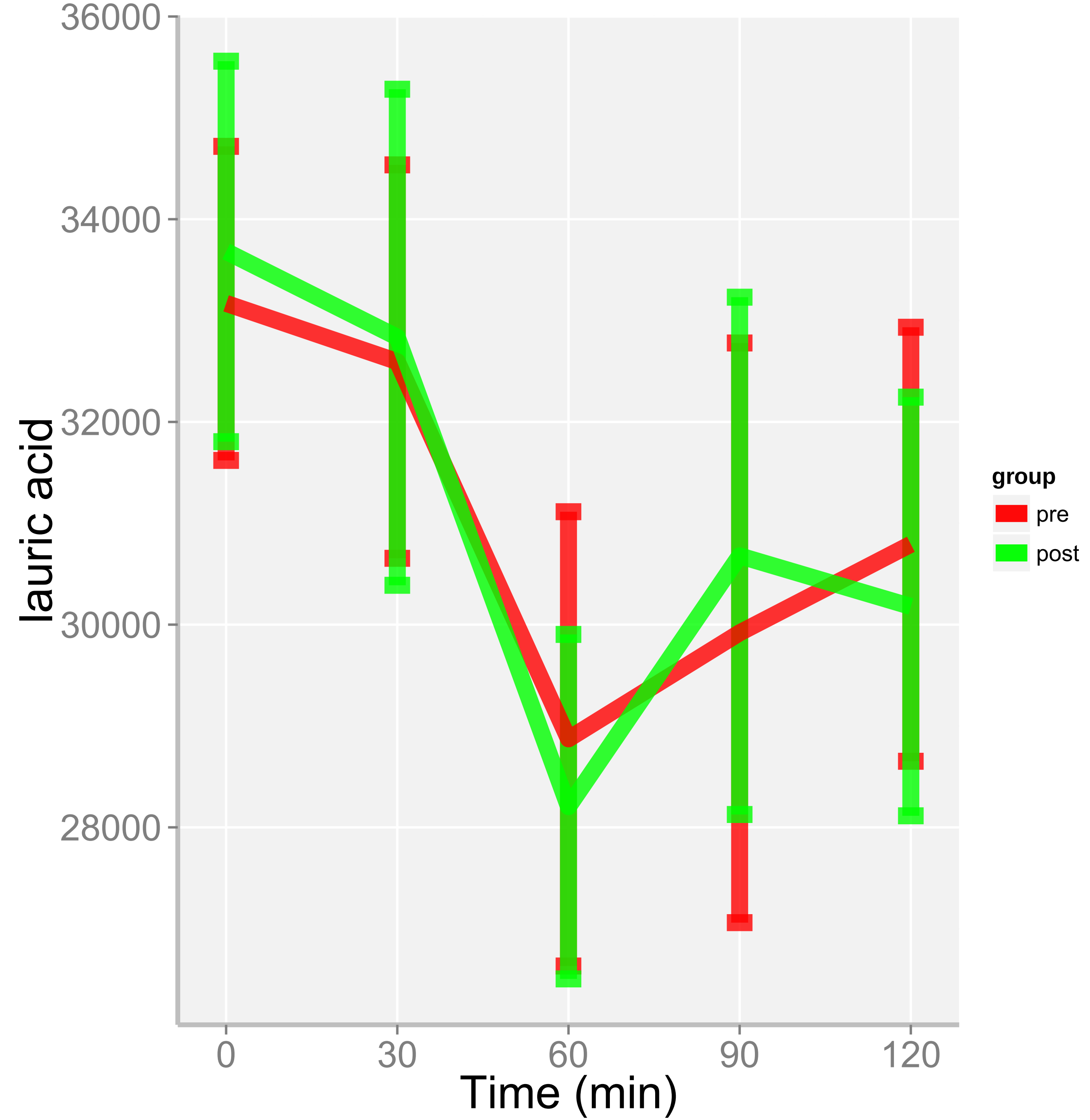

36000
34000
32000
30000
28000
lauric acid
0
30
60
90
120
Time (min)
group
pre
post

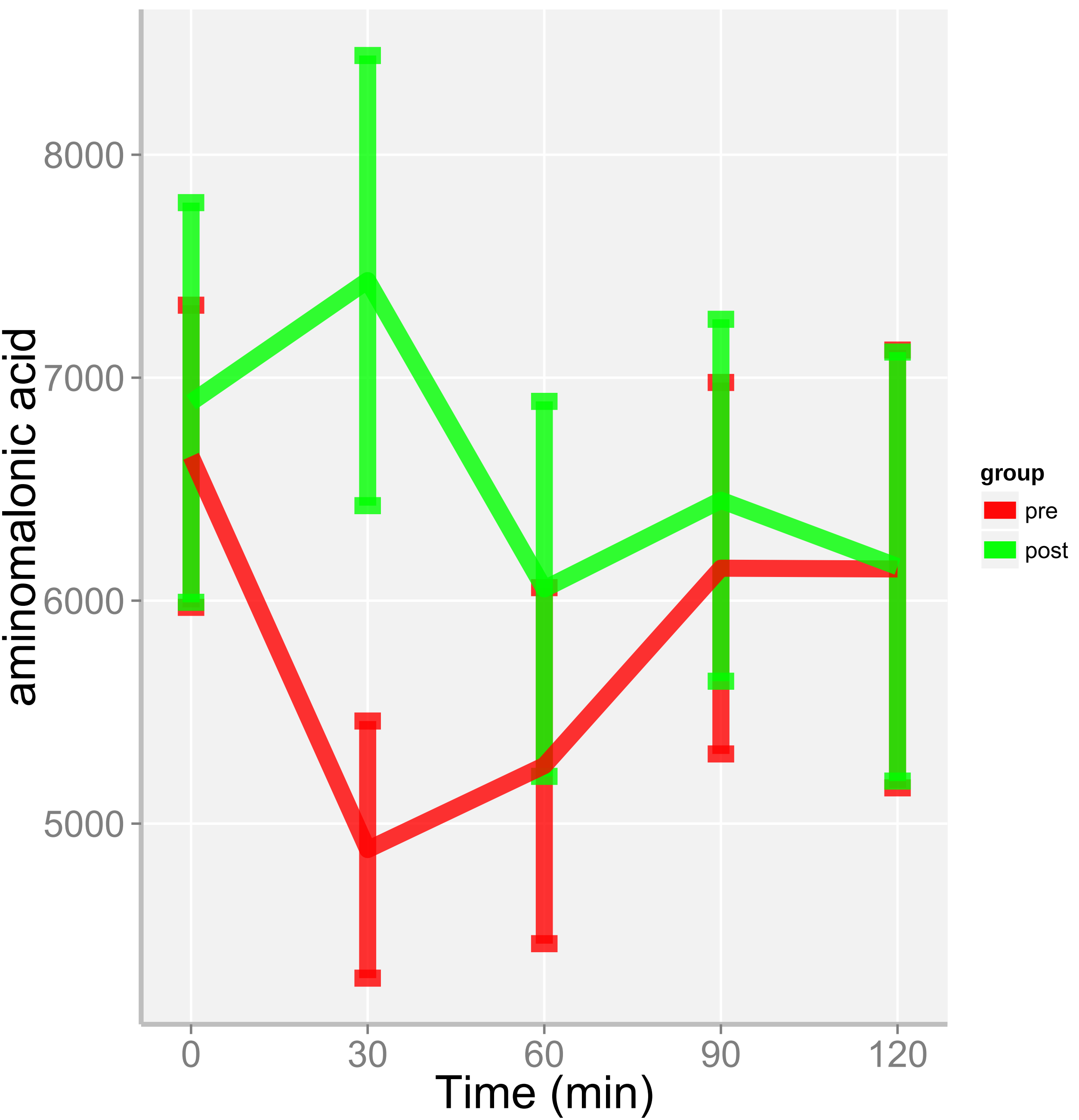

8000
7000
6000
5000
aminomalonic acid
0
30
60
90
120
Time (min)
group
pre
post

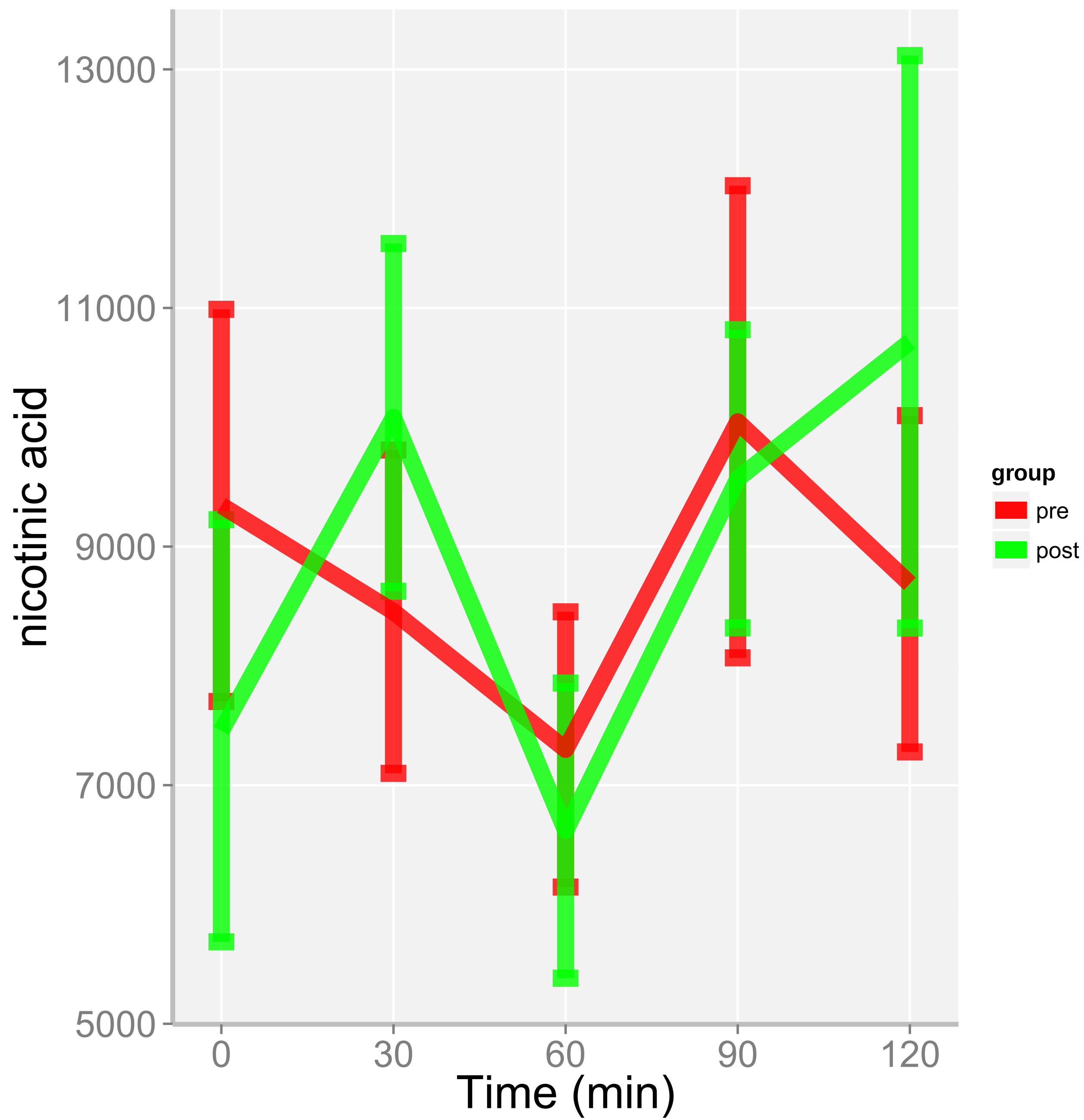

nicotinic acid
13000
11000
9000
7000
5000
0
30
60
90
120
Time (min)
group
pre
post

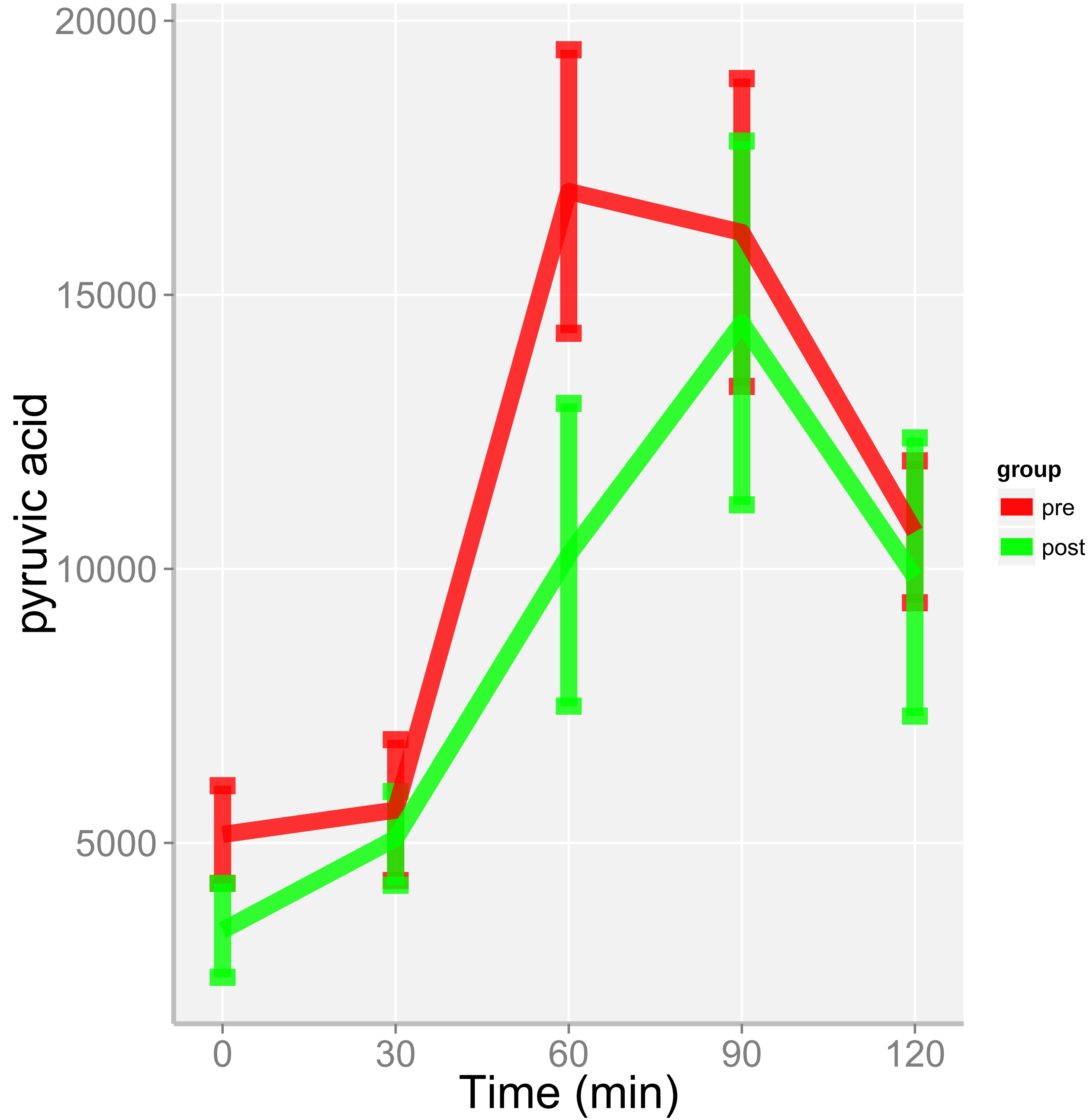

20000
15000
10000
5000
pyruvic acid
0
30
60
90
120
Time (min)
group
pre
post

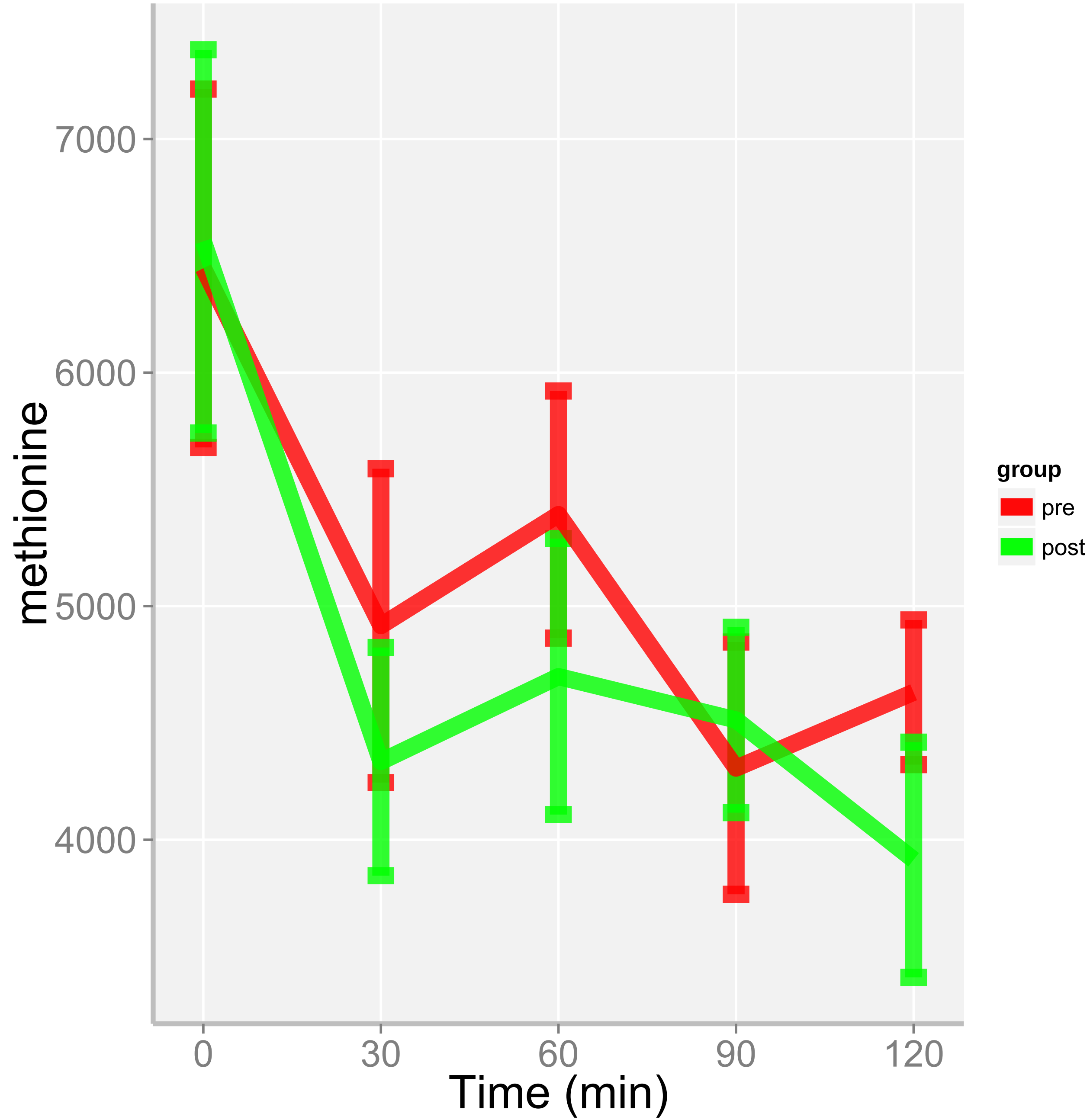

7000
6000
5000
4000
methionine
0
30
60
90
120
Time (min)
group
pre
post

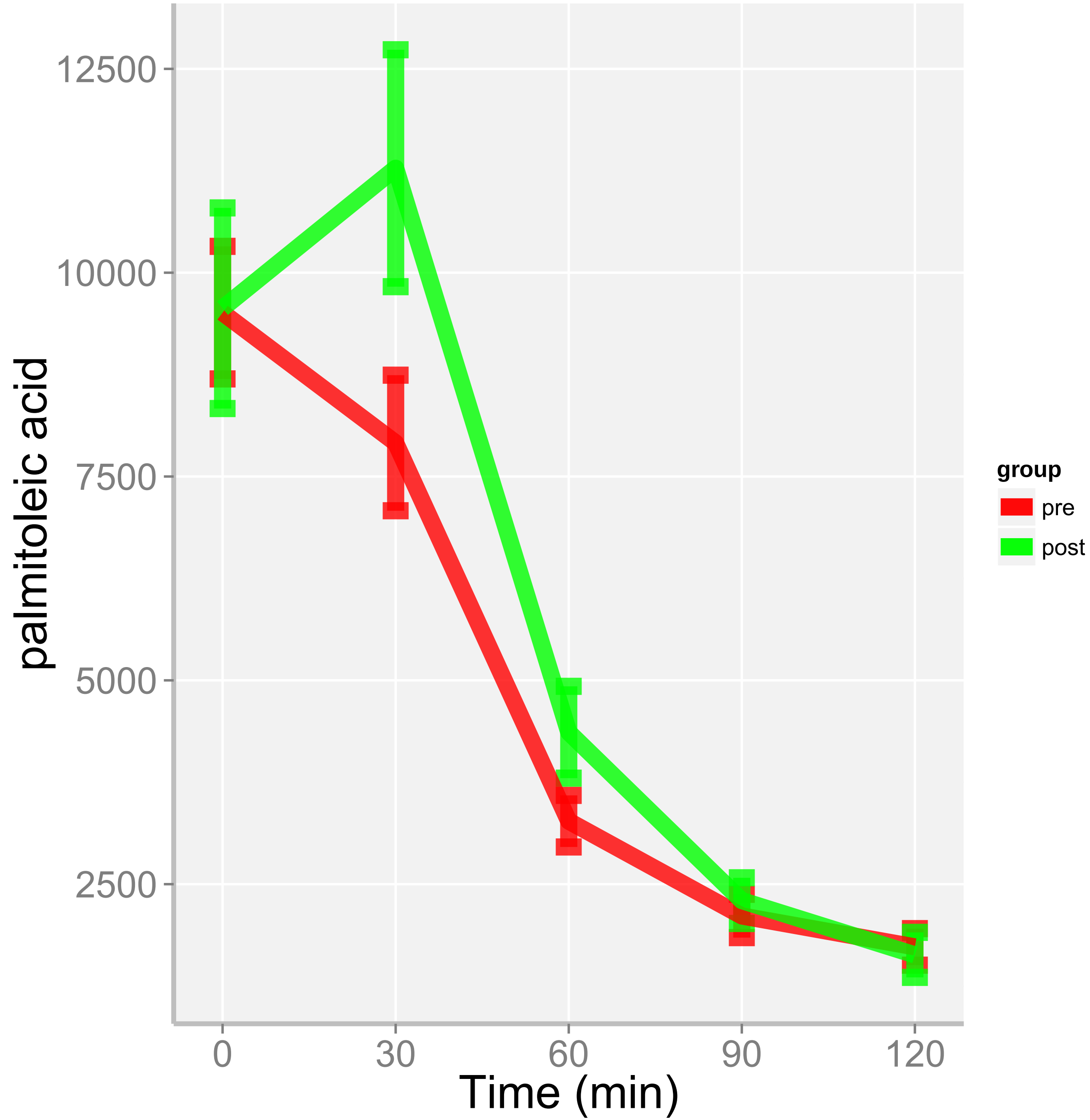

palmitoleic acid
12500
10000
7500
5000
2500
0
30
60
90
120
Time (min)
group
pre
post

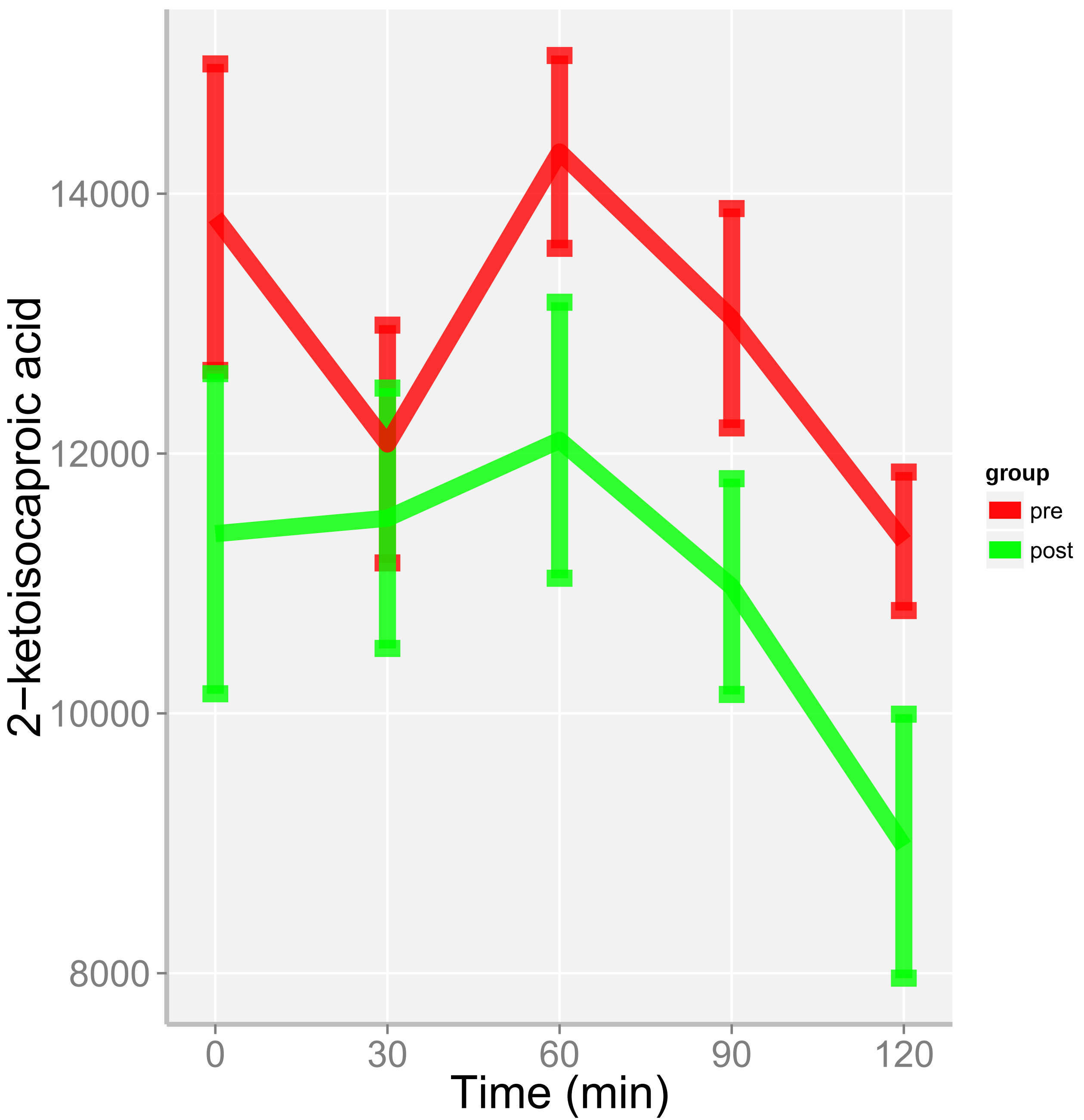

2−ketoisocaproic acid
14000
12000
10000
8000
0
30
60
90
120
Time (min)
group
pre
post

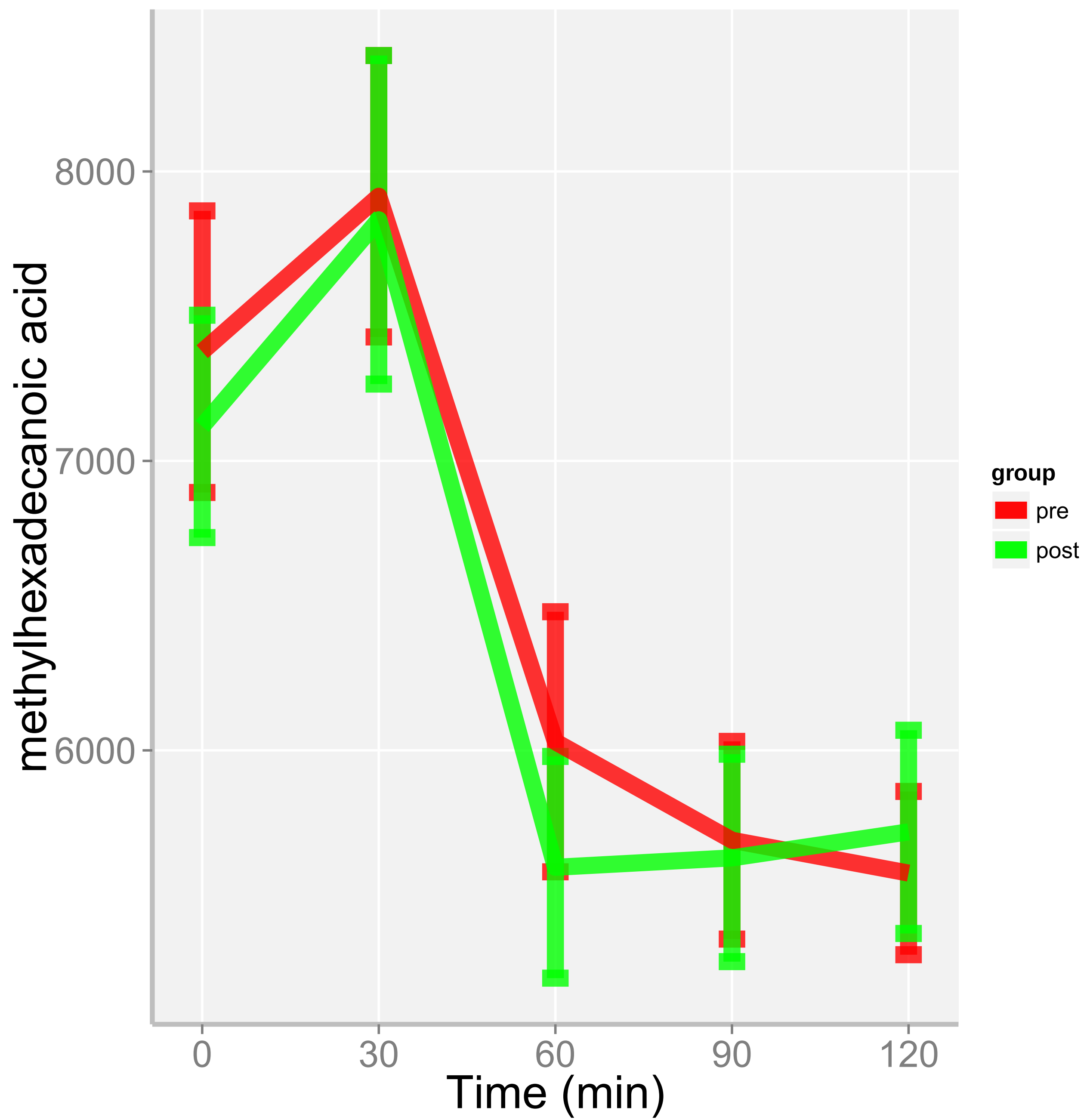

methylhexadecanoic acid
8000
7000
6000
0
30
60
90
120
Time (min)
group
pre
post

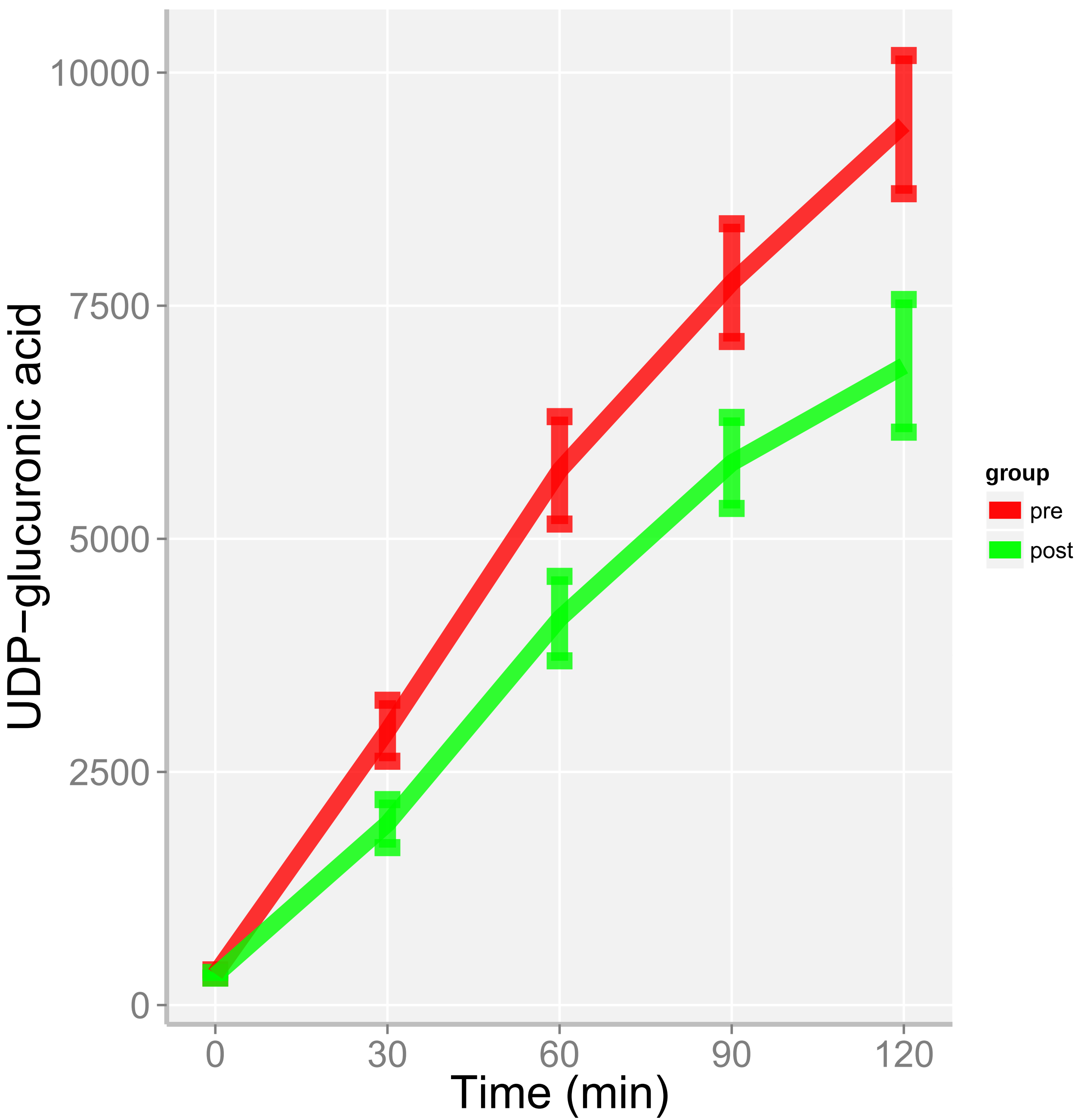

UDP−glucuronic acid
10000
7500
5000
2500
0
0
30
60
90
120
Time (min)
group
pre
post

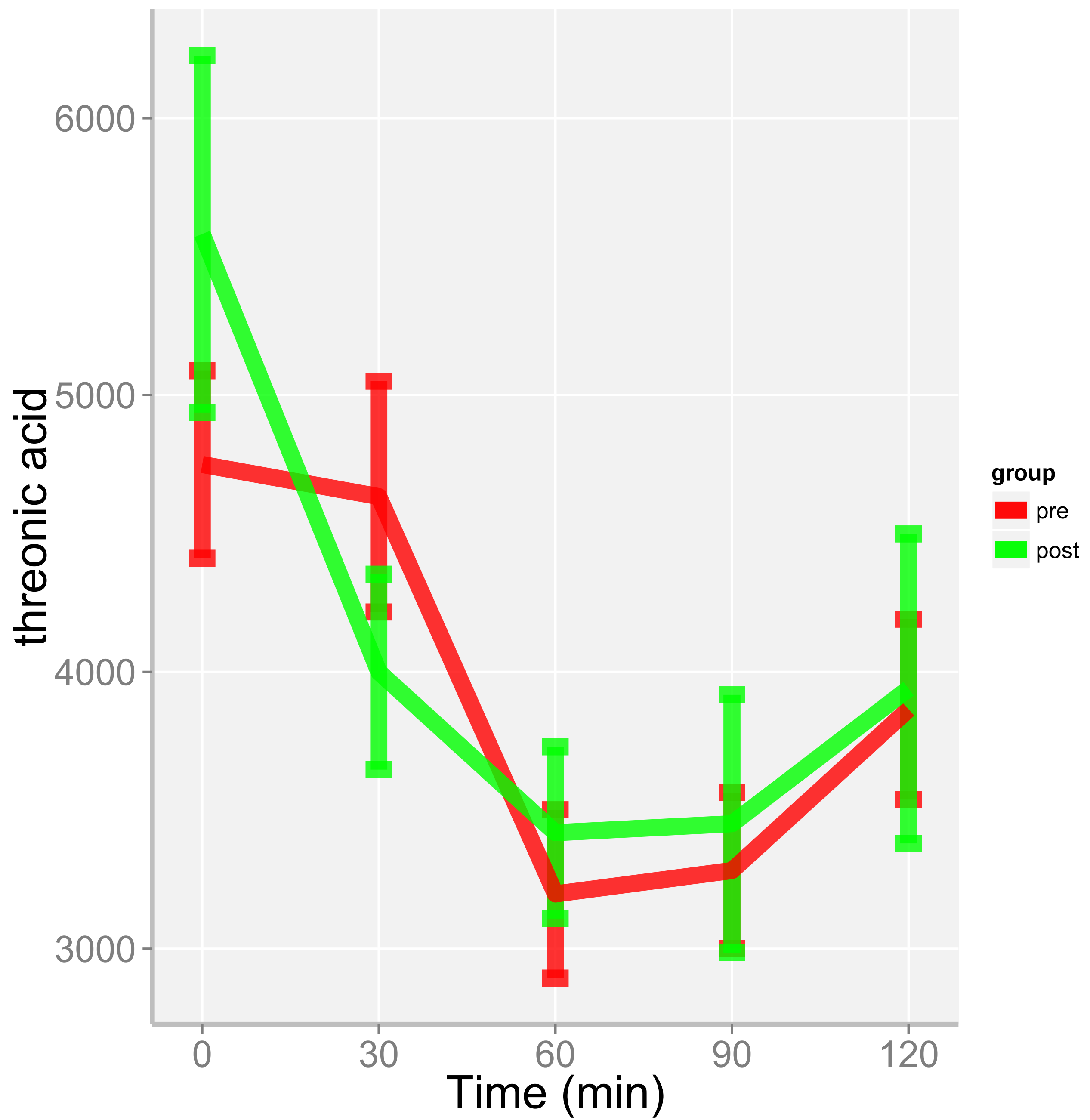

threonic acid
6000
5000
4000
3000
0
30
60
90
120
Time (min)
group
pre
post

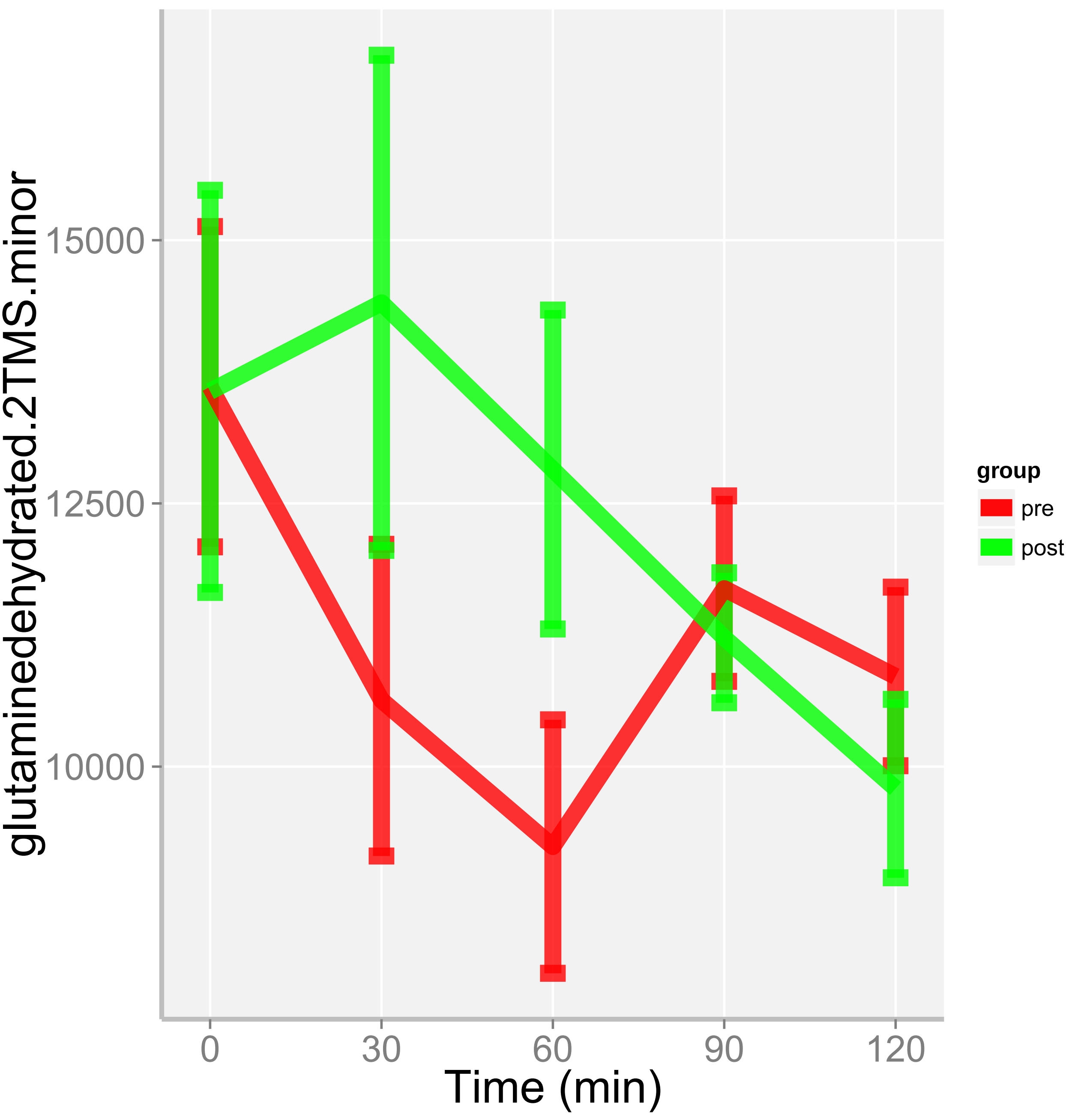

glutaminedehydrated.2TMS.minor
15000
12500
10000
0
30
60
90
120
Time (min)
group
pre
post

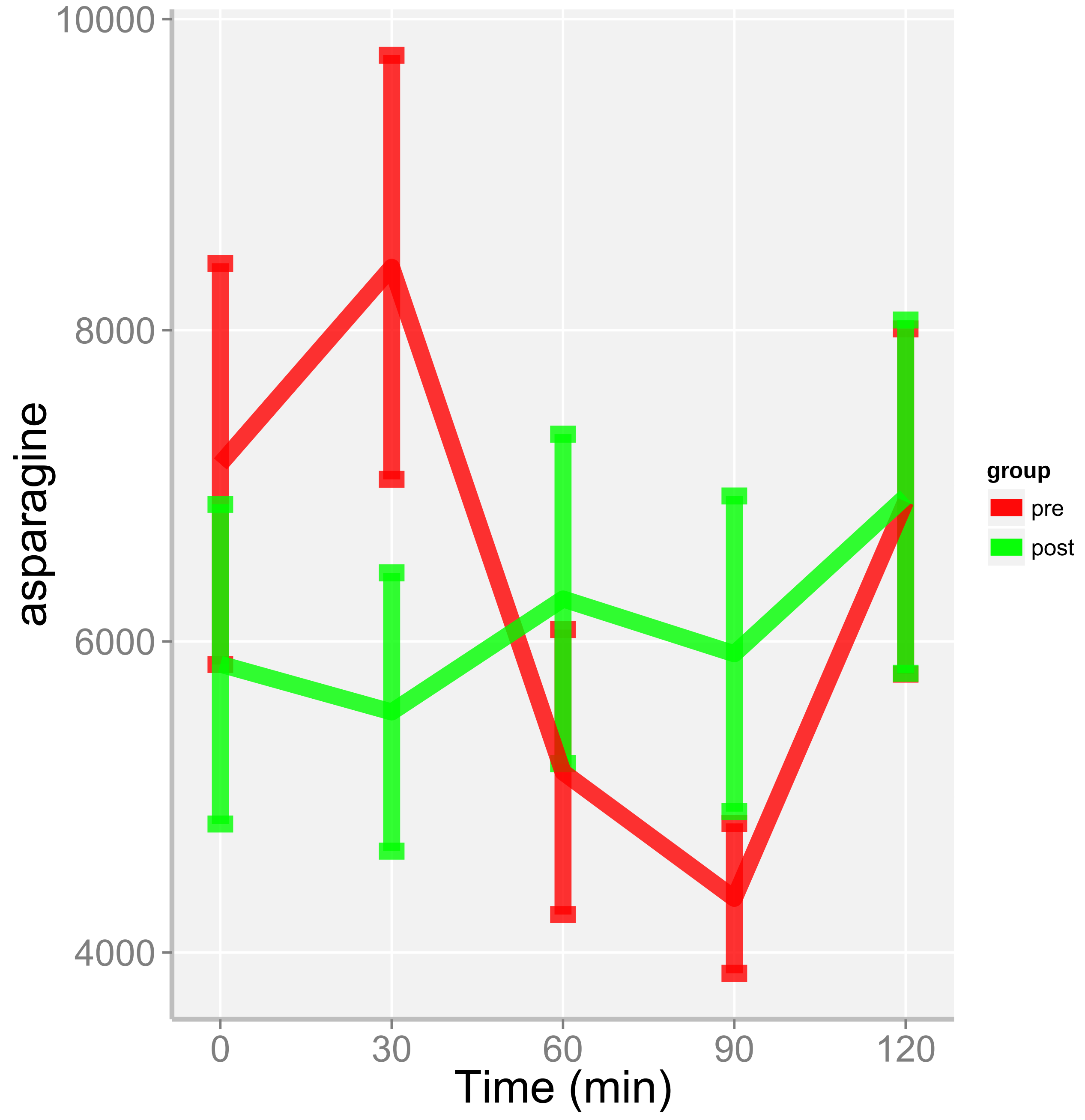

10000
8000
6000
4000
asparagine
0
30
60
90
120
Time (min)
group
pre
post

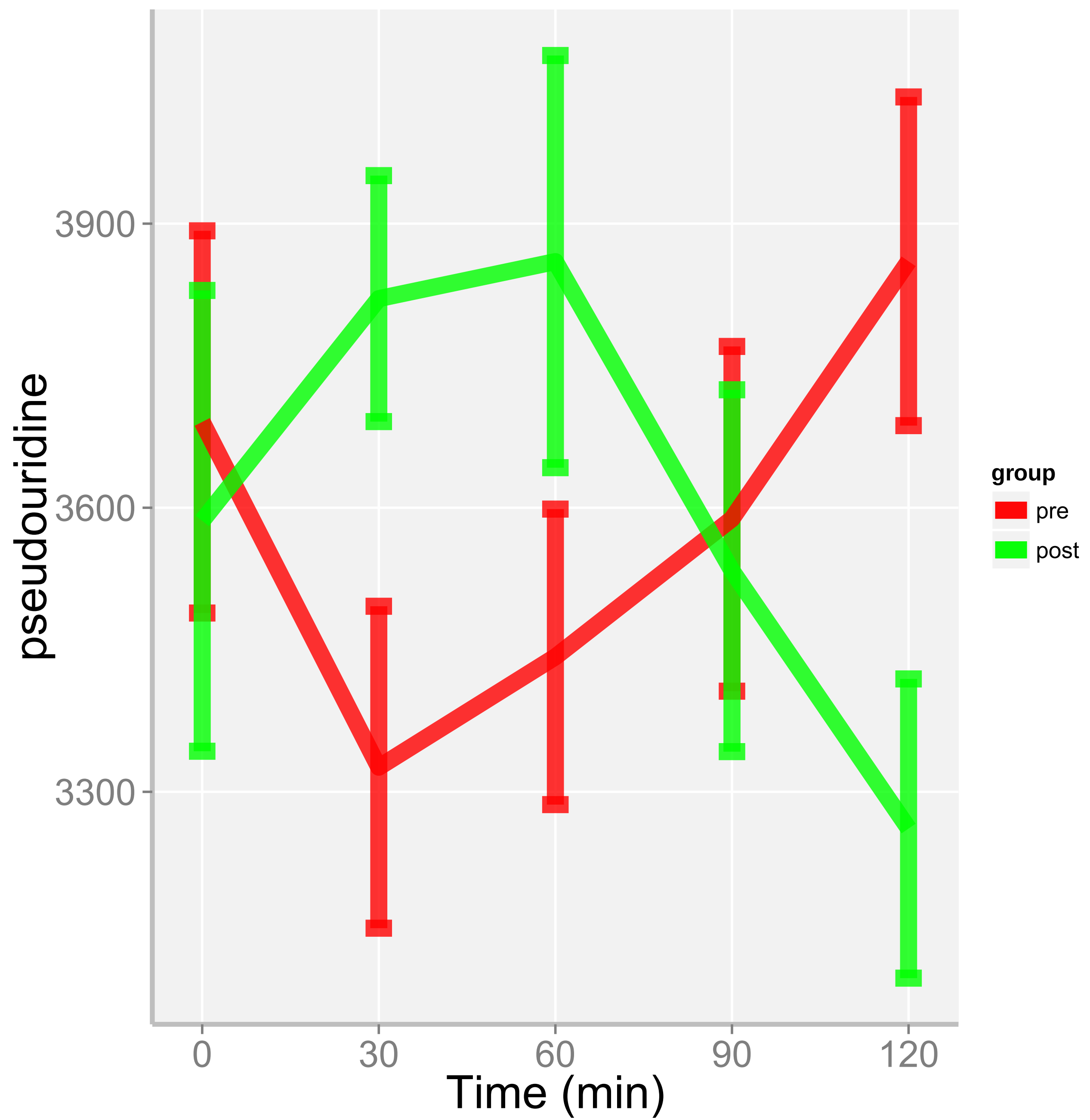

pseudouridine
3900
3600
3300
0
30
60
90
120
Time (min)
group
pre
post

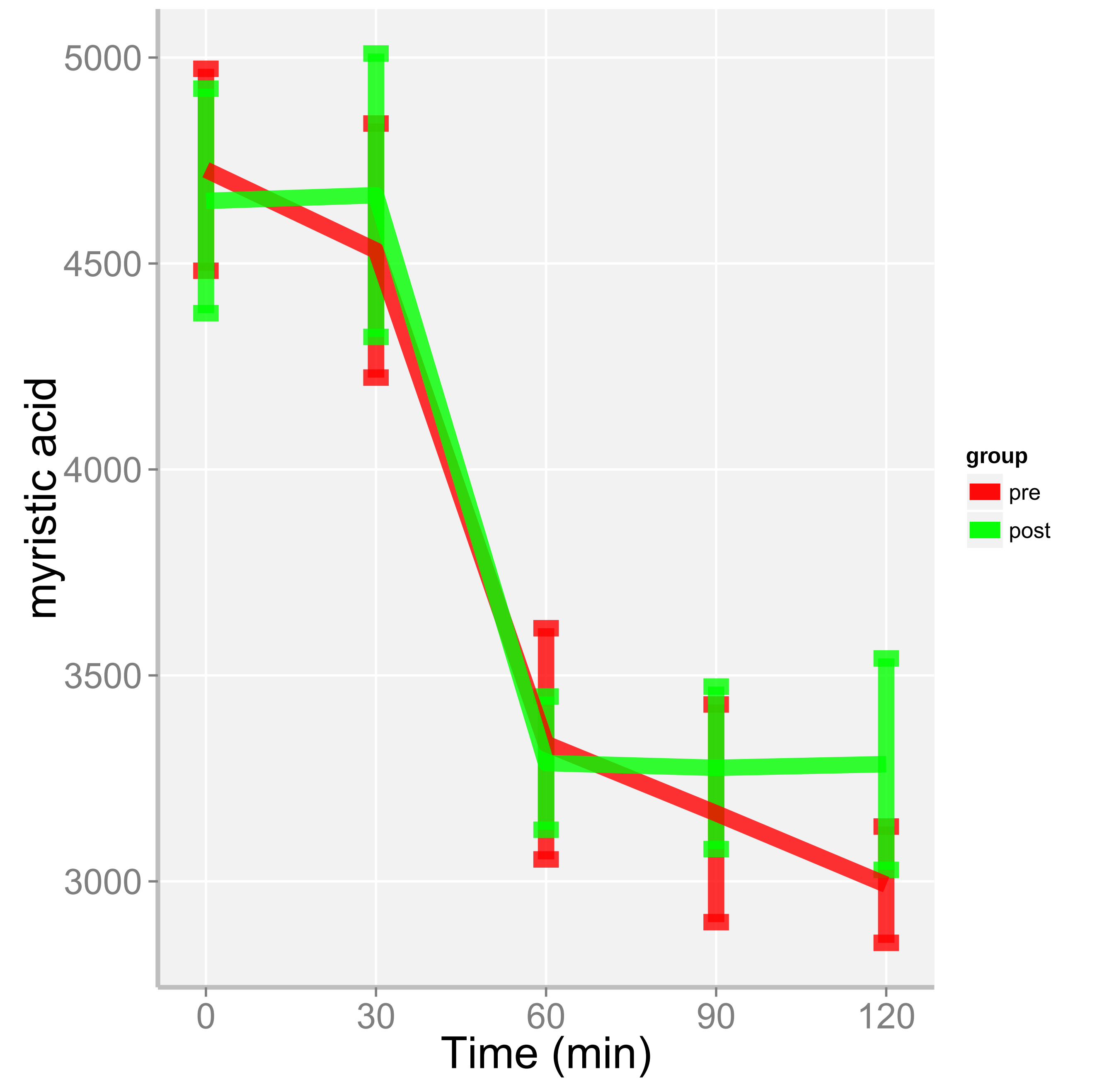

myristic acid
5000
4500
4000
3500
3000
0
30
60
90
120
Time (min)
group
pre
post

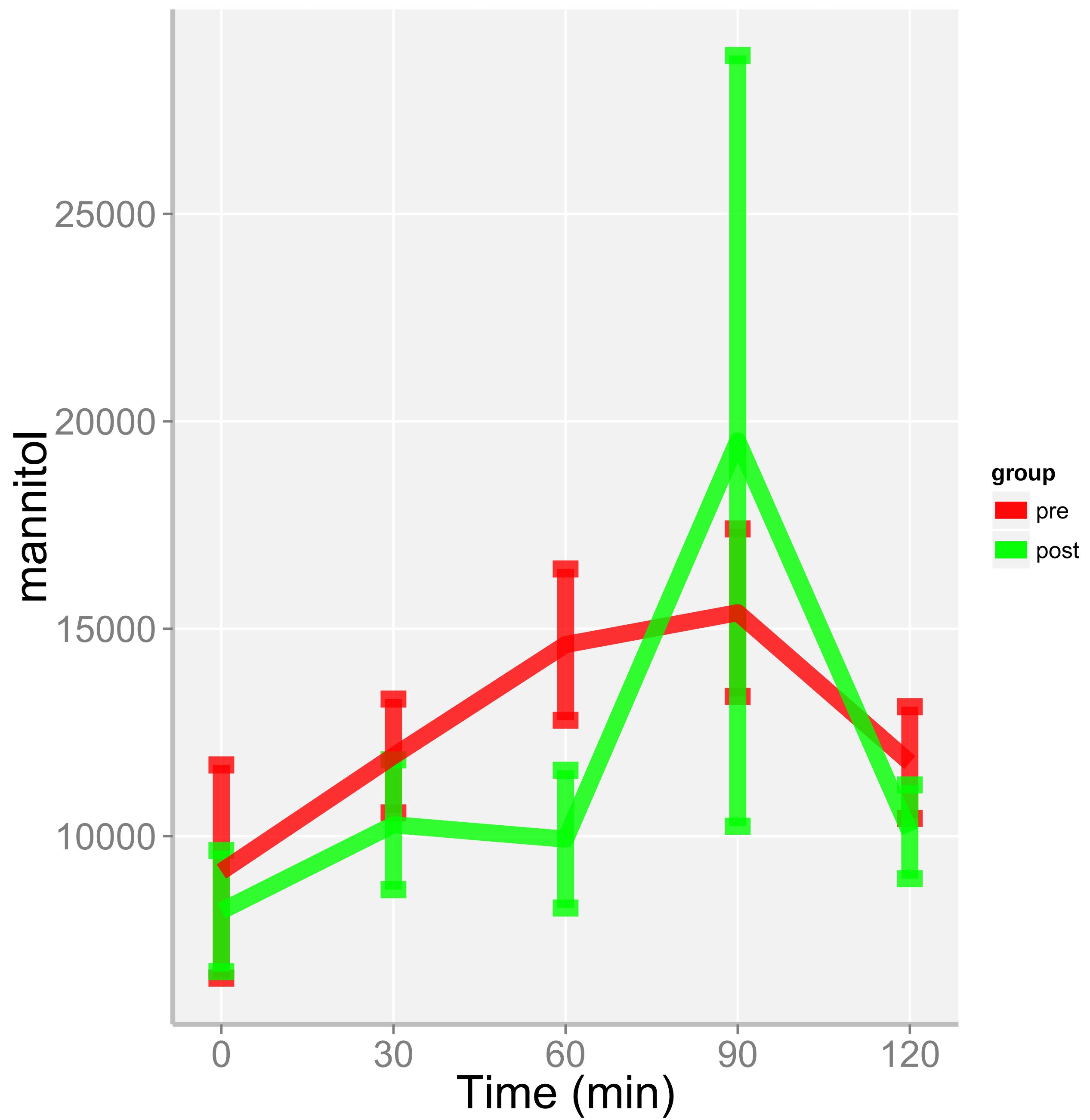

mannitol
25000
20000
15000
10000
0
30
60
90
120
Time (min)
group
pre
post

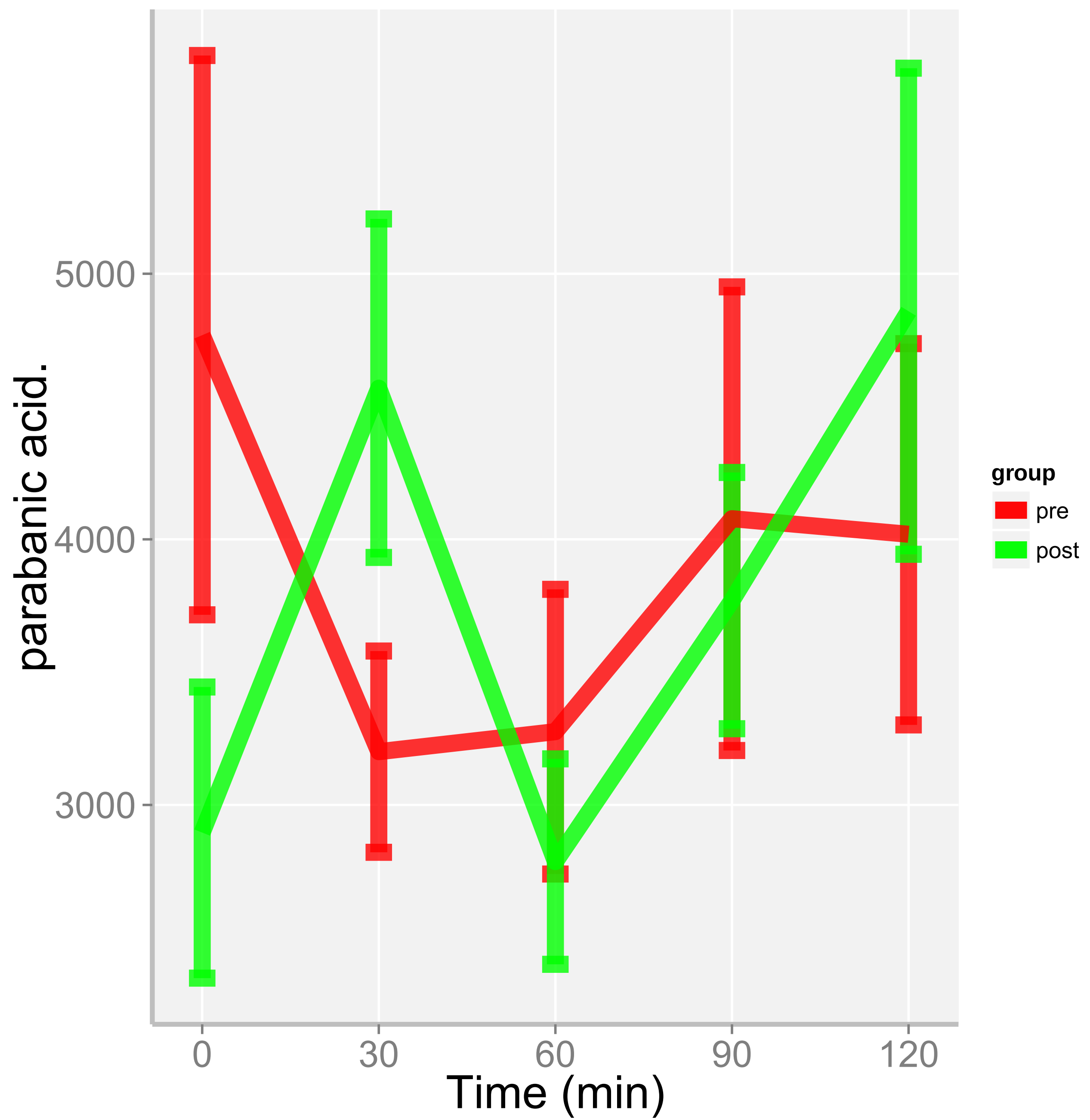

parabanic acid.
5000
4000
3000
0
30
60
90
120
Time (min)
group
pre
post

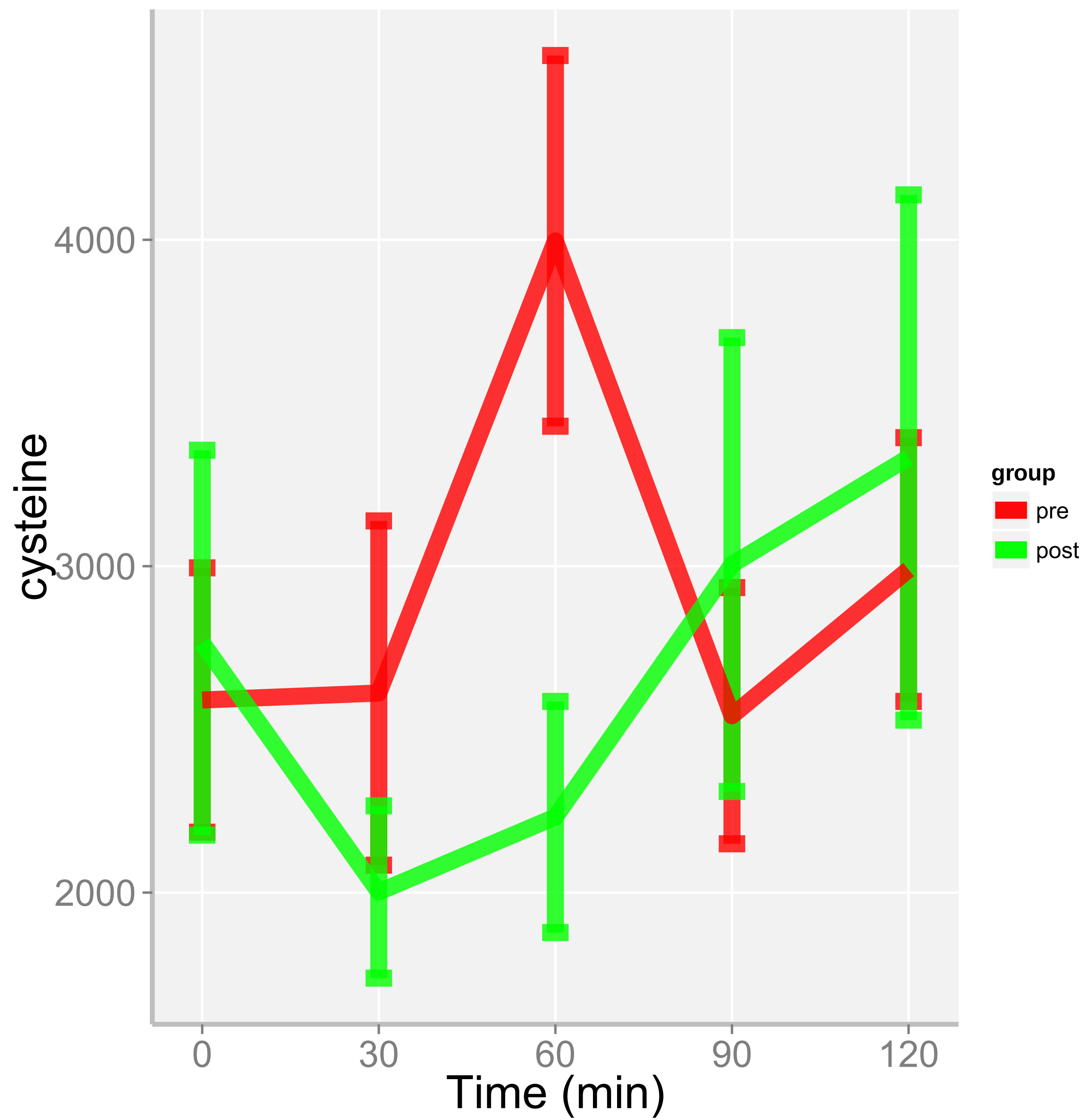

cysteine
4000
3000
2000
0
30
60
90
120
Time (min)
group
pre
post

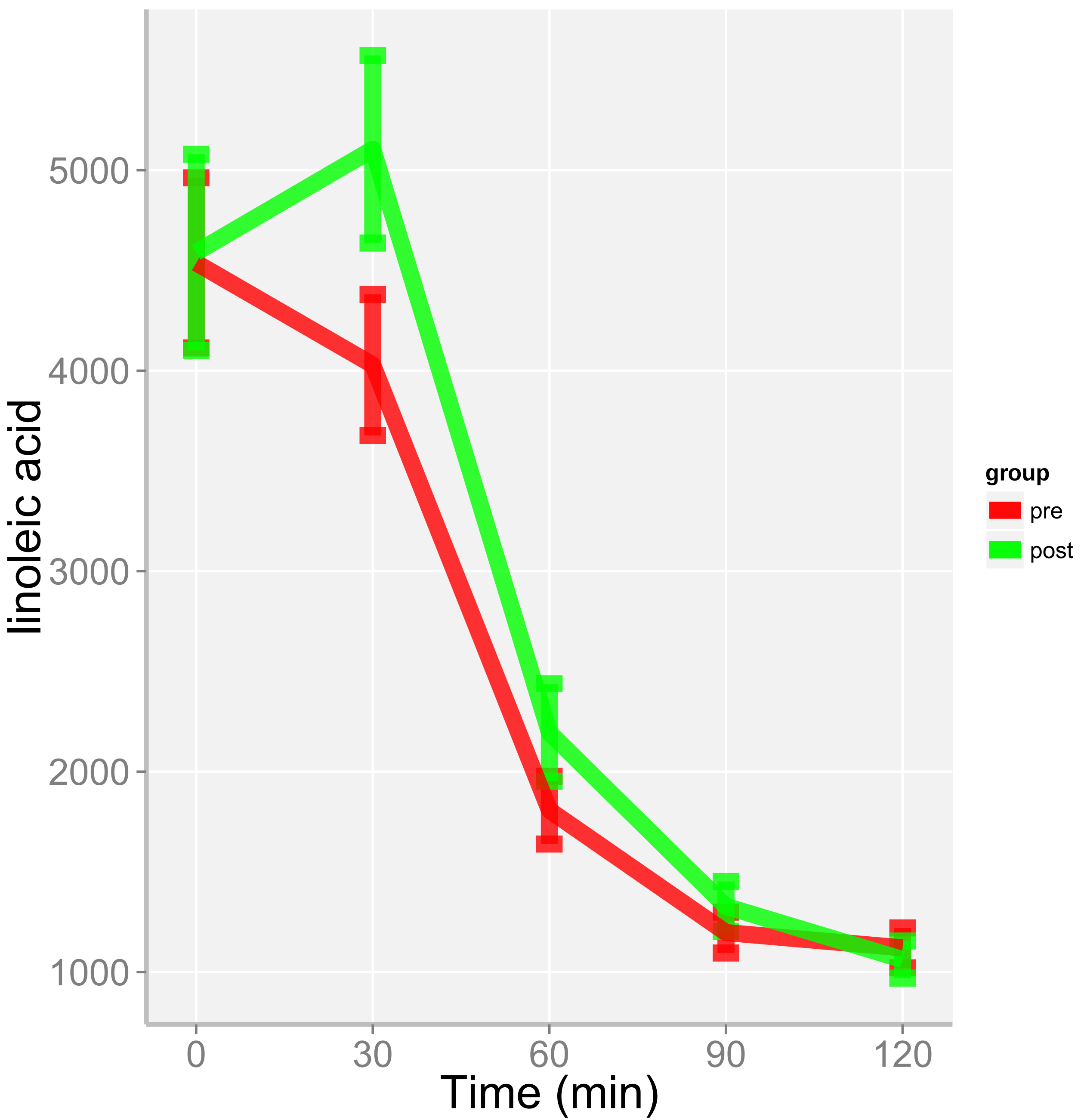

linoleic acid
5000
4000
3000
2000
1000
0
30
60
90
120
Time (min)
group
pre
post

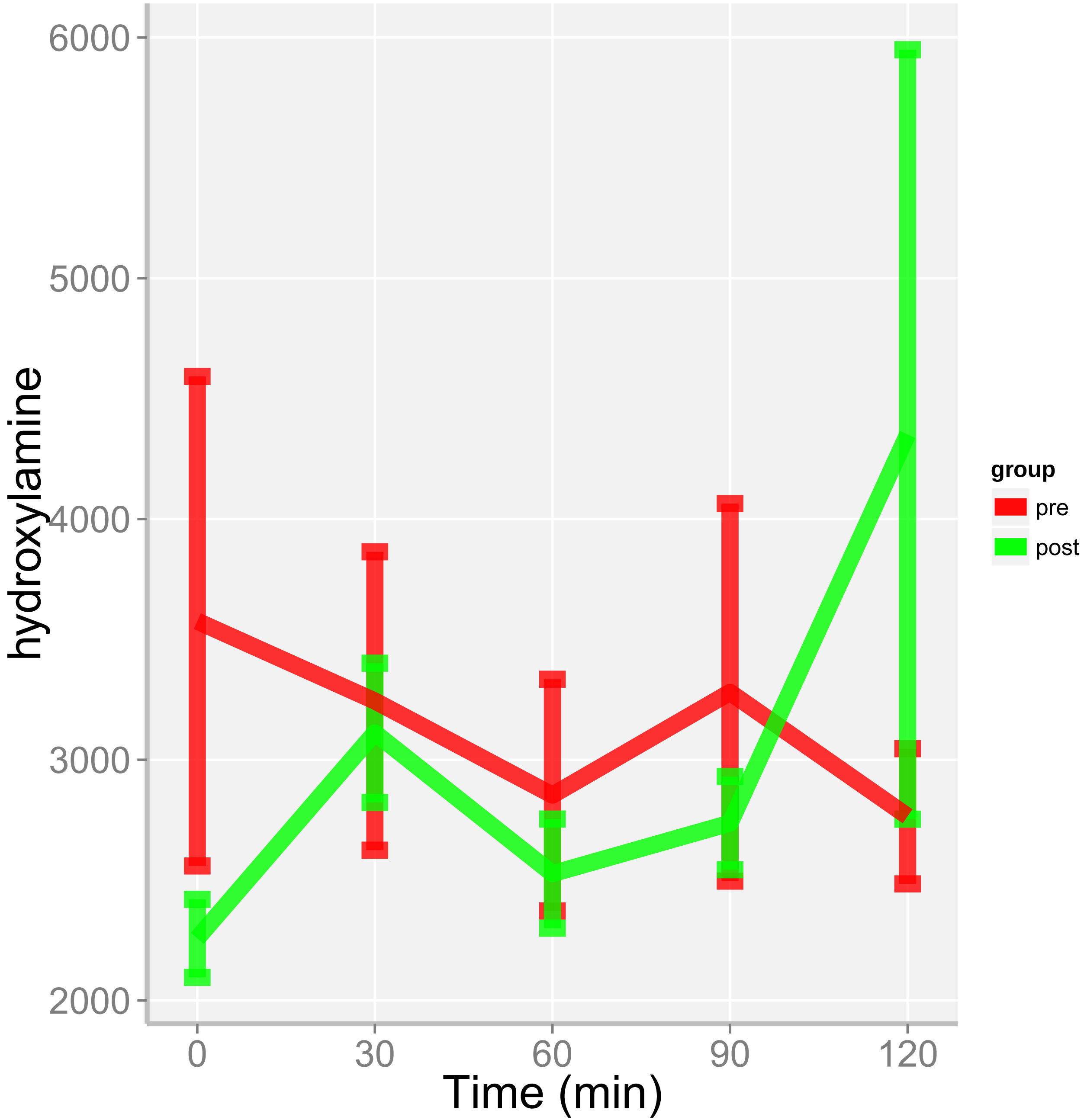

6000
5000
4000
3000
2000
hydroxylamine
0
30
60
90
120
Time (min)
group
pre
post

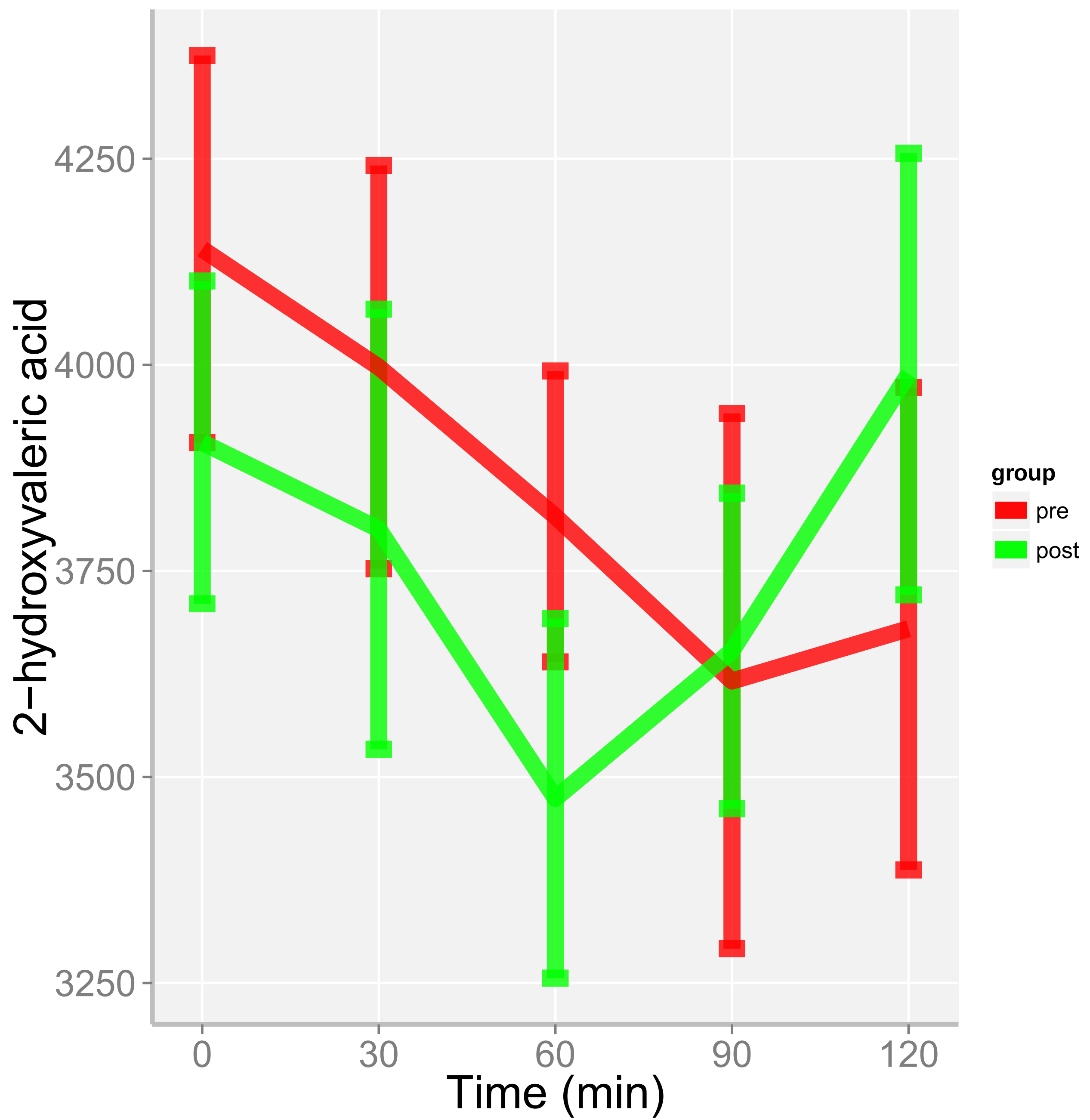

2−hydroxyvaleric acid
4250
4000
3750
3500
3250
0
30
60
90
120
Time (min)
group
pre
post

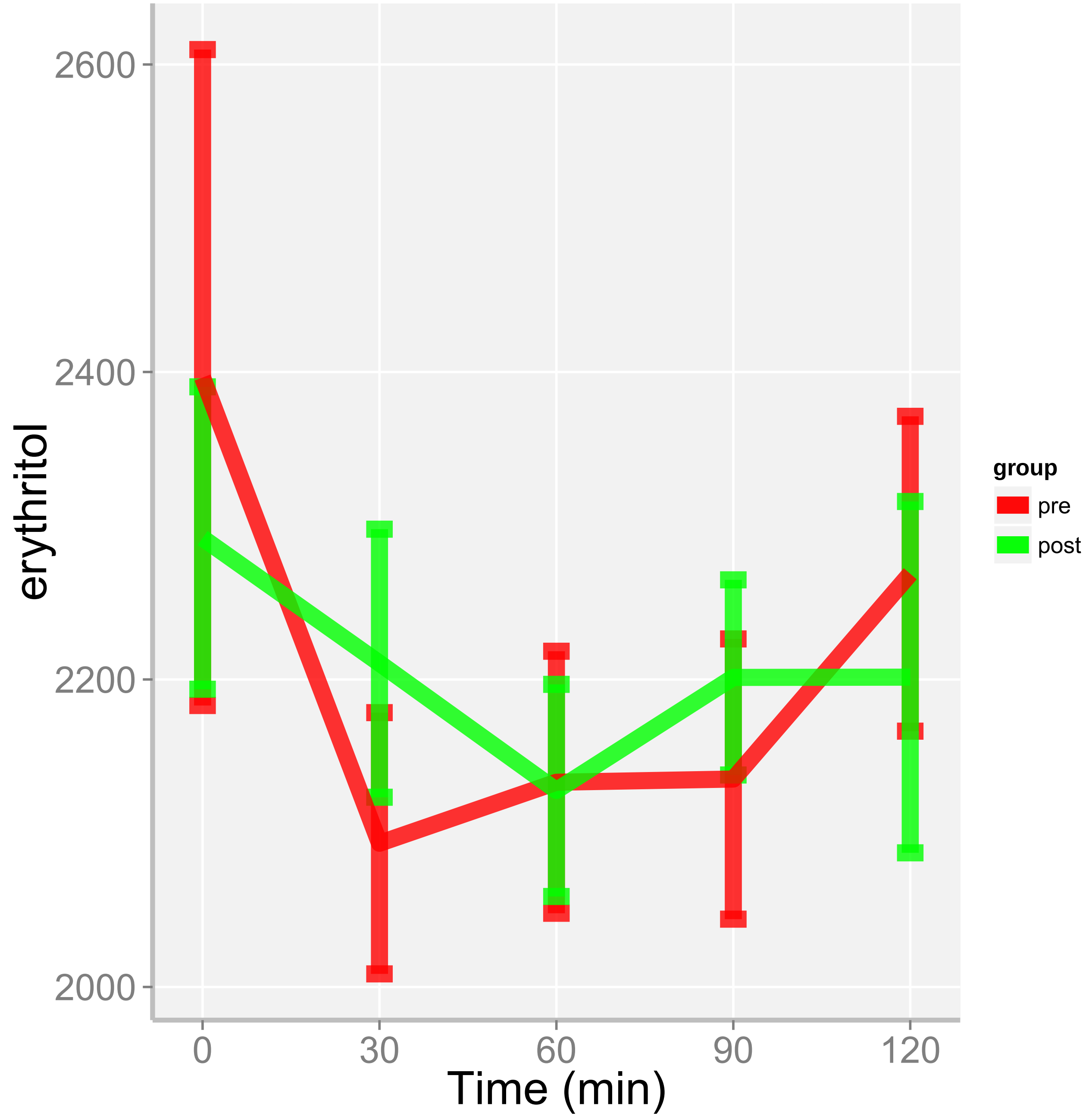

erythritol
2600
2400
2200
2000
0
30
60
90
120
Time (min)
group
pre
post

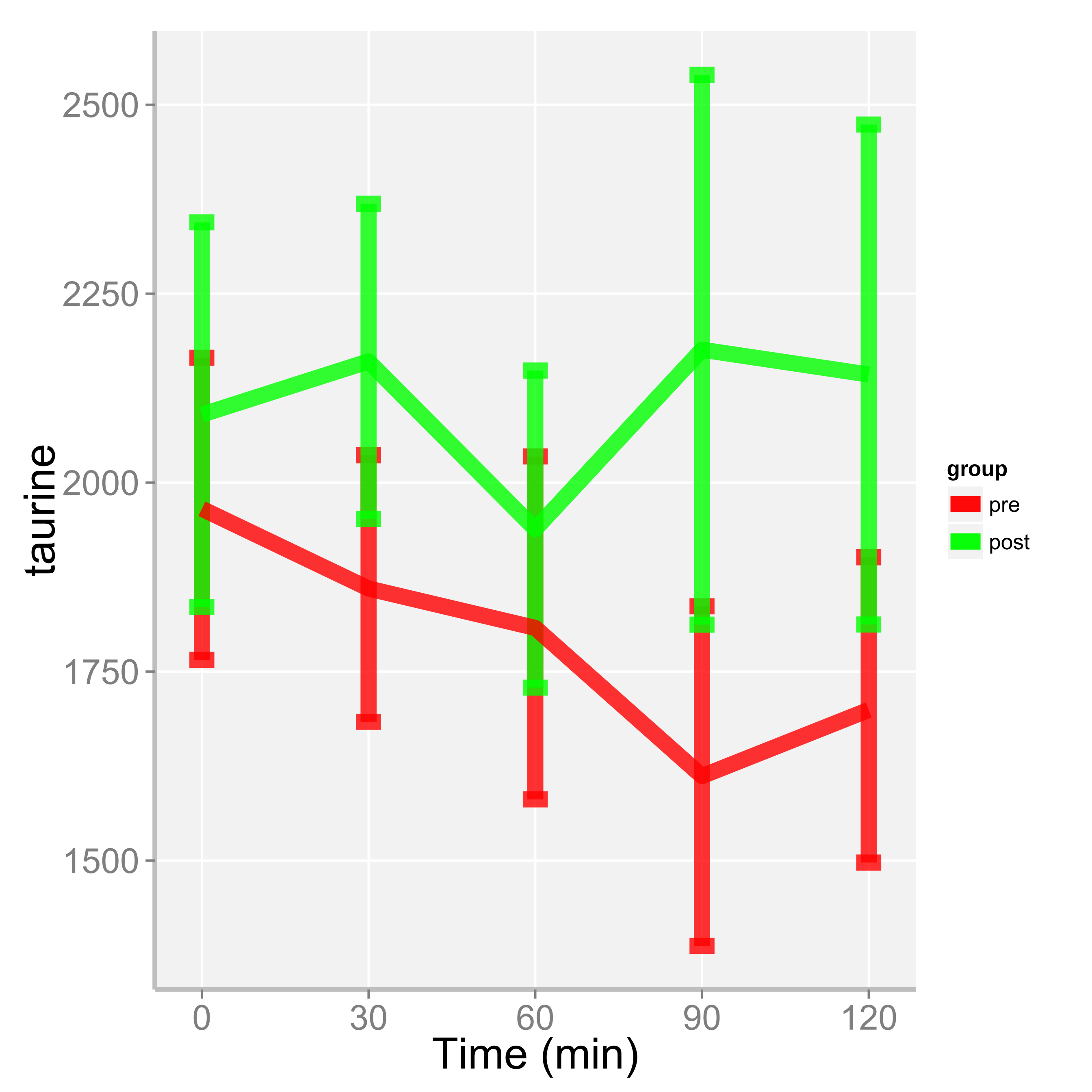
taurine
2500
2250
2000
1750
1500
0
30
60
90
120
Time (min)
group
pre
post

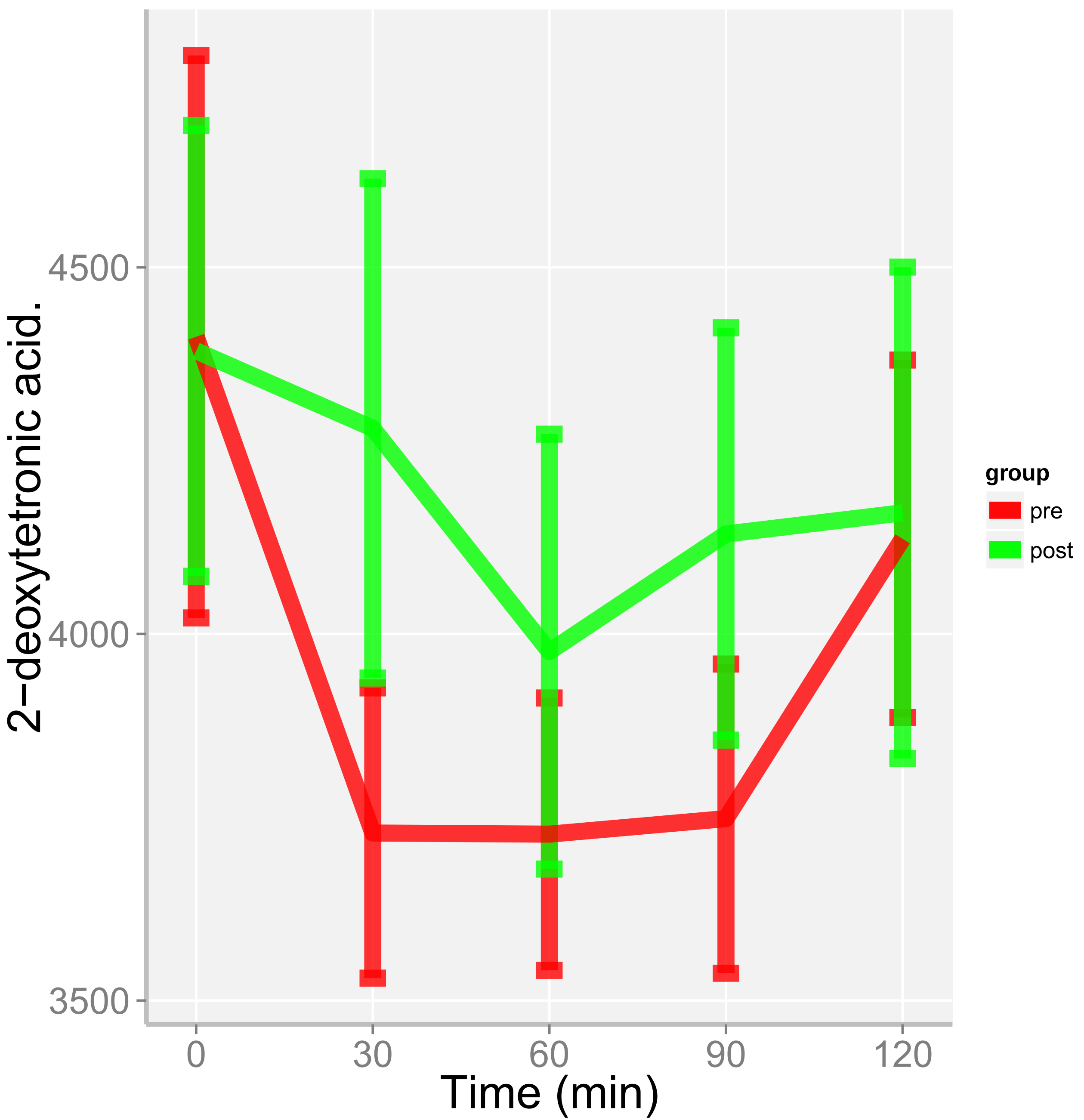

2−deoxytetronic acid.
4500
4000
3500
0
30
60
90
120
Time (min)
group
pre
post

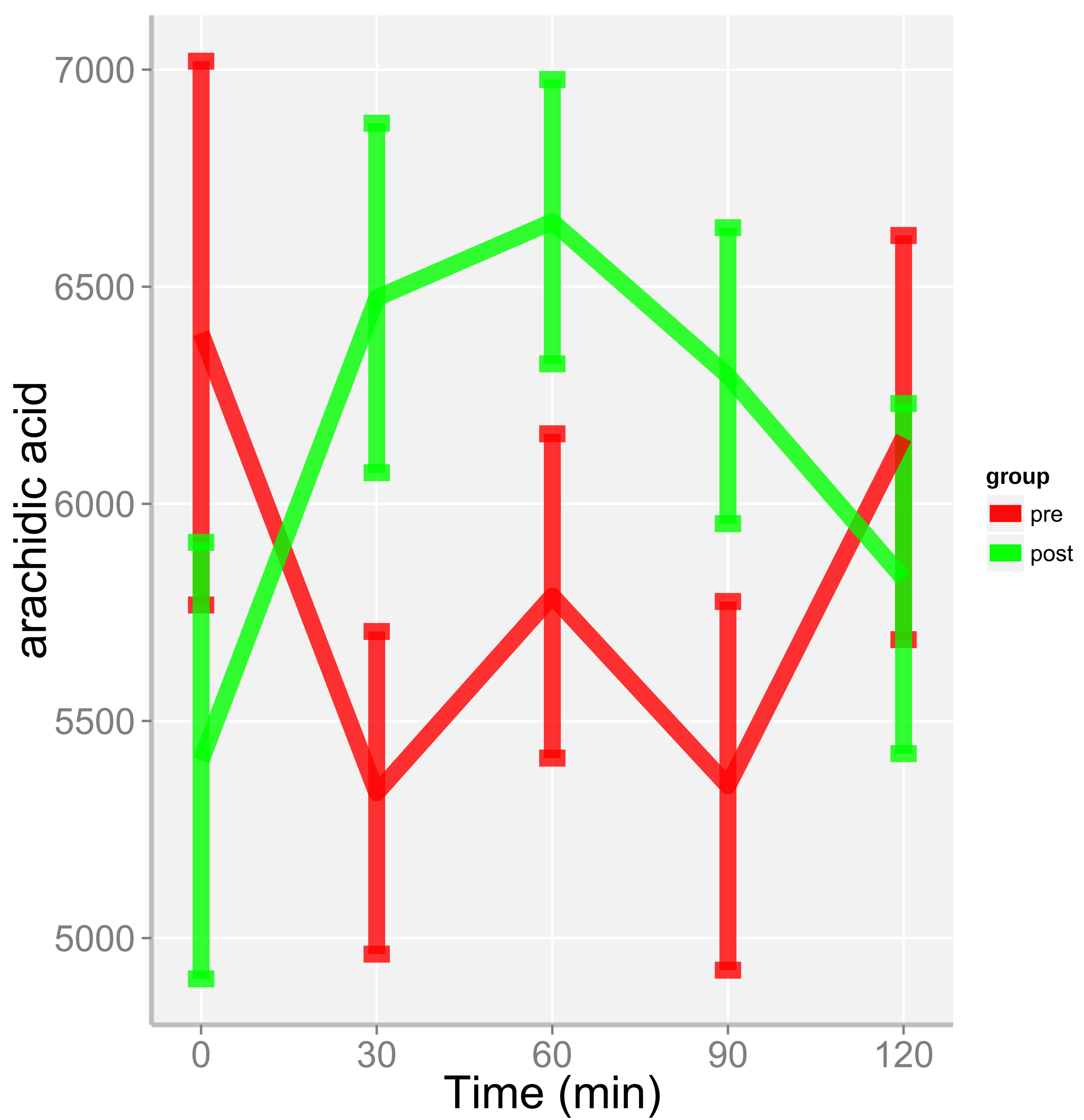

7000
6500
6000
5500
5000
arachidic acid
0
30
60
90
120
Time (min)
group
pre
post

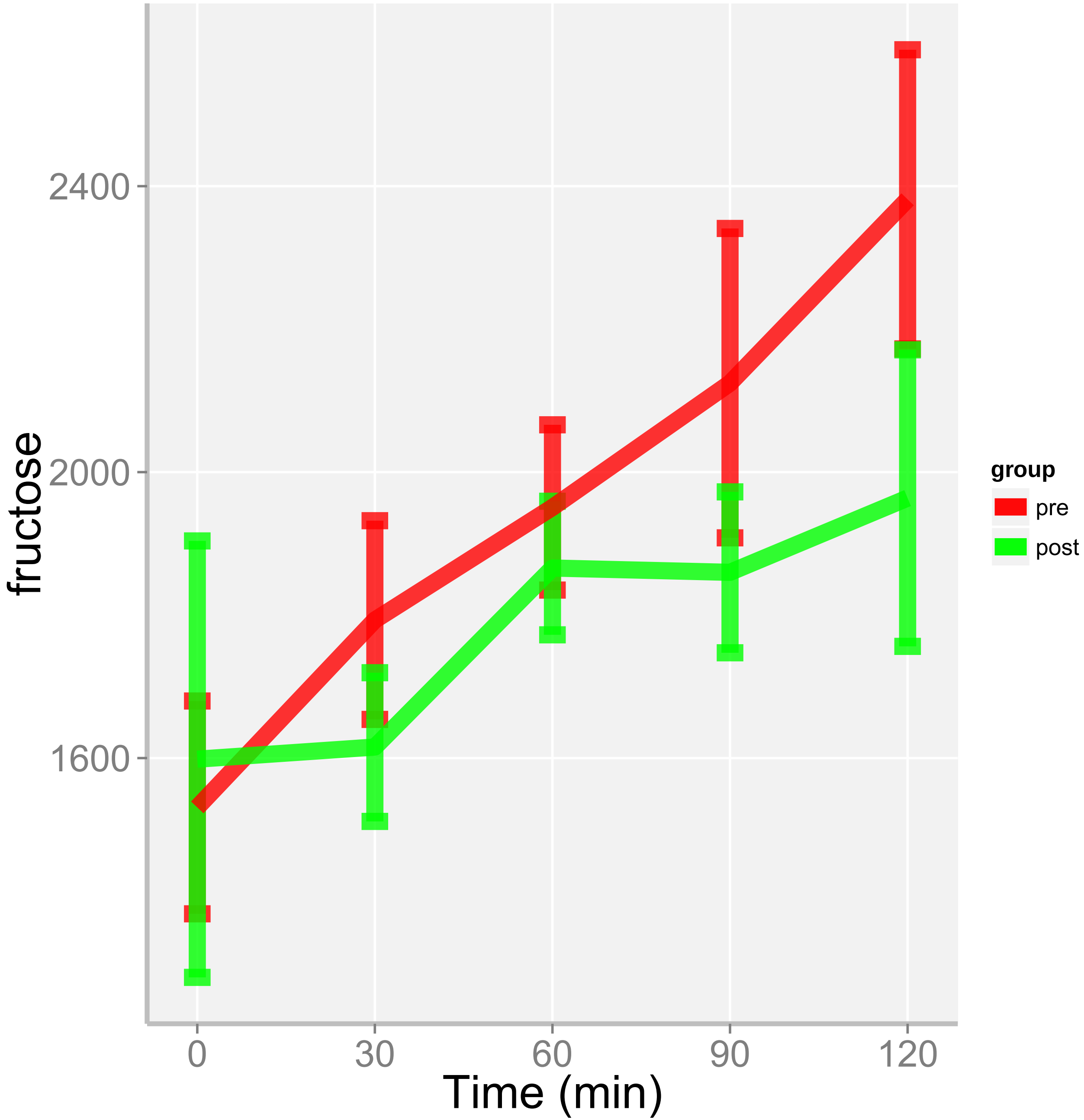

fructose
2400
2000
1600
0
30
60
90
120
Time (min)
group
pre
post

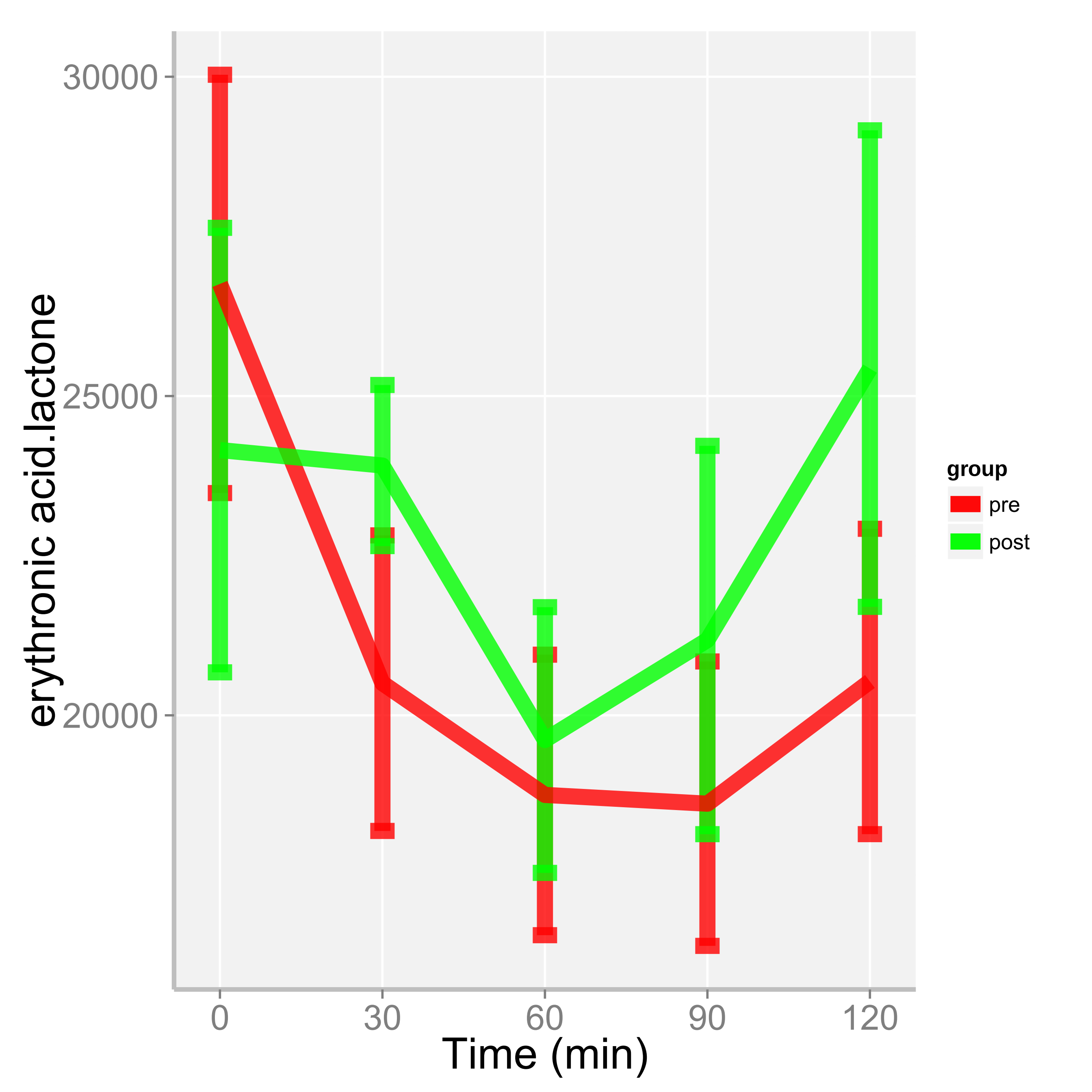

erythronic acid.lactone
30000
25000
20000
0
30
60
90
120
Time (min)
group
pre
post

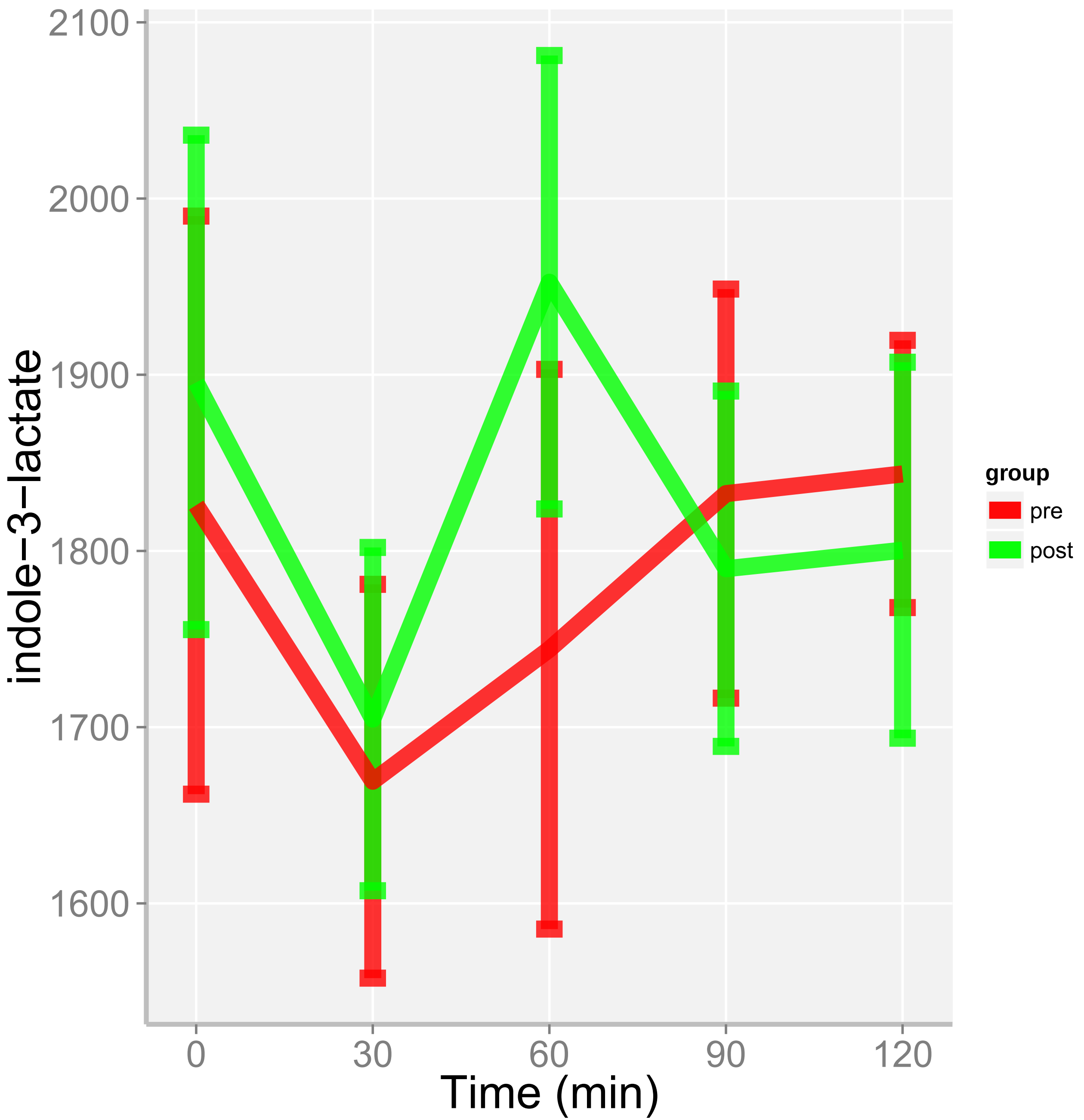

indole−3−lactate
2100
2000
1900
1800
1700
1600
0
30
60
90
120
Time (min)
group
pre
post

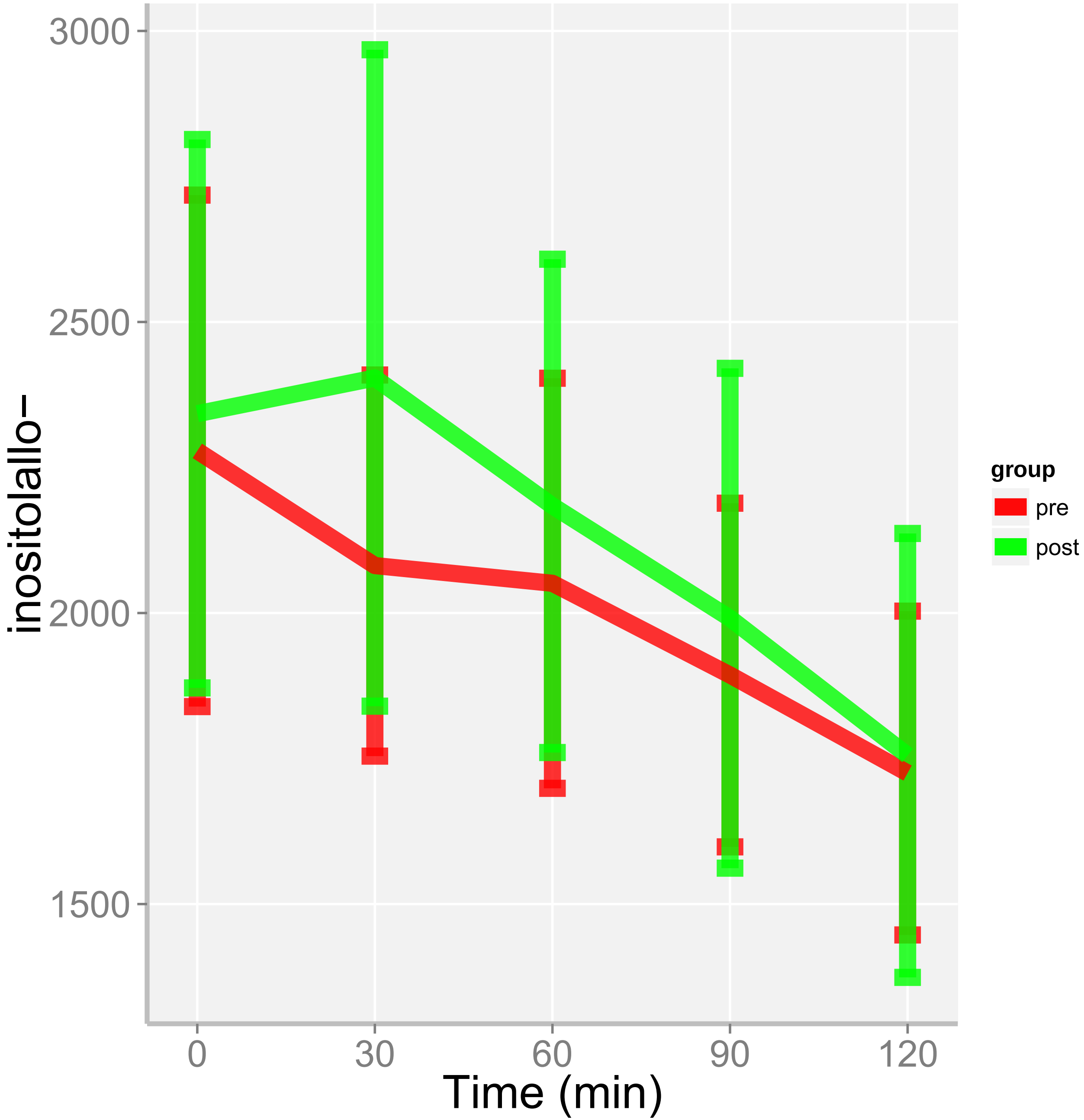

3000
2500
2000
1500
inositolallo−
0
30
60
90
120
Time (min)
group
pre
post

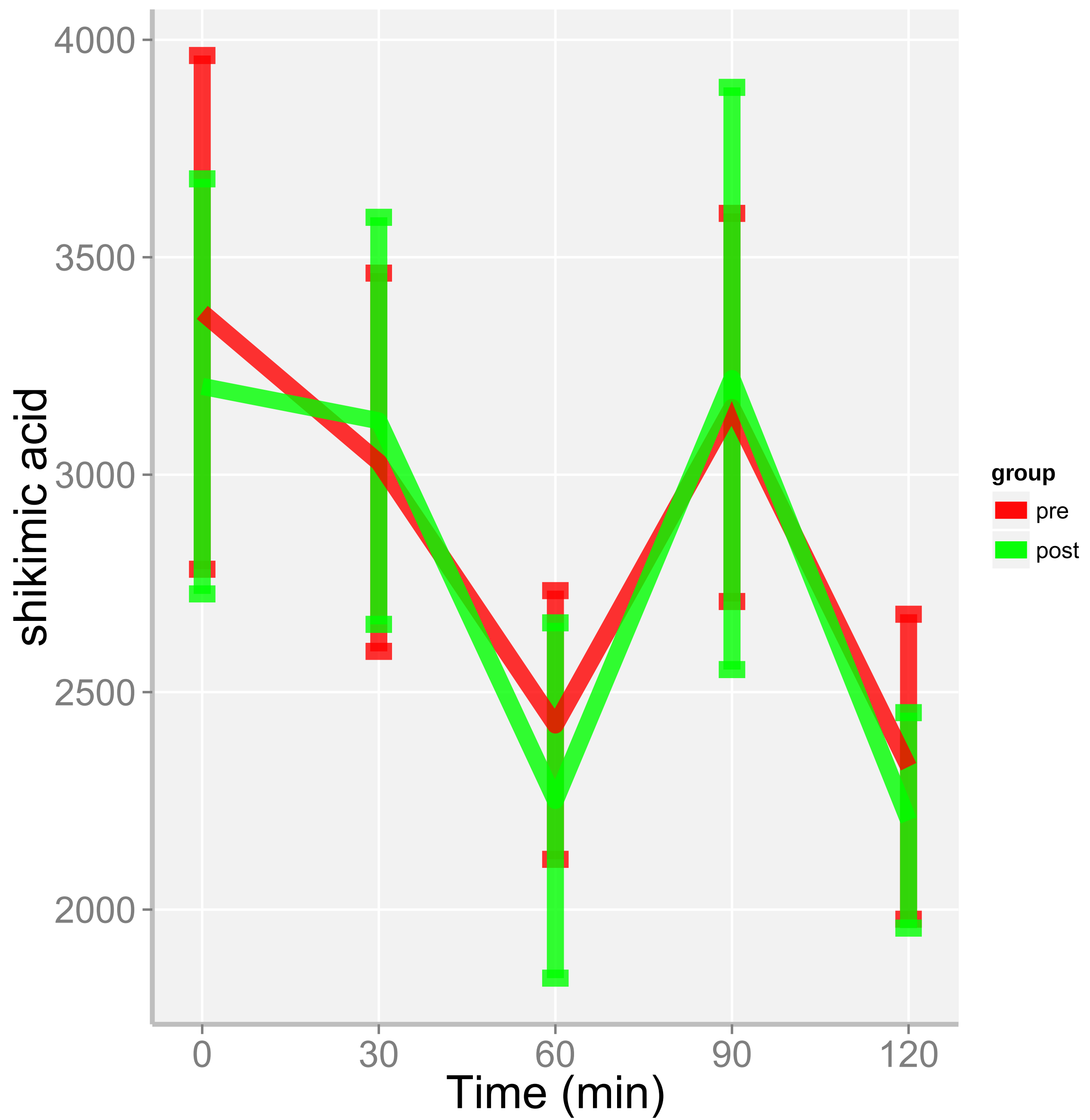

shikimic acid
4000
3500
3000
2500
2000
0
30
60
90
120
Time (min)
group
pre
post

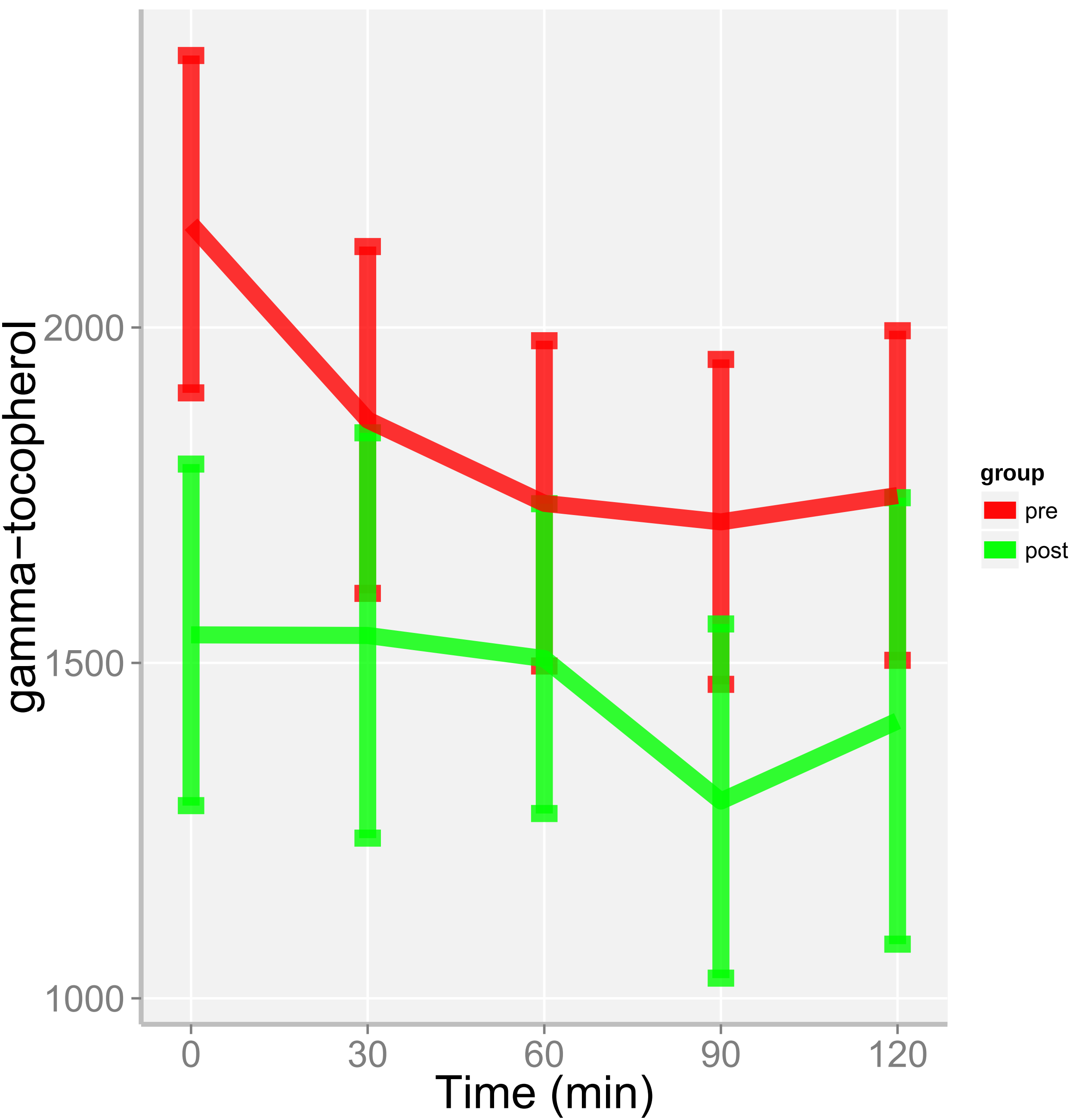
gamma−tocopherol
2000
1500
1000
0
30
60
90
120
Time (min)
group
pre
post

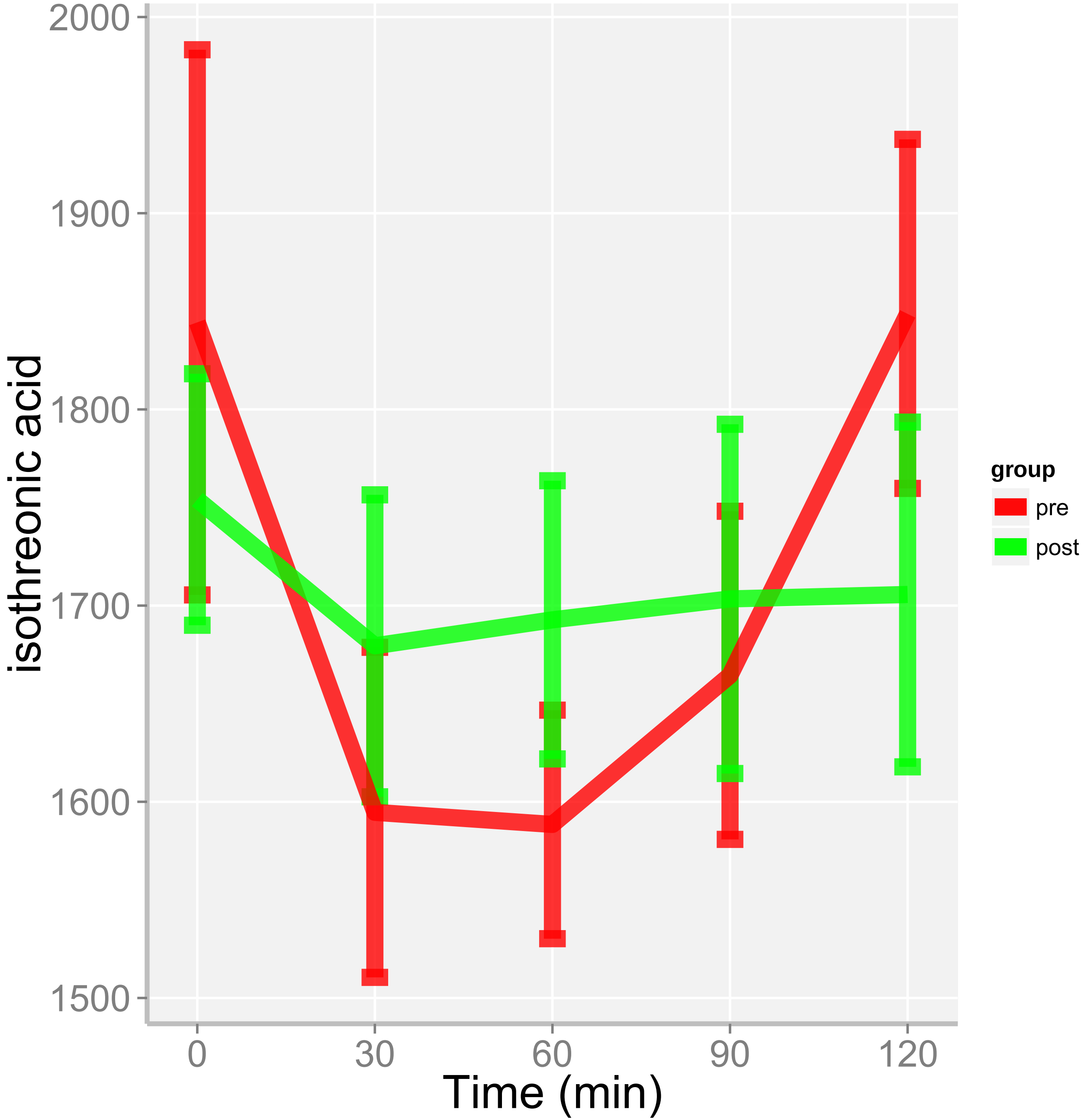
2000
1900
1800
1700
1600
1500
isothreonic acid
0
30
60
90
120
Time (min)
group
pre
post

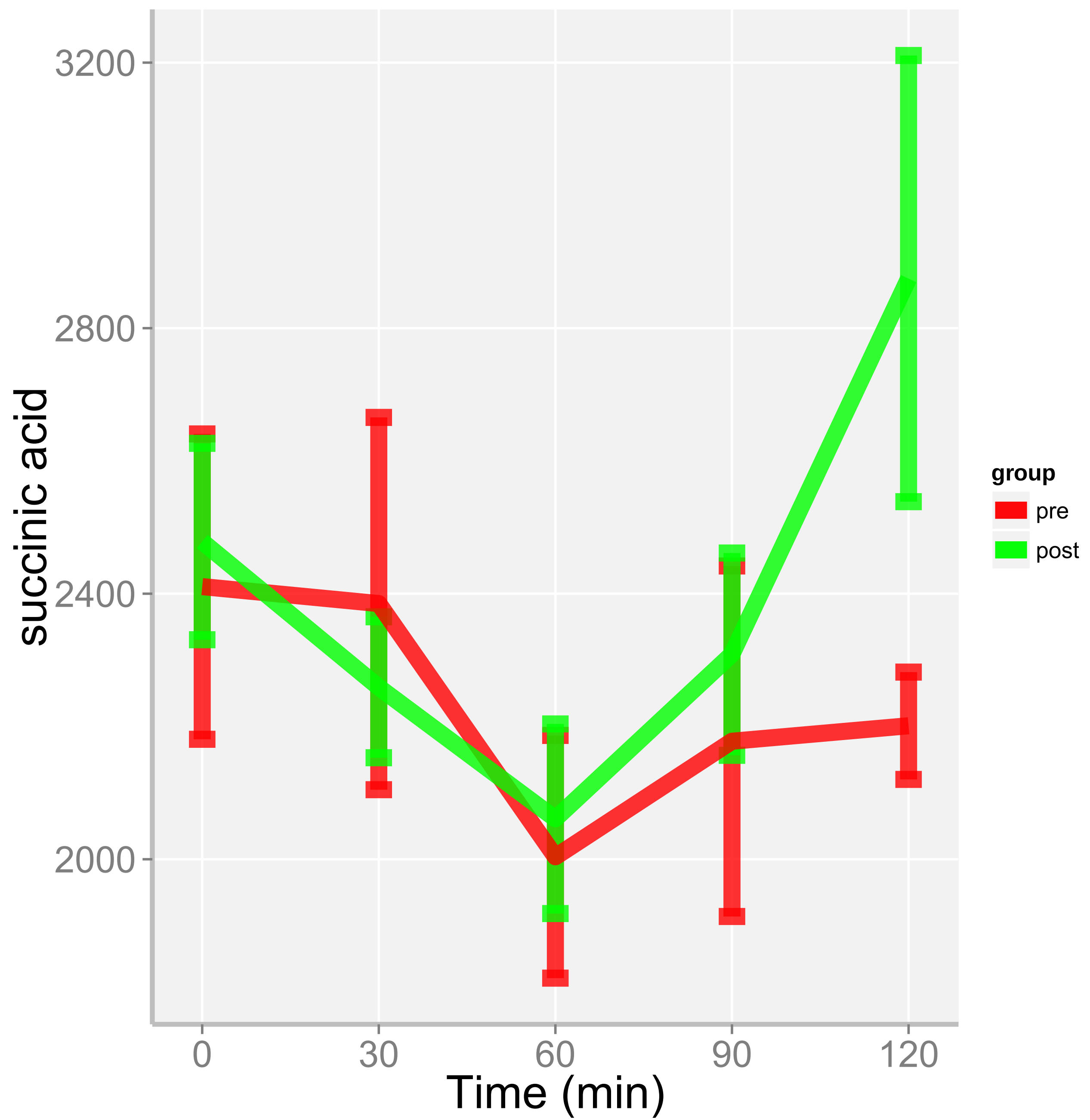

succinic acid
3200
2800
2400
2000
0
30
60
90
120
Time (min)
group
pre
post

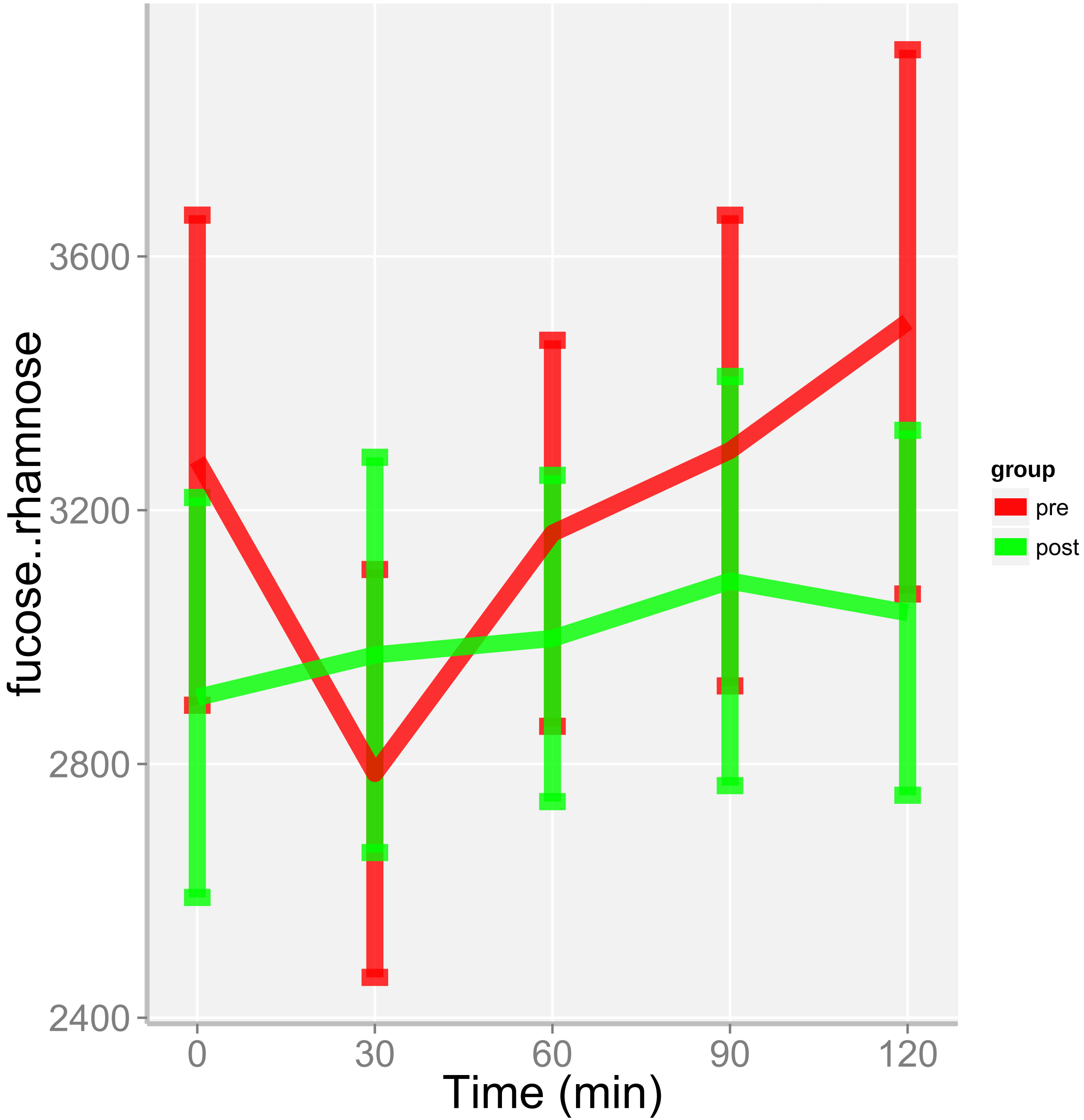

fucose..rhamnose
3600
3200
2800
2400
0
30
60
90
120
Time (min)
group
pre
post

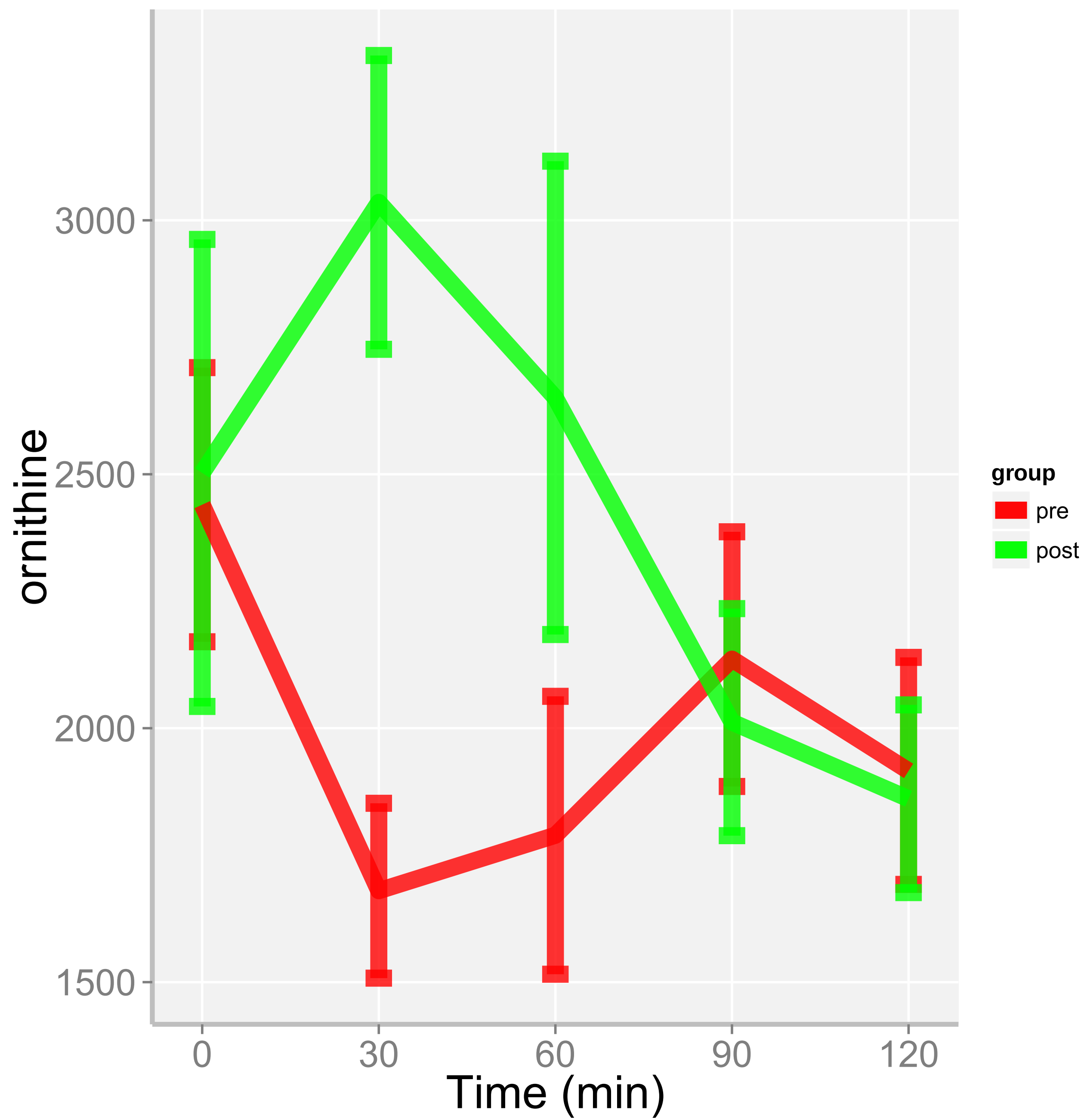

ornithine
3000
2500
2000
1500
0
30
60
90
120
Time (min)
group
pre
post

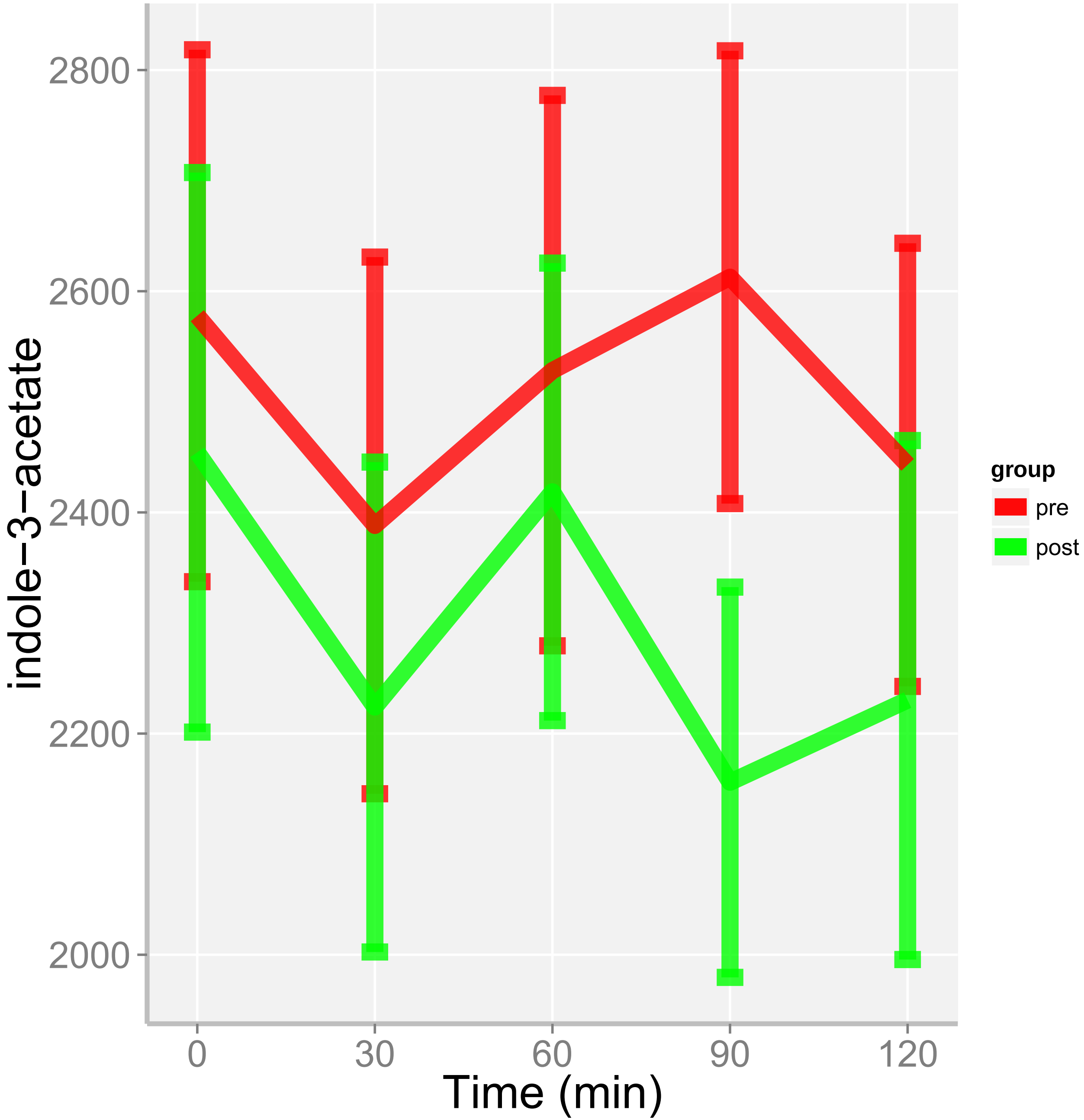

indole−3−acetate
2800
2600
2400
2200
2000
0
30
60
90
120
Time (min)
group
pre
post

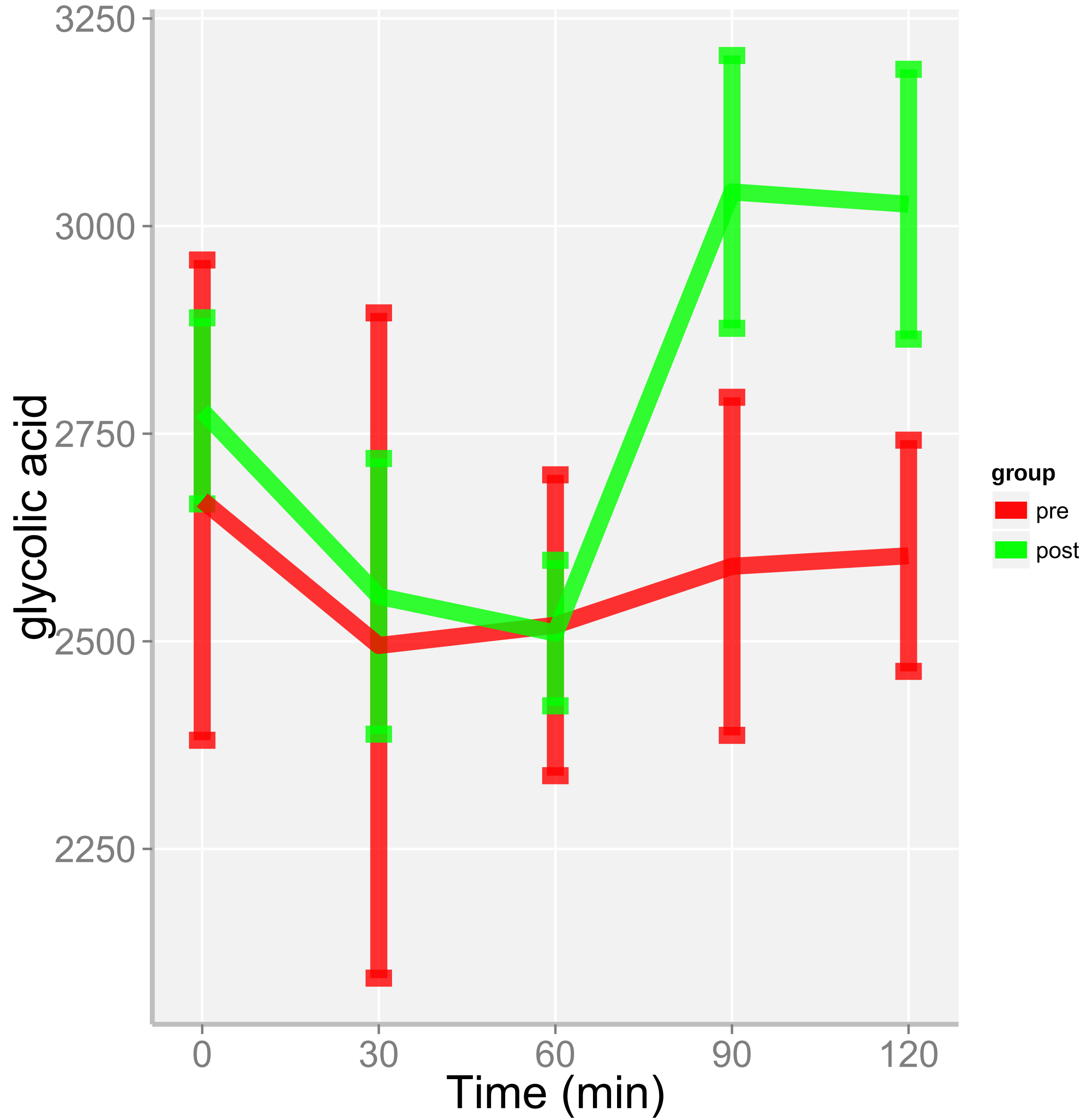

3250
3000
2750
2500
2250
glycolic acid
0
30
60
90
120
Time (min)
group
pre
post

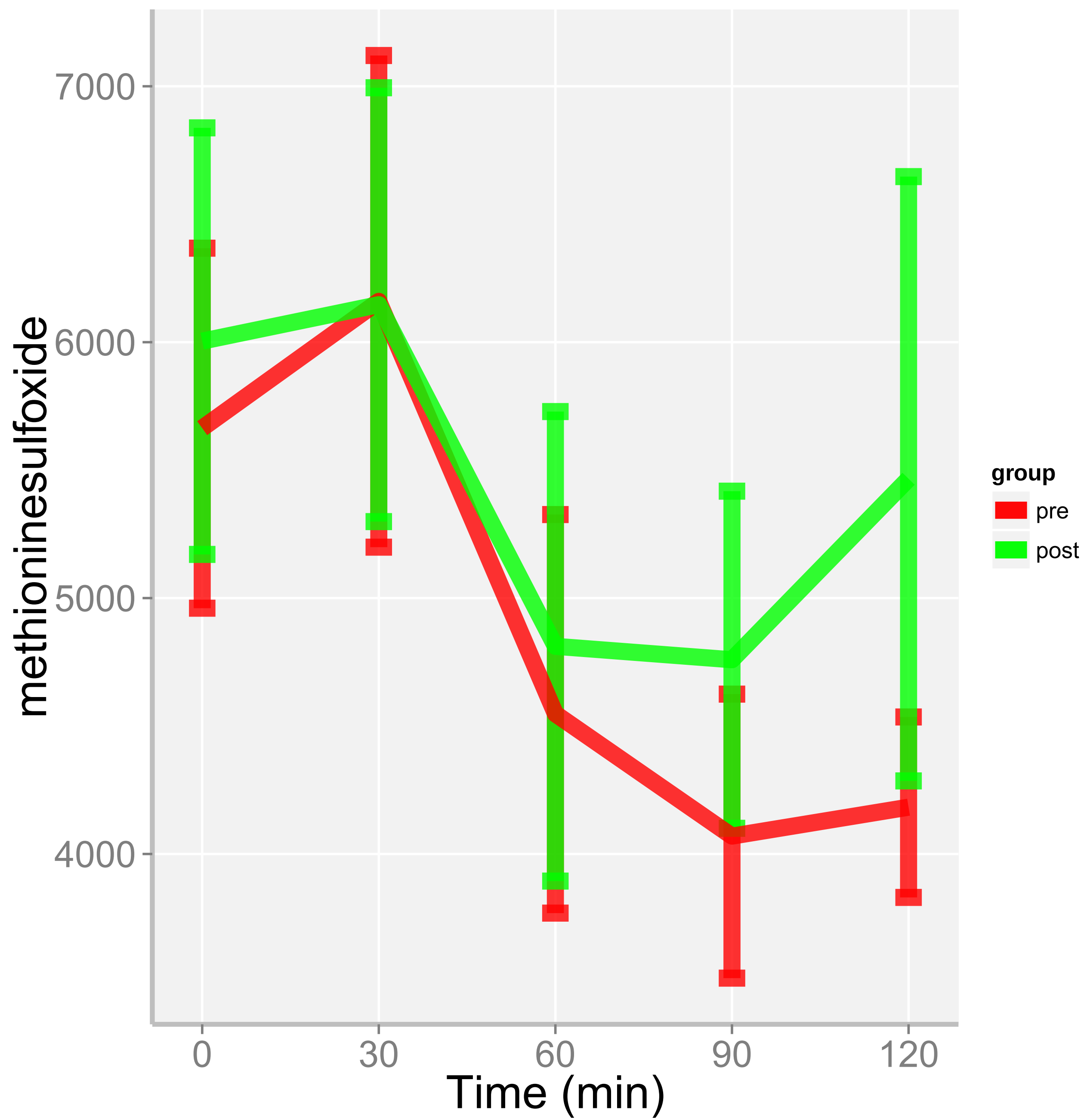

methioninesulfoxide
7000
6000
5000
4000
0
30
60
90
120
Time (min)
group
pre
post

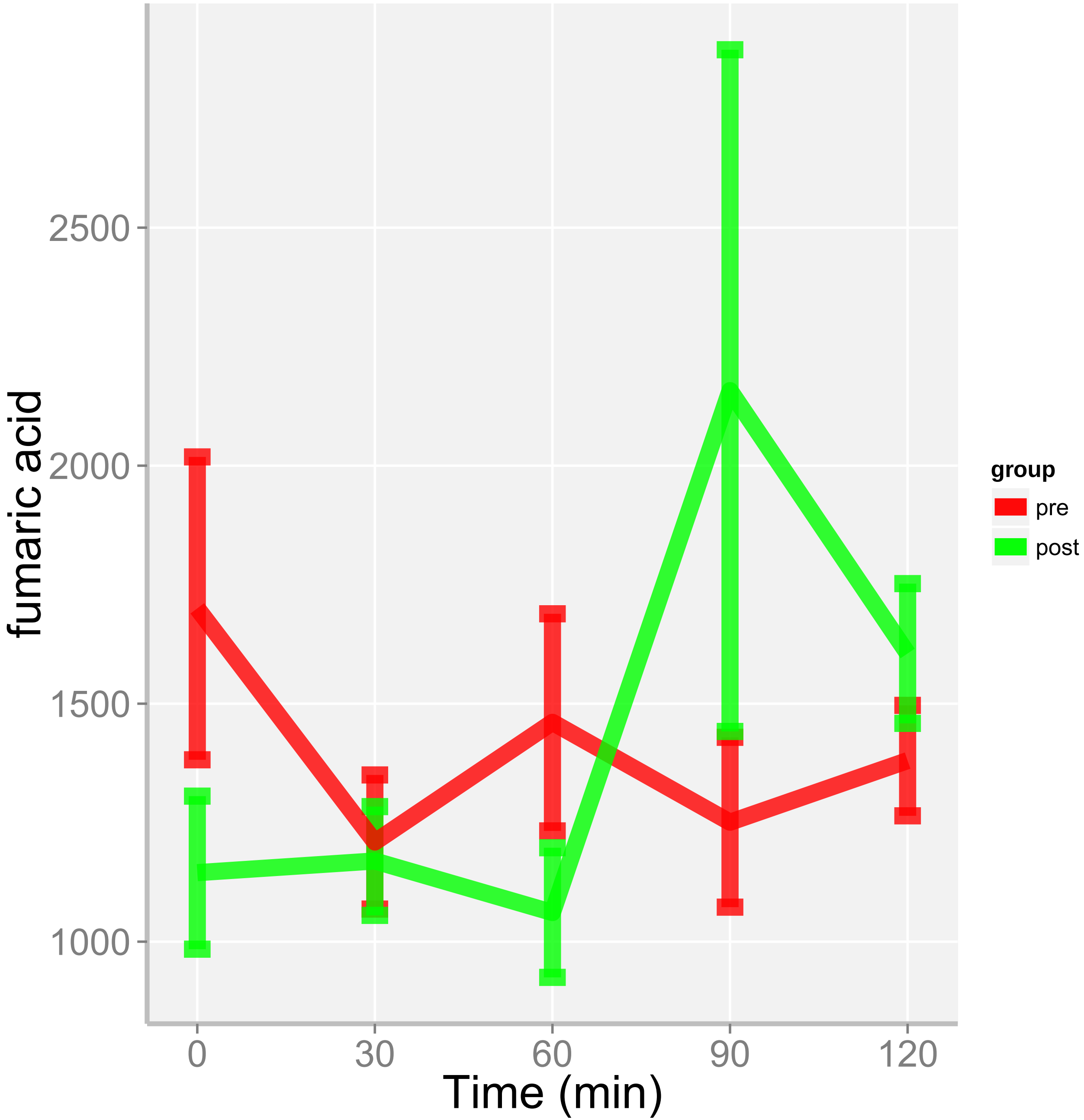

fumaric acid
2500
2000
1500
1000
0
30
60
90
120
Time (min)
group
pre
post

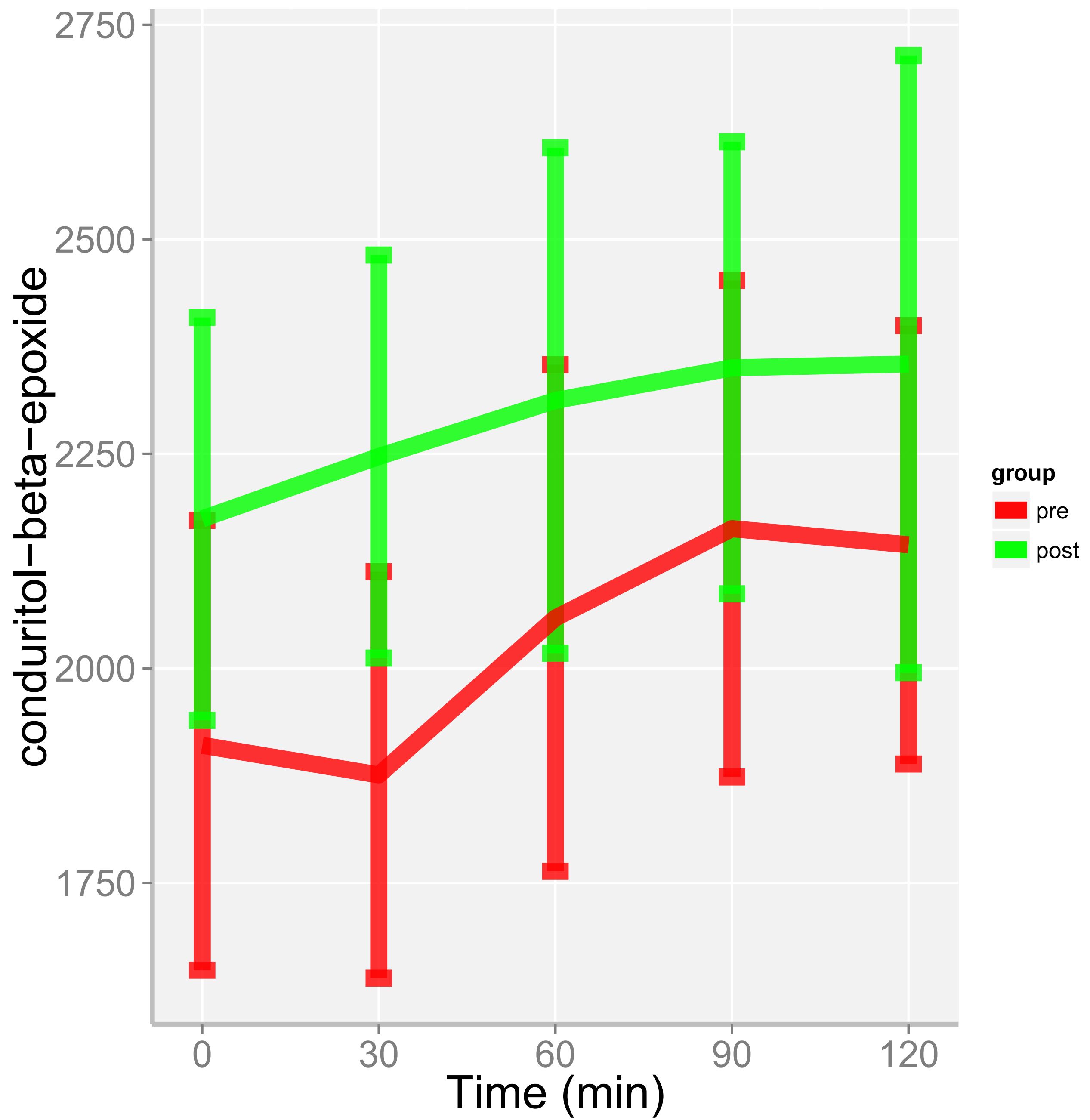

conduritol−beta−epoxide
2750
2500
2250
2000
1750
0
30
60
90
120
Time (min)
group
pre
post

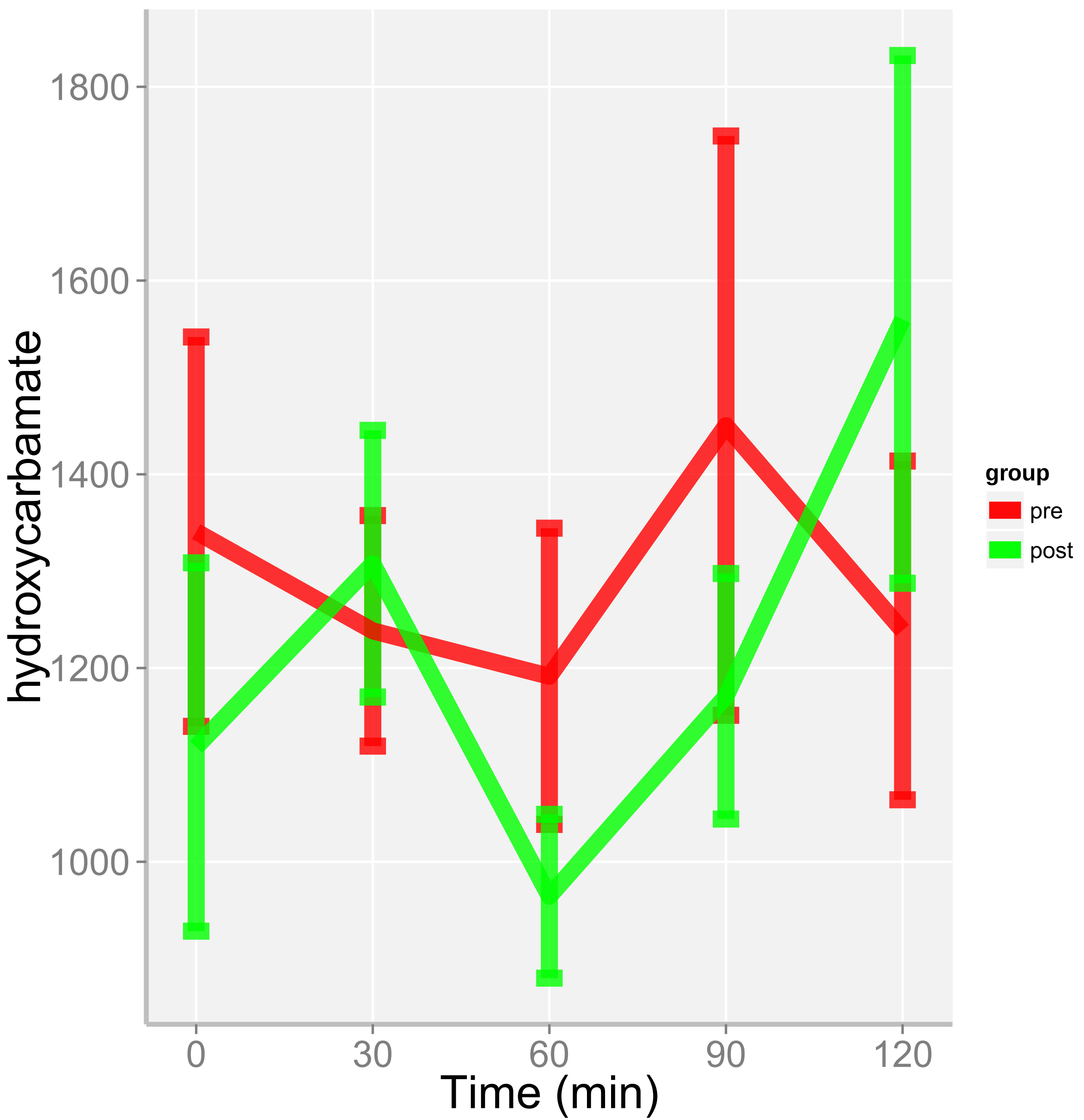

hydroxycarbamate
1800
1600
1400
1200
1000
0
30
60
90
120
Time (min)
group
pre
post

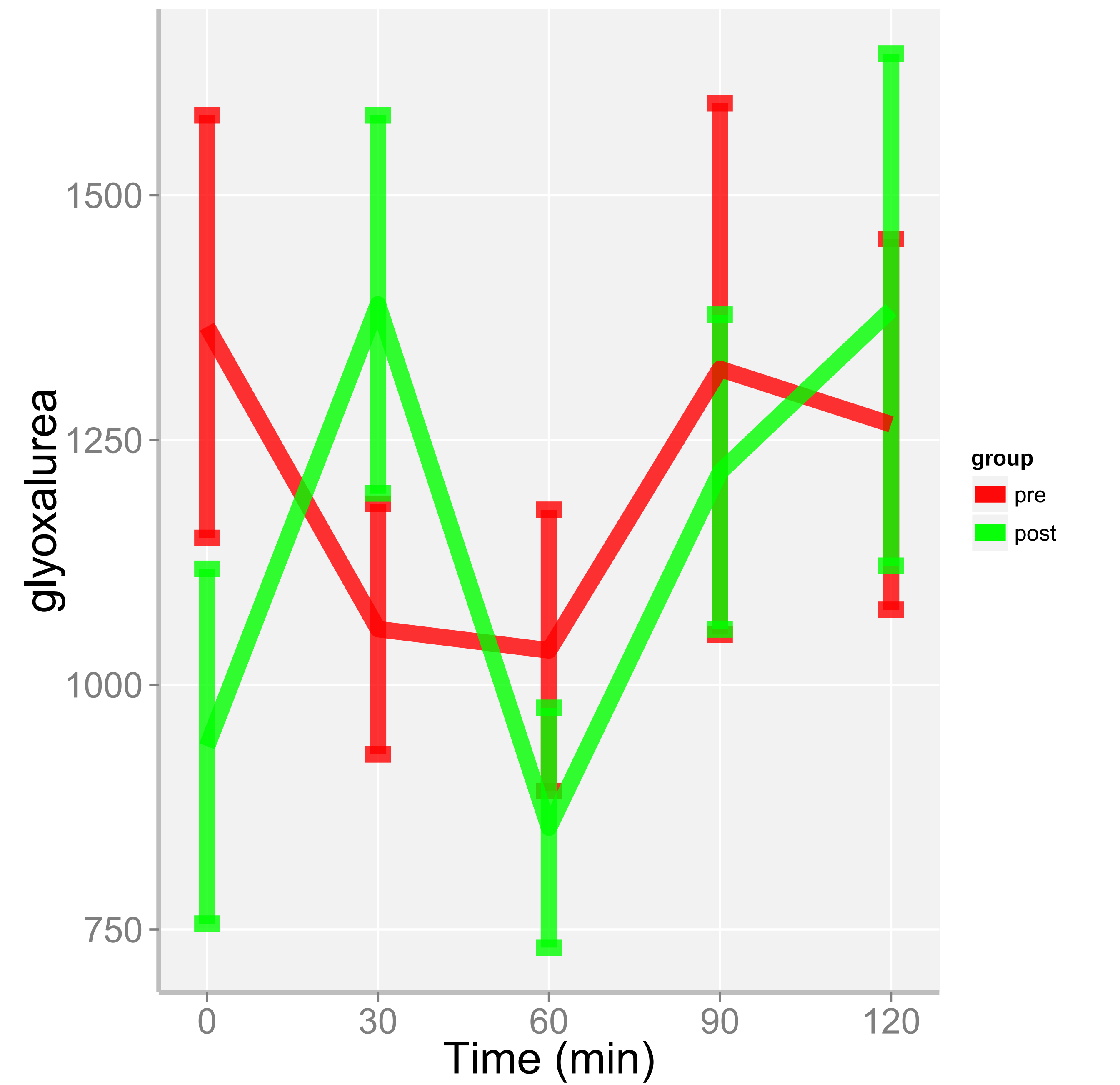

glyoxalurea
1500
1250
1000
750
0
30
60
90
120
Time (min)
group
pre
post

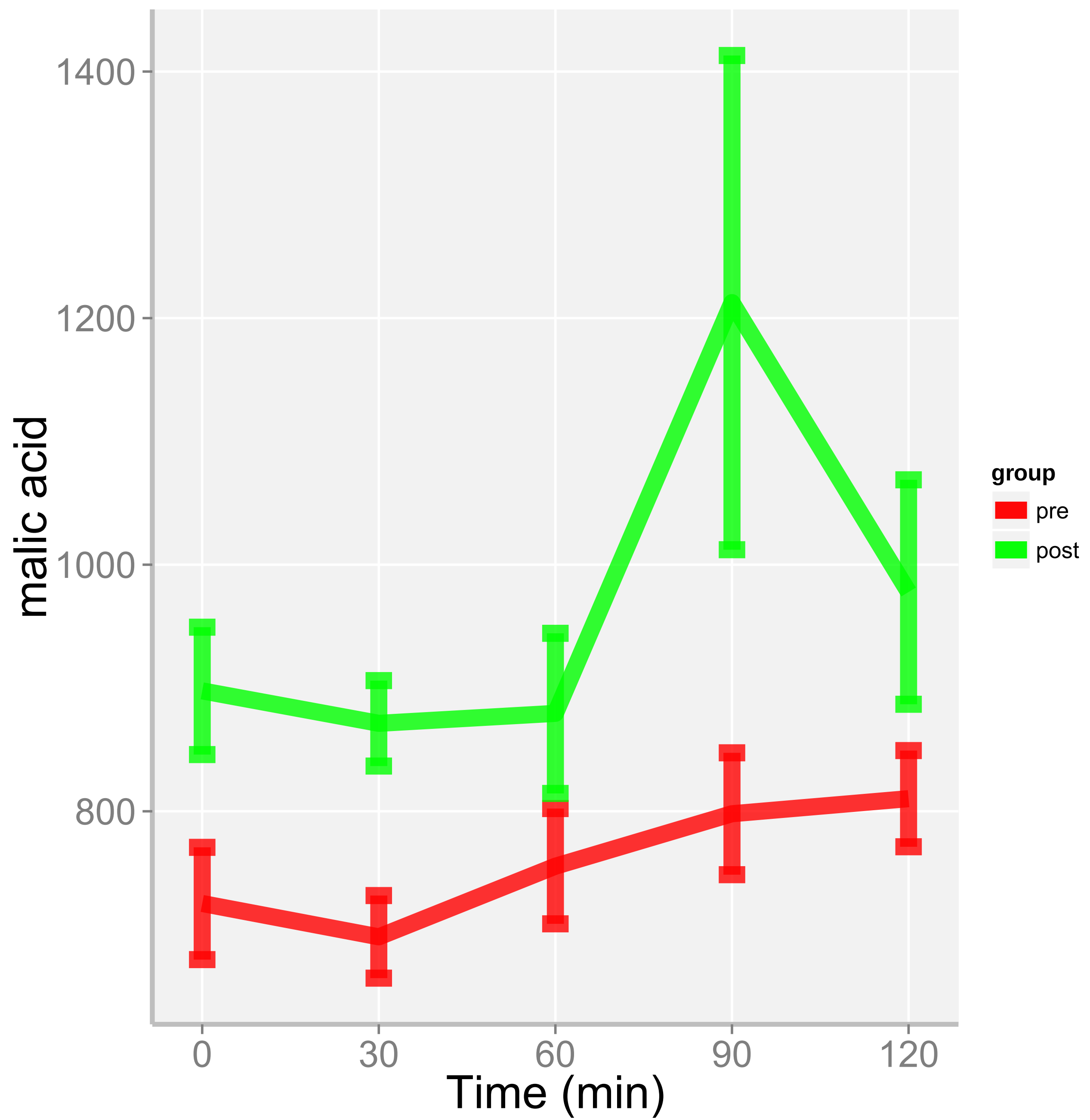

malic acid
1400
1200
1000
800
0
30
60
90
120
Time (min)
group
pre
post

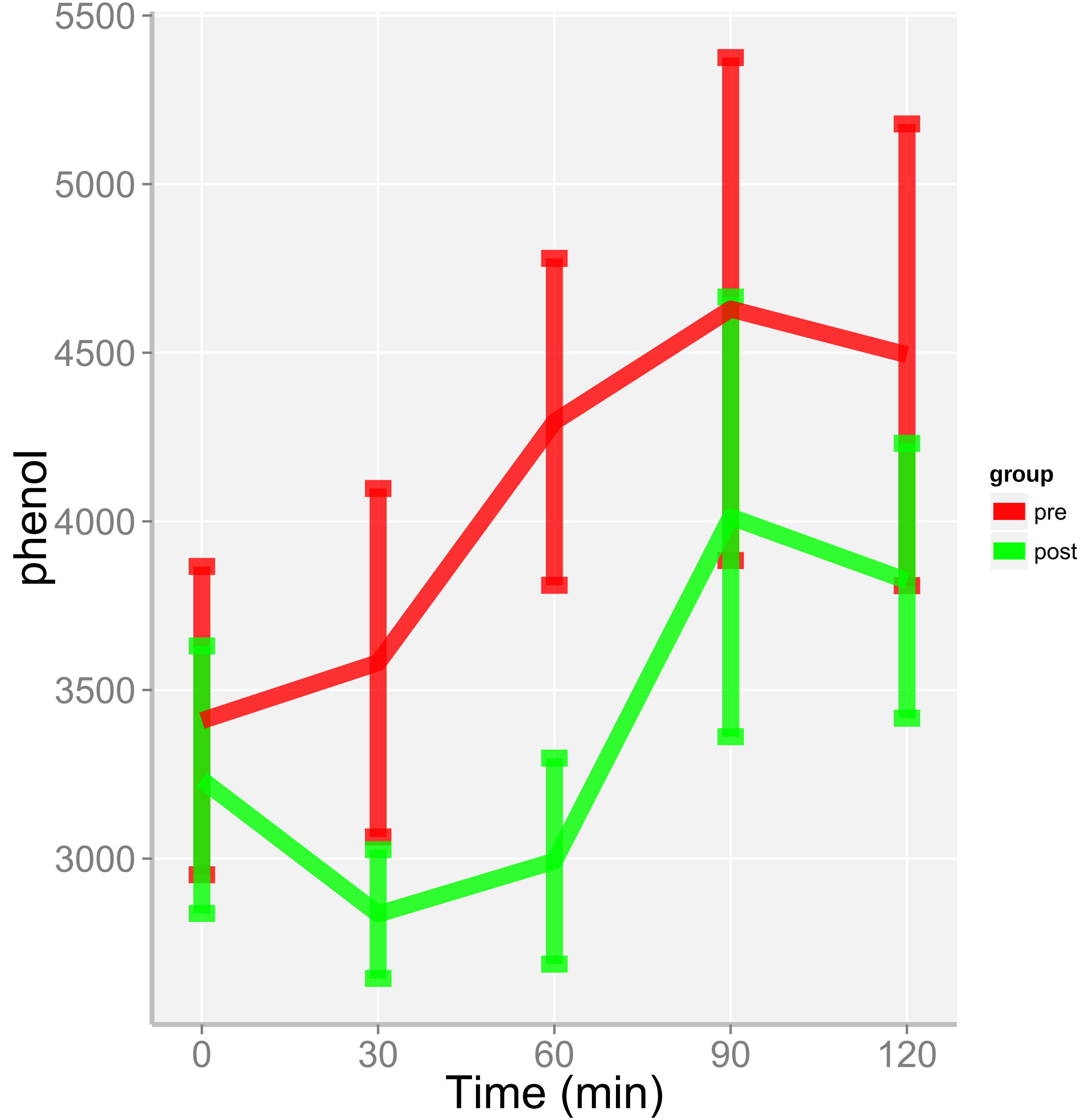

5500
5000
4500
4000
3500
3000
phenol
0
30
60
90
120
Time (min)
group
pre
post

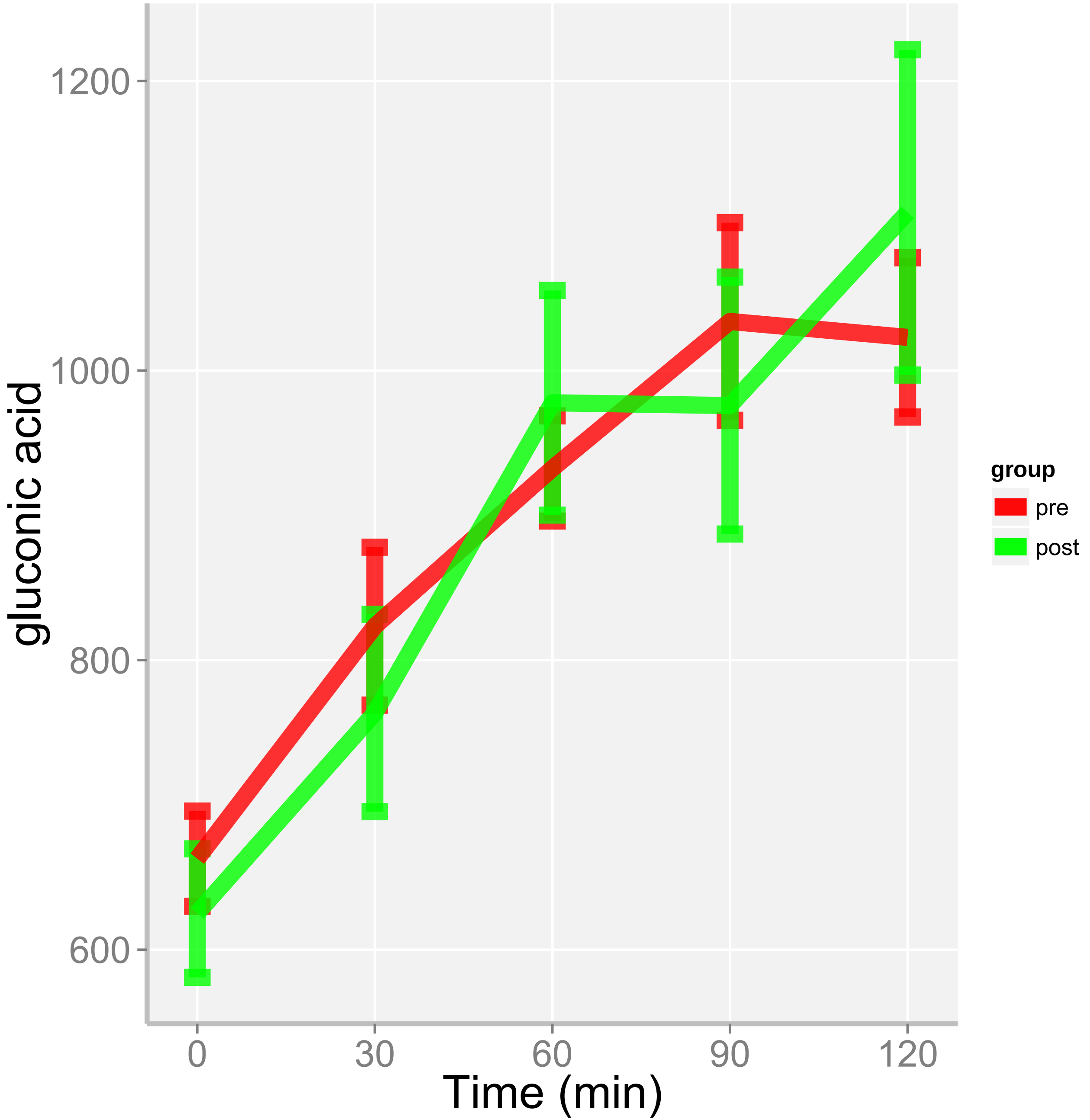

gluconic acid
1200
1000
800
600
0
30
60
90
120
Time (min)
group
pre
post

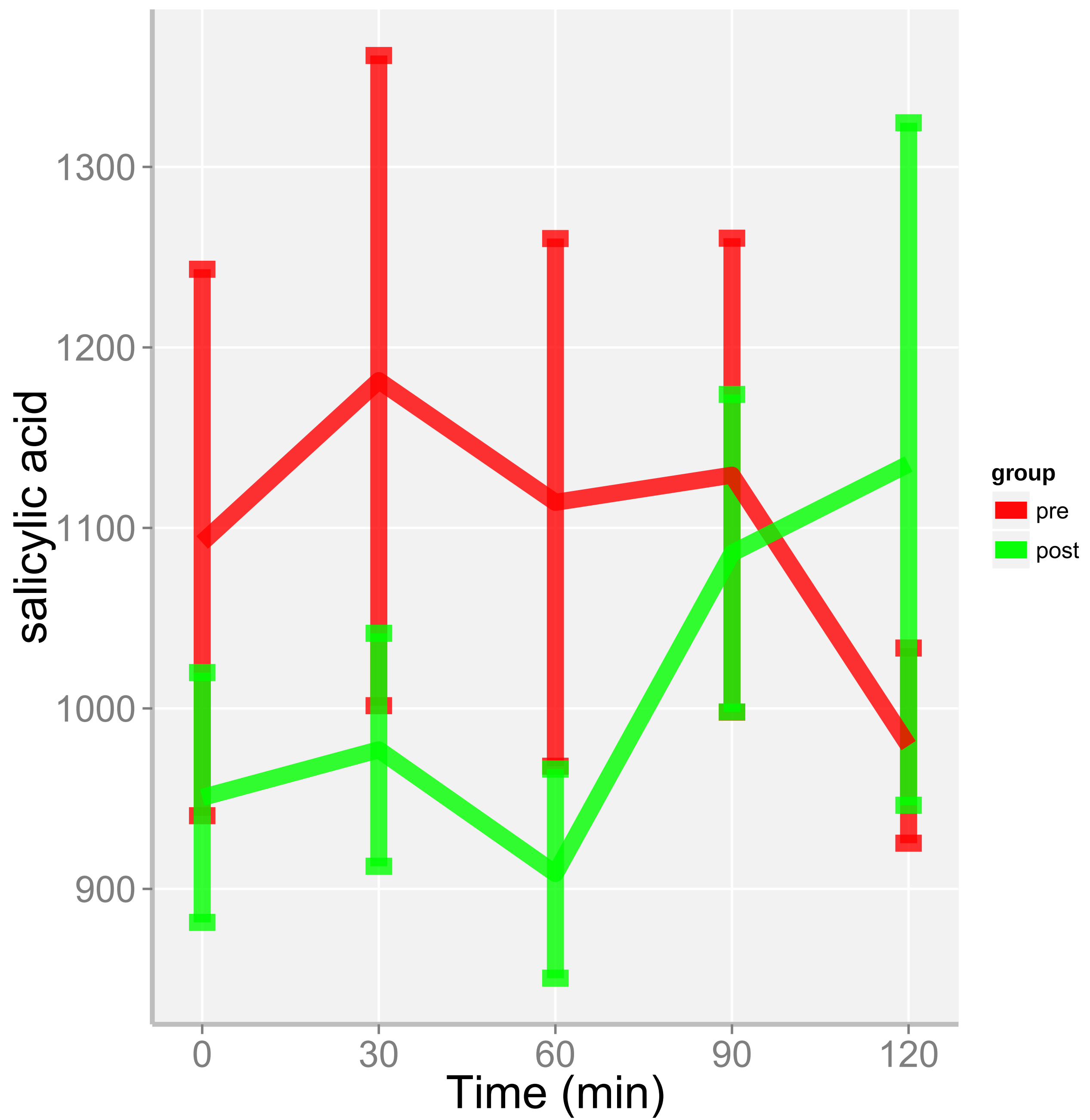

salicylic acid
1300
1200
1100
1000
900
0
30
60
90
120
Time (min)
group
pre
post

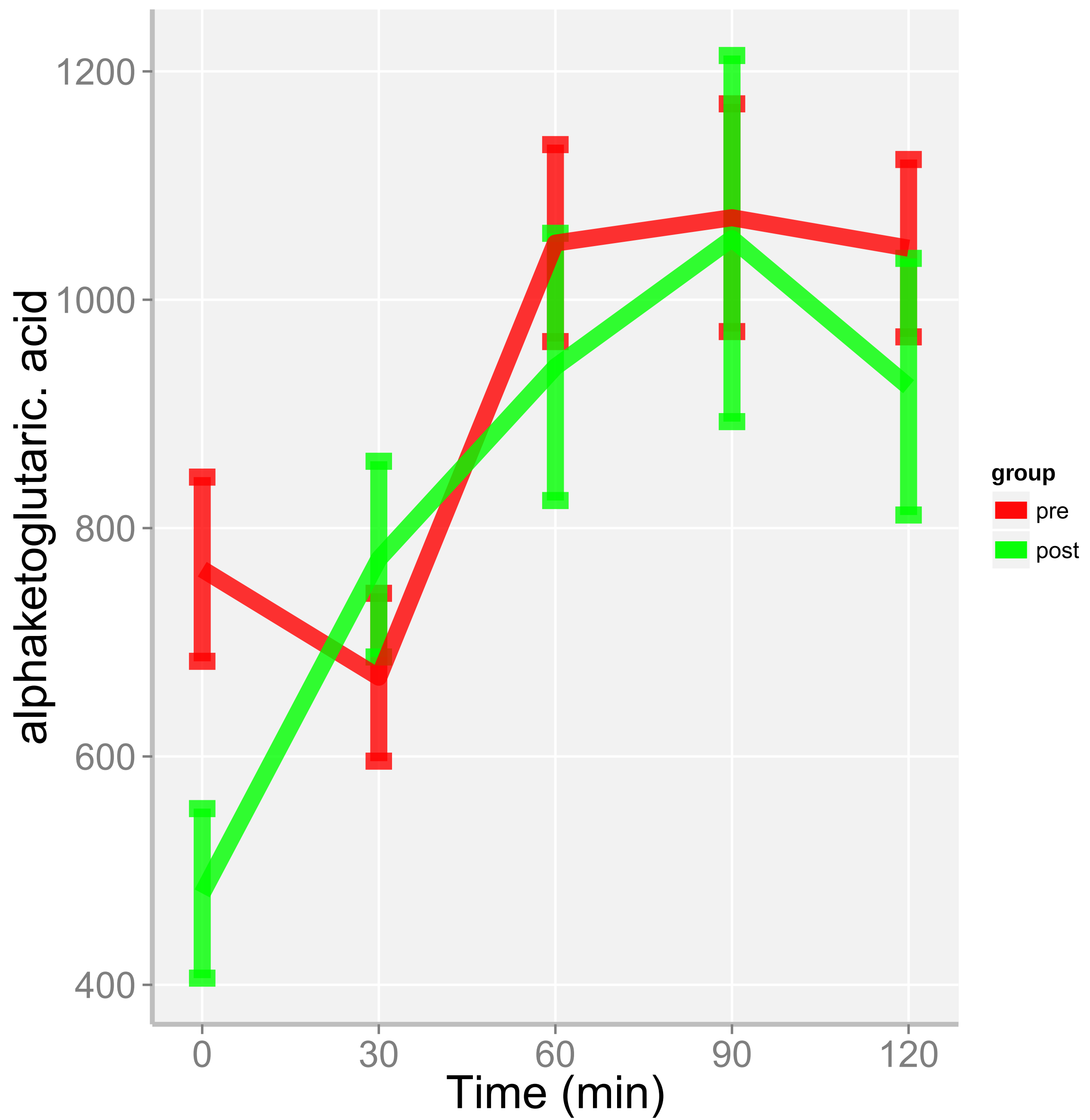

alphaketoglutaric. acid
1200
1000
800
600
400
0
30
60
90
120
Time (min)
group
pre
post

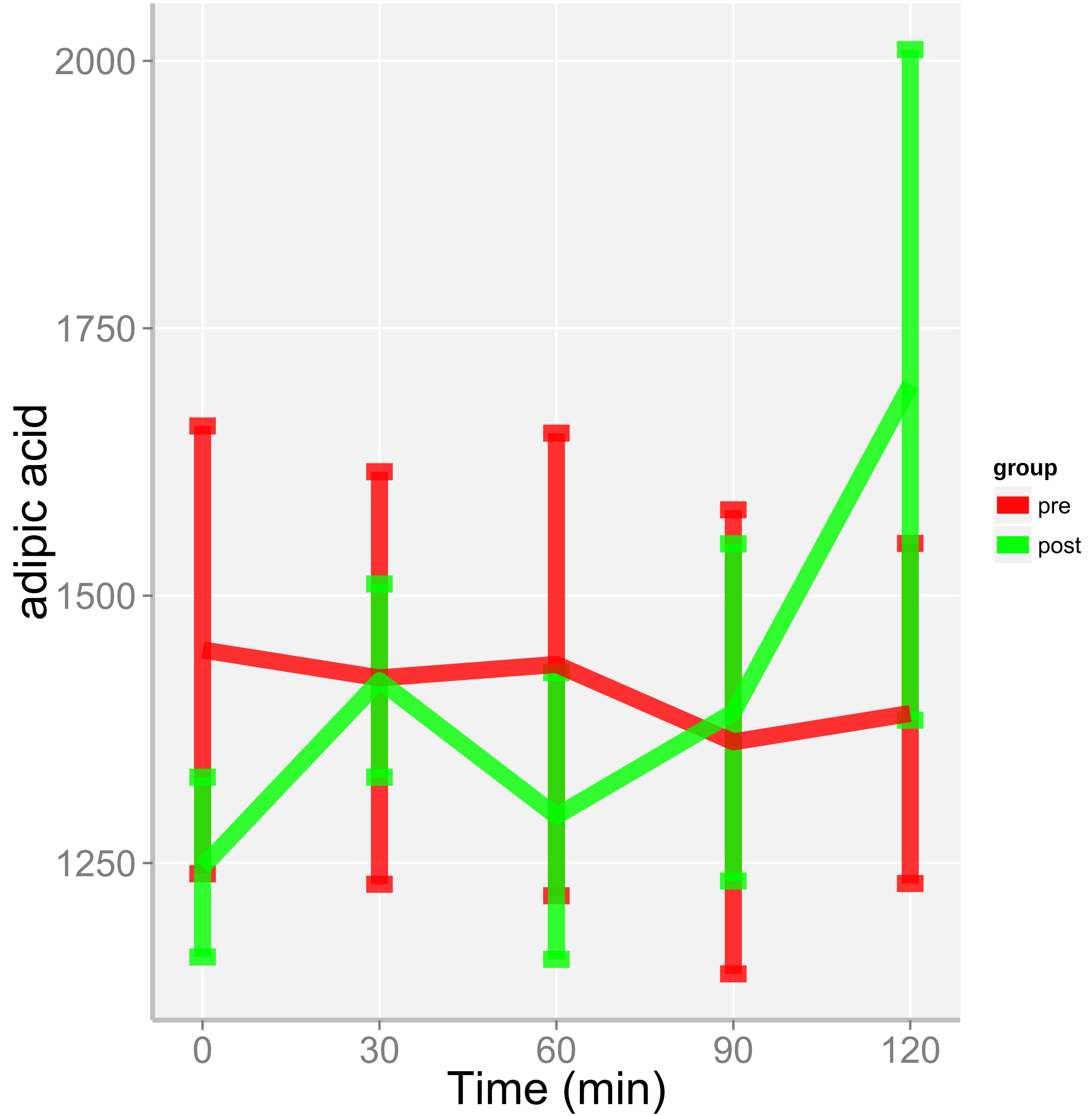

adipic acid
2000
1750
1500
1250
0
30
60
90
120
Time (min)
group
pre
post

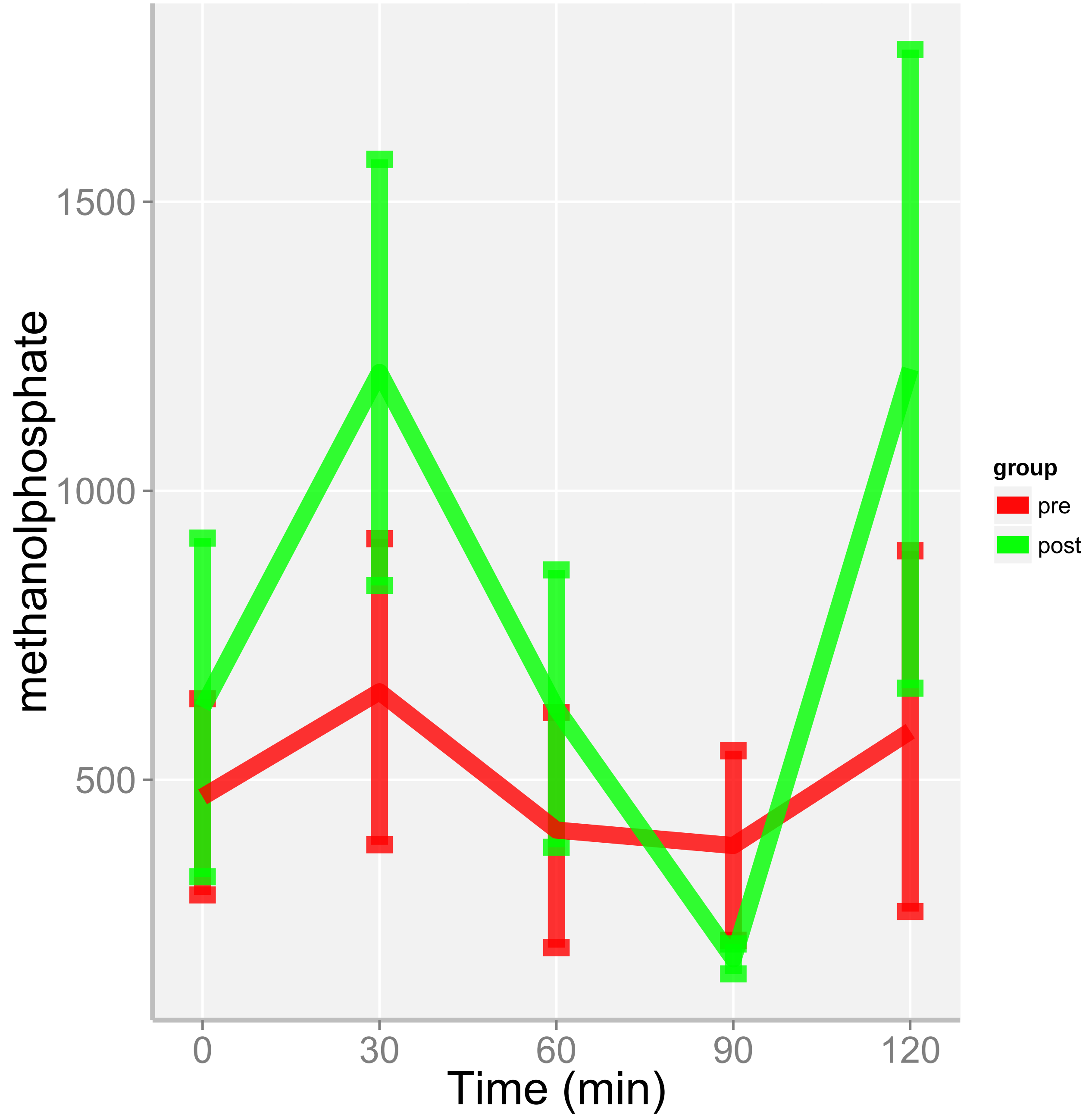
methanolphosphate
1500
1000
500
0
30
60
90
120
Time (min)
group
pre
post

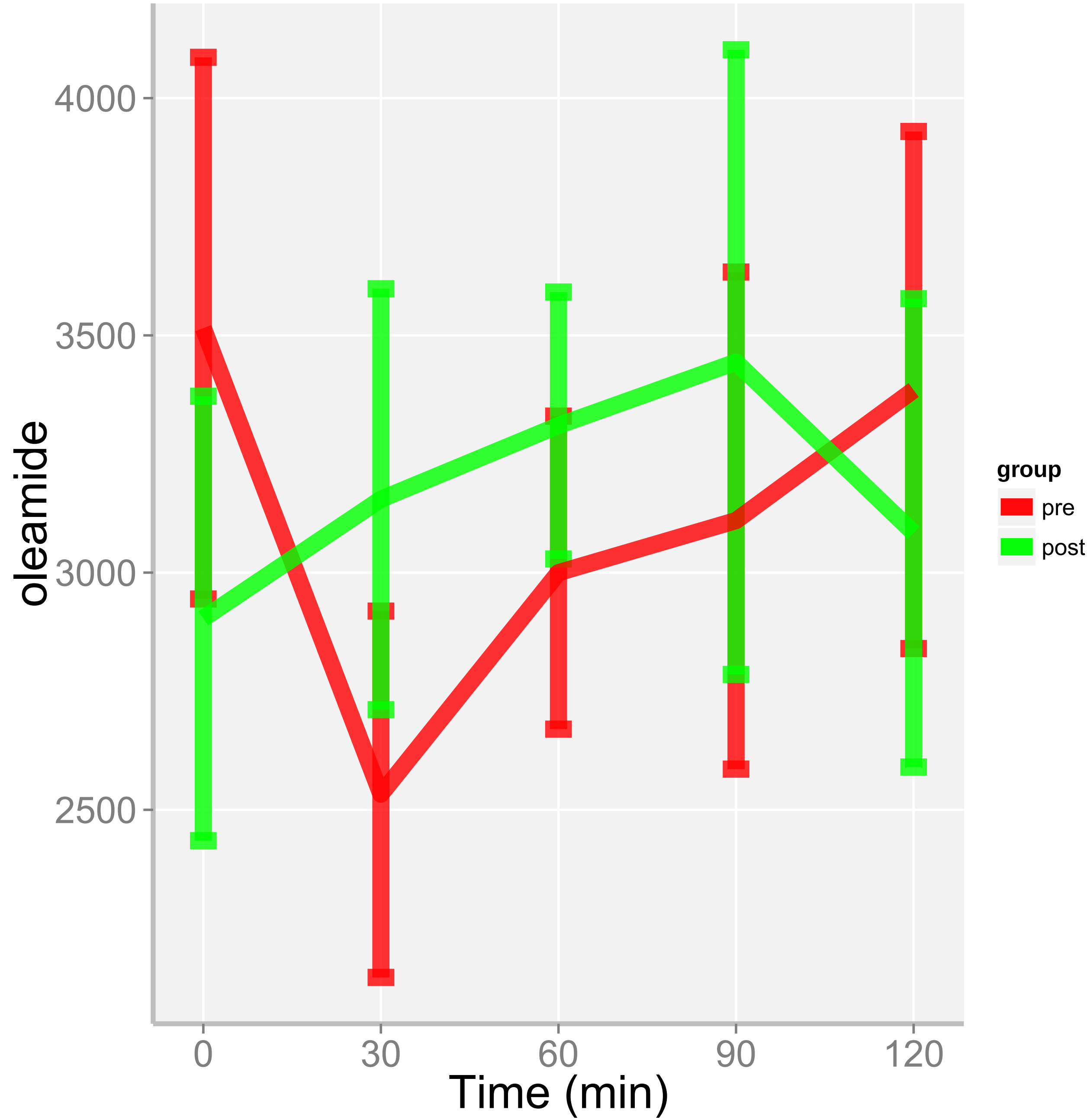

oleamide
4000
3500
3000
2500
0
30
60
90
120
Time (min)
group
pre
post

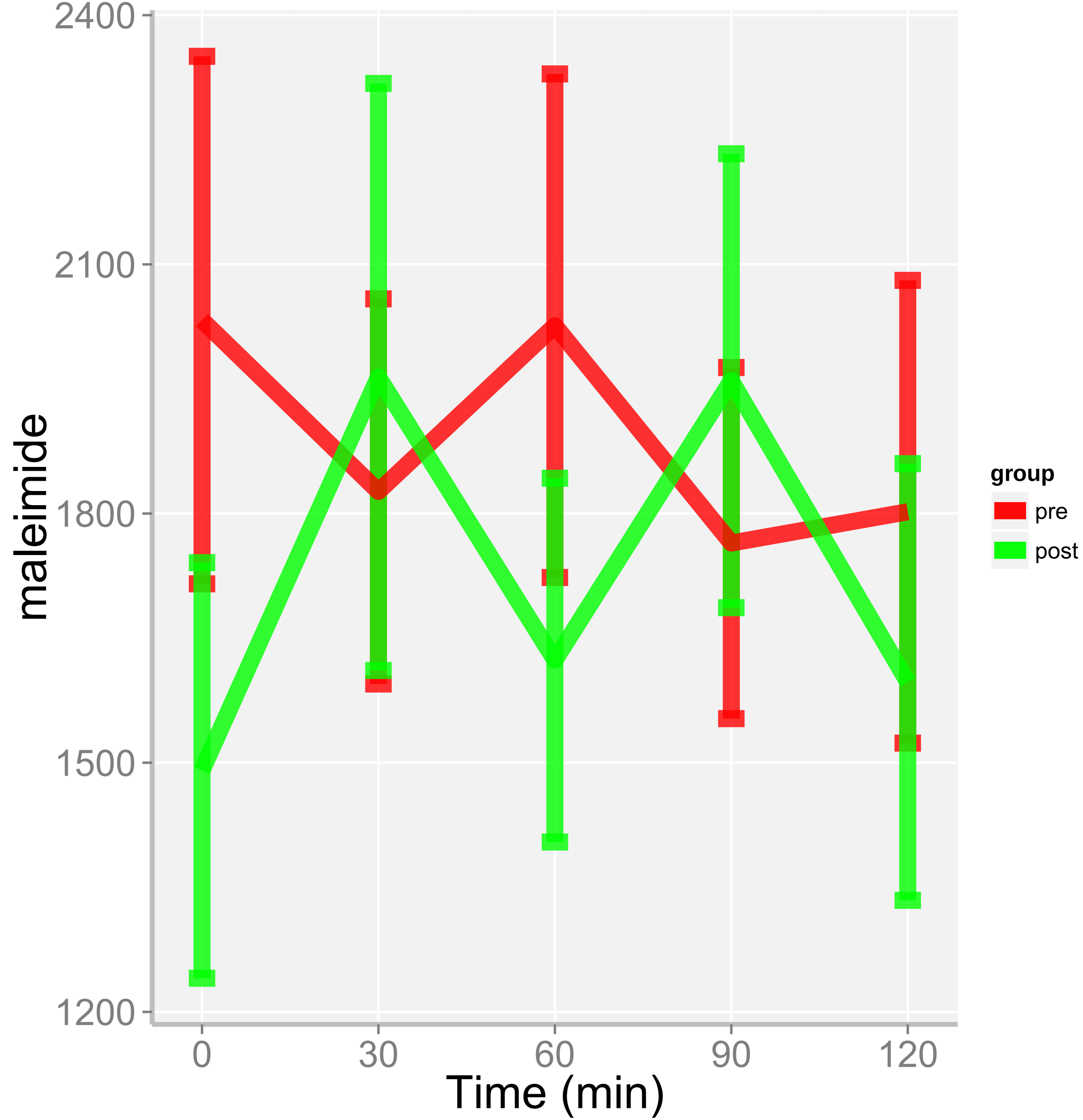

2400
2100
1800
1500
1200
maleimide
0
30
60
90
120
Time (min)
group
pre
post

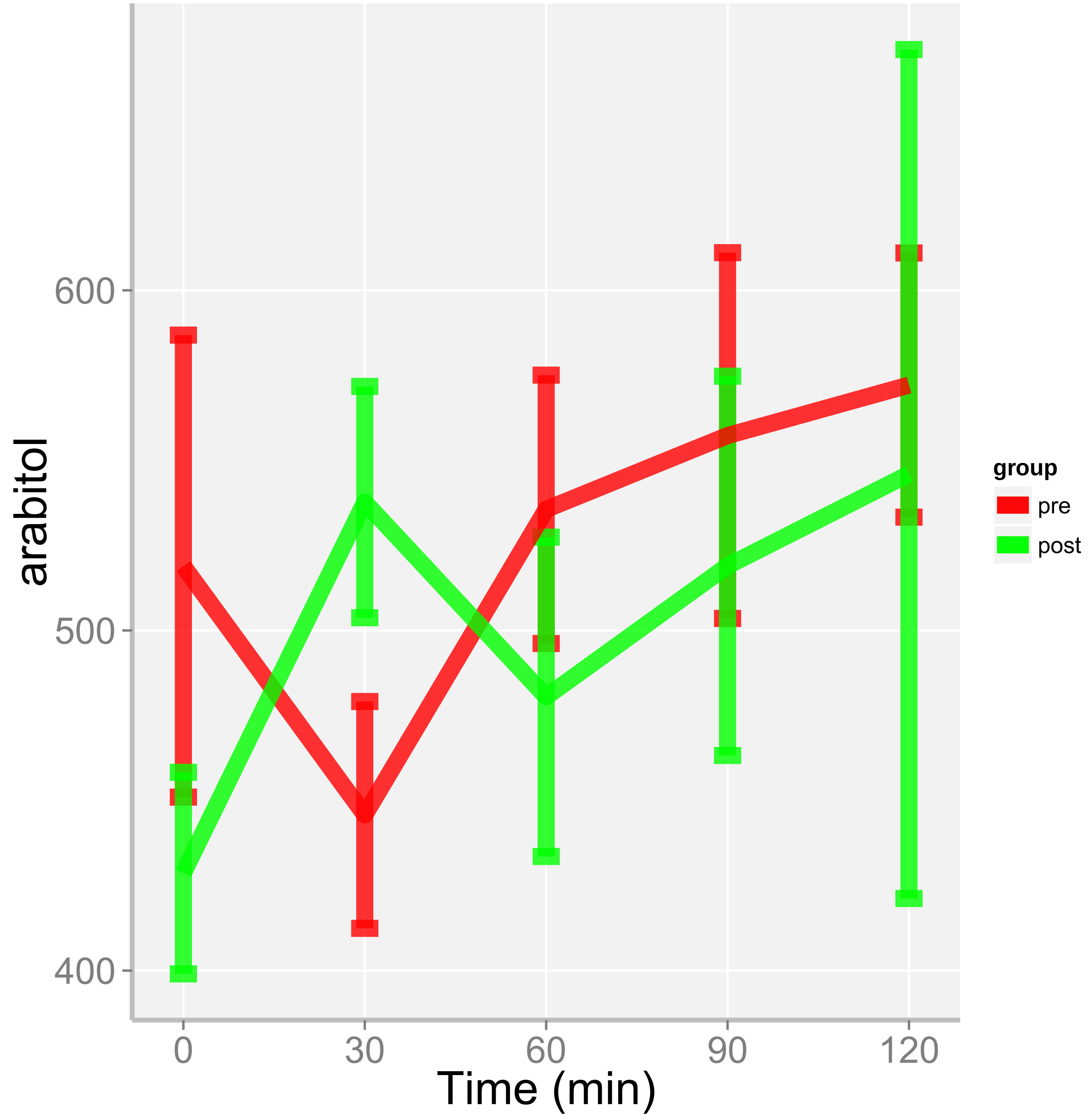

arabitol
600
500
400
0
30
60
90
120
Time (min)
group
pre
post

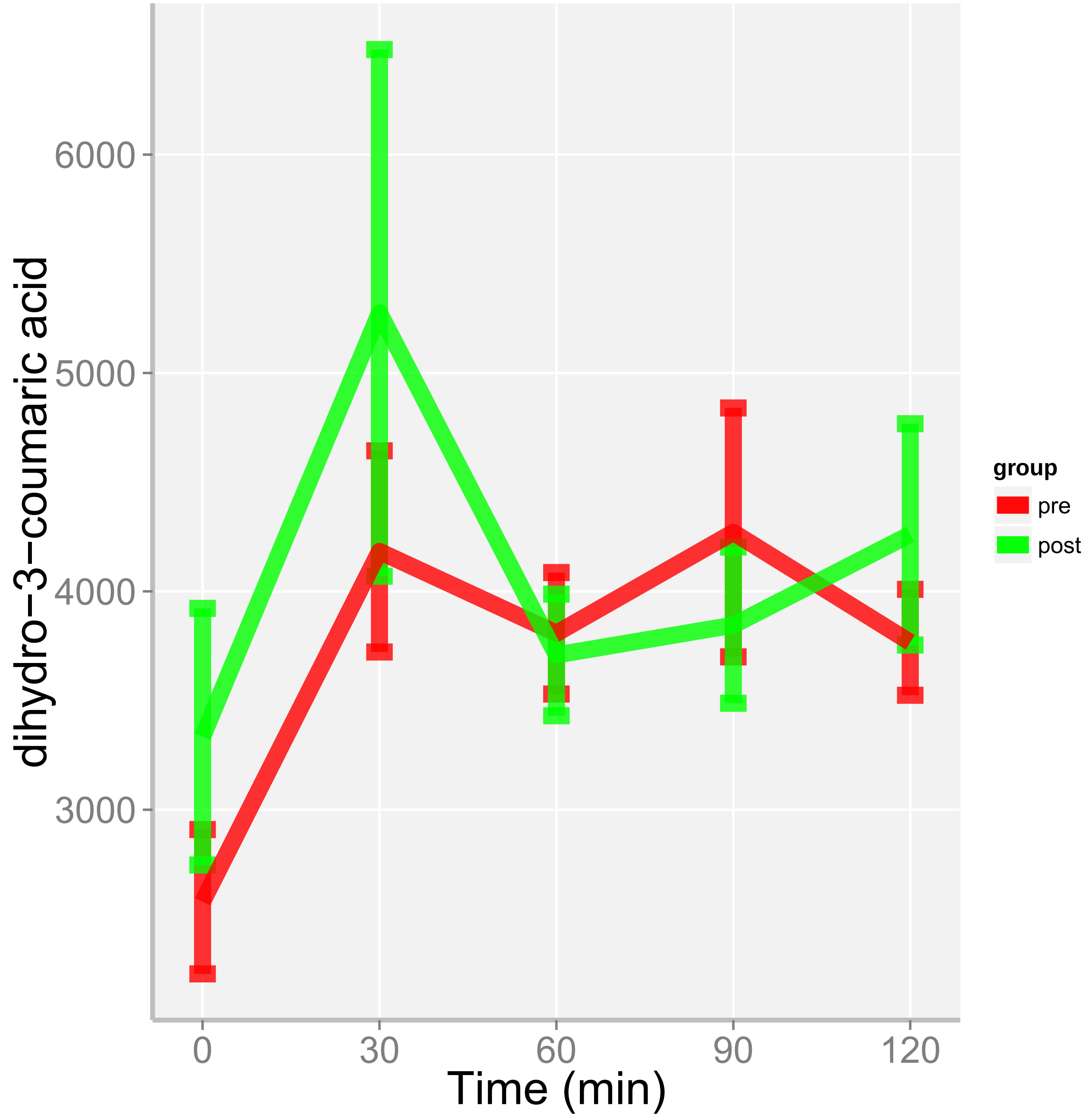

dihydro−3−coumaric acid
6000
5000
4000
3000
0
30
60
90
120
Time (min)
group
pre
post

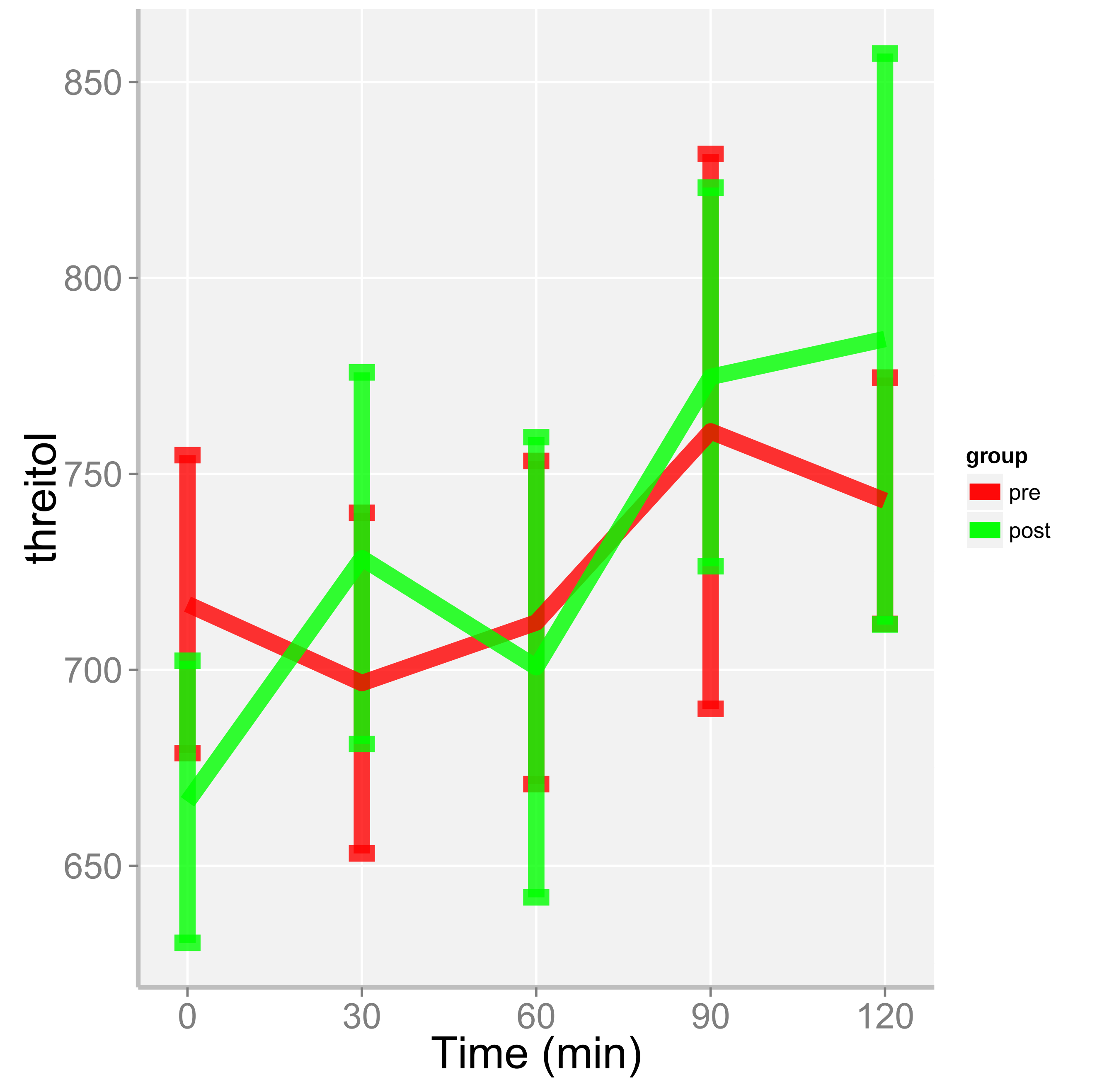

threitol
850
800
750
700
650
0
30
60
90
120
Time (min)
group
pre
post

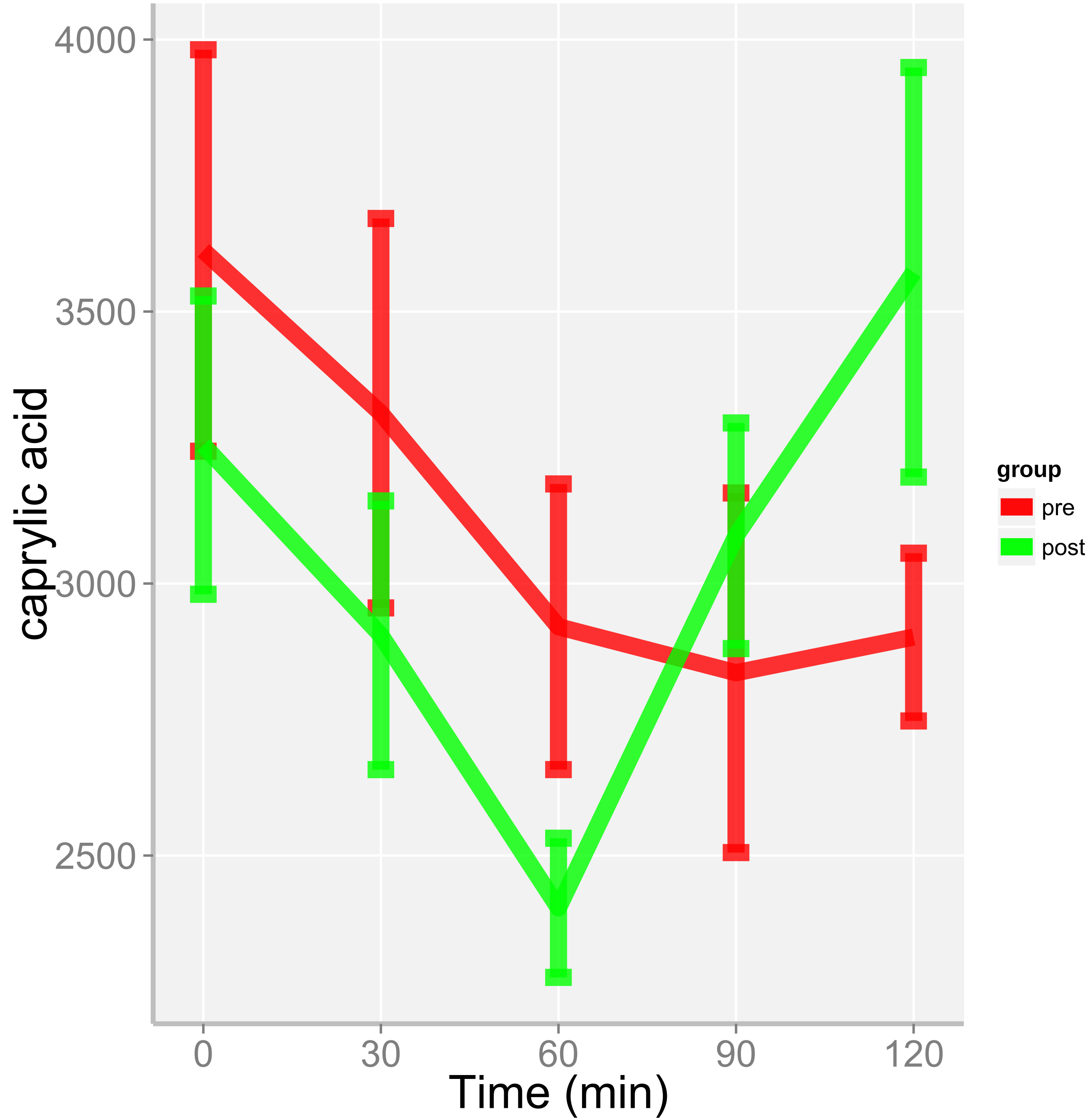

caprylic acid
4000
3500
3000
2500
0
30
60
90
120
Time (min)
group
pre
post

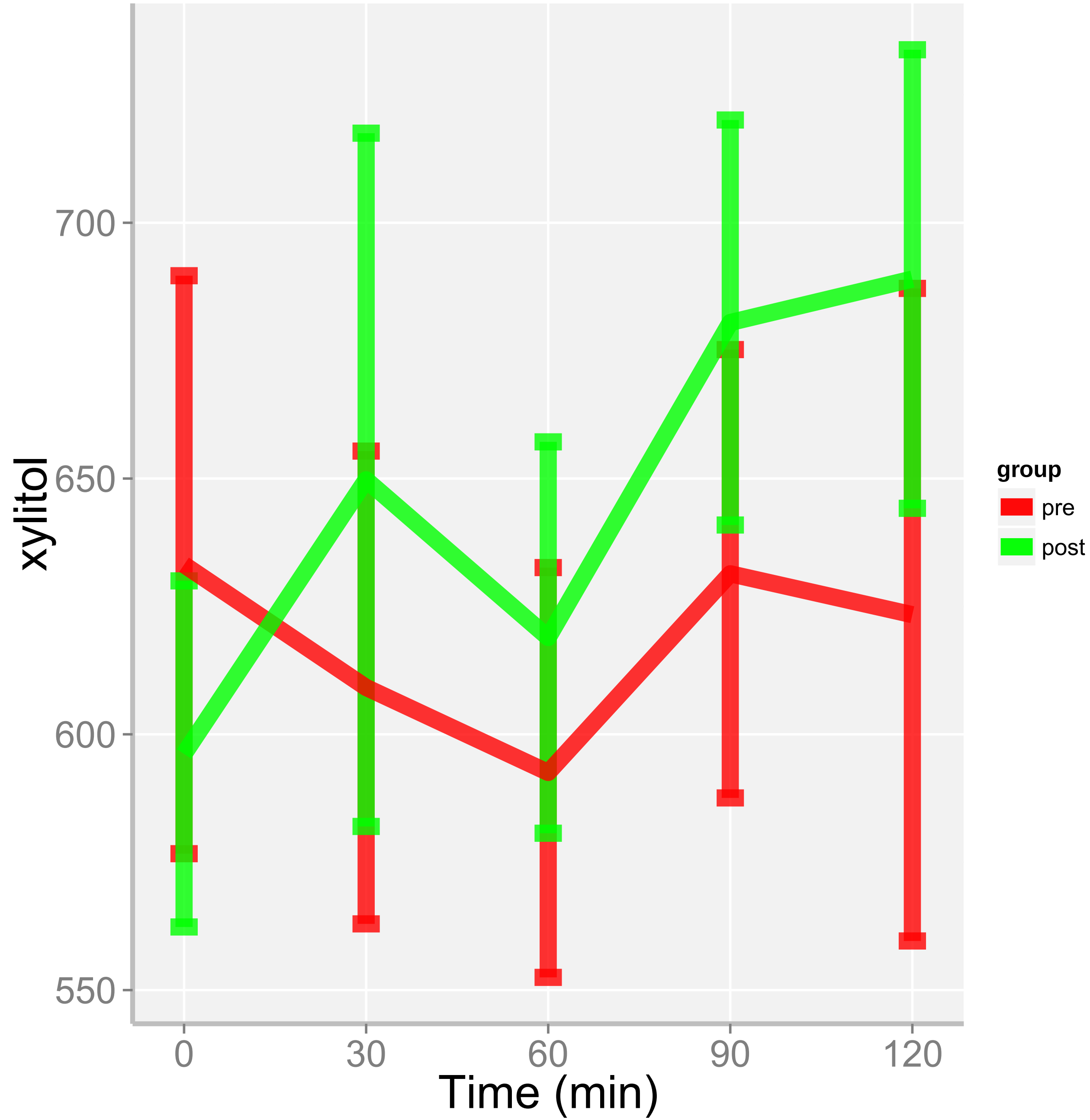

xylitol
700
650
600
550
0
30
60
90
120
Time (min)
group
pre
post

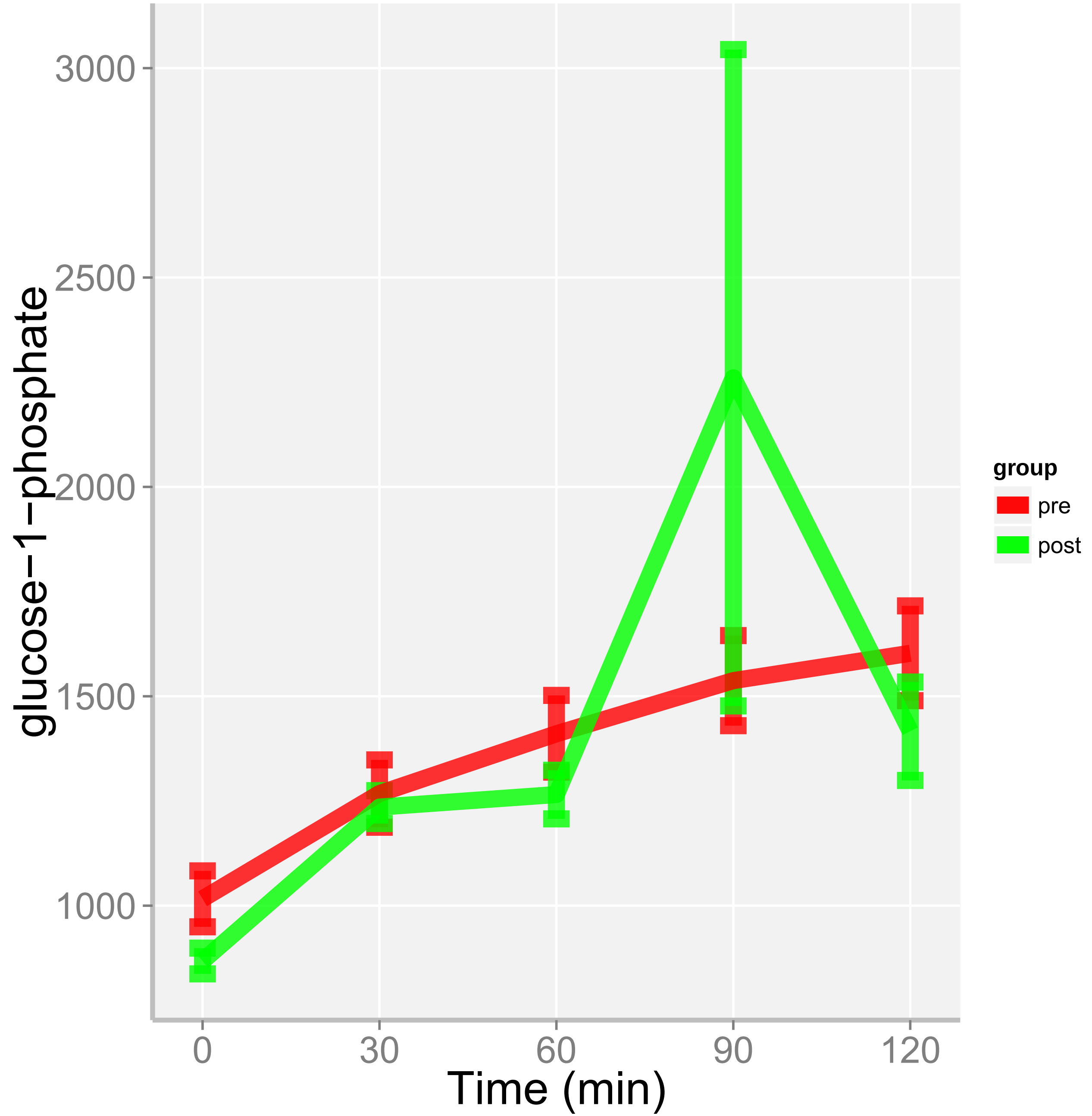

glucose−1−phosphate
3000
2500
2000
1500
1000
0
30
60
90
120
Time (min)
group
pre
post

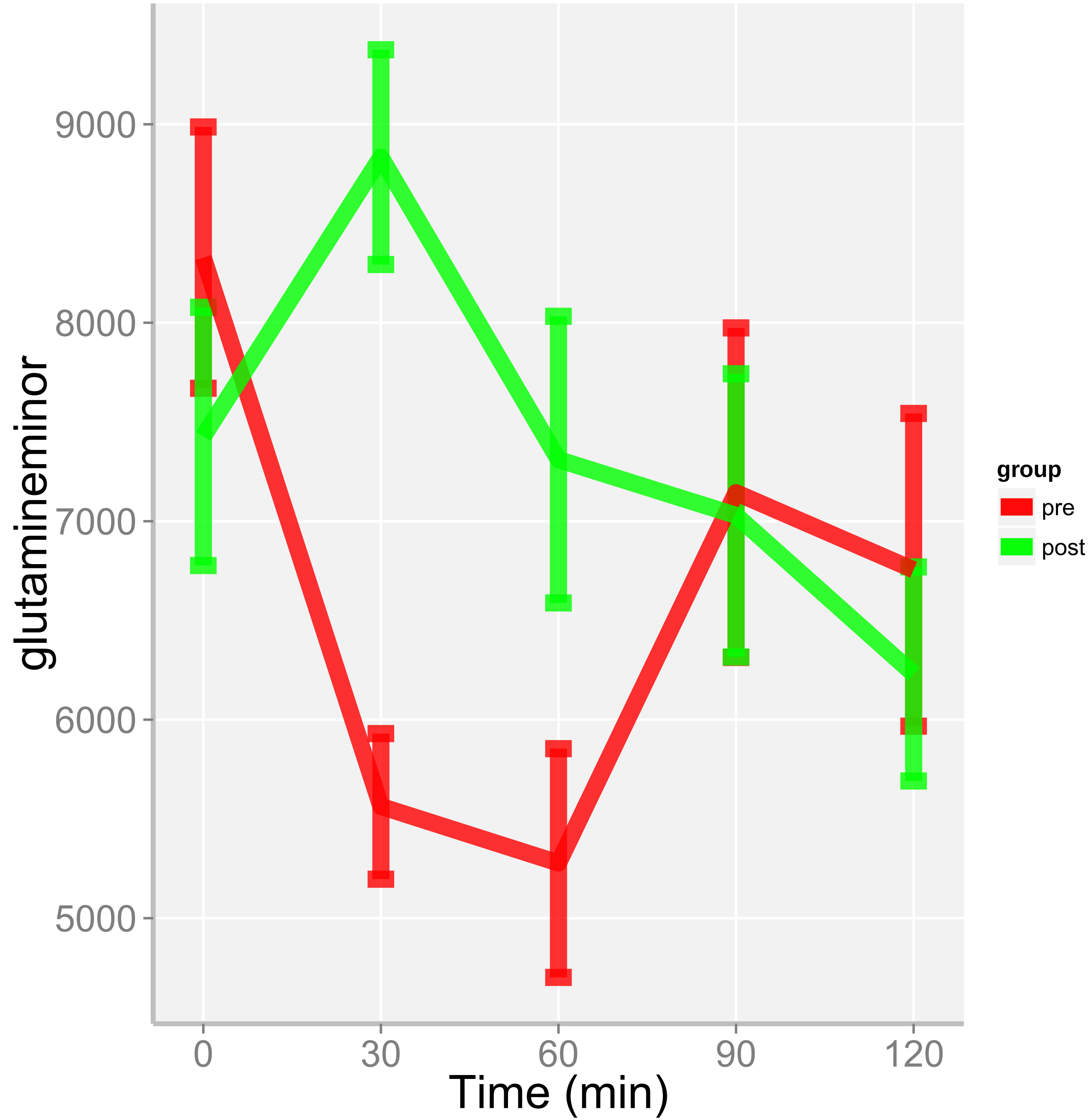

glutamineminor
9000
8000
7000
6000
5000
0
30
60
90
120
Time (min)
group
pre
post

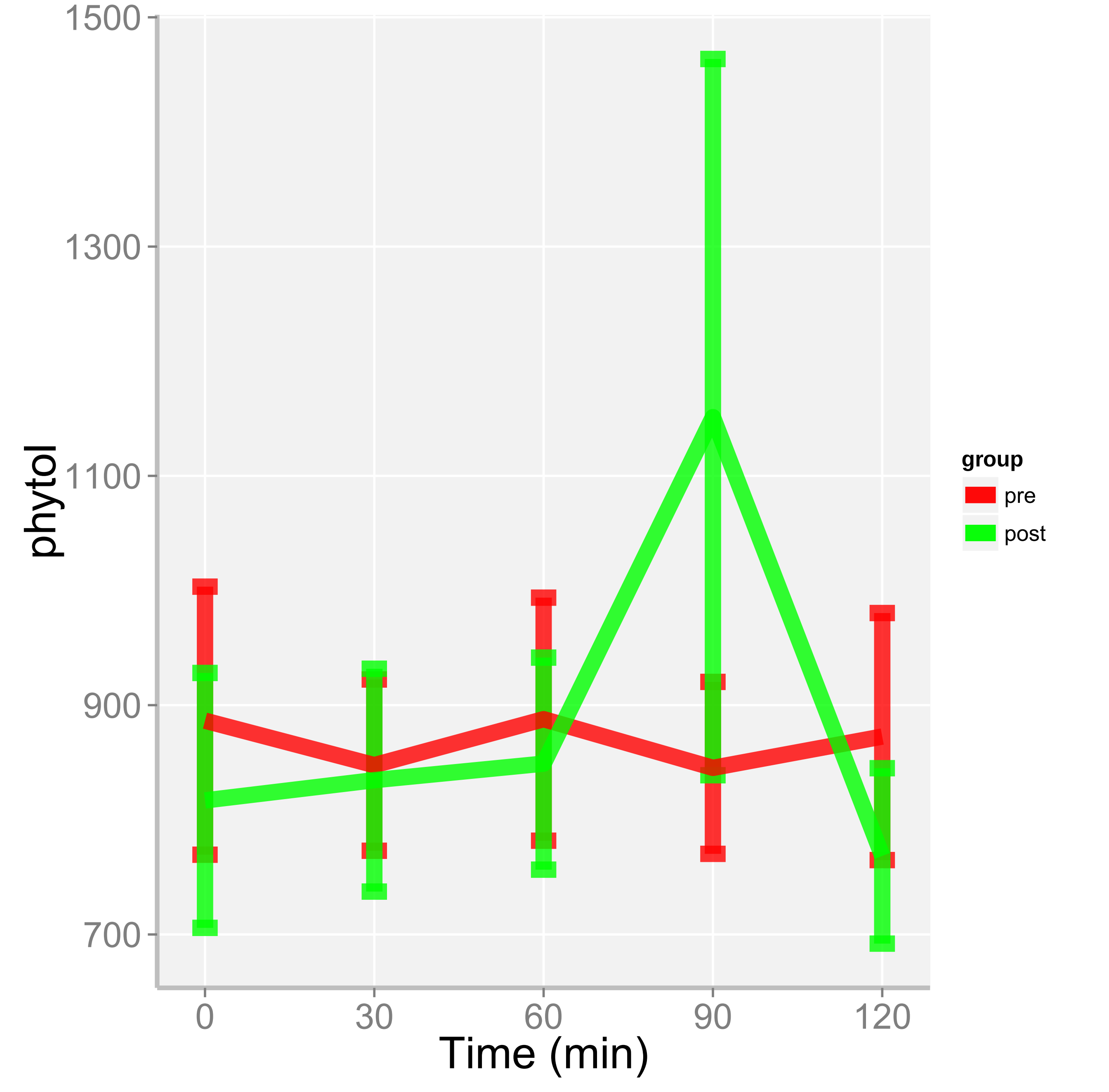

phytol
1500
1300
1100
900
700
0
30
60
90
120
Time (min)
group
pre
post

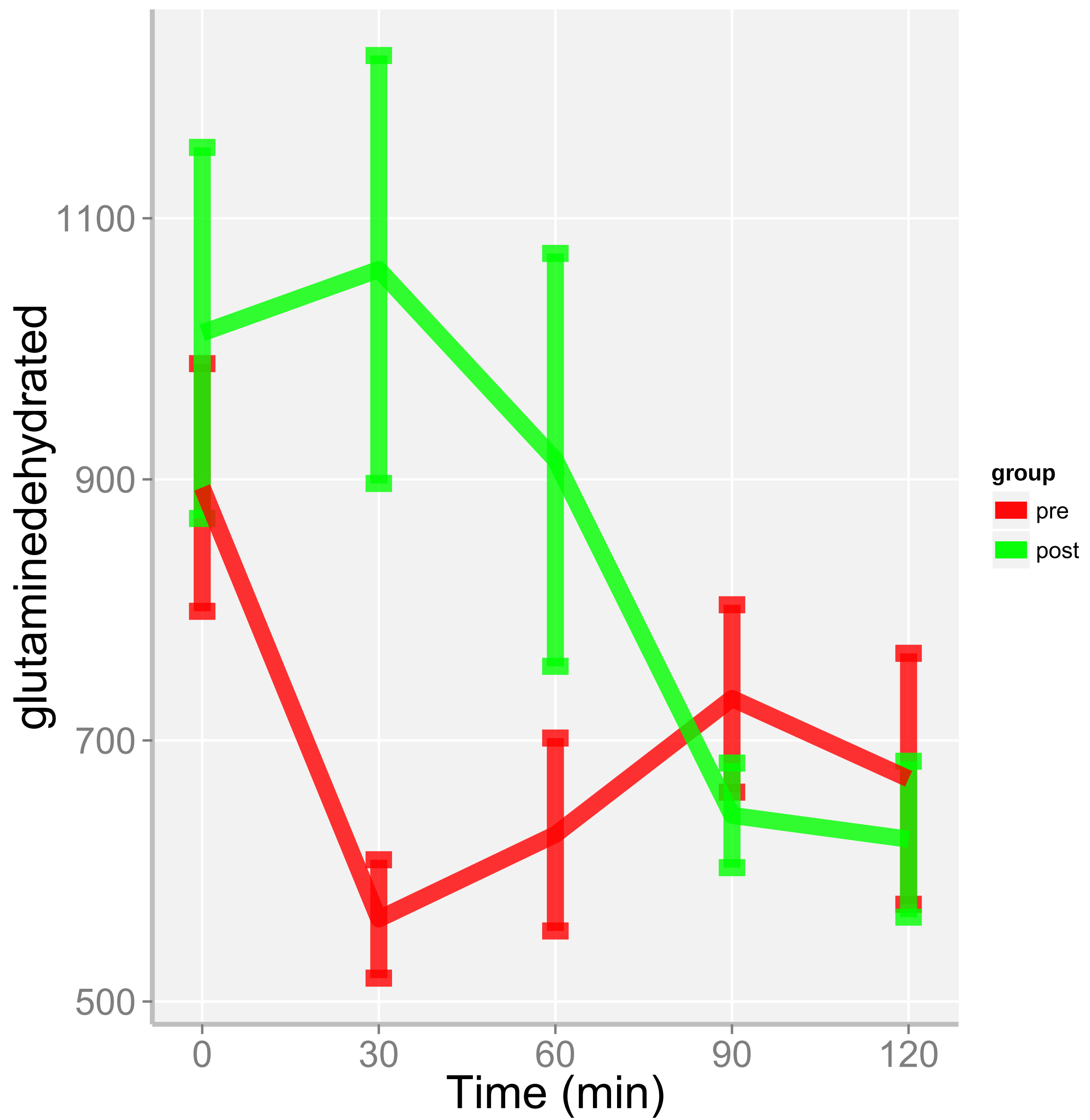

glutaminedehydrated
1100
900
700
500
0
30
60
90
120
Time (min)
group
pre
post

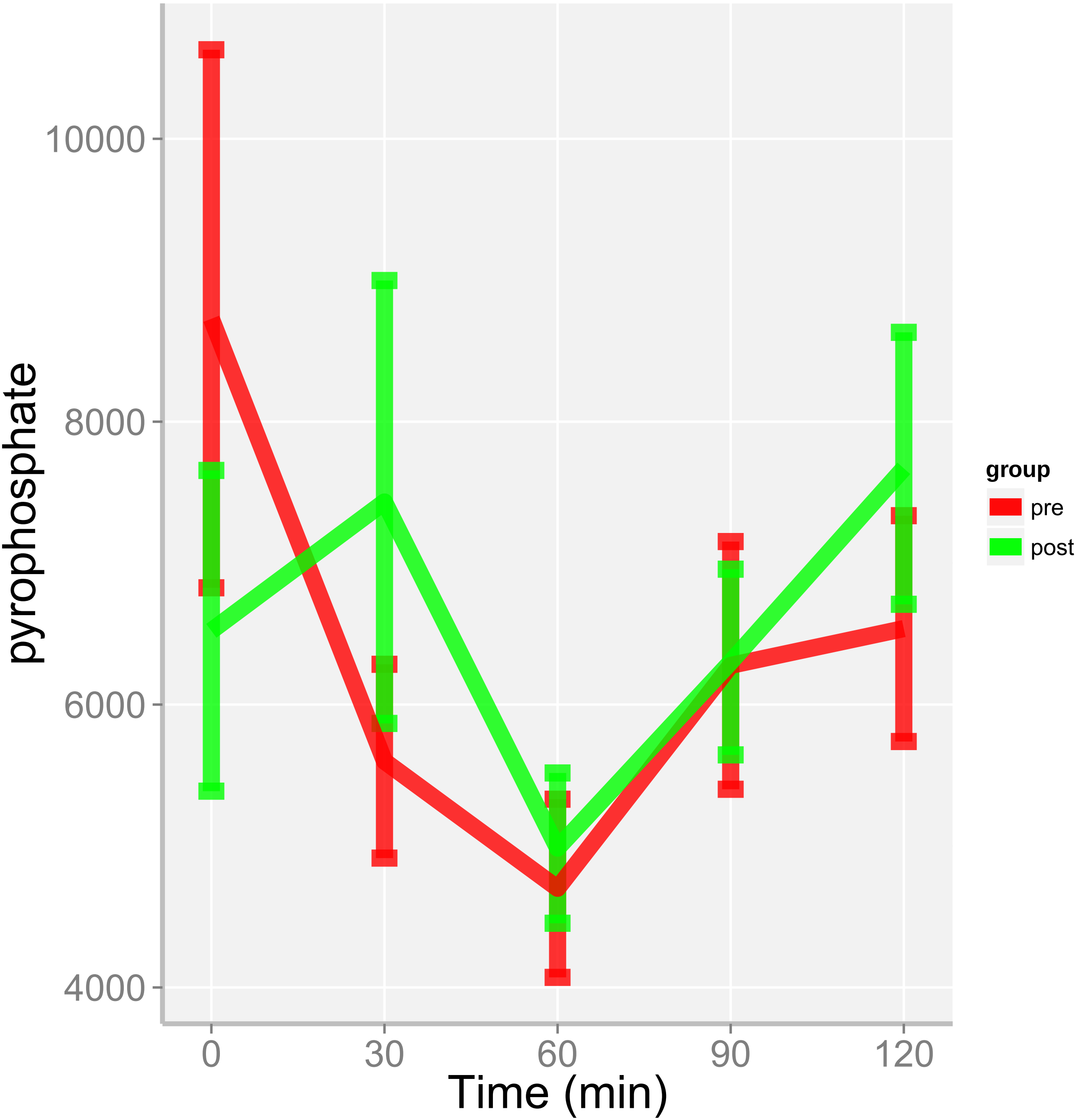

pyrophosphate
10000
8000
6000
4000
0
30
60
90
120
Time (min)
group
pre
post

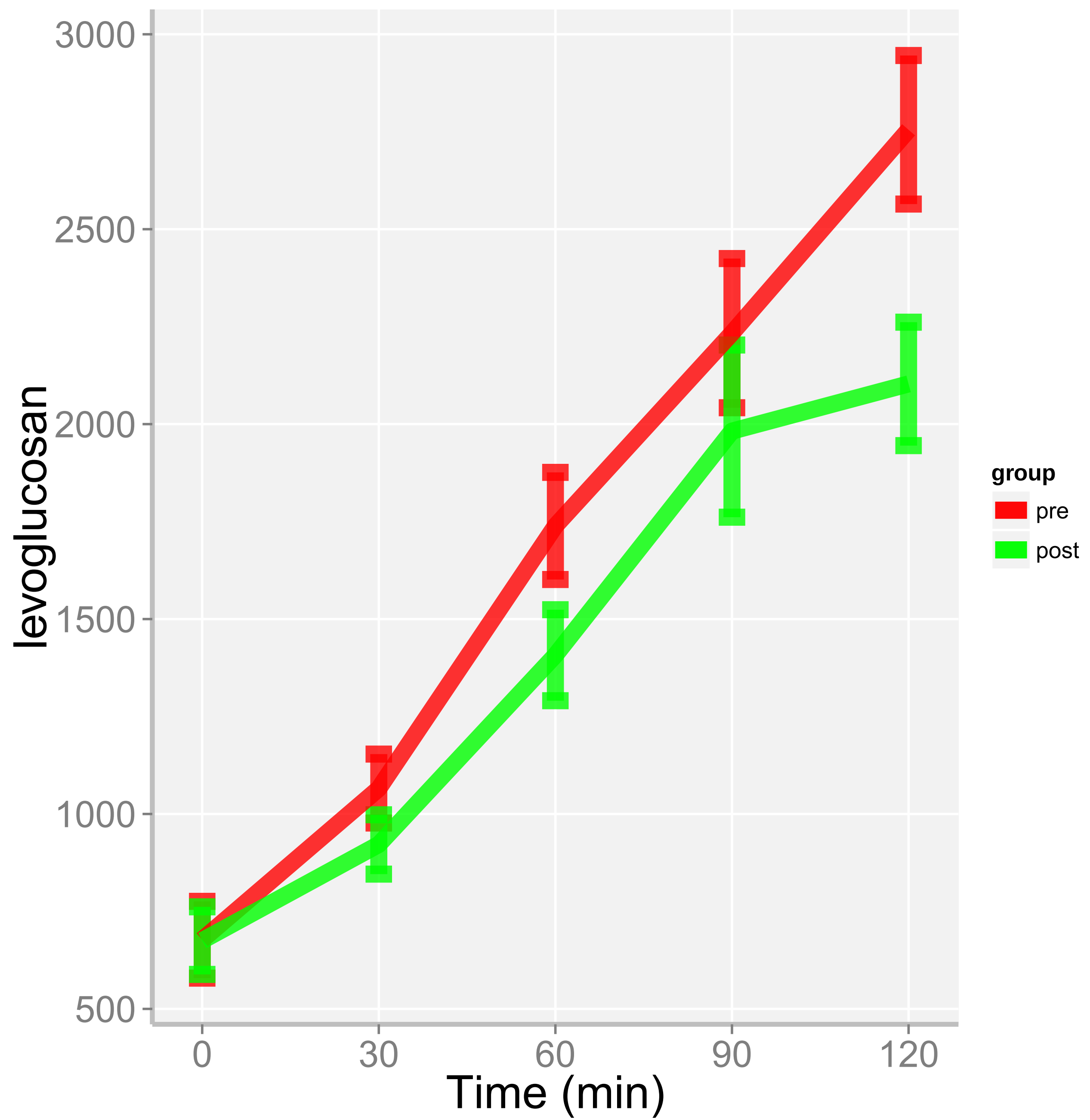

levoglucosan
3000
2500
2000
1500
1000
500
0
30
60
90
120
Time (min)
group
pre
post

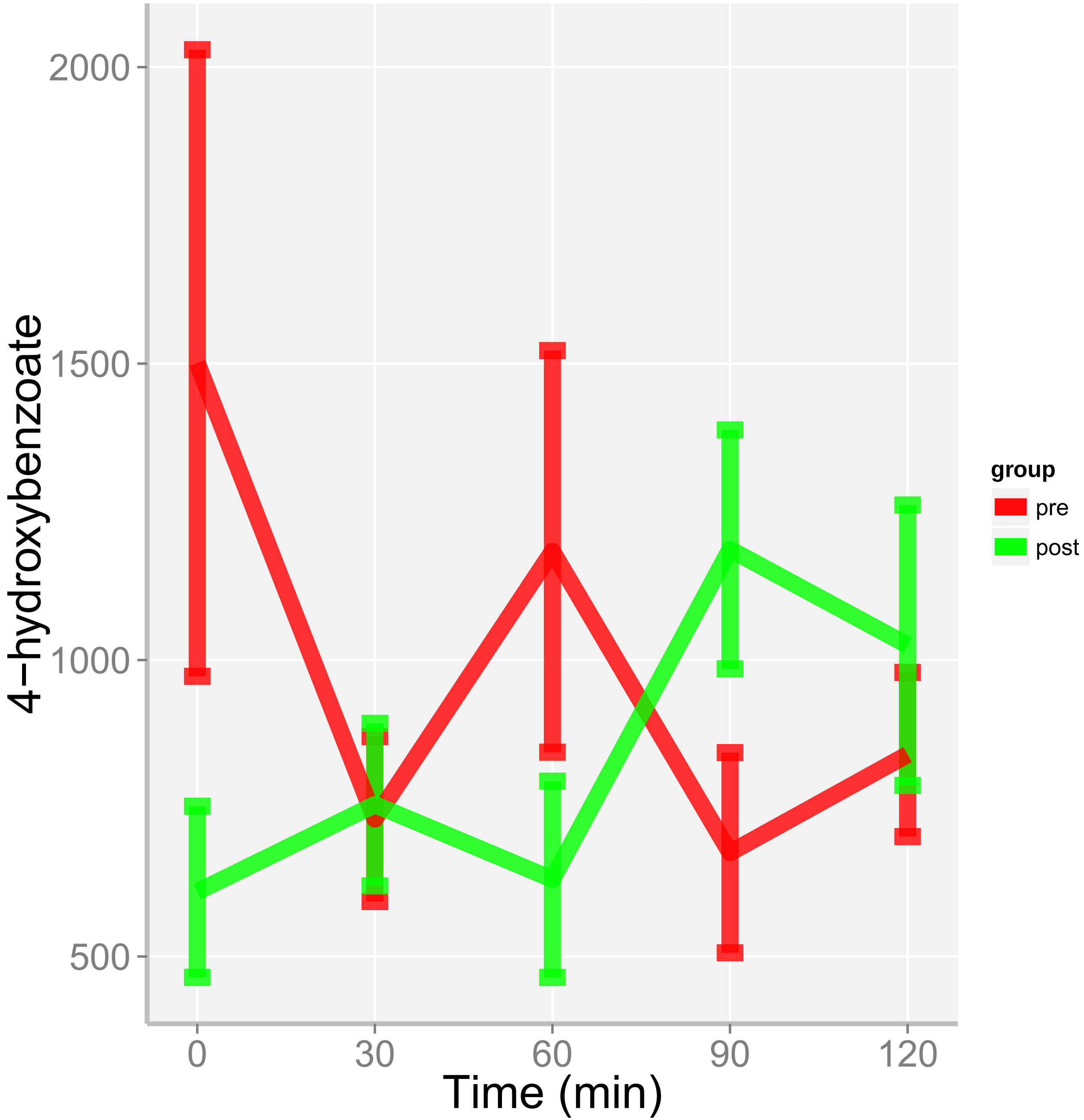

2000
1500
1000
500
4−hydroxybenzoate
0
30
60
90
120
Time (min)
group
pre
post

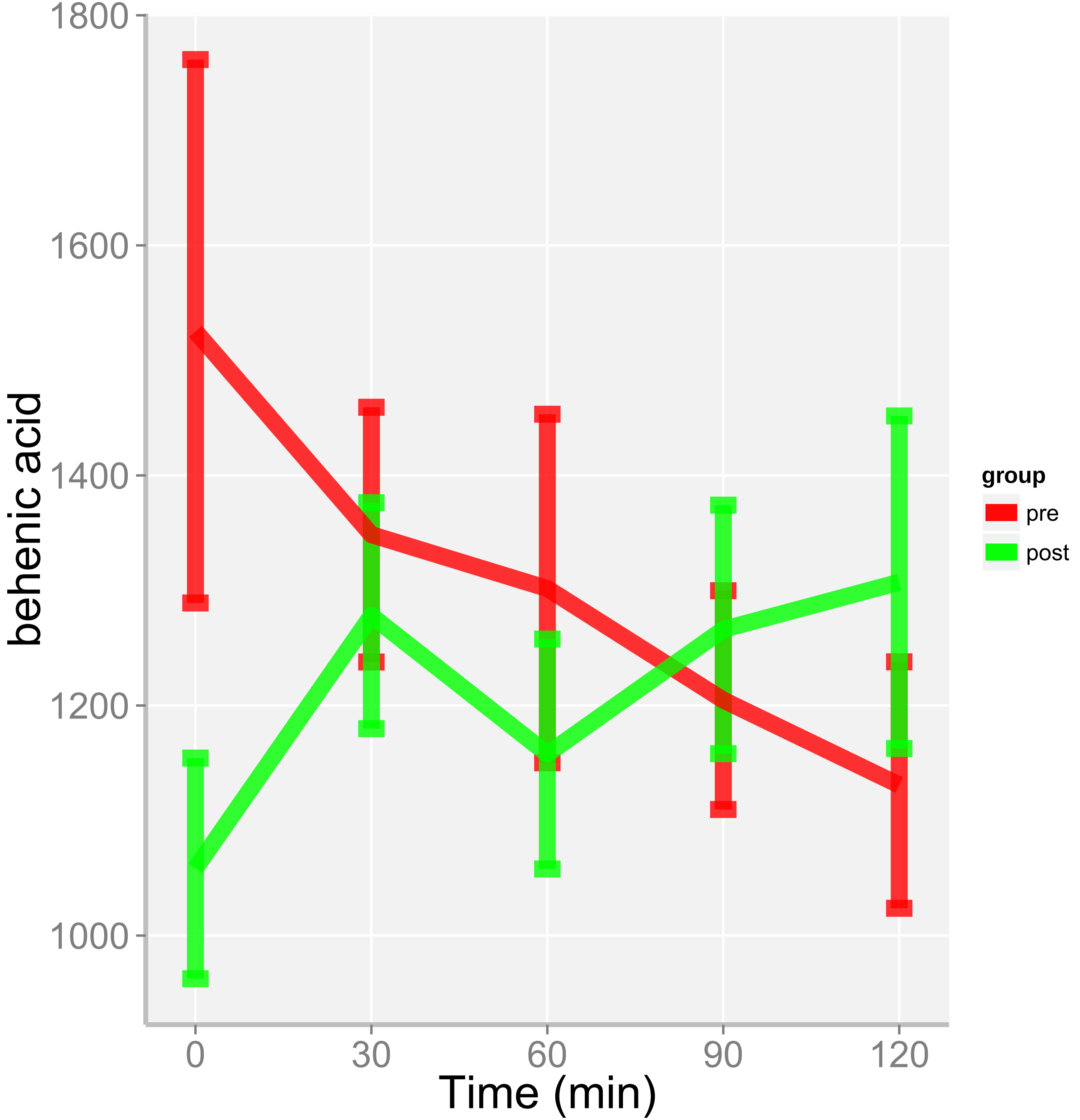

1800
1600
1400
1200
1000
behenic acid
0
30
60
90
120
Time (min)
group
pre
post

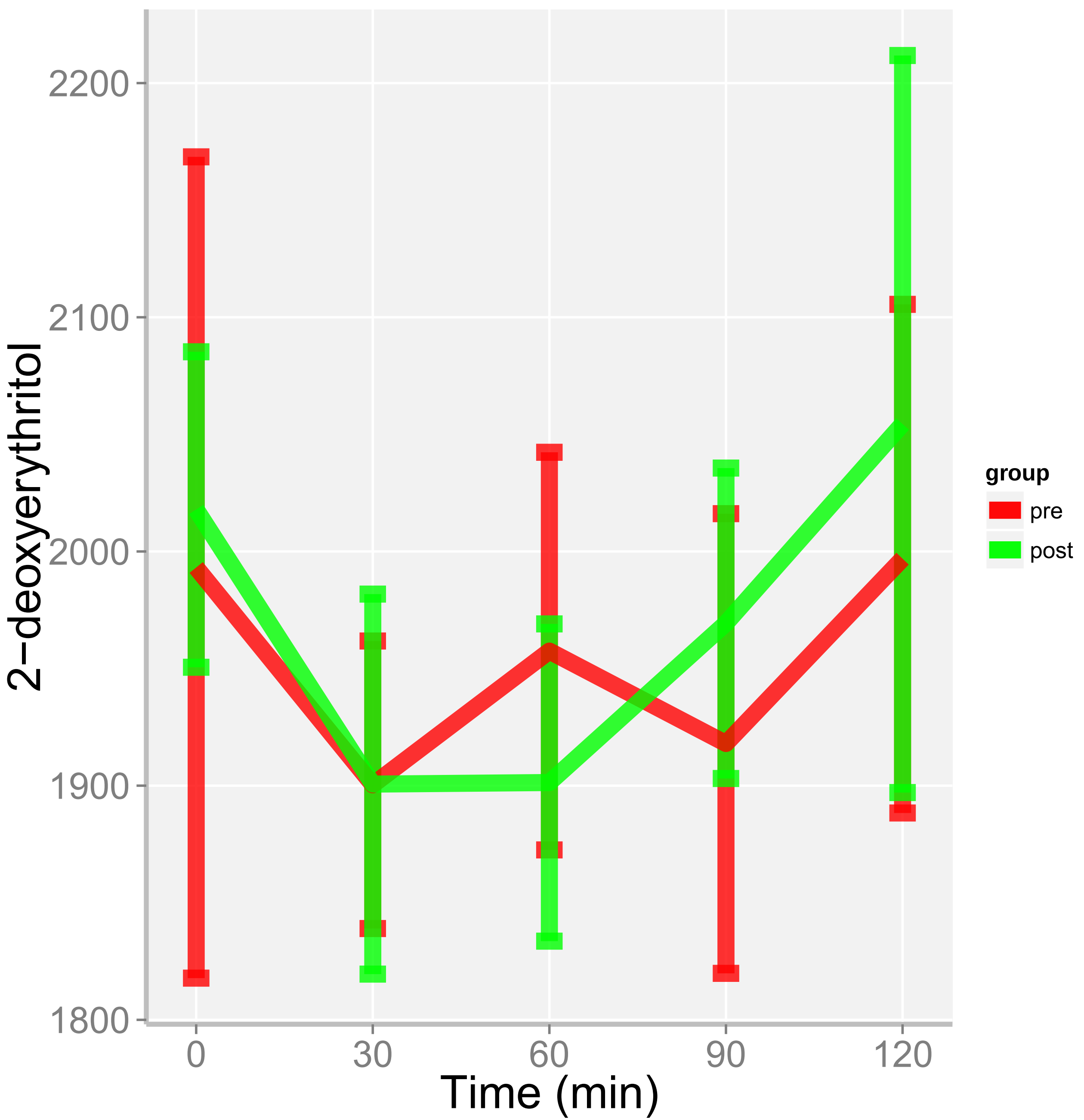

2−deoxyerythritol
2200
2100
2000
1900
1800
0
30
60
90
120
Time (min)
group
pre
post

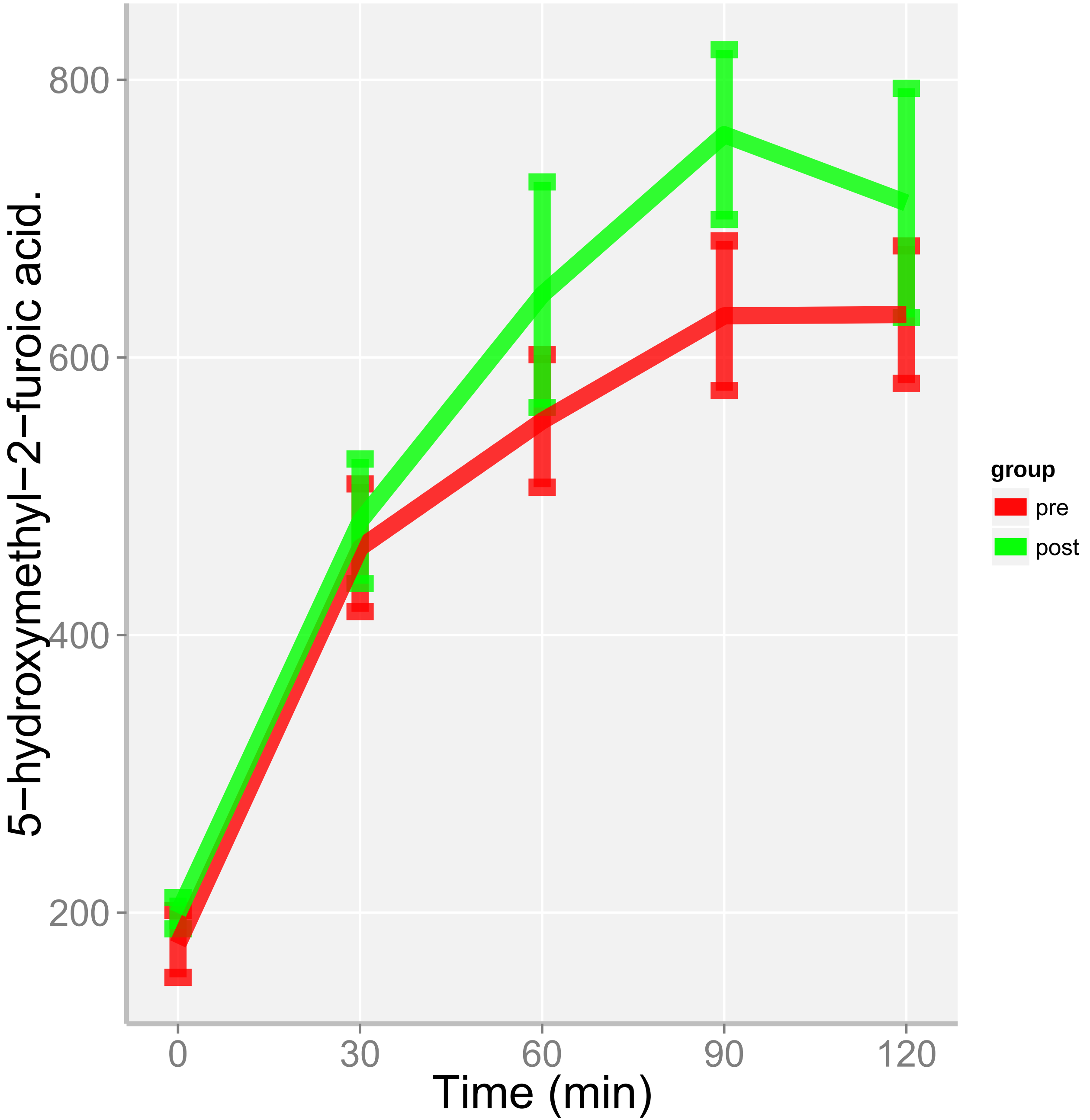

5−hydroxymethyl−2−furoic acid.
800
600
400
200
0
30
60
90
120
Time (min)
group
pre
post

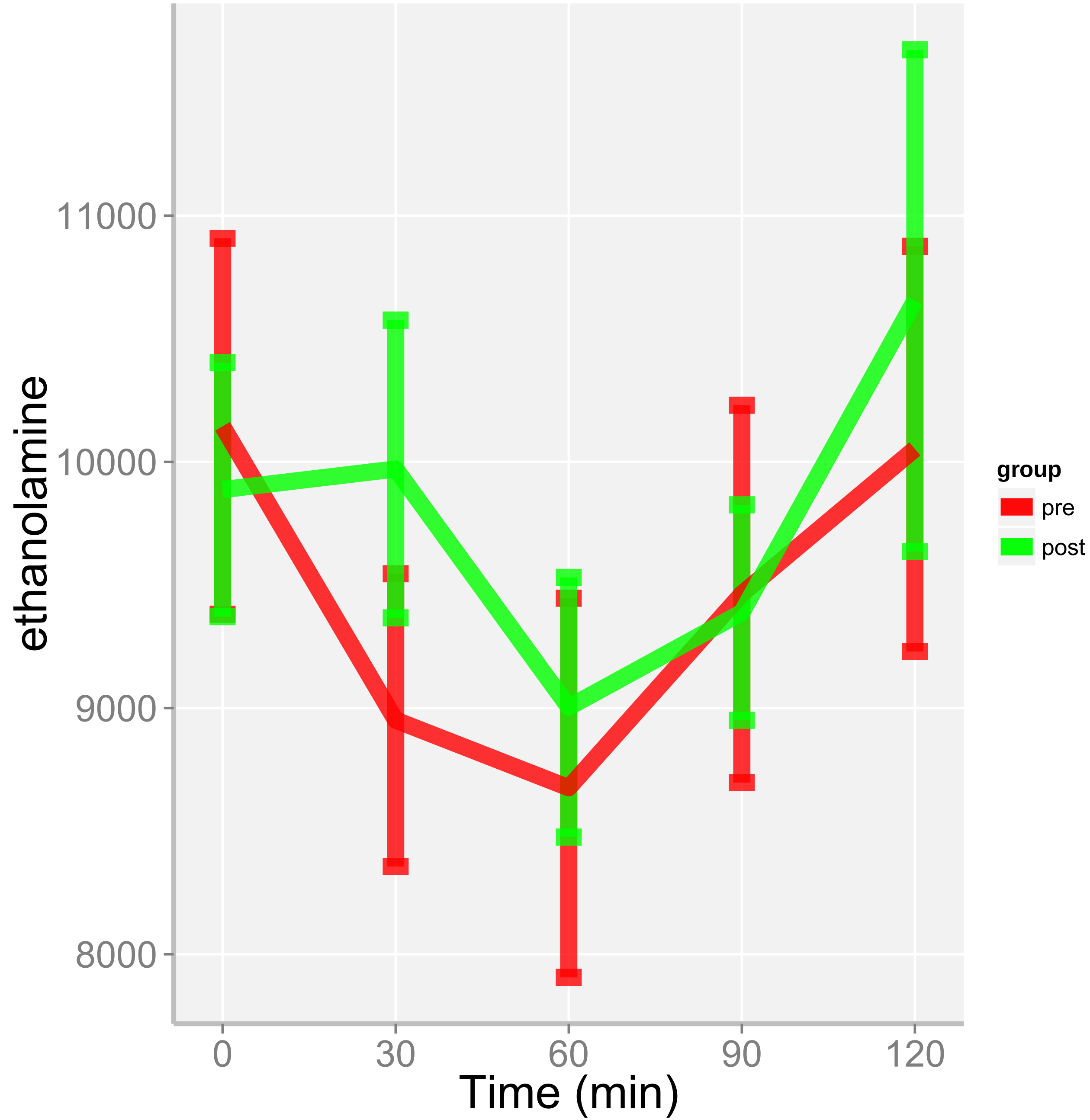

ethanolamine
11000
10000
9000
8000
0
30
60
90
120
Time (min)
group
pre
post

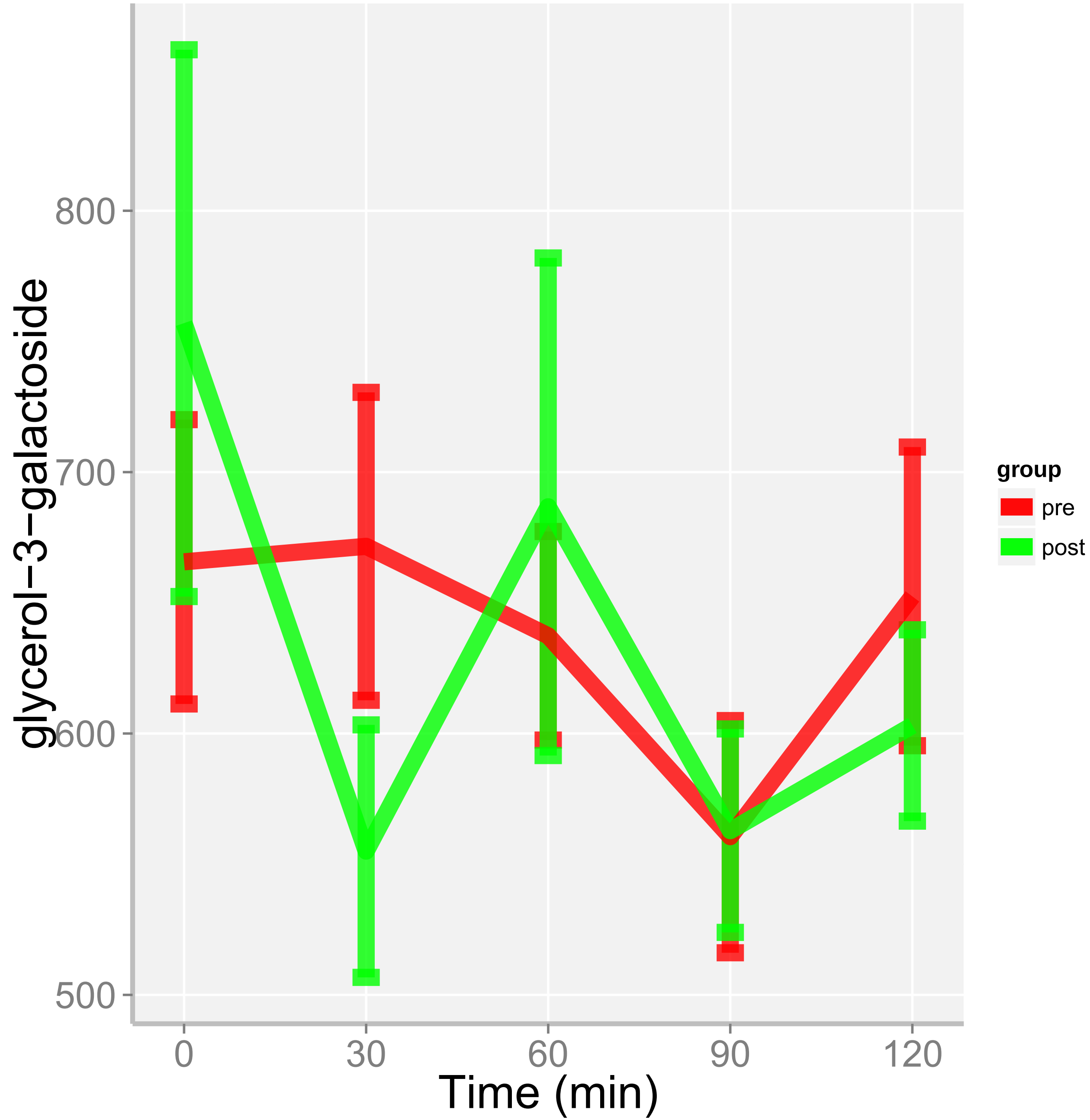

glycerol−3−galactoside
800
700
600
500
0
30
60
90
120
Time (min)
group
pre
post

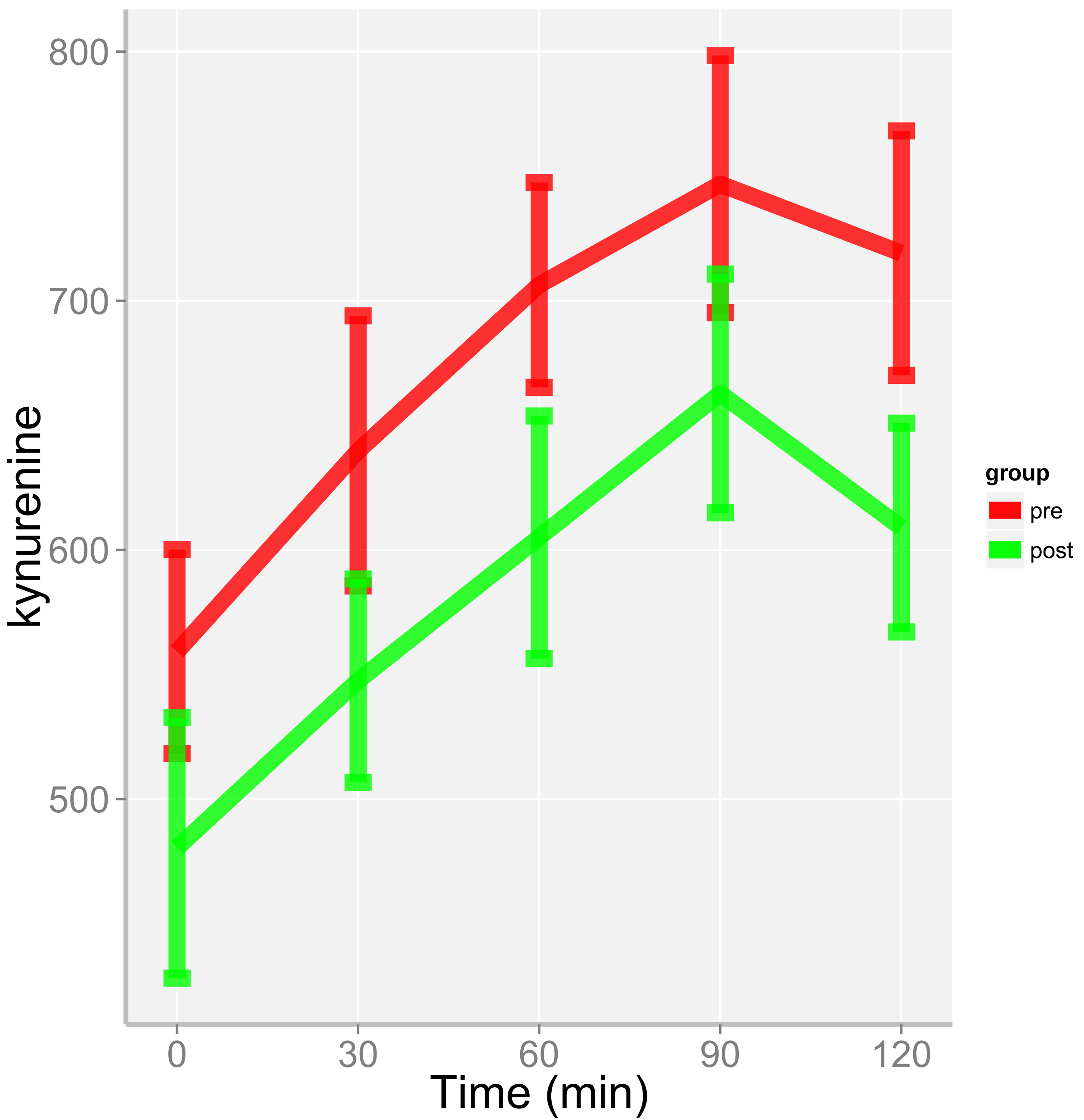

kynurenine
800
700
600
500
0
30
60
90
120
Time (min)
group
pre
post

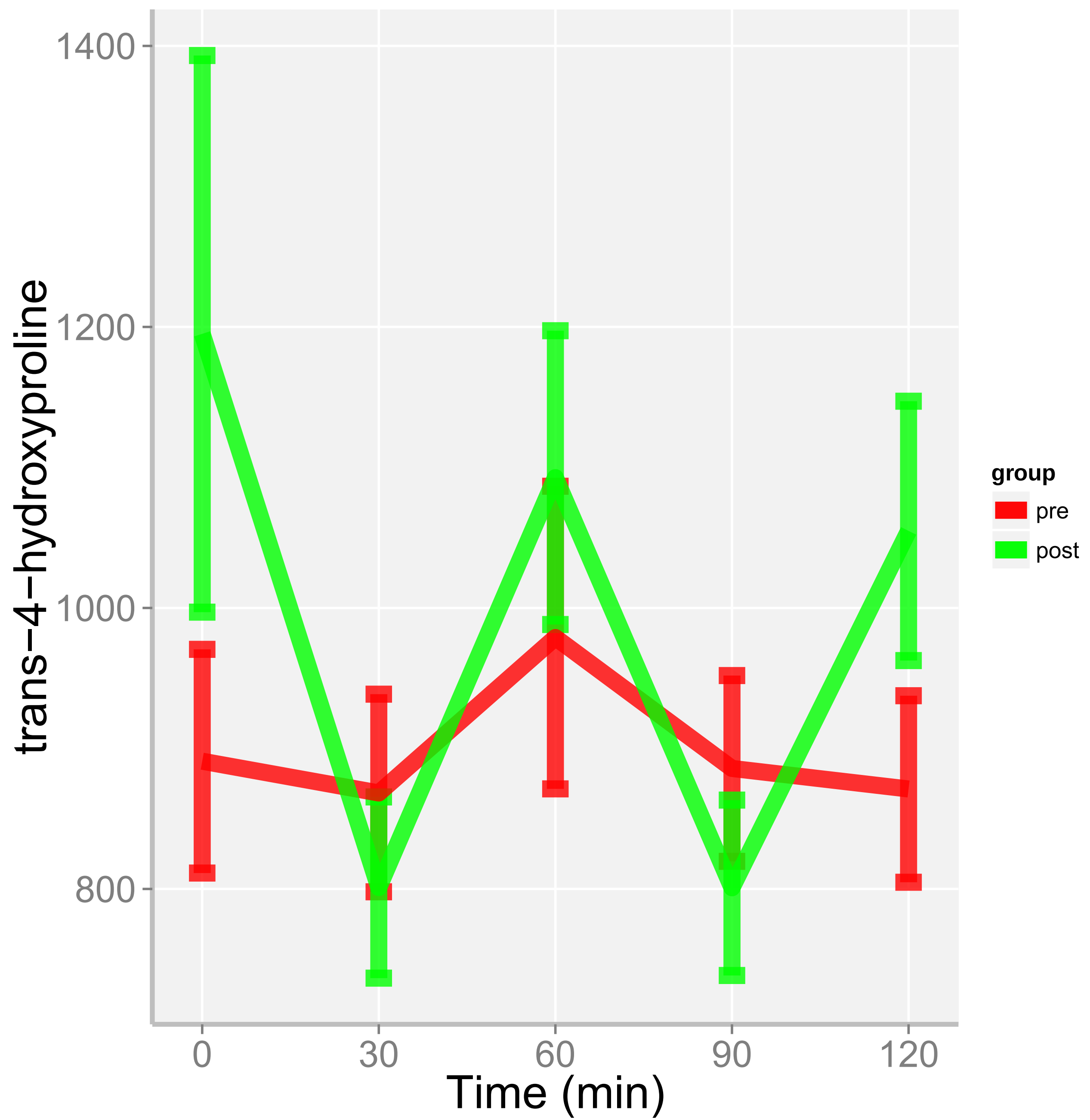

trans−4−hydroxyproline
1400
1200
1000
800
0
30
60
90
120
Time (min)
group
pre
post

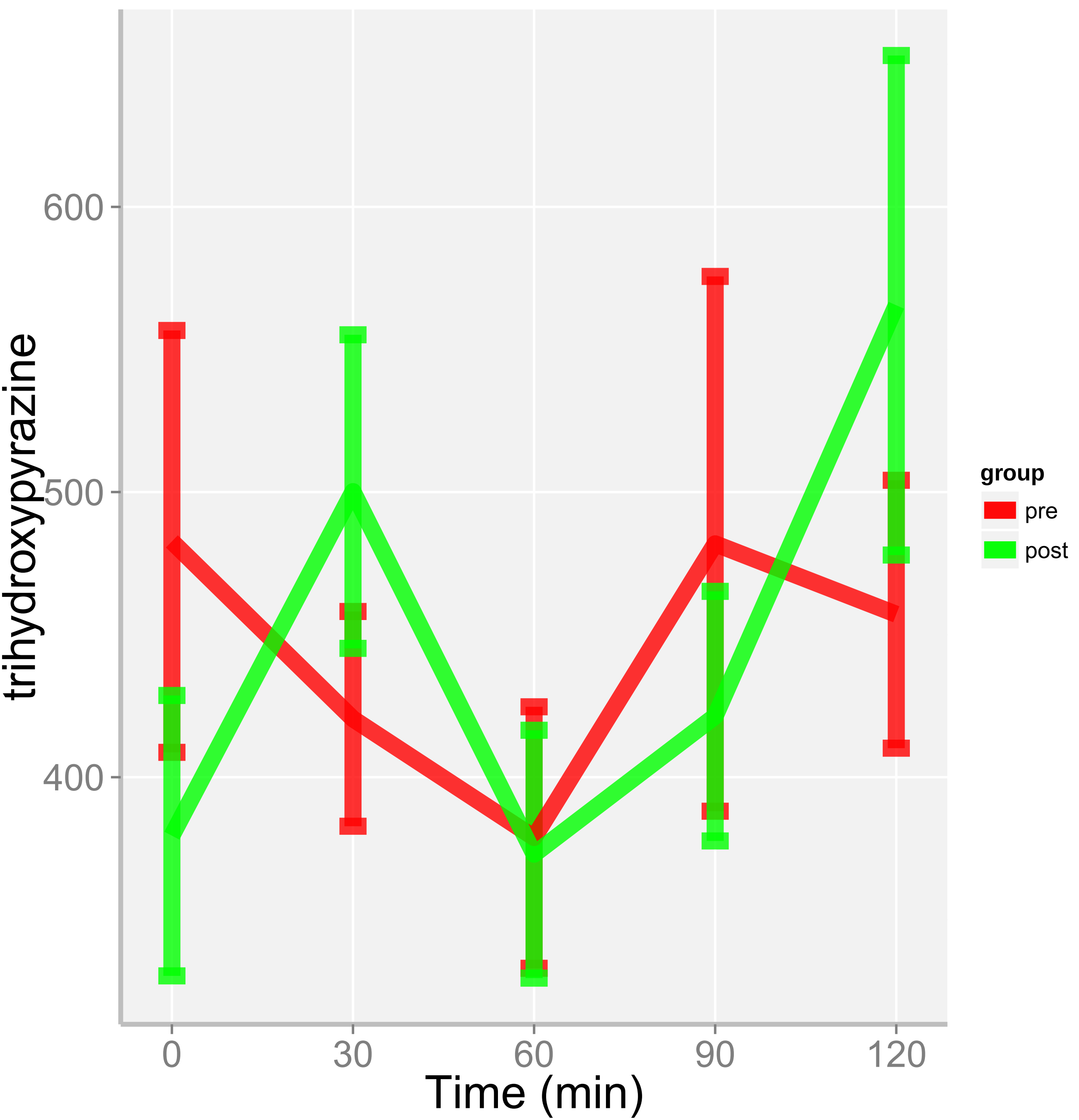

trihydroxypyrazine
600
500
400
0
30
60
90
120
Time (min)
group
pre
post

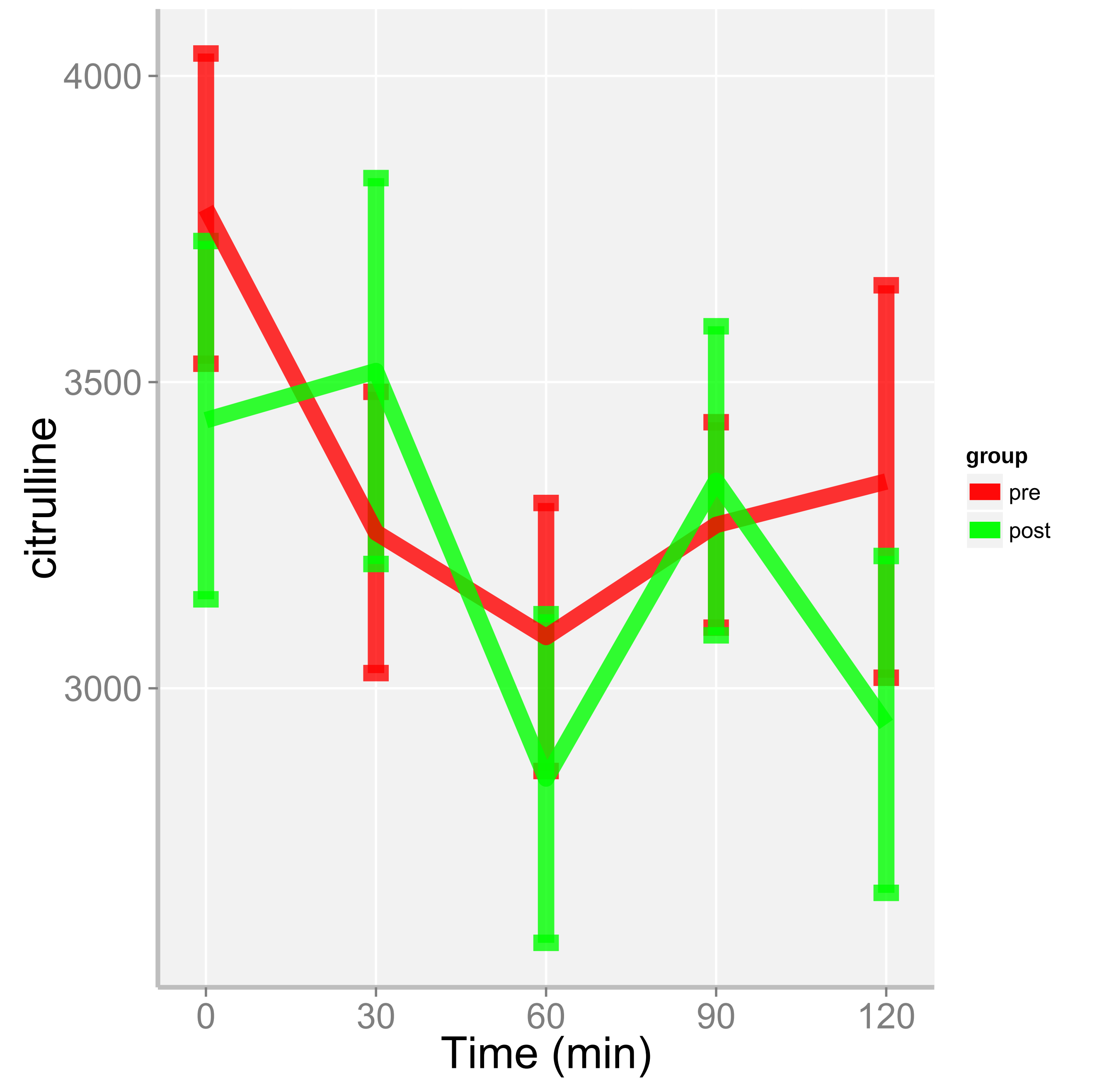

citrulline
4000
3500
3000
0
30
60
90
120
Time (min)
group
pre
post

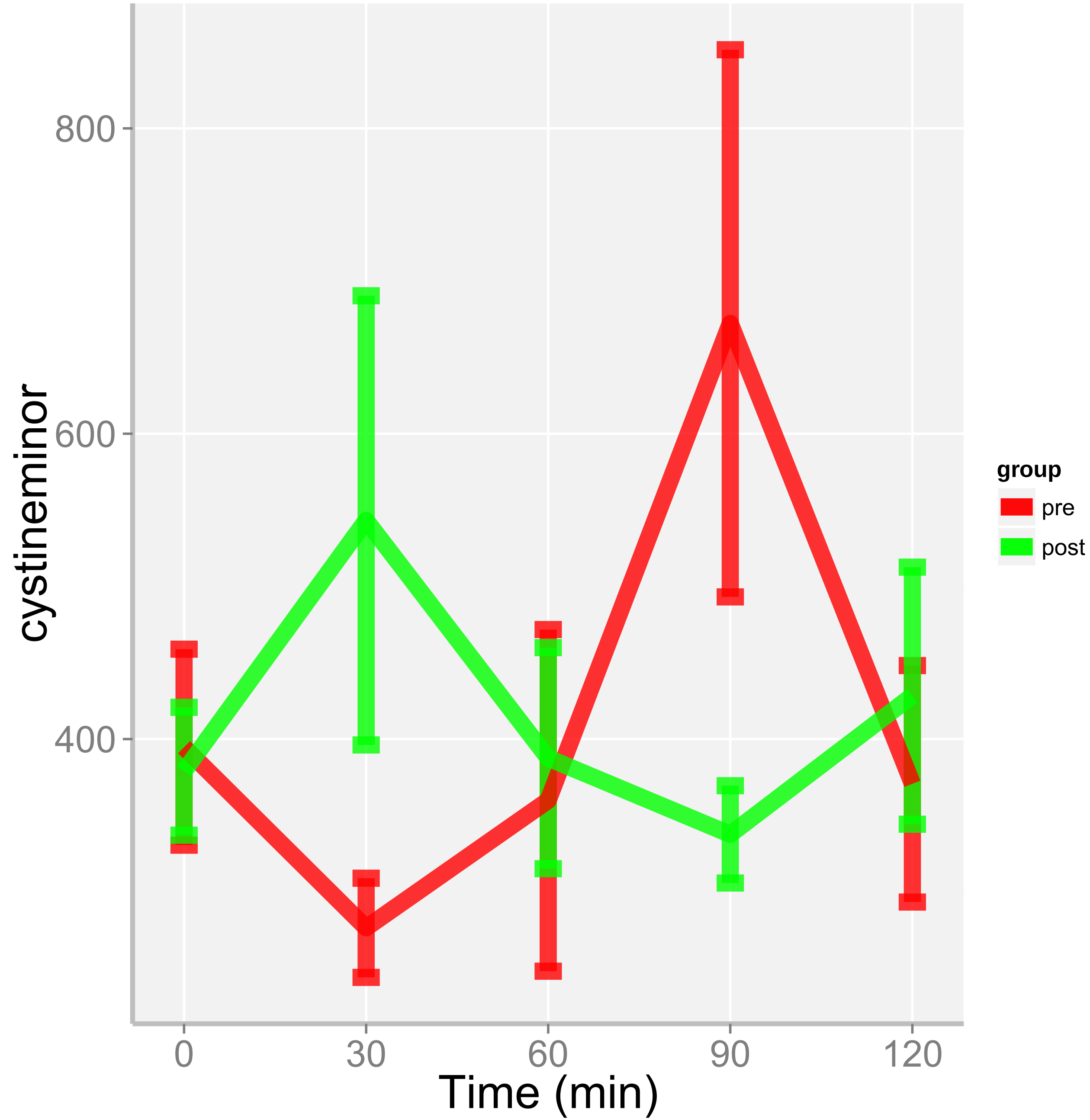

800
600
400
cystineminor
0
30
60
90
120
Time (min)
group
pre
post

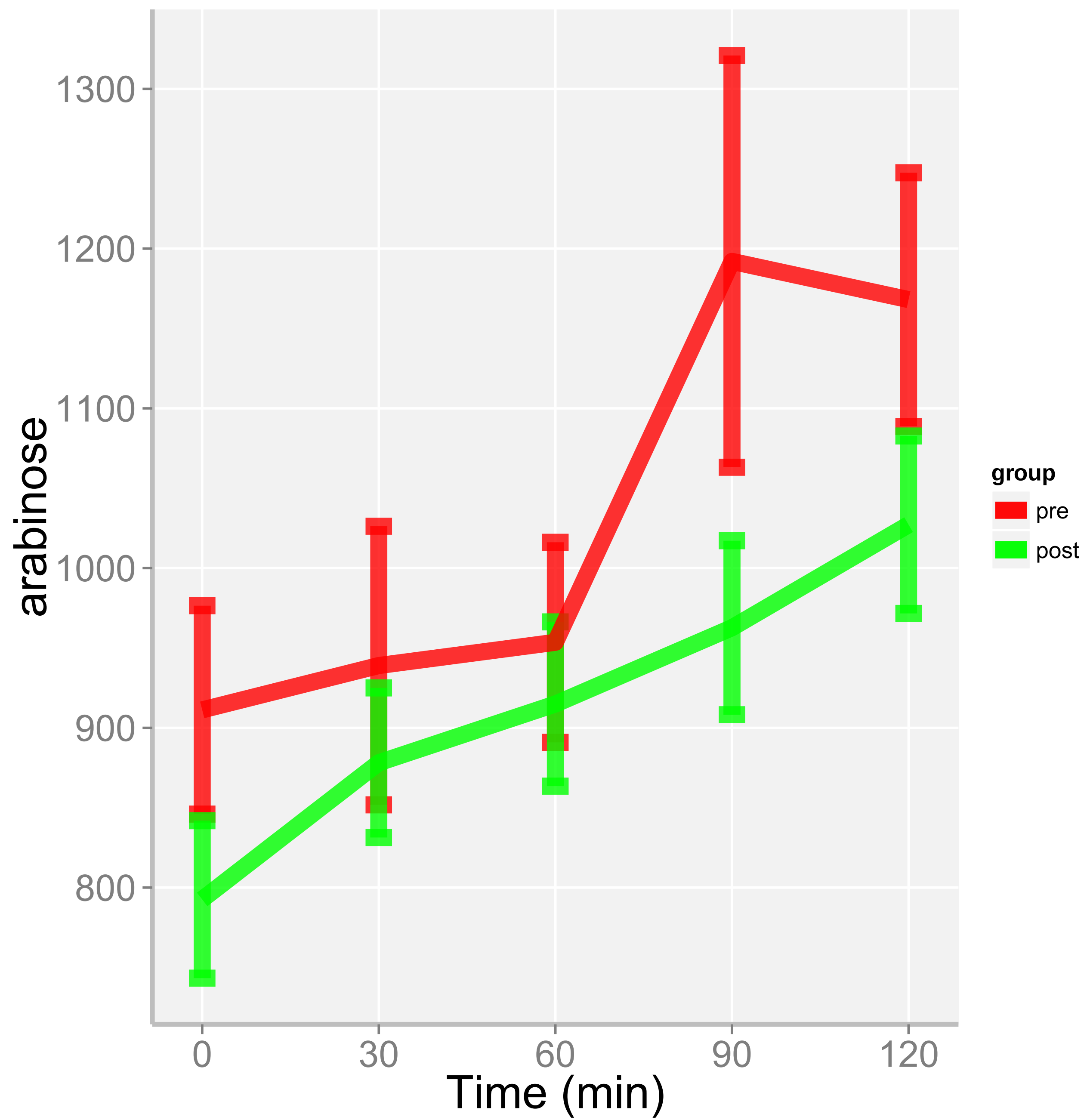

arabinose
1300
1200
1100
1000
900
800
0
30
60
90
120
Time (min)
group
pre
post

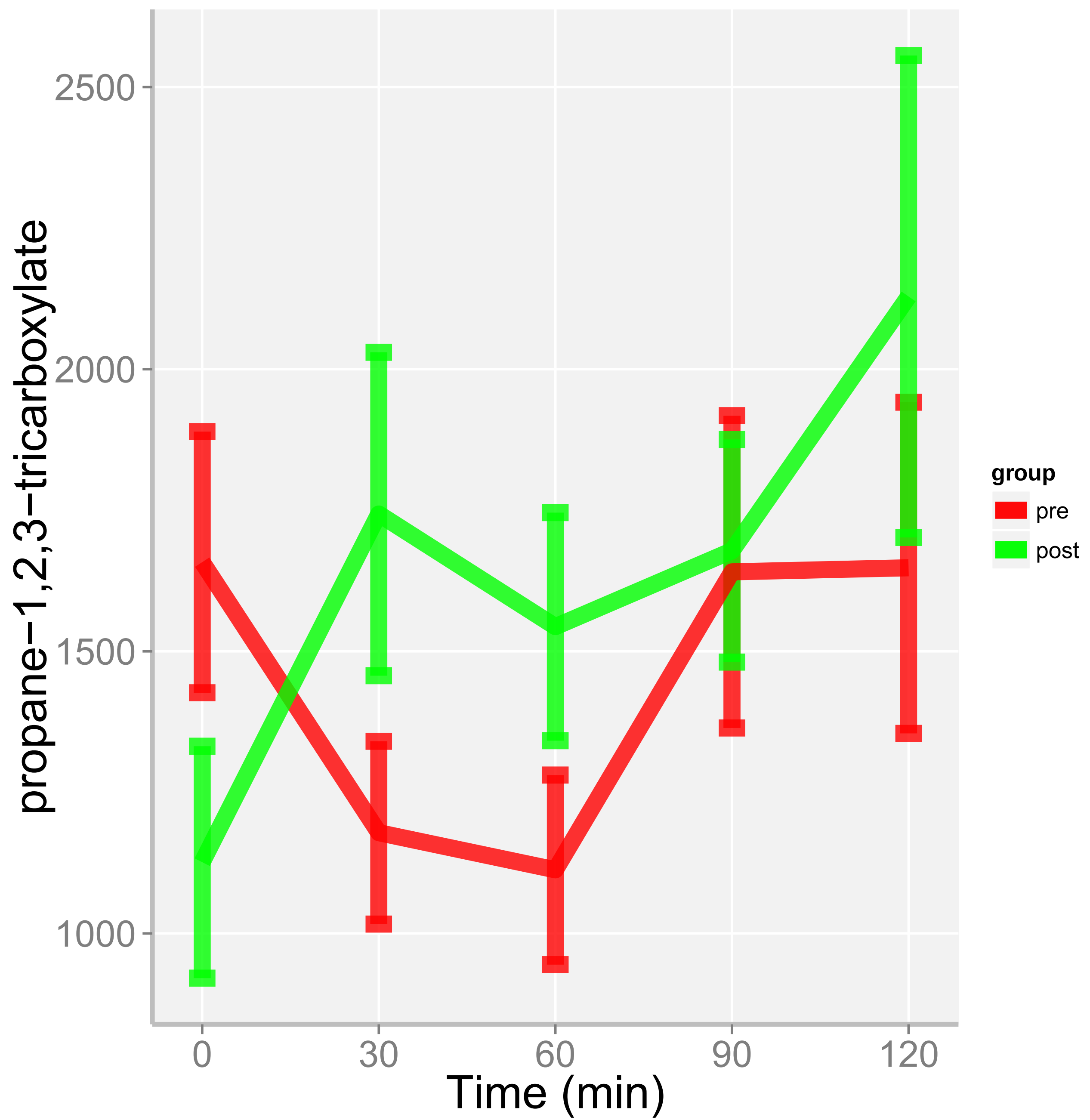

propane−1,2,3−tricarboxylate
2500
2000
1500
1000
0
30
60
90
120
Time (min)
group
pre
post

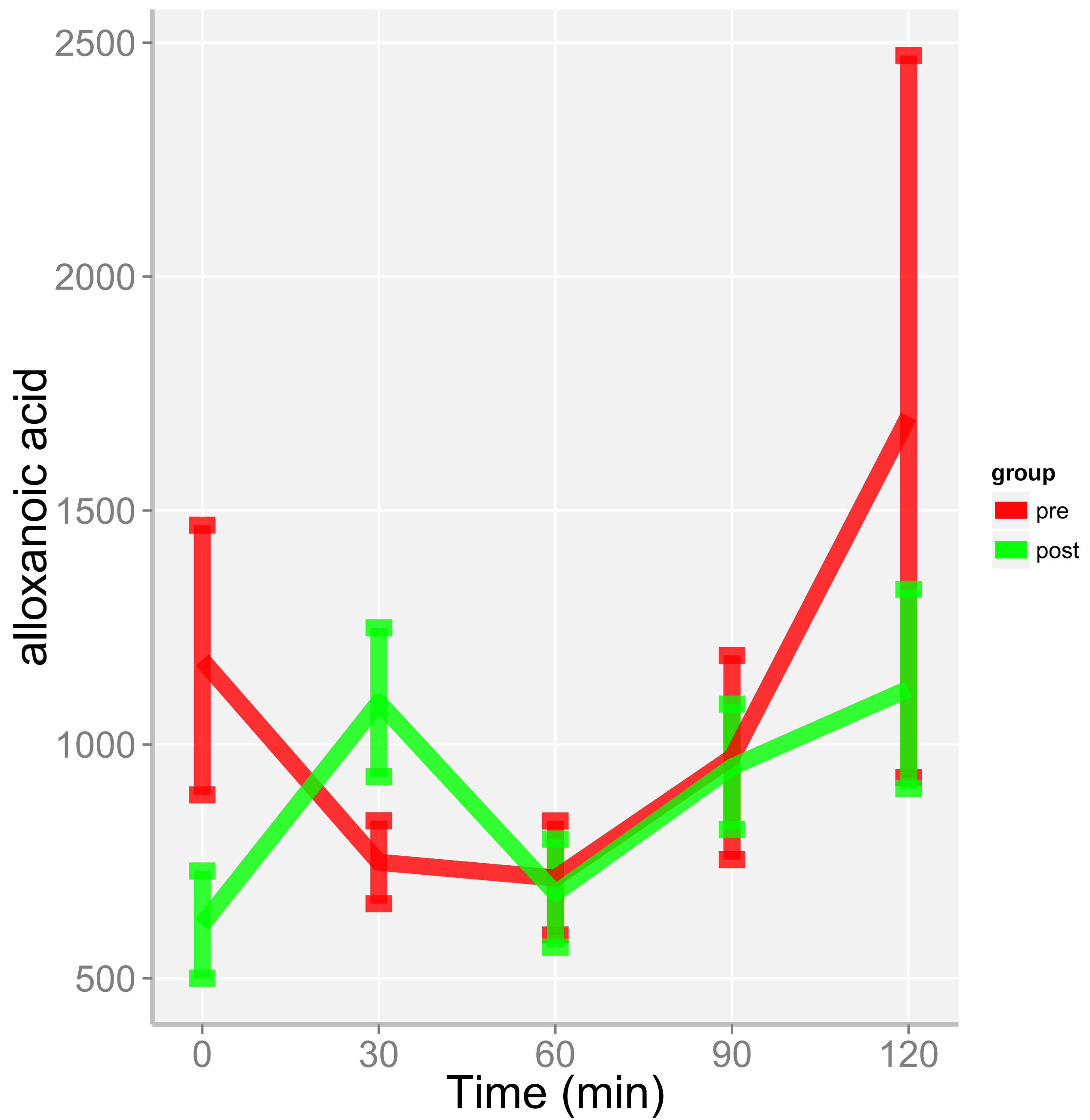

alloxanoic acid
2500
2000
1500
1000
500
0
30
60
90
120
Time (min)
group
pre
post

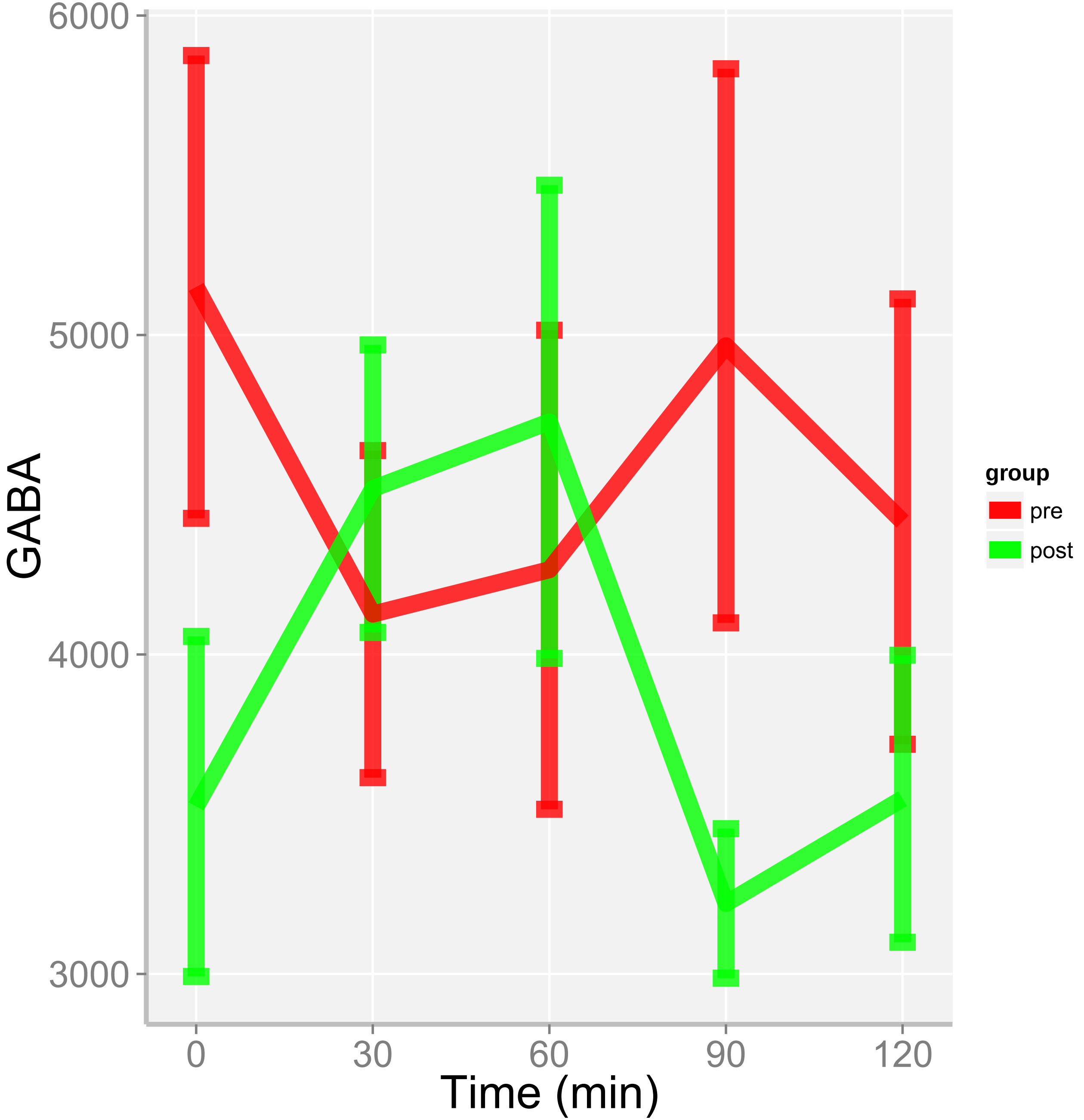
6000
5000
4000
3000
GABA
0
30
60
90
120
Time (min)
group
pre
post

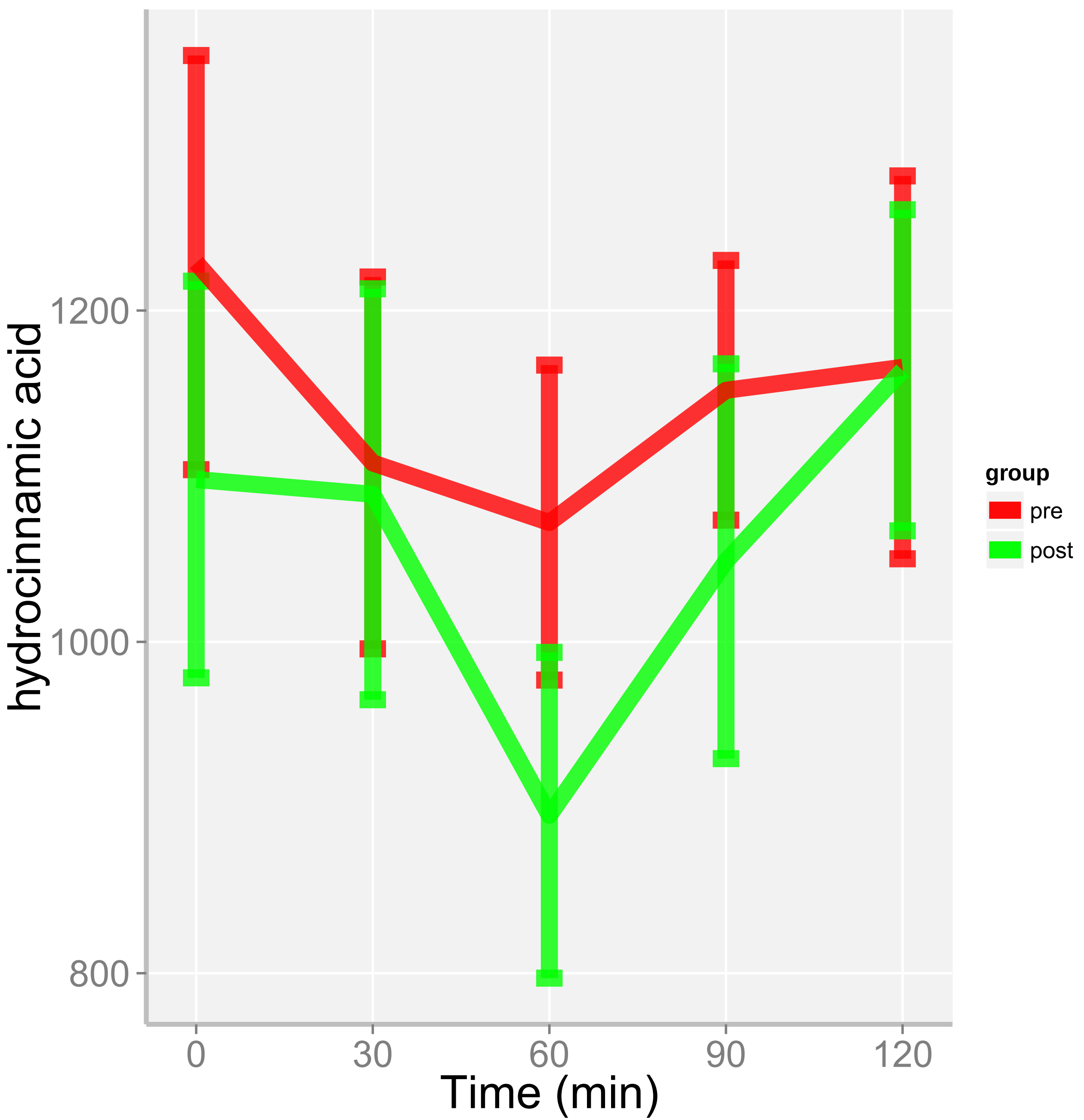

hydrocinnamic acid
1200
1000
800
0
30
60
90
120
Time (min)
group
pre
post

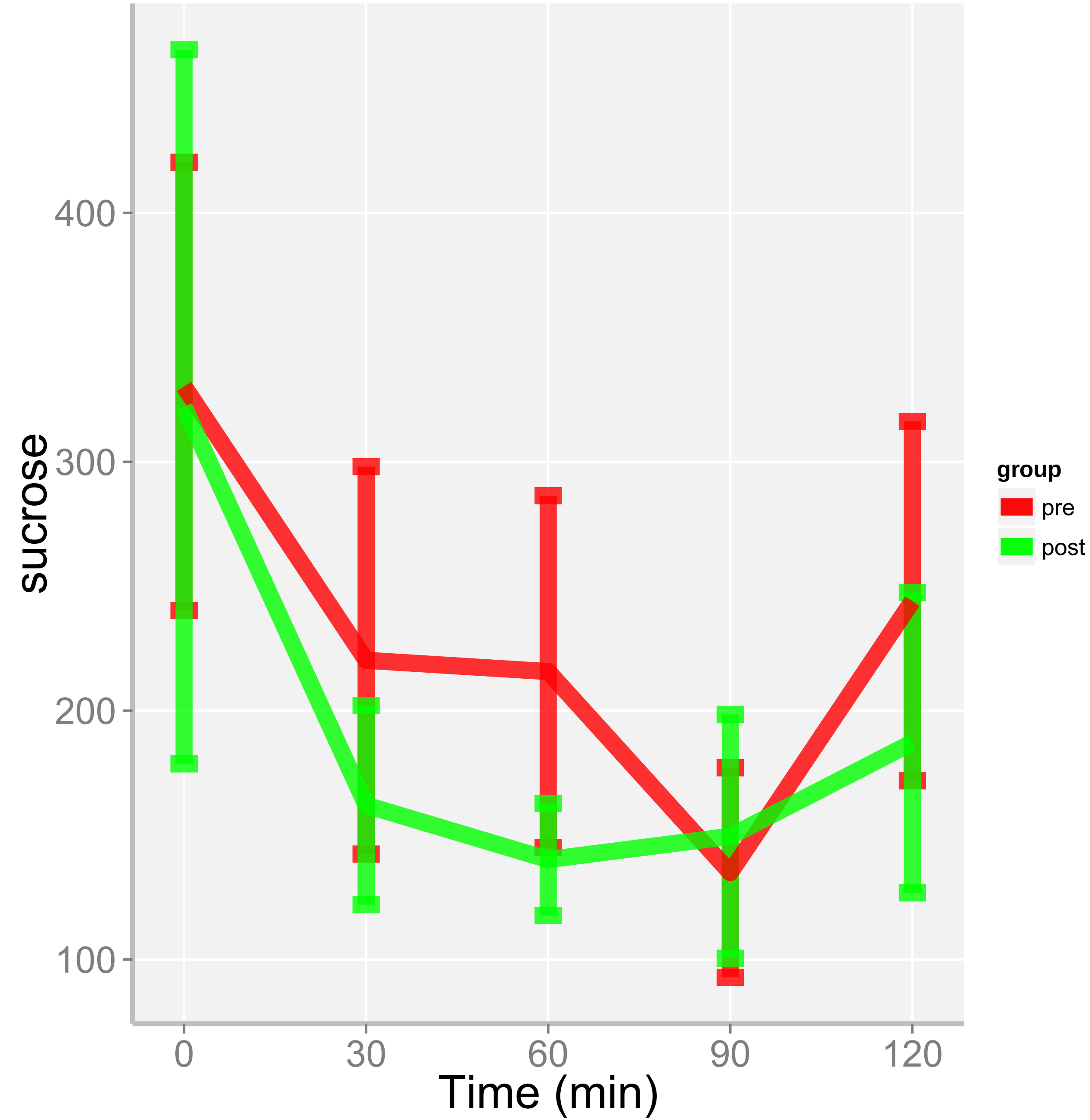

sucrose
400
300
200
100
0
30
60
90
120
Time (min)
group
pre
post

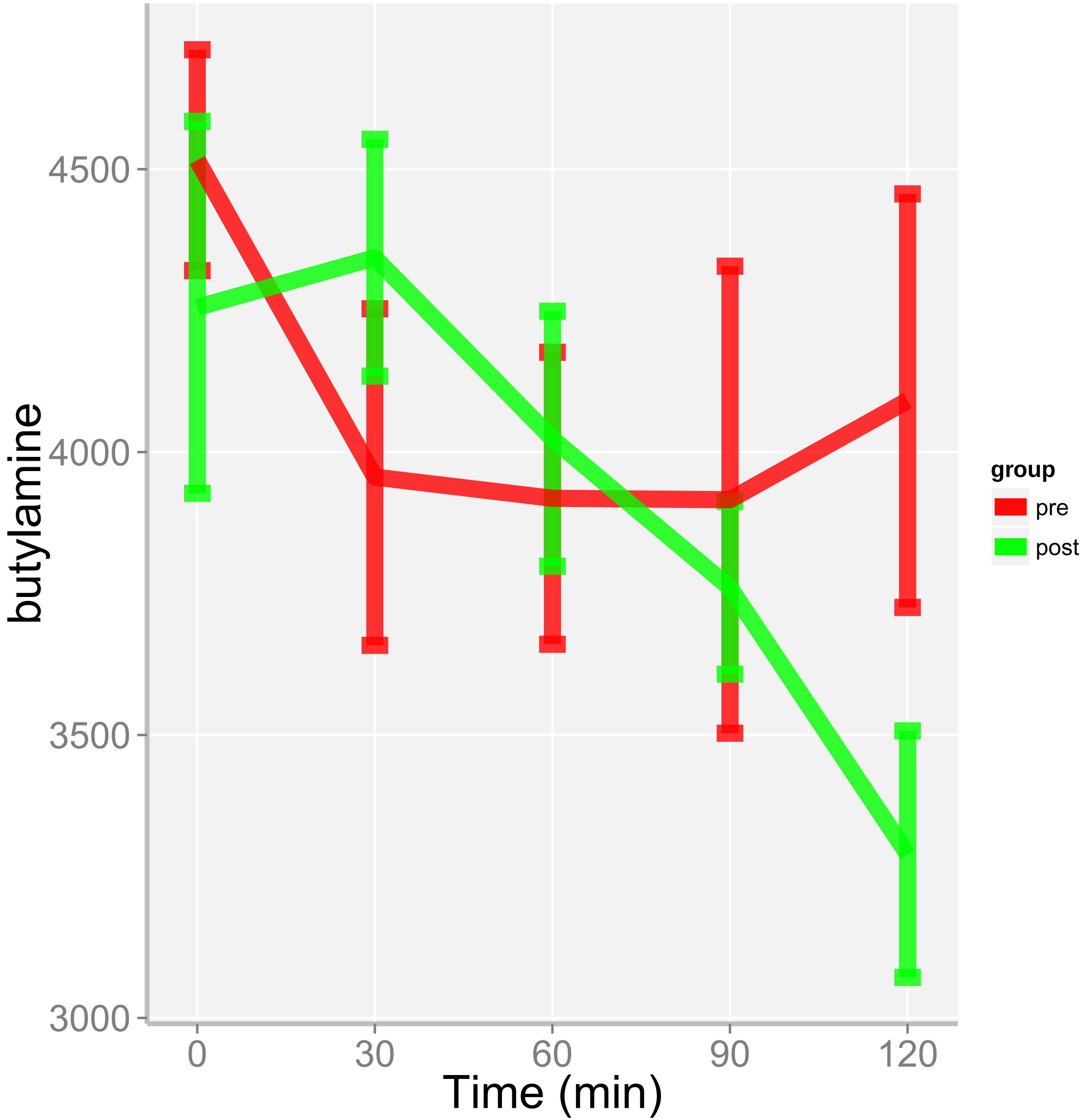

butylamine
4500
4000
3500
3000
0
30
60
90
120
Time (min)
group
pre
post

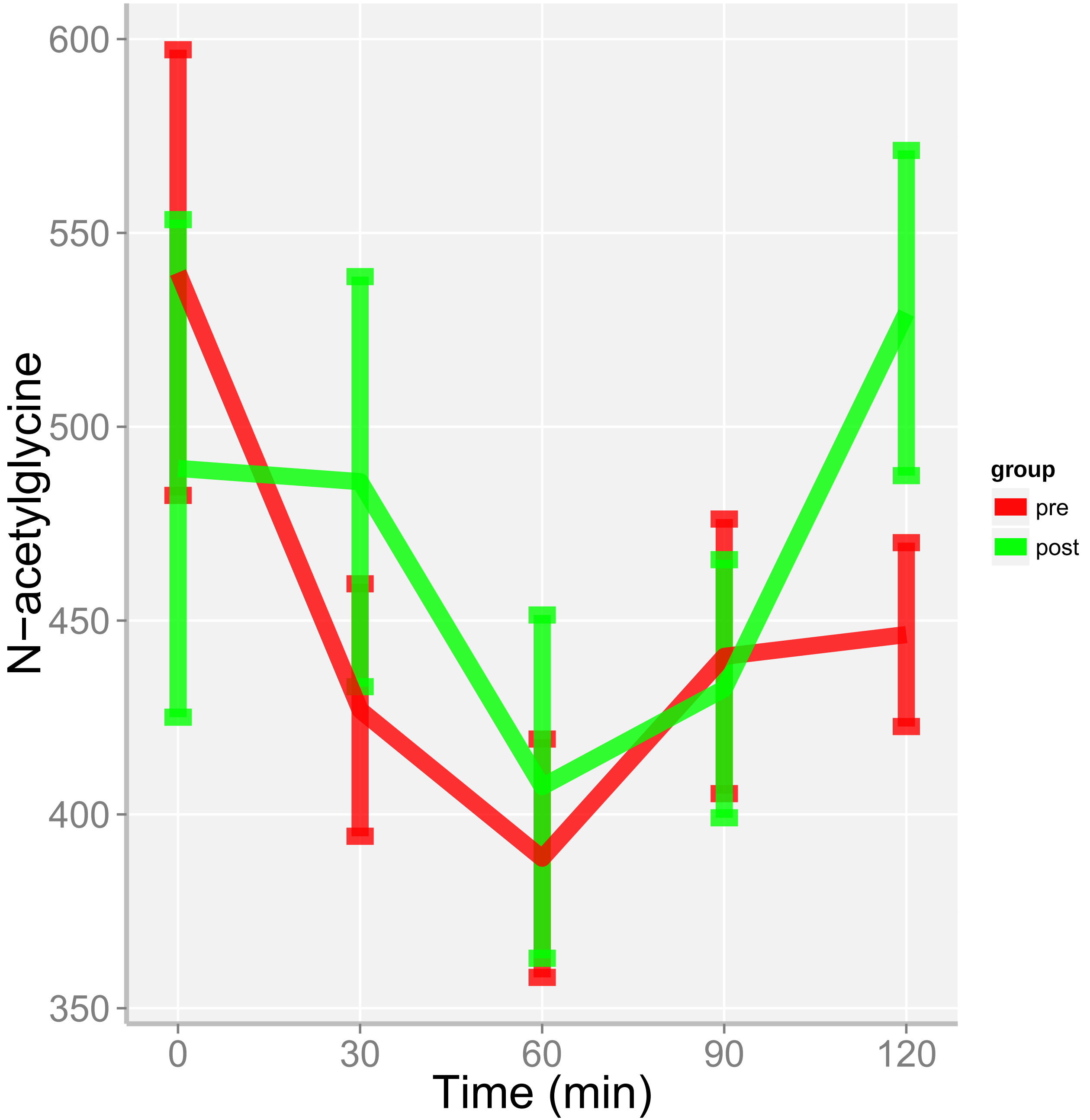

600
550
500
450
400
350
0
30
60
90
120
Time (min)
N−acetylglycine
group
pre
post

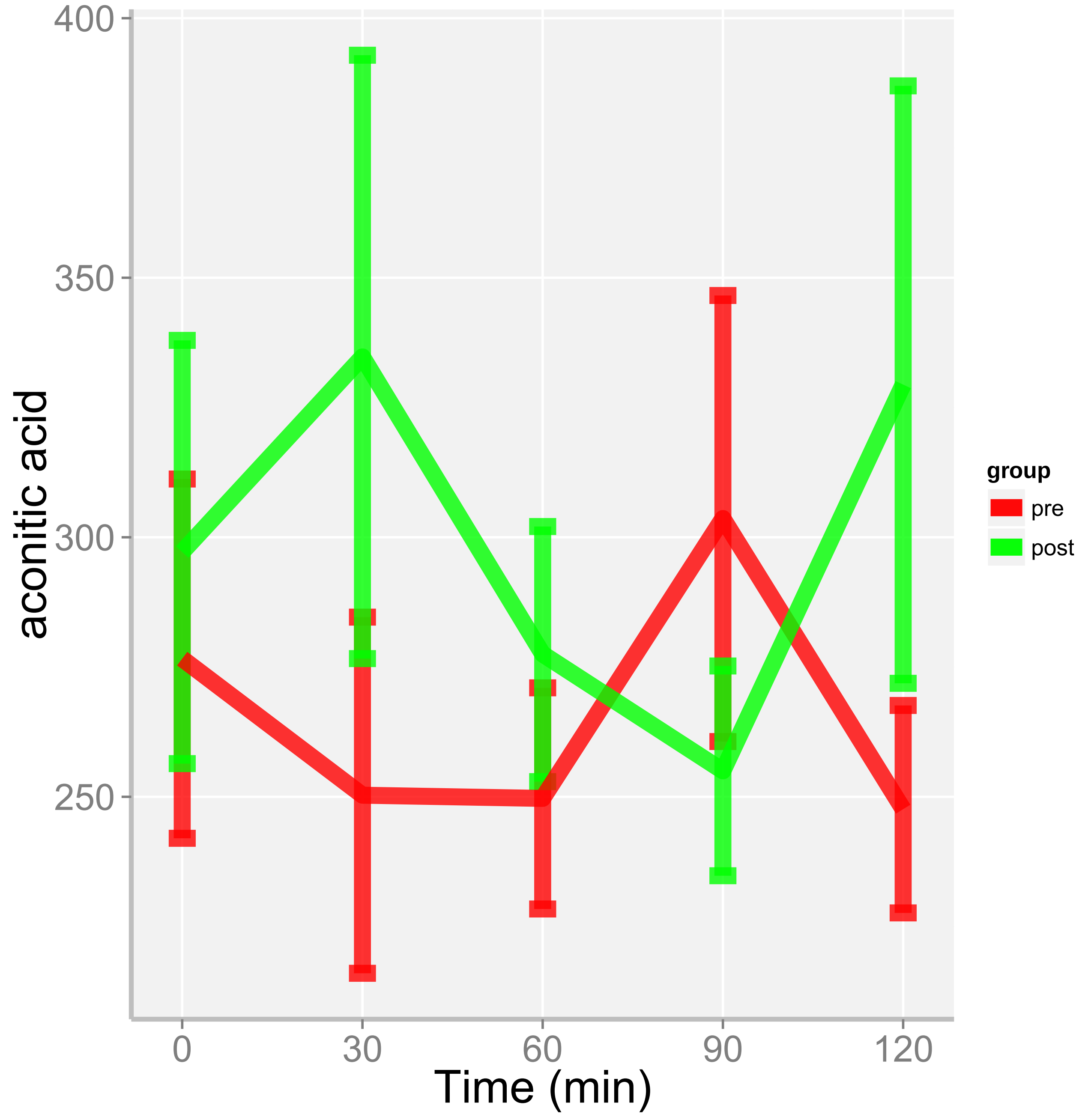

aconitic acid
400
350
300
250
0
30
60
90
120
Time (min)
group
pre
post

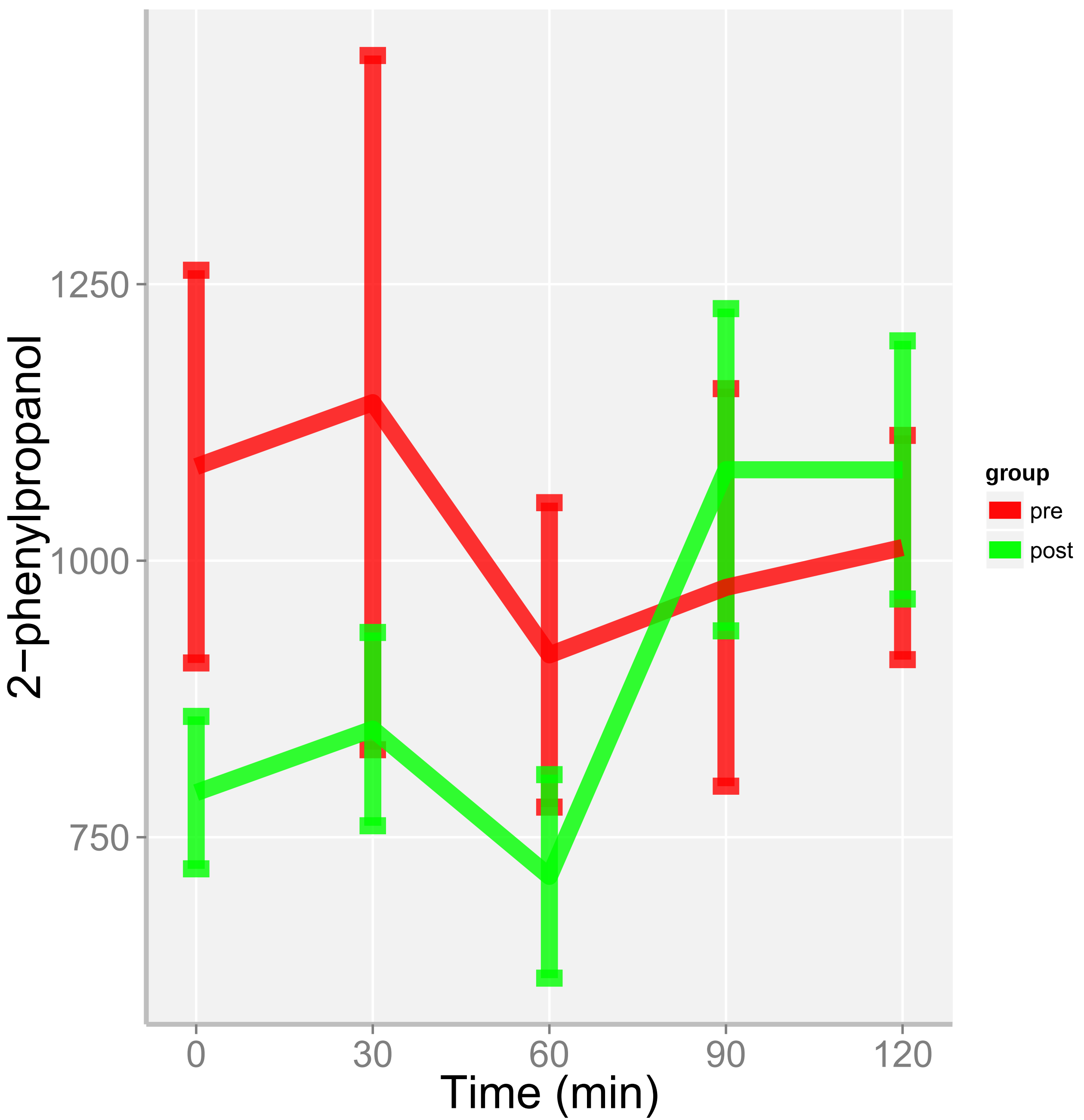

2−phenylpropanol
1250
1000
750
0
30
60
90
120
Time (min)
group
pre
post

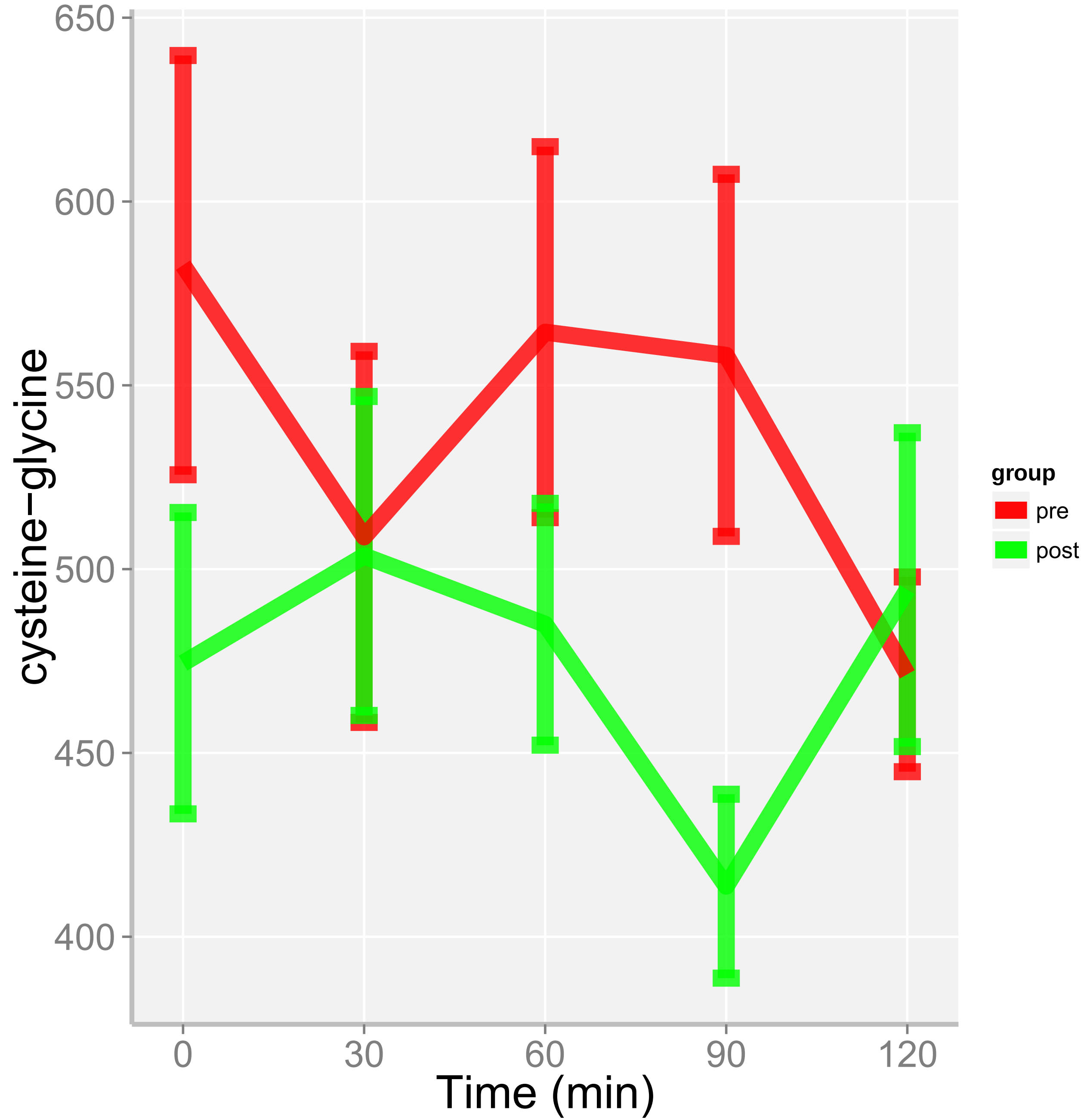

650
600
550
500
450
400
cysteine−glycine
0
30
60
90
120
Time (min)
group
pre
post

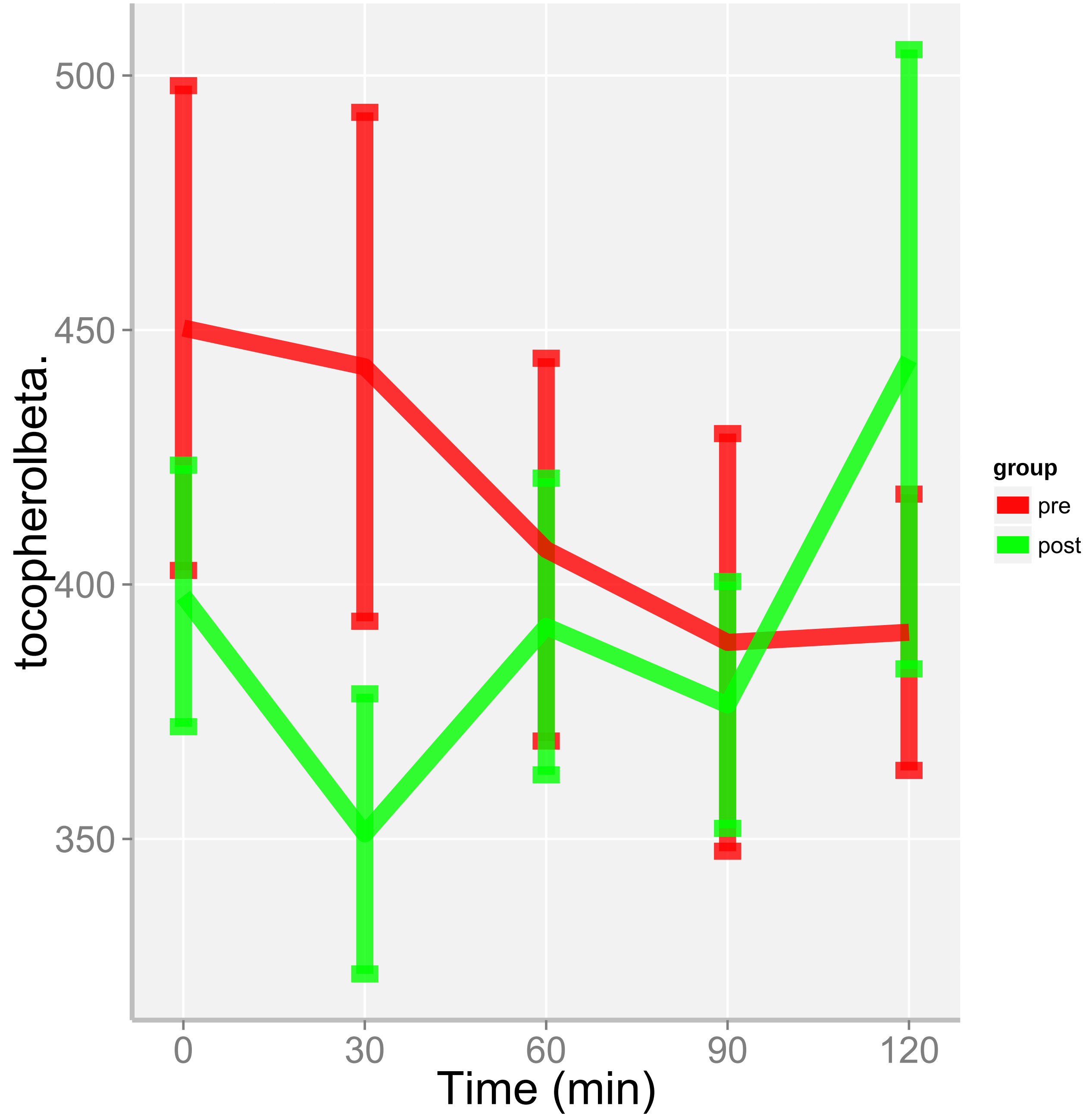

500
450
400
350
tocopherolbeta.
0
30
60
90
120
Time (min)
group
pre
post

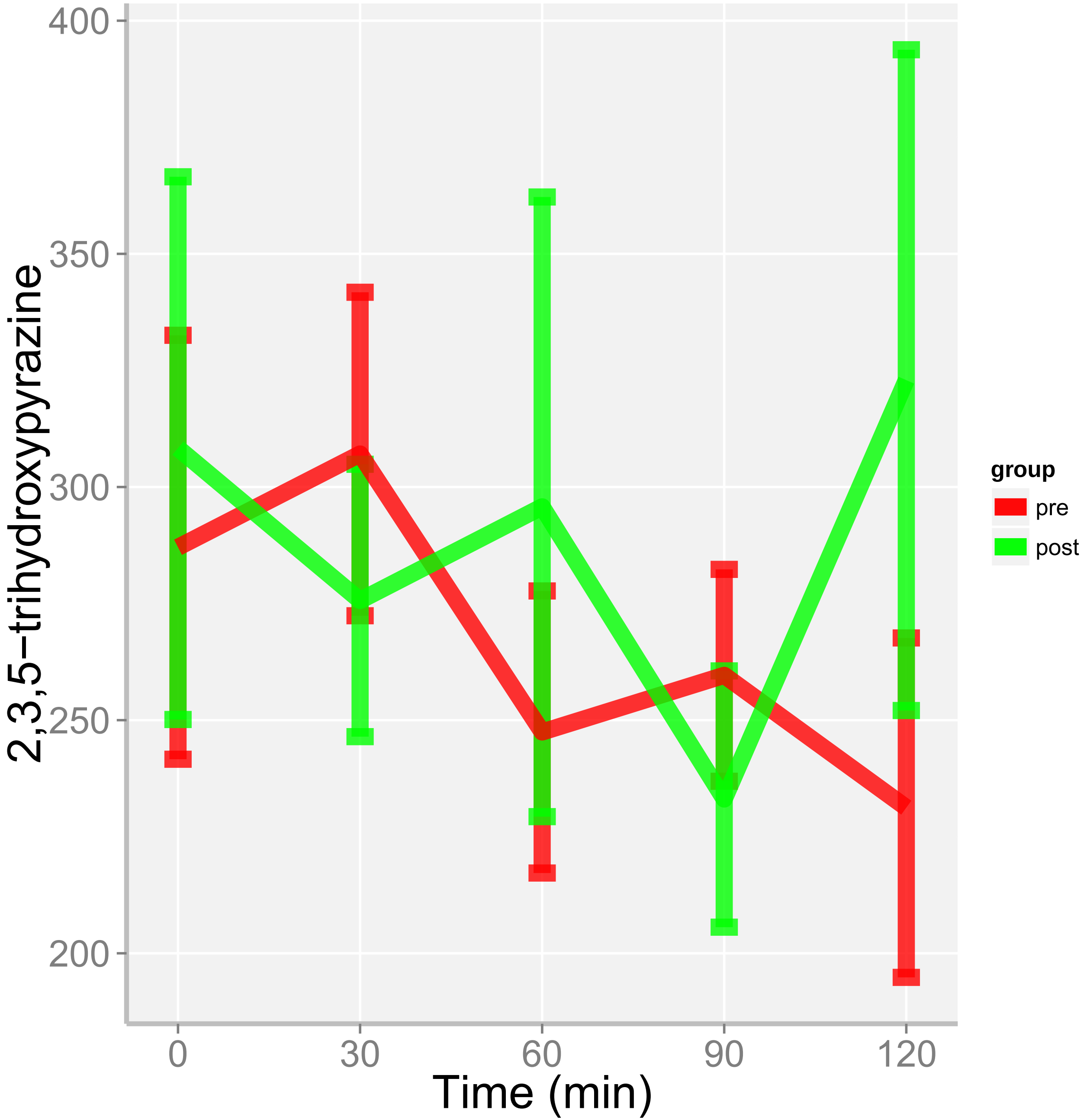

2,3,5−trihydroxypyrazine
400
350
300
250
200
0
30
60
90
120
Time (min)
group
pre
post

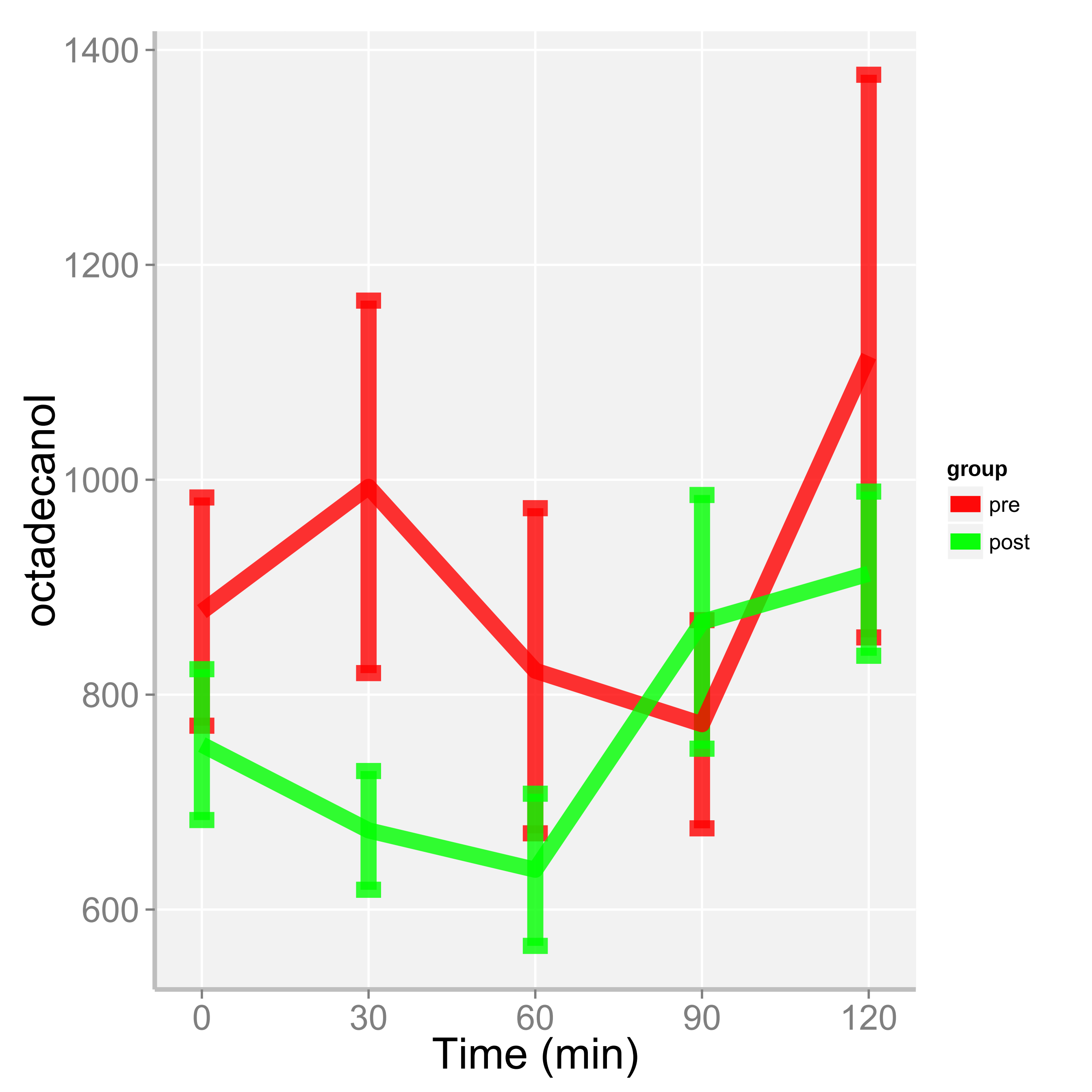

octadecanol
1400
1200
1000
800
600
0
30
60
90
120
Time (min)
group
pre
post

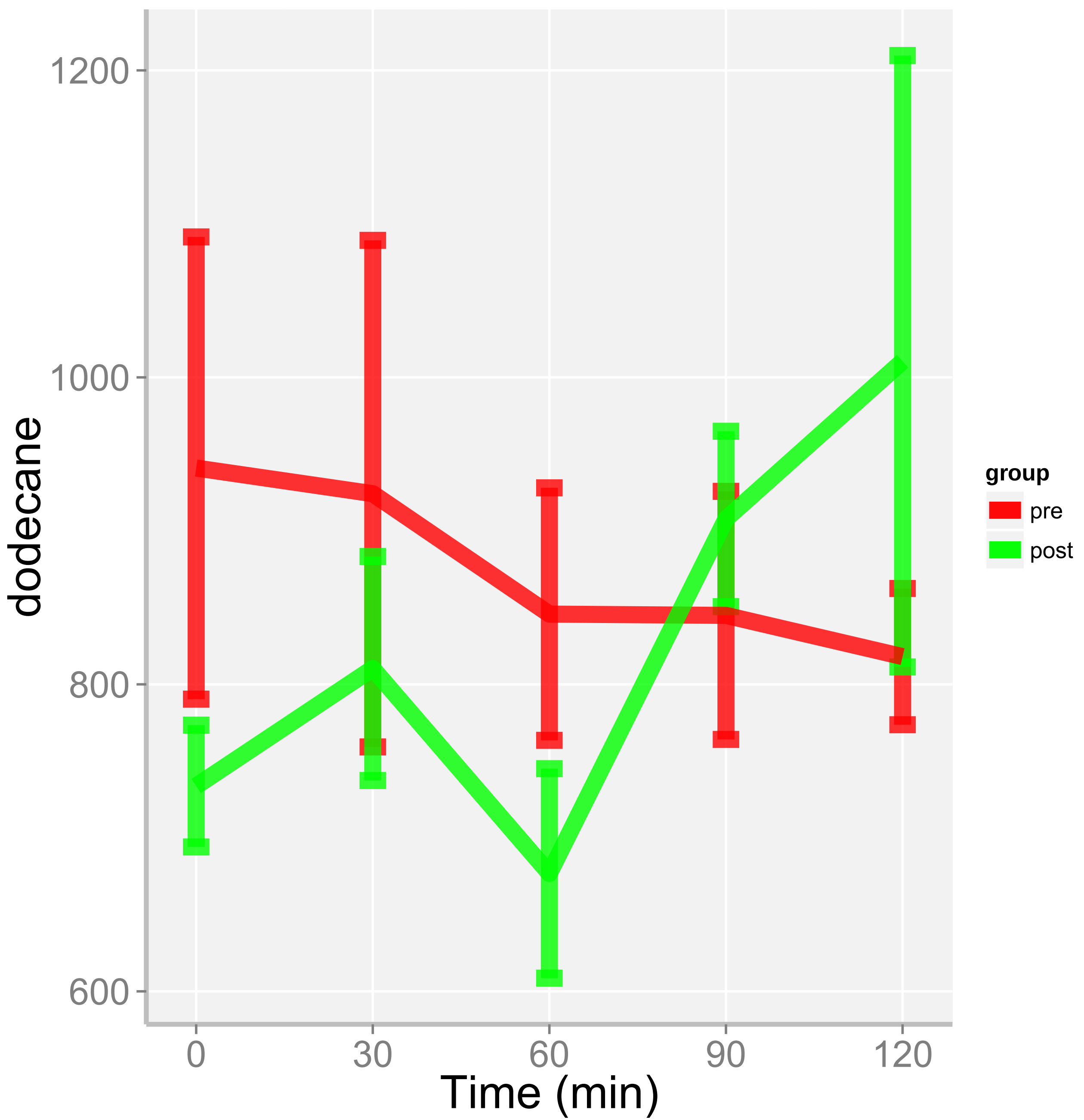

dodecane
1200
1000
800
600
0
30
60
90
120
Time (min)
group
pre
post

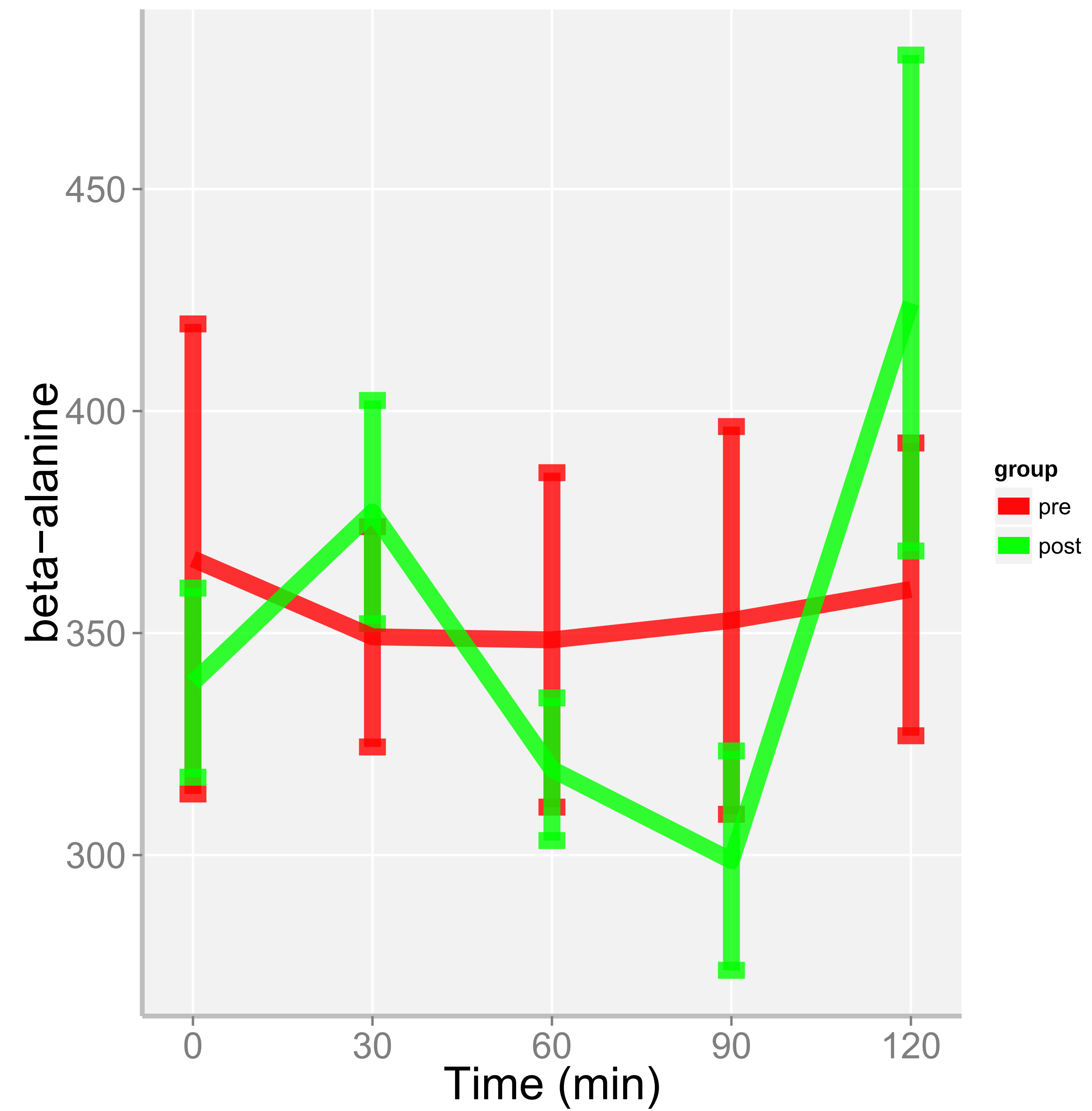

450
400
350
300
beta−alanine
0
30
60
90
120
Time (min)
group
pre
post

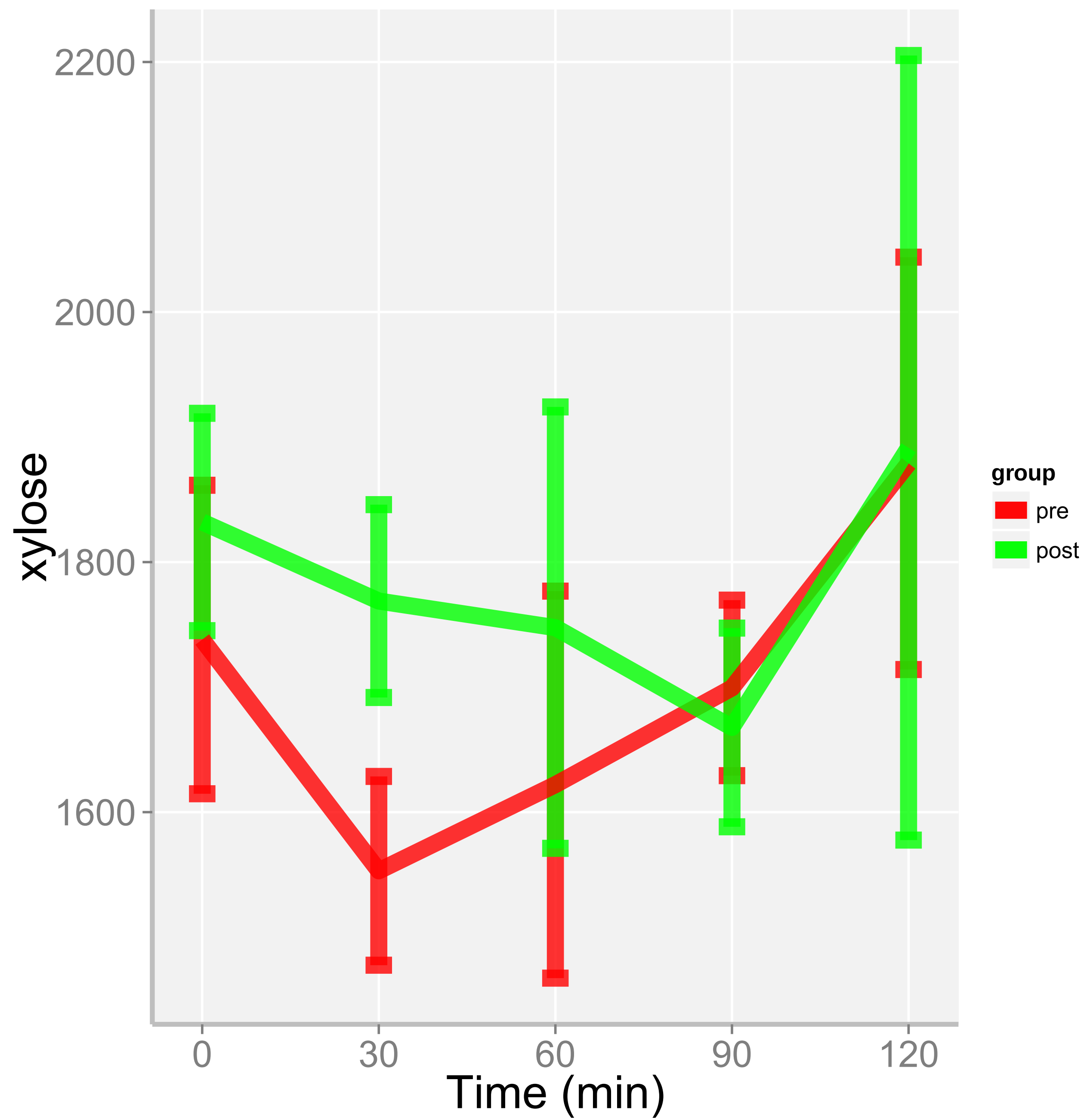

2200
2000
1800
1600
xylose
0
30
60
90
120
Time (min)
group
pre
post

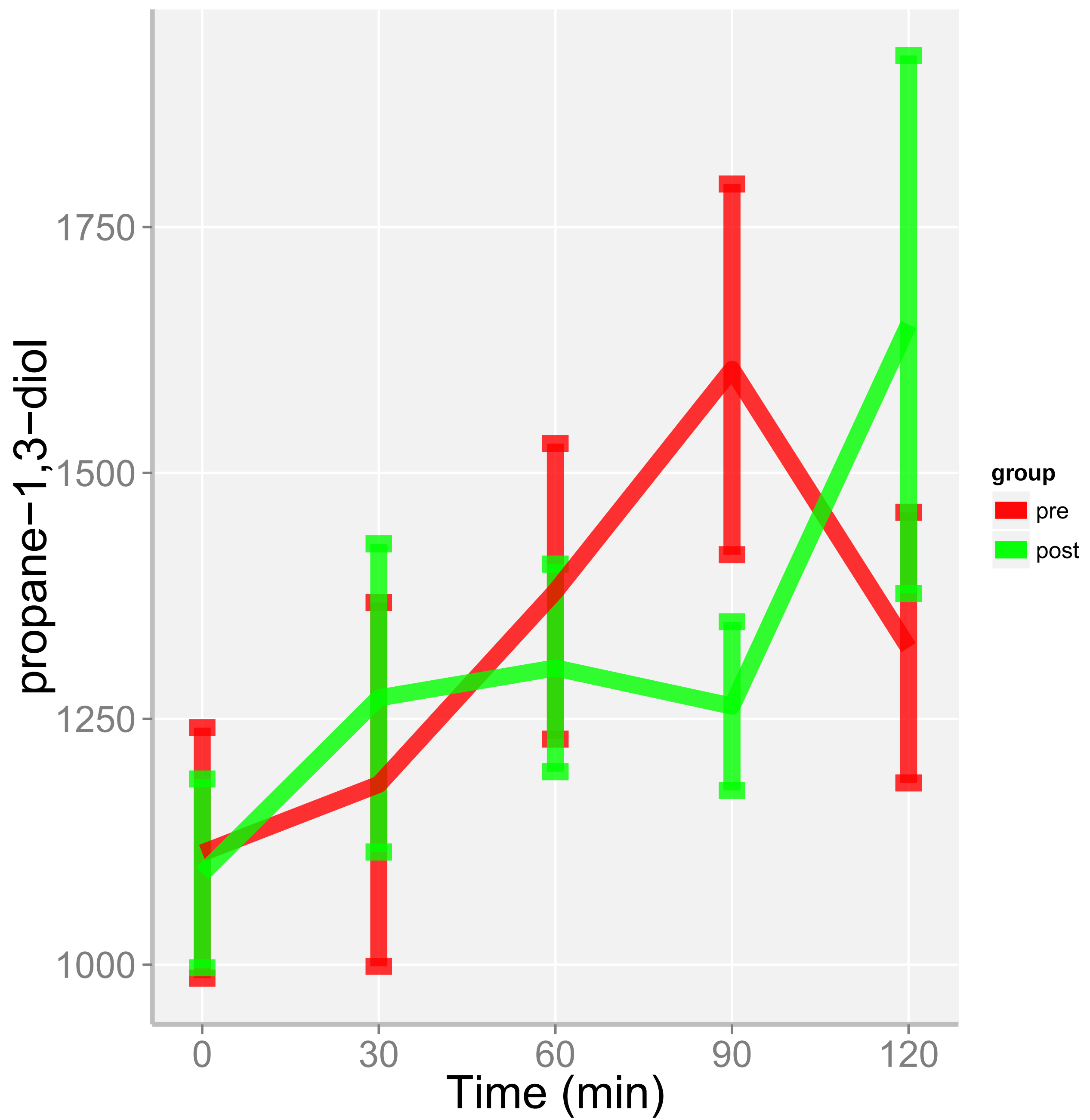

propane−1,3−diol
1750
1500
1250
1000
0
30
60
90
120
Time (min)
group
pre
post

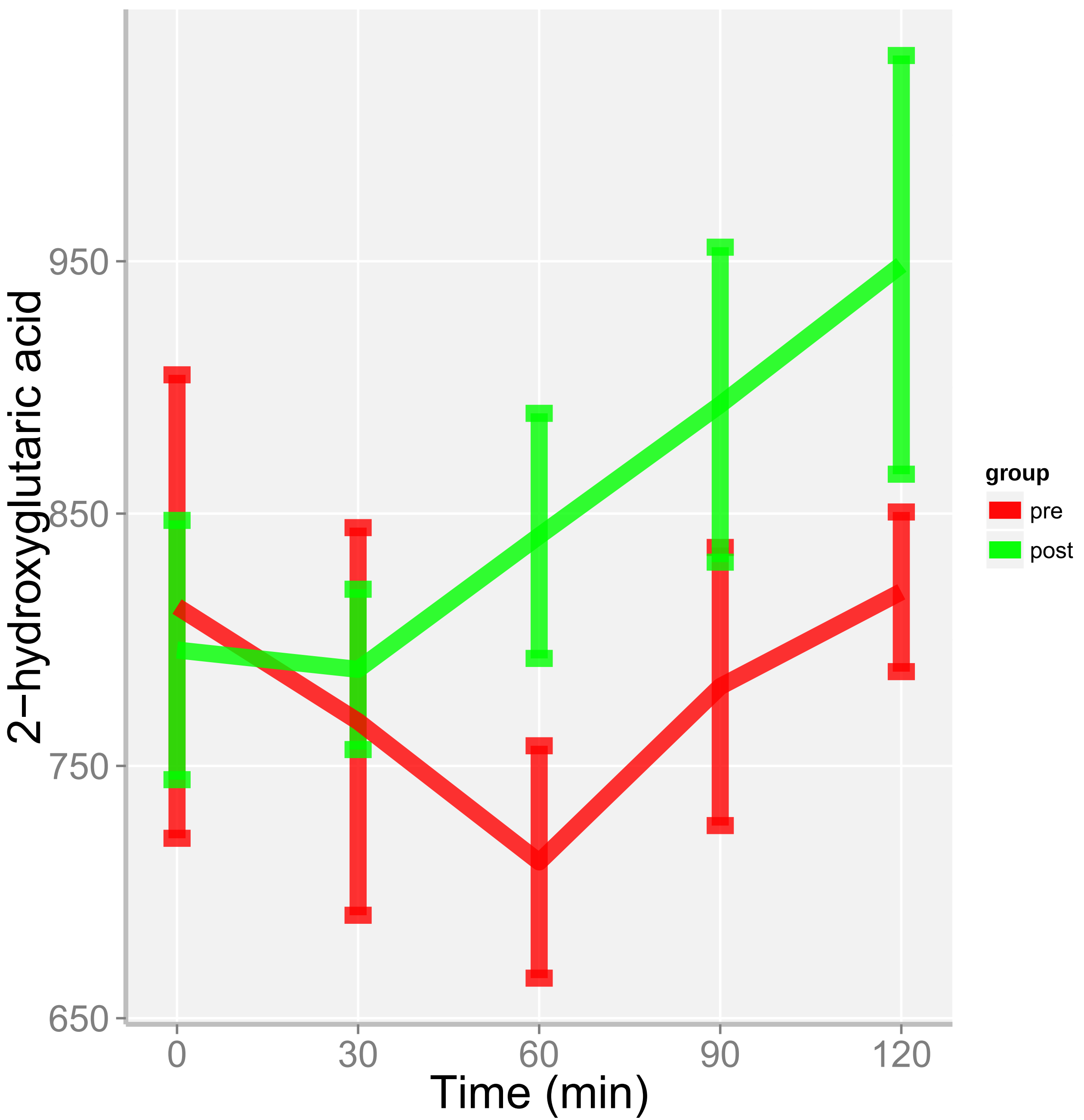

2−hydroxyglutaric acid
950
850
750
650
0
30
60
90
120
Time (min)
group
pre
post

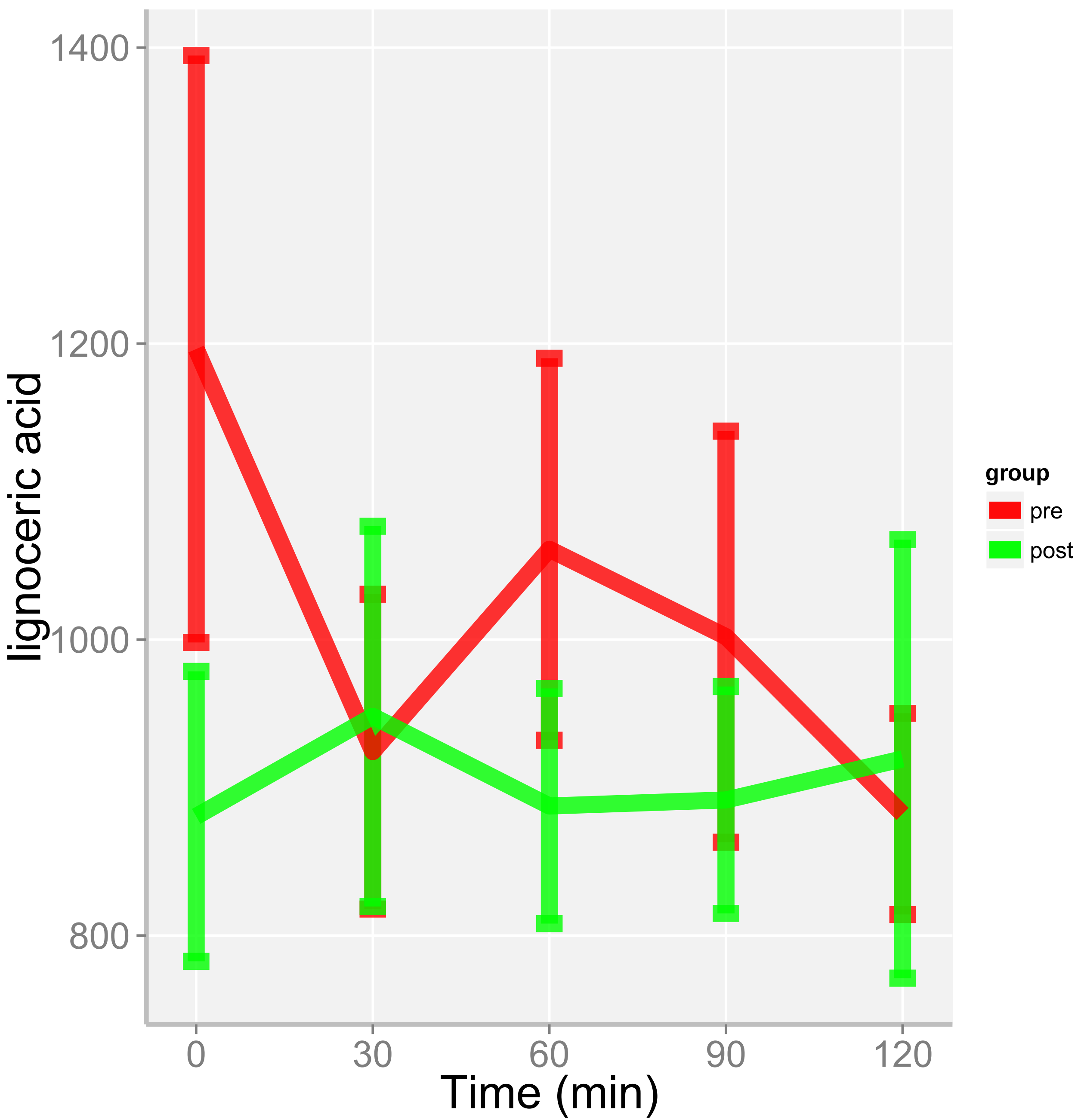

lignoceric acid
1400
1200
1000
800
0
30
60
90
120
Time (min)
group
pre
post

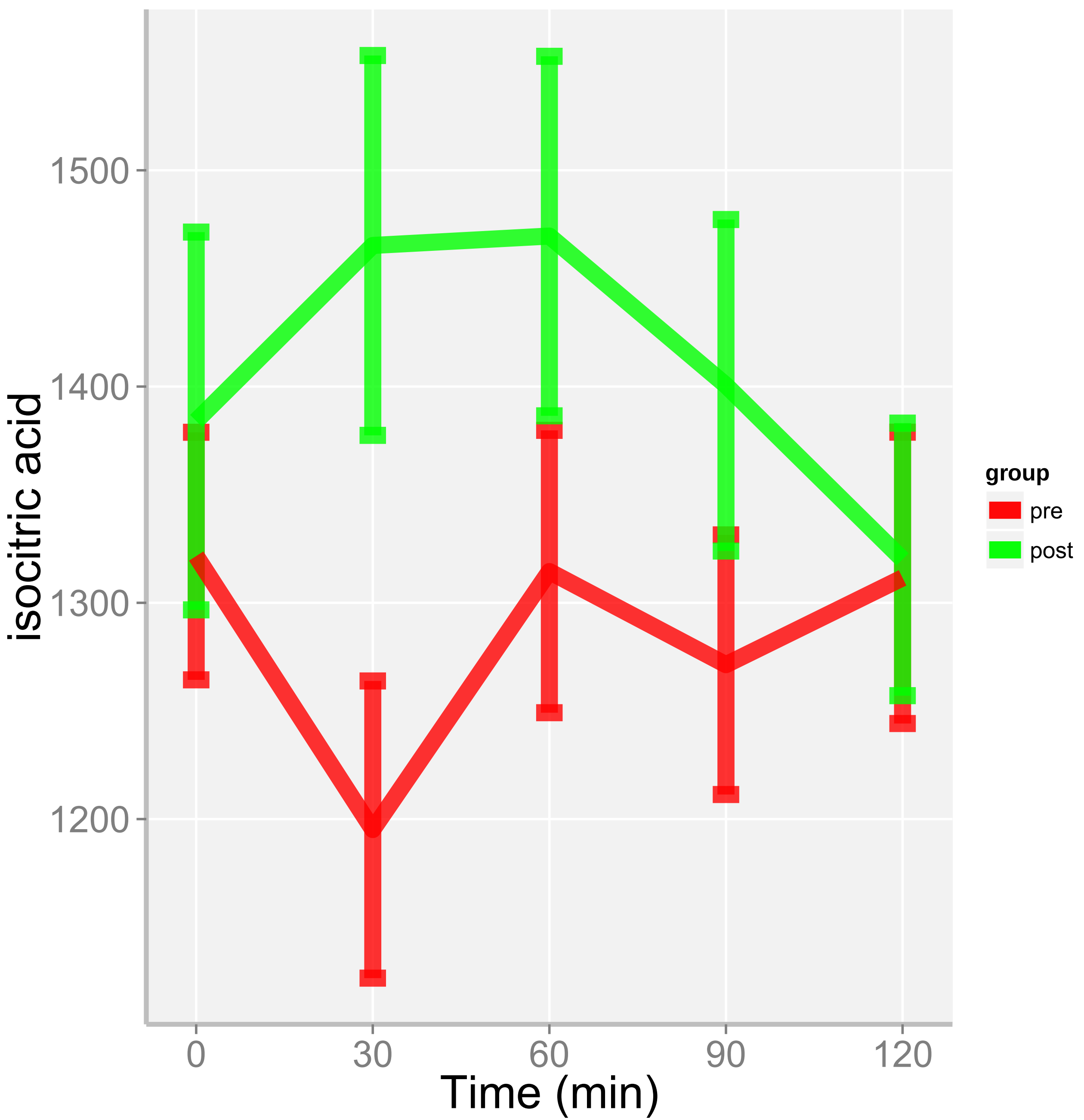

isocitric acid
1500
1400
1300
1200
0
30
60
90
120
Time (min)
group
pre
post

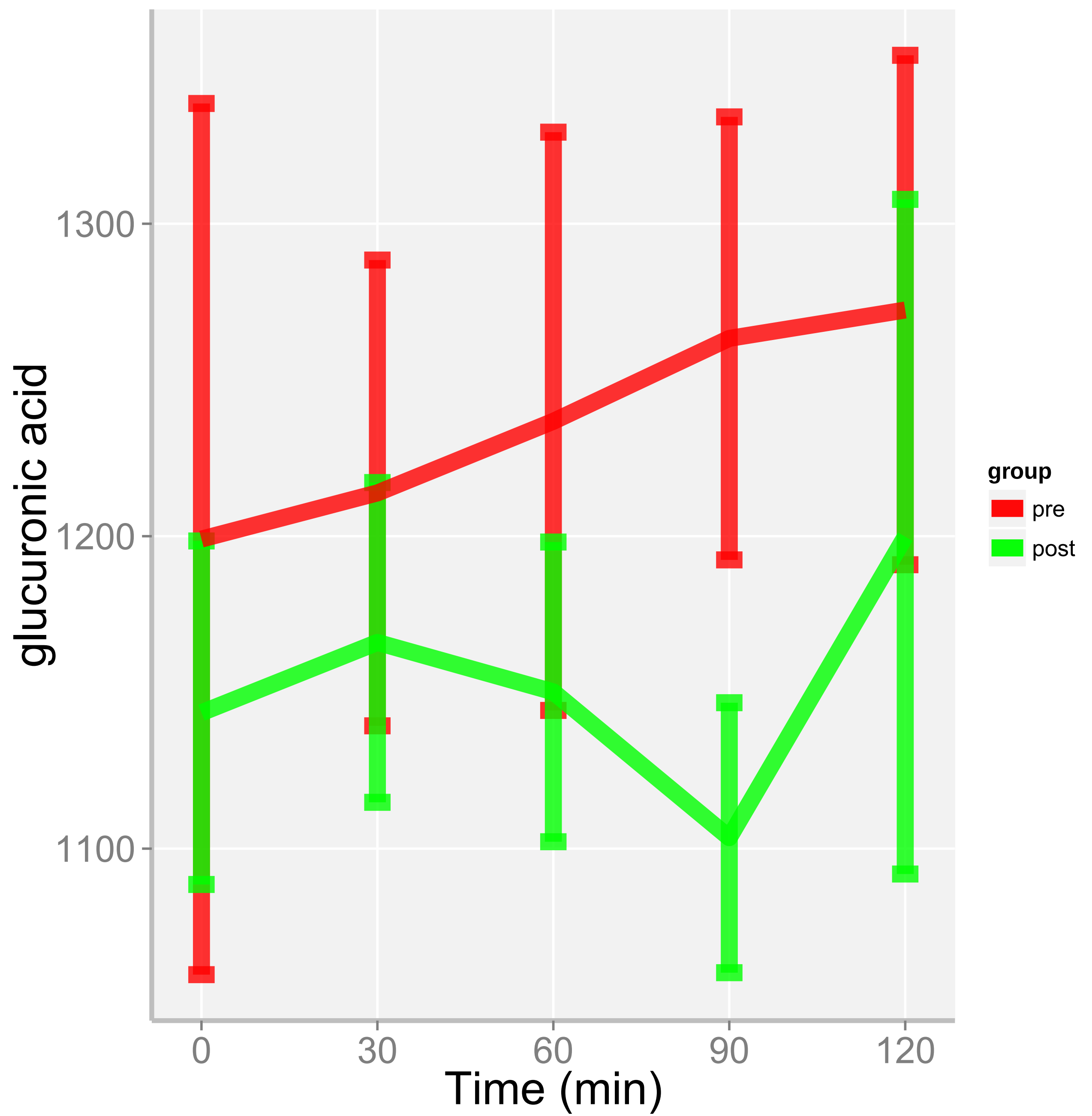
glucuronic acid
1300
1200
1100
0
30
60
90
120
Time (min)
group
pre
post

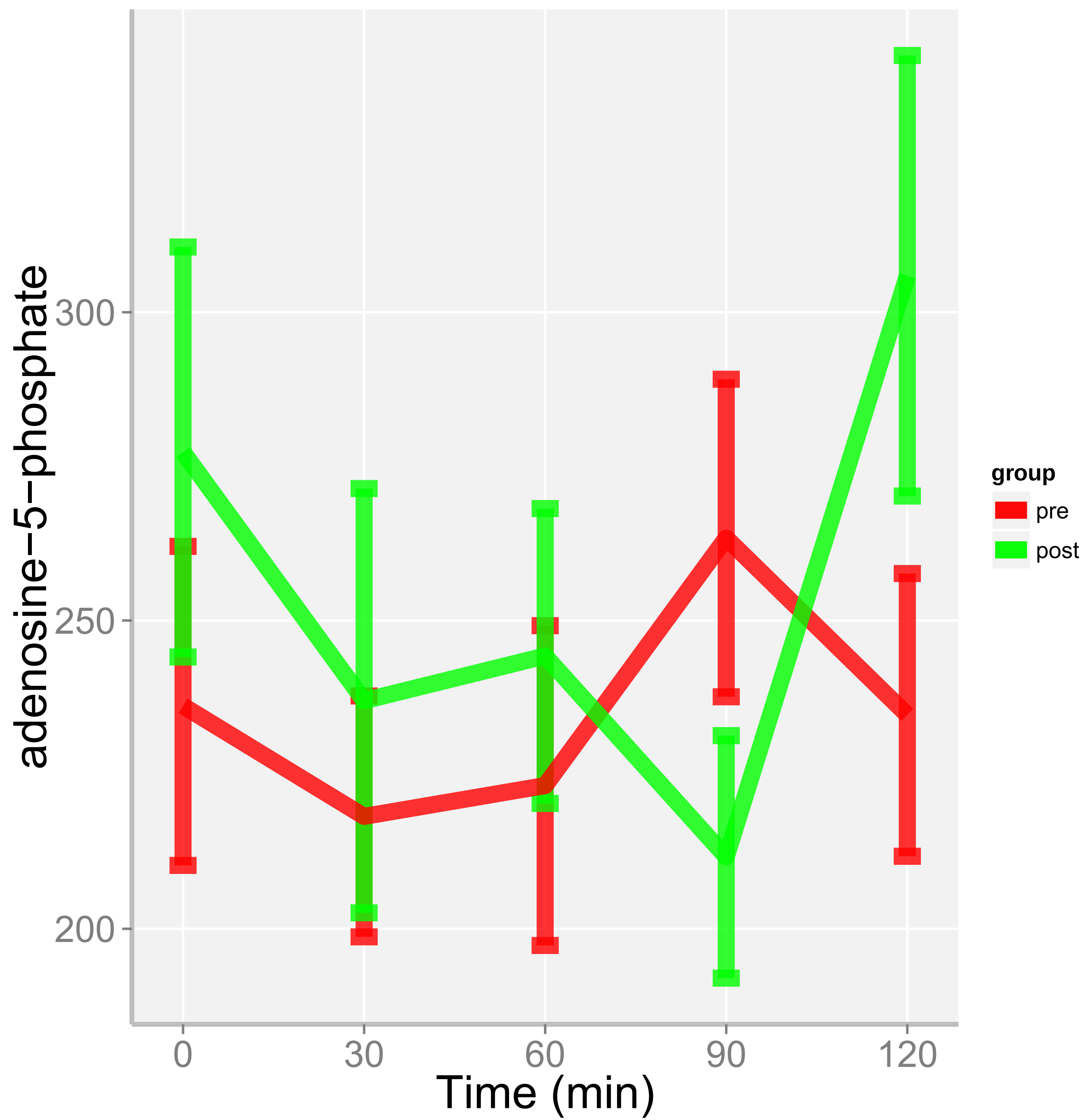
adenosine−5−phosphate
300
250
200
0
30
60
90
120
Time (min)
group
pre
post

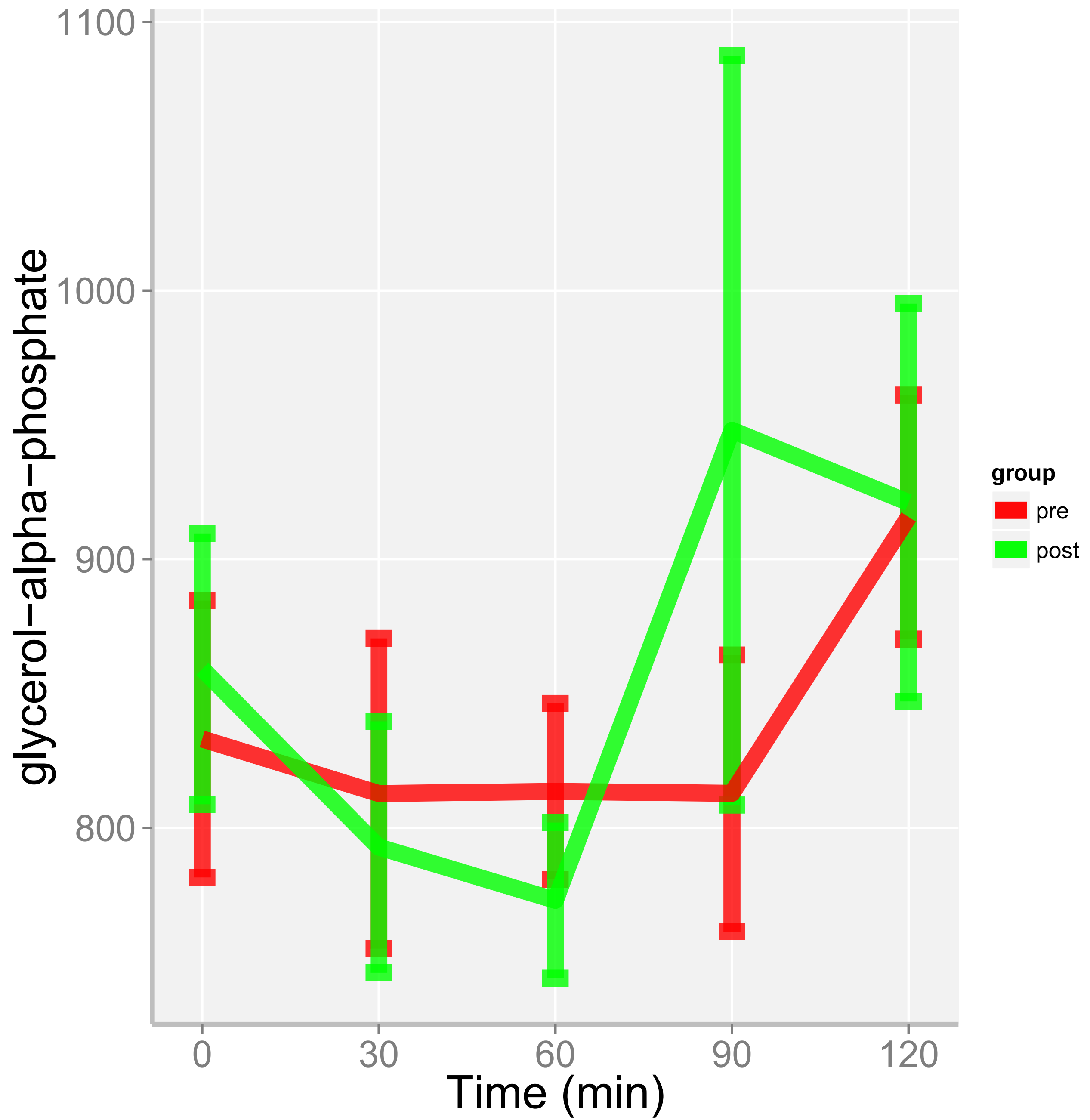

glycerol−alpha−phosphate
1100
1000
900
800
0
30
60
90
120
Time (min)
group
pre
post

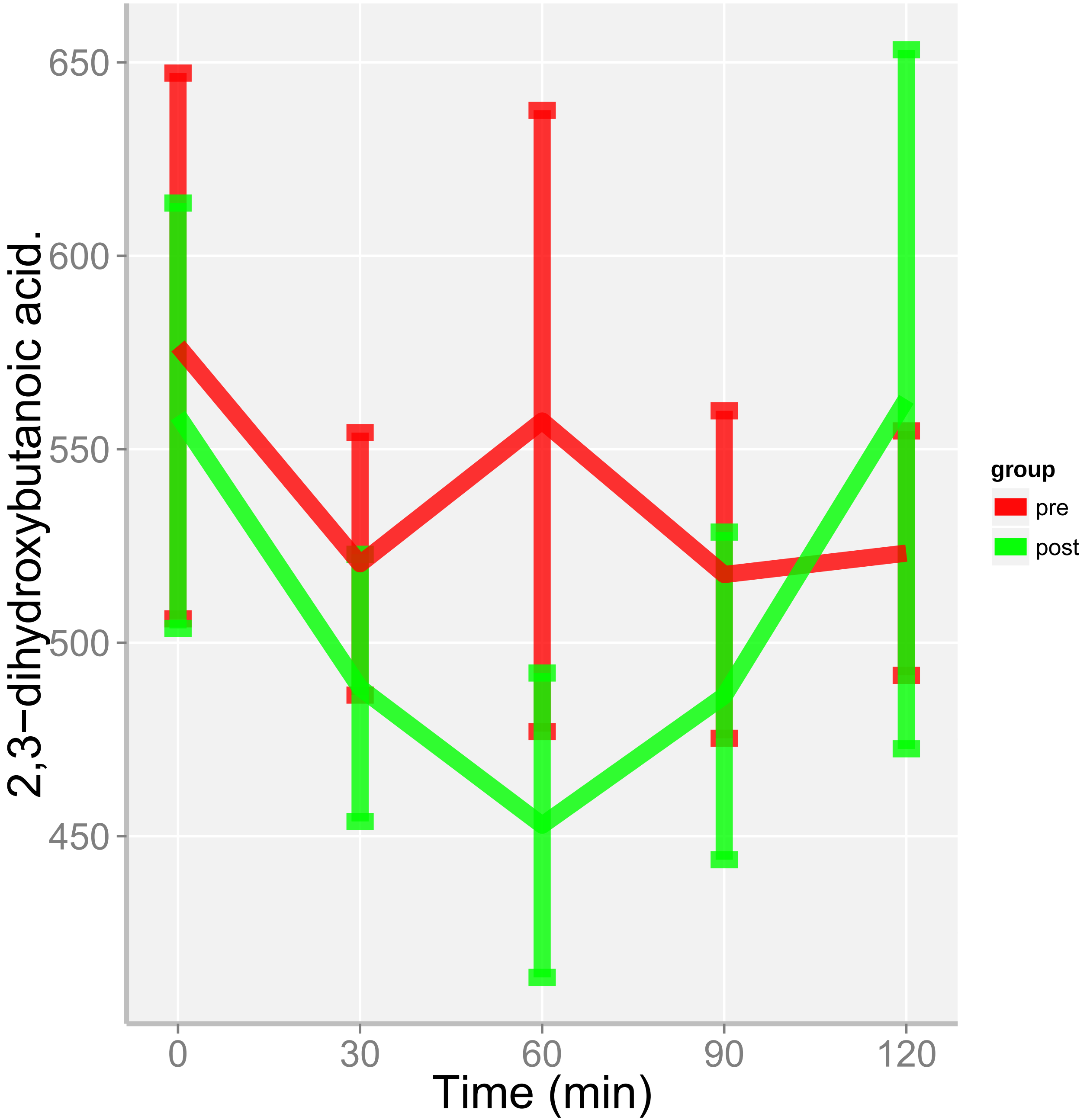

2,3−dihydroxybutanoic acid.
650
600
550
500
450
0
30
60
90
120
Time (min)
group
pre
post

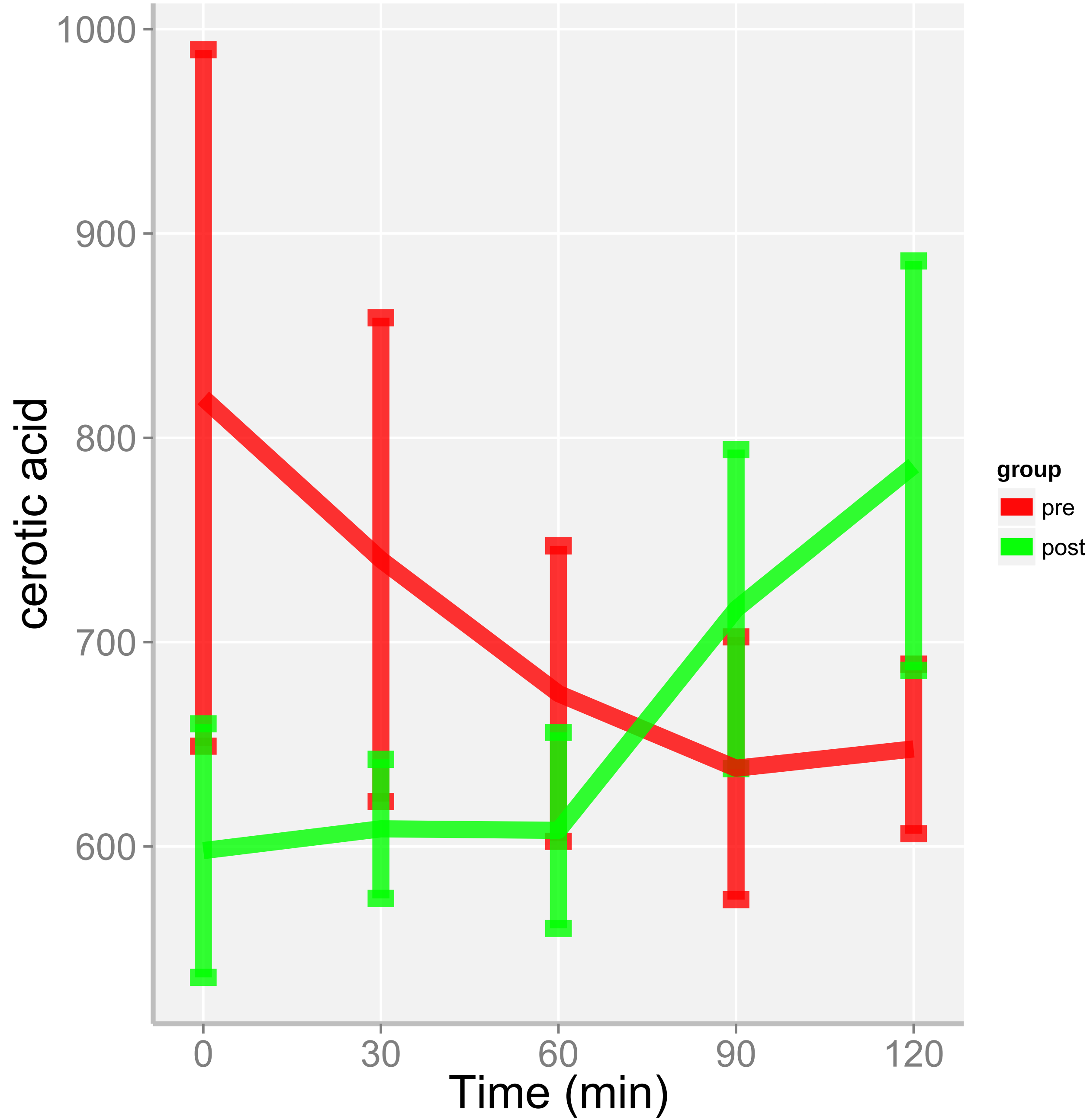

cerotic acid
1000
900
800
700
600
0
30
60
90
120
Time (min)
group
pre
post

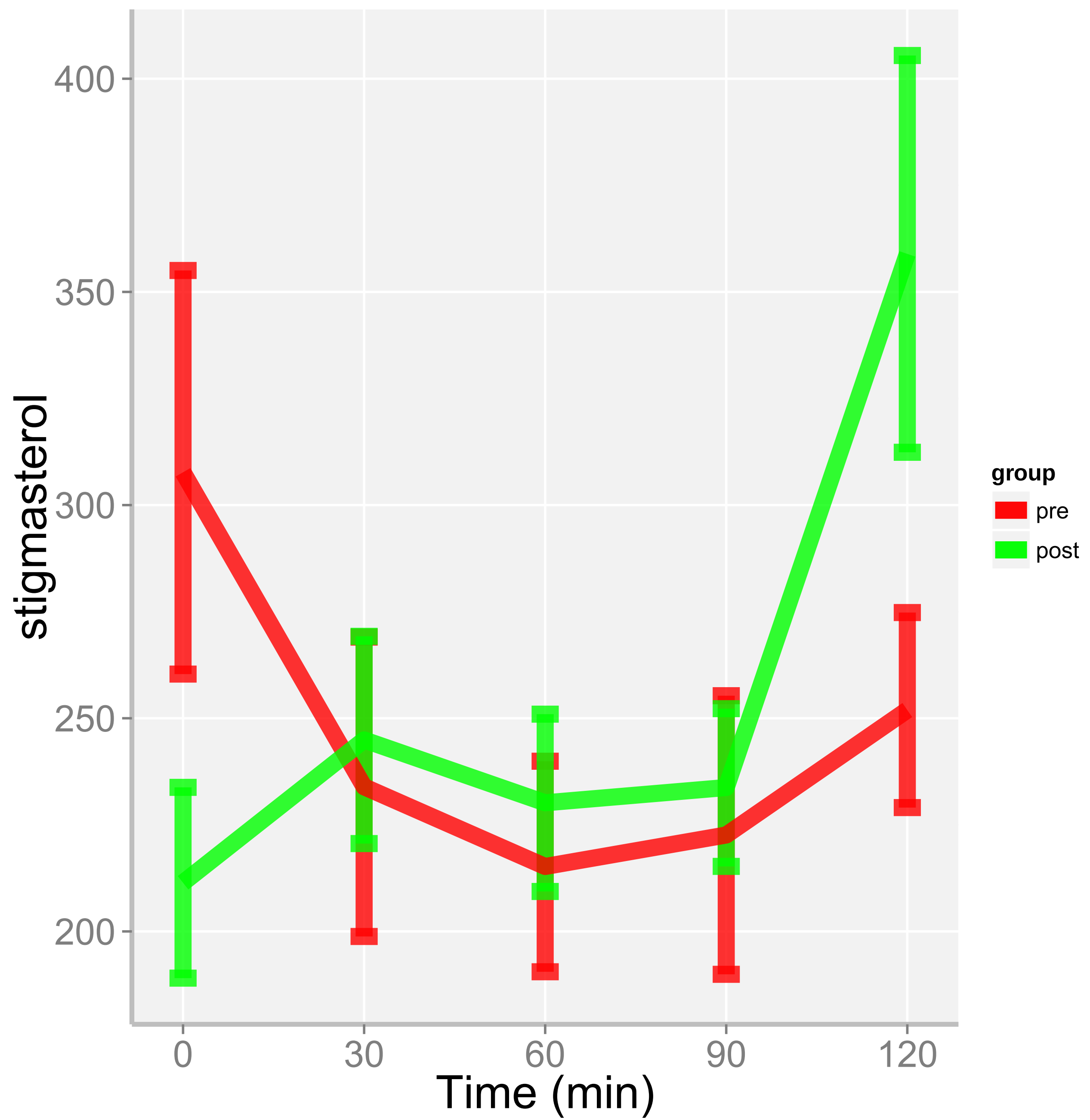

400
350
300
250
200
stigmasterol
0
30
60
90
120
Time (min)
group
pre
post

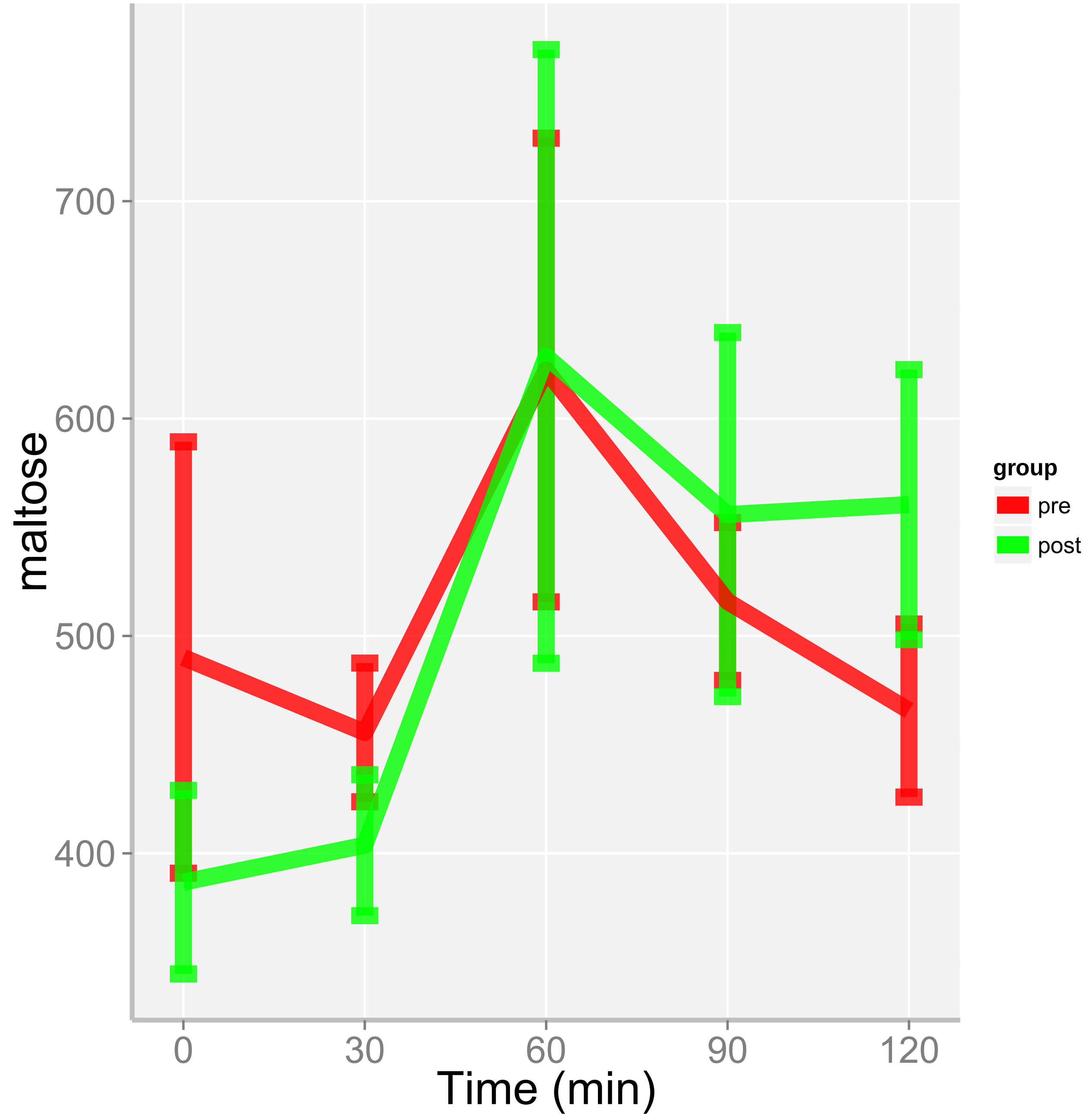

maltose
700
600
500
400
0
30
60
90
120
Time (min)
group
pre
post

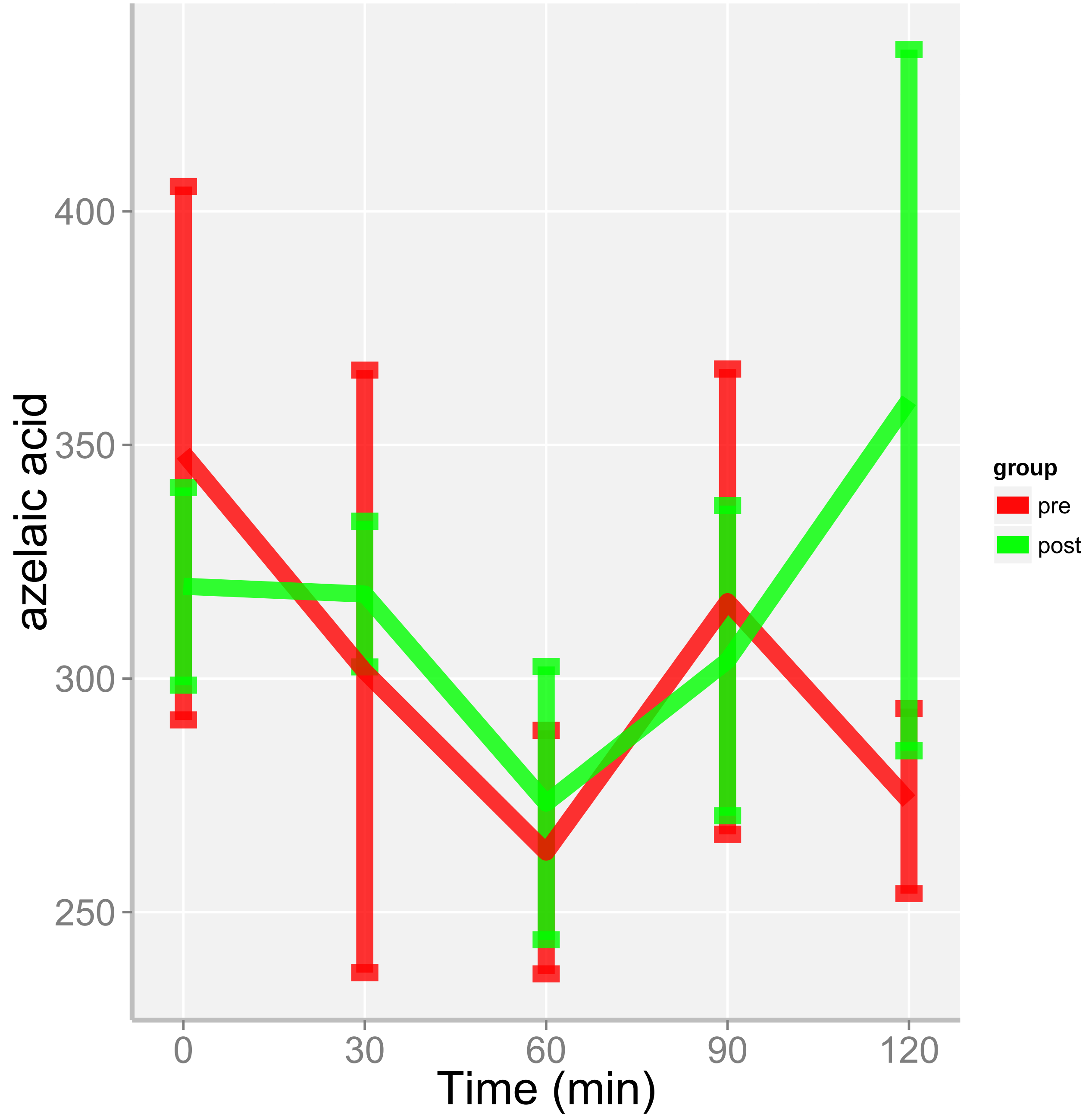

azelaic acid
400
350
300
250
0
30
60
90
120
Time (min)
group
pre
post

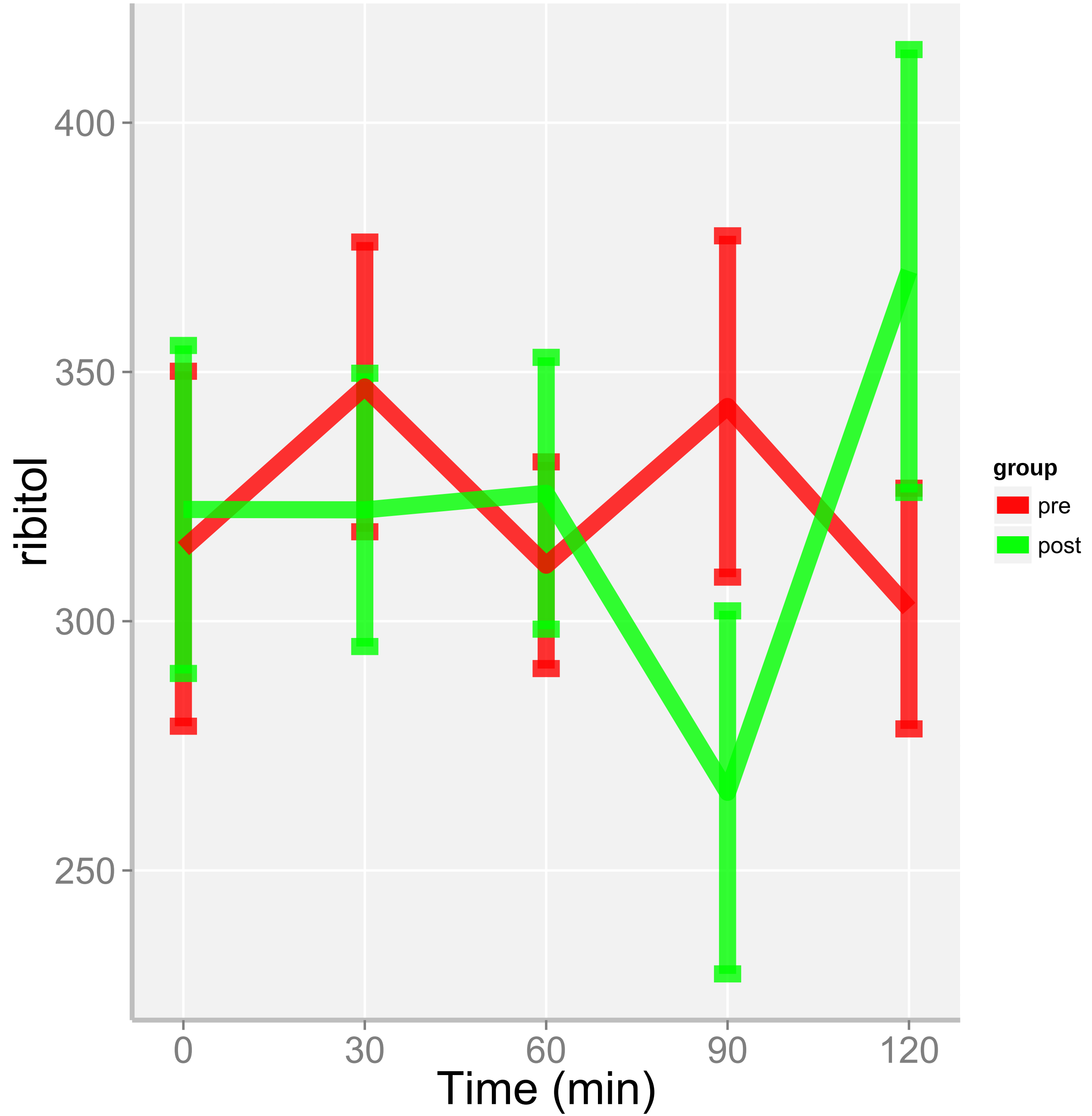
ribitol
400
350
300
250
0
30
60
90
120
Time (min)
group
pre
post

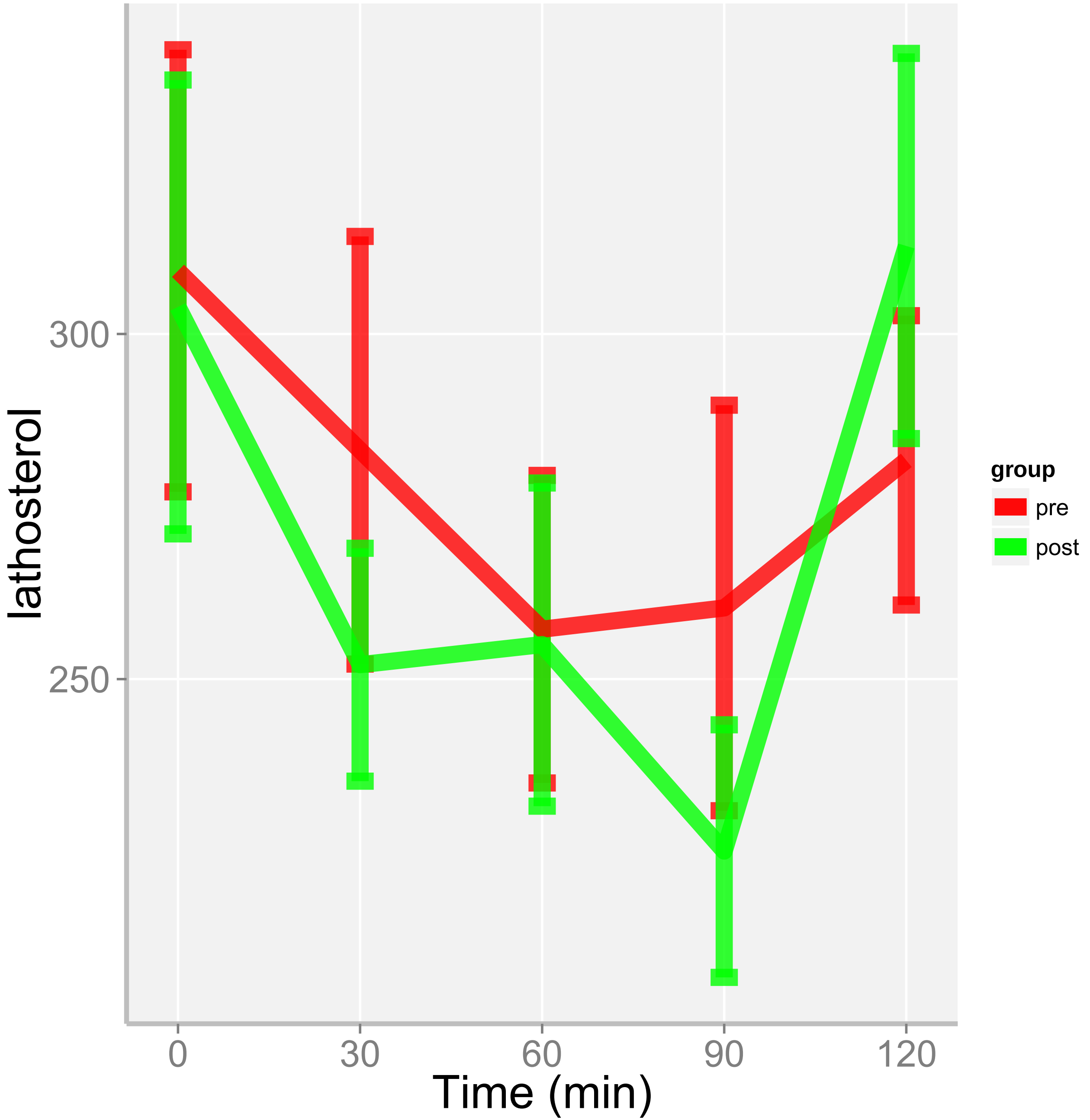

lathosterol
300
250
0
30
60
90
120
Time (min)
group
pre
post

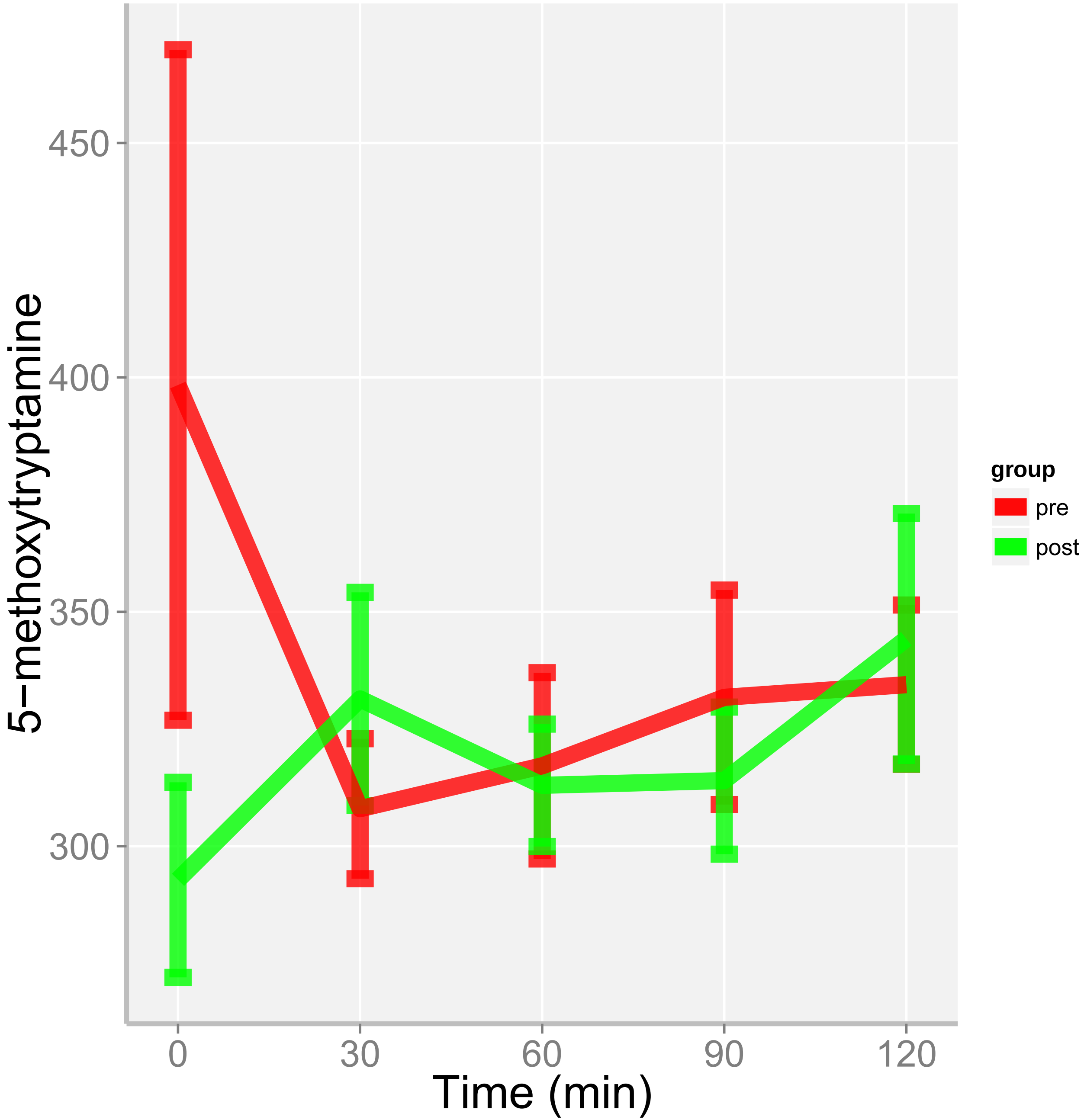

5−methoxytryptamine
450
400
350
300
0
30
60
90
120
Time (min)
group
pre
post

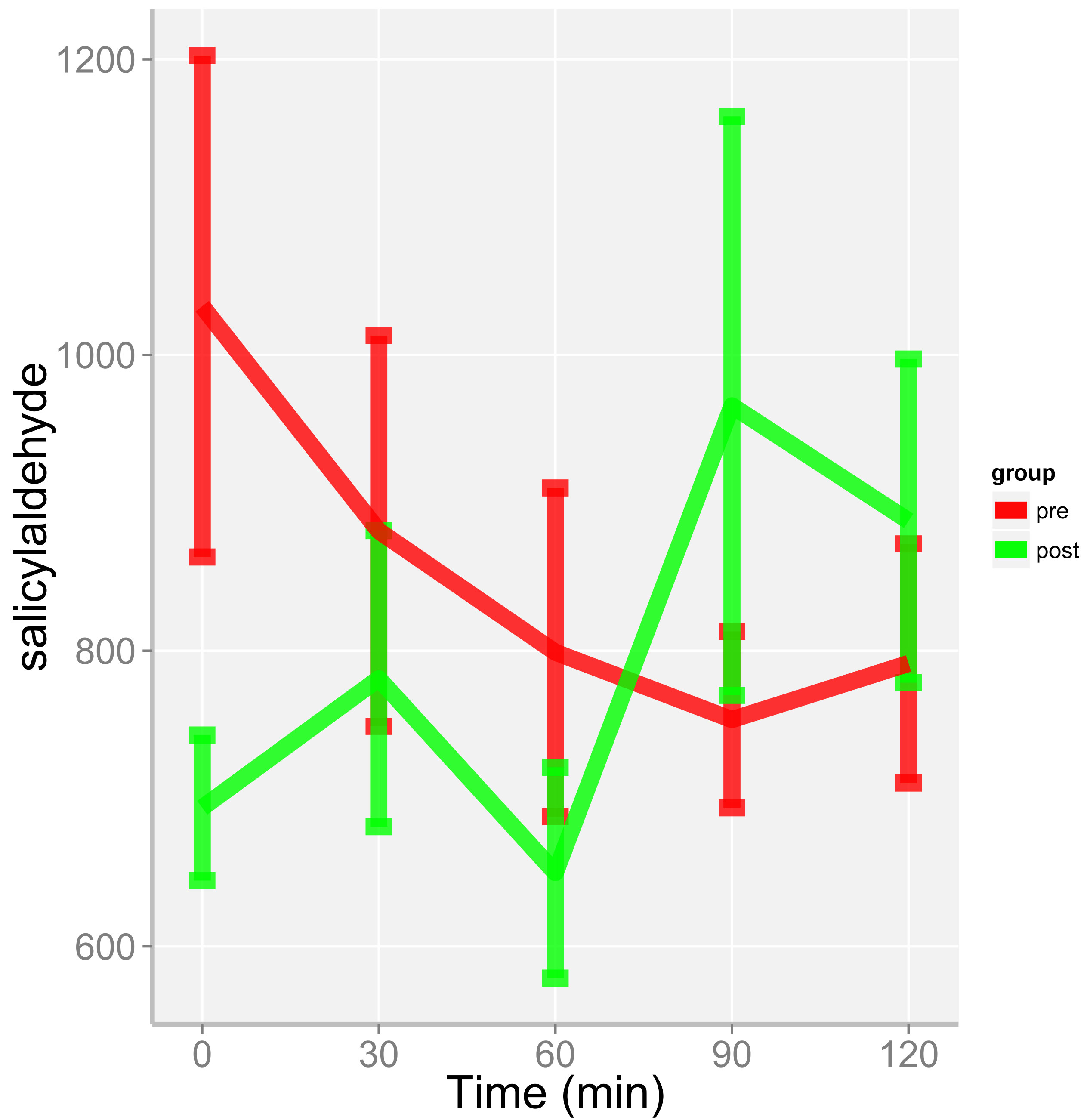

salicylaldehyde
1200
1000
800
600
0
30
60
90
120
Time (min)
group
pre
post

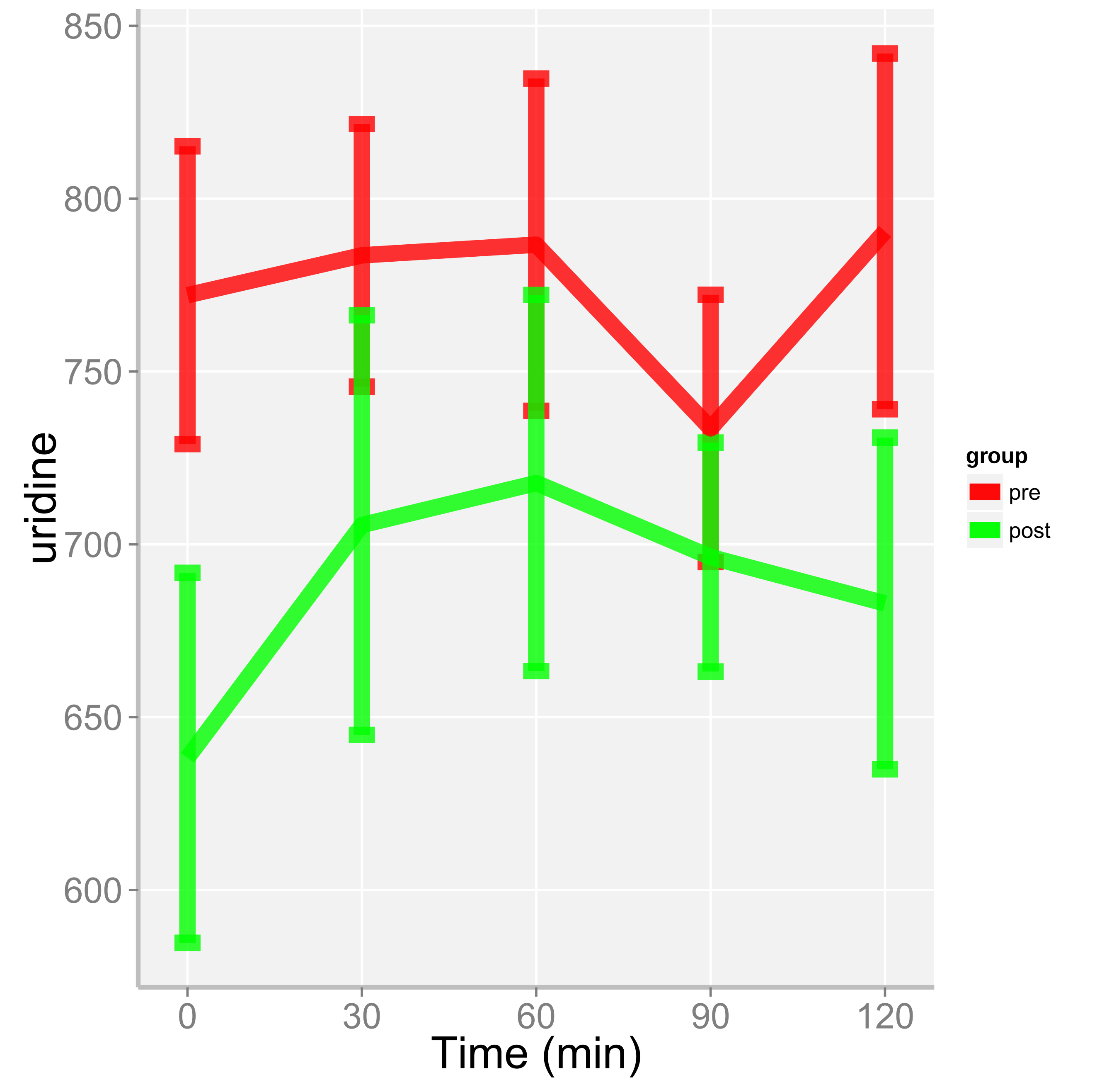

uridine
850
800
750
700
650
600
0
30
60
90
120
Time (min)
group
pre
post

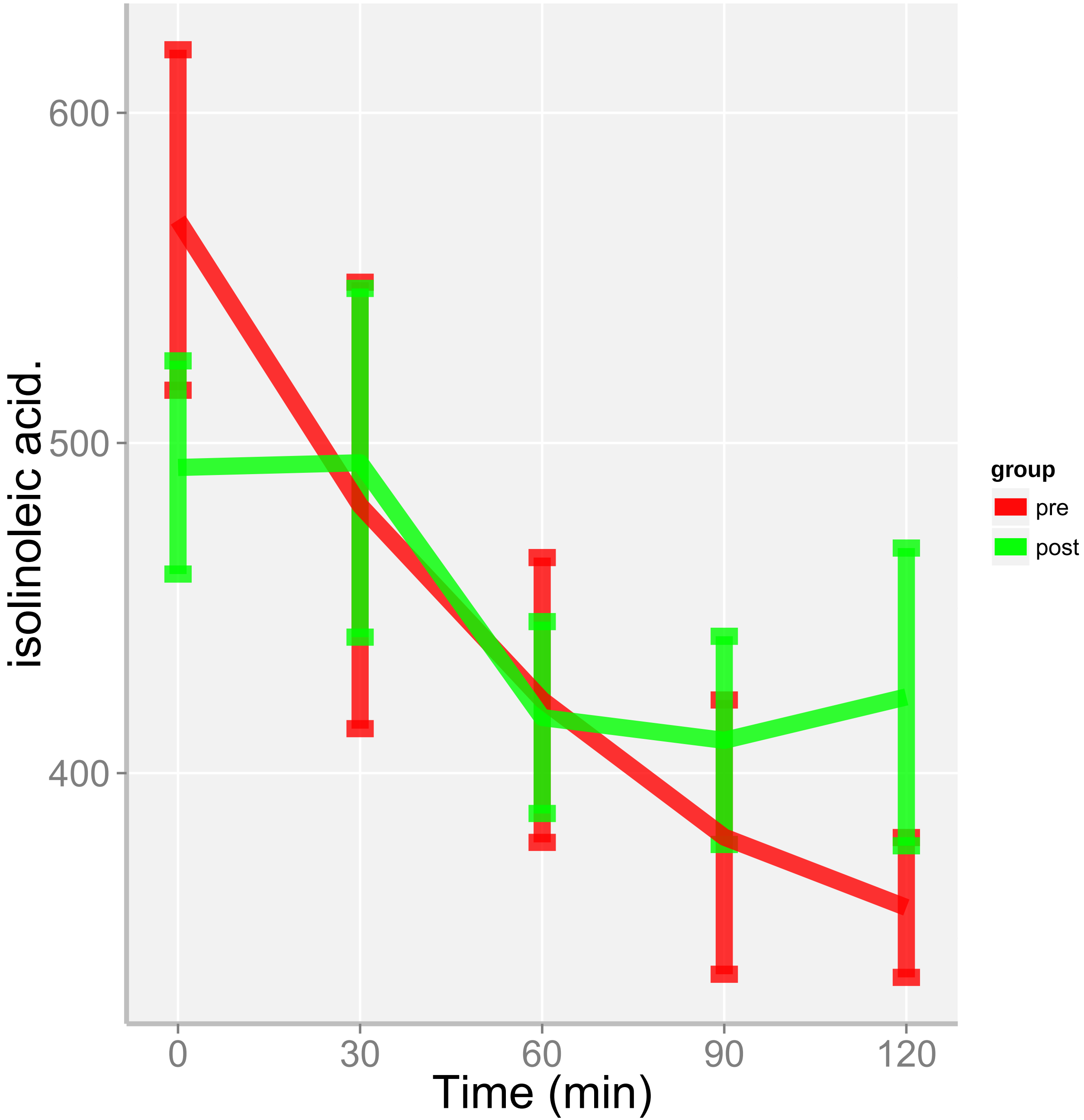

isolinoleic acid.
600
500
400
0
30
60
90
120
Time (min)
group
pre
post

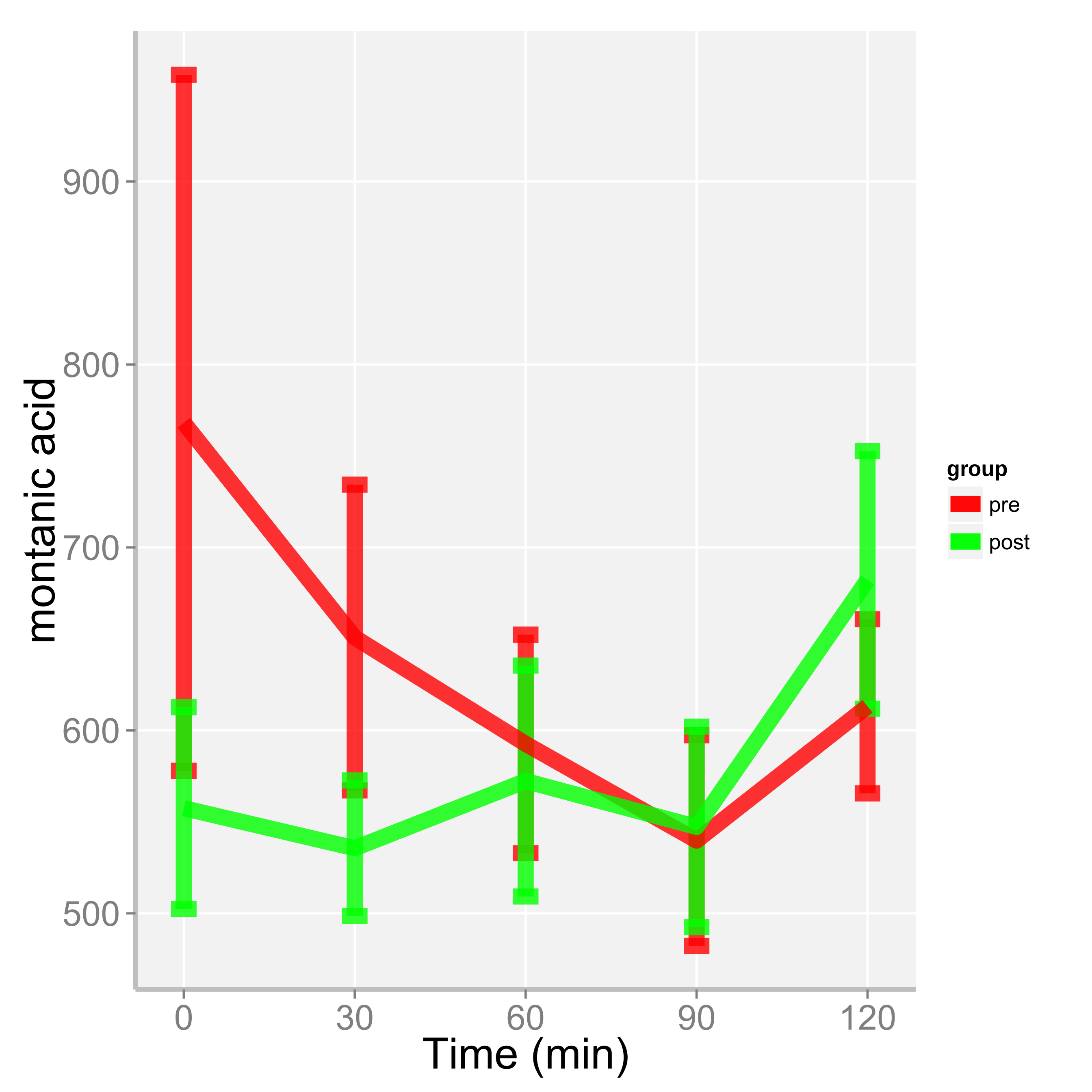

montanic acid
900
800
700
600
500
0
30
60
90
120
Time (min)
group
pre
post

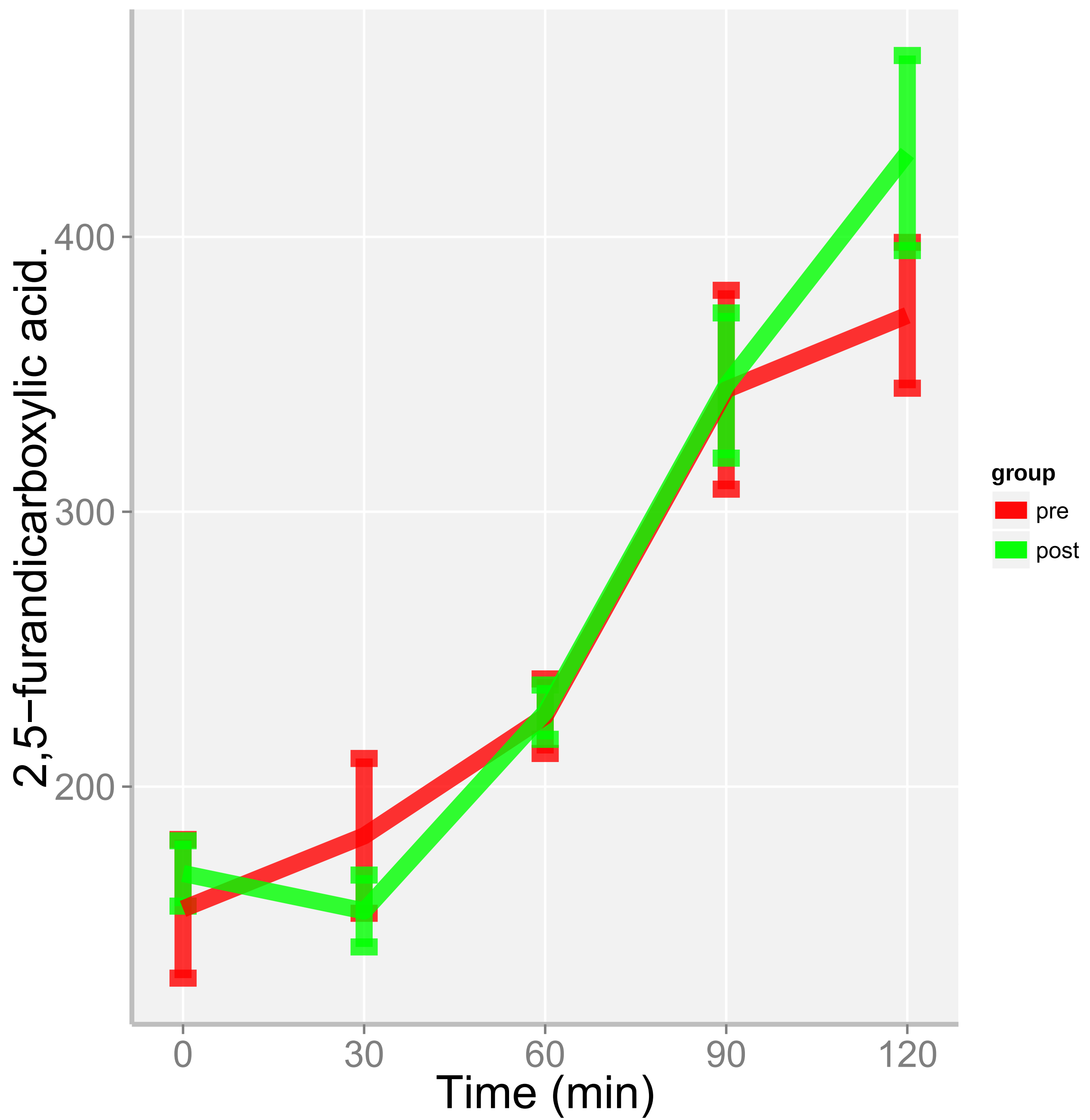

2,5−furandicarboxylic acid.
400
300
200
0
30
60
90
120
Time (min)
group
pre
post

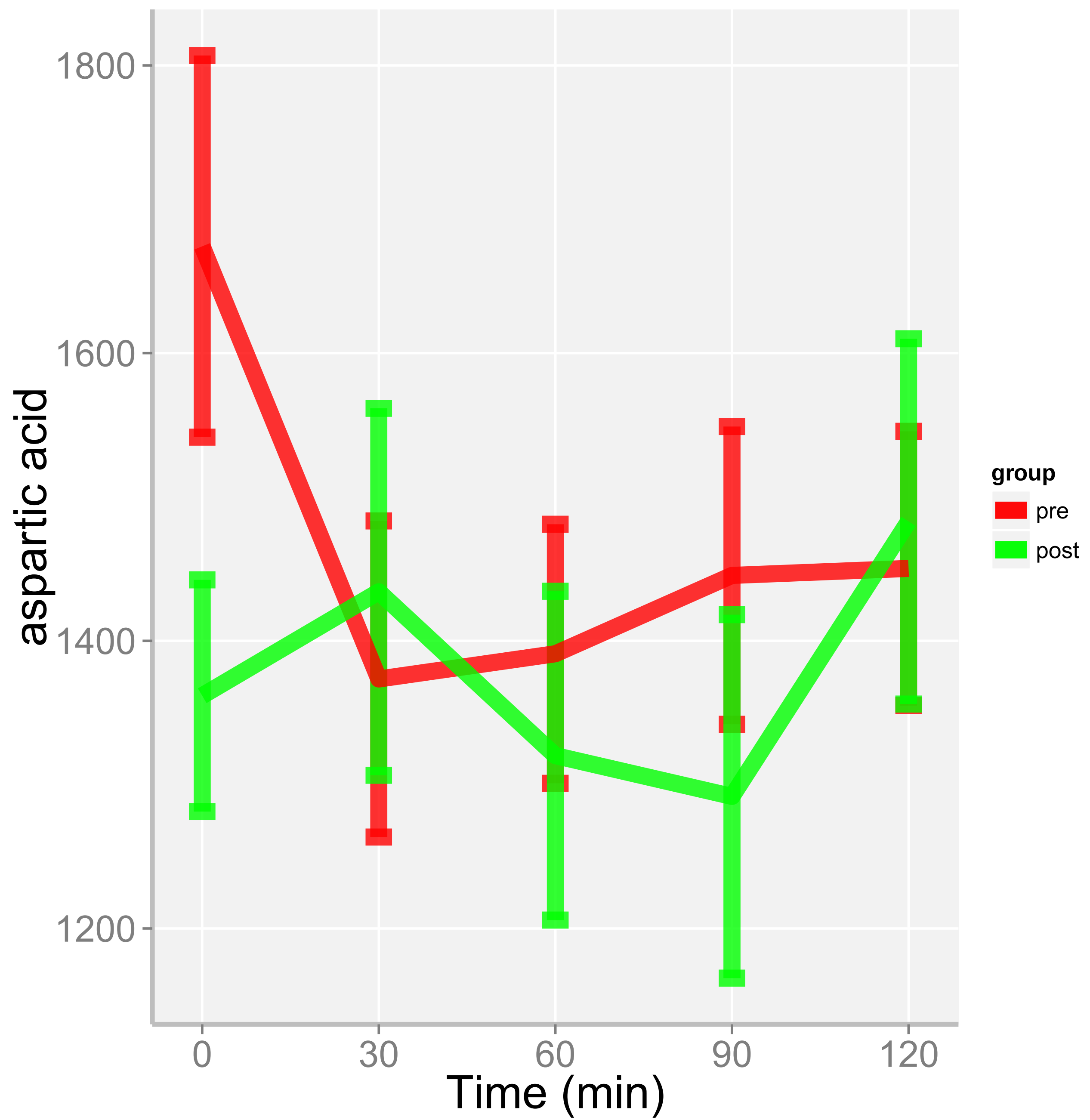

aspartic acid
1800
1600
1400
1200
0
30
60
90
120
Time (min)
group
pre
post

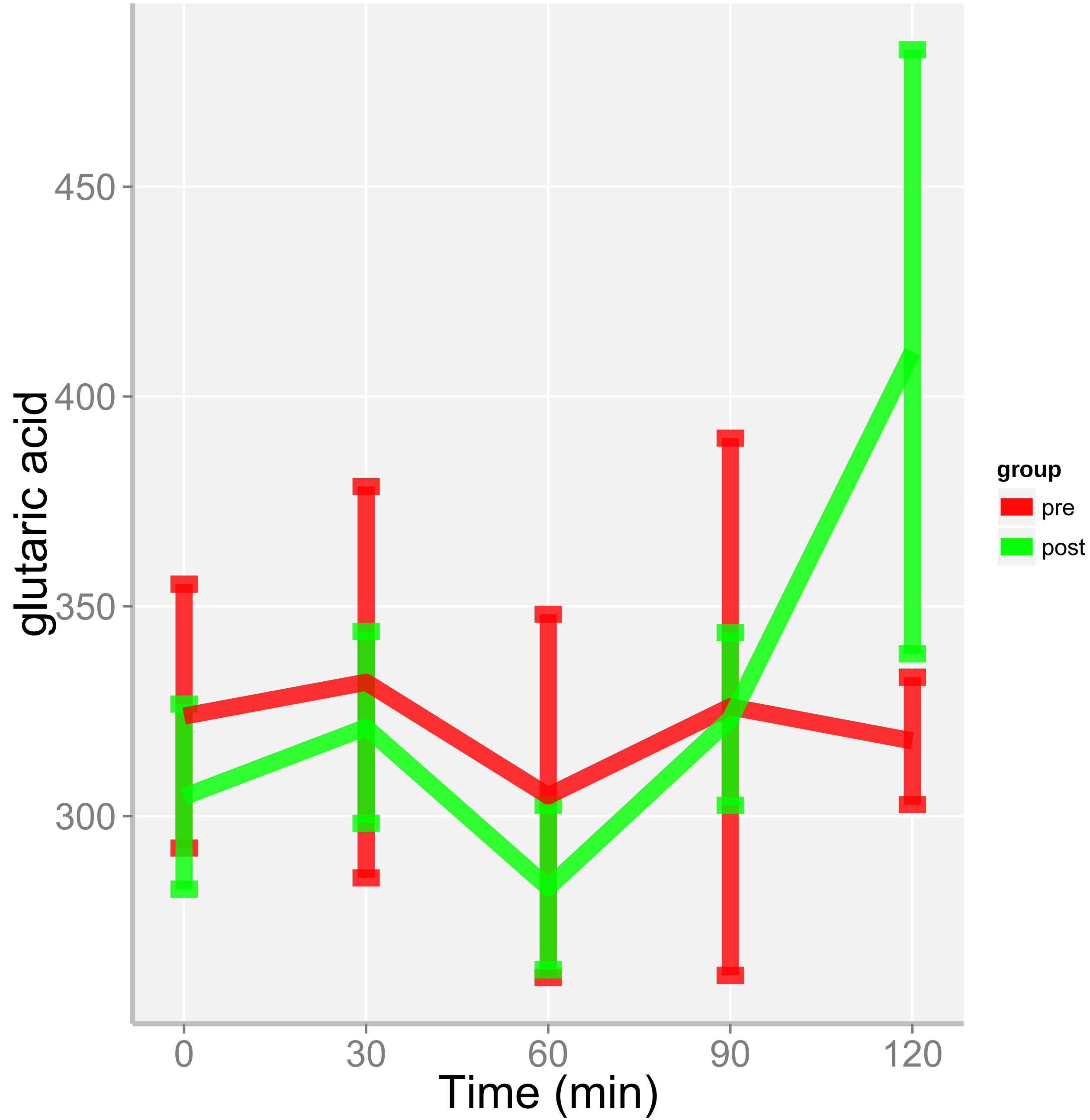

glutaric acid
450
400
350
300
0
30
60
90
120
Time (min)
group
pre
post

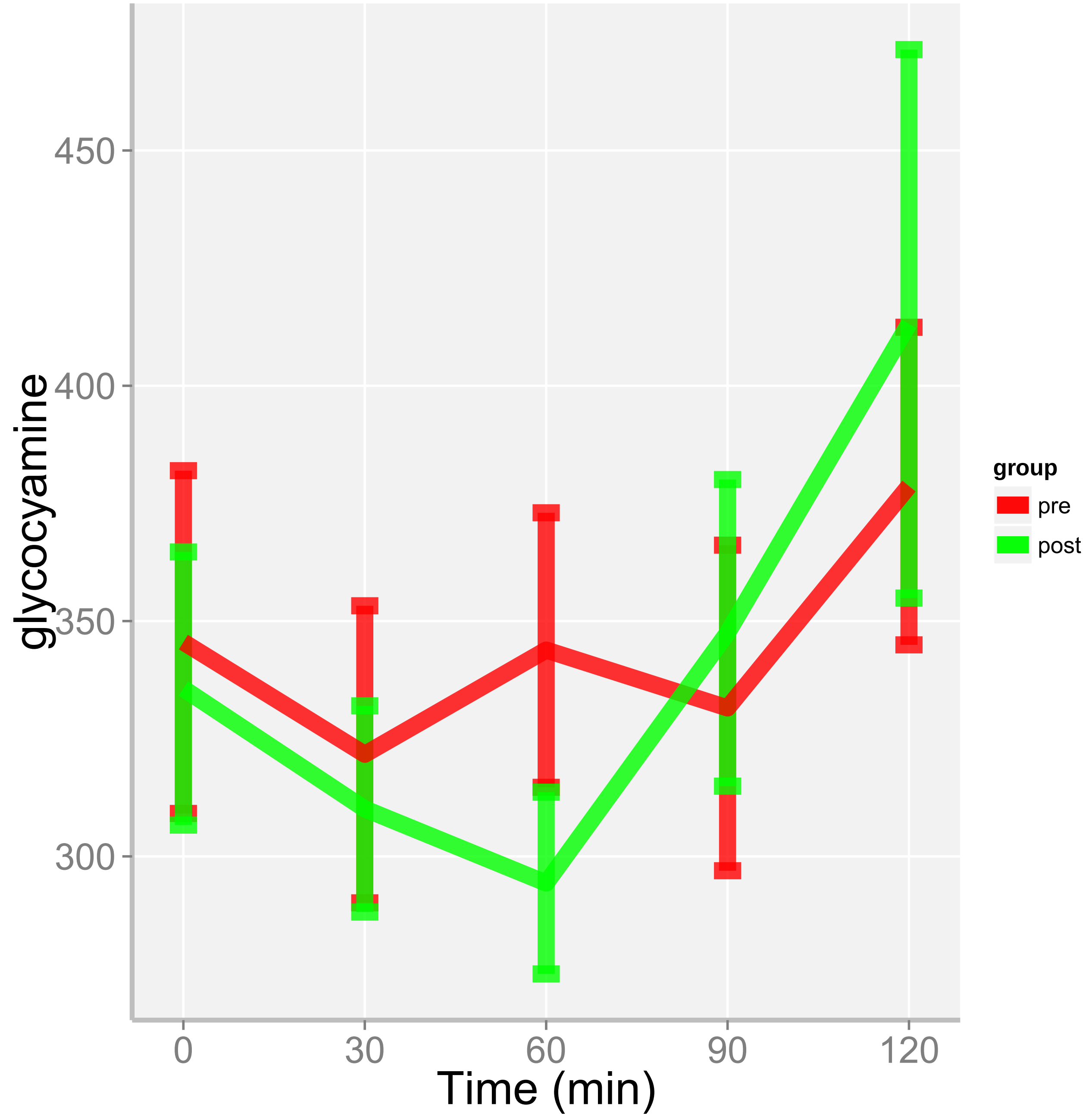

glycocyamine
450
400
350
300
0
30
60
90
120
Time (min)
group
pre
post

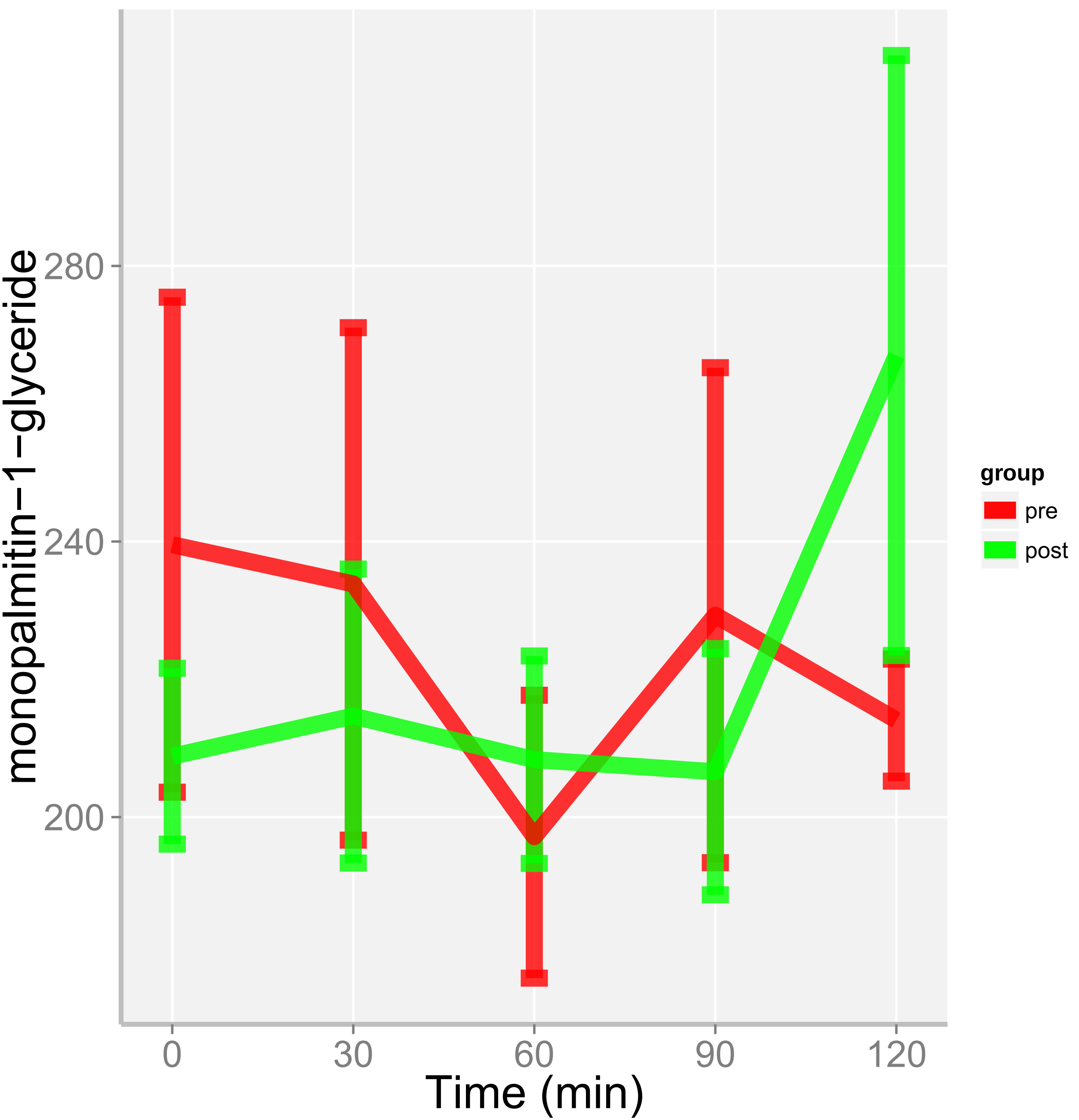

monopalmitin−1−glyceride
280
240
200
0
30
60
90
120
Time (min)
group
pre
post

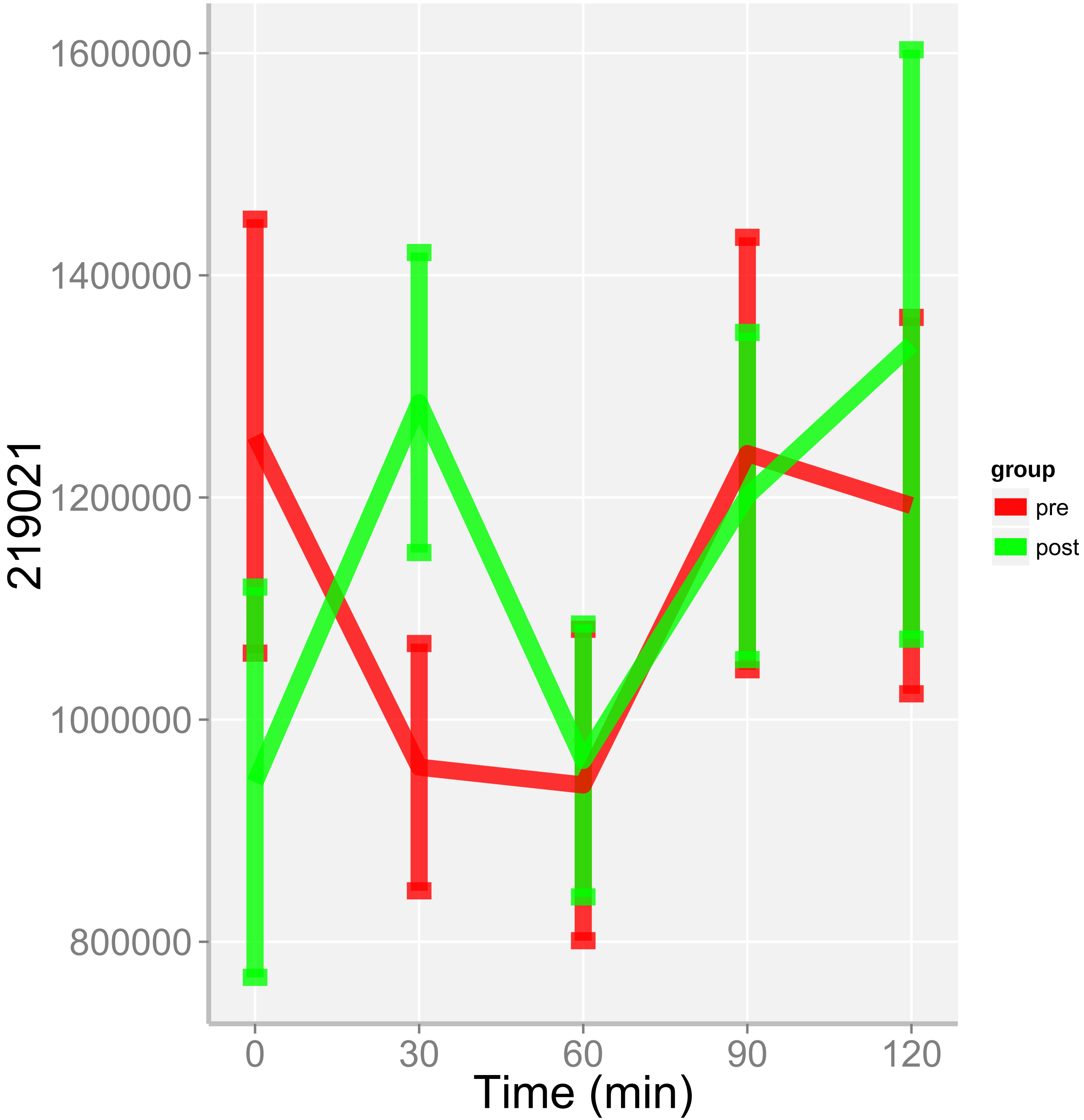

1600000
1400000
1200000
1000000
800000
219021
0
30
60
90
120
Time (min)
group
pre
post

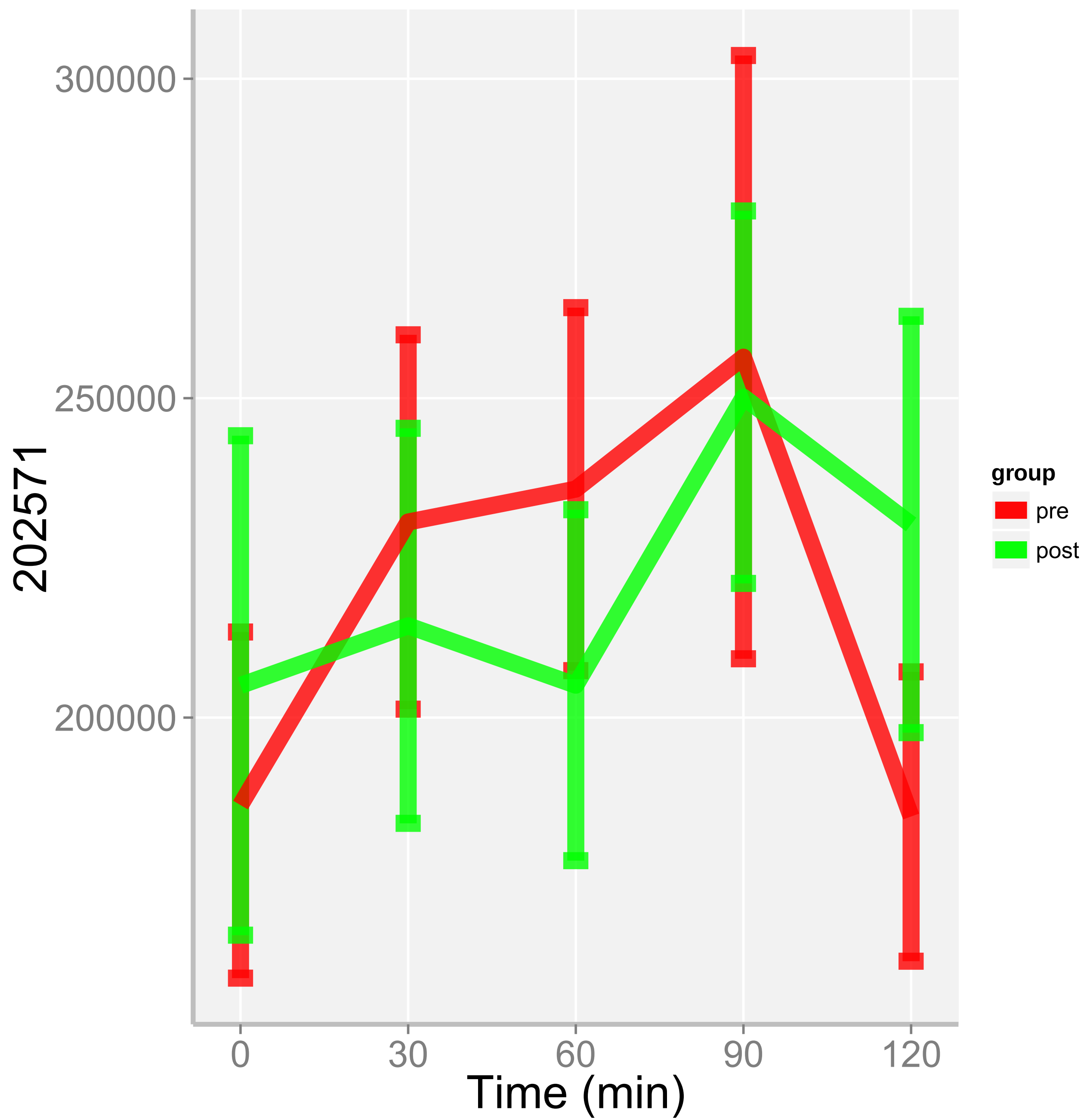

300000
250000
200000
202571
0
30
60
90
120
Time (min)
group
pre
post

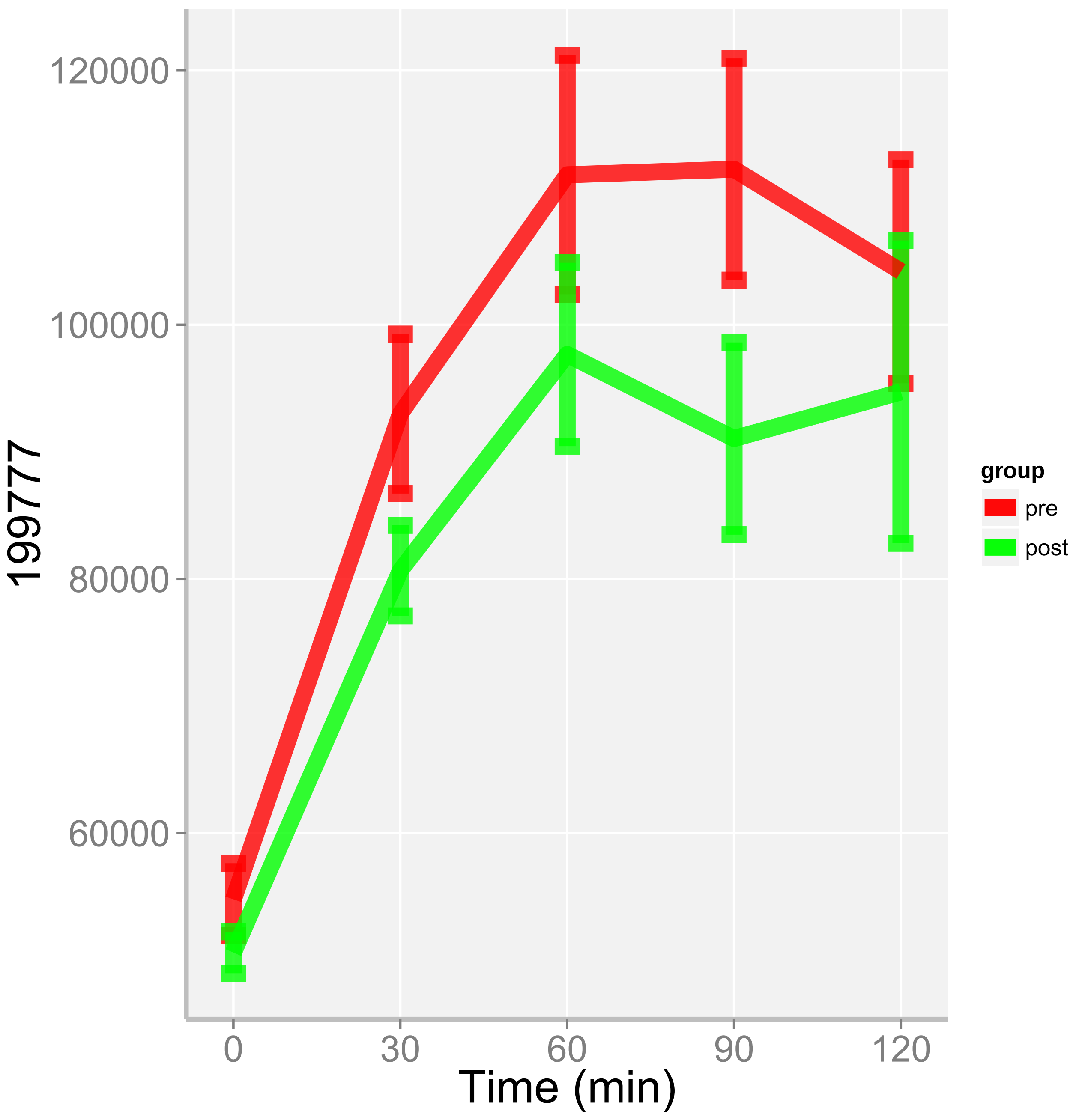

199777
120000
100000
80000
60000
0
30
60
90
120
Time (min)
group
pre
post

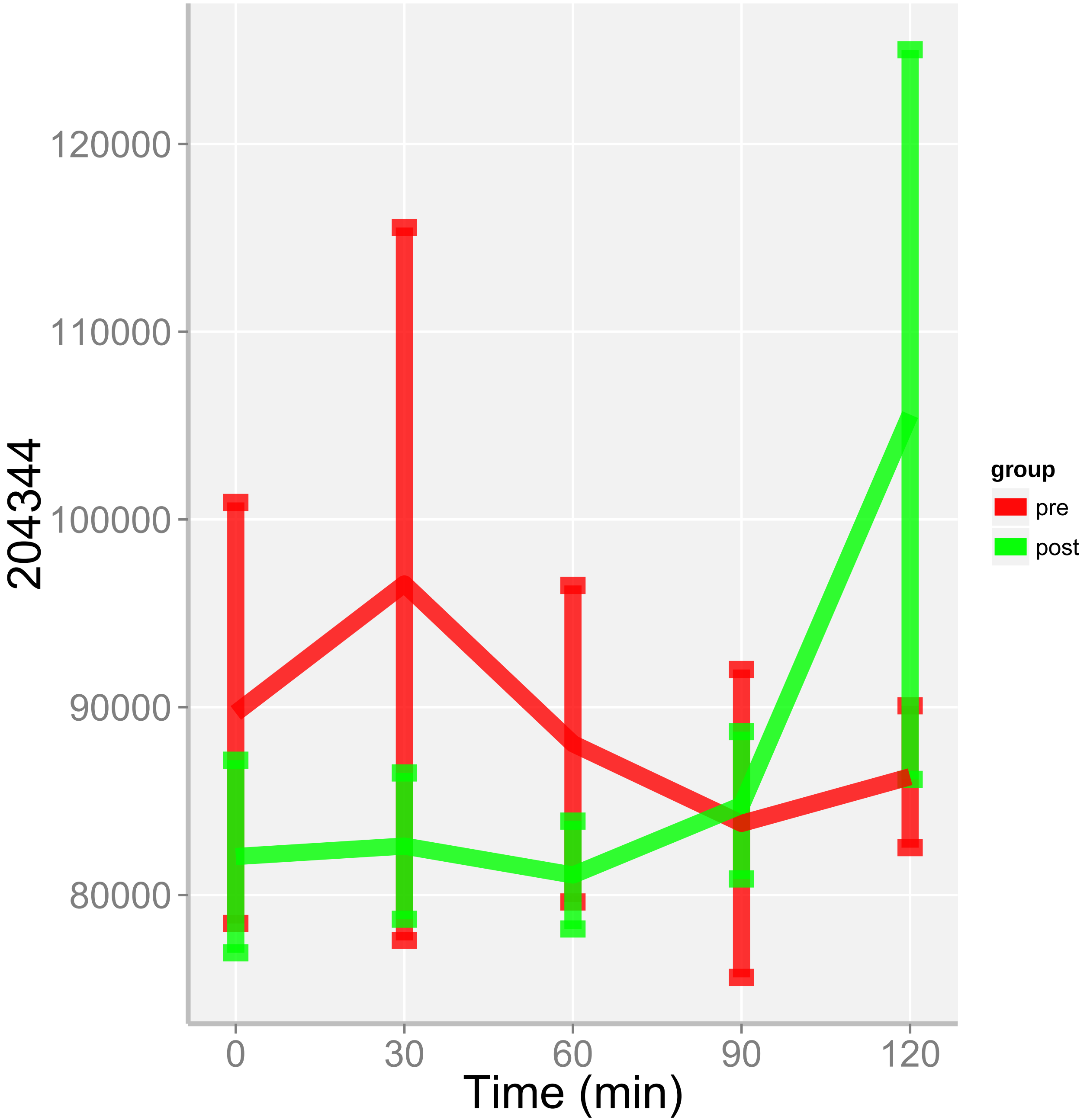

2043344
120000
110000
100000
90000
80000
0
30
60
90
120
Time (min)
group
pre
post

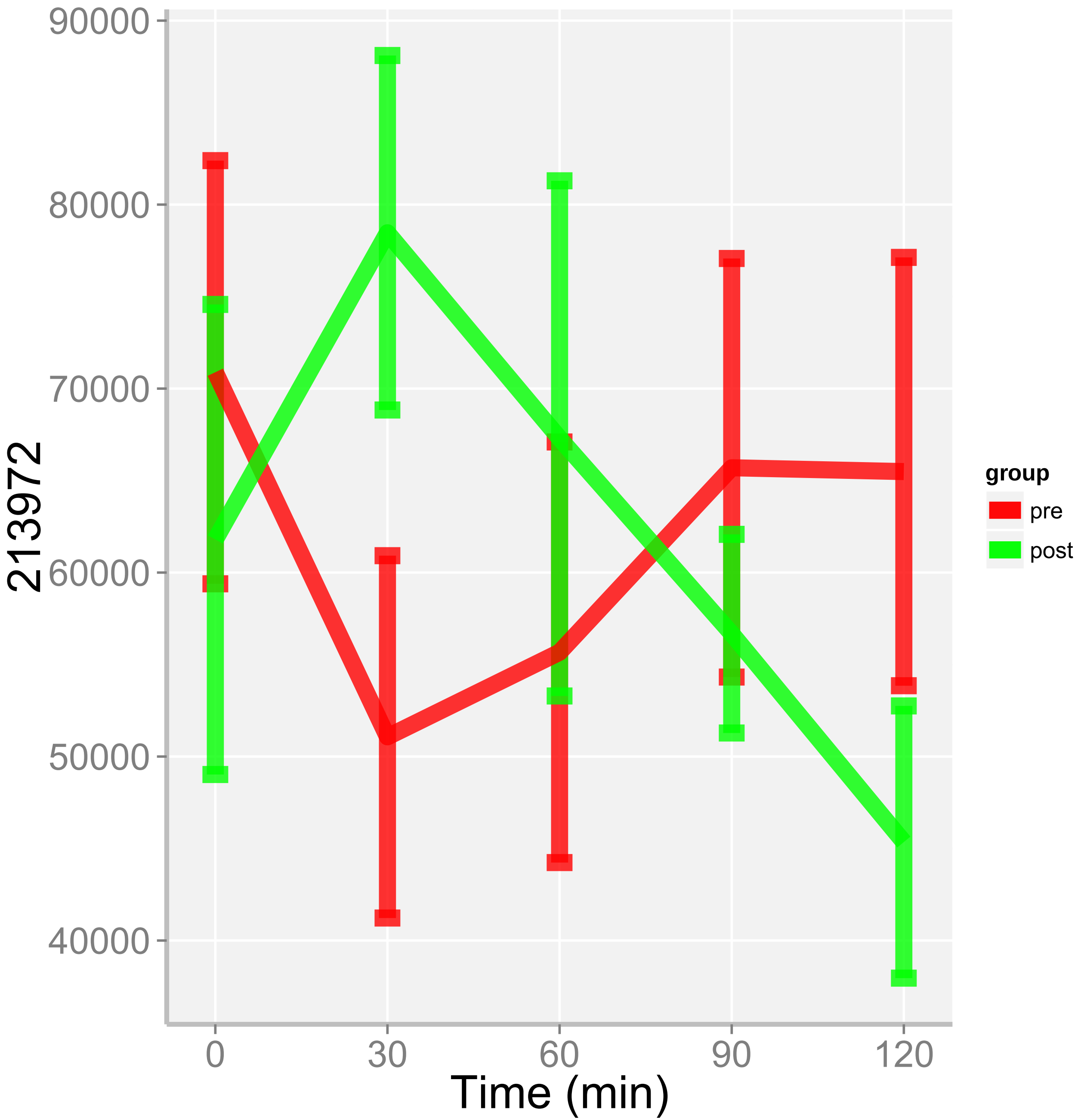

90000
80000
70000
60000
50000
40000
213972
0
30
60
90
120
Time (min)
group
pre
post

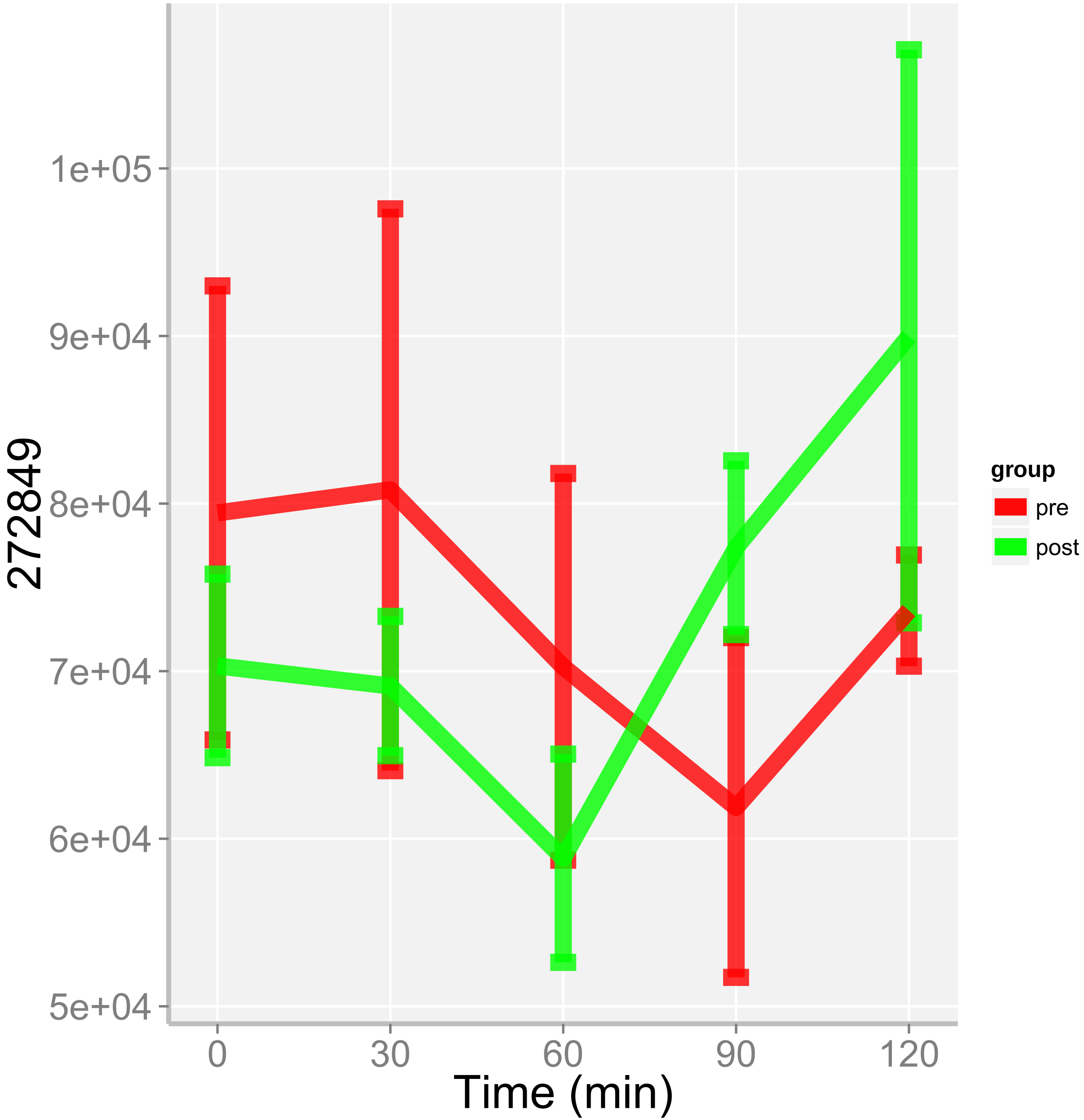

1e+05
9e+04
8e+04
7e+04
6e+04
5e+04
272849
0
30
60
90
120
Time (min)
group
pre
post

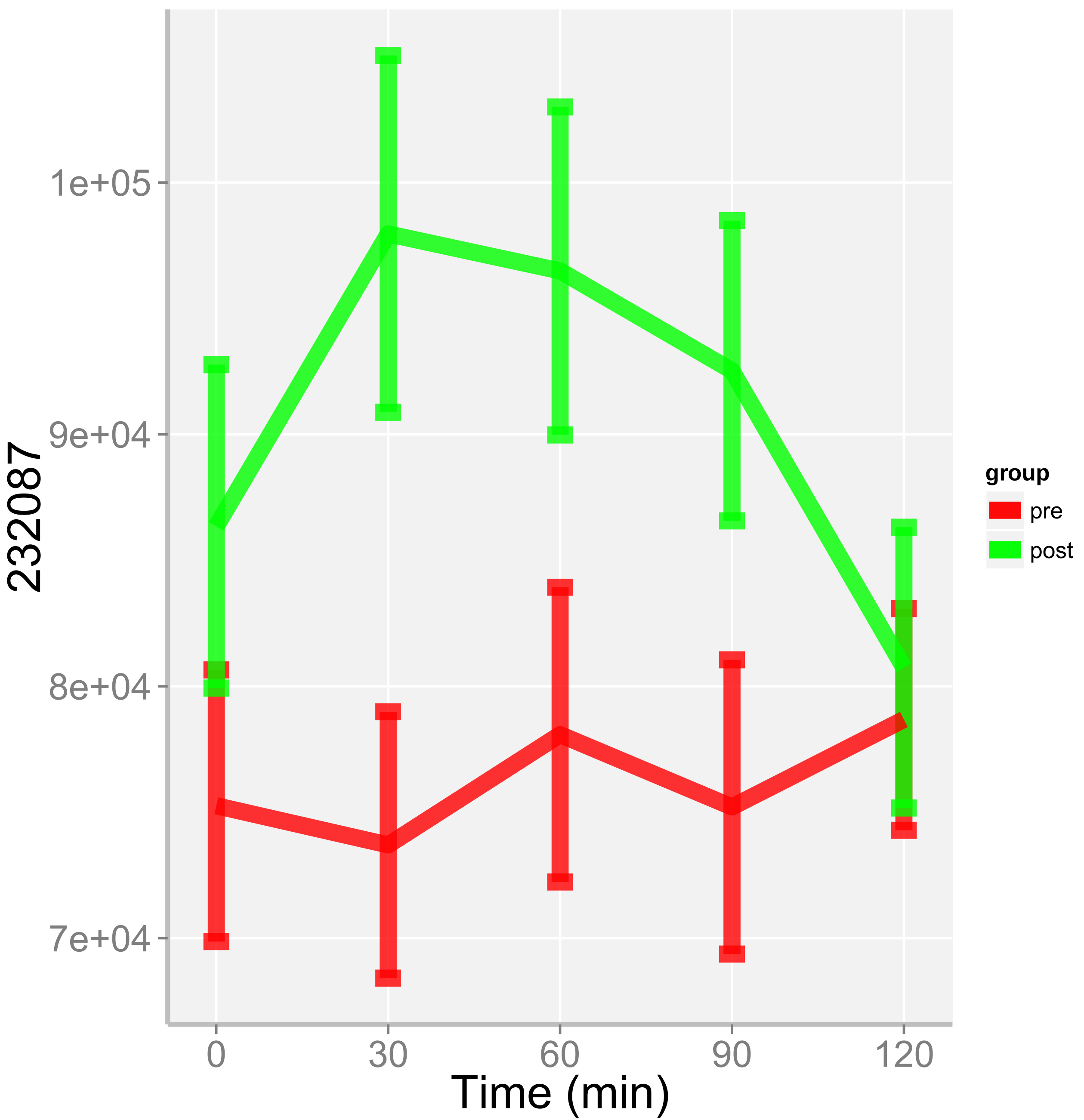
232087
1e+05
9e+04
8e+04
7e+04
0
30
60
90
120
Time (min)
group
pre
post

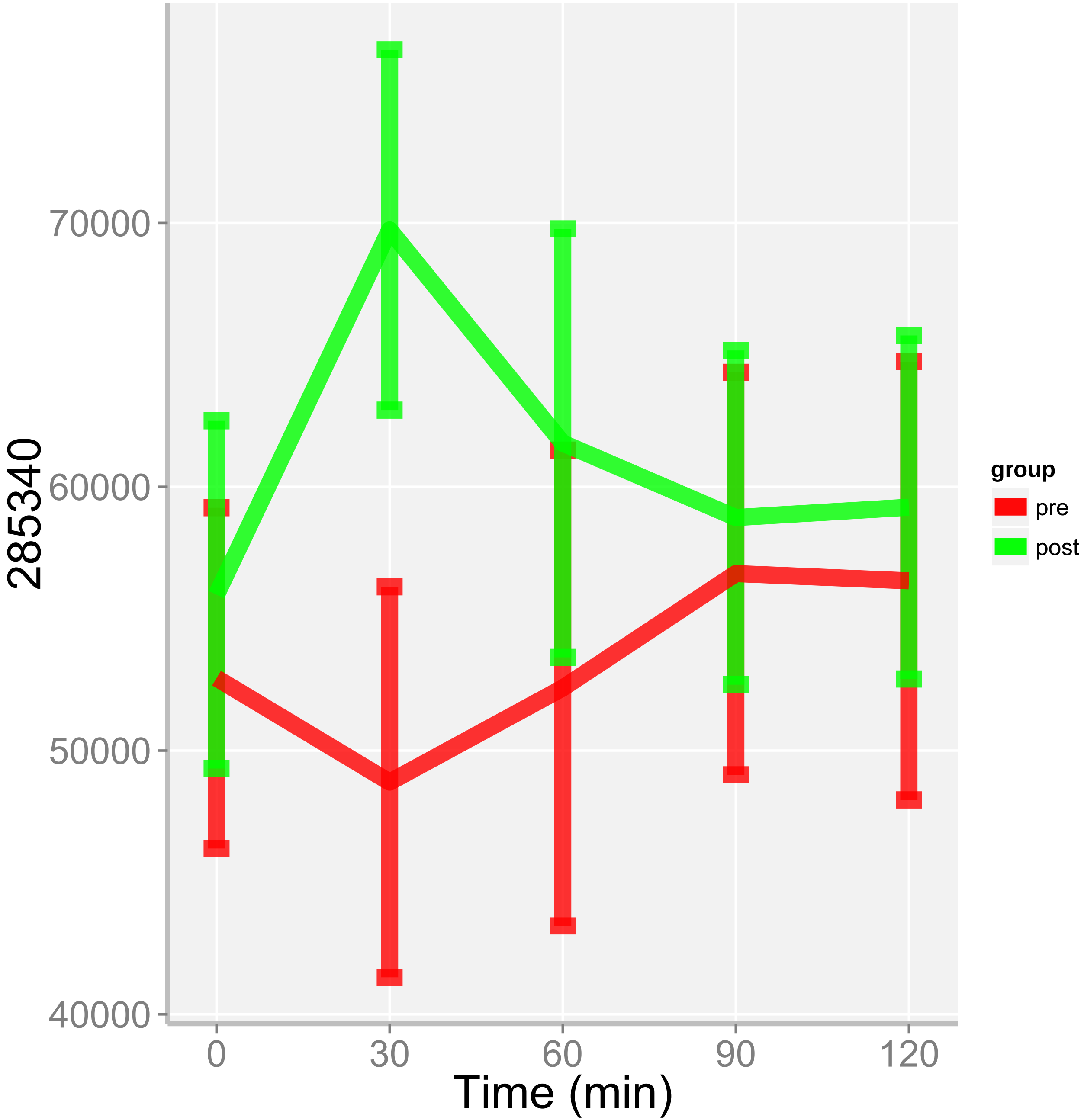

285340
70000
60000
50000
40000
0
30
60
90
120
Time (min)
group
pre
post

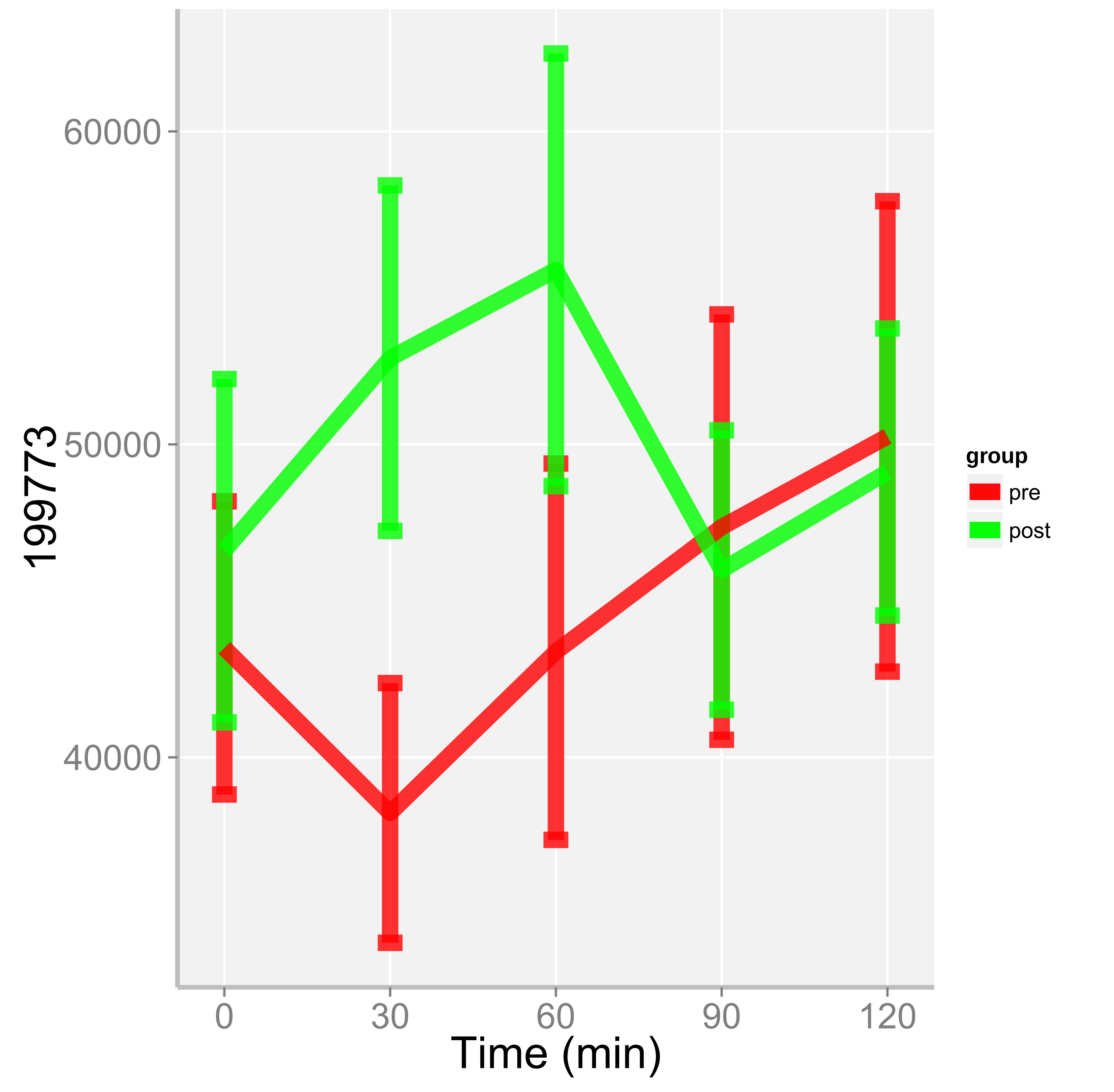

199773
60000
50000
40000
0
30
60
90
120
Time (min)
group
pre
post

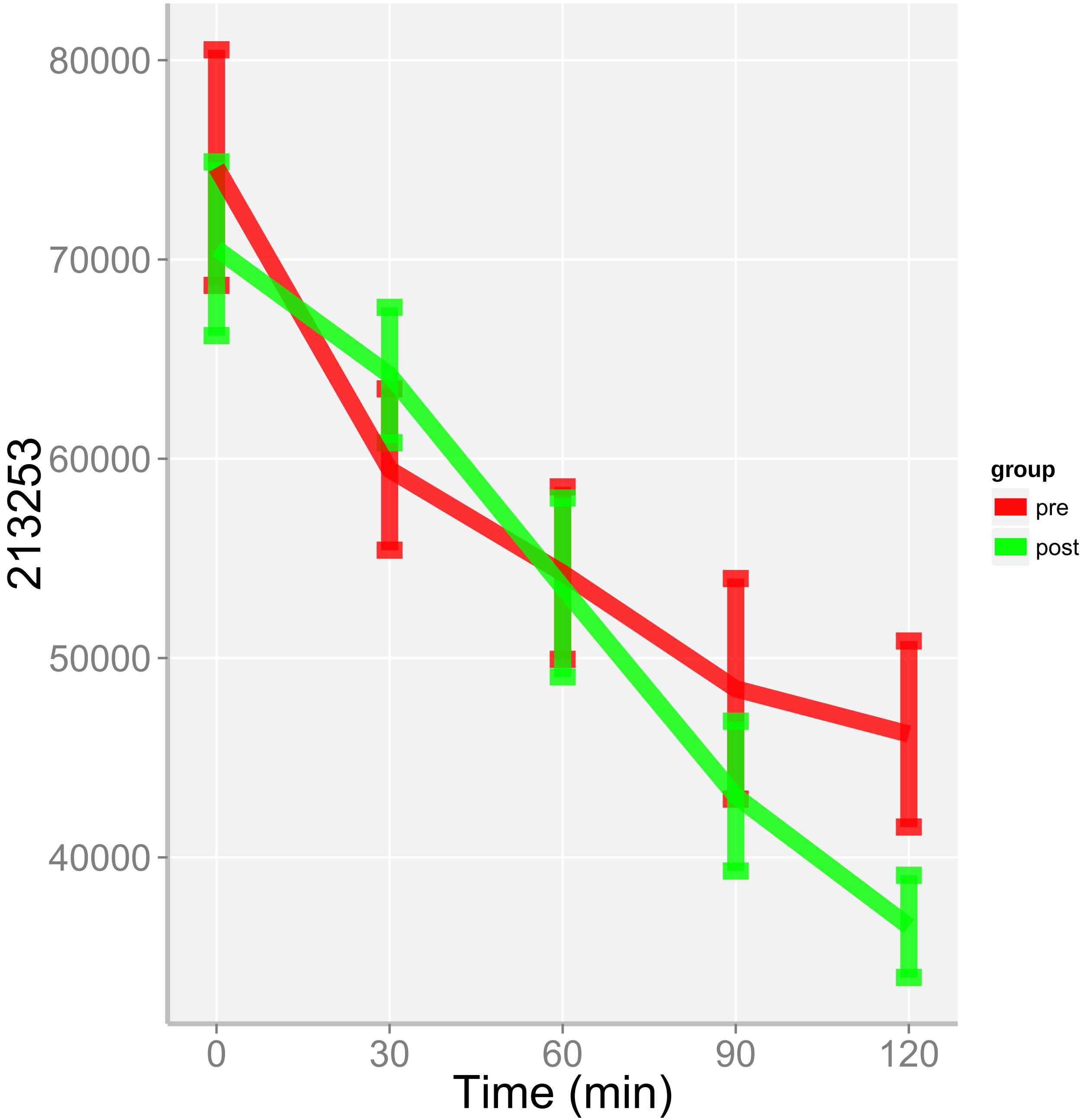

213253
80000
70000
60000
50000
40000
0
30
60
90
120
Time (min)
group
pre
post

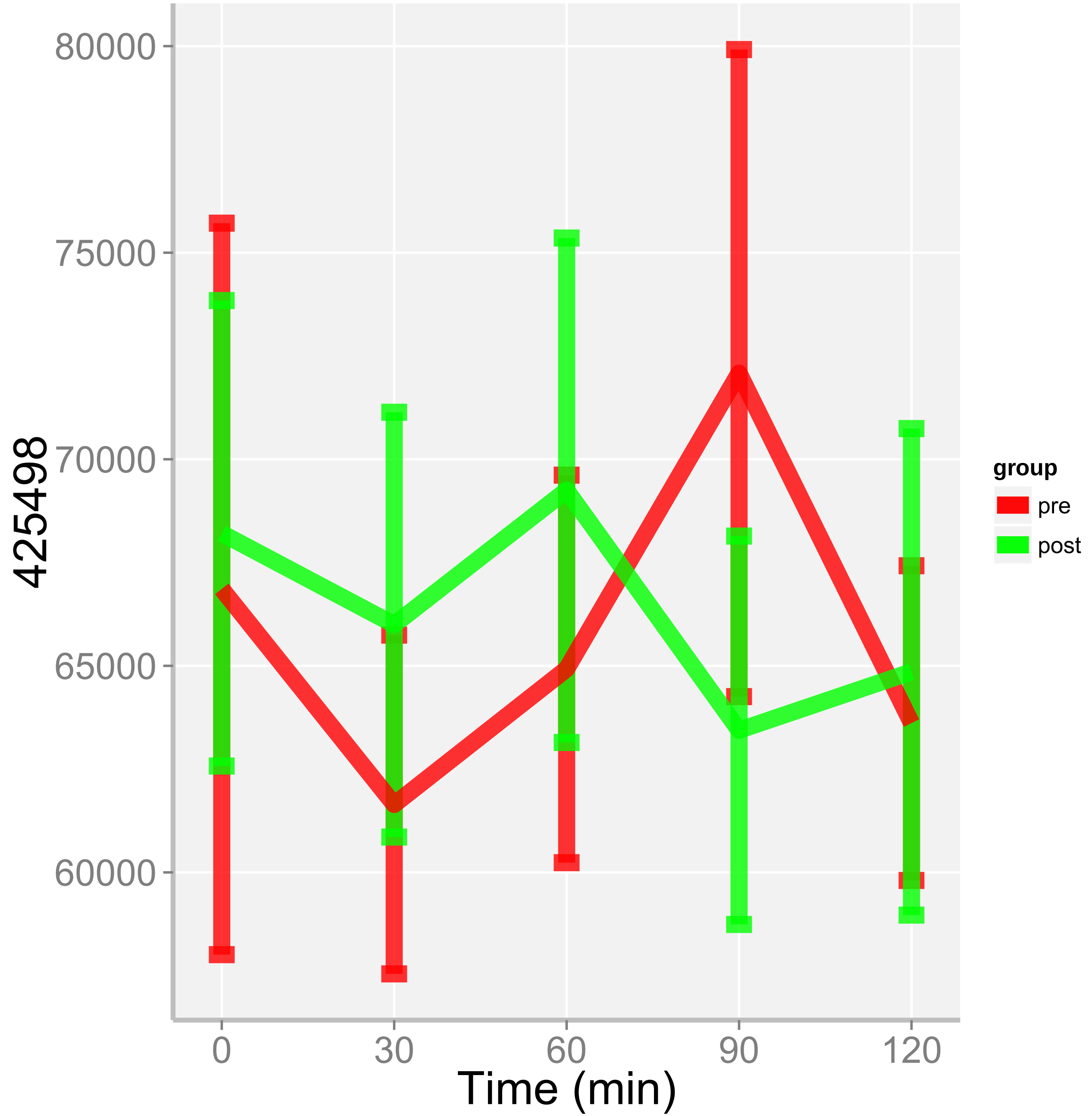

80000
75000
70000
65000
60000
425498
0
30
60
90
120
Time (min)
group
pre
post

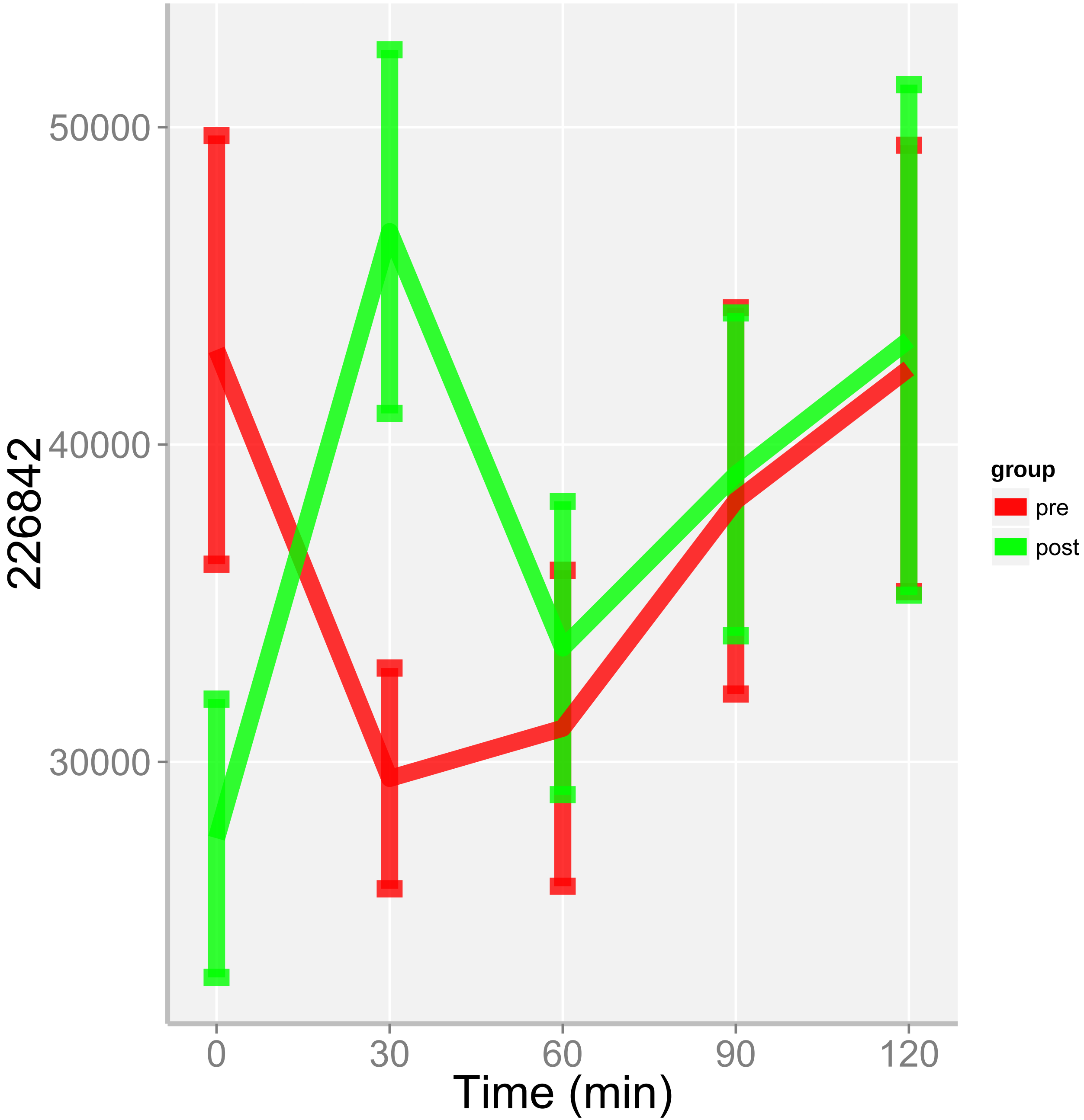

226842
50000
40000
30000
0
30
60
90
120
Time (min)
group
pre
post

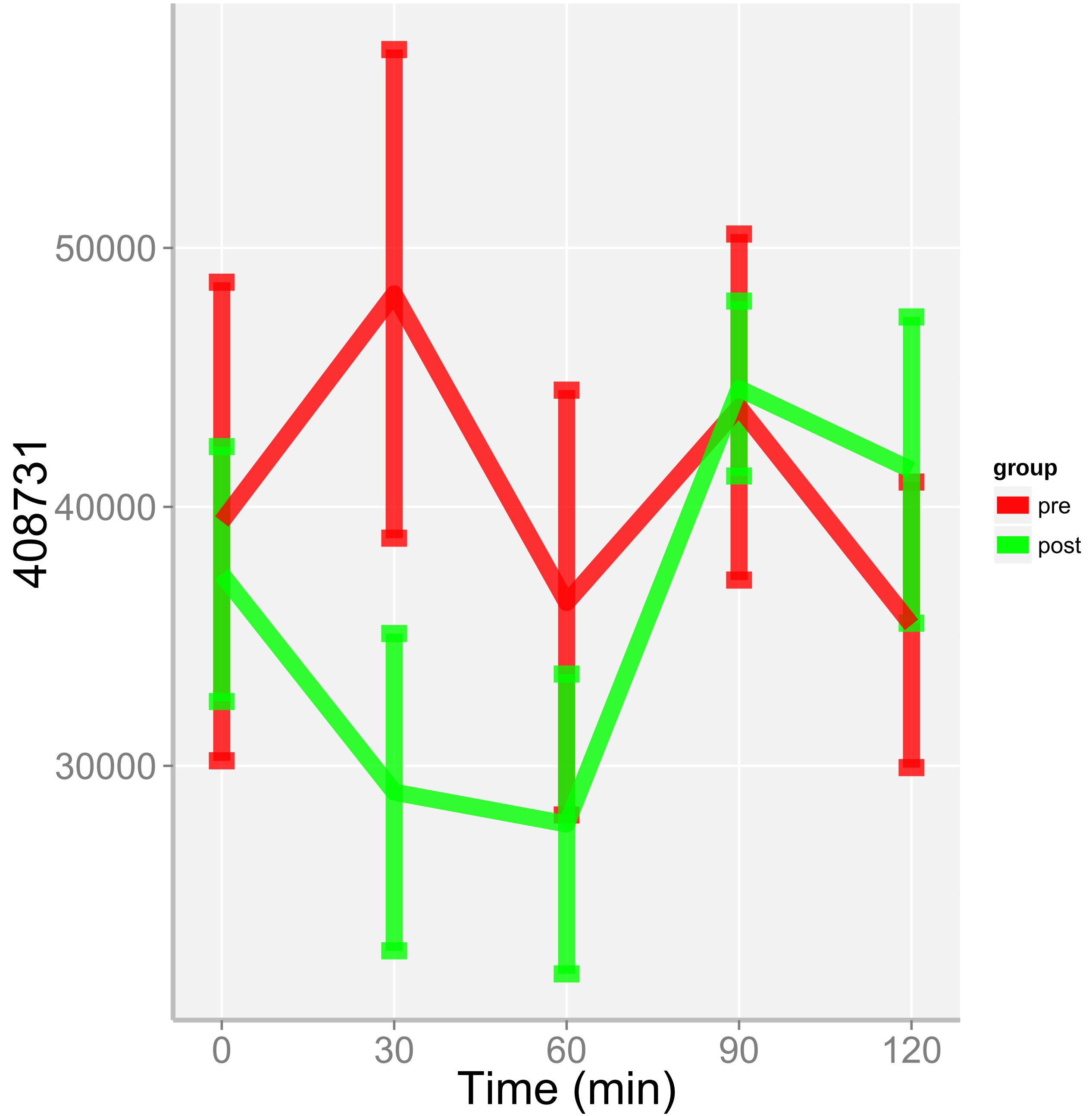

408731
50000
40000
30000
0
30
60
90
120
Time (min)
group
pre
post

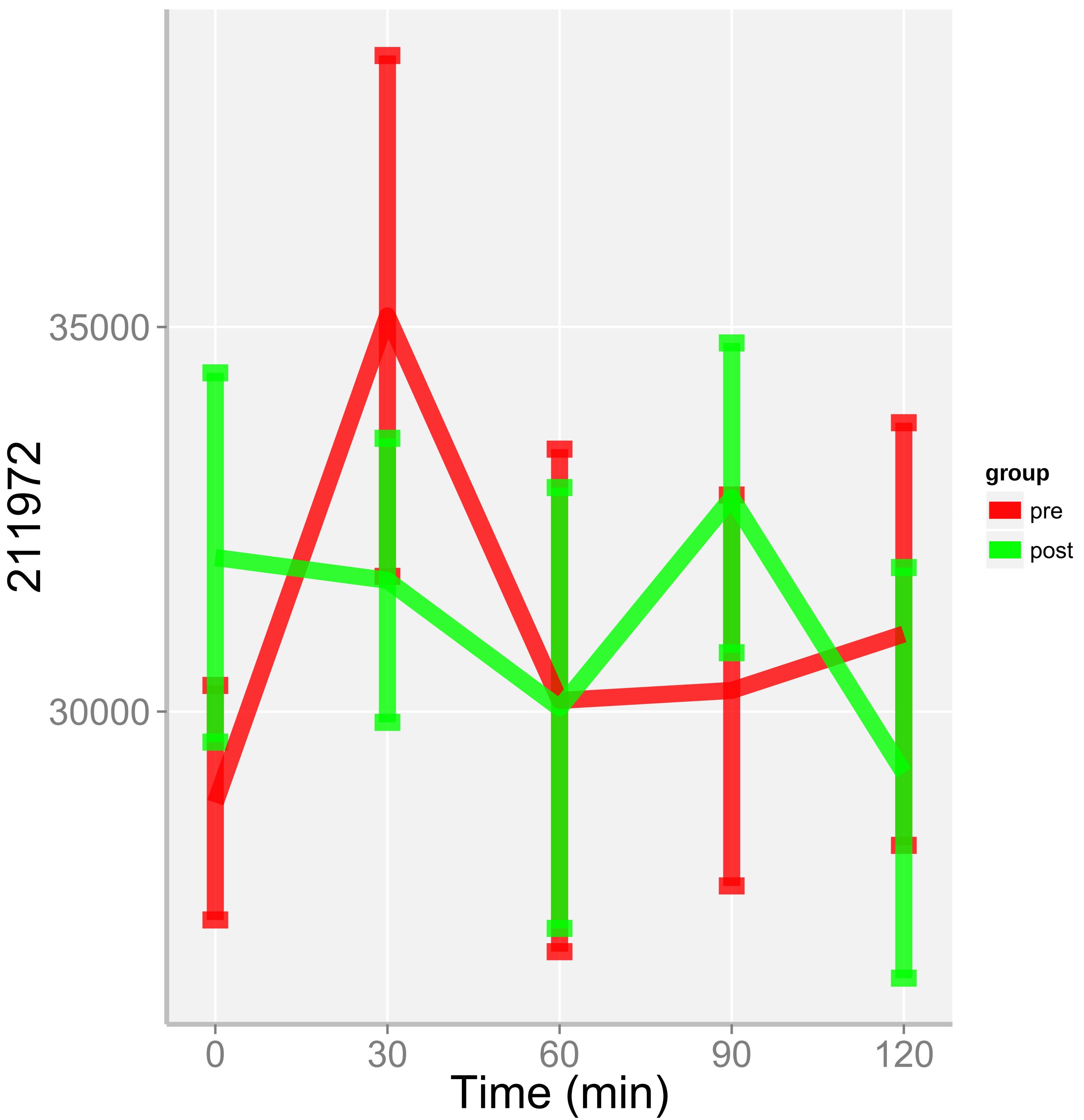

211972
35000
30000
0
30
60
90
120
Time (min)
group
pre
post

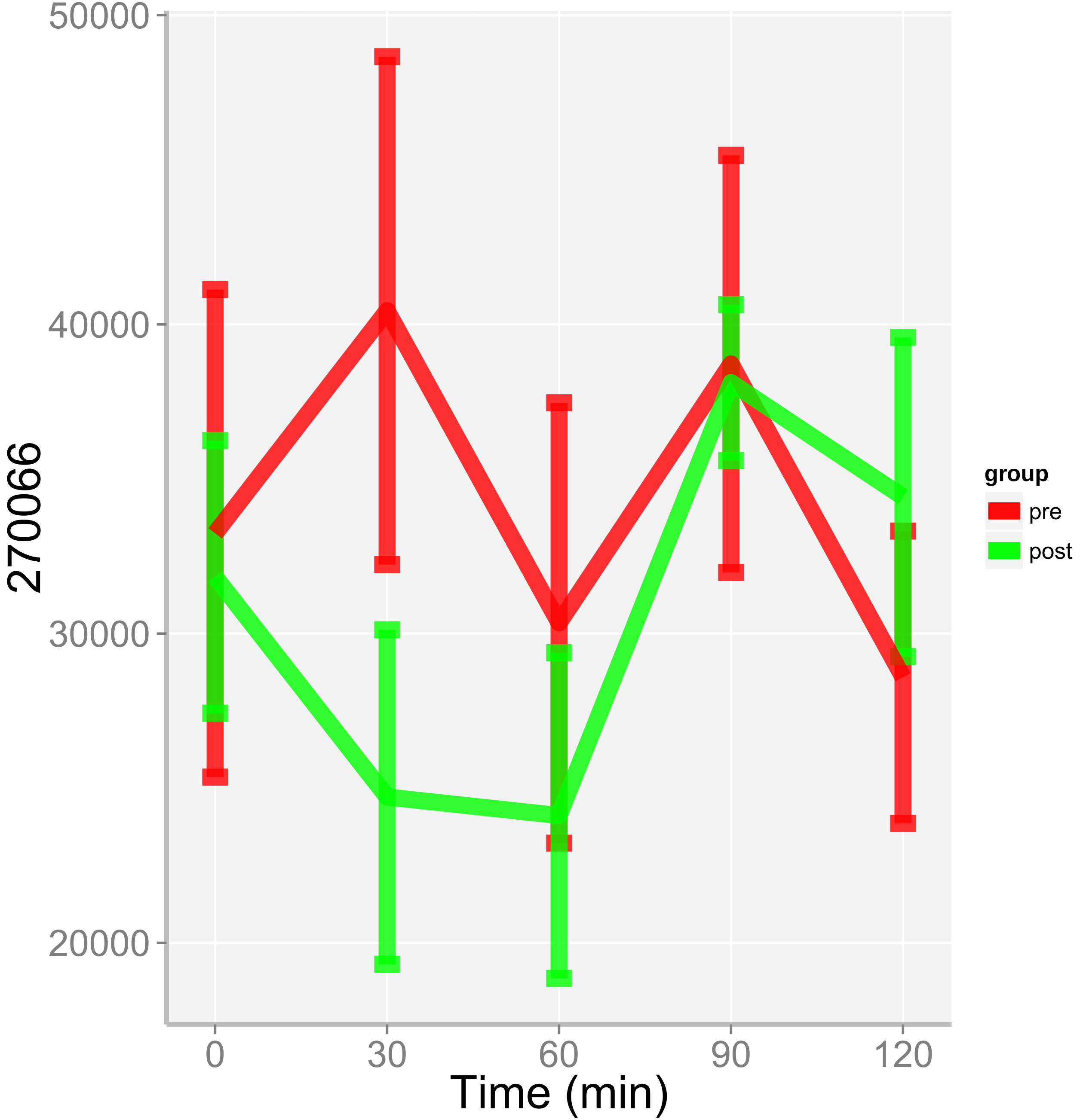

50000
40000
30000
20000
270066
0
30
60
90
120
Time (min)
group
pre
post

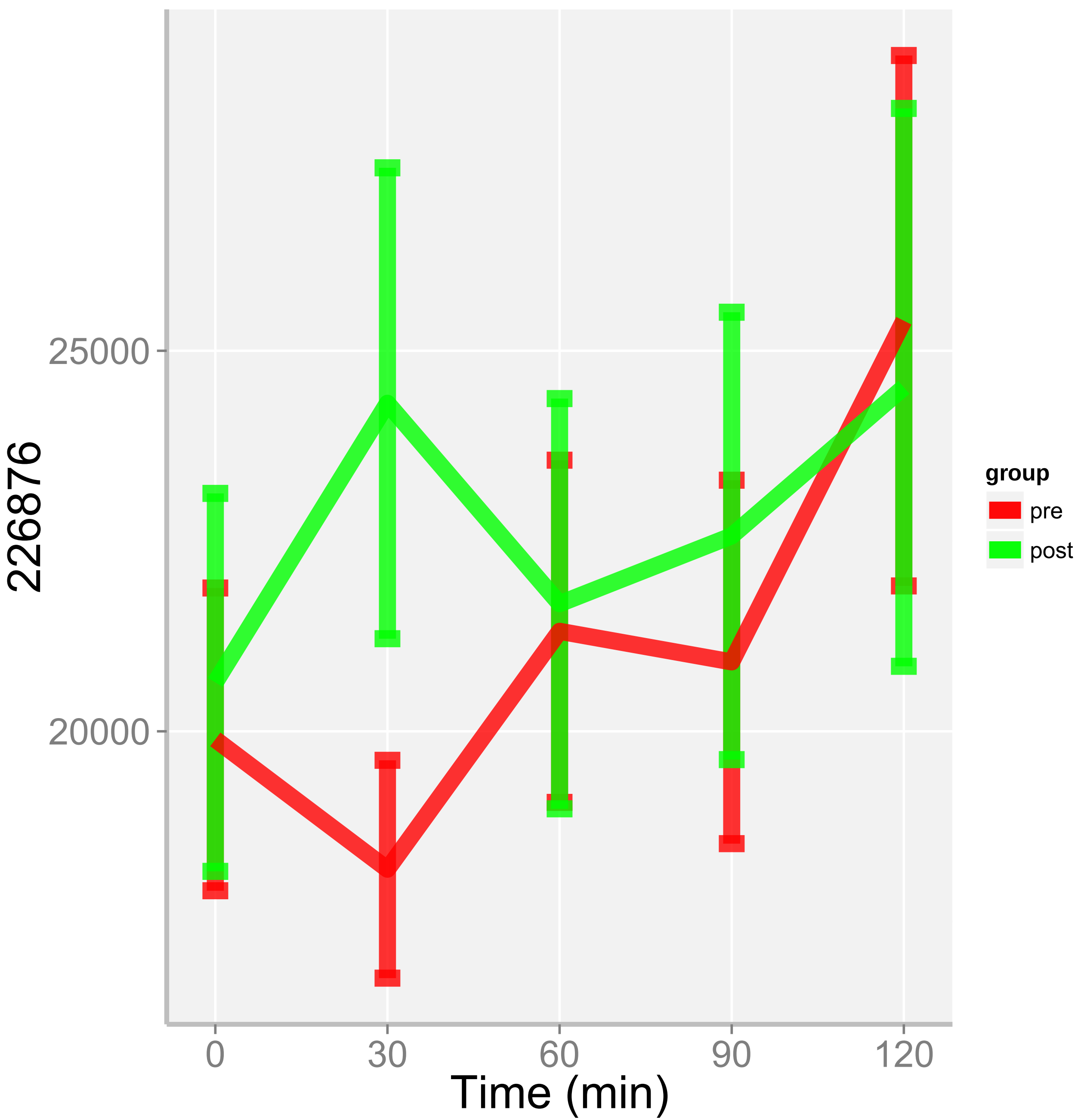

226876
25000
20000
0
30
60
90
120
Time (min)
group
pre
post

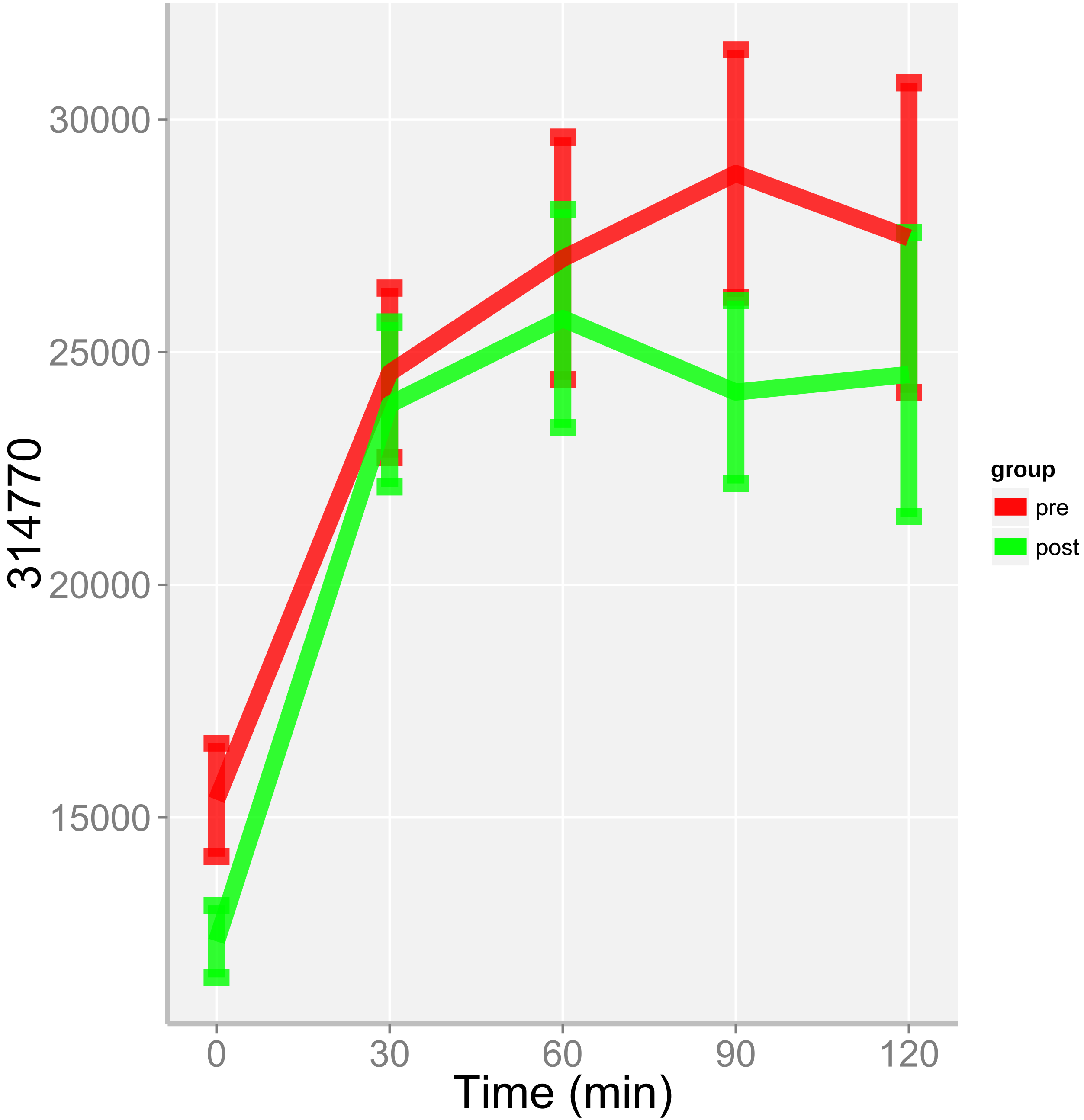

314770
30000
25000
20000
15000
0
30
60
90
120
Time (min)
group
pre
post

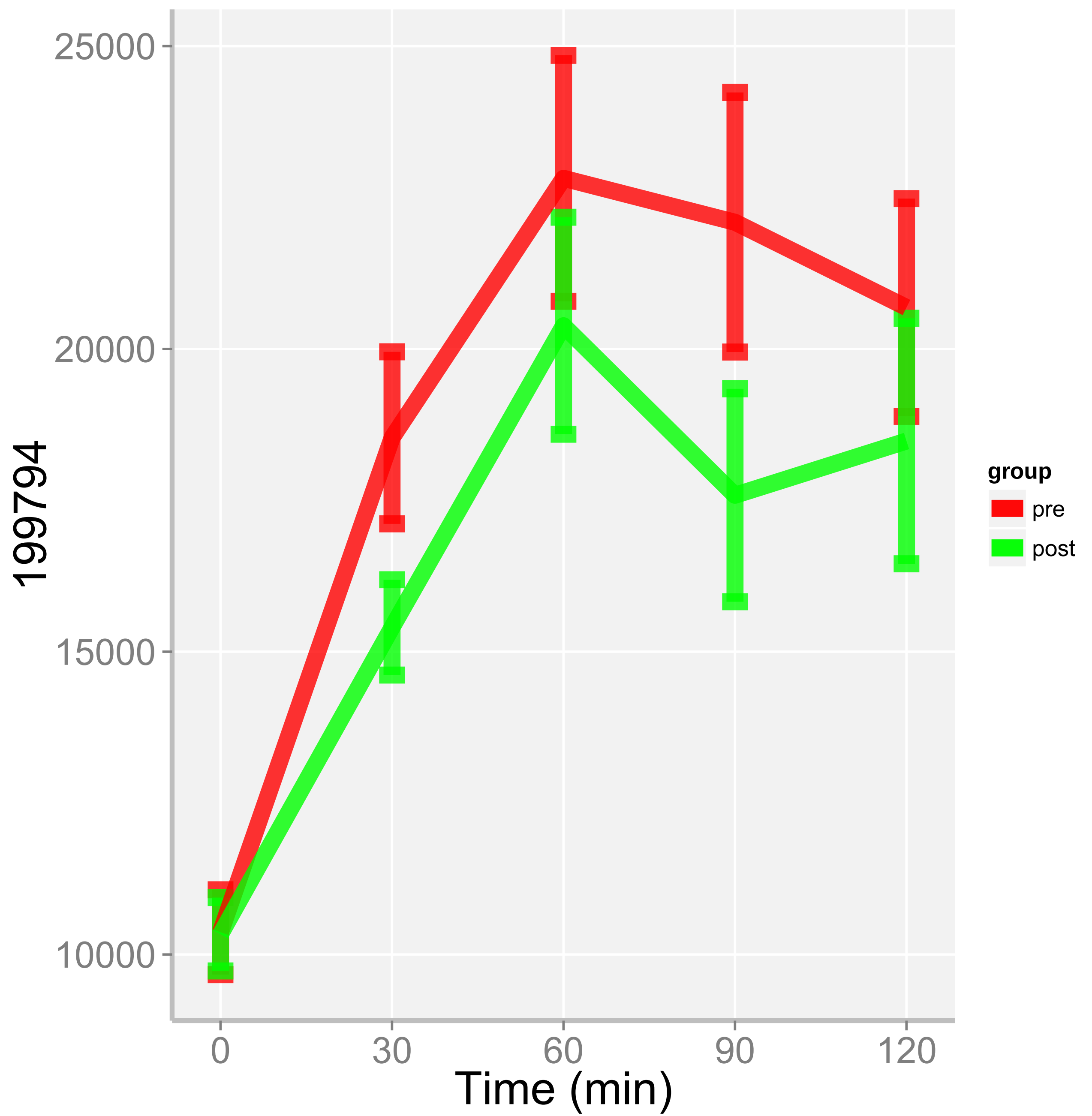

25000
20000
15000
10000
199794
0
30
60
90
120
Time (min)
group
pre
post

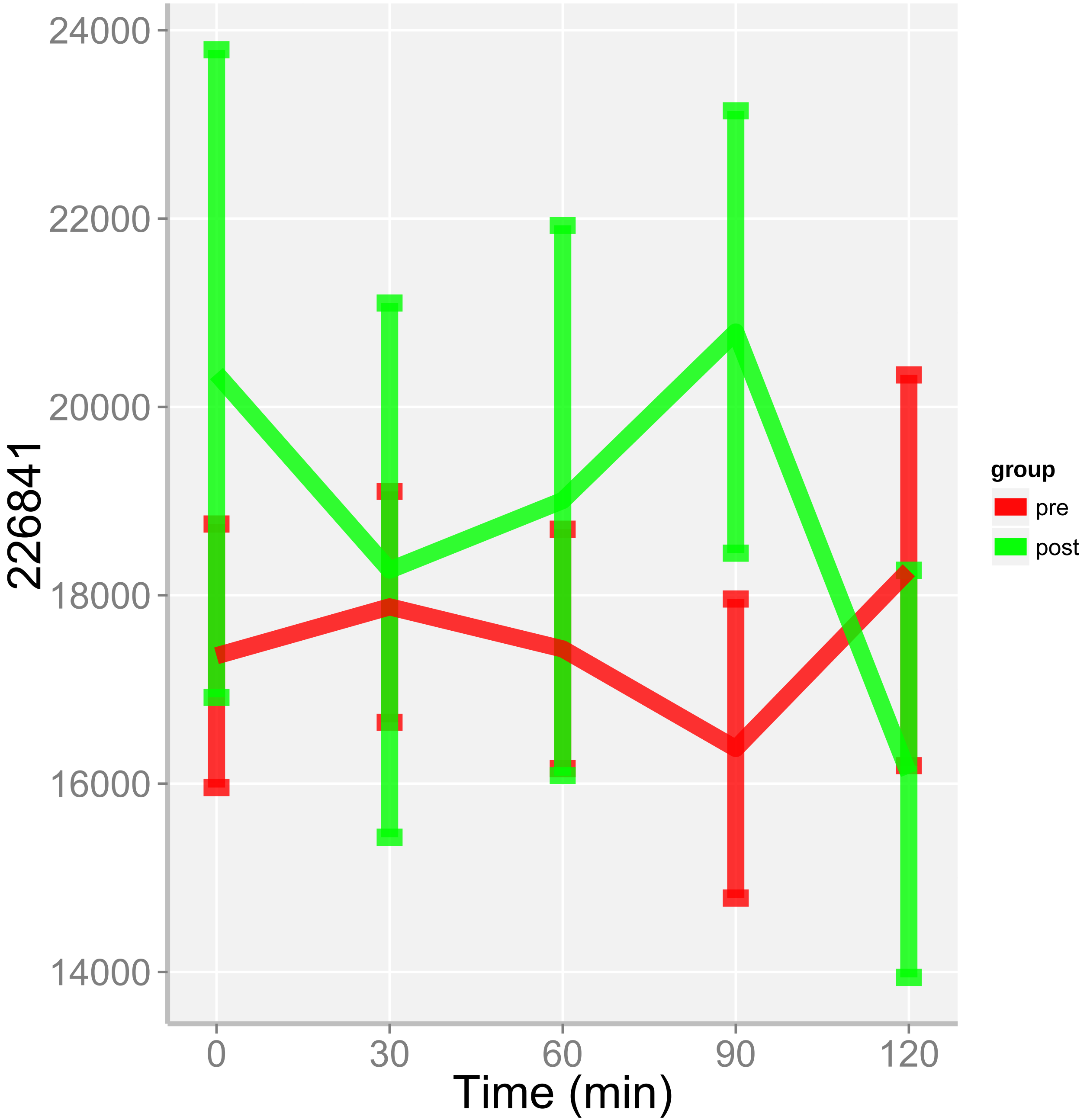

24000
22000
20000
18000
16000
14000
226841
0
30
60
90
120
Time (min)
group
pre
post

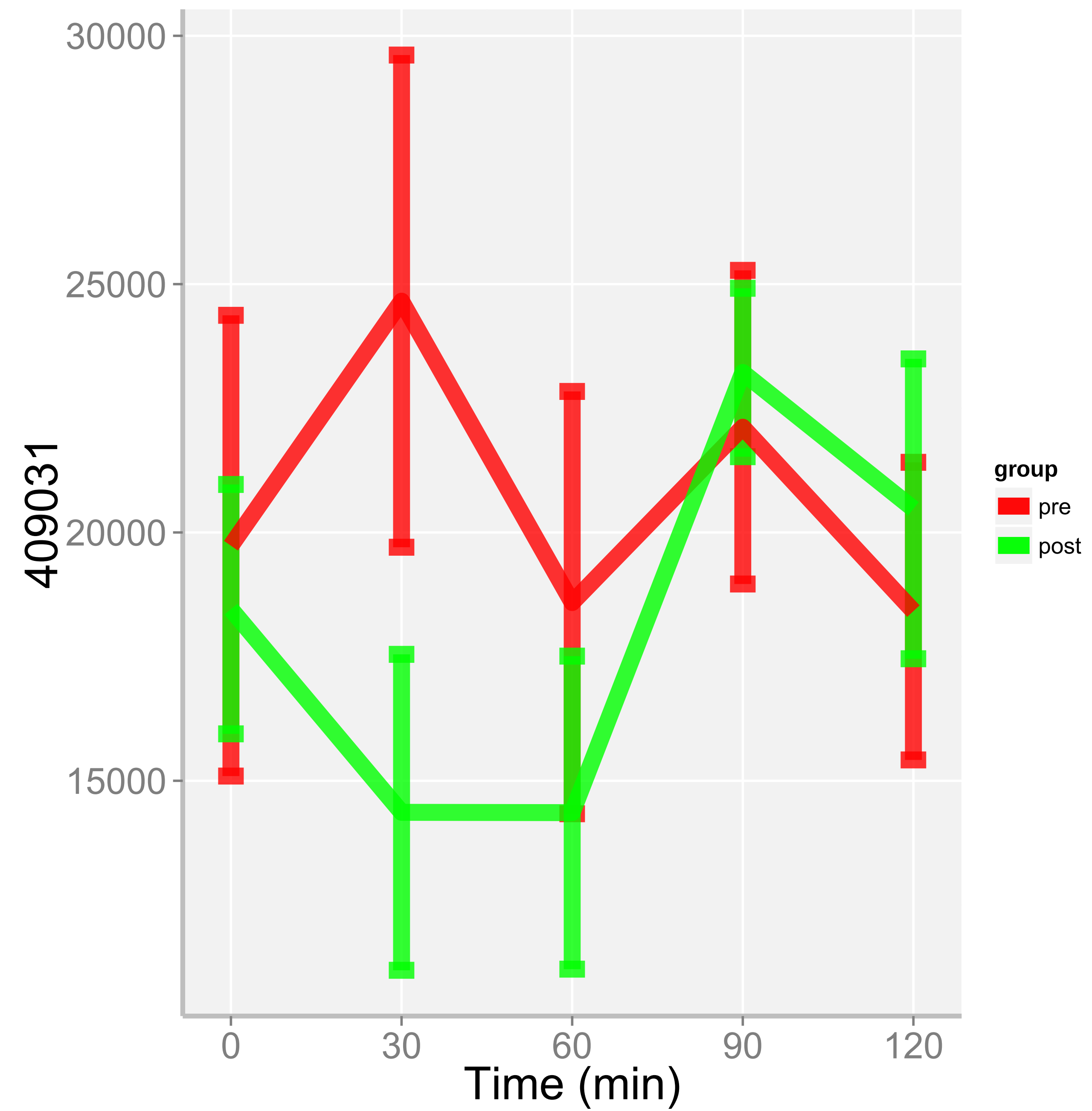
30000
25000
20000
15000
409031
0
30
60
90
120
Time (min)
group
pre
post

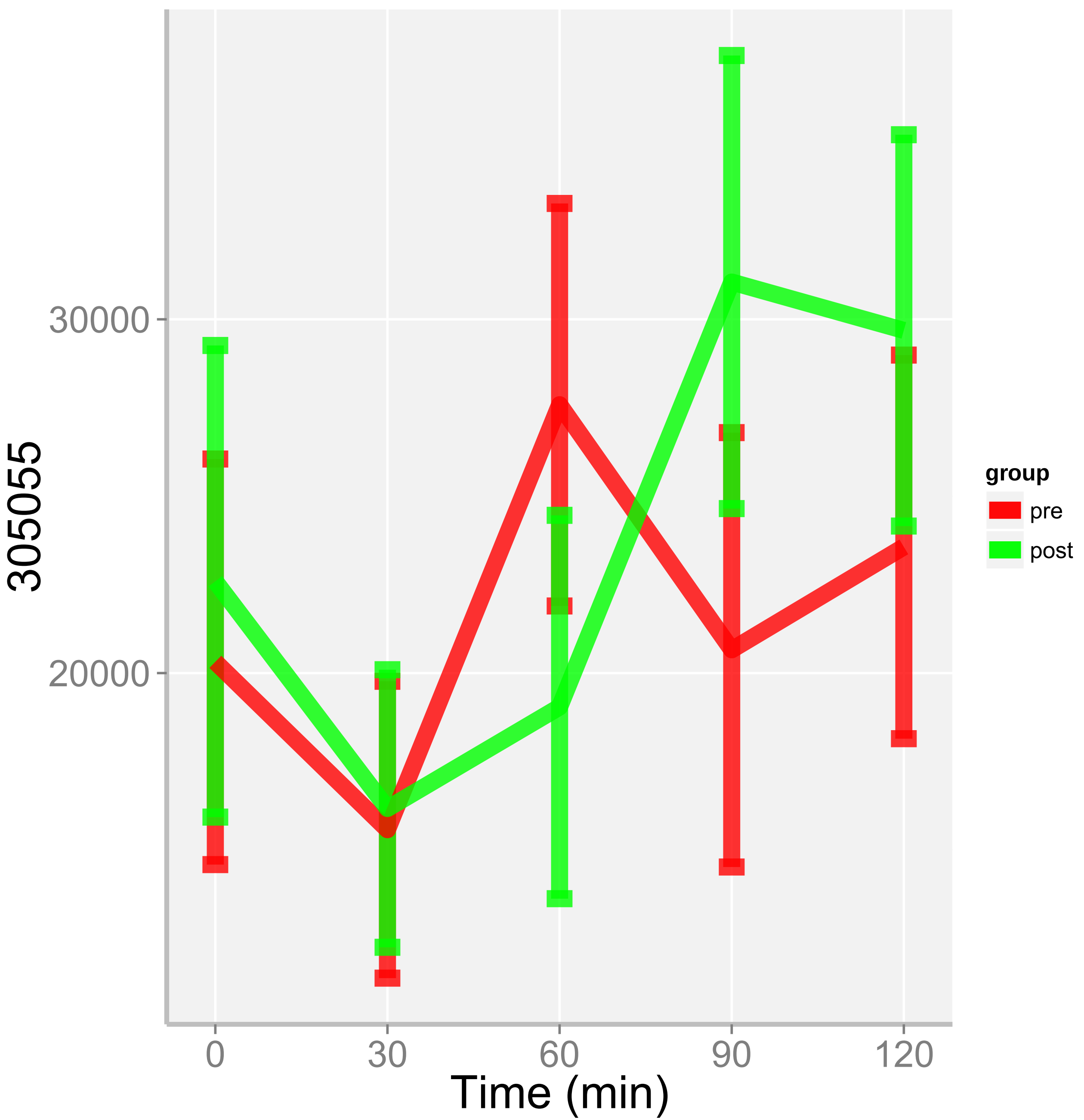

305055
30000
20000
0
30
60
90
120
Time (min)
group
pre
post

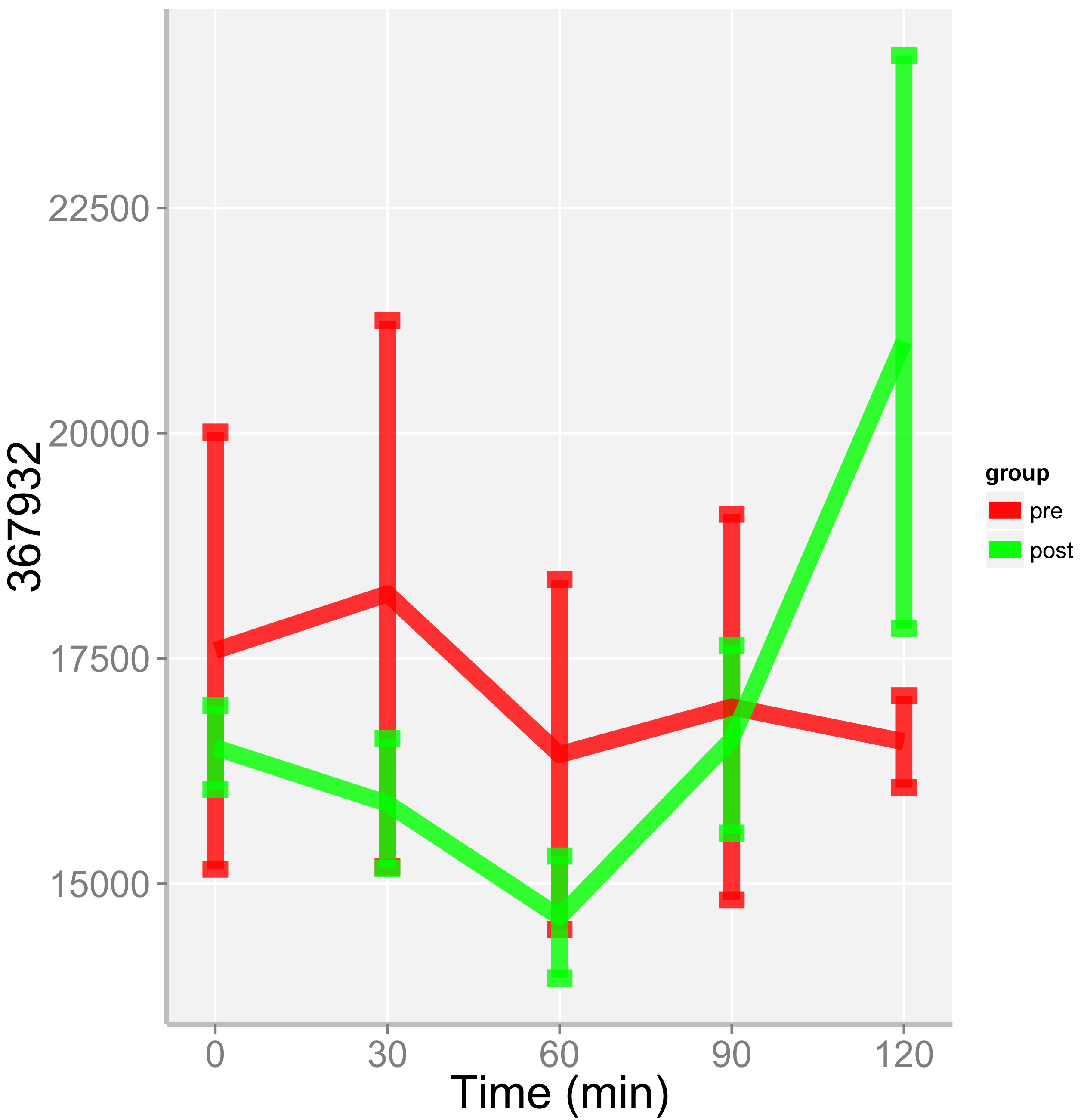

367932
22500
20000
17500
15000
0
30
60
90
120
Time (min)
group
pre
post

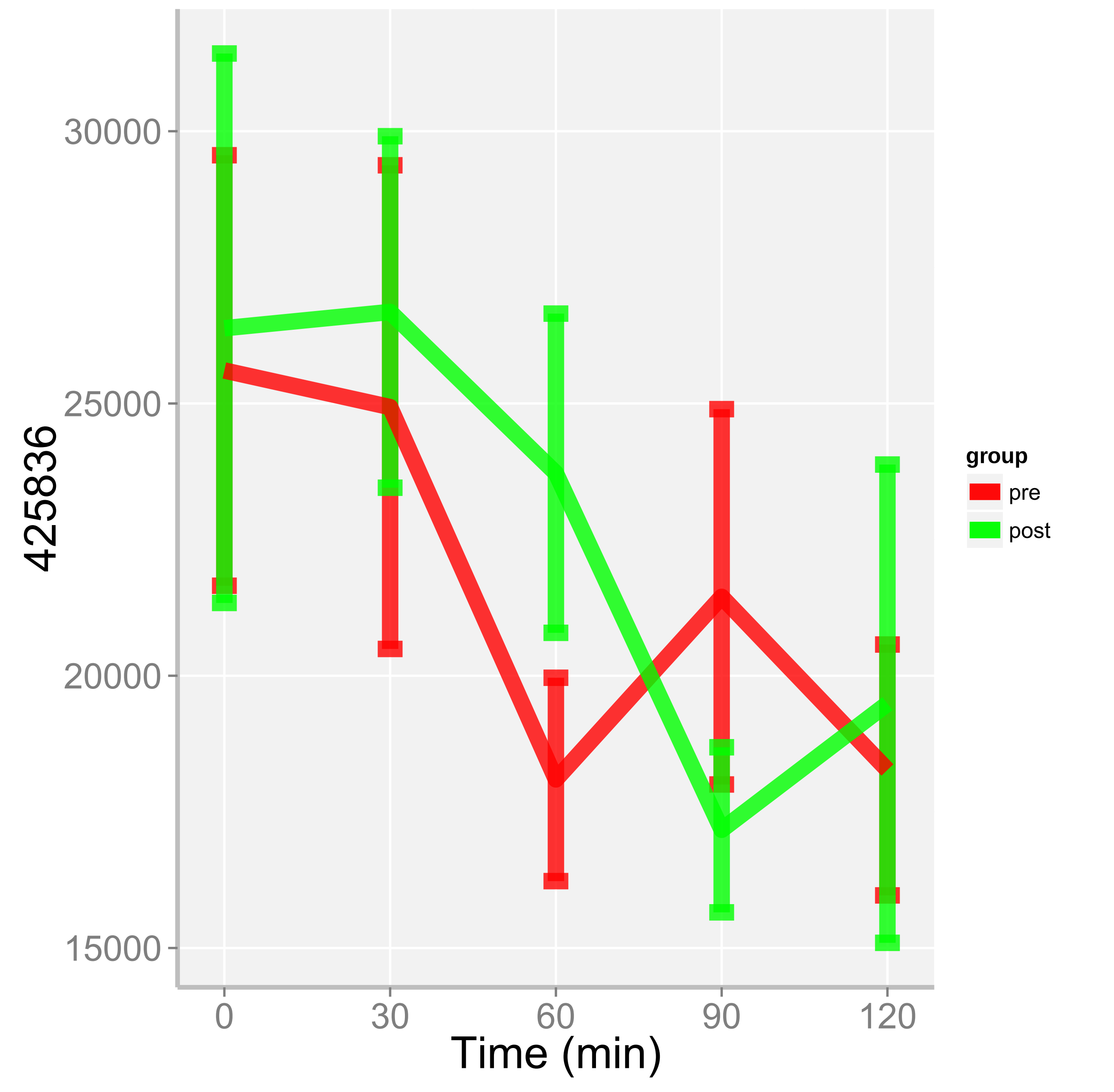

425836
30000
25000
20000
15000
0
30
60
90
120
Time (min)
group
pre
post

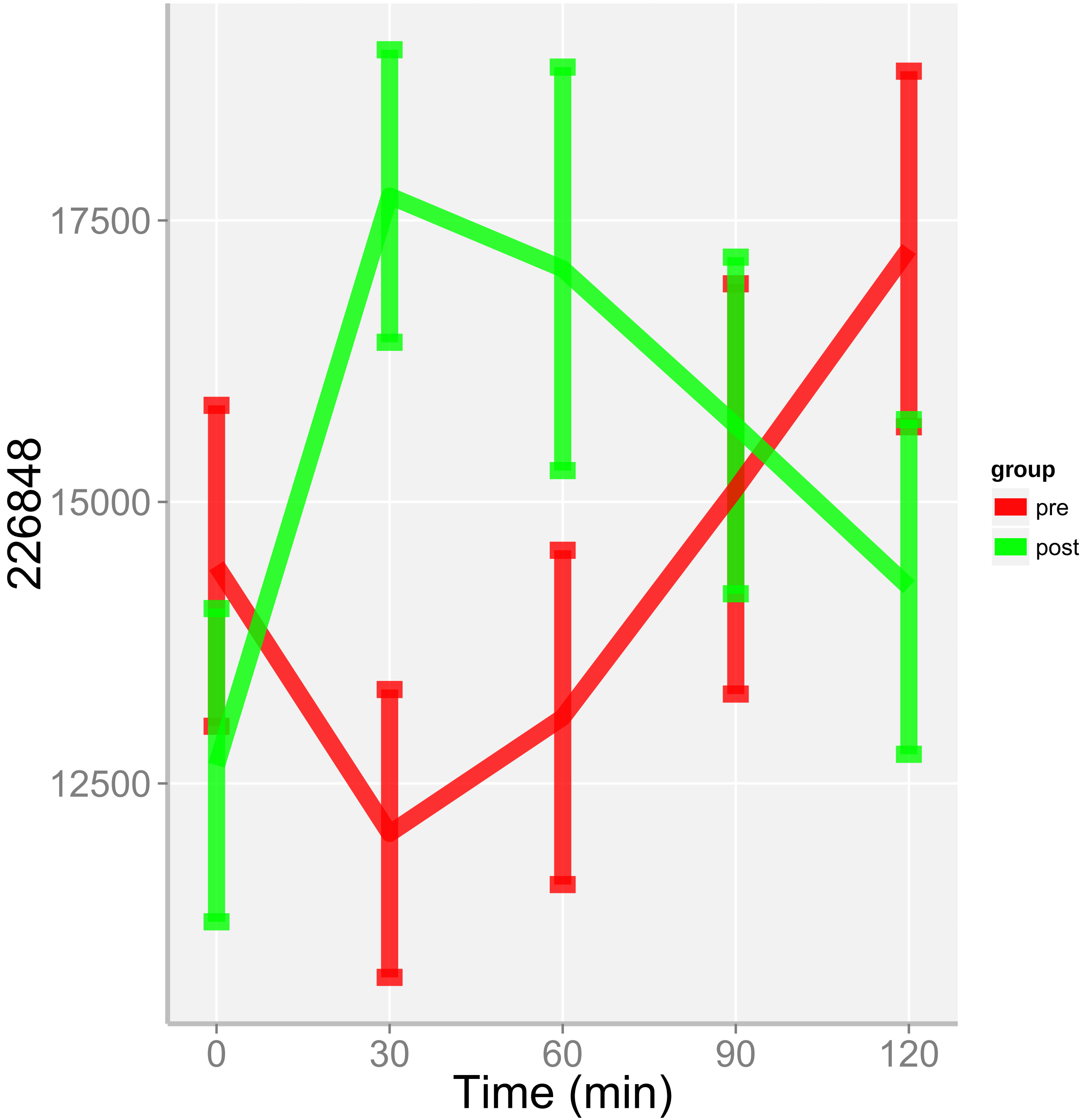

226848
17500
15000
12500
0
30
60
90
120
Time (min)
group
pre
post

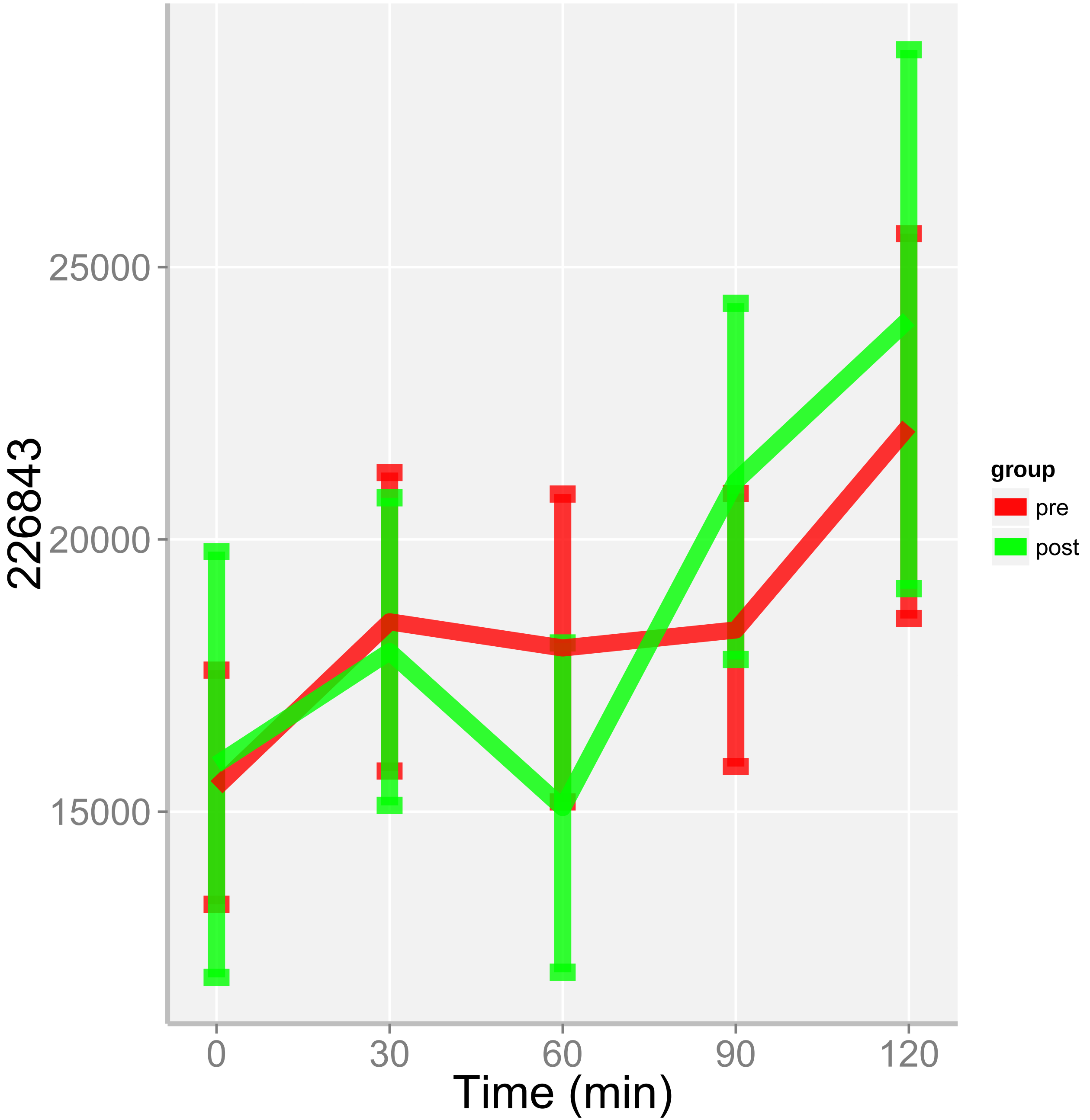

226843
25000
20000
15000
0
30
60
90
120
Time (min)
group
pre
post

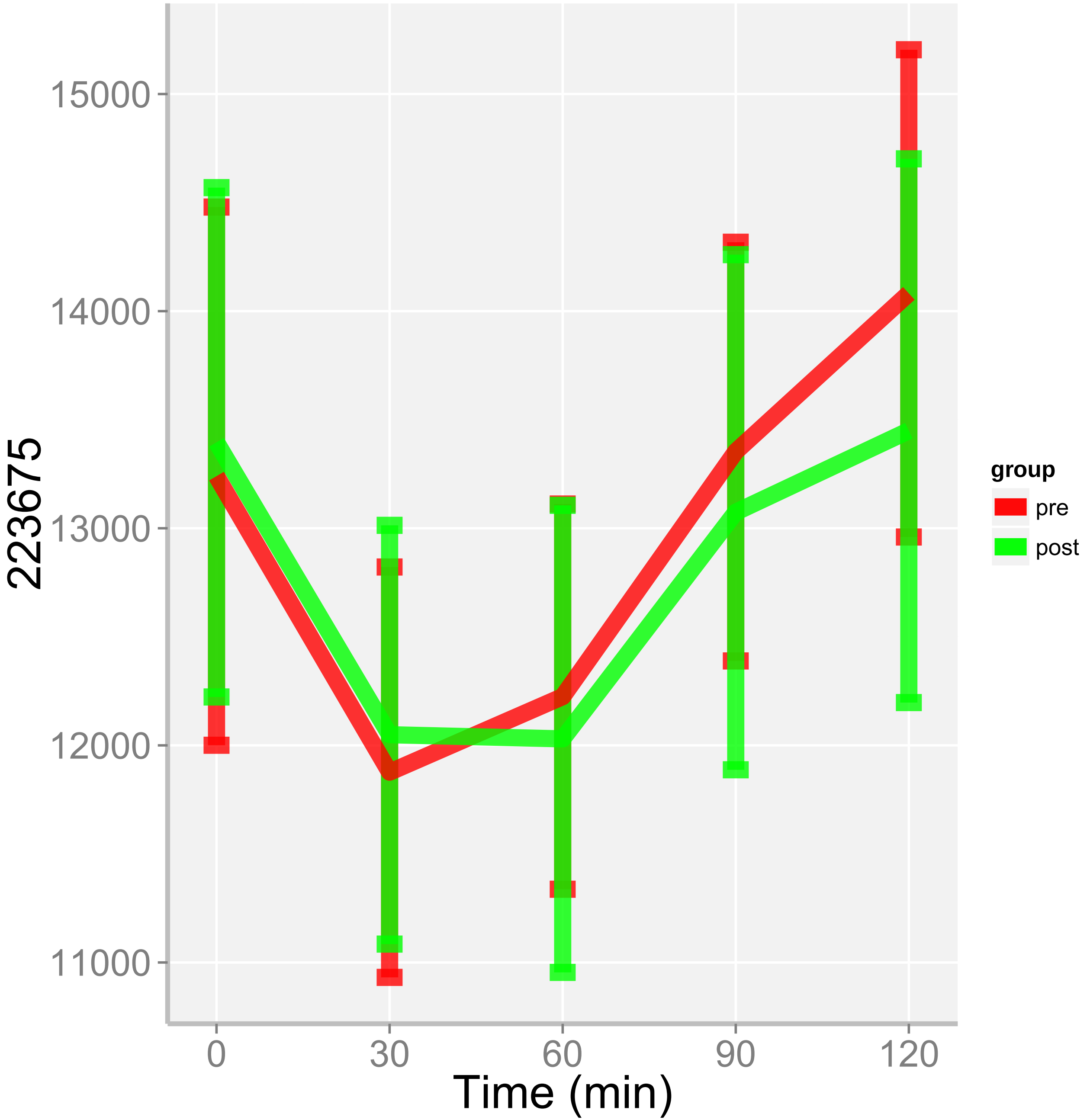

223675
15000
14000
13000
12000
11000
0
30
60
90
120
Time (min)
group
pre
post

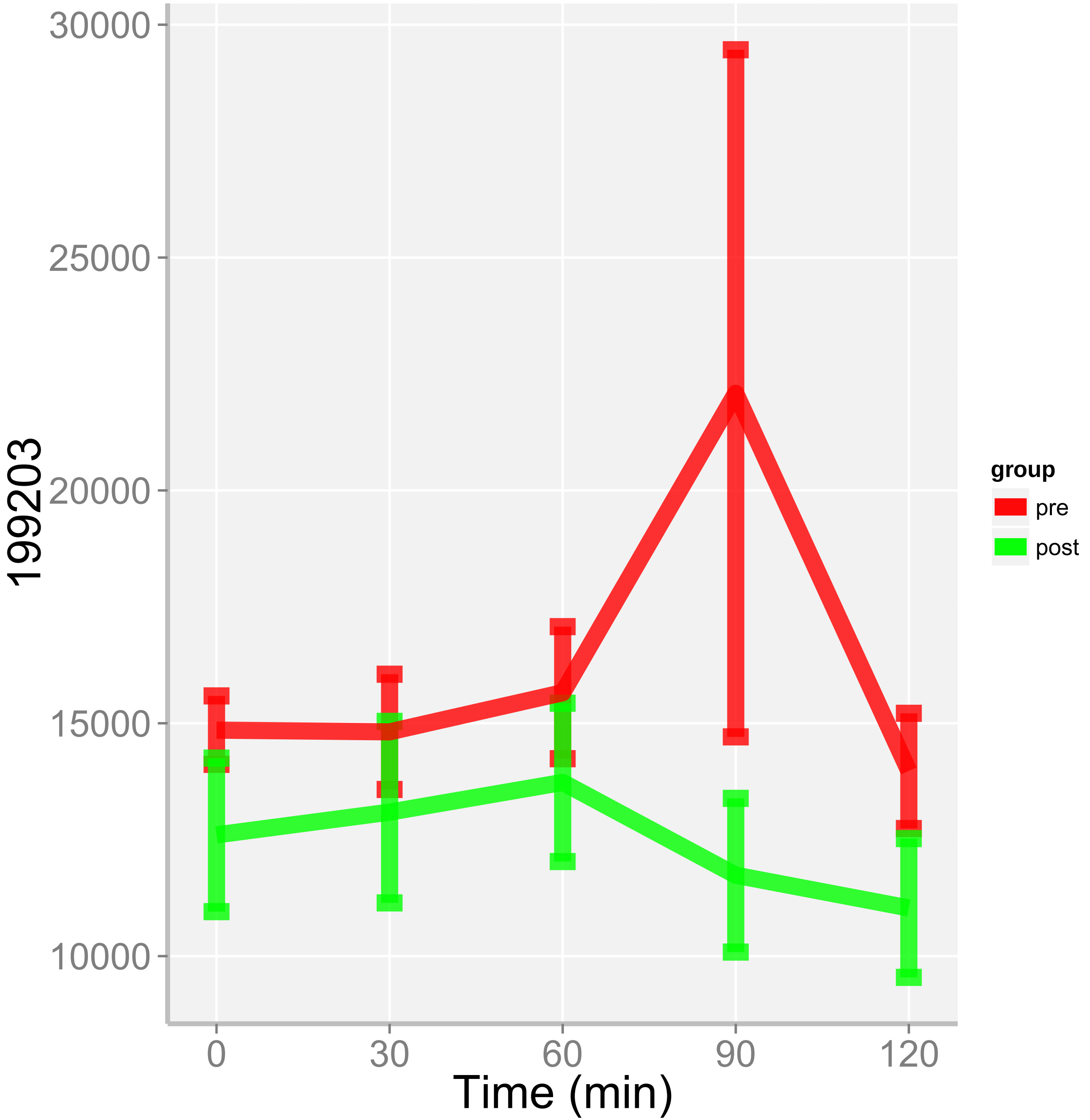

30000
25000
20000
15000
10000
1992O3
0
30
60
90
120
Time (min)
group
pre
post

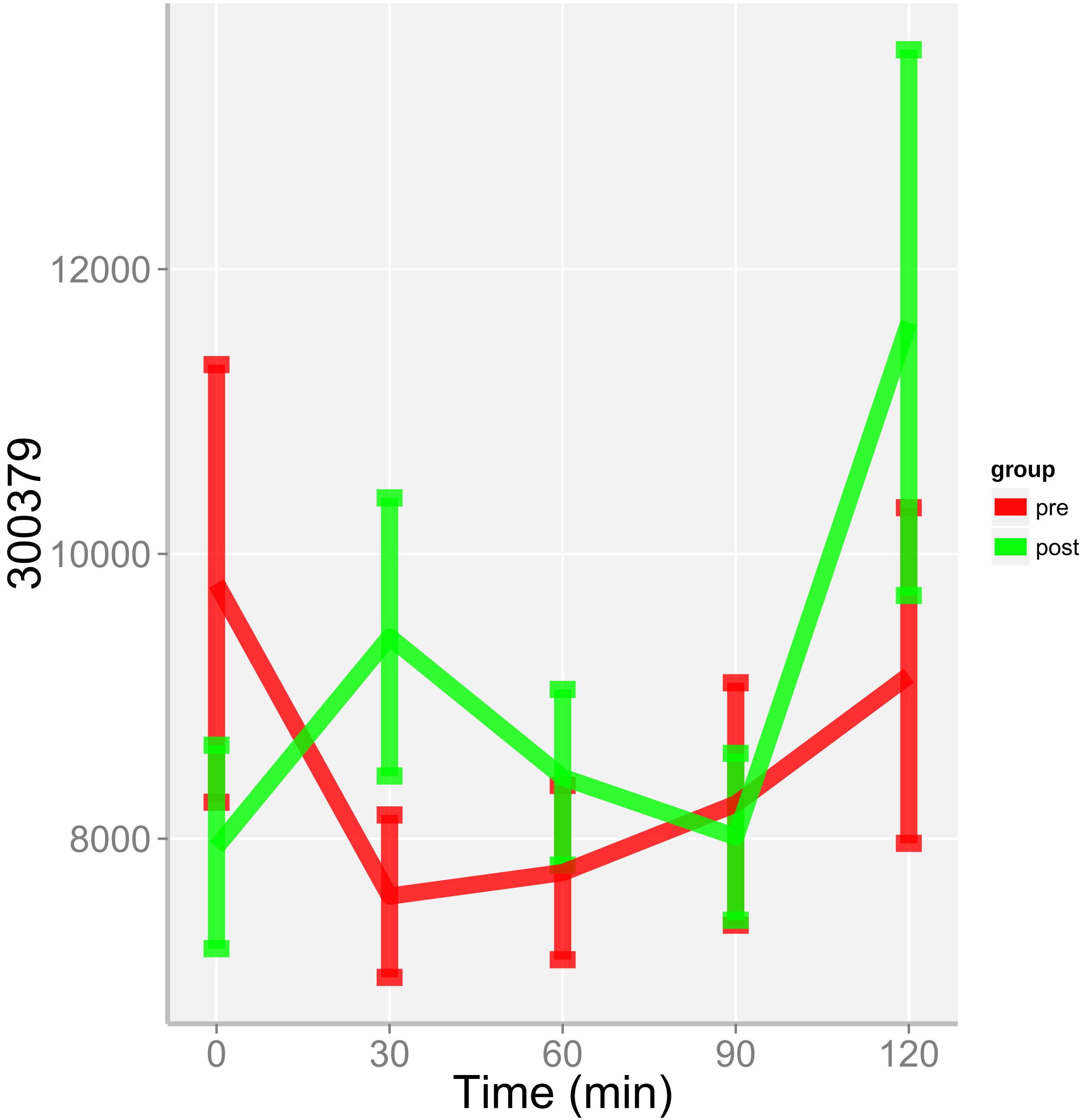

300379
12000
10000
8000
0
30
60
90
120
Time (min)
group
pre
post

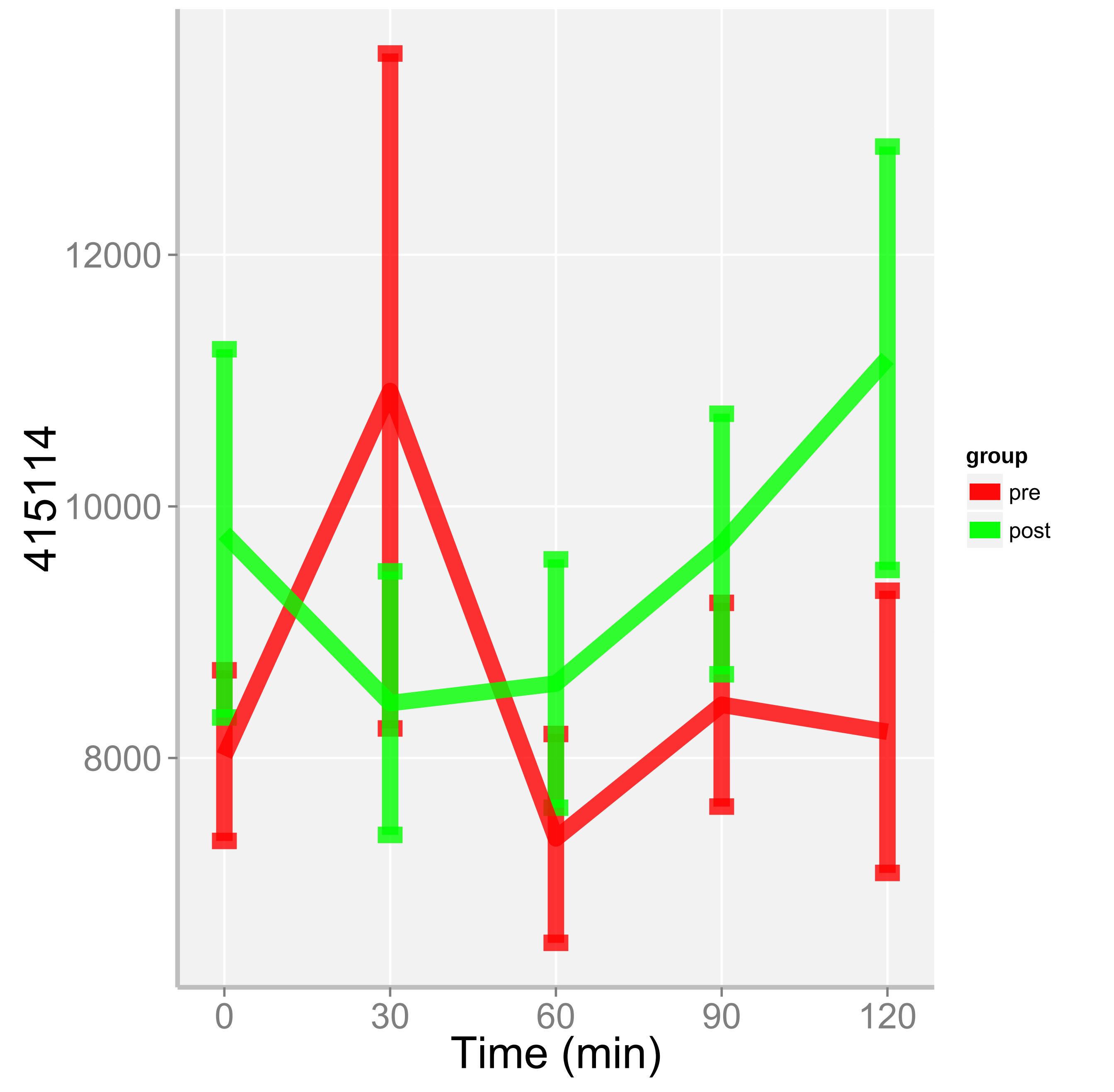

415114
12000
10000
8000
0
30
60
90
120
Time (min)
group
pre
post

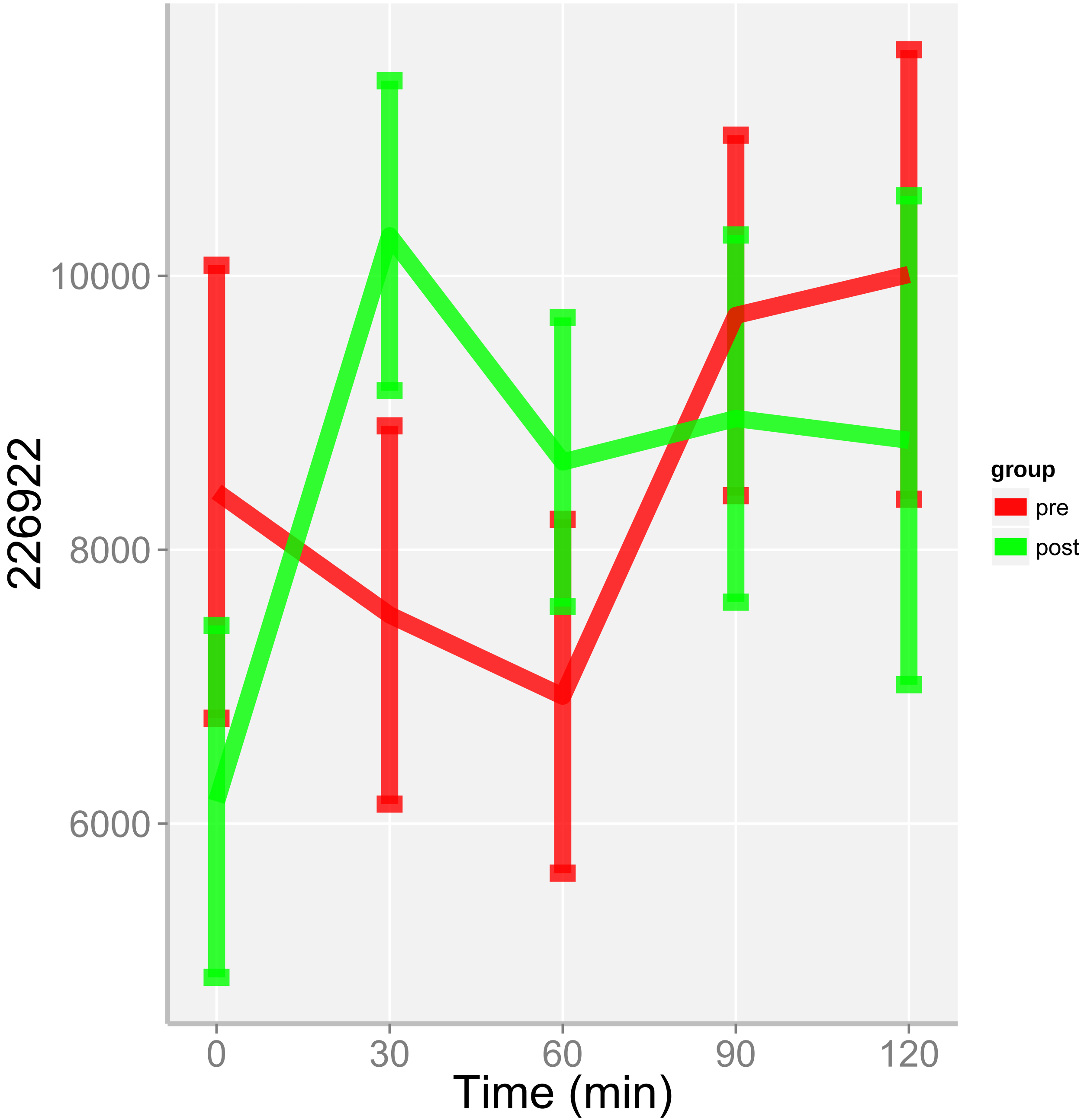

226922
10000
8000
6000
0
30
60
90
120
Time (min)
group
pre
post

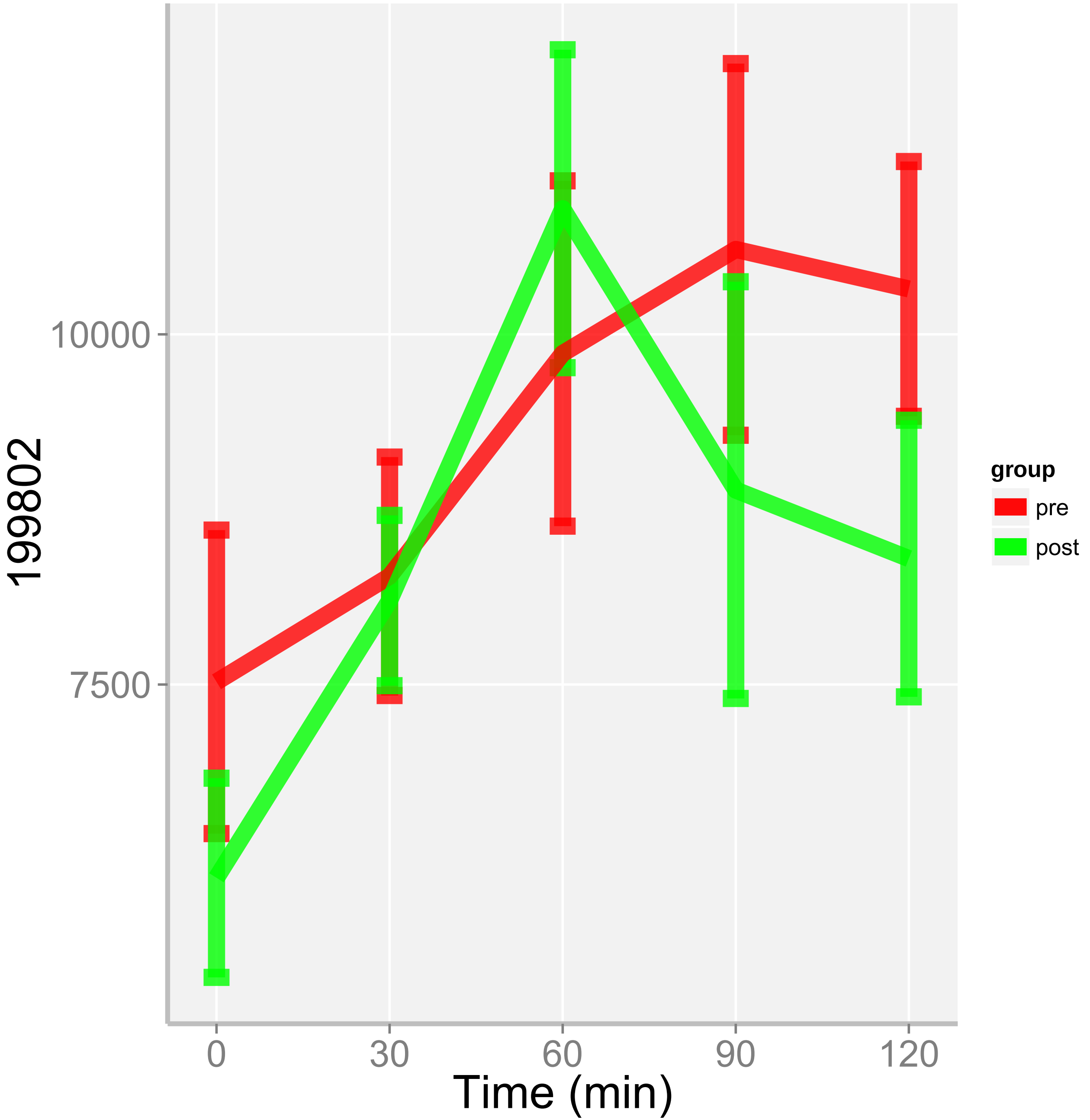

199802
10000
7500
0
30
60
90
120
Time (min)
group
pre
post

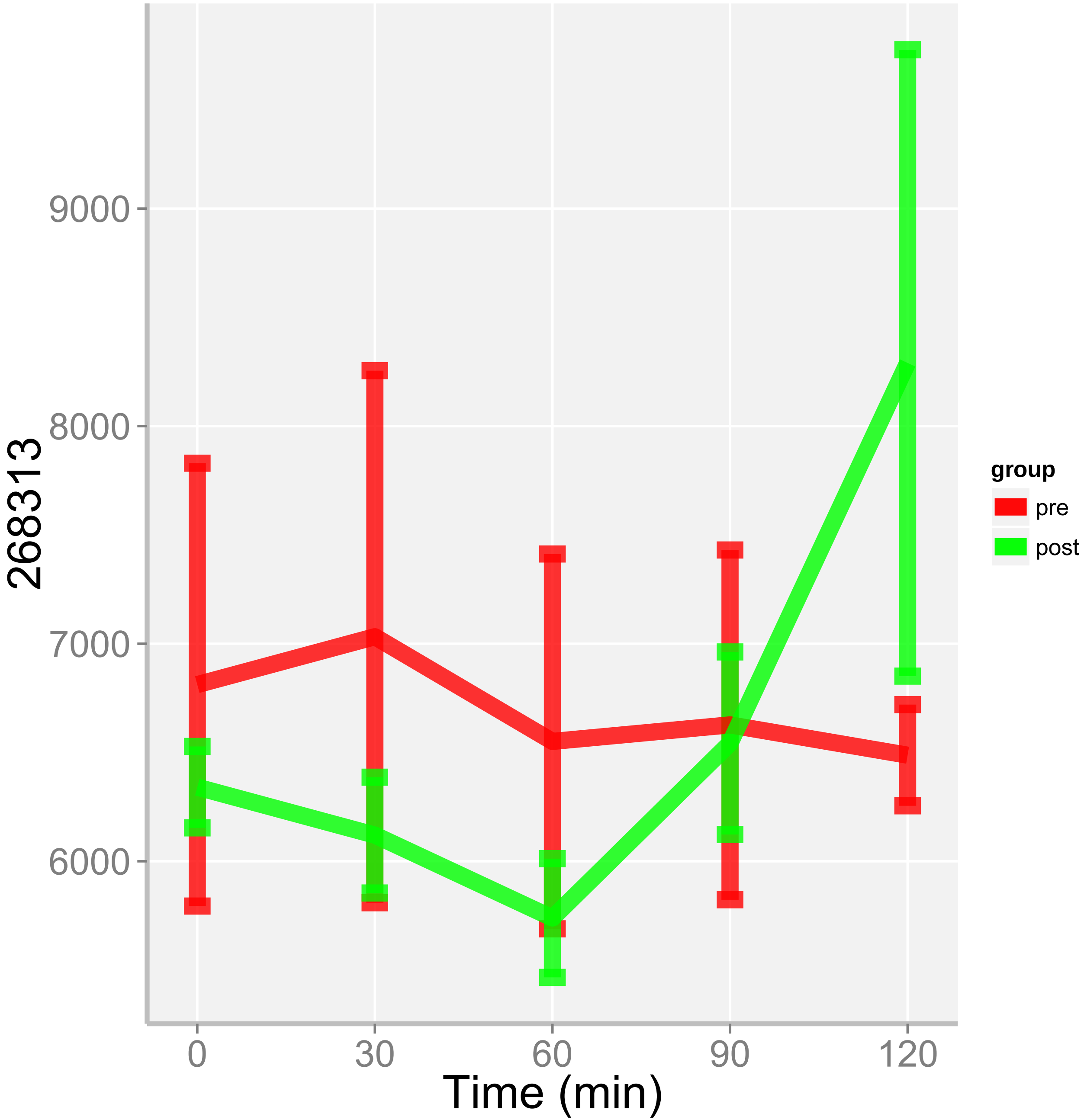

268313
9000
8000
7000
6000
0
30
60
90
120
Time (min)
group
pre
post

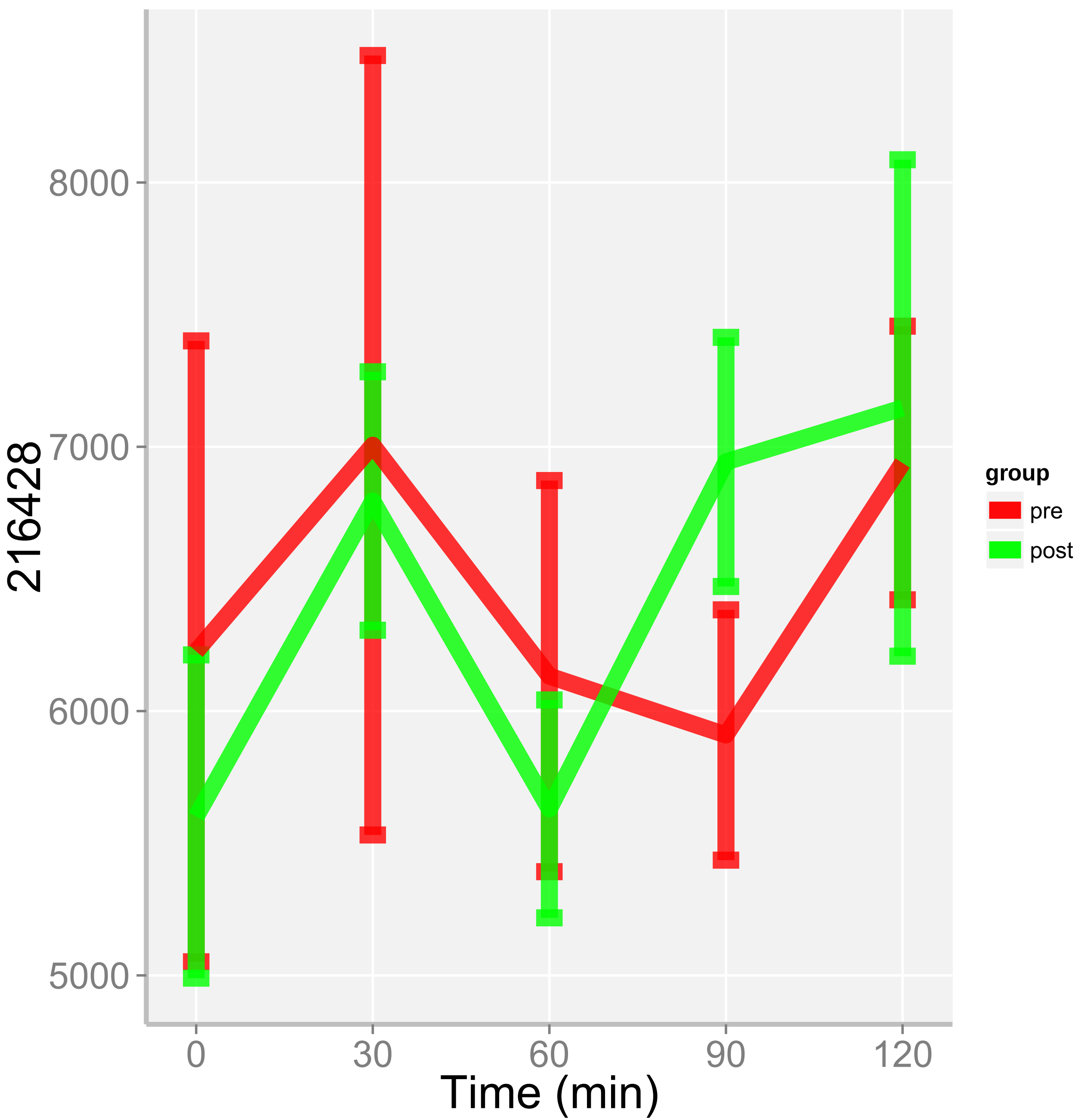
216428
8000
7000
6000
5000
0
30
60
90
120
Time (min)
group
pre
post

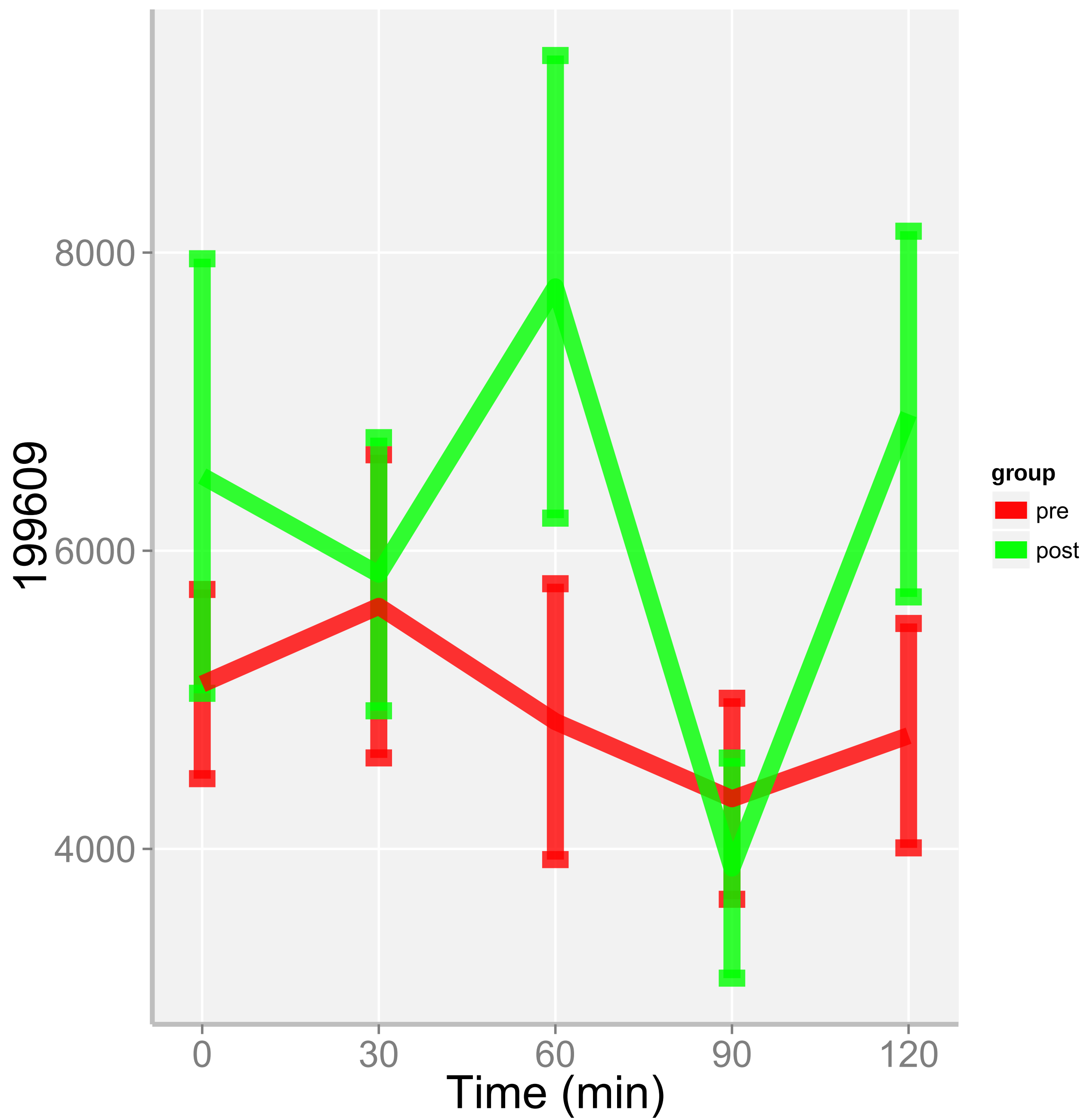

199609
8000
6000
4000
0
30
60
90
120
Time (min)
group
pre
post

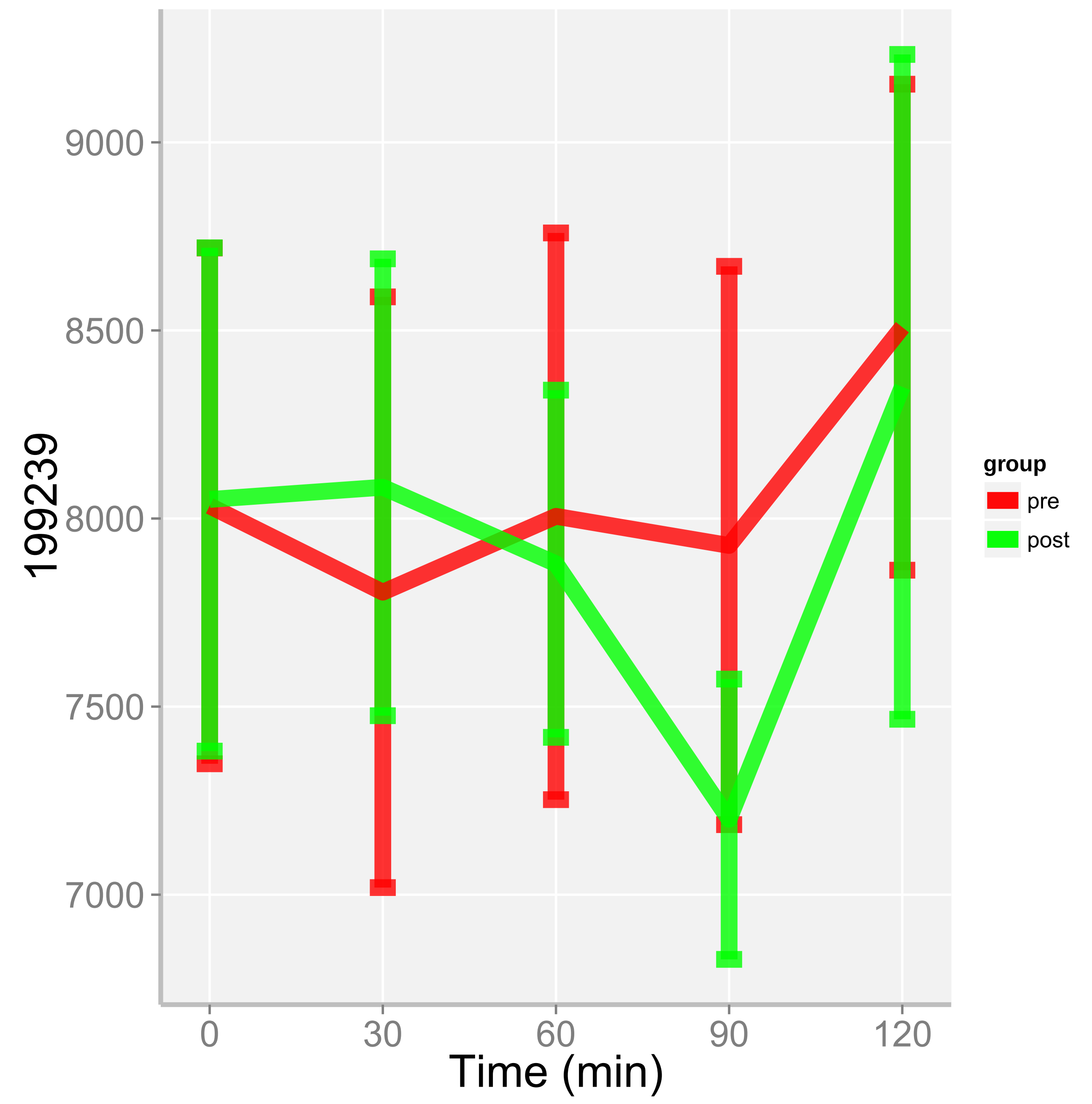

9000
8500
8000
7500
7000
199239
0
30
60
90
120
Time (min)
group
pre
post

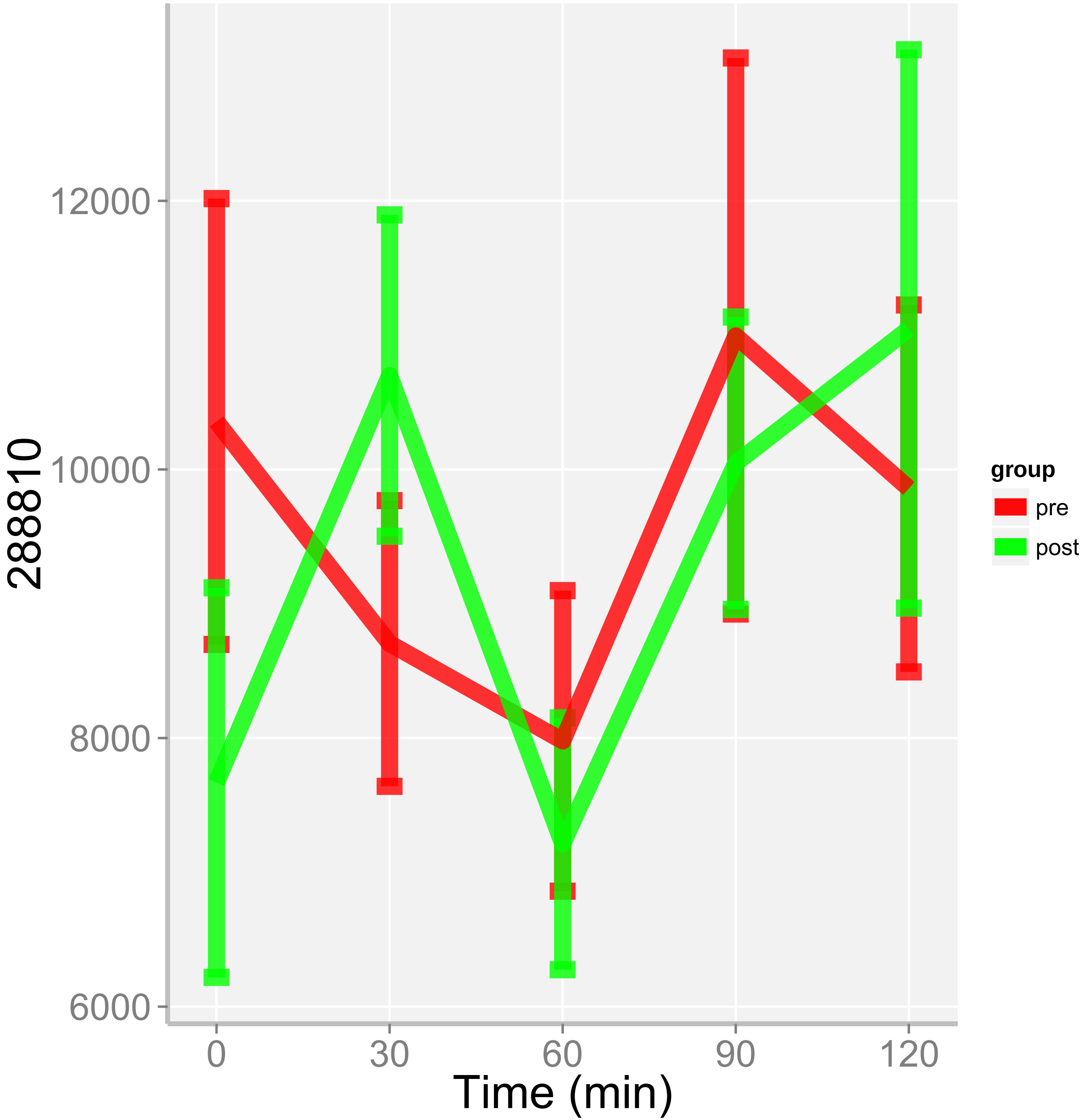

288810
12000
10000
8000
6000
0
30
60
90
120
Time (min)
group
pre
post

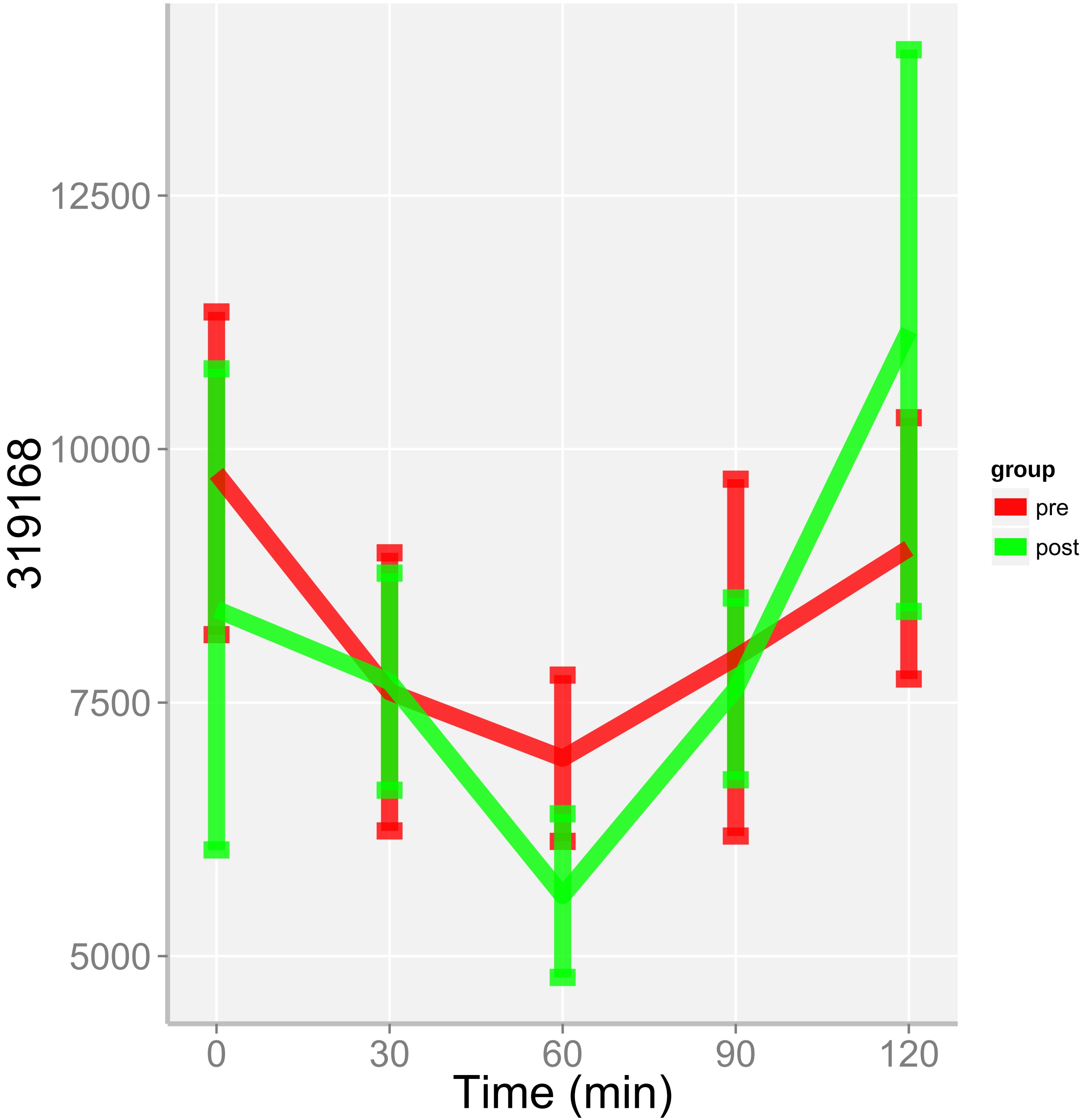

12500
10000
7500
5000
0
30
60
90
120
Time (min)
319168
group
pre
post

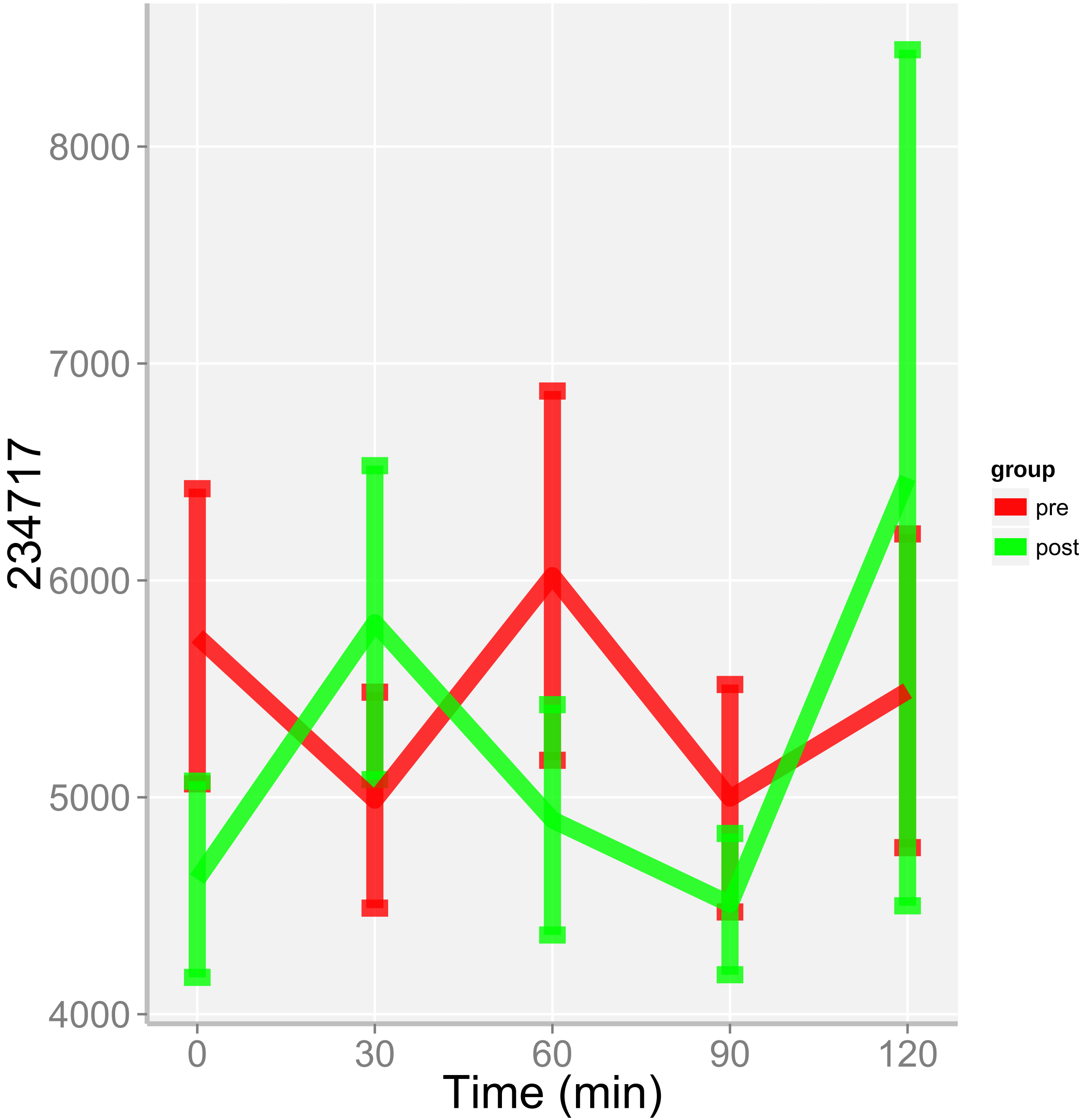

2347717
8000
7000
6000
5000
4000
0
30
60
90
120
Time (min)
group
pre
post

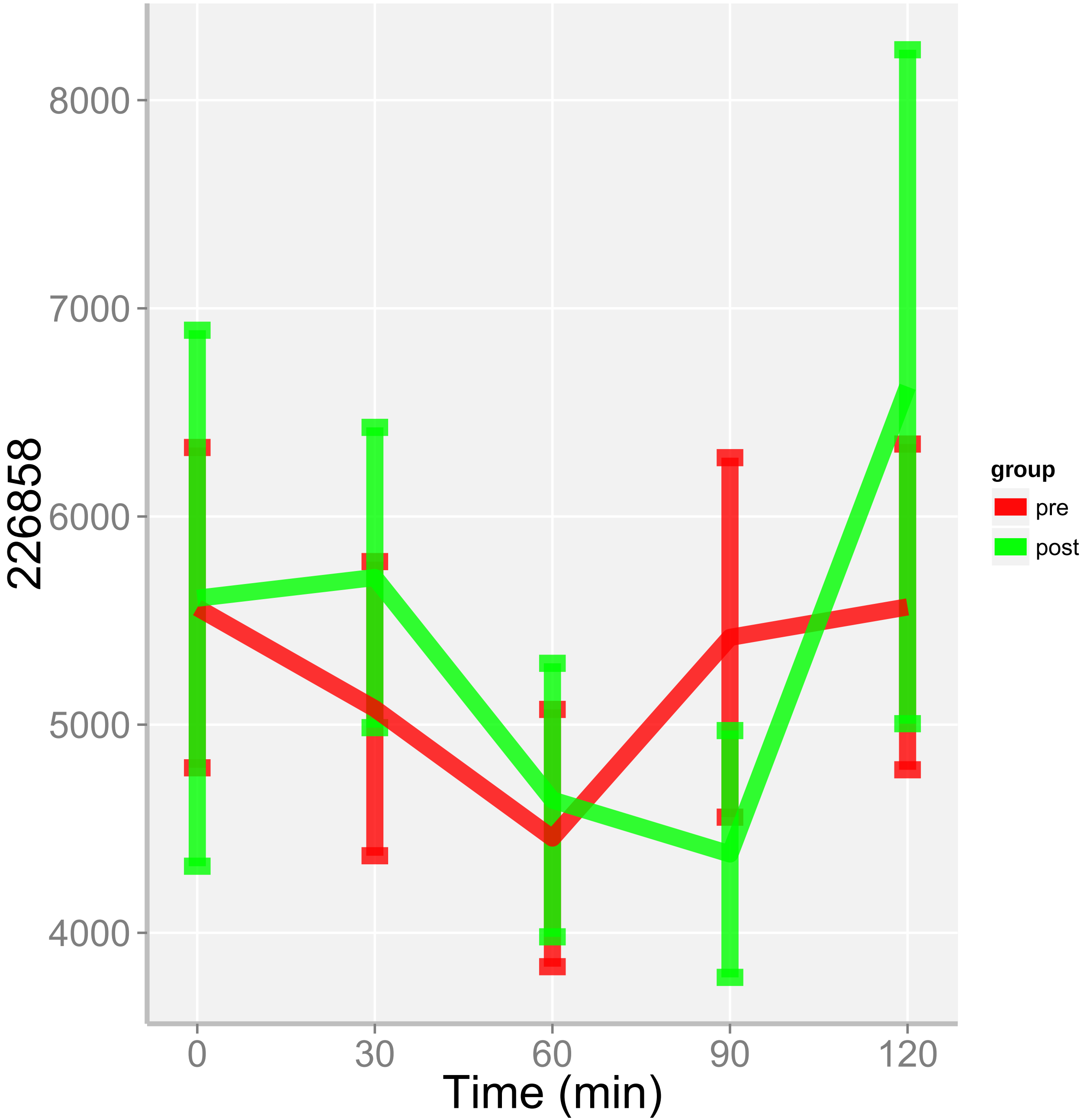

8000
7000
6000
5000
4000
226858
0
30
60
90
120
Time (min)
group
pre
post

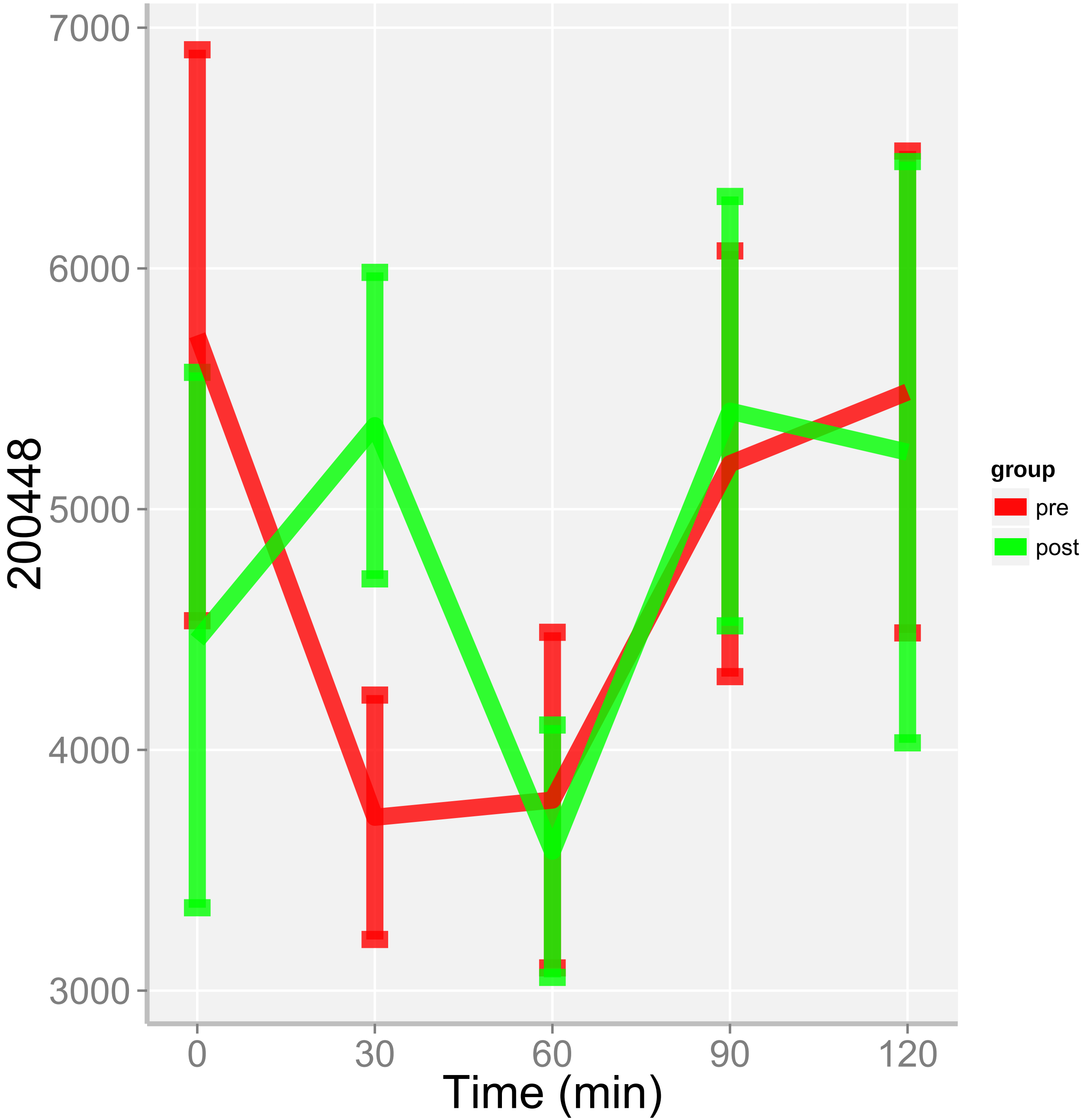

7000
6000
5000
4000
3000
200448
0
30
60
90
120
Time (min)
group
pre
post

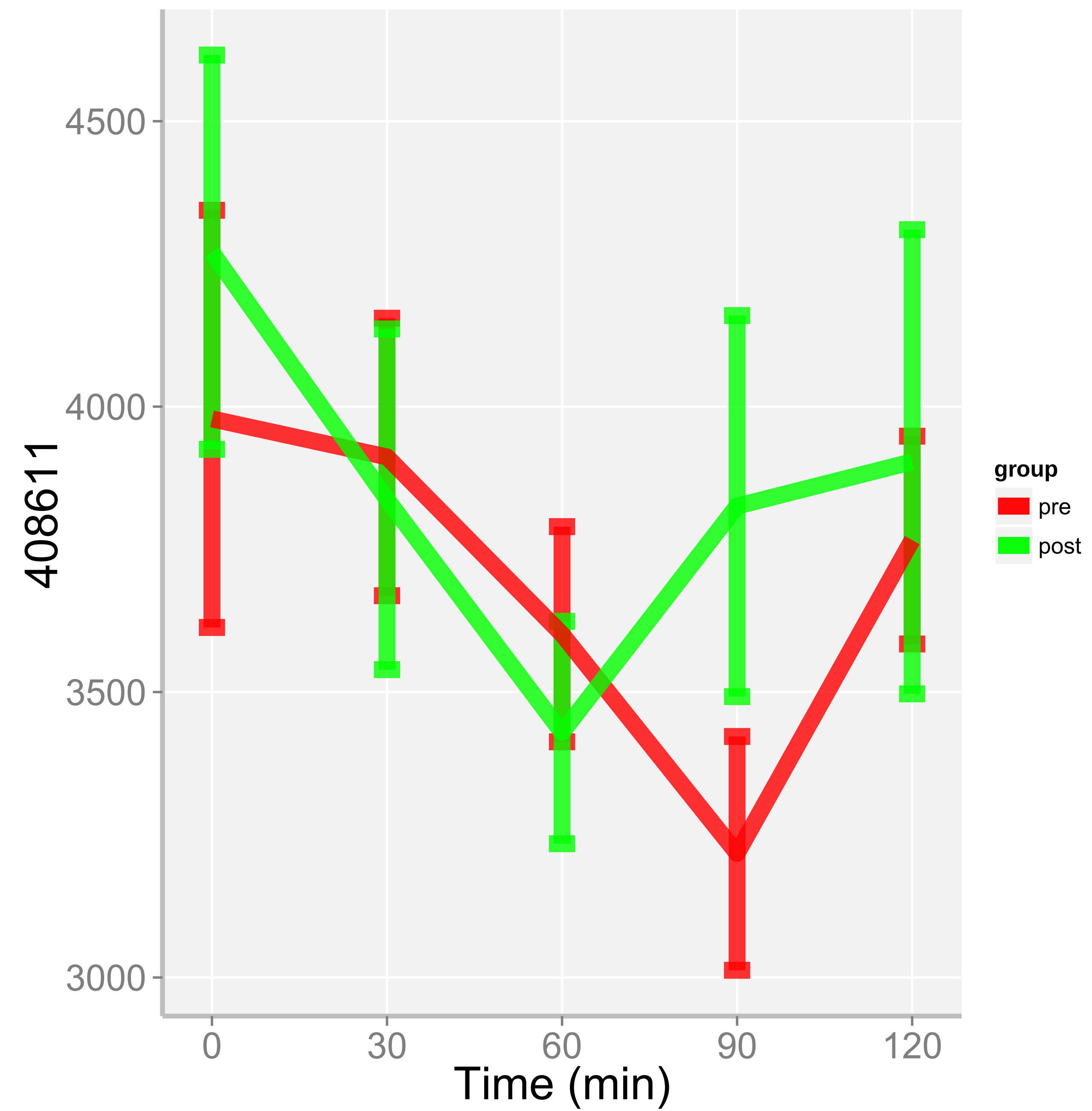
4500
4000
3500
3000
408611
0
30
60
90
120
Time (min)
group
pre
post

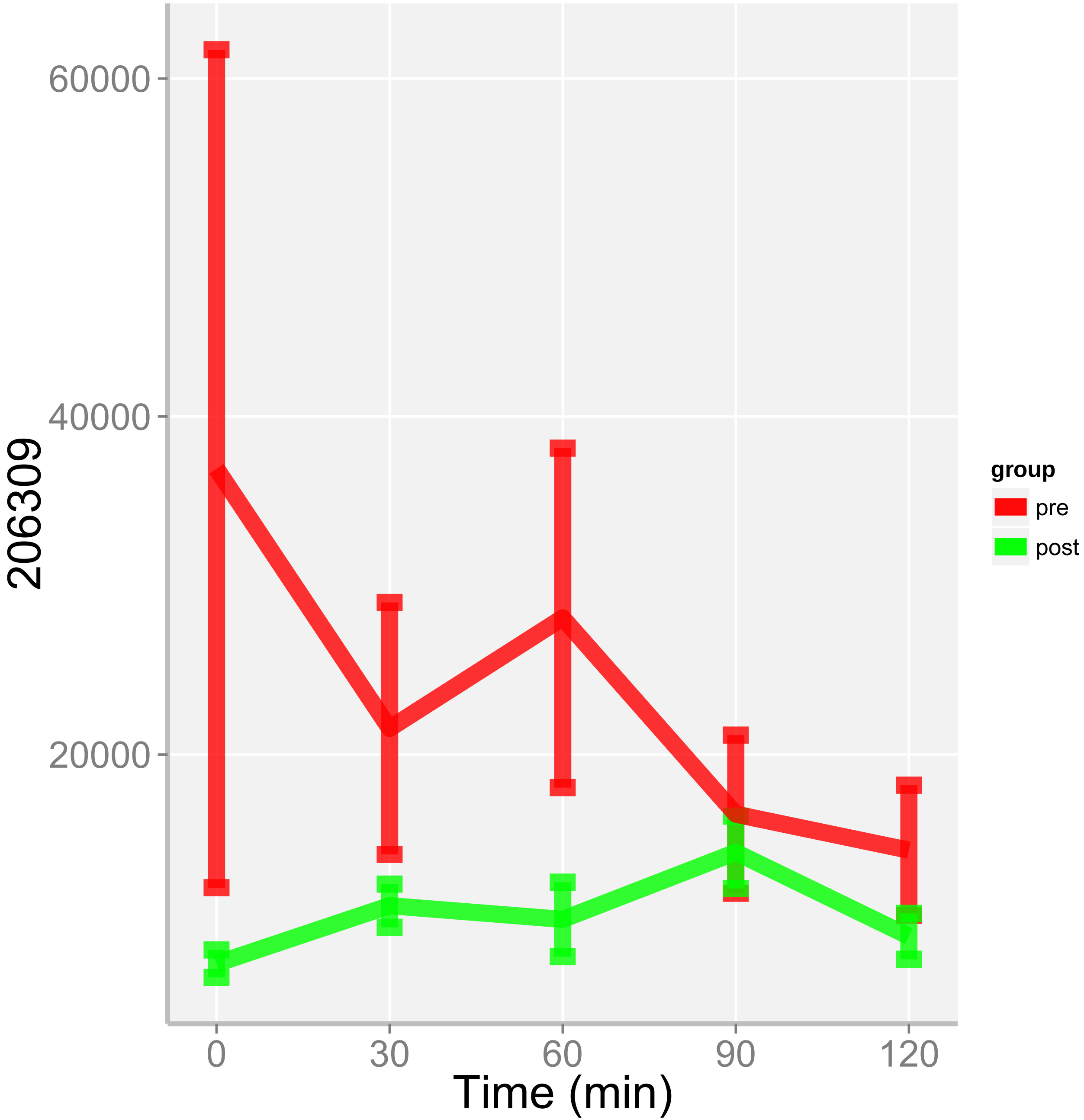

60000
40000
20000
206309
0
30
60
90
120
Time (min)
group
pre
post

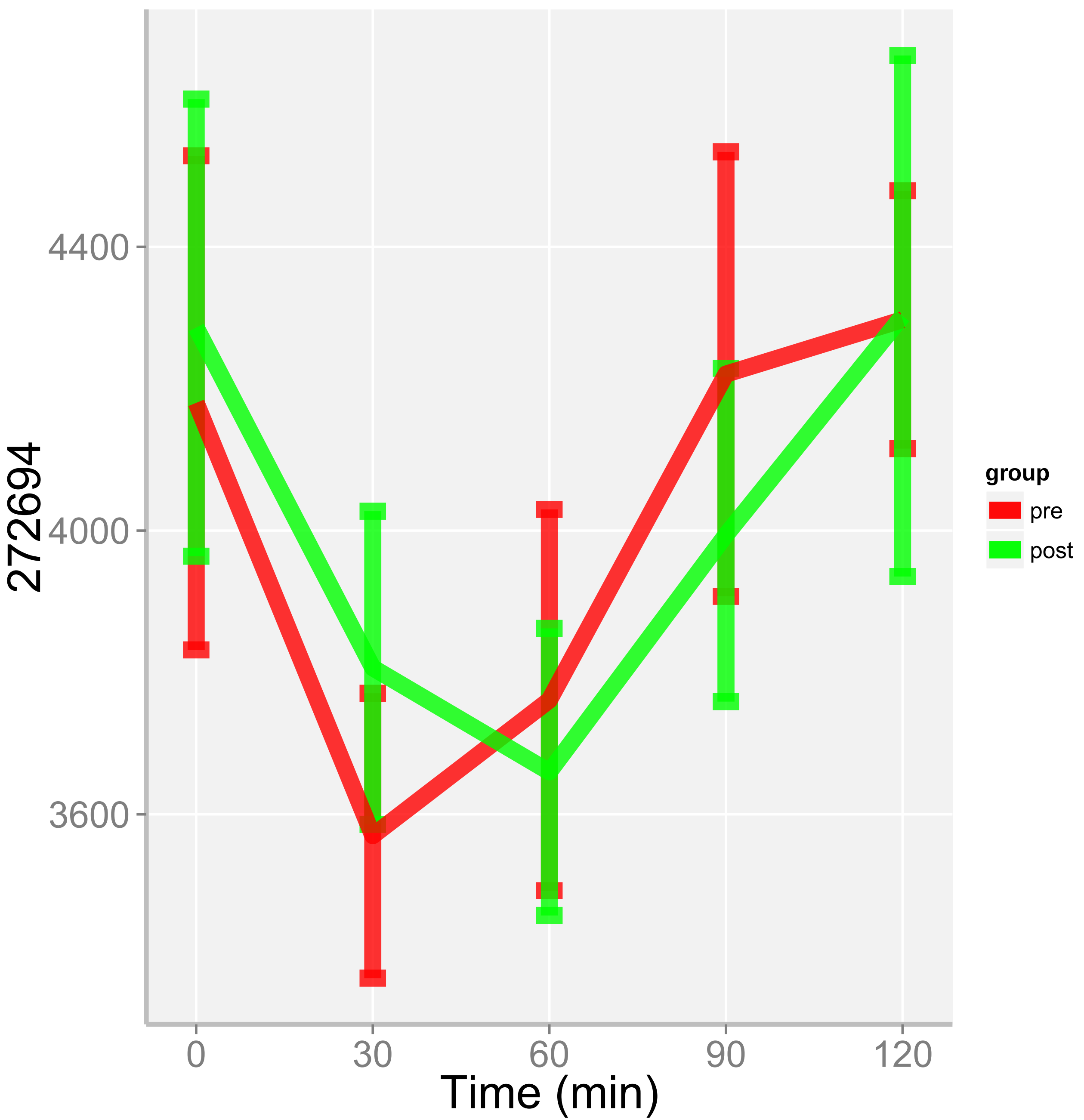

272694
4400
4000
3600
0
30
60
90
120
Time (min)
group
pre
post

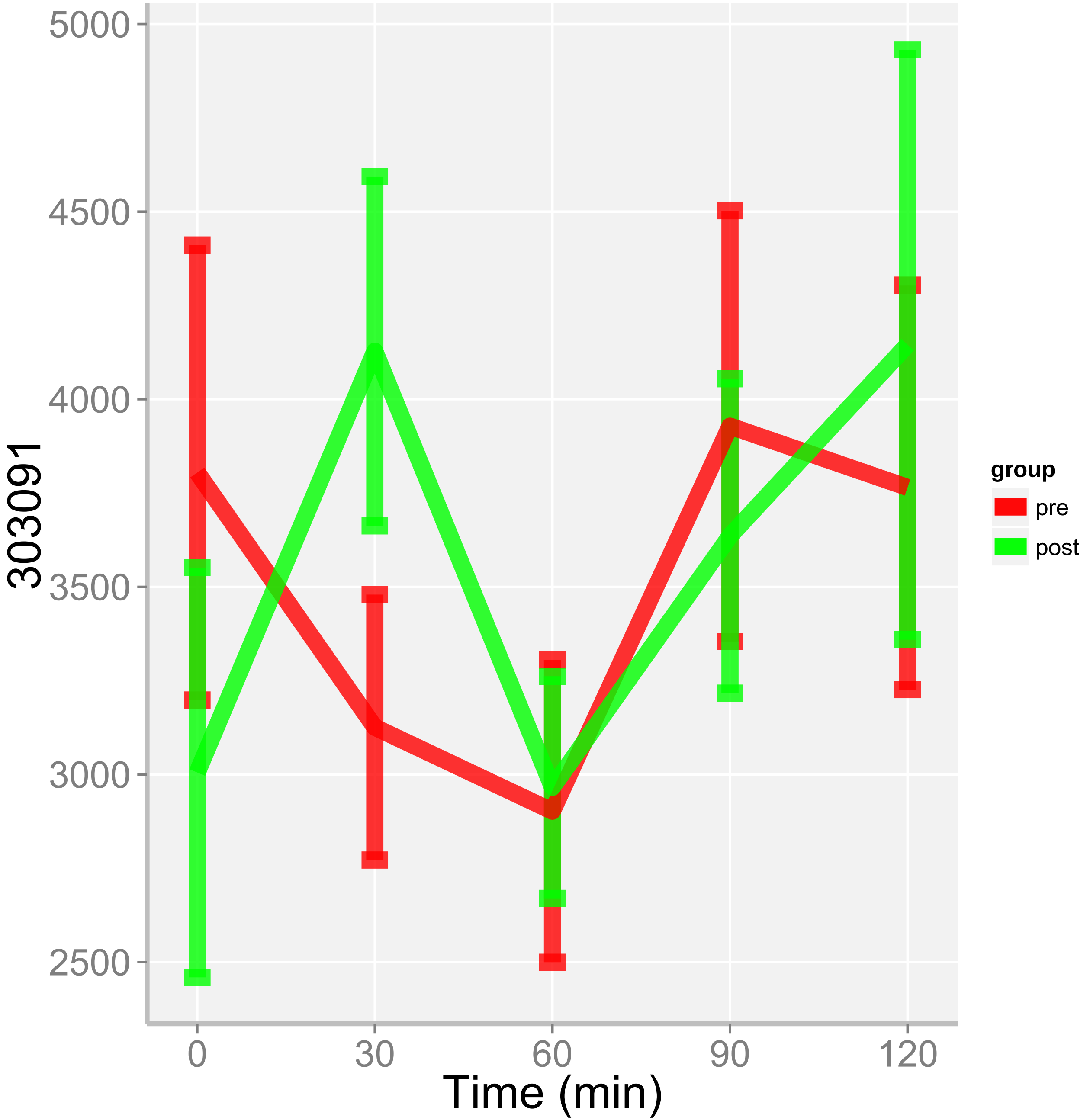

5000
4500
4000
3500
3000
2500
303091
0
30
60
90
120
Time (min)
group
pre
post

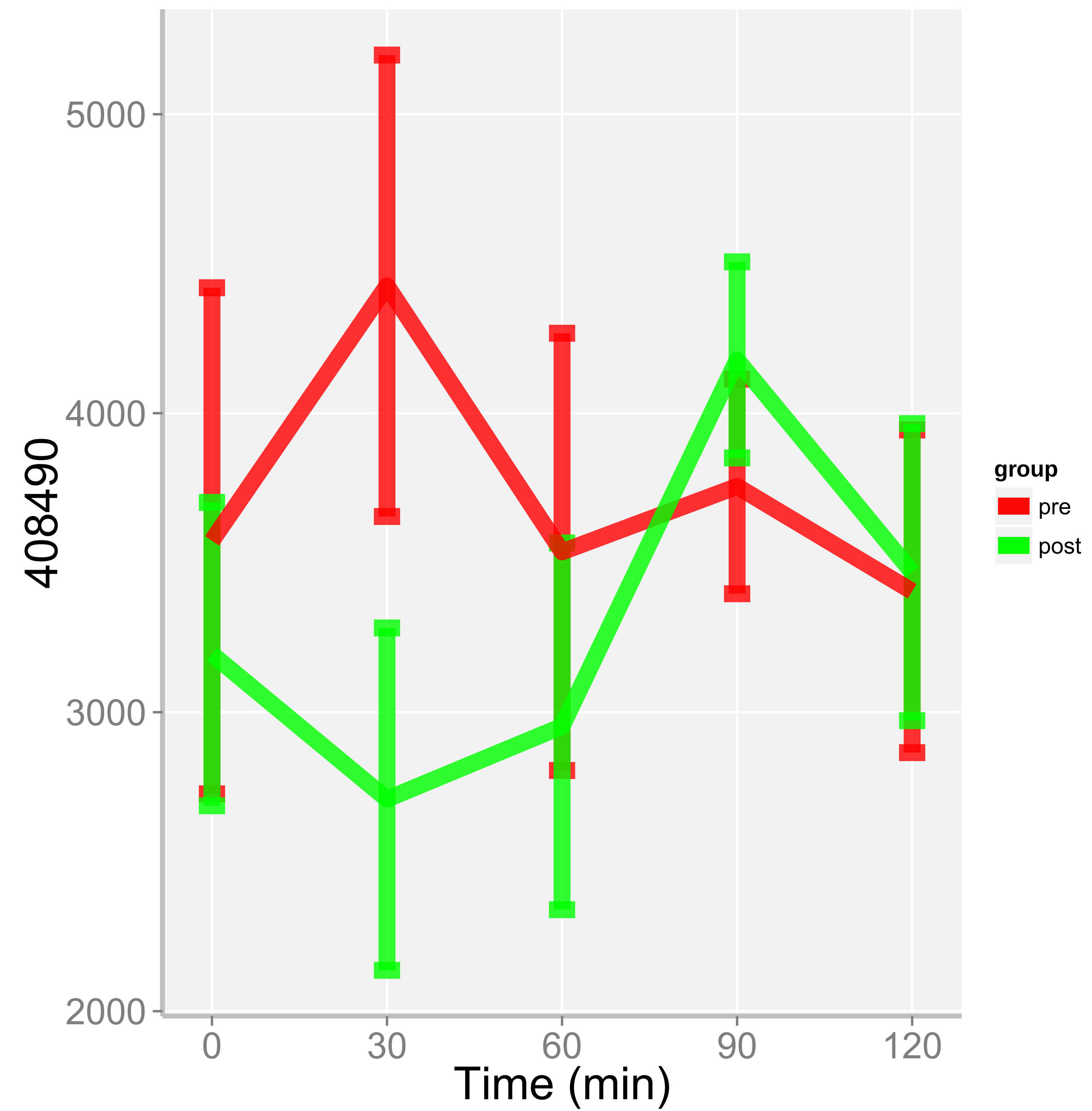
5000
4000
3000
2000
408490
0
30
60
90
120
Time (min)
group
pre
post

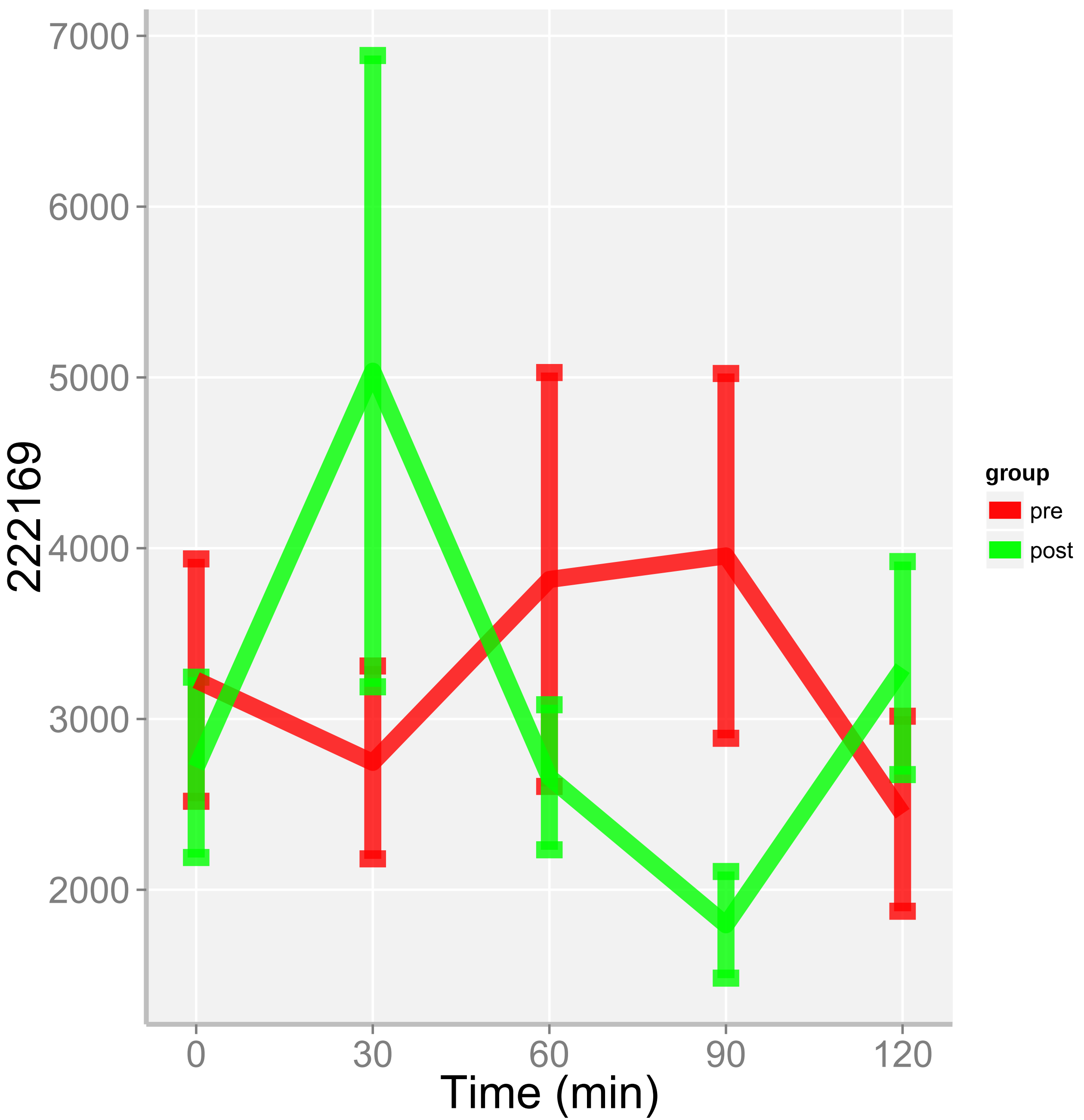
7000
6000
5000
4000
3000
2000
222169
0
30
60
90
120
Time (min)
group
pre
post

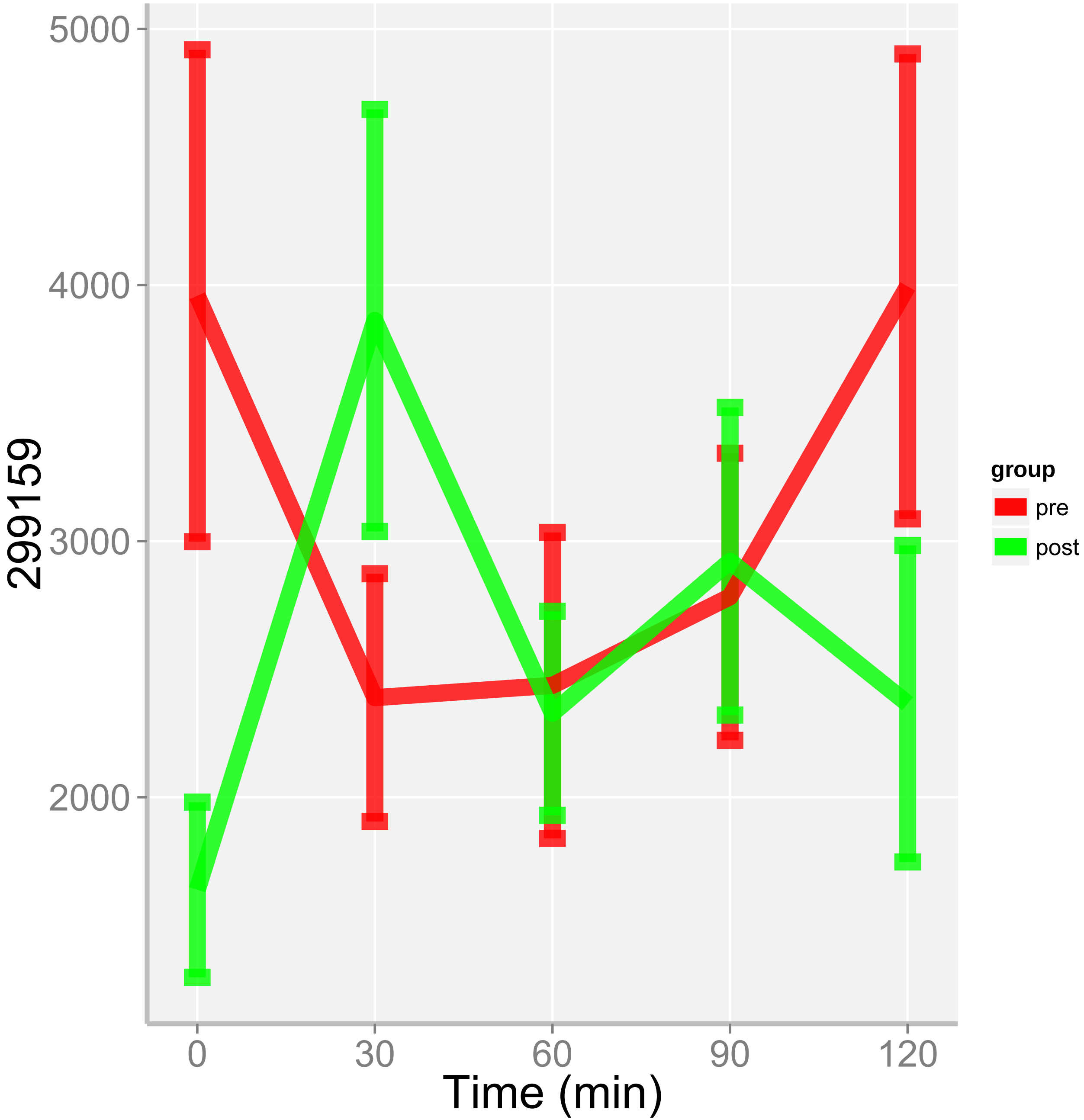

5000
4000
3000
2000
299159
0
30
60
90
120
Time (min)
group
pre
post

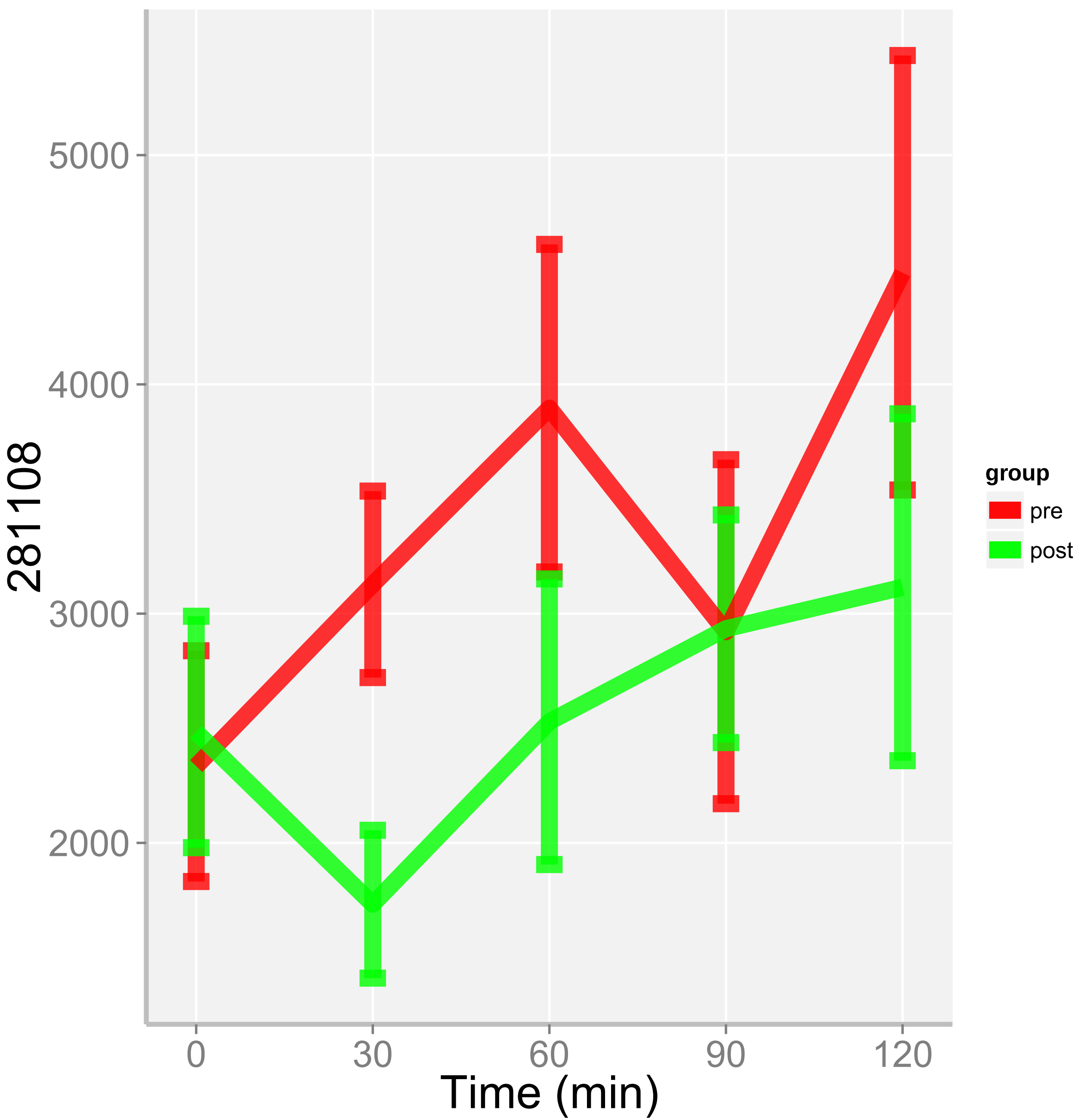

5000
4000
3000
2000
281108
0
30
60
90
120
Time (min)
group
pre
post

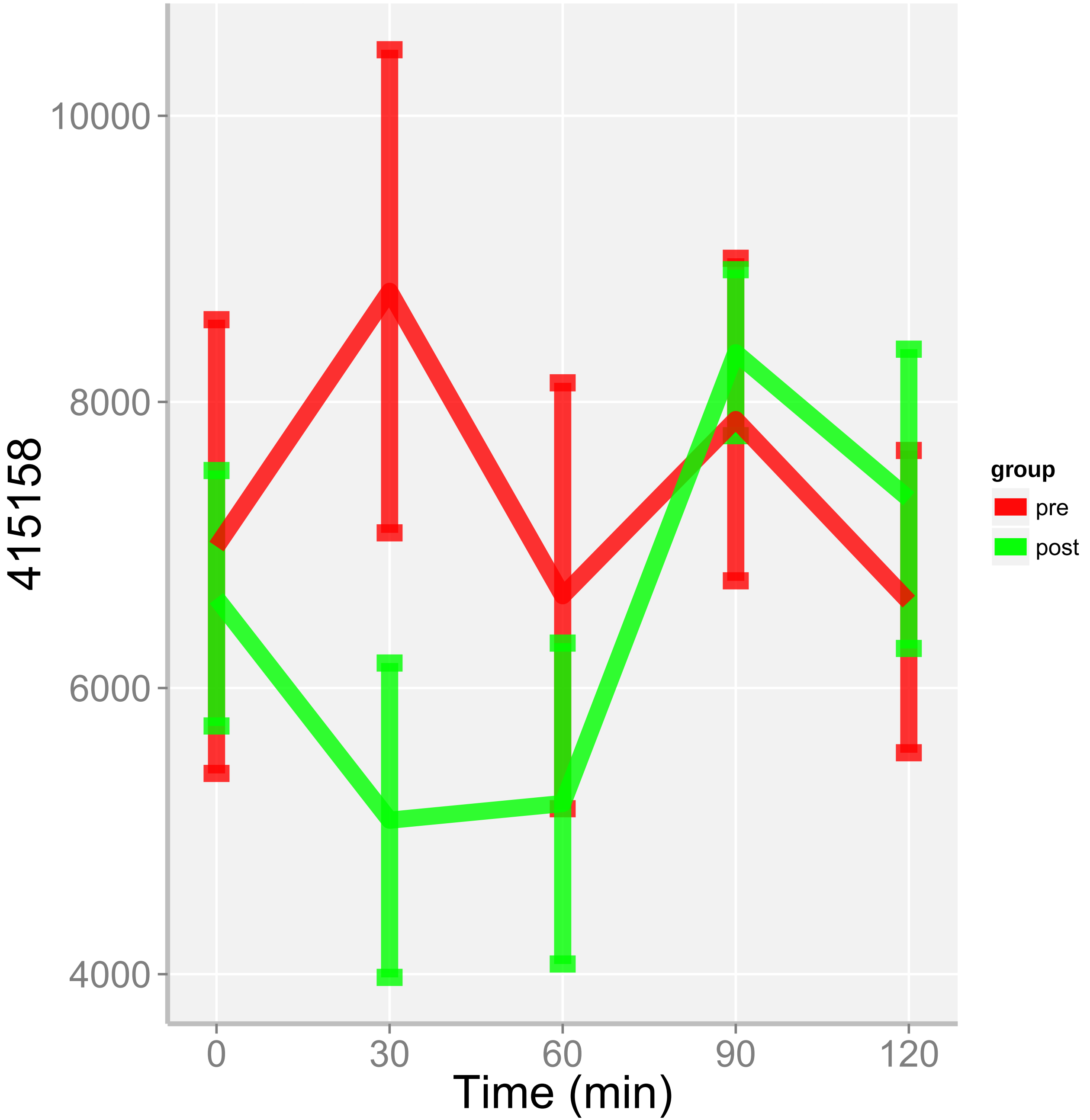

415158
10000
8000
6000
4000
0
30
60
90
120
Time (min)
group
pre
post

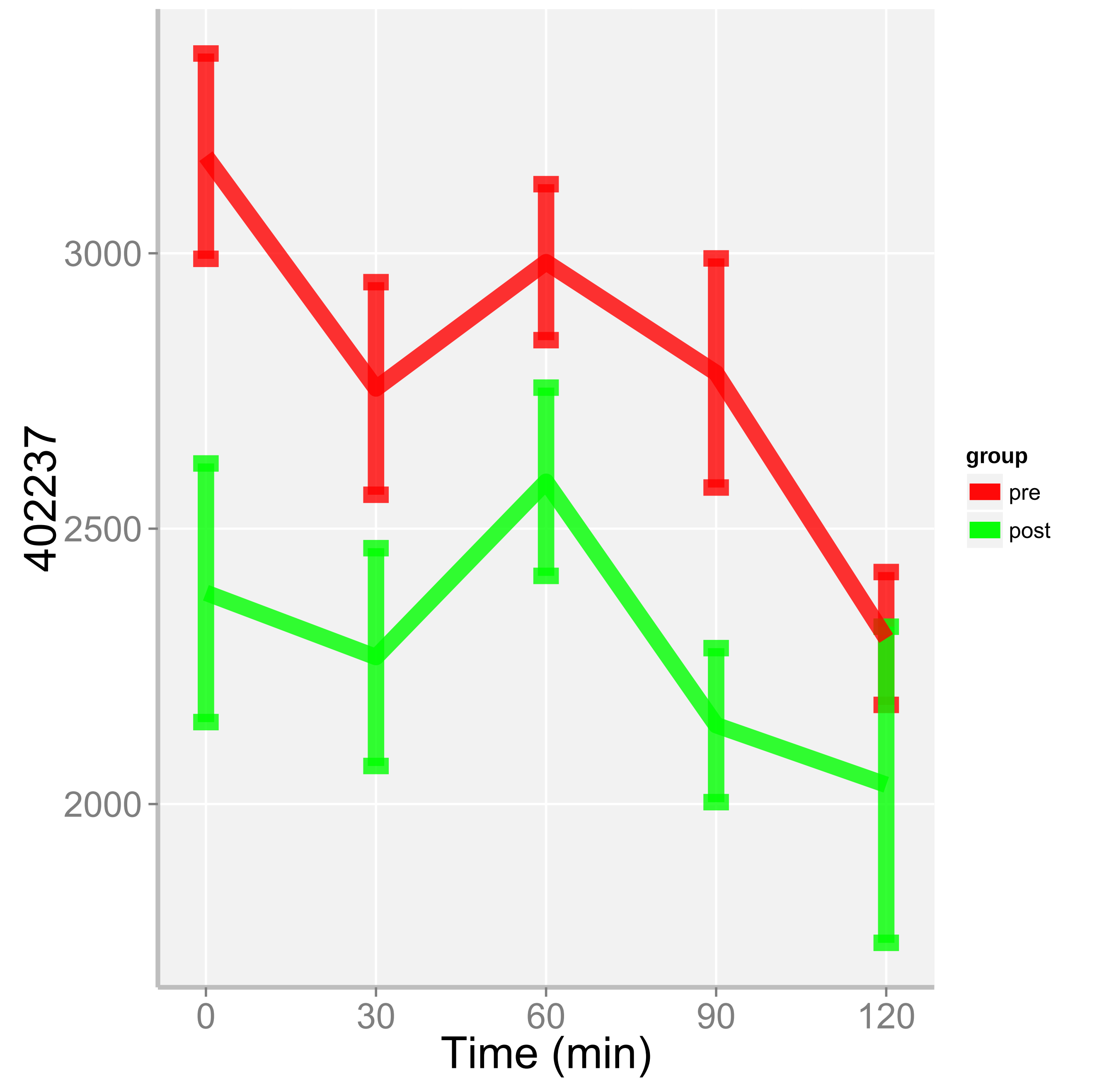
402237
3000
2500
2000
0
30
60
90
120
Time (min)
group
pre
post

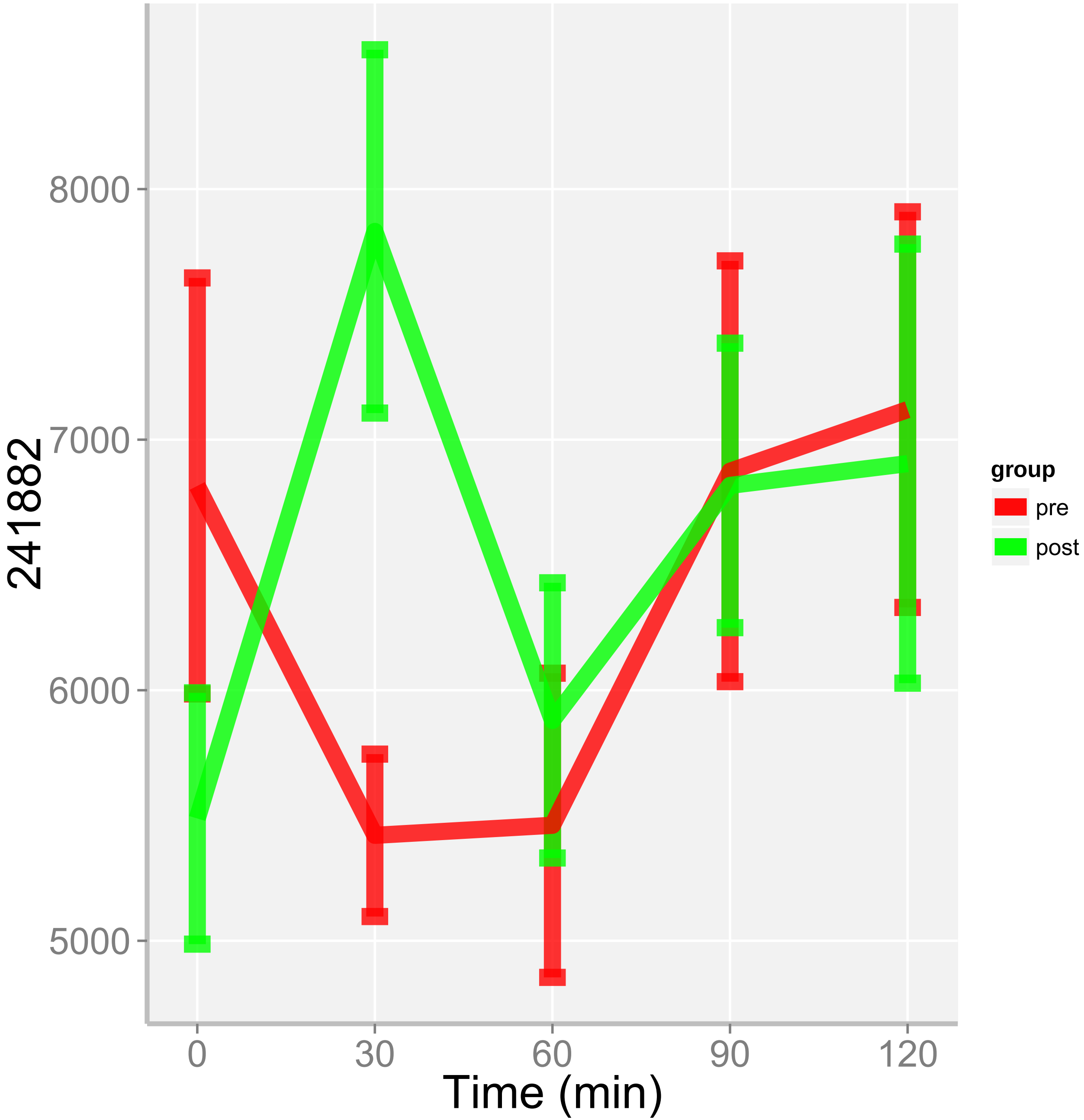

8000
7000
6000
5000
241882
0
30
60
90
120
Time (min)
group
pre
post

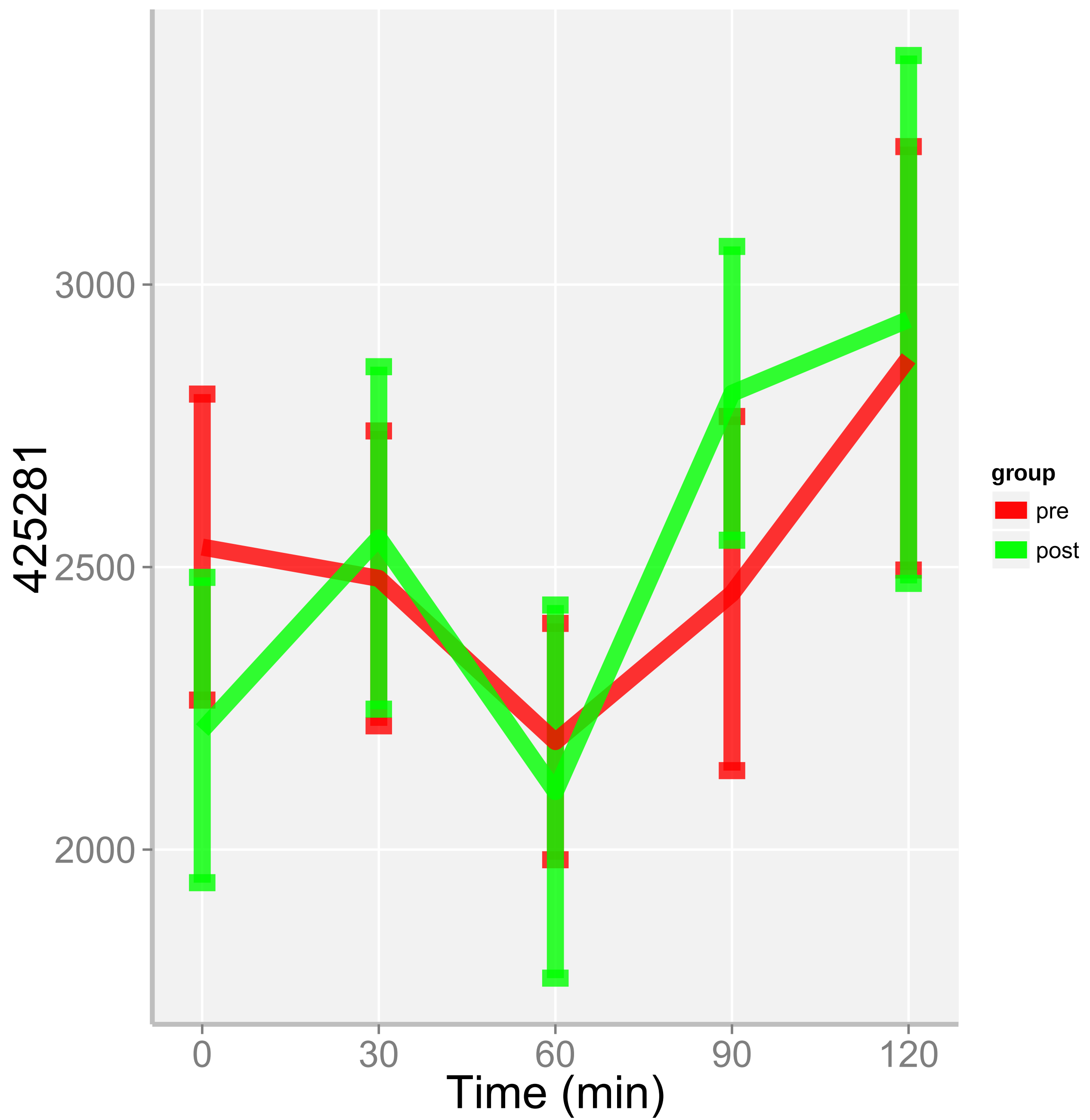

425281
3000
2500
2000
0
30
60
90
120
Time (min)
group
pre
post

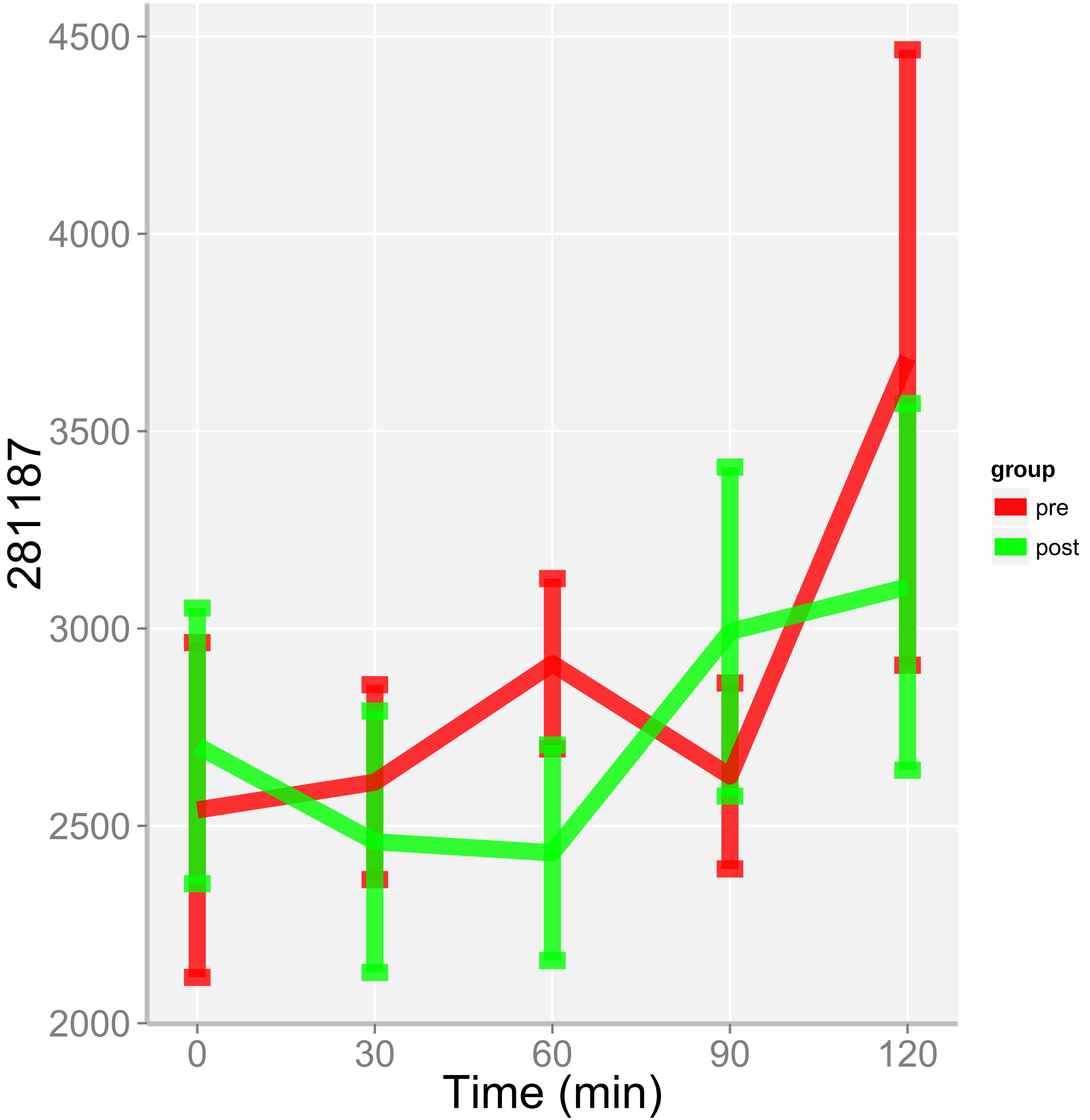

281187
4500
4000
3500
3000
2500
2000
0
30
60
90
120
Time (min)
group
pre
post

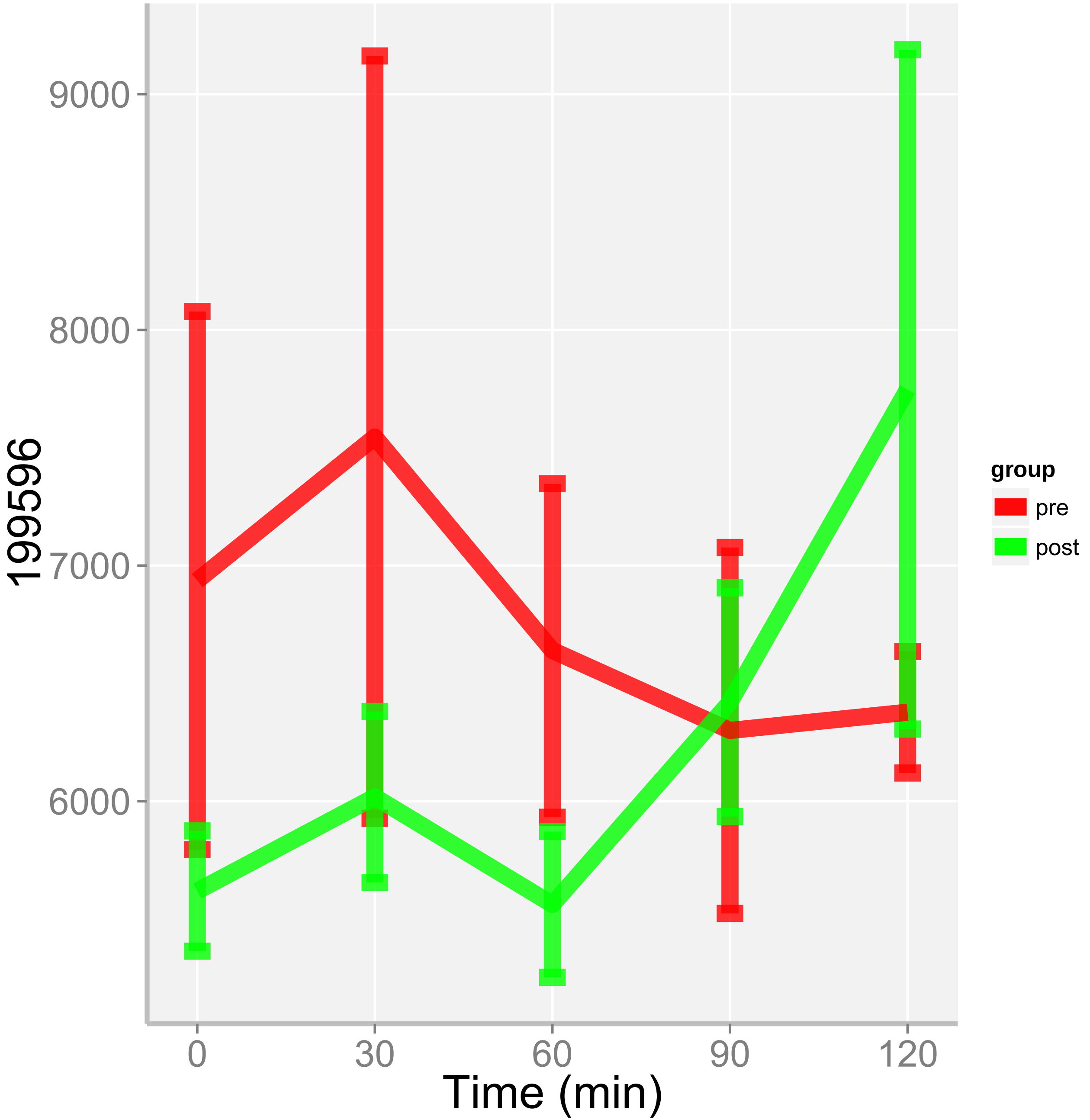

199596
9000
8000
7000
6000
0
30
60
90
120
Time (min)
group
pre
post

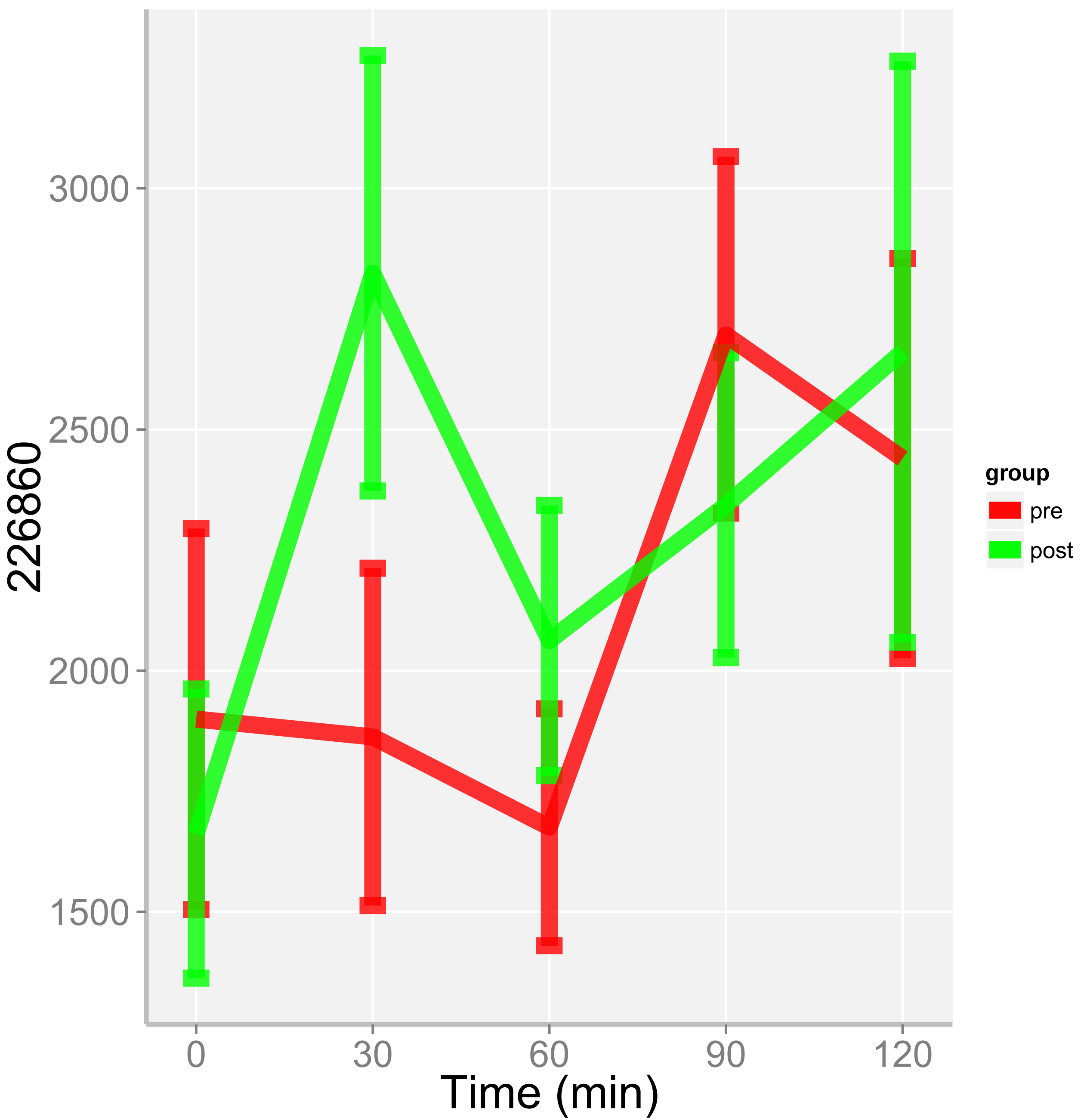

226860
3000
2500
2000
1500
0
30
60
90
120
Time (min)
group
pre
post

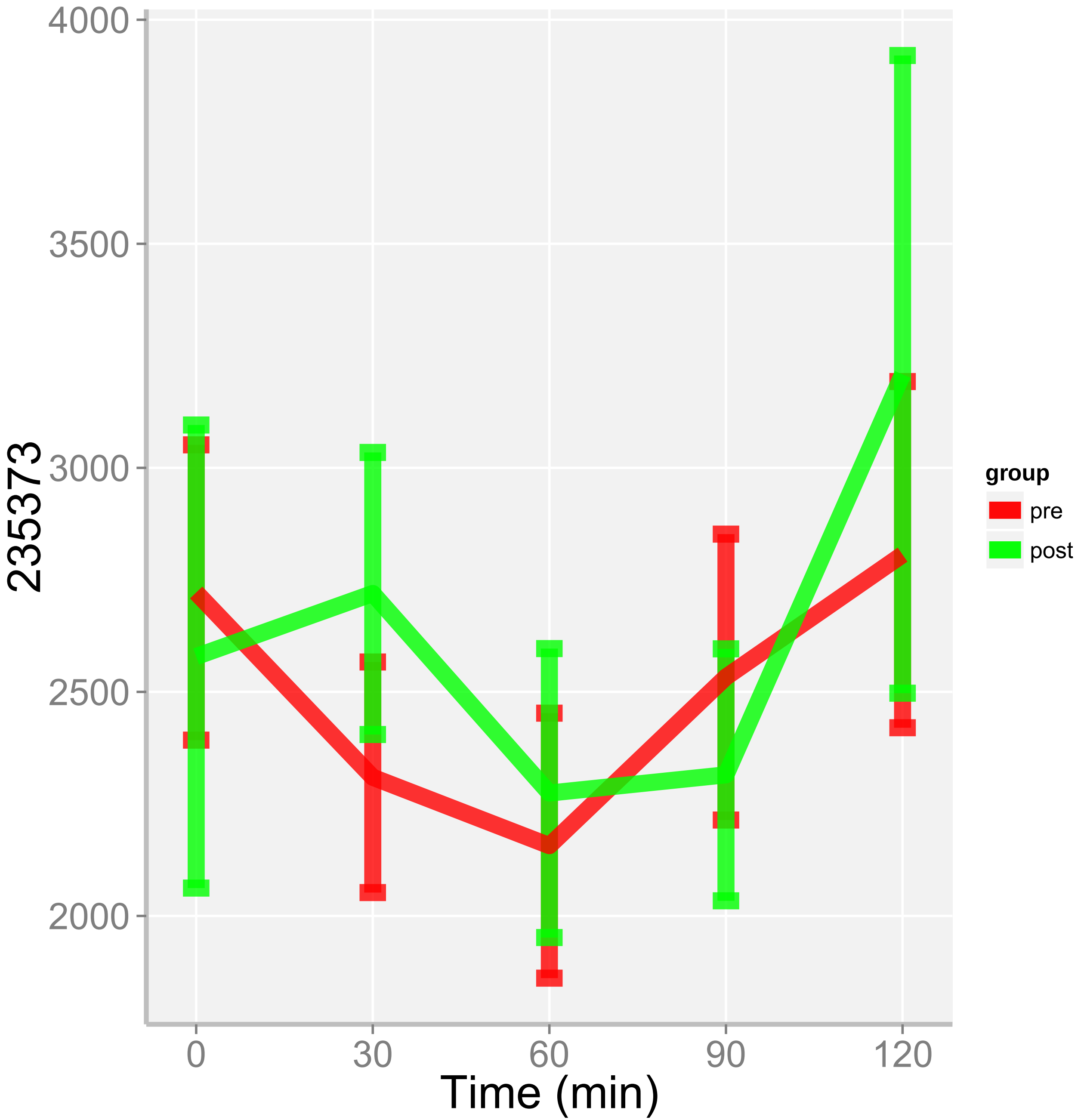

4000
3500
3000
2500
2000
235373
0
30
60
90
120
Time (min)
group
pre
post

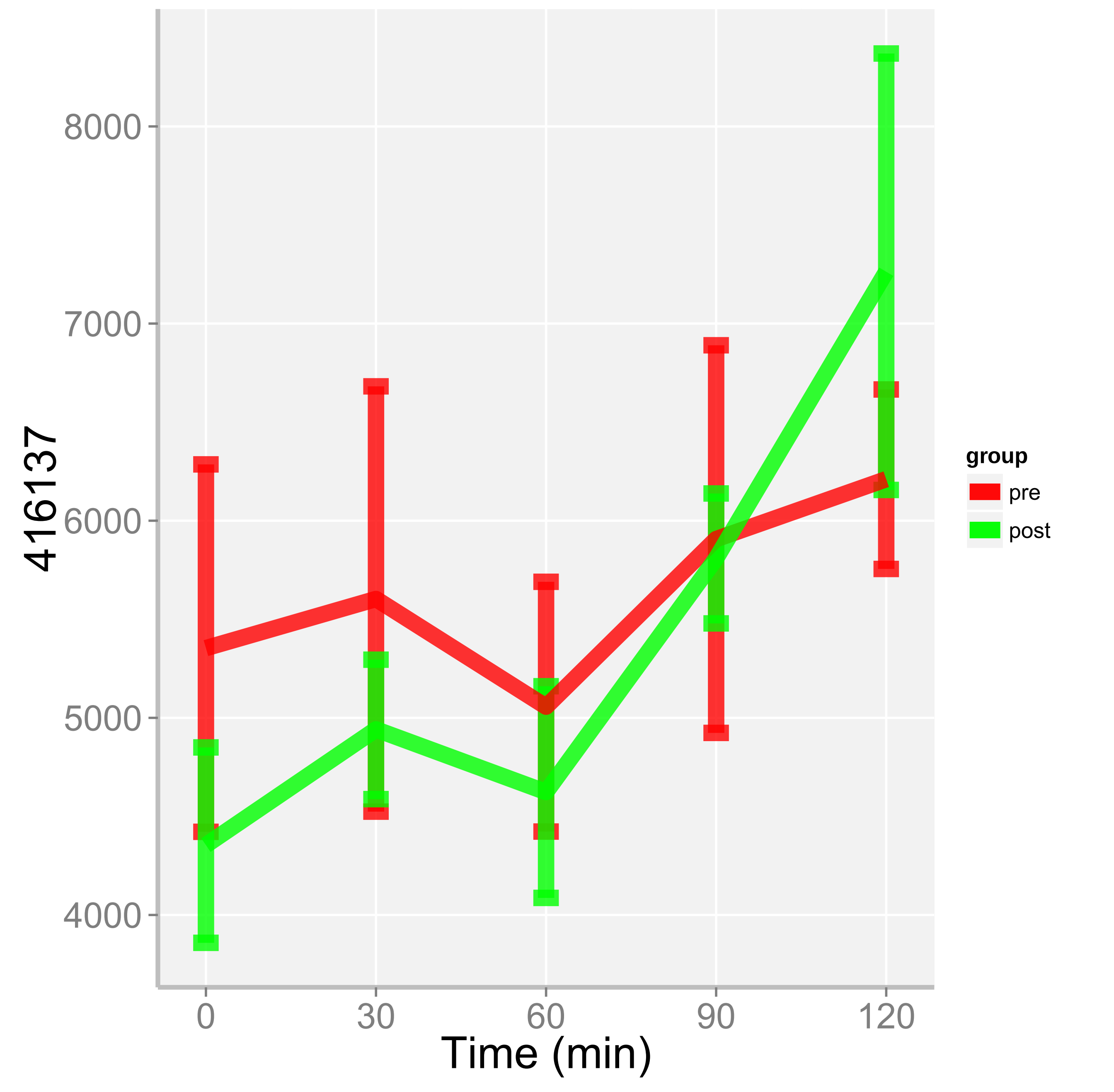

4161137
8000
7000
6000
5000
4000
0
30
60
90
120
Time (min)
group
pre
post

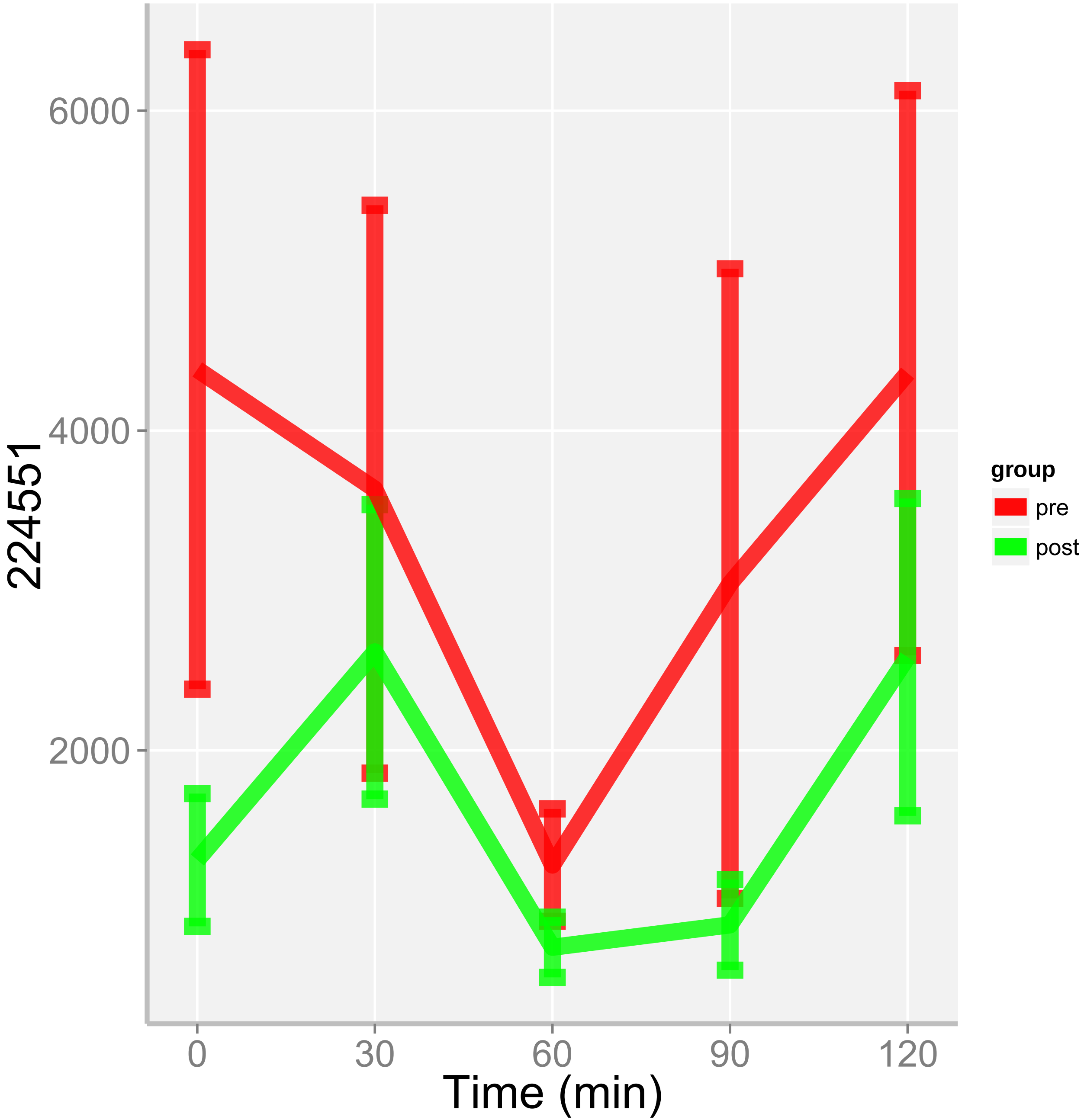

224551
6000
4000
2000
0
30
60
90
120
Time (min)
group
pre
post

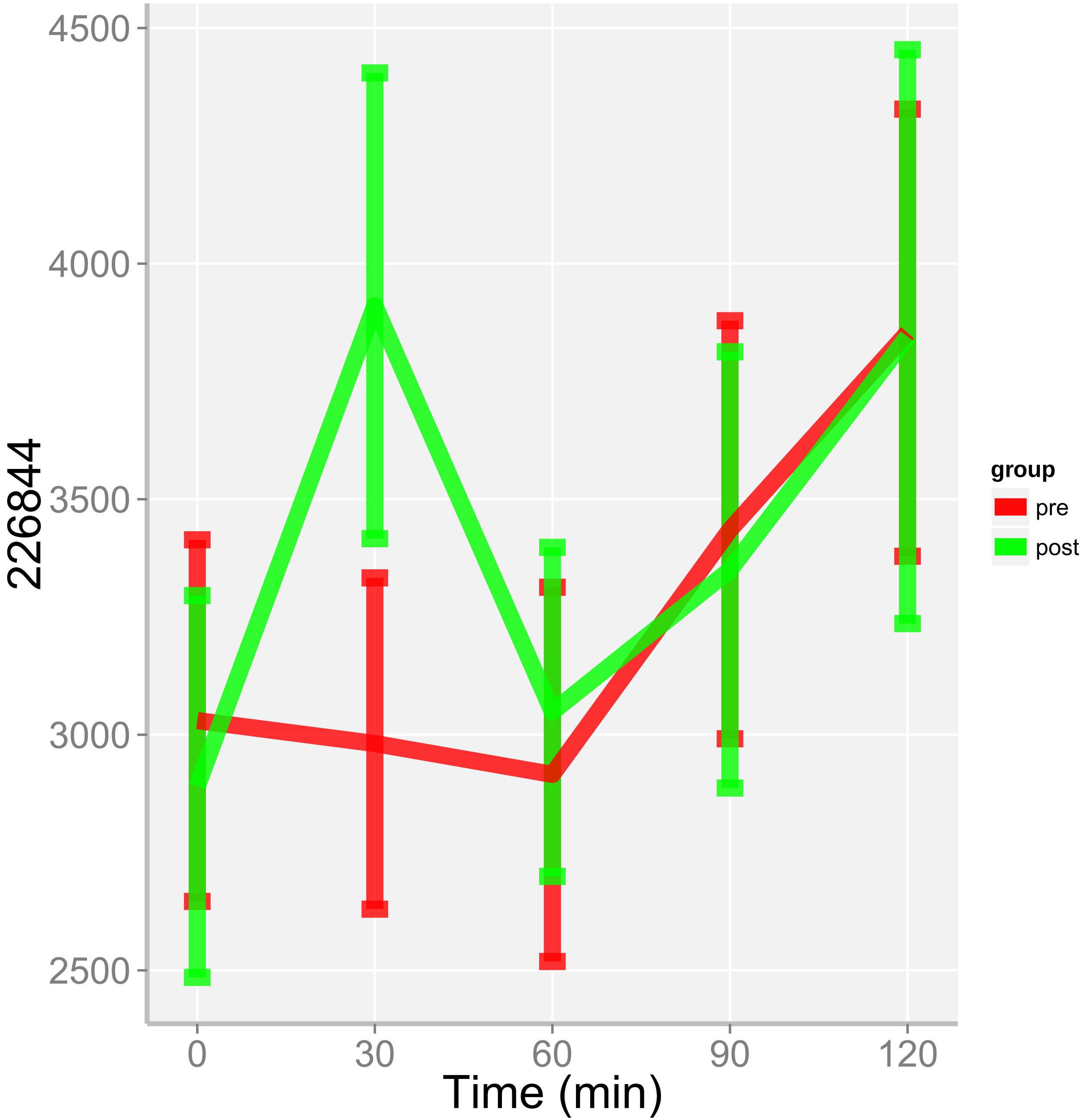

4500
4000
3500
3000
2500
226844
0
30
60
90
120
Time (min)
group
pre
post

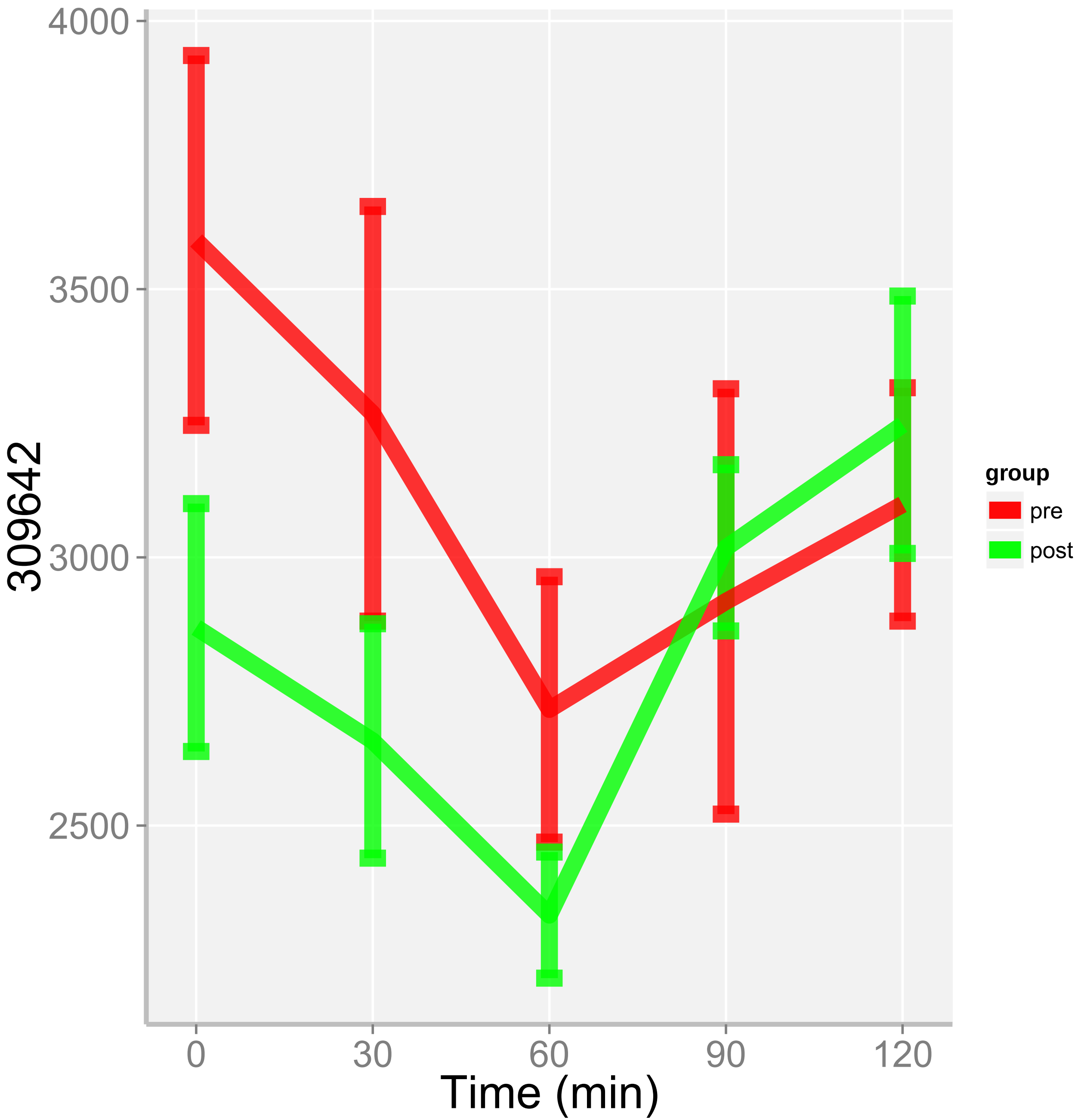
4000
3500
3000
2500
309642
0
30
60
90
120
Time (min)
group
pre
post

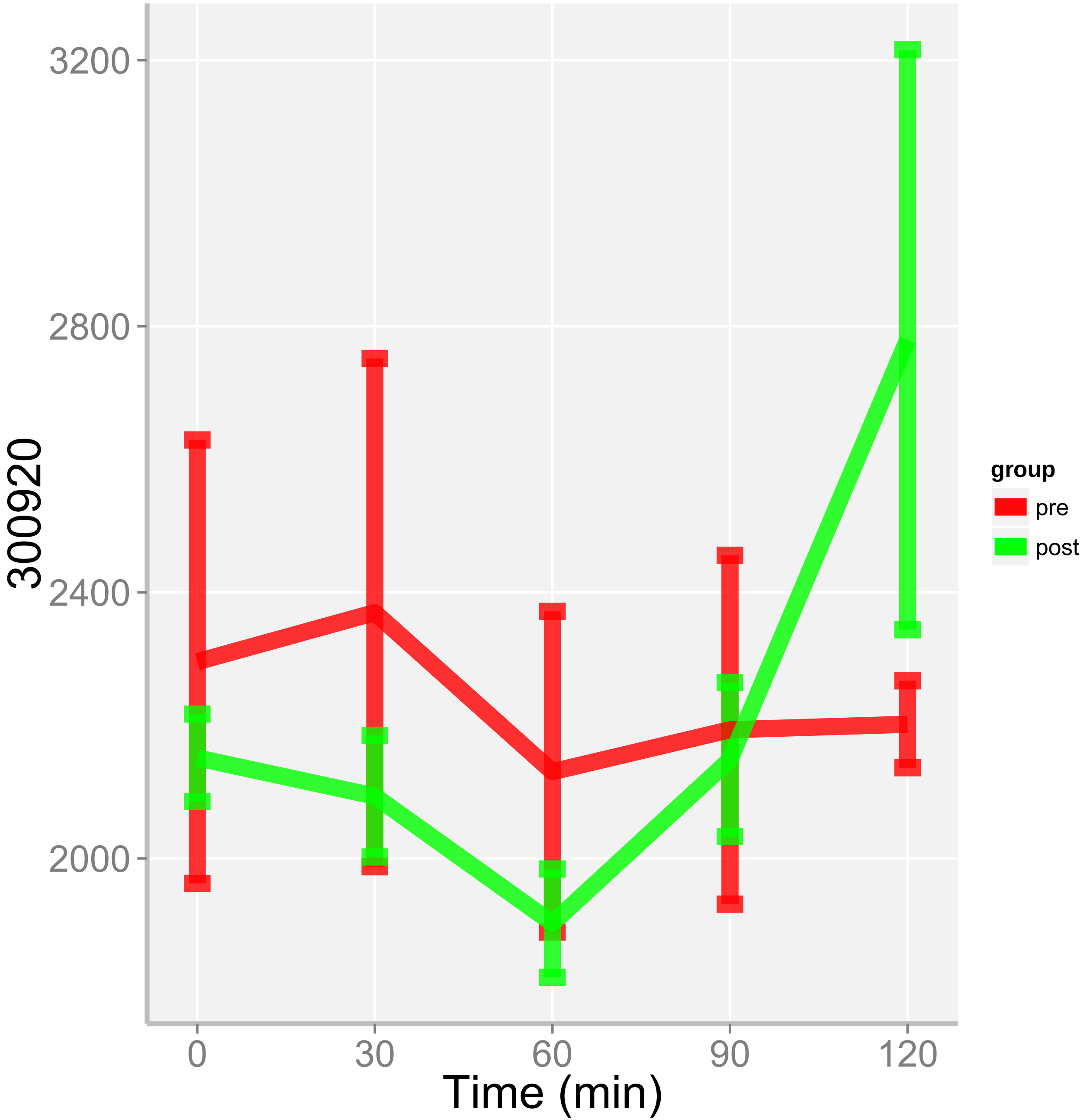

3200
2800
2400
2000
300920
0
30
60
90
120
Time (min)
group
pre
post

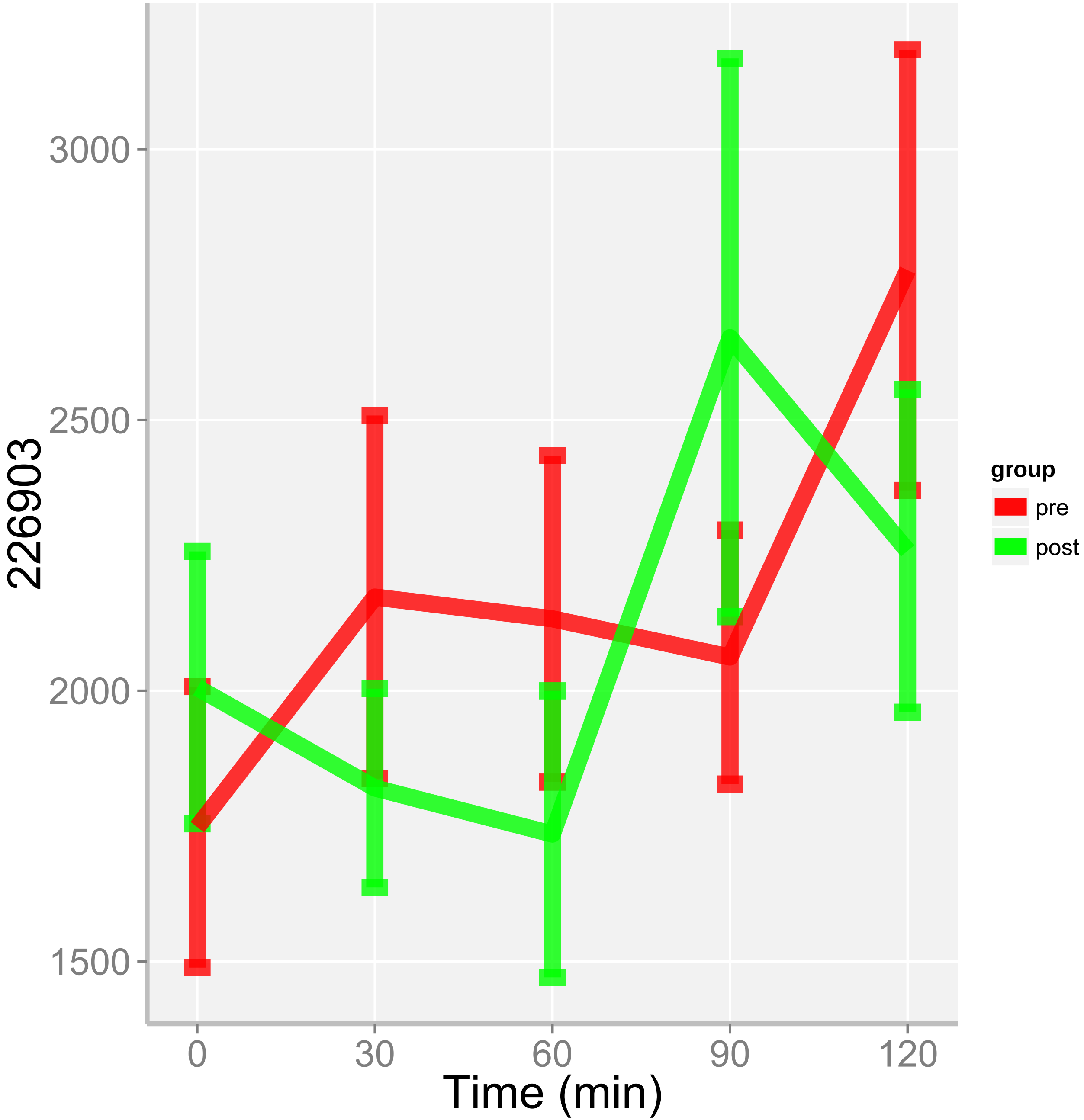

3000
2500
2000
1500
226903
0
30
60
90
120
Time (min)
group
pre
post

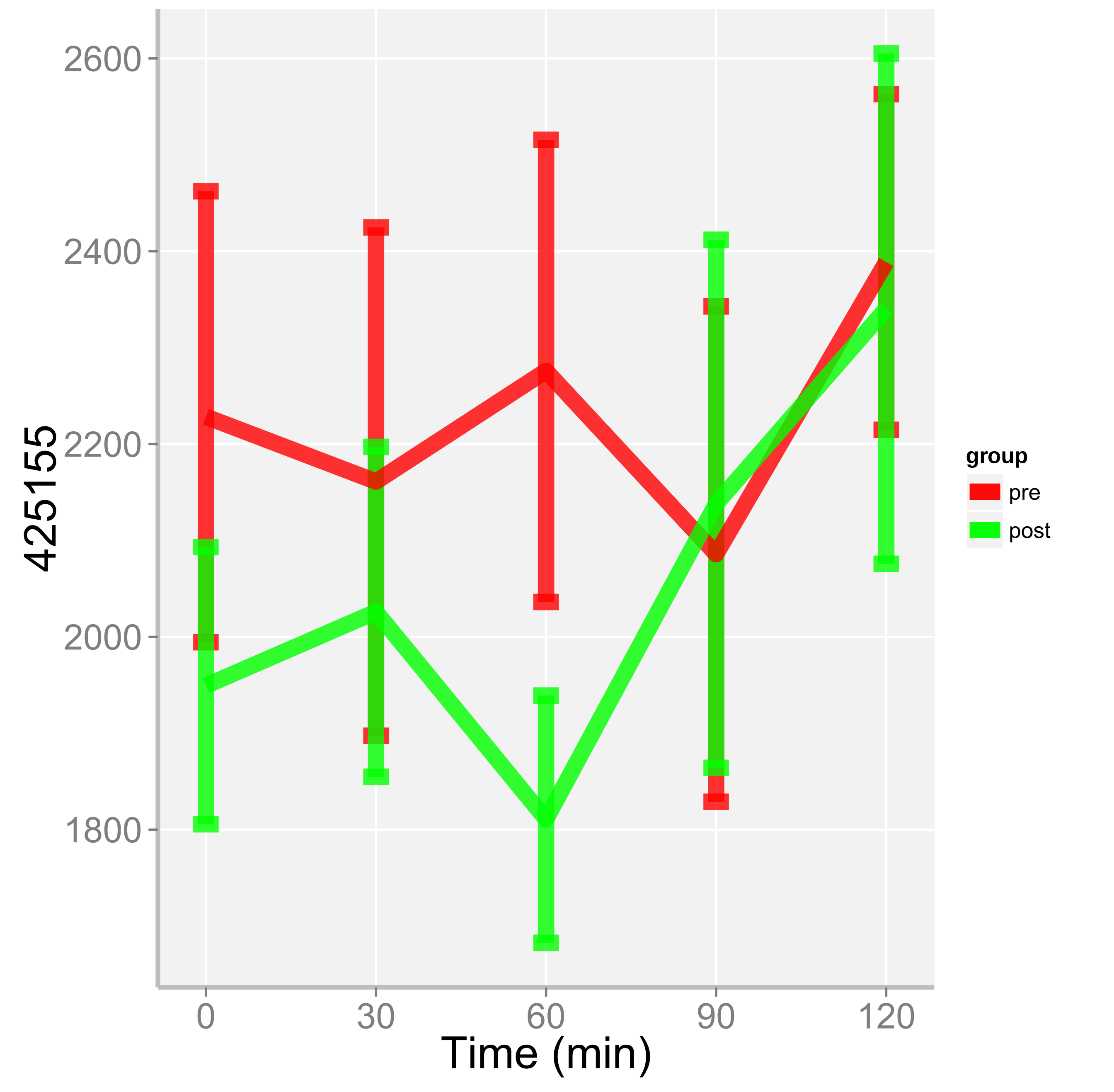

4251155
2600
2400
2200
2000
1800
0
30
60
90
120
Time (min)
group
pre
post

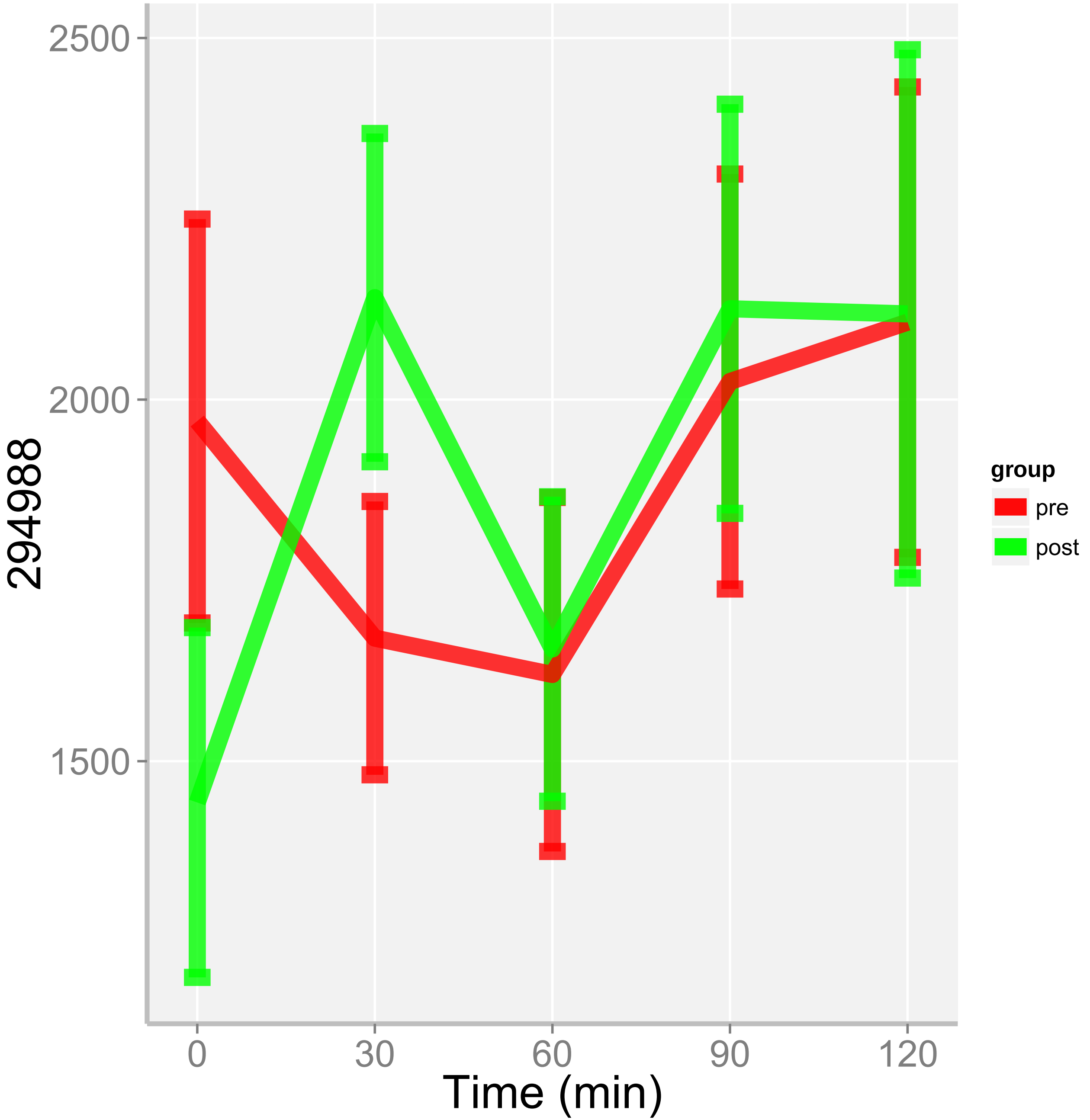

294988
2500
2000
1500
0
30
60
90
120
Time (min)
group
pre
post

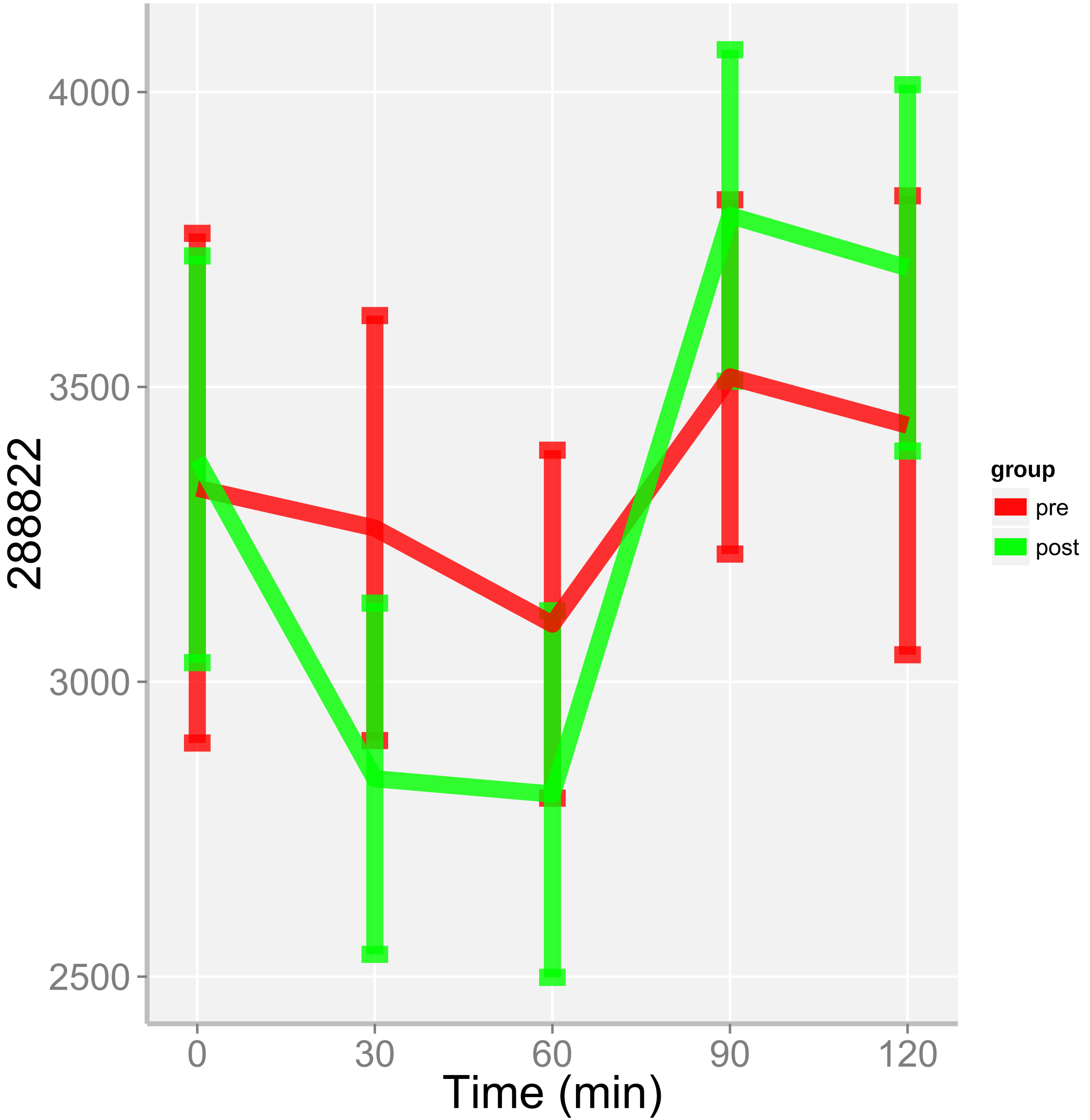

4000
3500
3000
2500
288822
0
30
60
90
120
Time (min)
group
pre
post

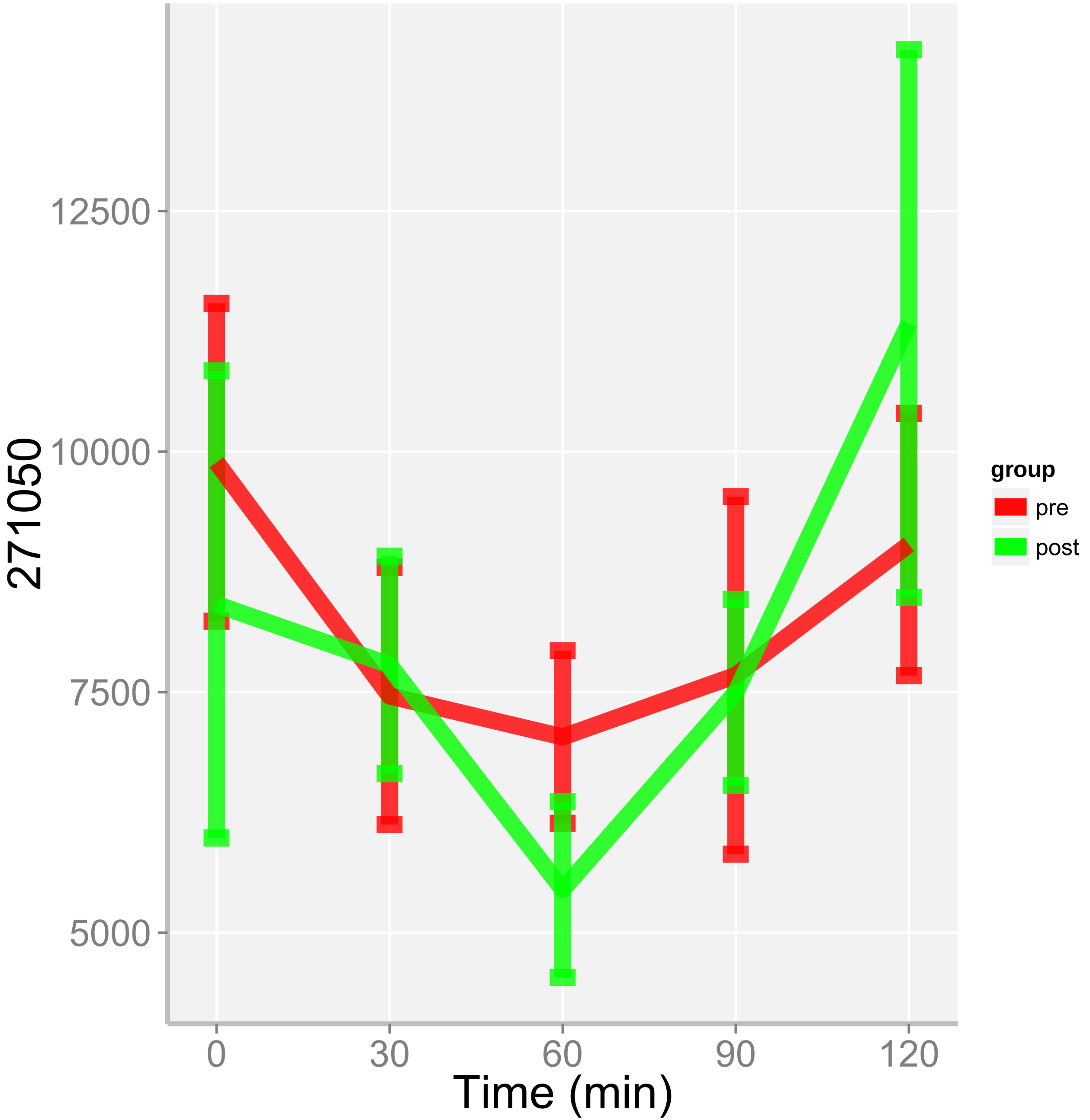

271050
12500
10000
7500
5000
0
30
60
90
120
Time (min)
group
pre
post

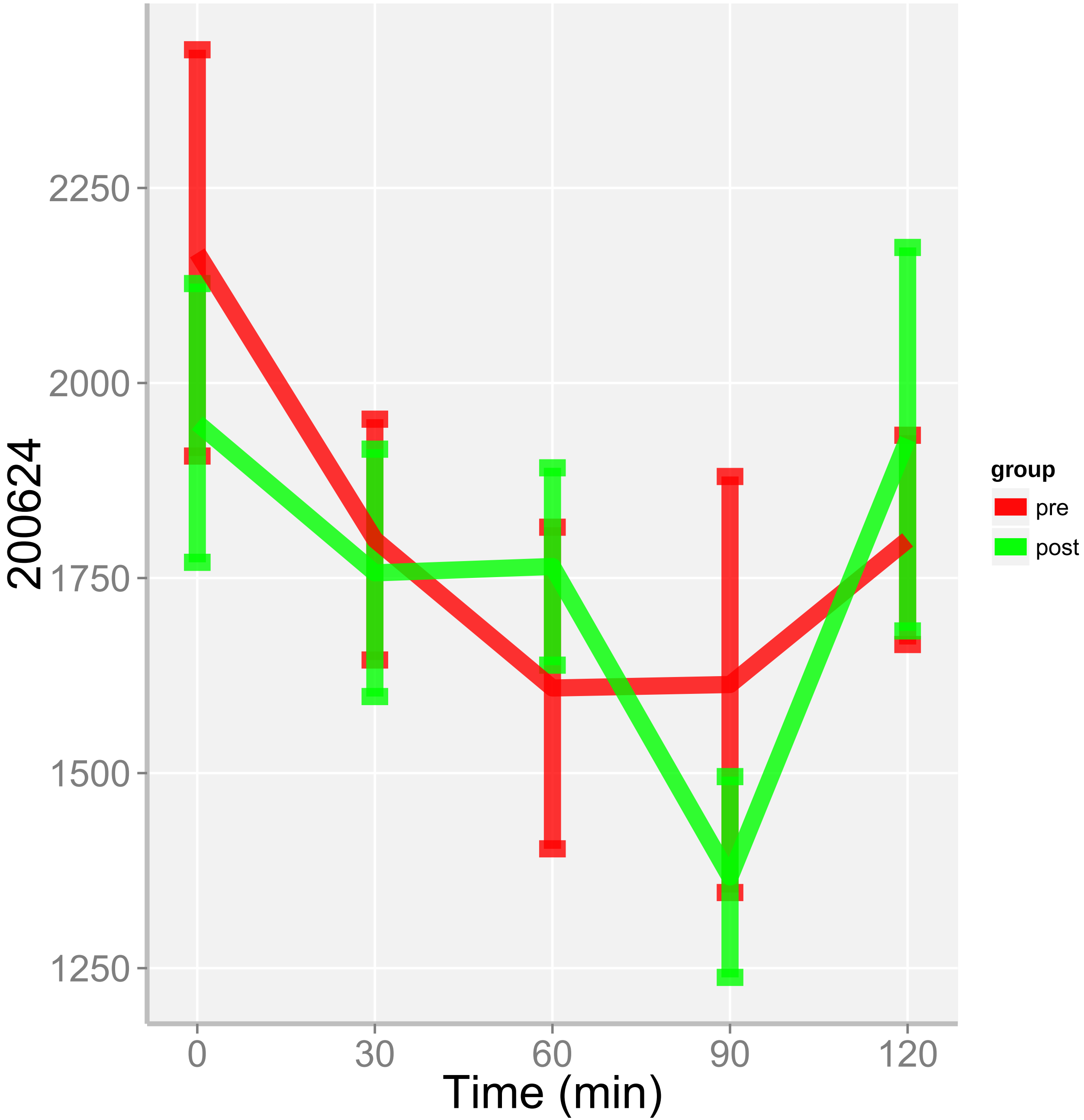
2250
2000
1750
1500
1250
200624
0
30
60
90
120
Time (min)
group
pre
post

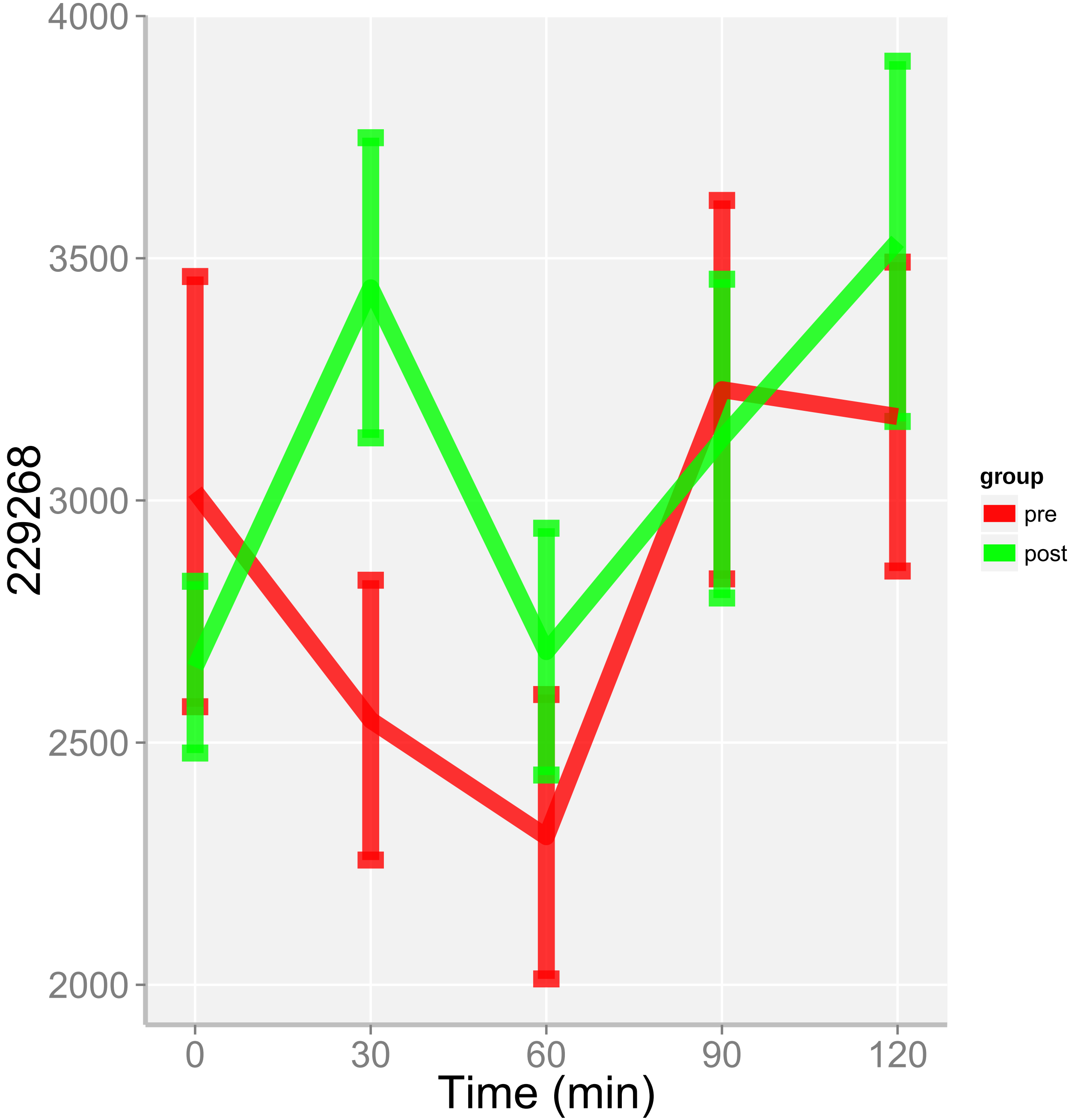

4000
3500
3000
2500
2000
229268
0
30
60
90
120
Time (min)
group
pre
post

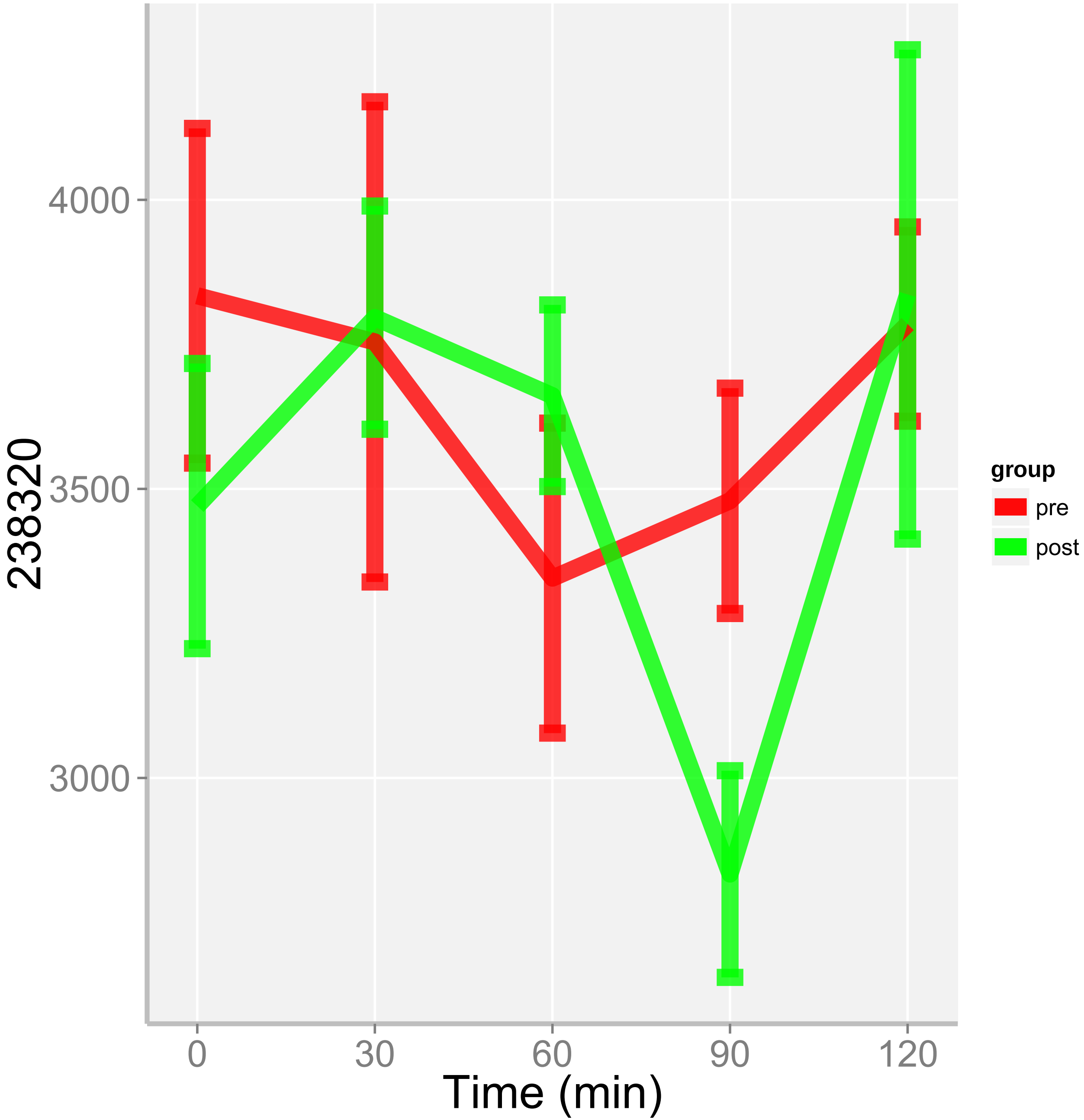

238320
4000
3500
3000
0
30
60
90
120
Time (min)
group
pre
post

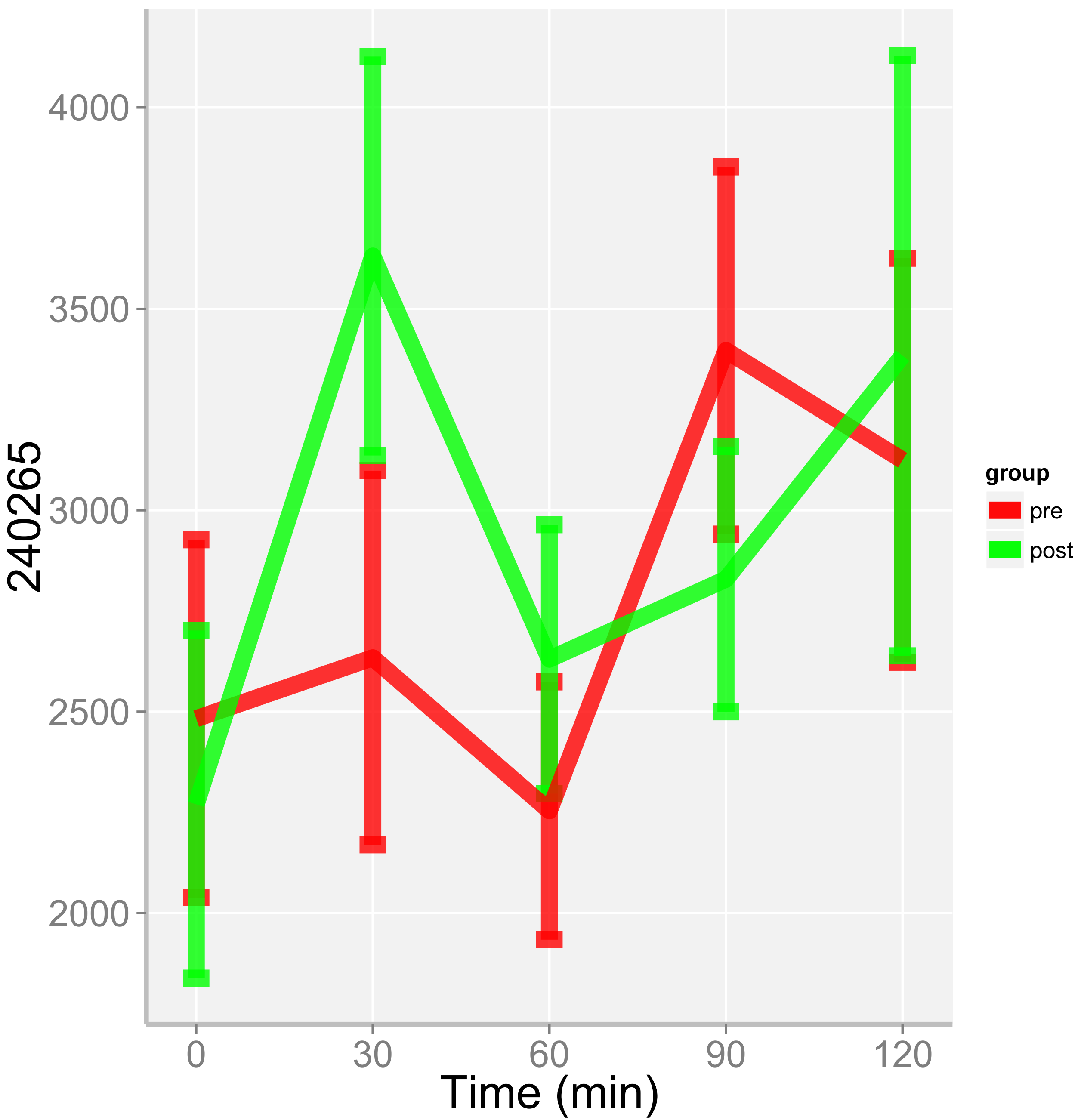

4000
3500
3000
2500
2000
2402655
0
30
60
90
120
Time (min)
group
pre
post

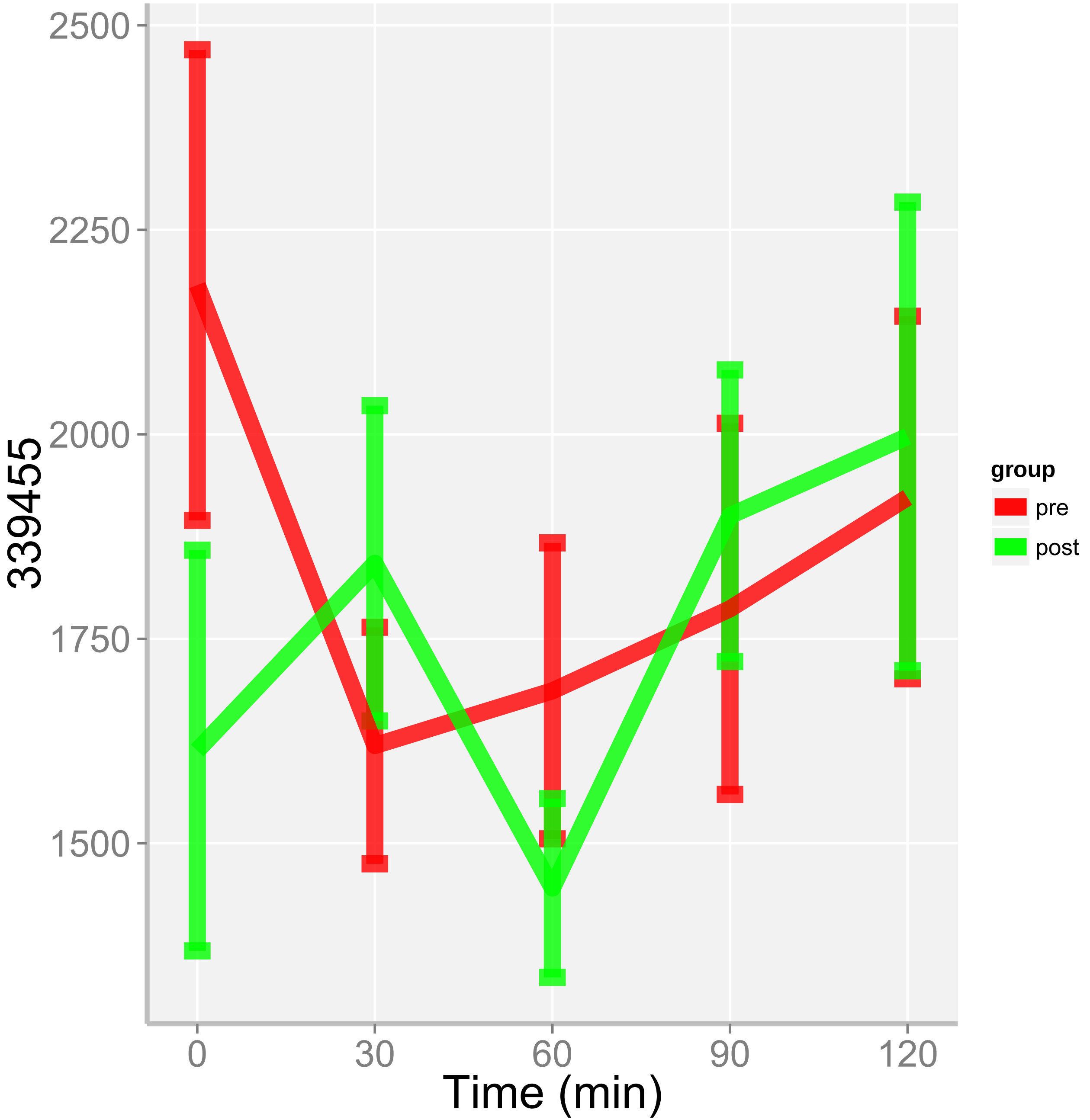

2500
2250
2000
1750
1500
339455
0
30
60
90
120
Time (min)
group
pre
post

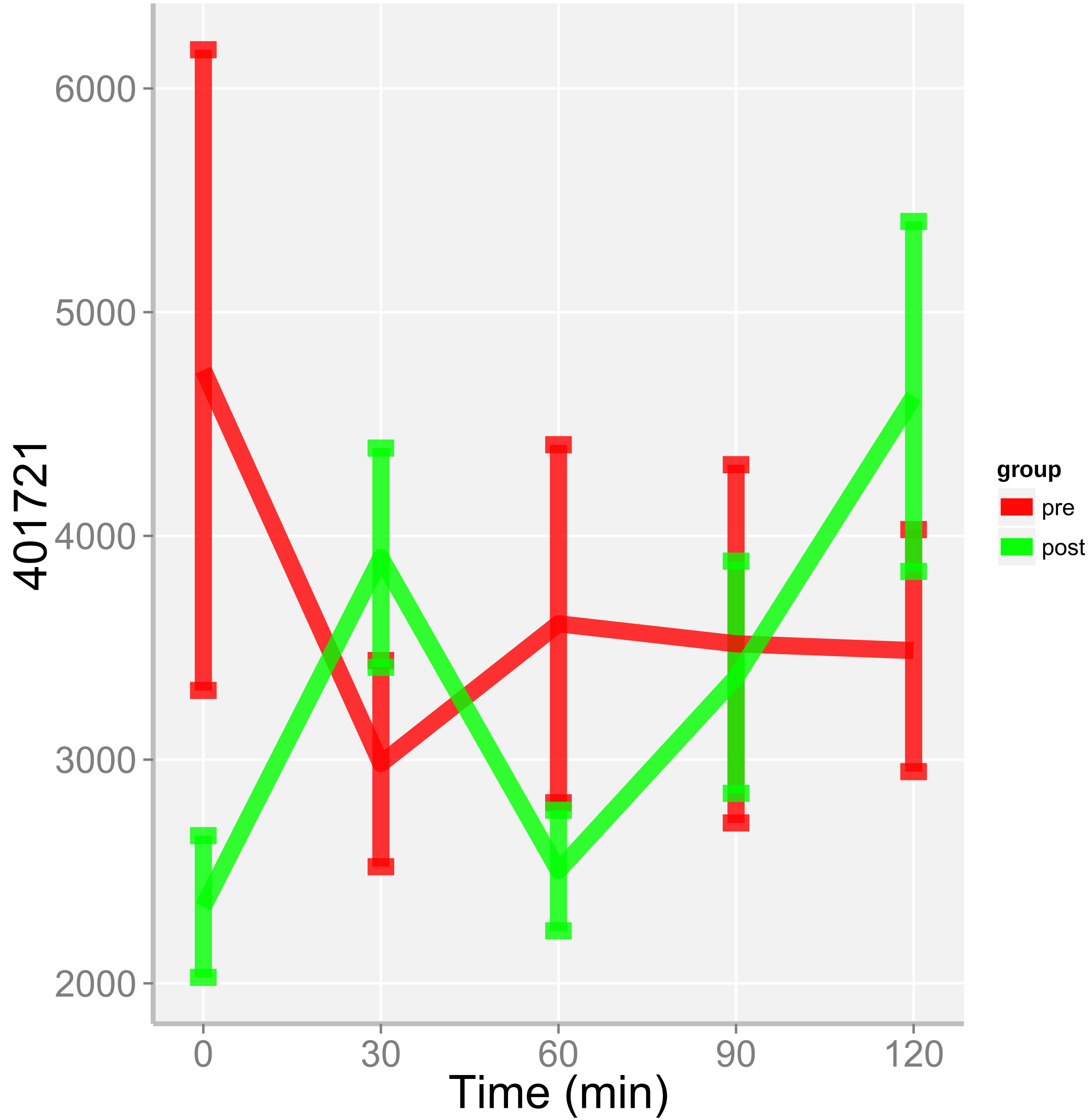
6000
5000
4000
3000
2000
401721
0
30
60
90
120
Time (min)
group
pre
post

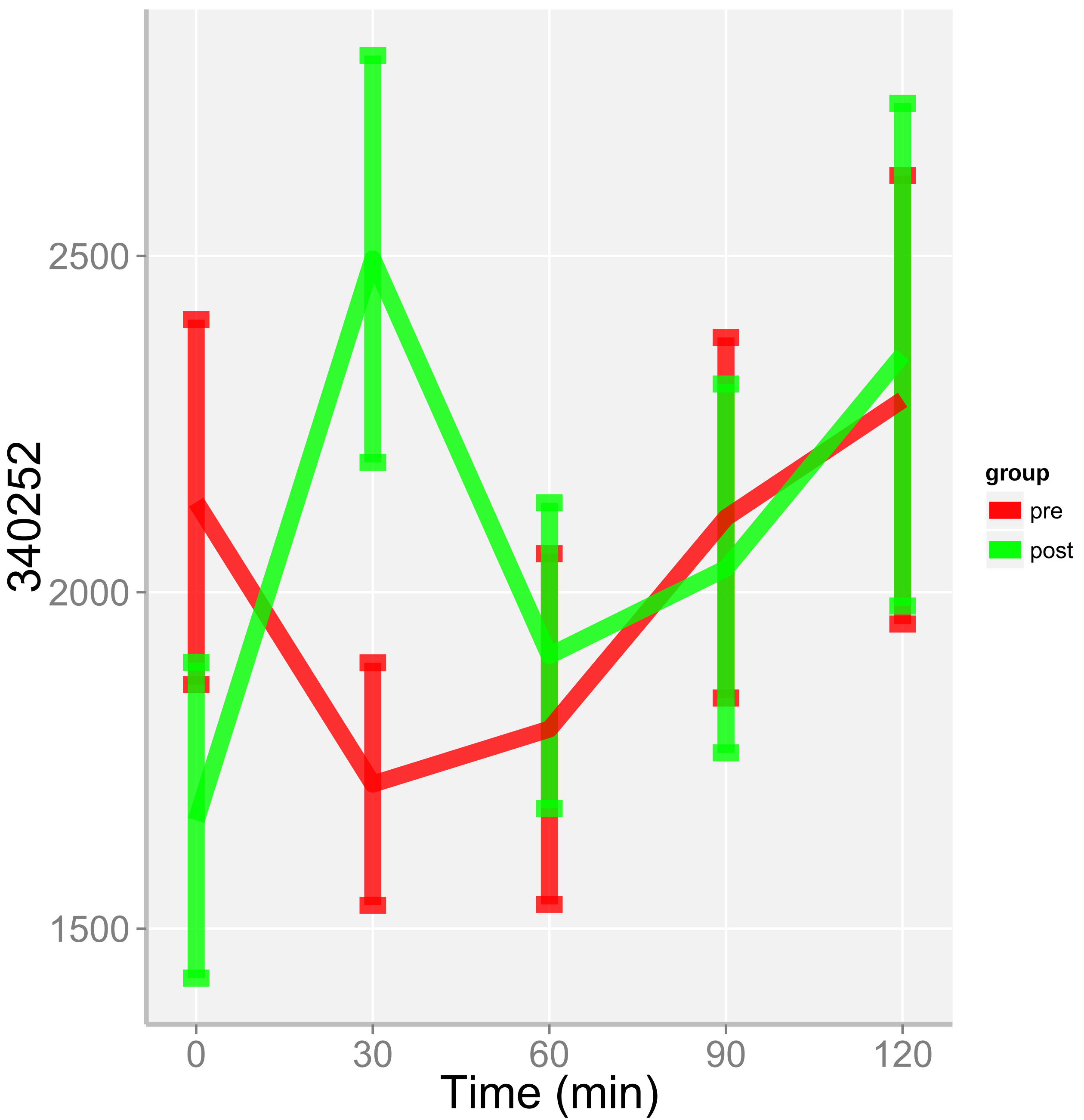

340252
2500
2000
1500
0
30
60
90
120
Time (min)
group
pre
post

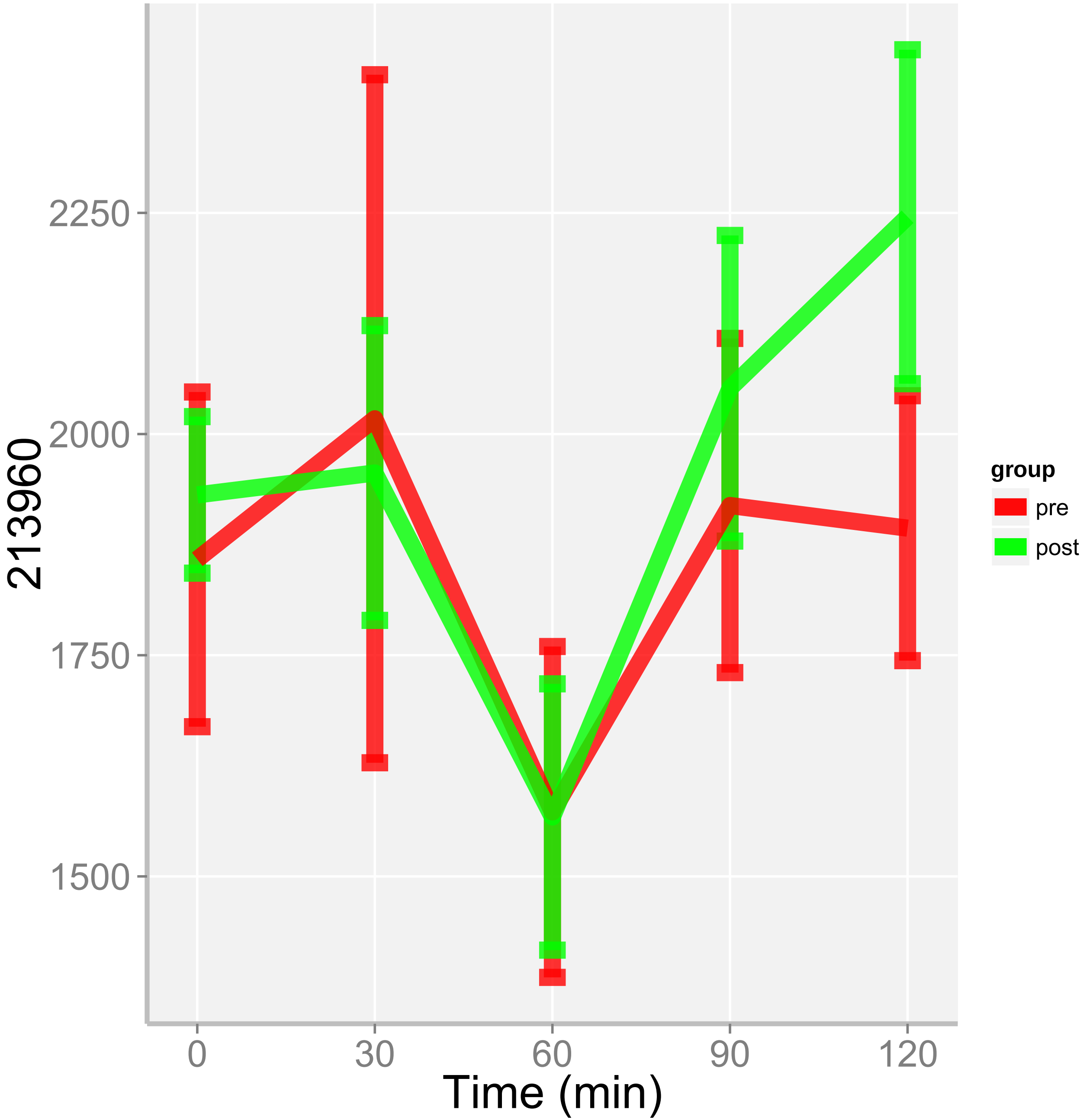

2250
2000
1750
1500
213960
0
30
60
90
120
Time (min)
group
pre
post

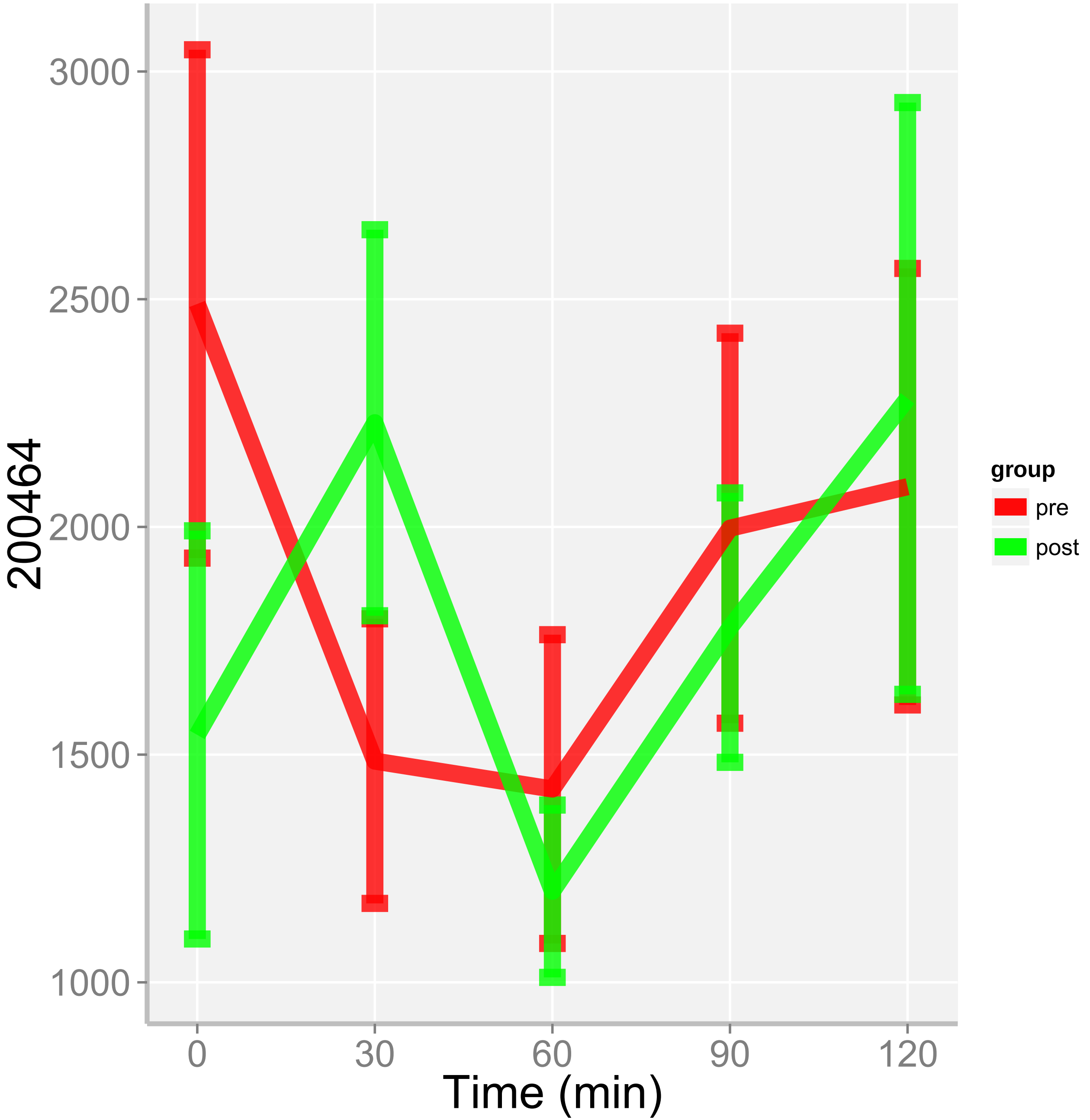

200464
3000
2500
2000
1500
1000
0
30
60
90
120
Time (min)
group
pre
post

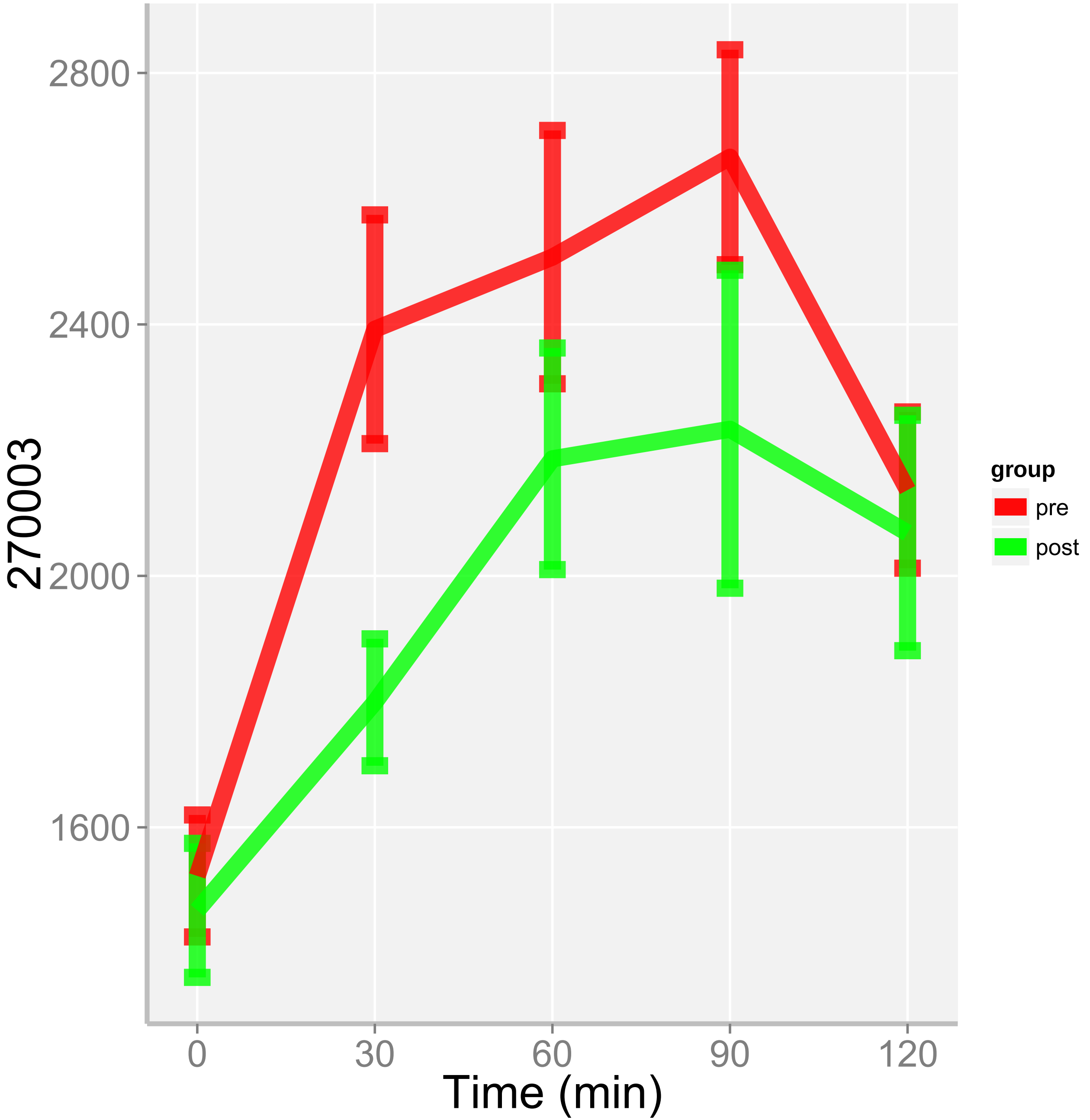

2800
2400
2000
1600
270003
0
30
60
90
120
Time (min)
group
pre
post

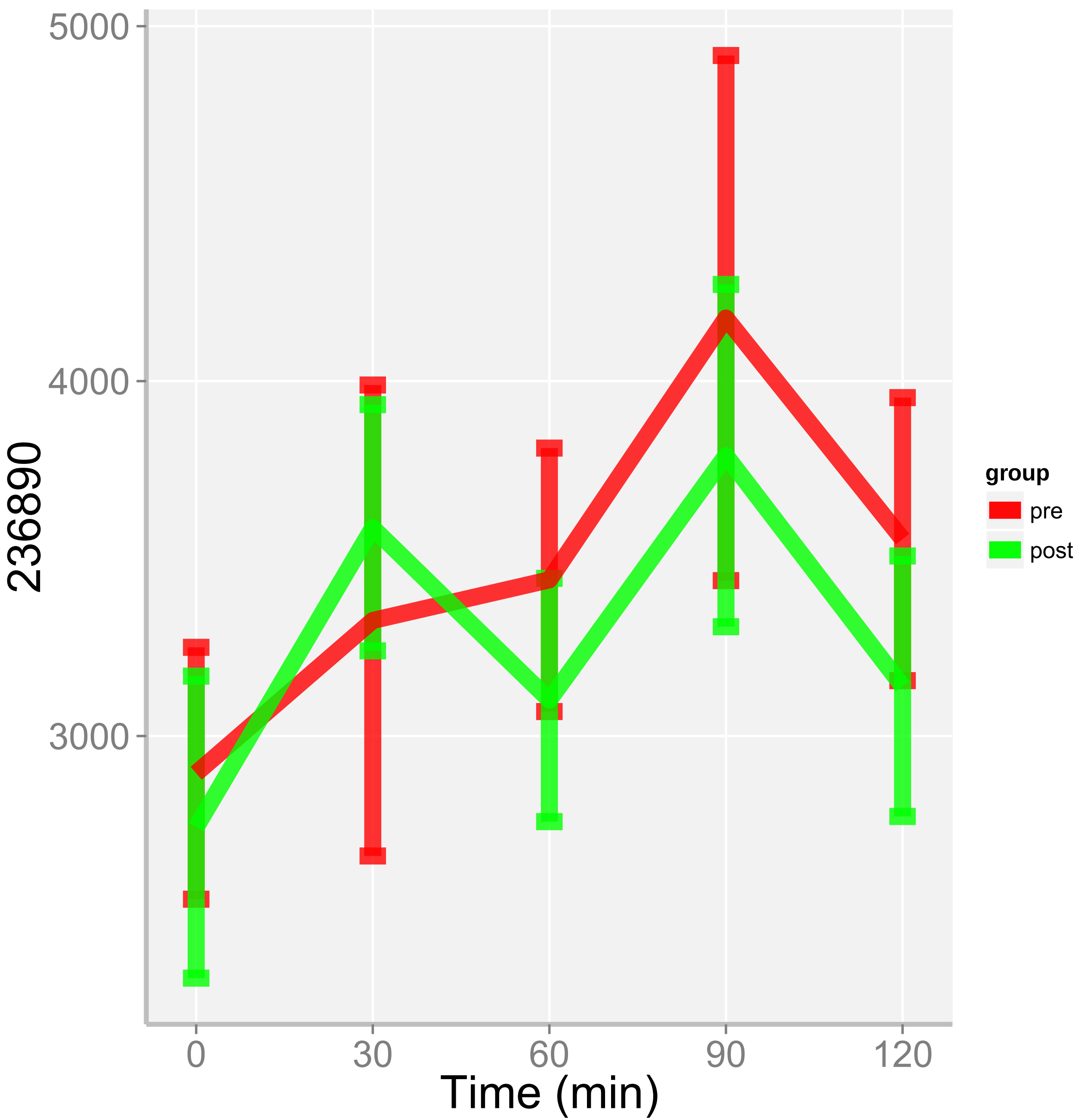
5000
4000
3000
236890
0
30
60
90
120
Time (min)
group
pre
post

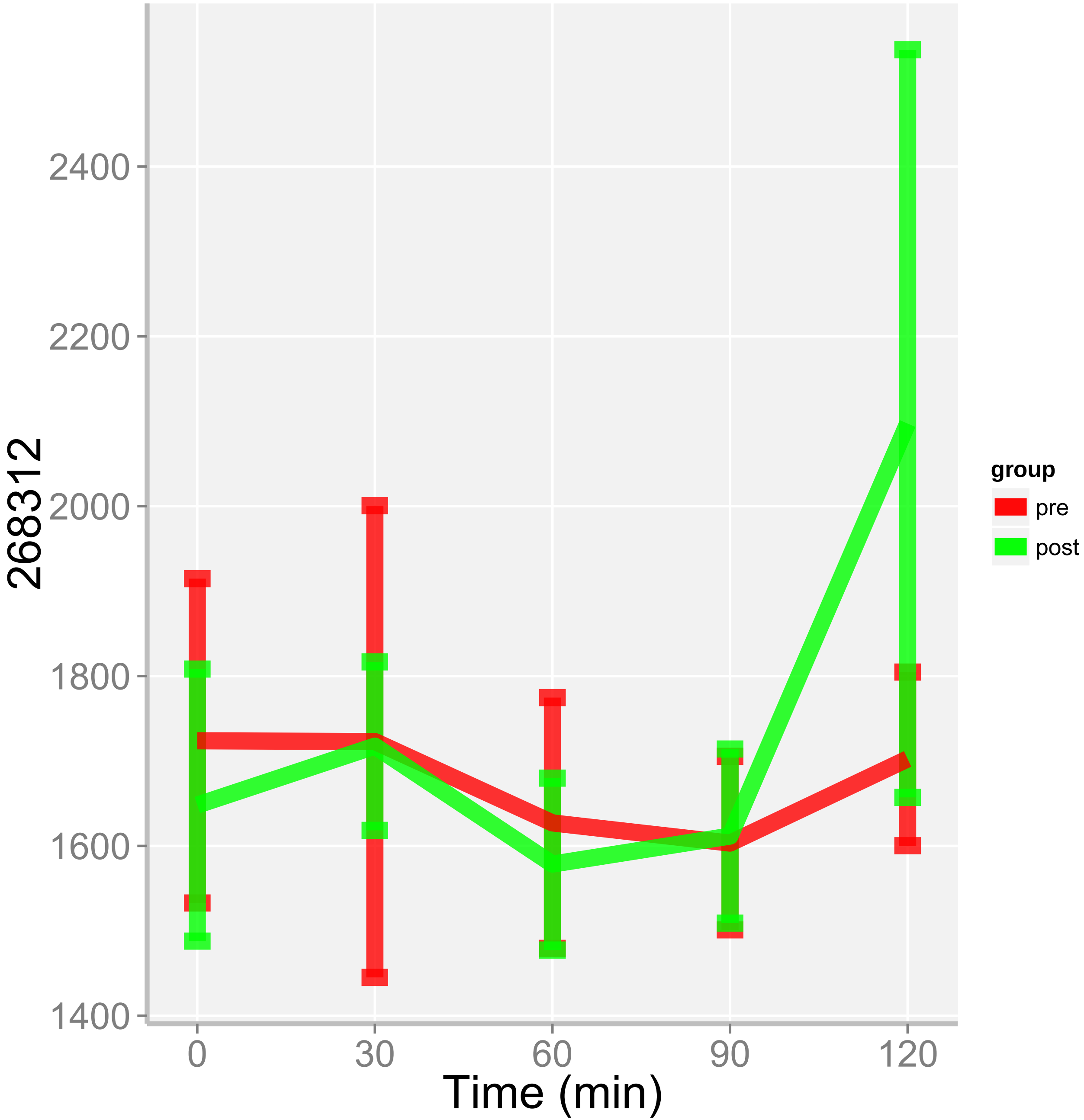

268312
2400
2200
2000
1800
1600
1400
0
30
60
90
120
Time (min)
group
pre
post

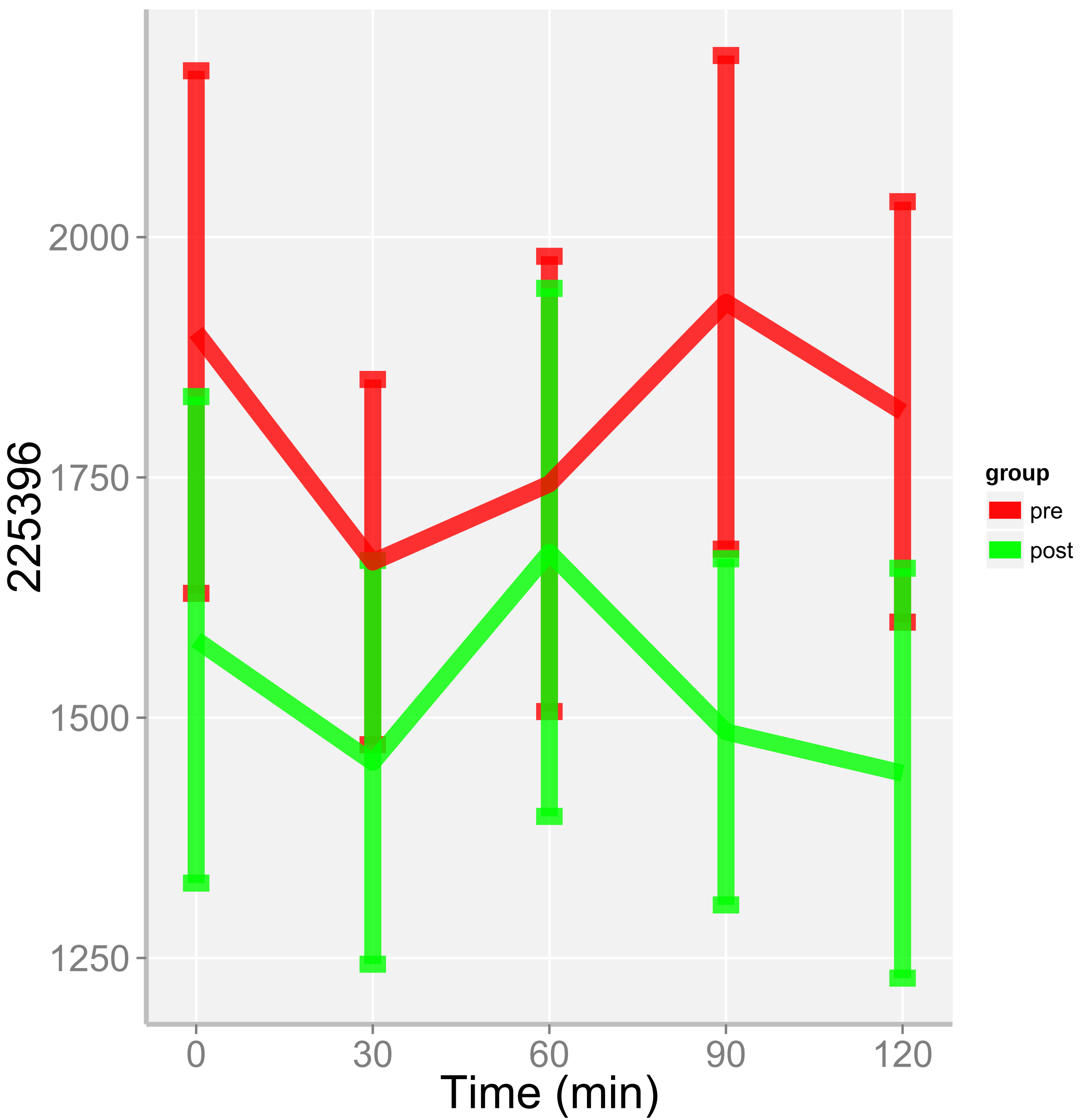

225396
2000
1750
1500
1250
0
30
60
90
120
Time (min)
group
pre
post

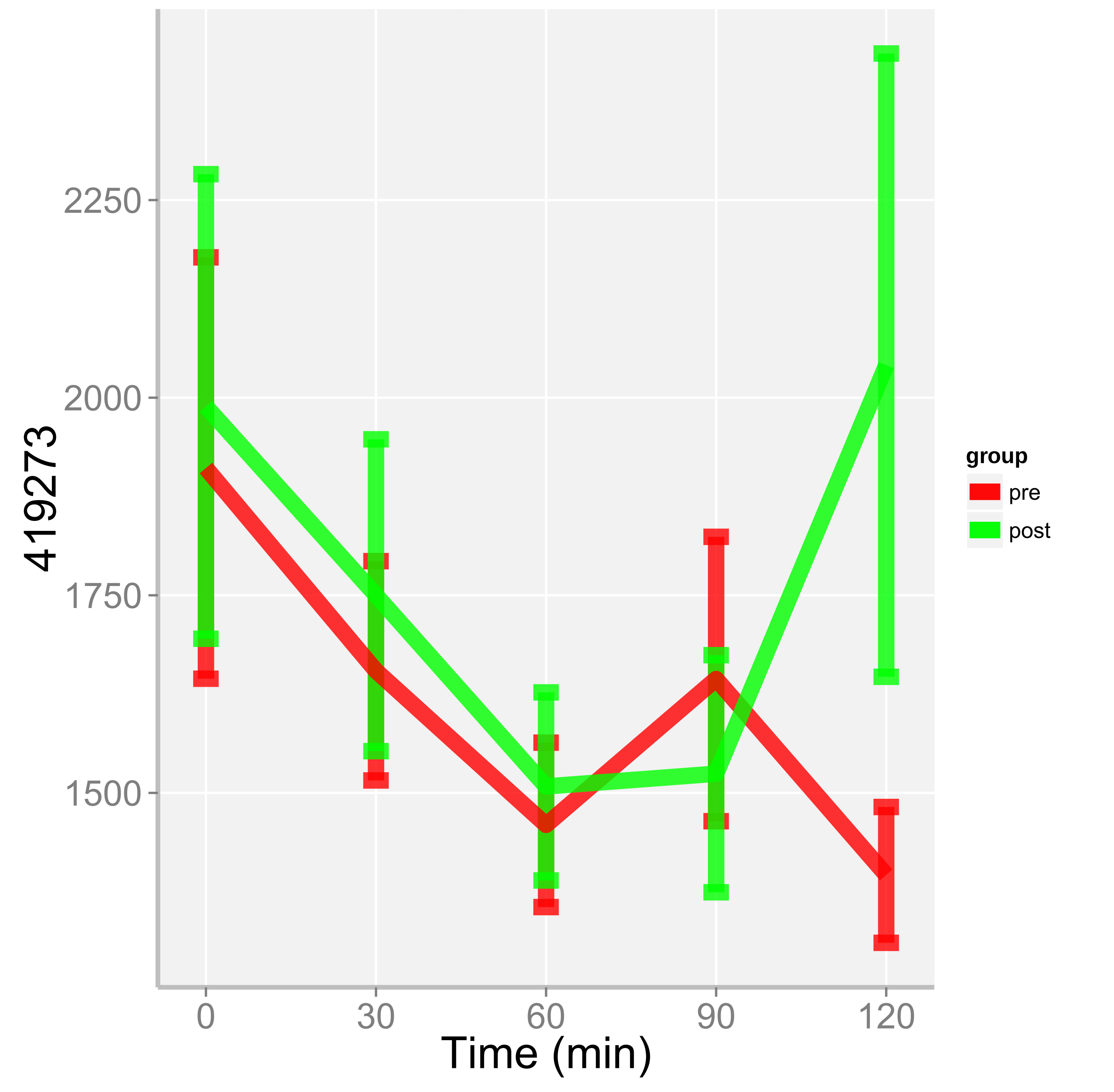
2250
2000
1750
1500
419273
0
30
60
90
120
Time (min)
group
pre
post

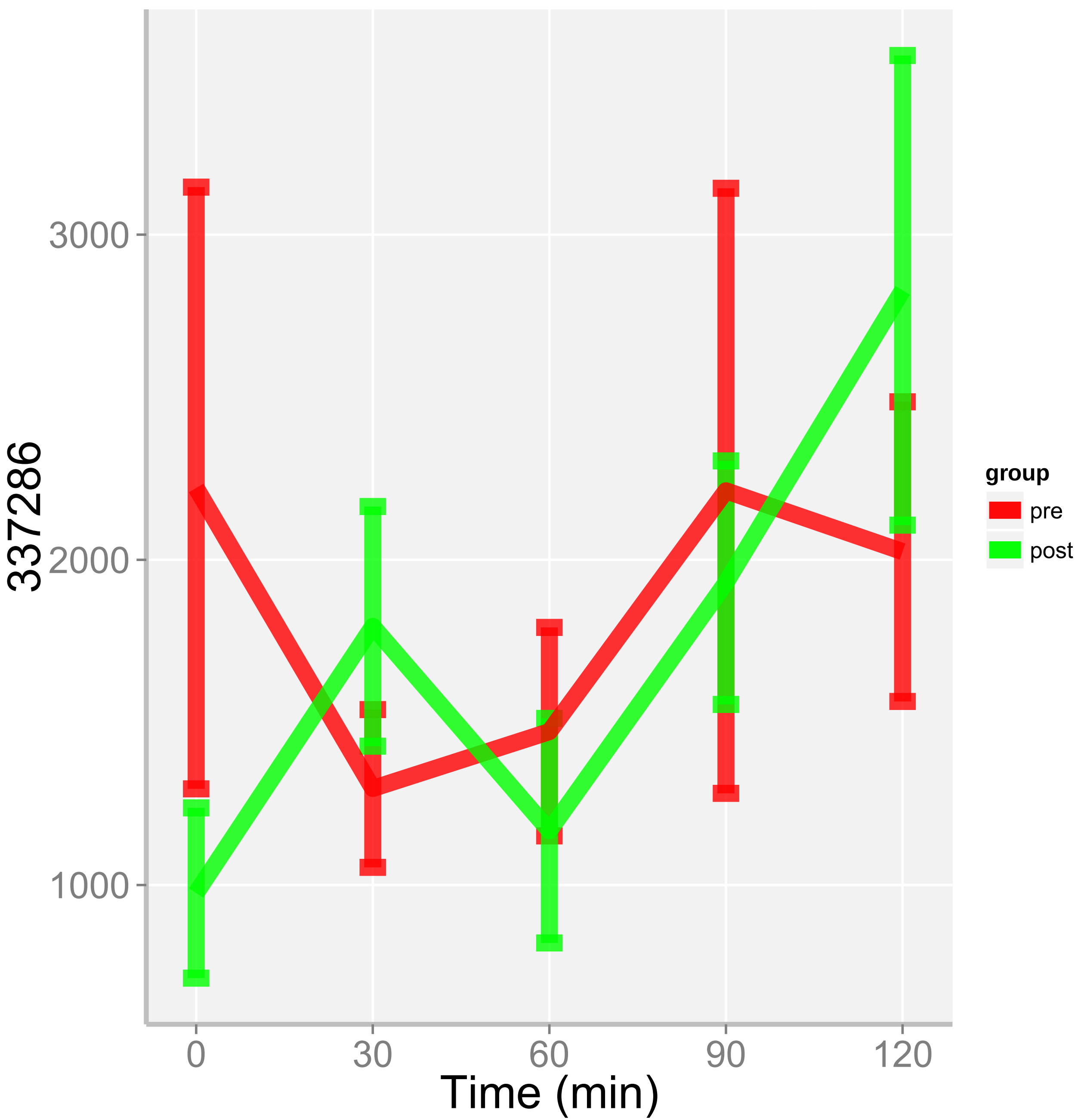

337286
3000
2000
1000
0
30
60
90
120
Time (min)
group
pre
post

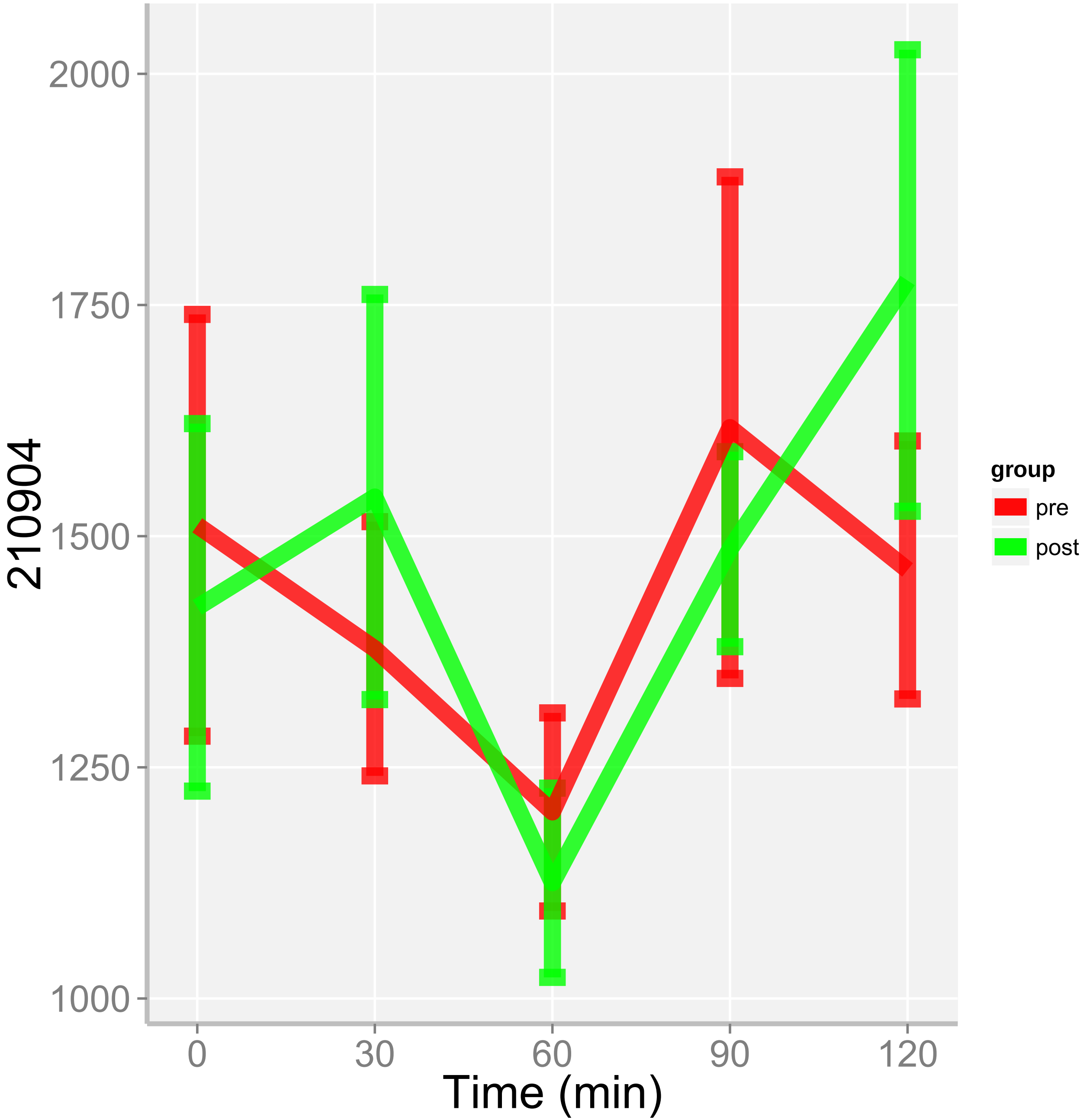

2000
1750
1500
1250
1000
210904
0
30
60
90
120
Time (min)
group
pre
post

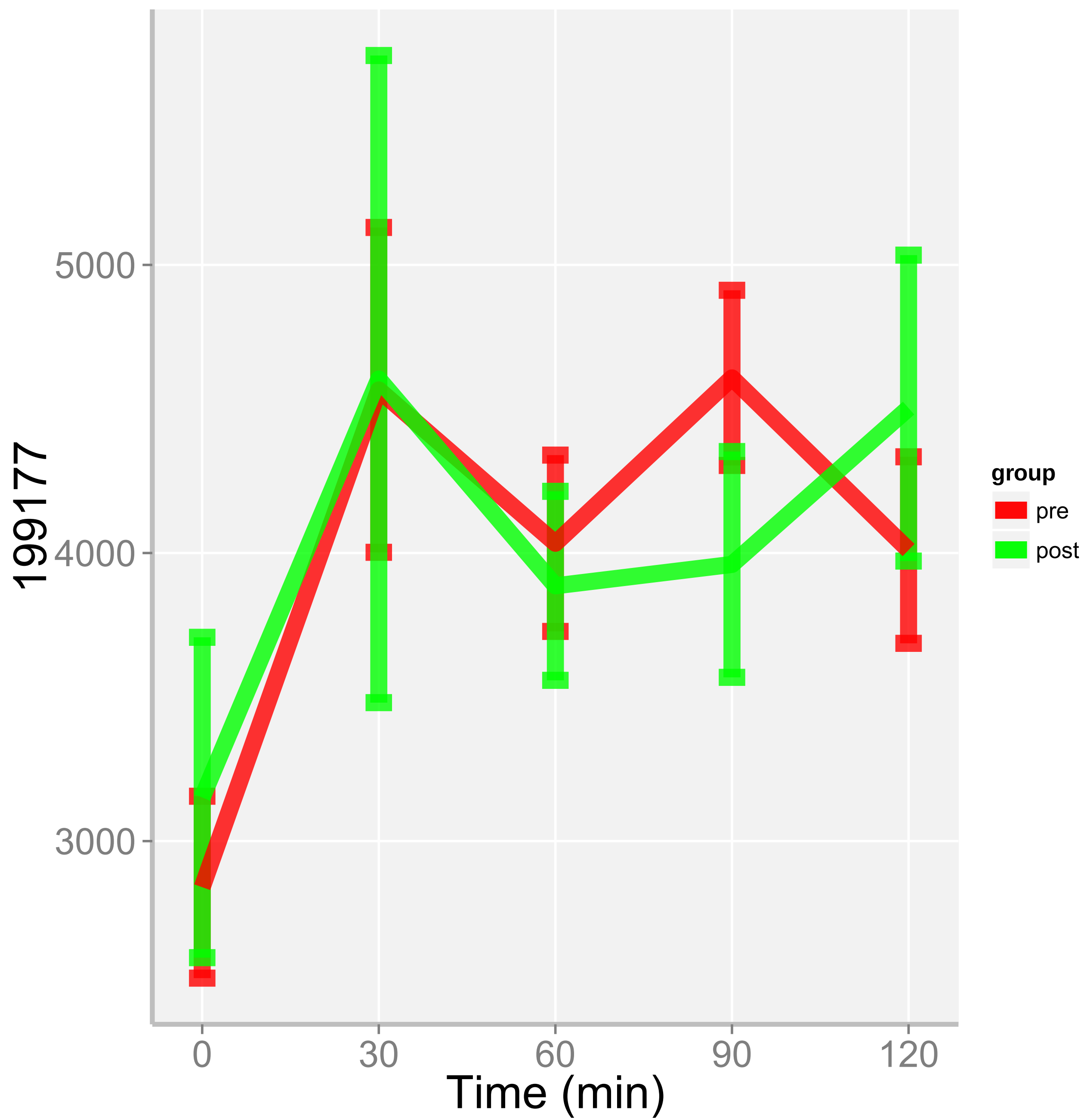

199177
5000
4000
3000
0
30
60
90
120
Time (min)
group
pre
post

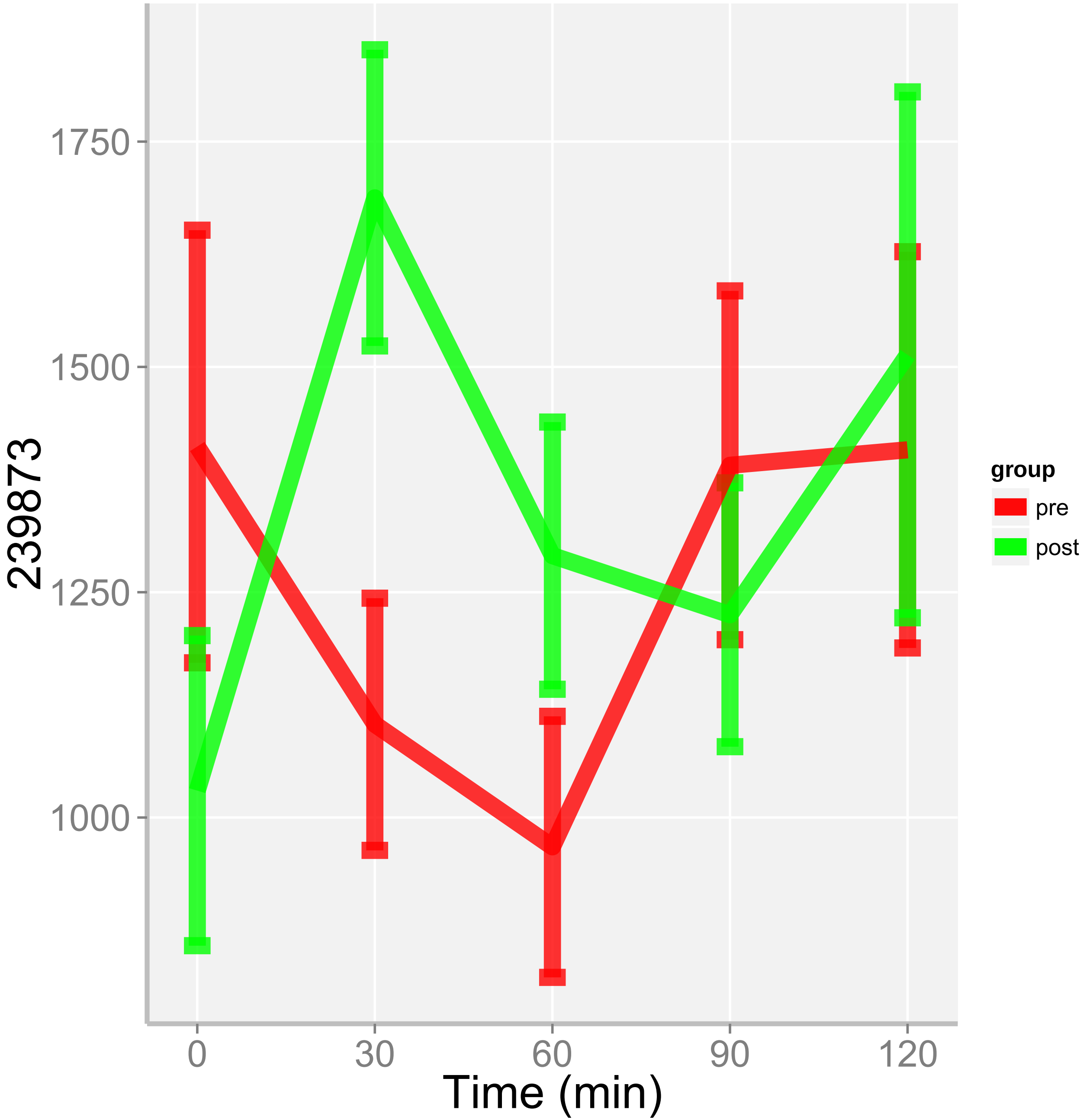

239873
1750
1500
1250
1000
0
30
60
90
120
Time (min)
group
pre
post

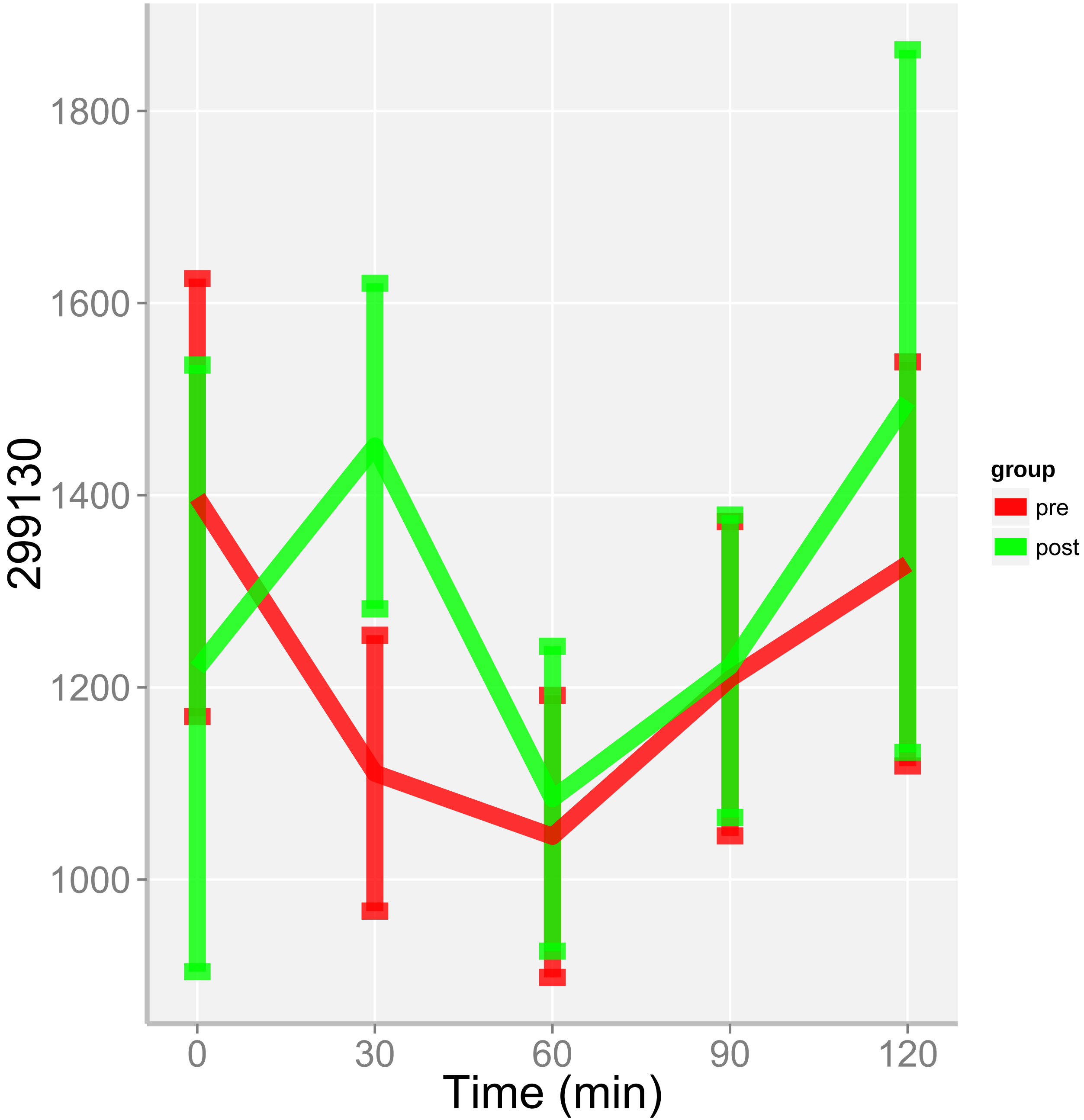

299130
1800
1600
1400
1200
1000
0
30
60
90
120
Time (min)
group
pre
post

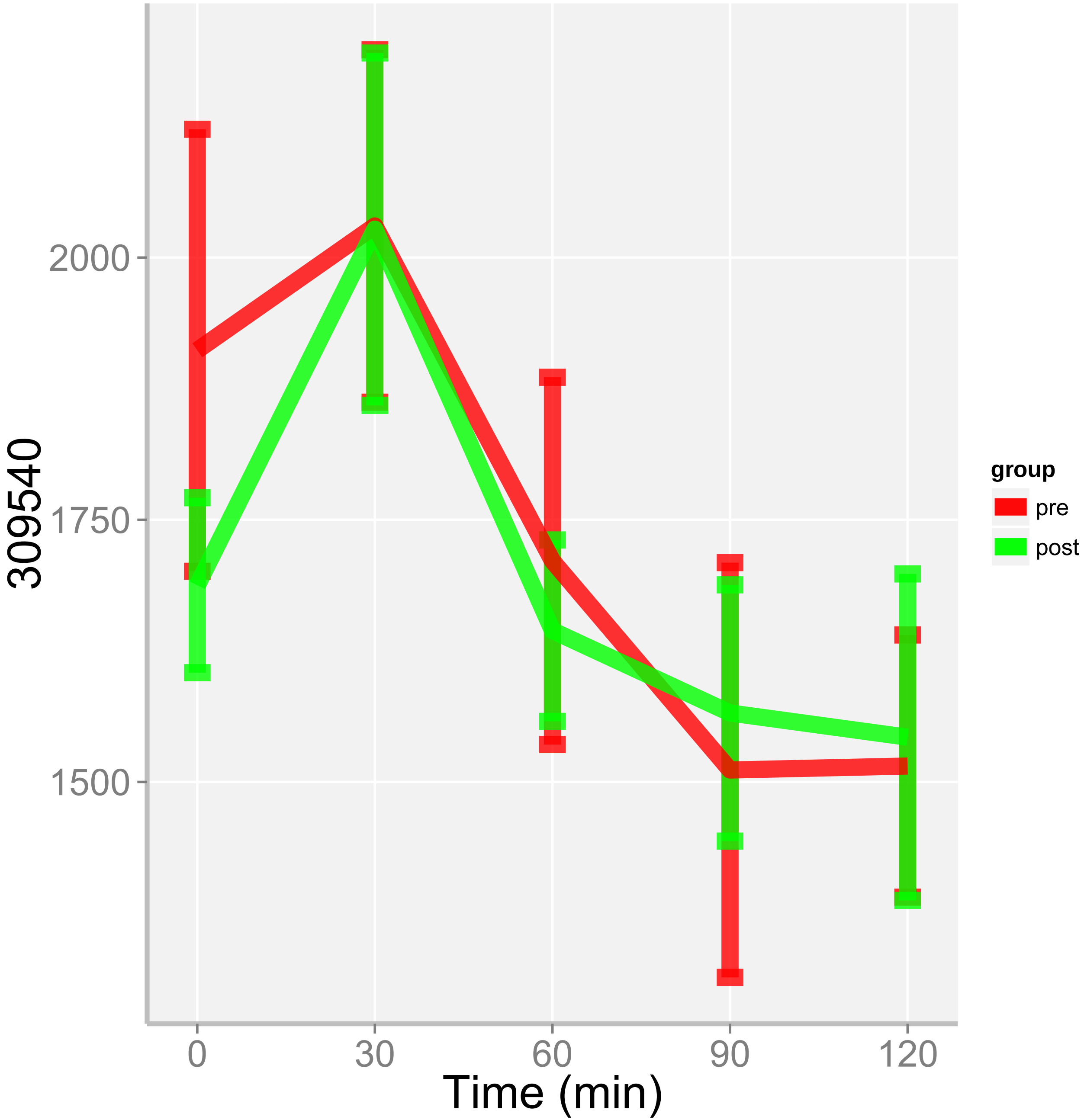

2000
1750
1500
309540
0
30
60
90
120
Time (min)
group
pre
post

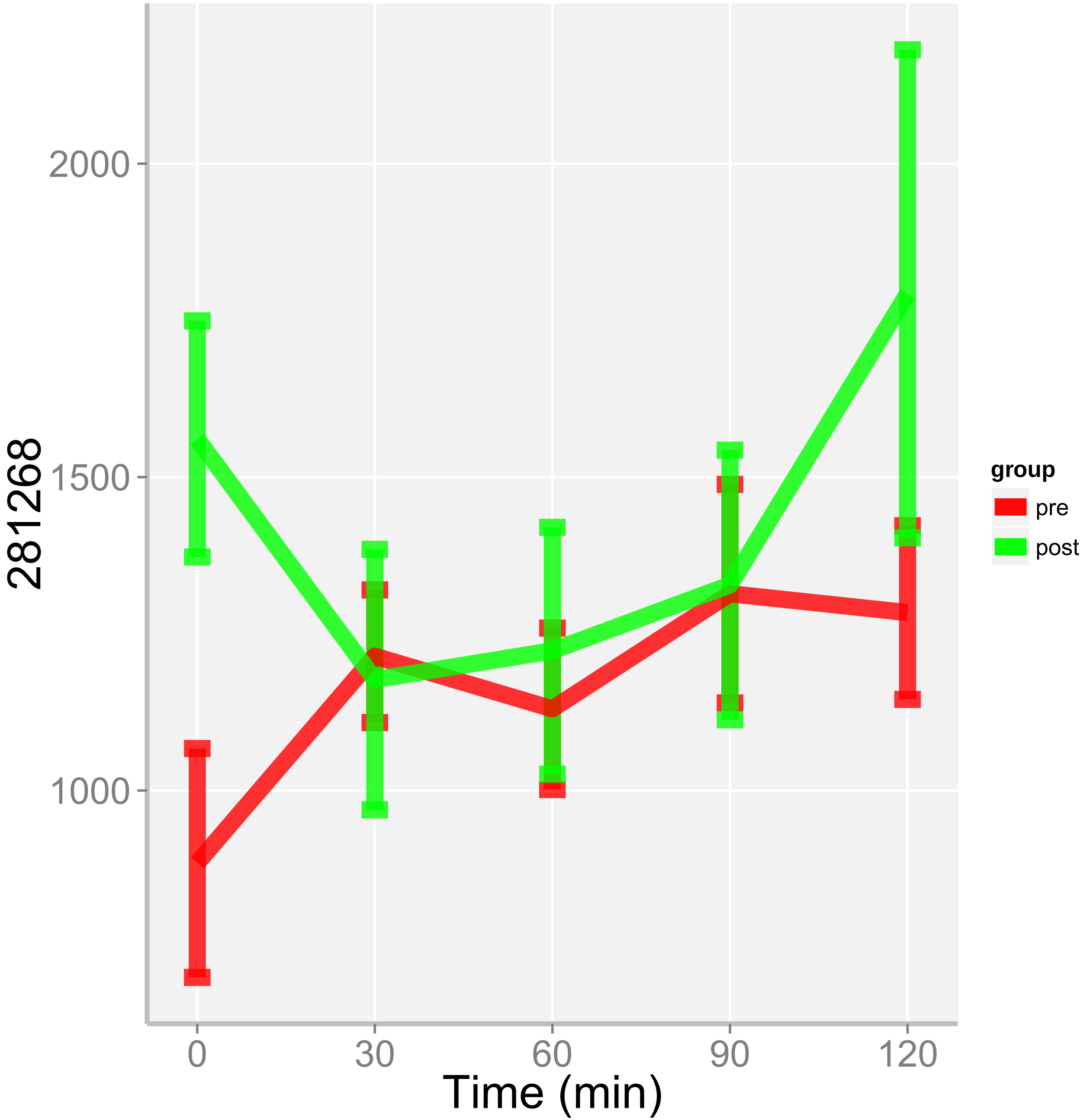

2000
1500
1000
281268
0
30
60
90
120
Time (min)
group
pre
post

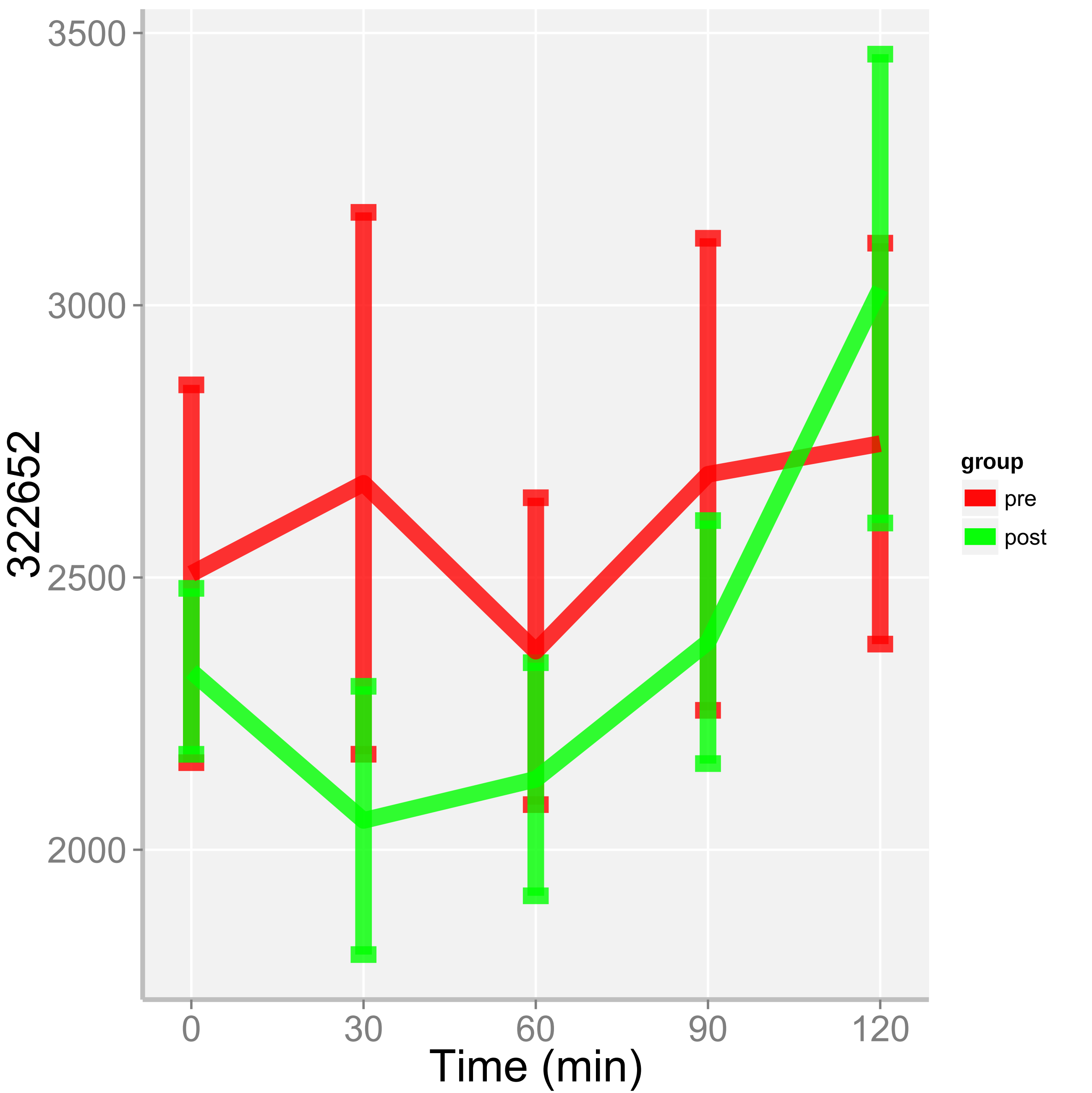

3500
3000
2500
2000
322652
0
30
60
90
120
Time (min)
group
pre
post

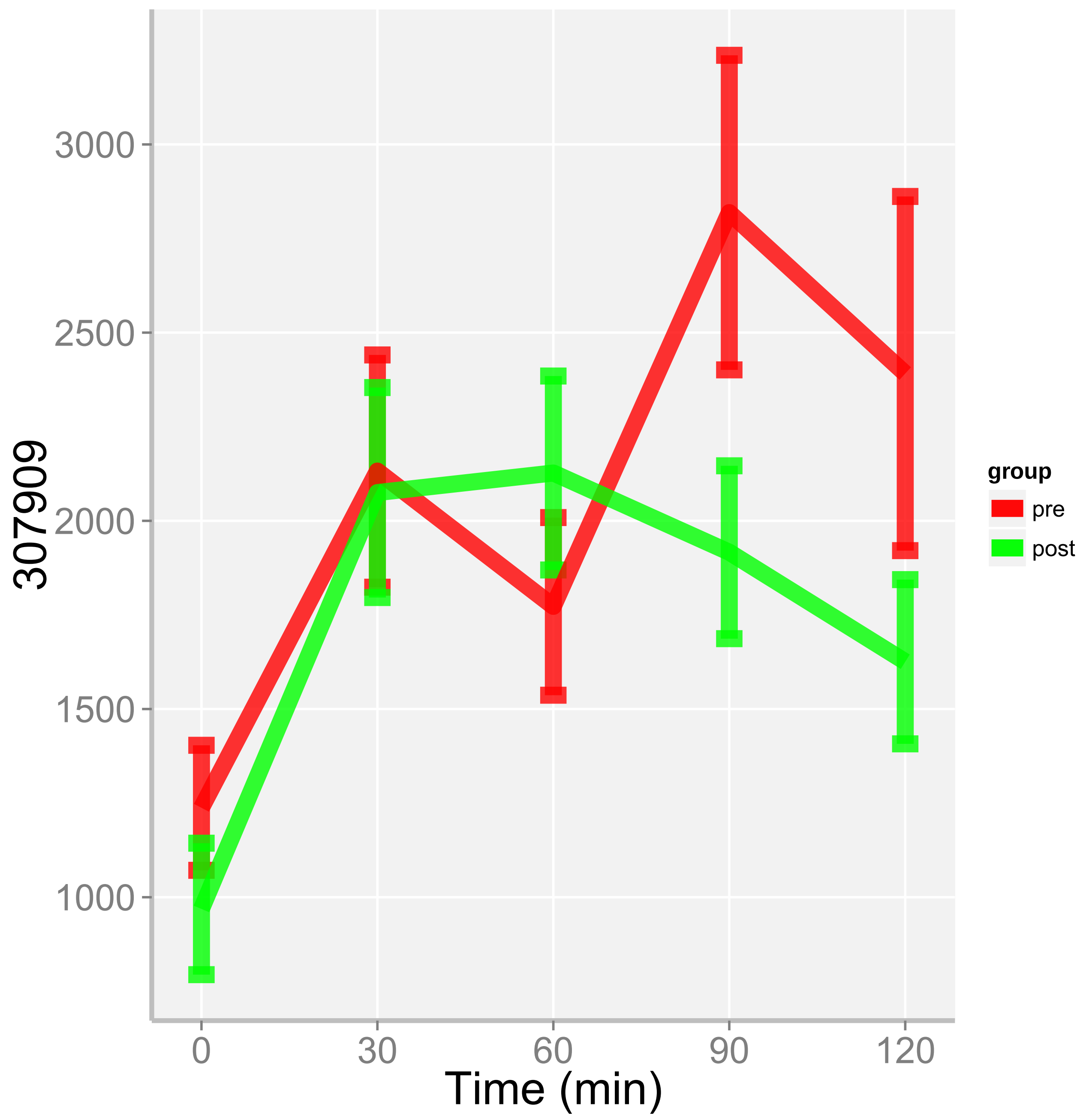

3000
2500
2000
1500
1000
307909
0
30
60
90
120
Time (min)
group
pre
post

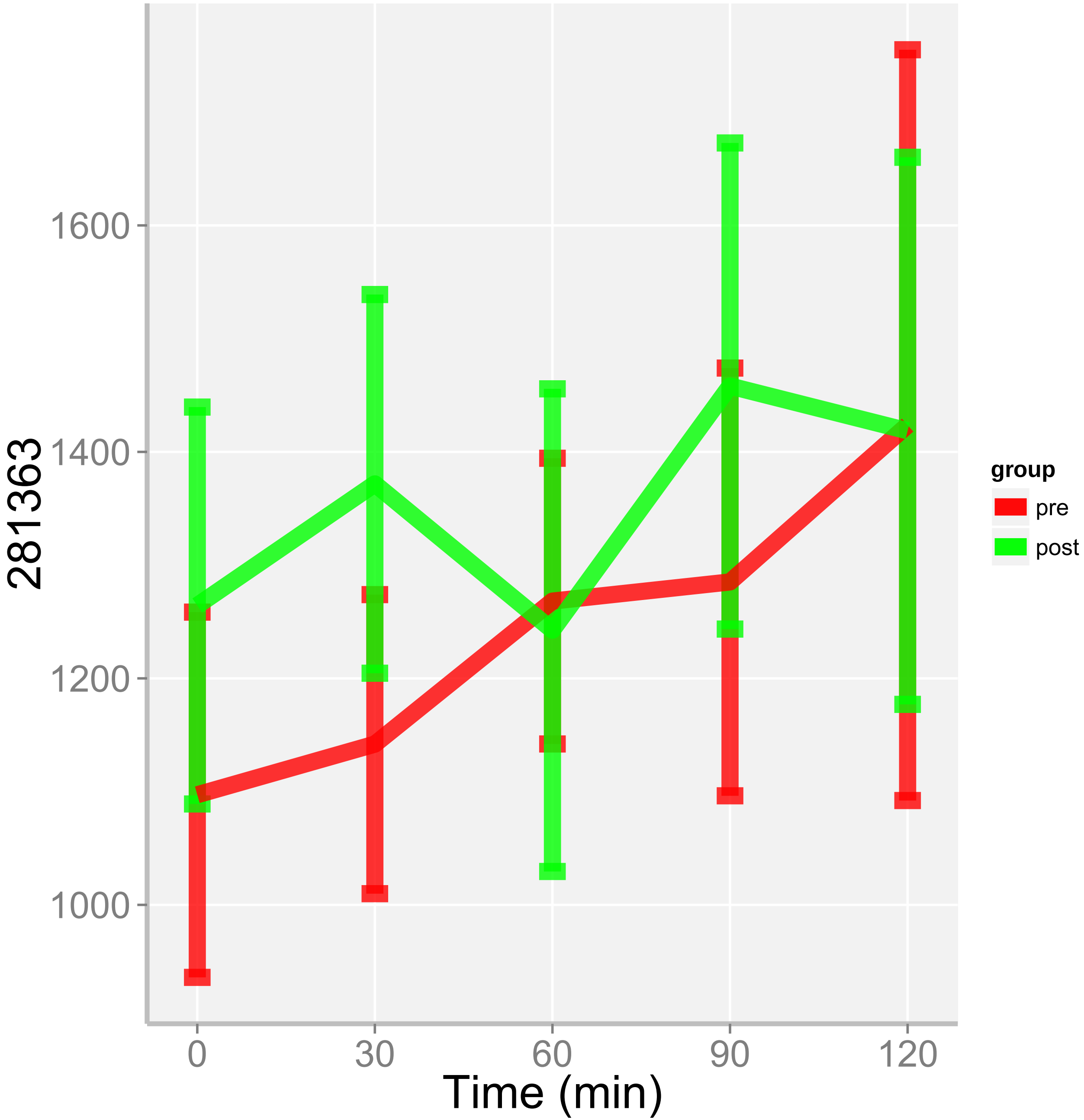

281363
1000
1200
1400
1600
0
30
60
90
120
Time (min)
group
pre
post

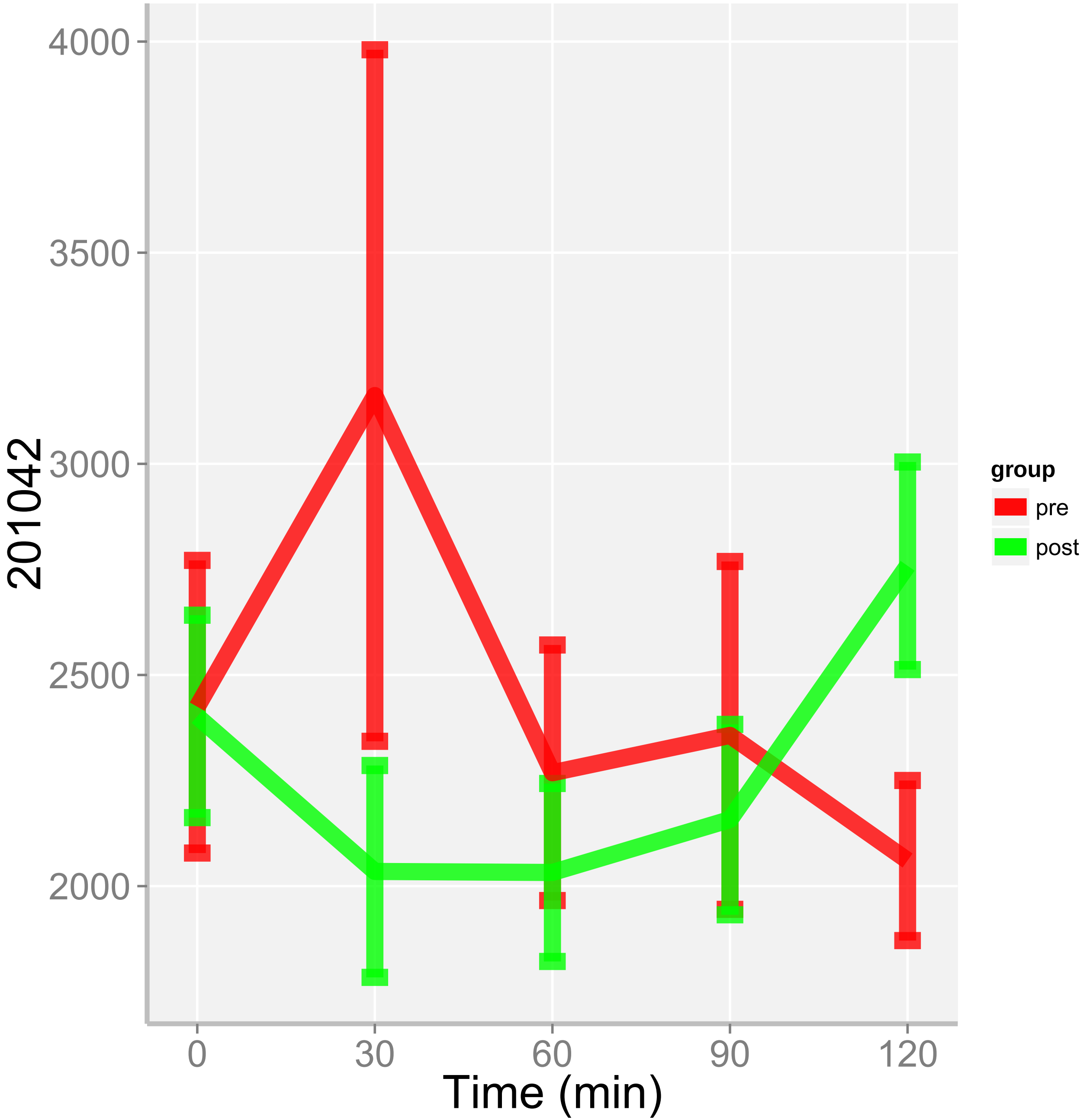

4000
3500
3000
2500
2000
201042
0
30
60
90
120
Time (min)
group
pre
post

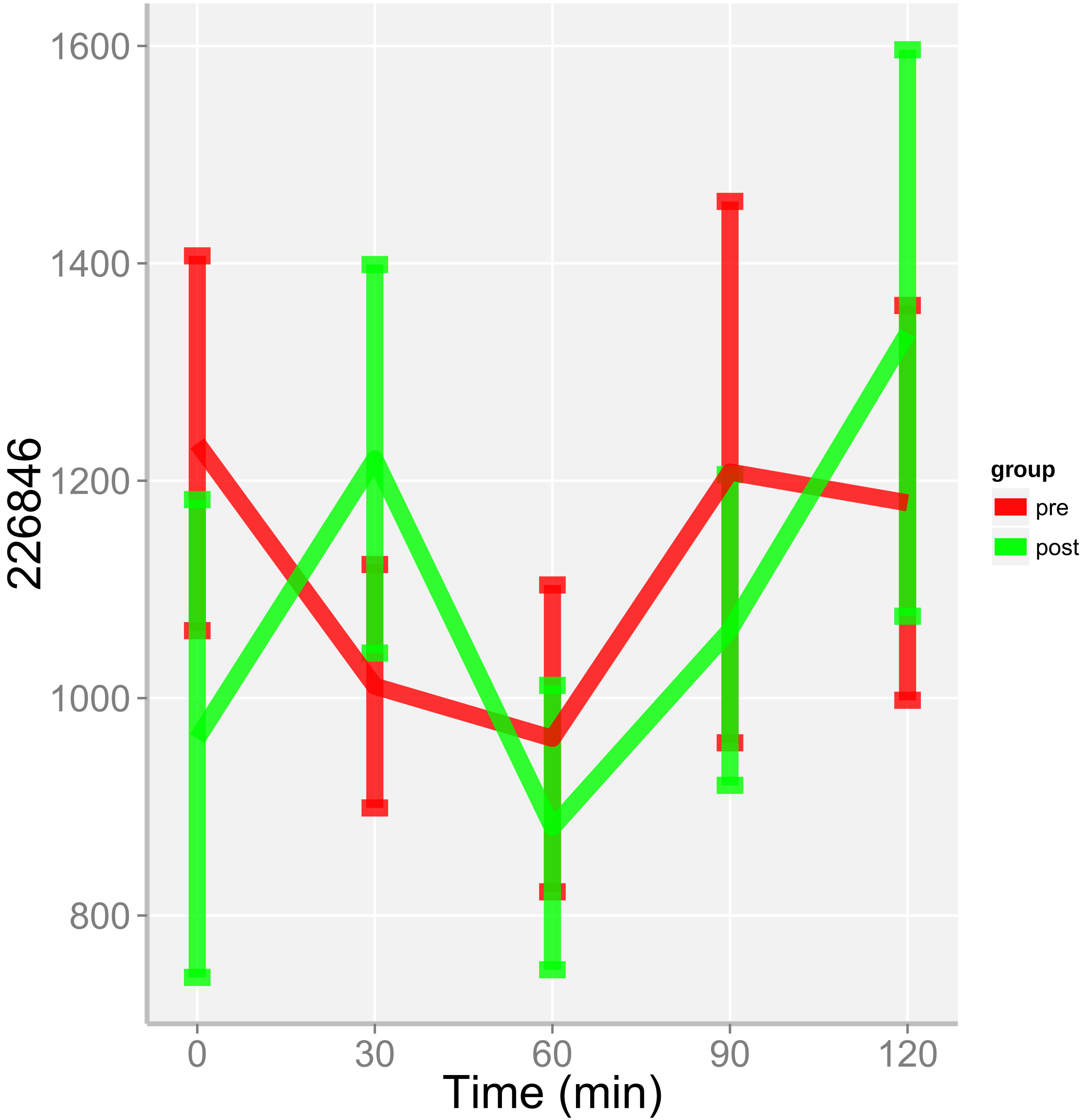

226846
1600
1400
1200
1000
800
0
30
60
90
120
Time (min)
group
pre
post

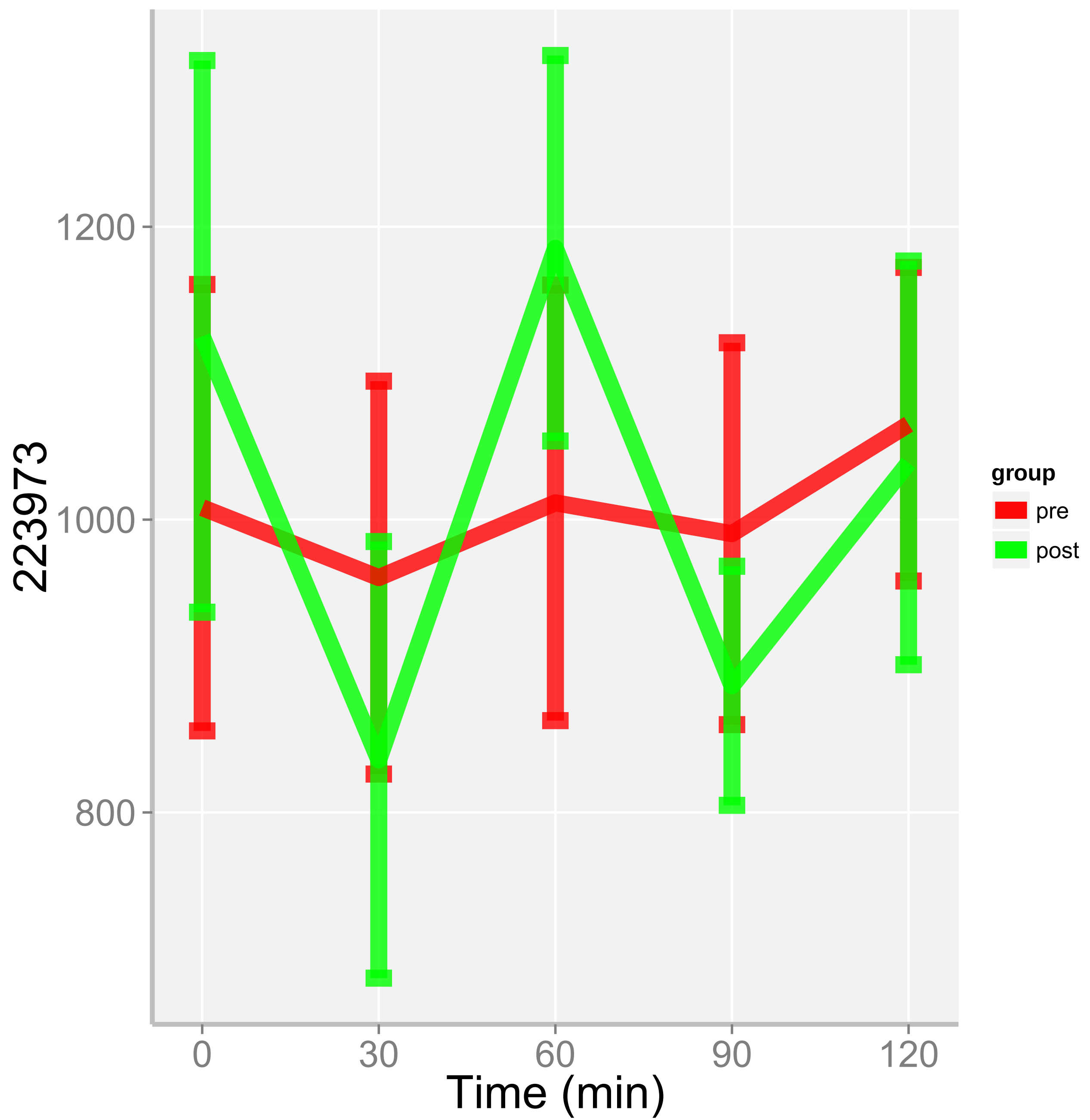

223973
1200
1000
800
0
30
60
90
120
Time (min)
group
pre
post

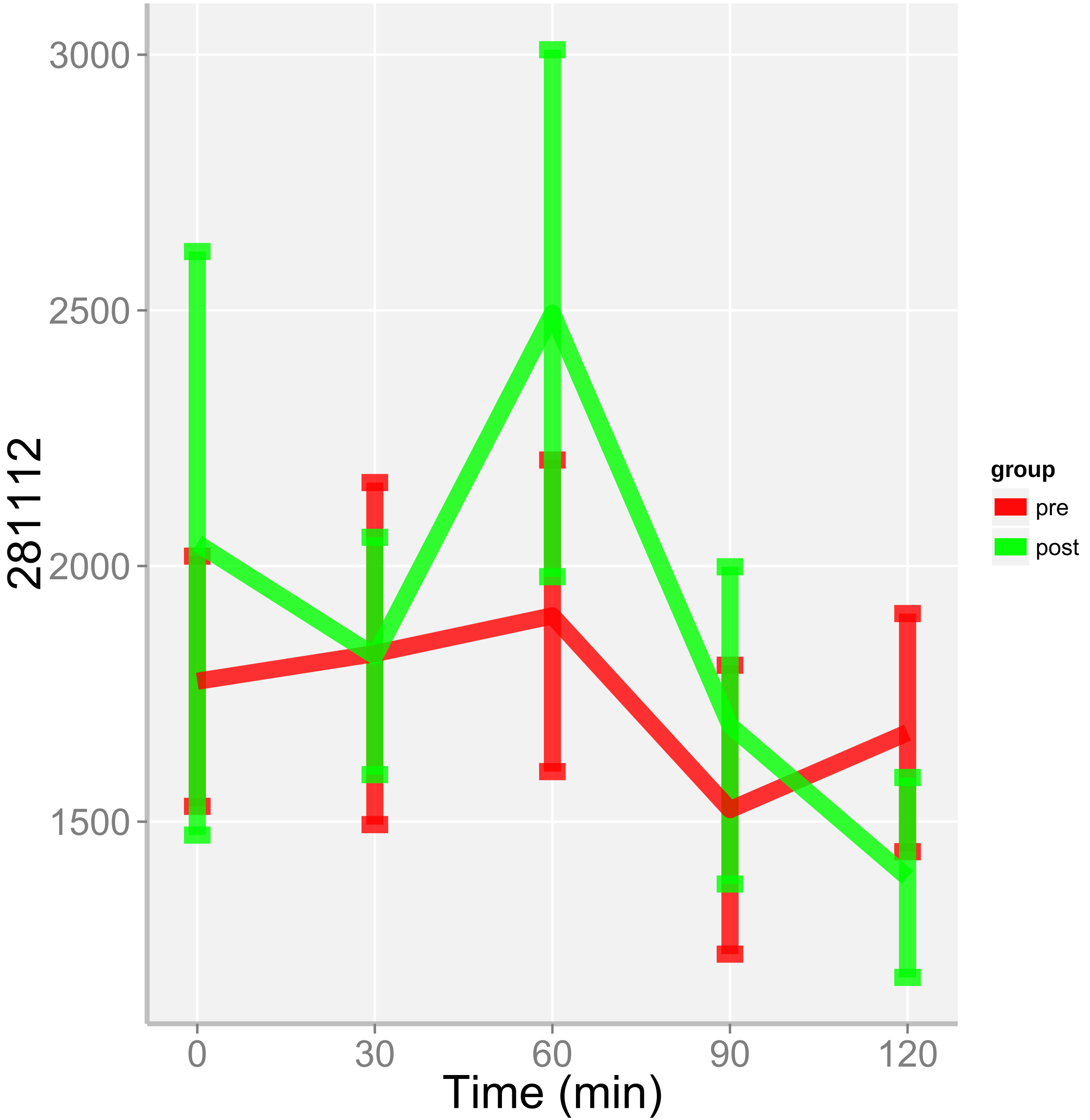

281112
3000
2500
2000
1500
0
30
60
90
120
Time (min)
group
pre
post

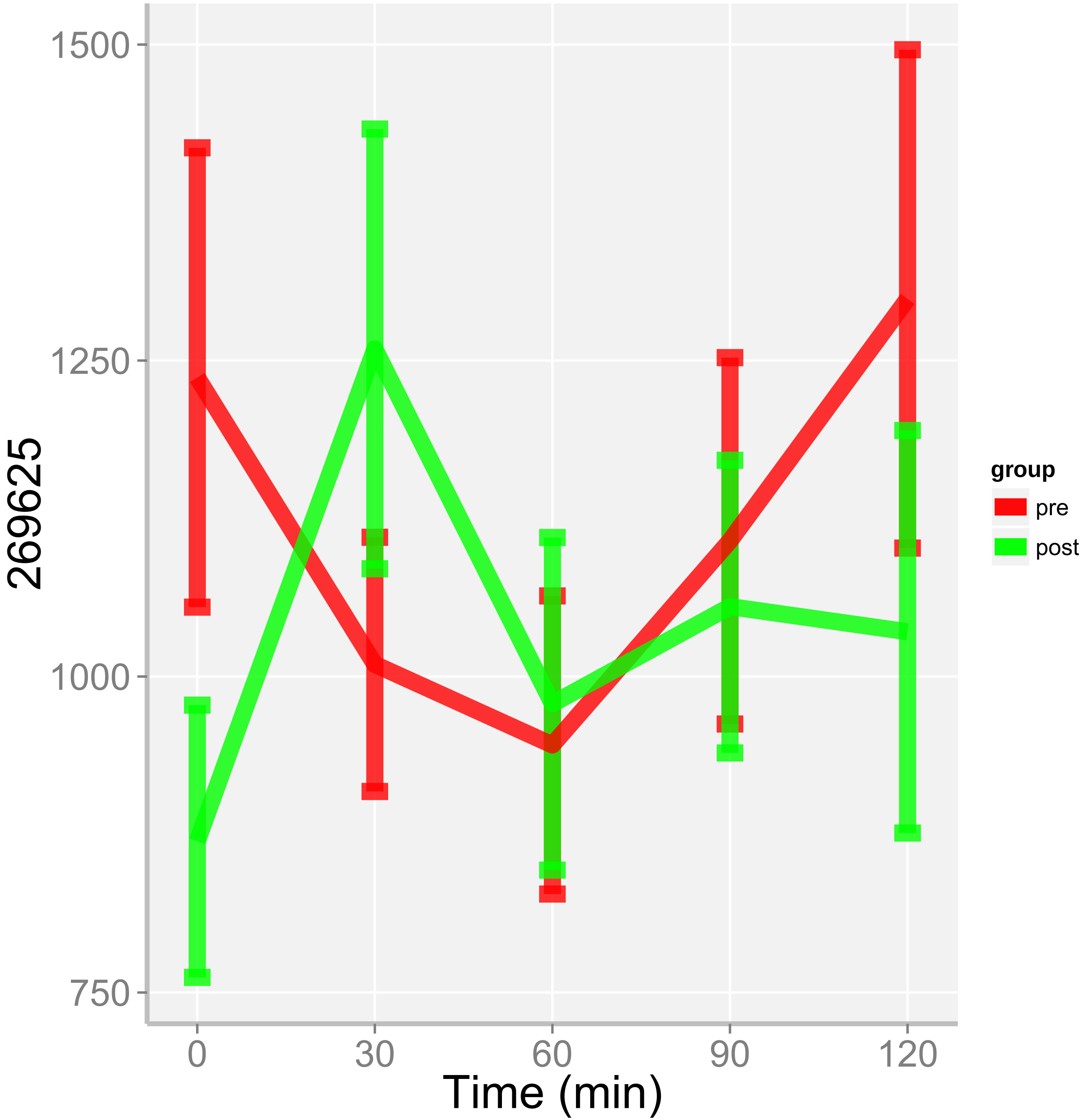

1500
1250
1000
750
269625
0
30
60
90
120
Time (min)
group
pre
post

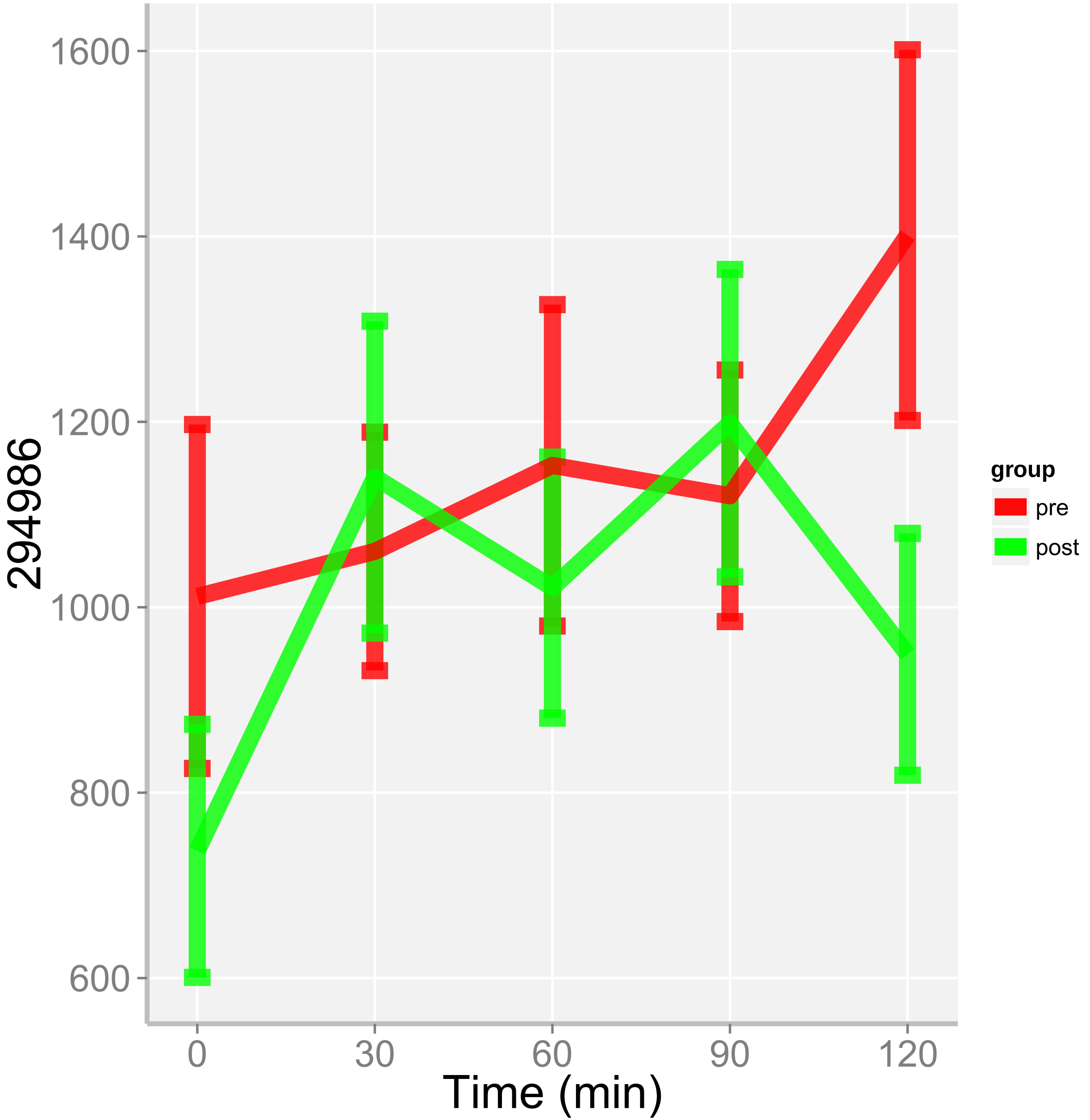
294986
1600
1400
1200
1000
800
600
0
30
60
90
120
Time (min)
group
pre
post

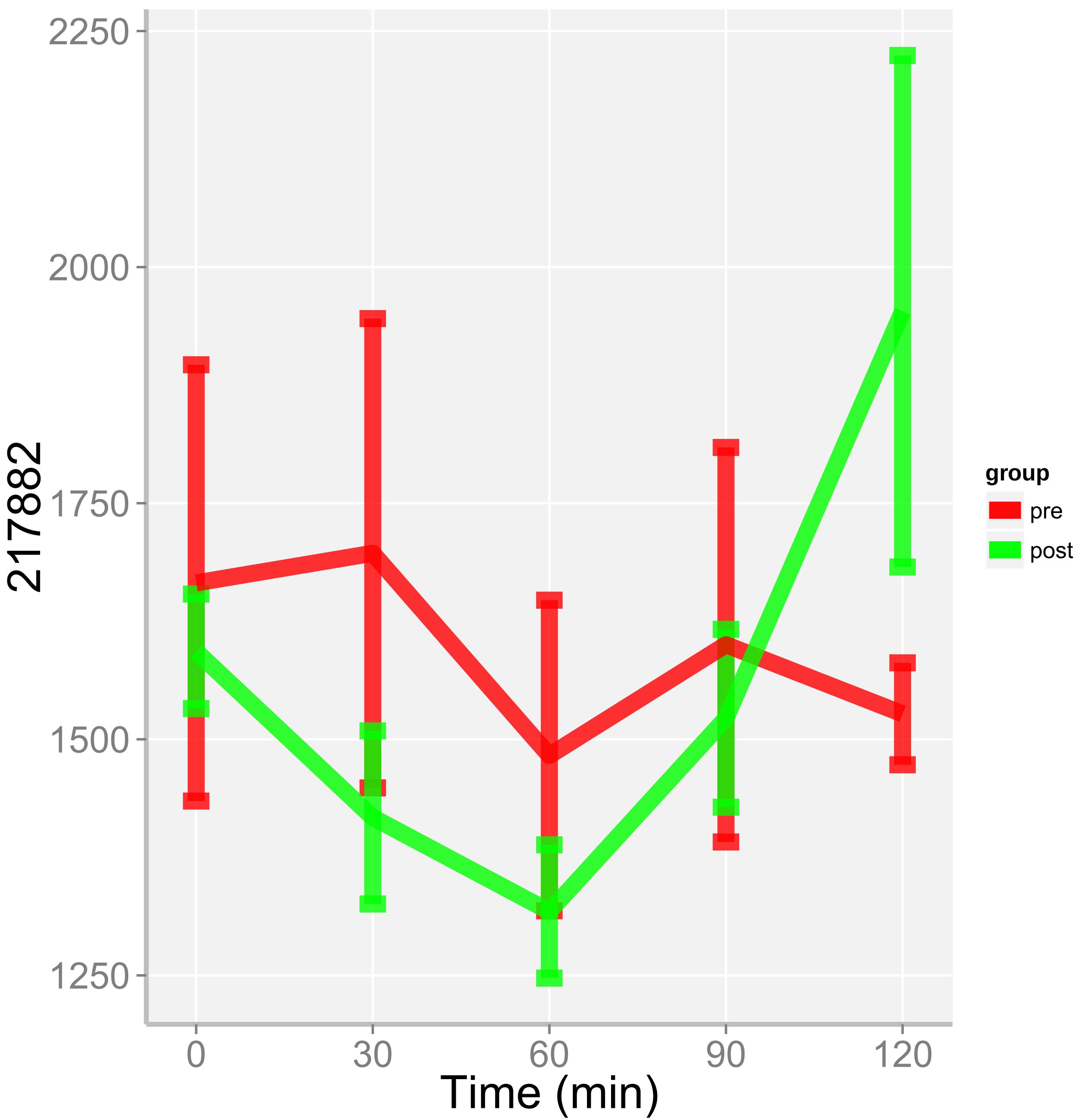

2250
2000
1750
1500
1250
217882
0
30
60
90
120
Time (min)
group
pre
post

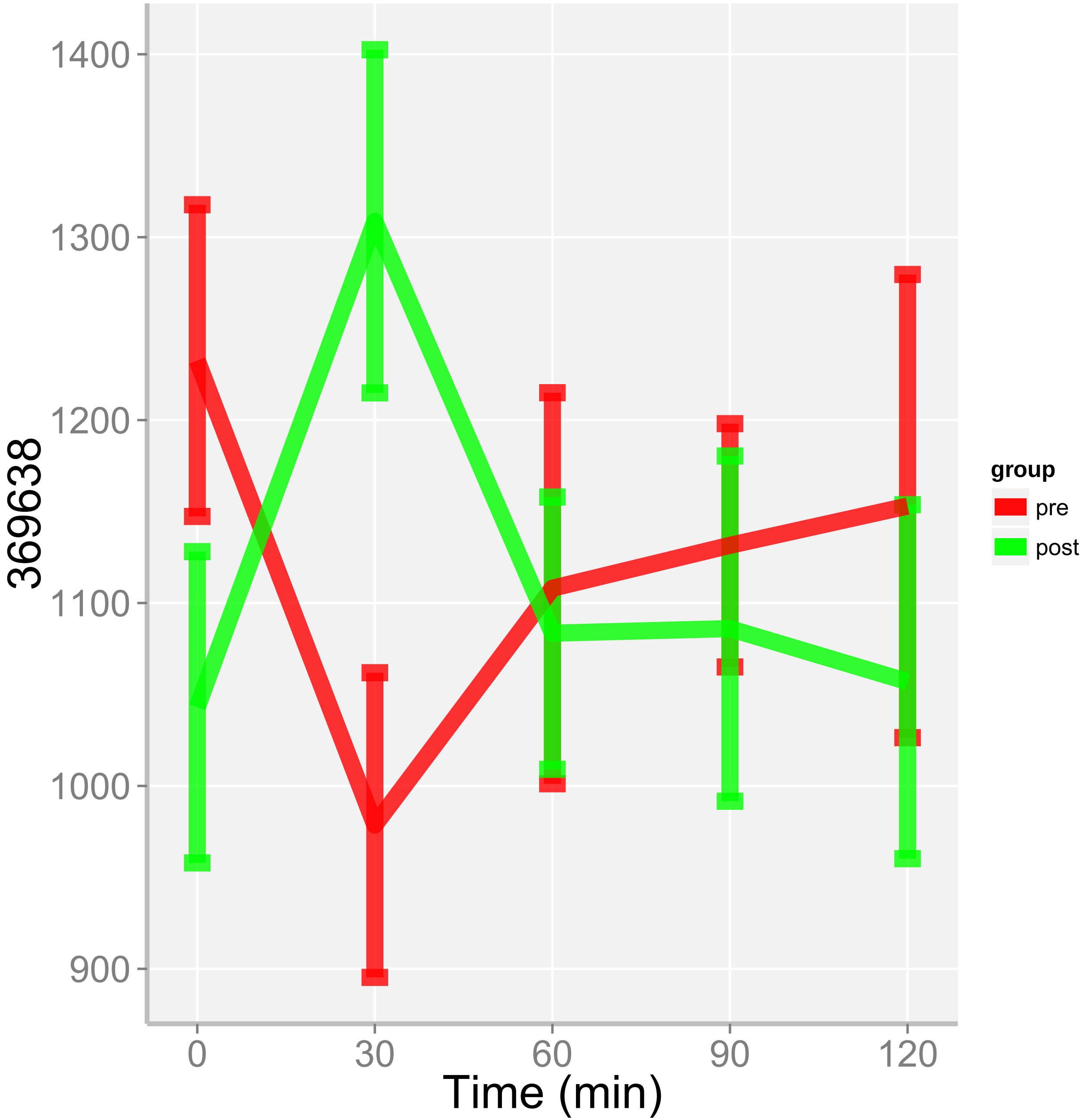

3696638
1400
1300
1200
1100
1000
900
0
30
60
90
120
Time (min)
group
pre
post

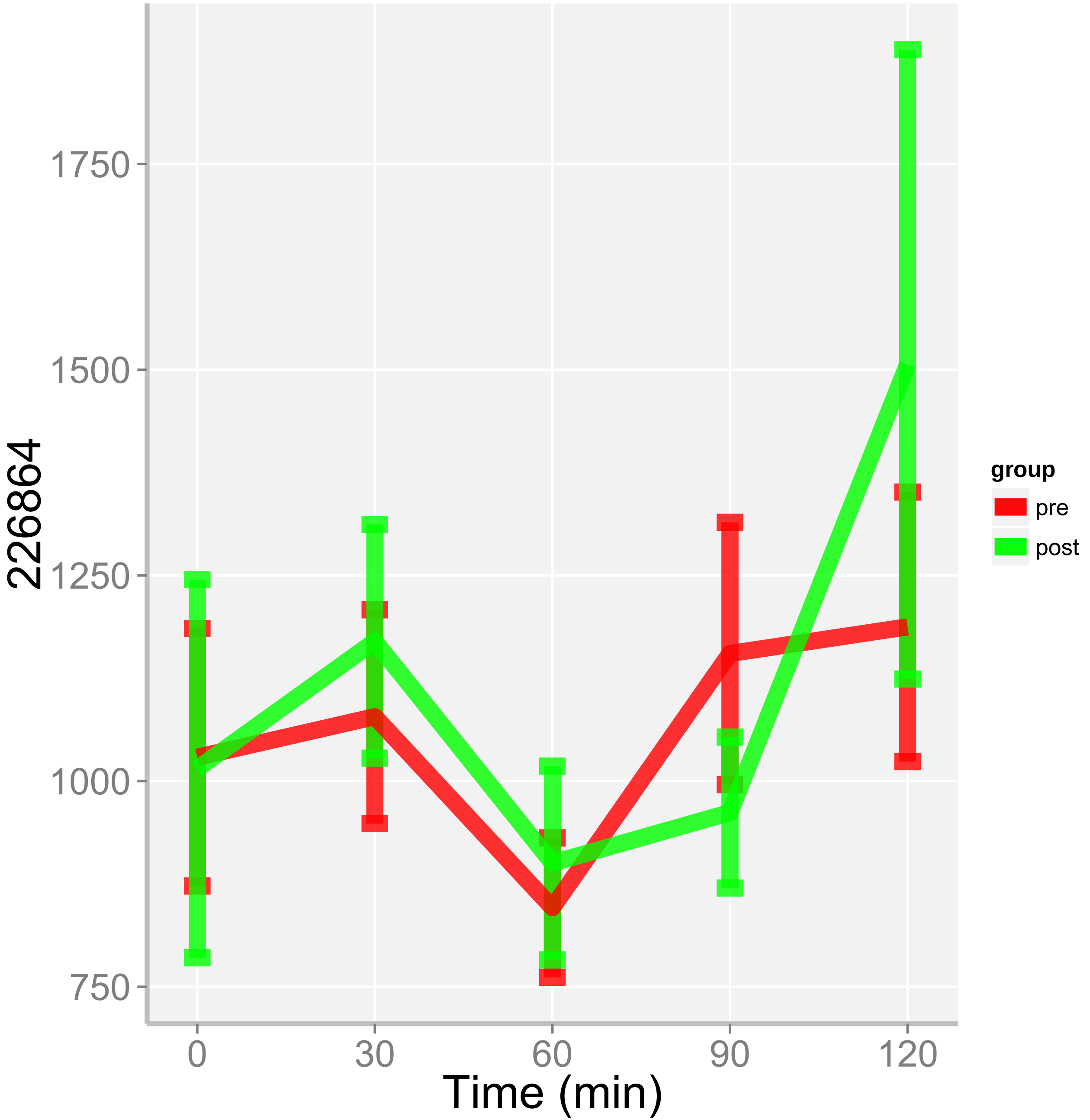

226864
1750
1500
1250
1000
750
0
30
60
90
120
Time (min)
group
pre
post

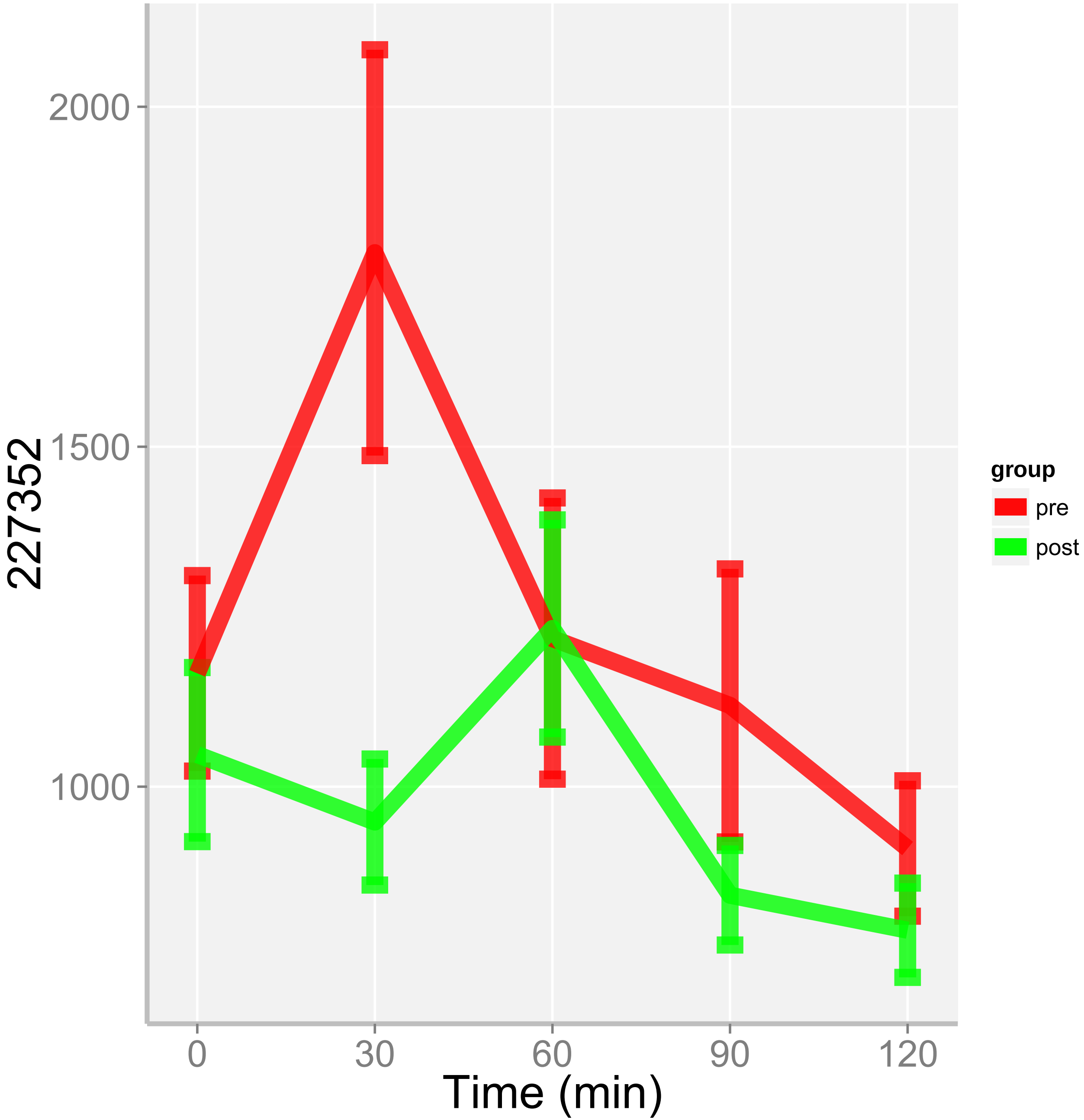

2000
1500
1000
227352
0
30
60
90
120
Time (min)
group
pre
post

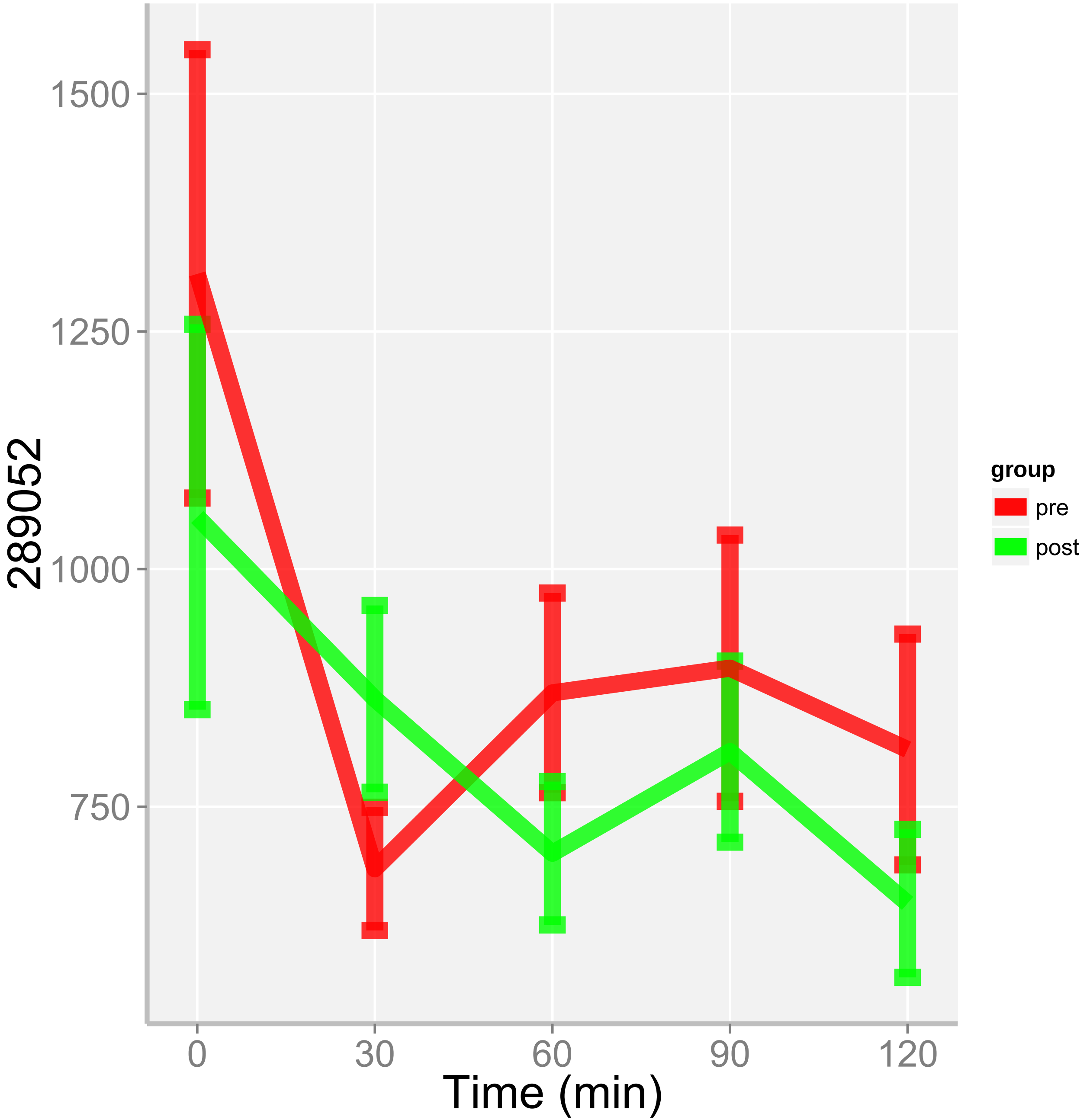

1500
1250
1000
750
289052
0
30
60
90
120
Time (min)
group
pre
post

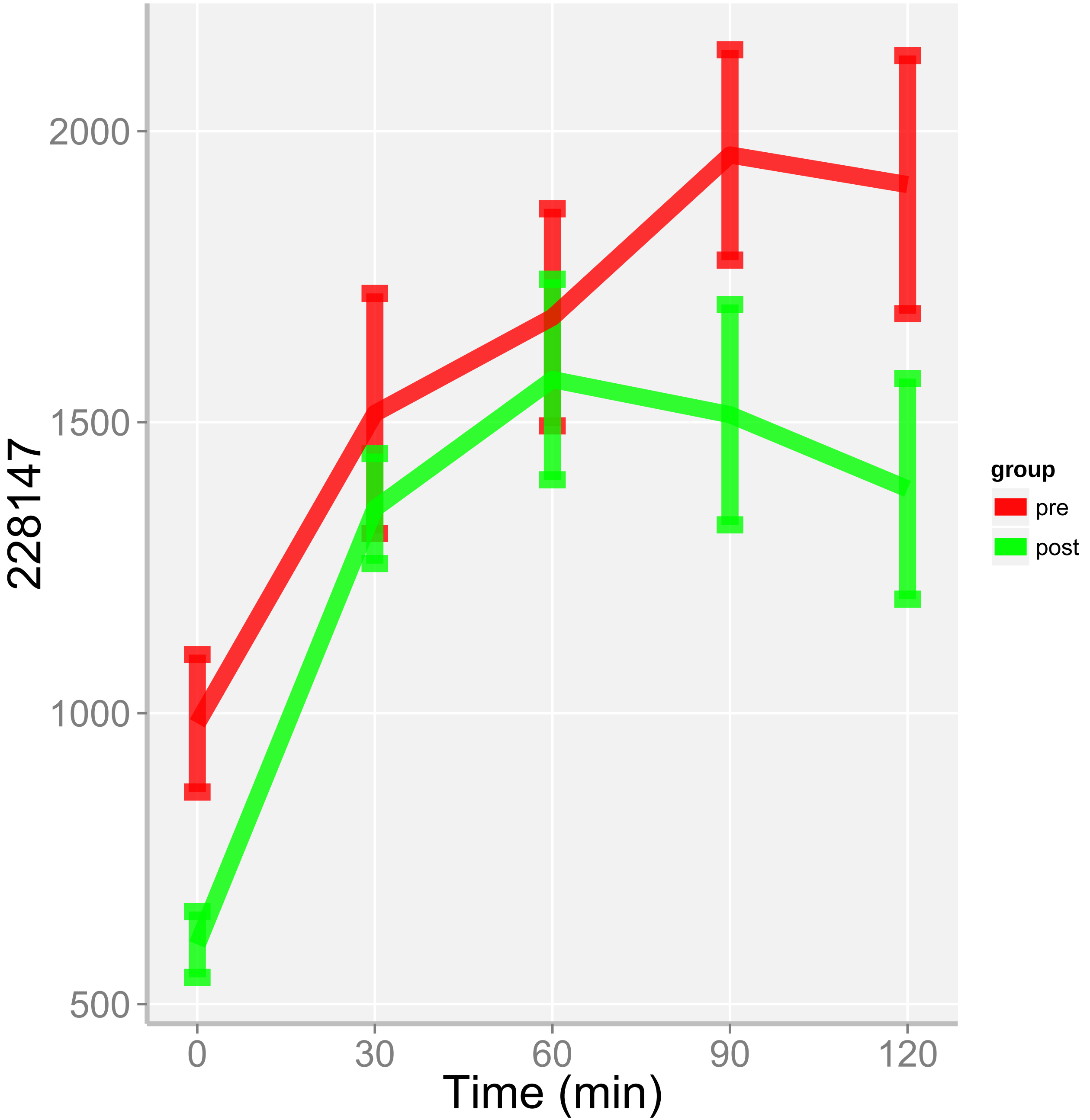

2000
1500
1000
500
228147
0
30
60
90
120
Time (min)
group
pre
post

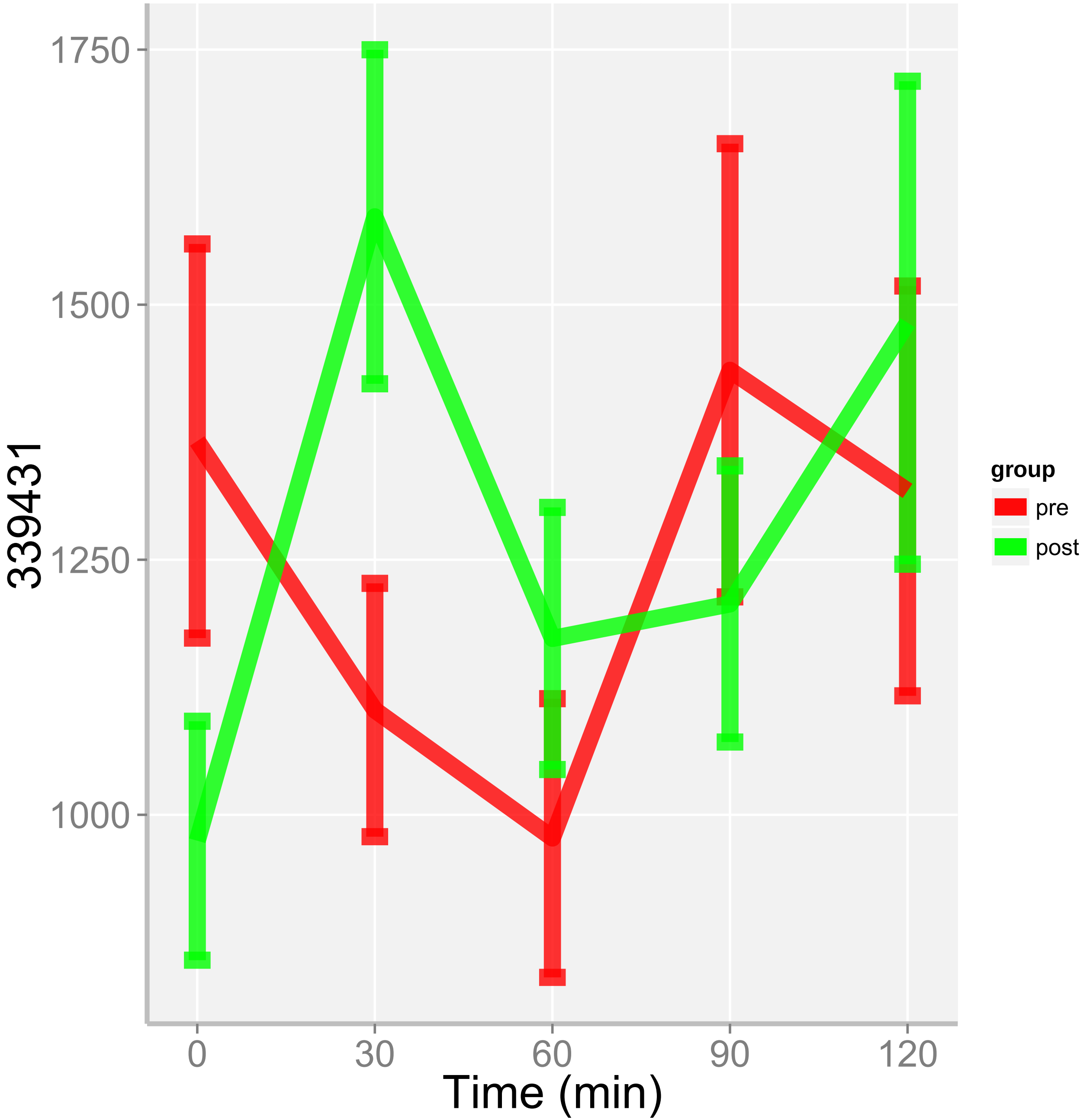

3394
31
1750
1500
1250
1000
0
30
60
90
120
Time (min)
group
pre
post

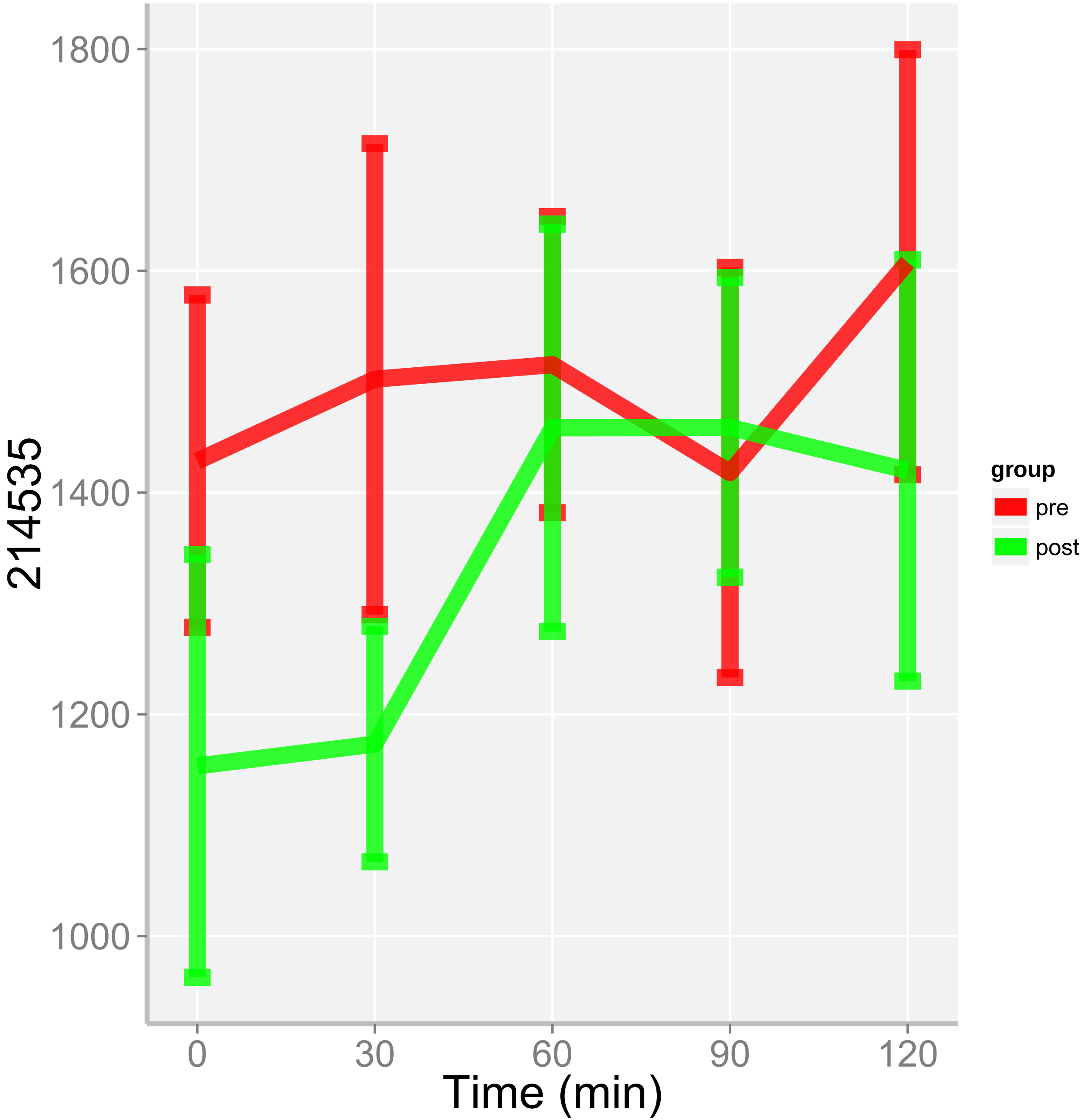

214535
1800
1600
1400
1200
1000
0
30
60
90
120
Time (min)
group
pre
post

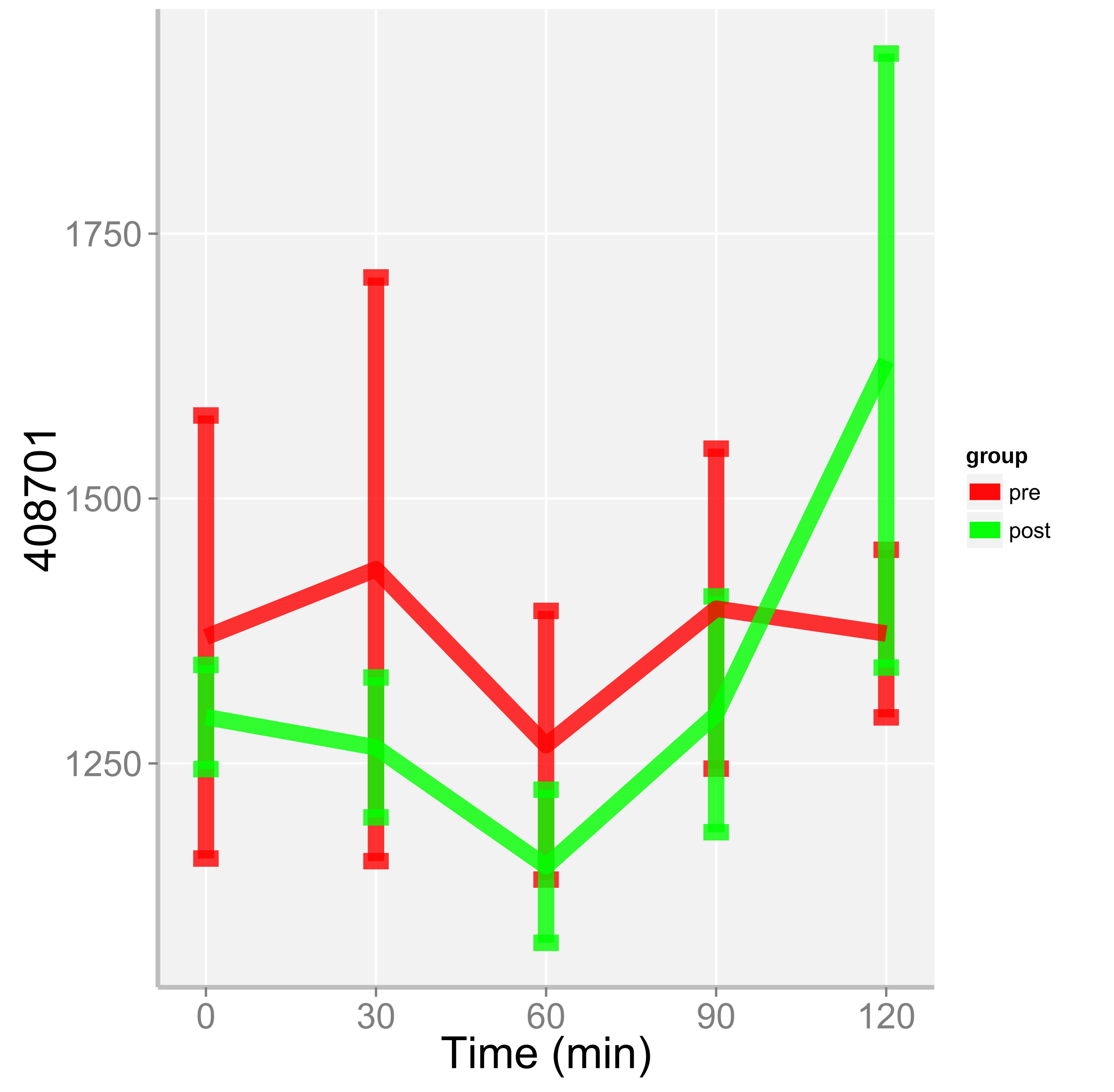

4087O1
1750
1500
1250
0
30
60
90
120
Time (min)
group
pre
post

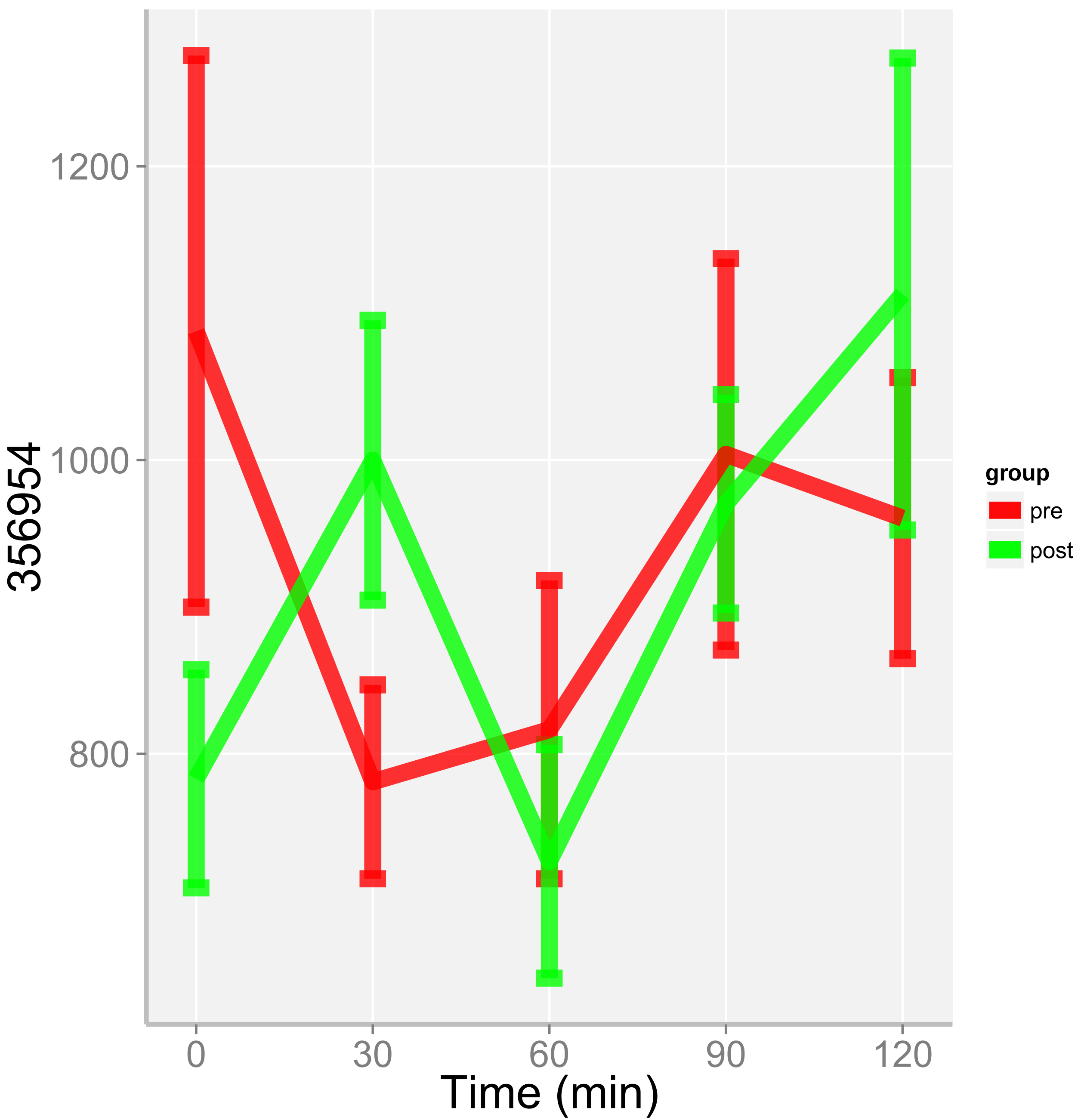

356954
1200
1000
800
0
30
60
90
120
Time (min)
group
pre
post

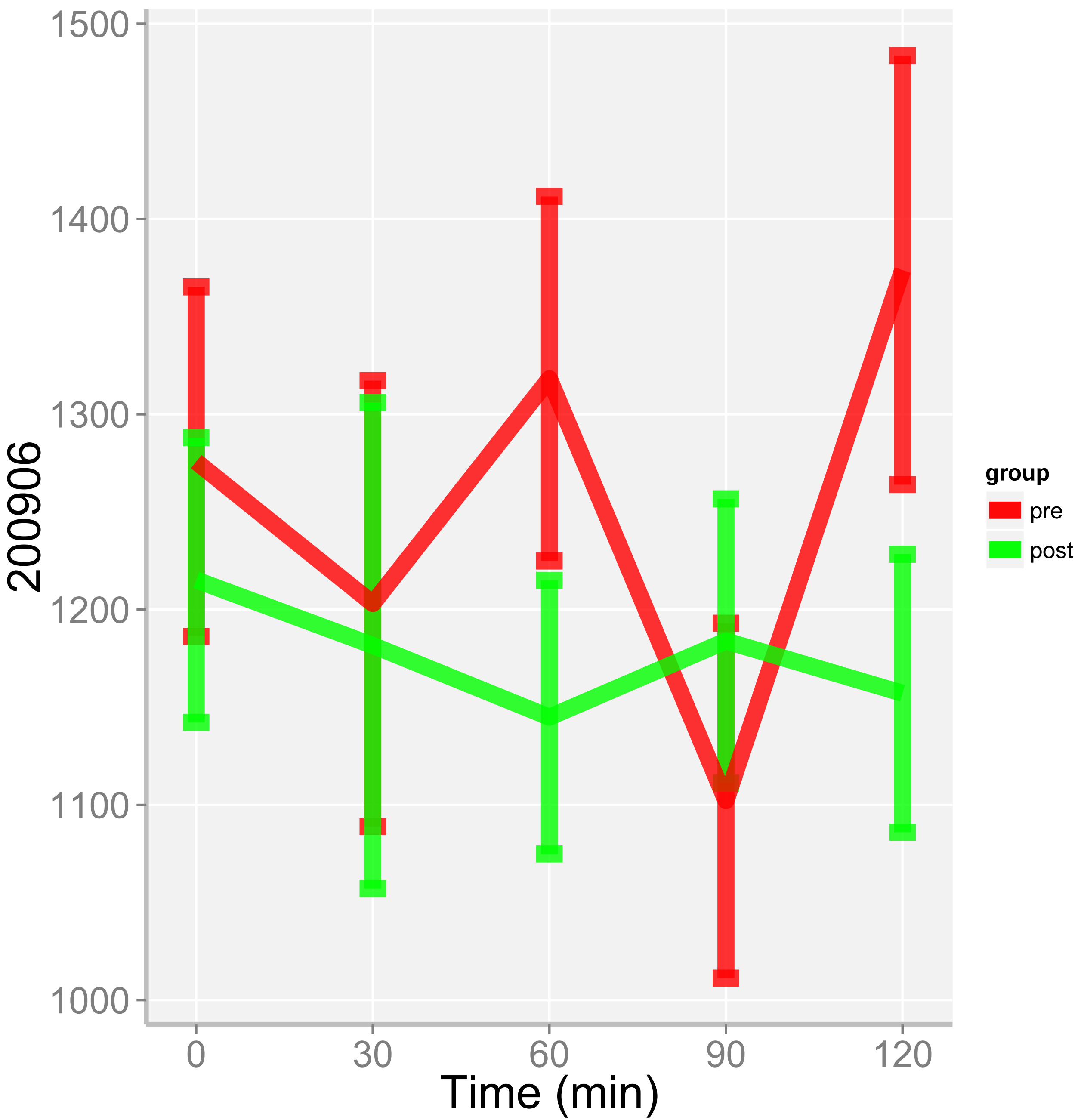

1500
1400
1300
1200
1100
1000
200906
0
30
60
90
120
Time (min)
group
pre
post

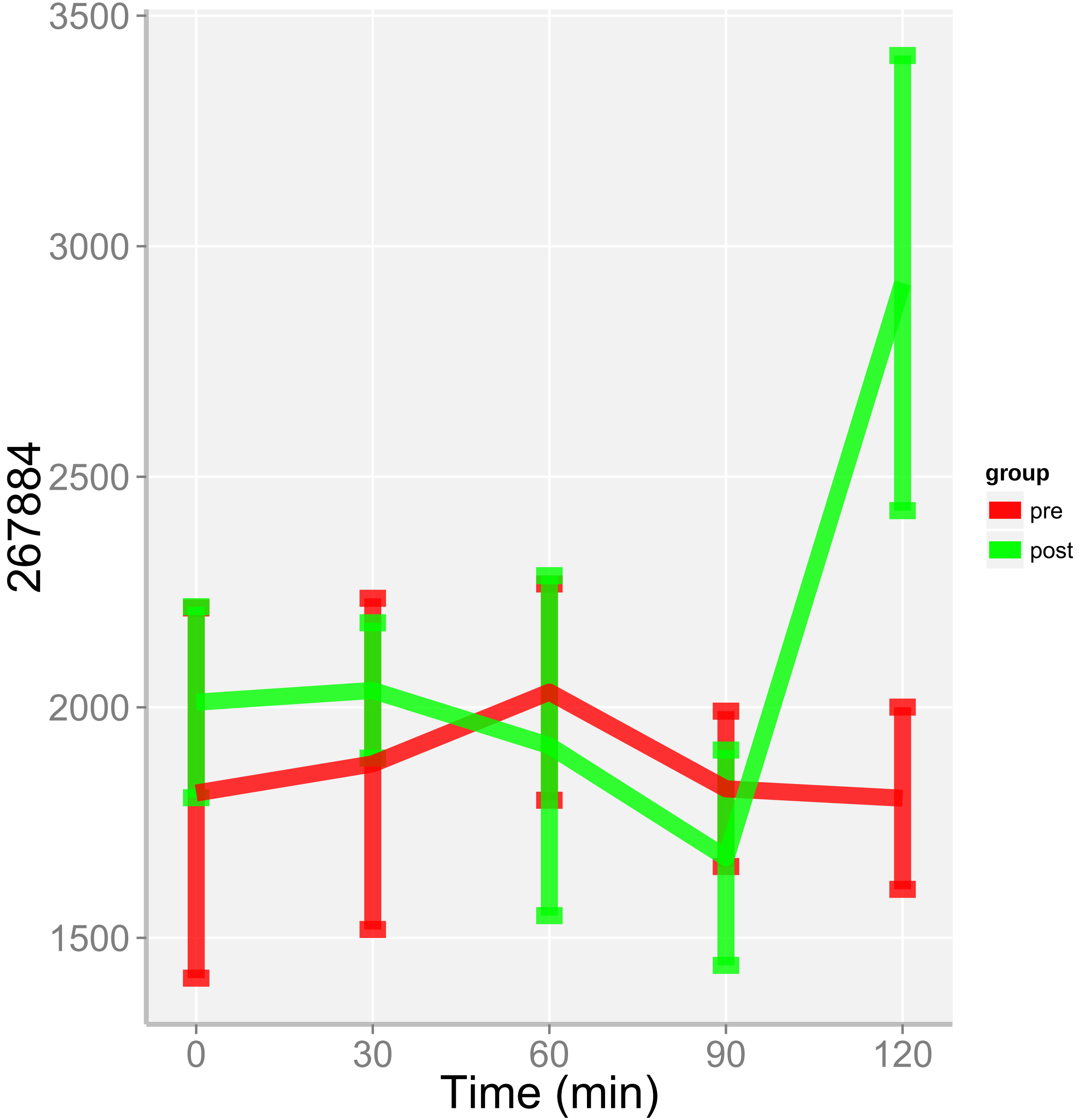

3500
3000
2500
2000
1500
267884
0
30
60
90
120
Time (min)
group
pre
post

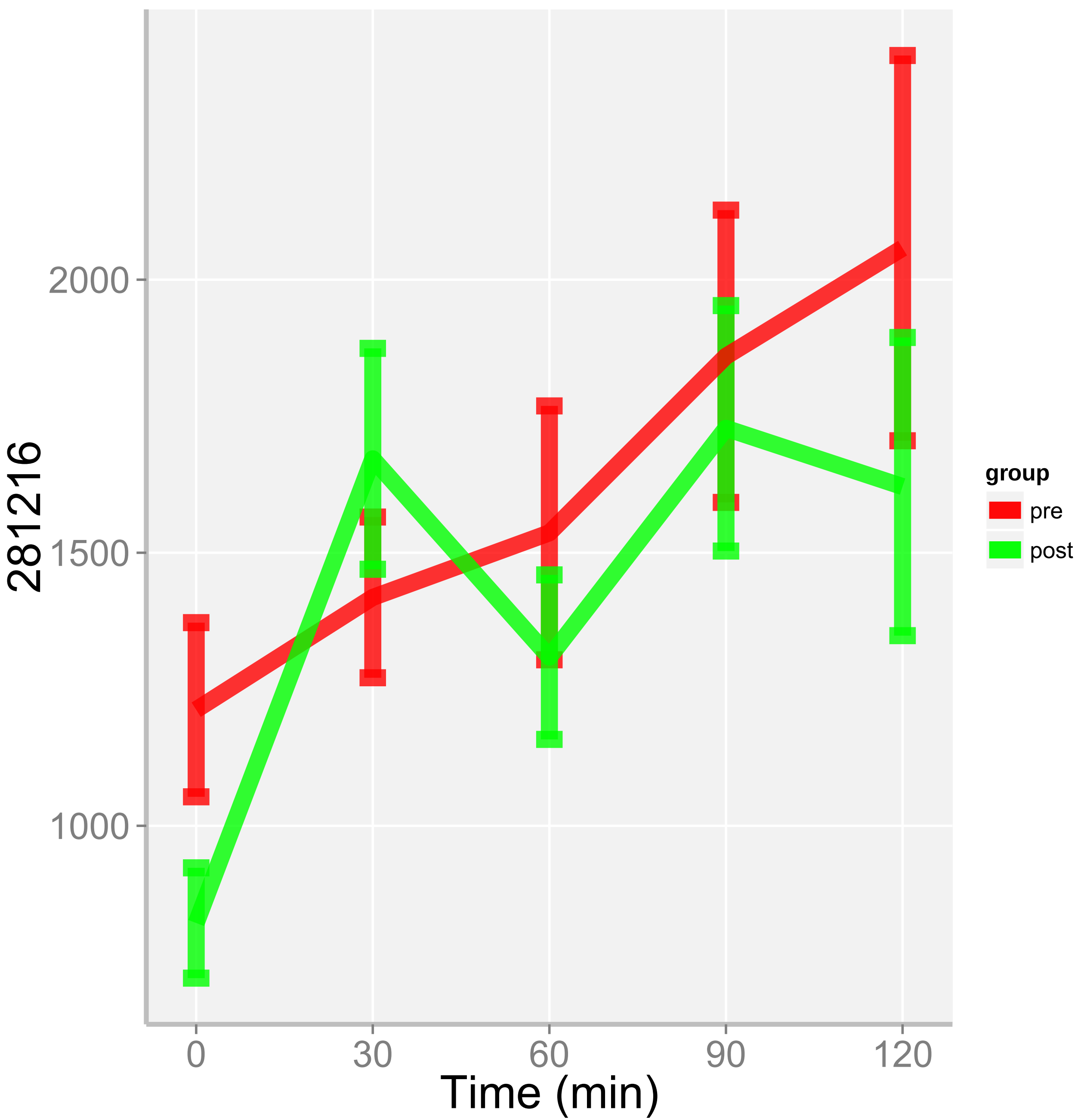
281216
2000
1500
1000
0
30
60
90
120
Time (min)
group
pre
post

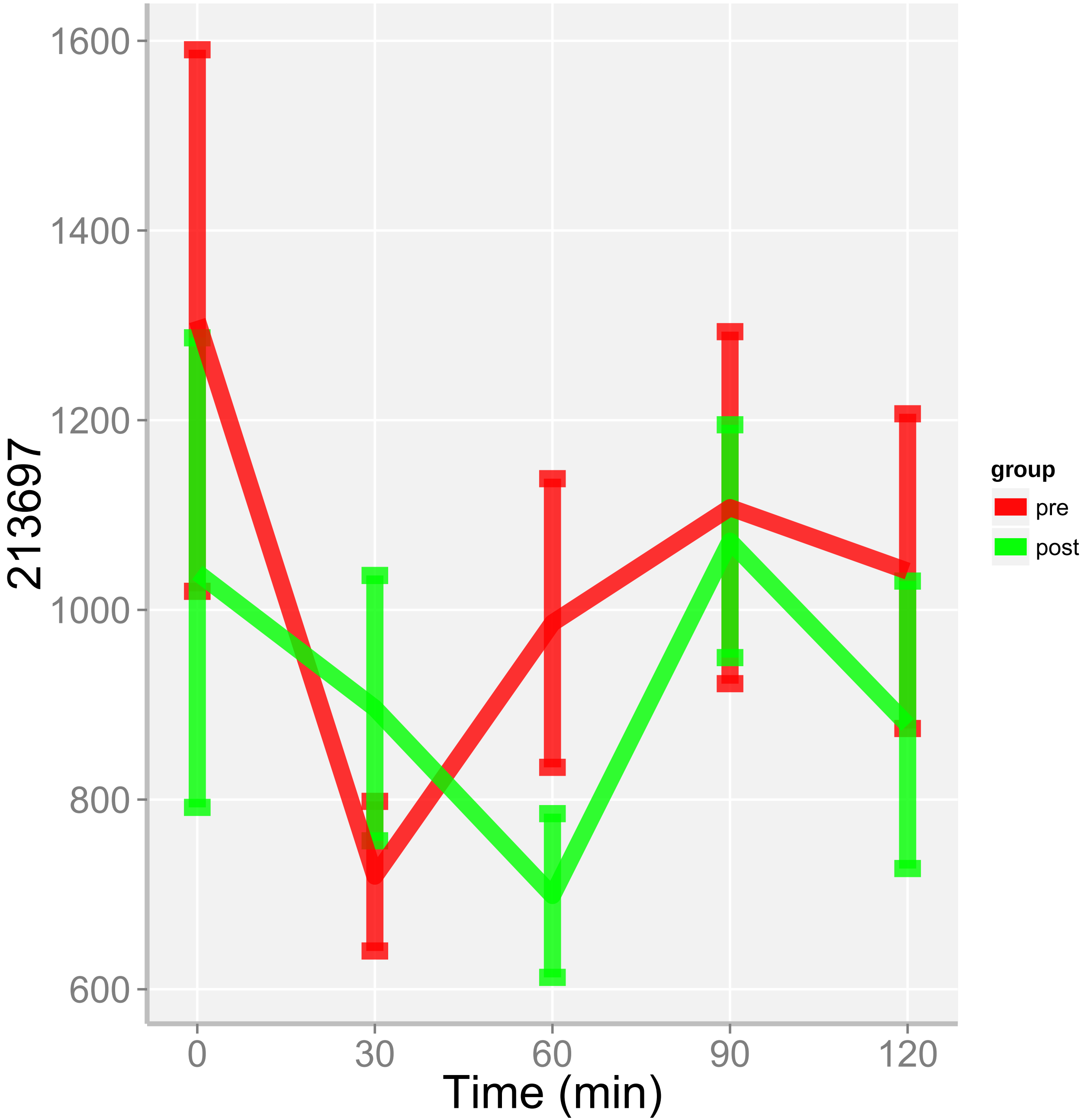

213697
1600
1400
1200
1000
800
600
0
30
60
90
120
Time (min)
group
pre
post

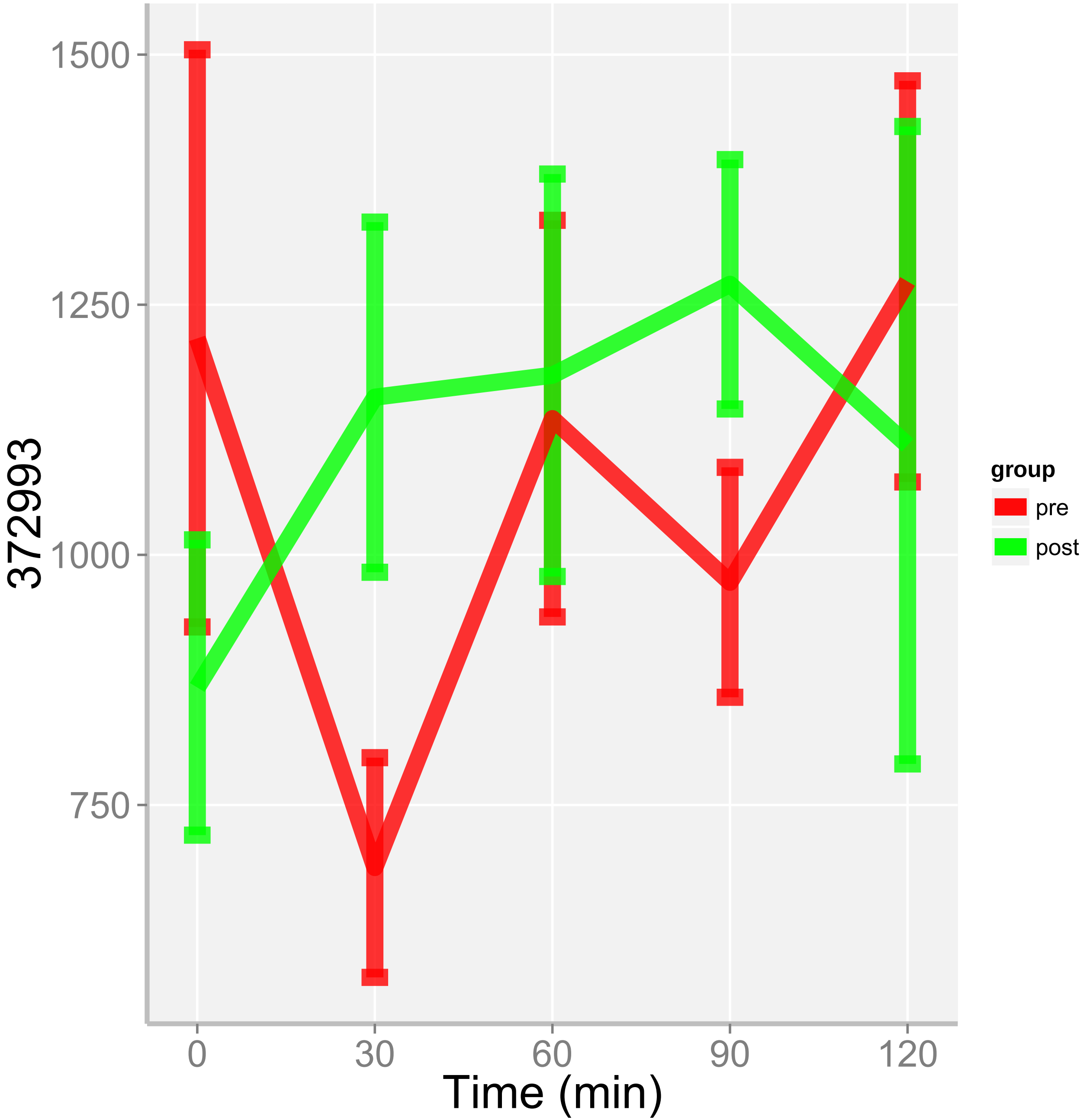

1500
1250
1000
750
372993
0
30
60
90
120
Time (min)
group
pre
post

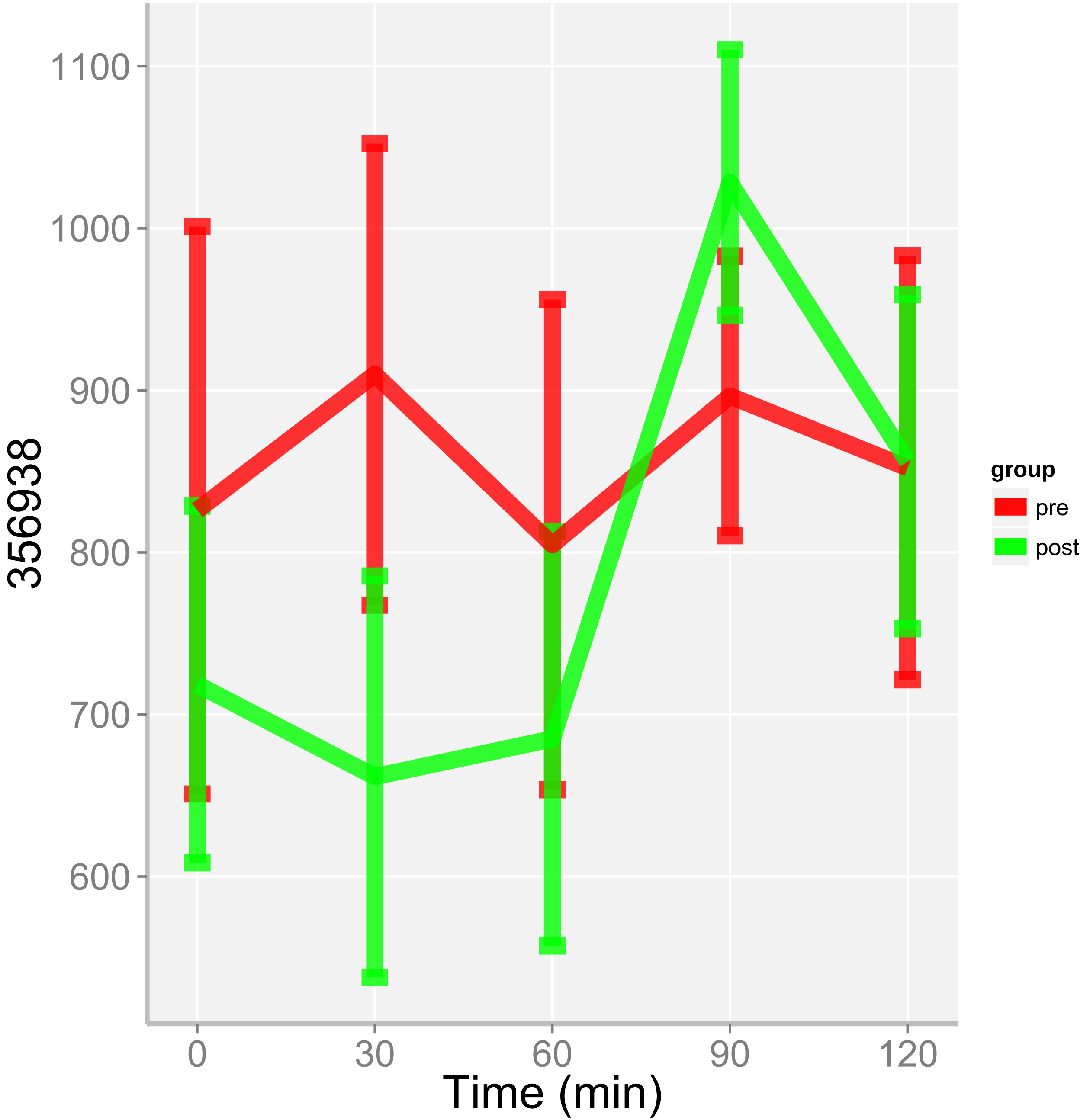

1100
1000
900
800
700
600
0
30
60
90
120
Time (min)
356938
group
pre
post

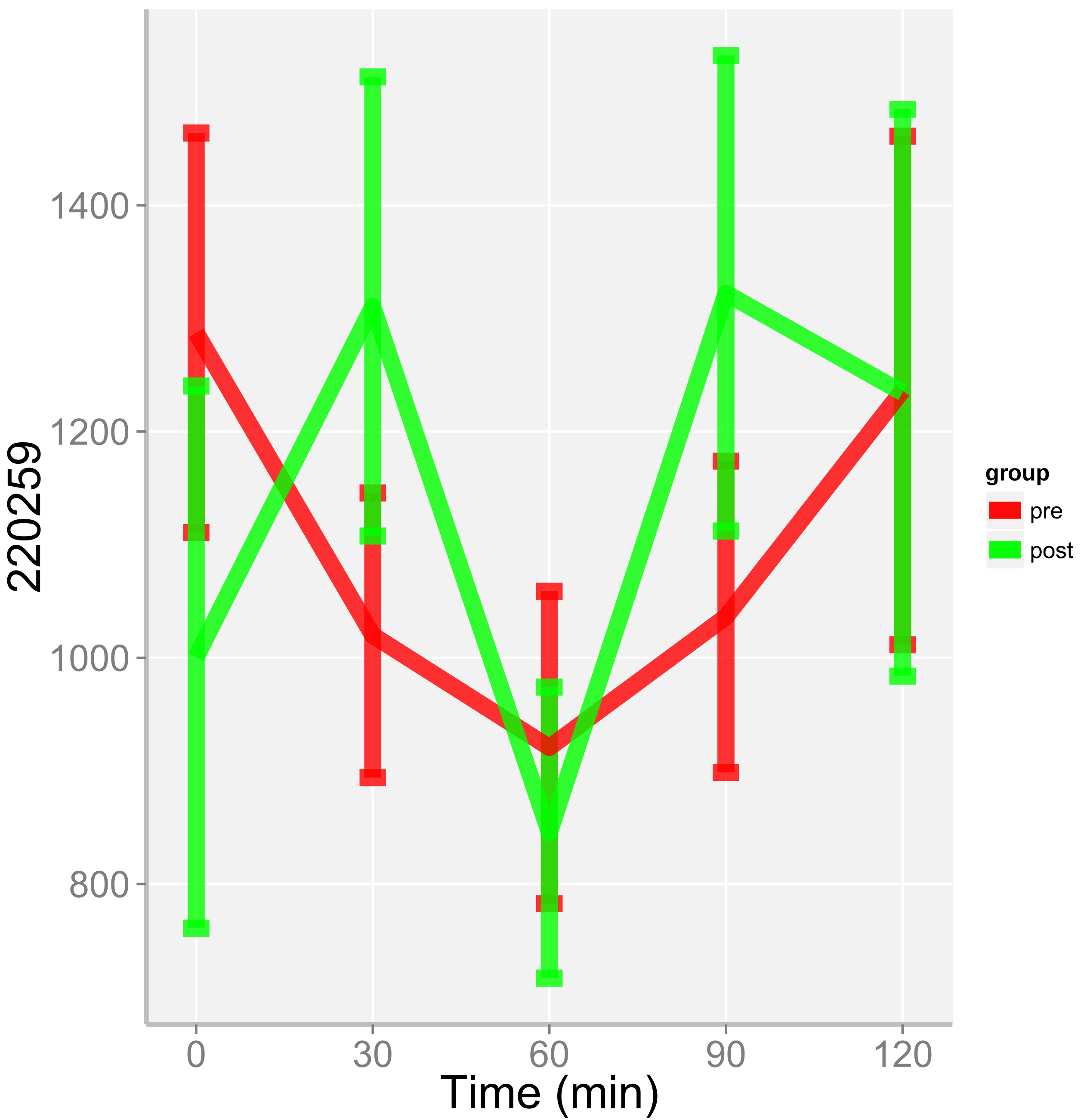

220259
1400
1200
1000
800
0
30
60
90
120
Time (min)
group
pre
post

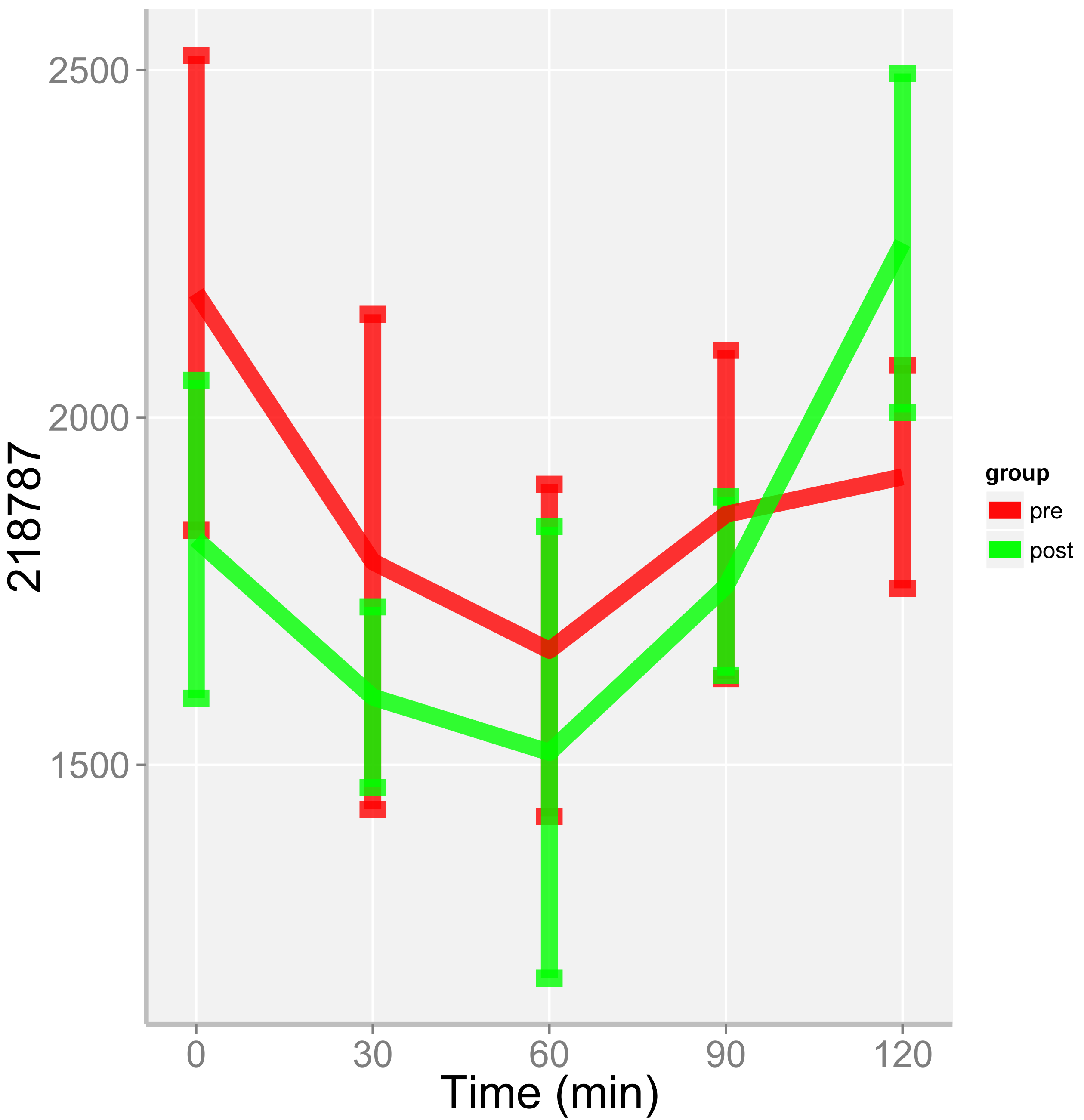

2500
2000
1500
218787
0
30
60
90
120
Time (min)
group
pre
post

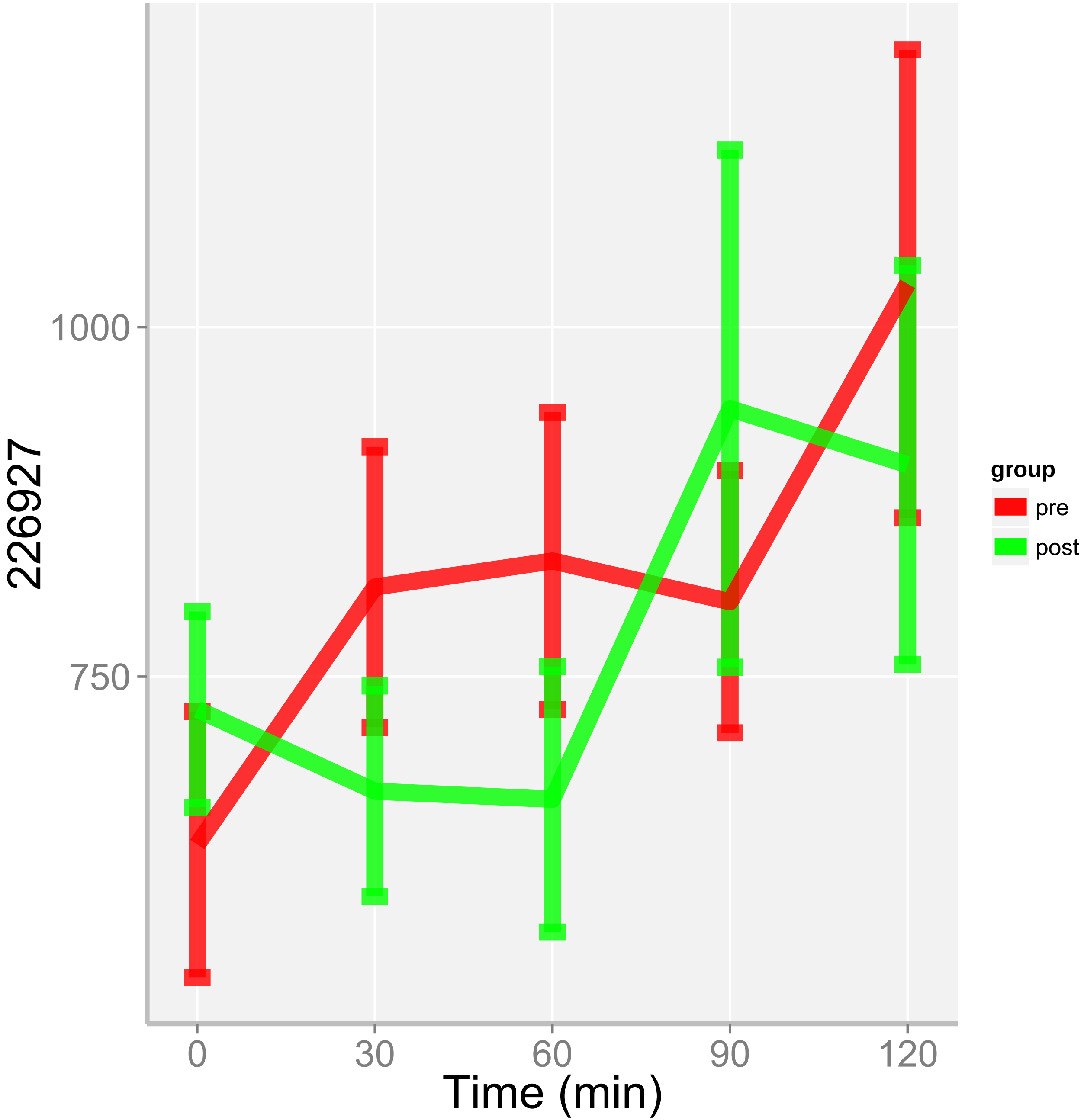

226927
1000
750
0
30
60
90
120
Time (min)
group
pre
post

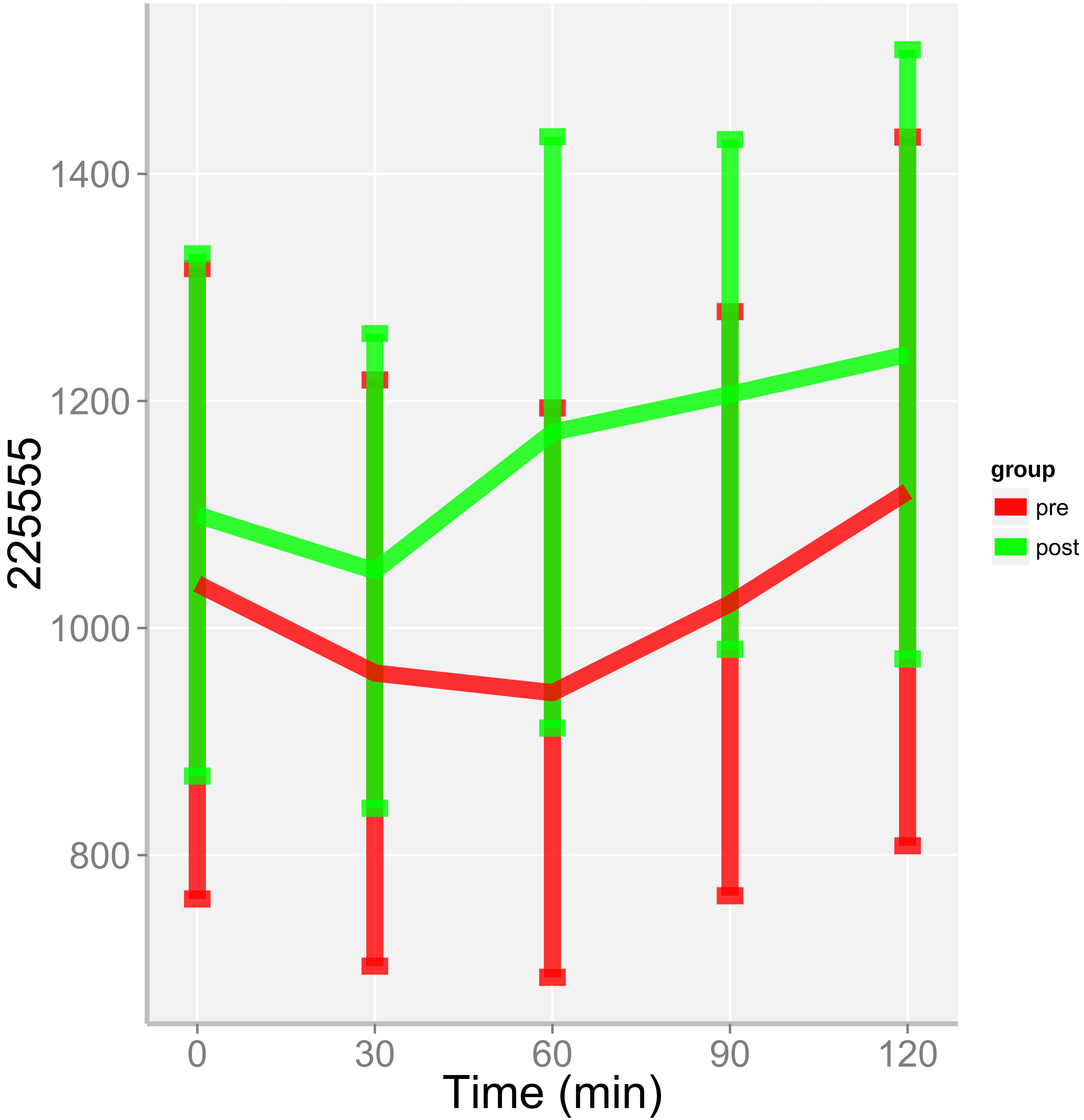

225555
1400
1200
1000
800
0
30
60
90
120
Time (min)
group
pre
post

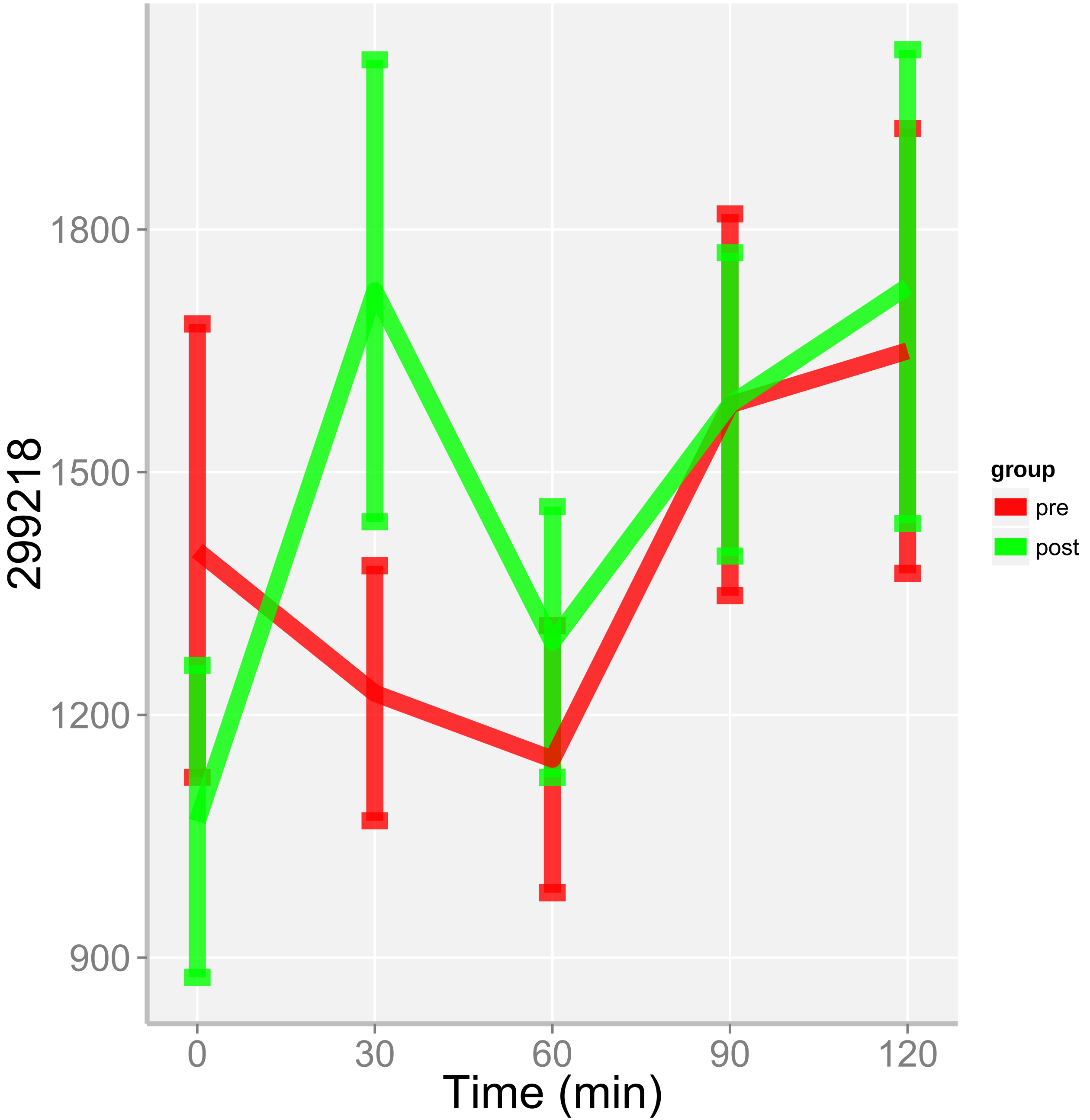

299218
1800
1500
1200
900
0
30
60
90
120
Time (min)
group
pre
post

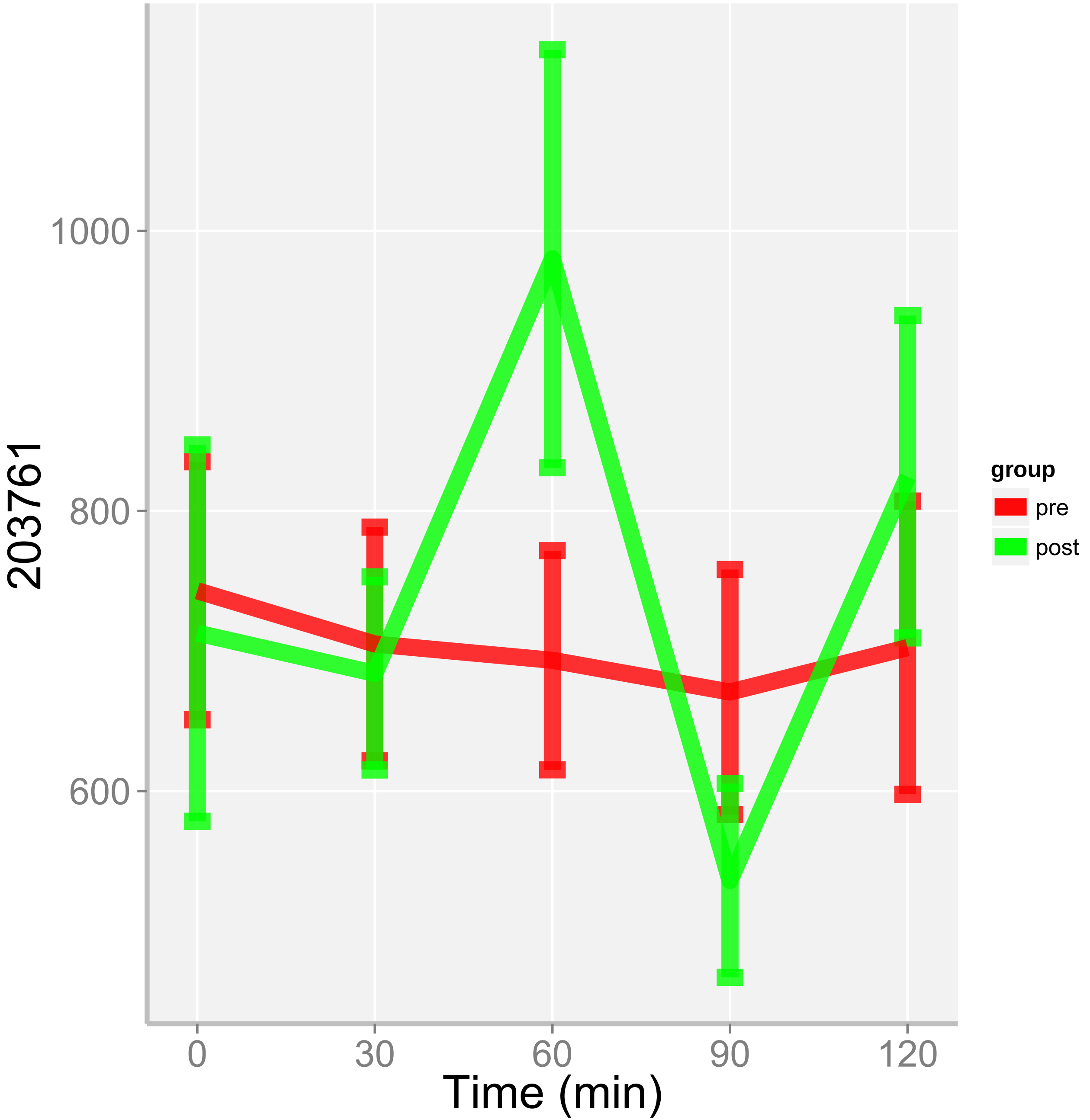

203761
1000
800
600
0
30
60
90
120
Time (min)
group
pre
post

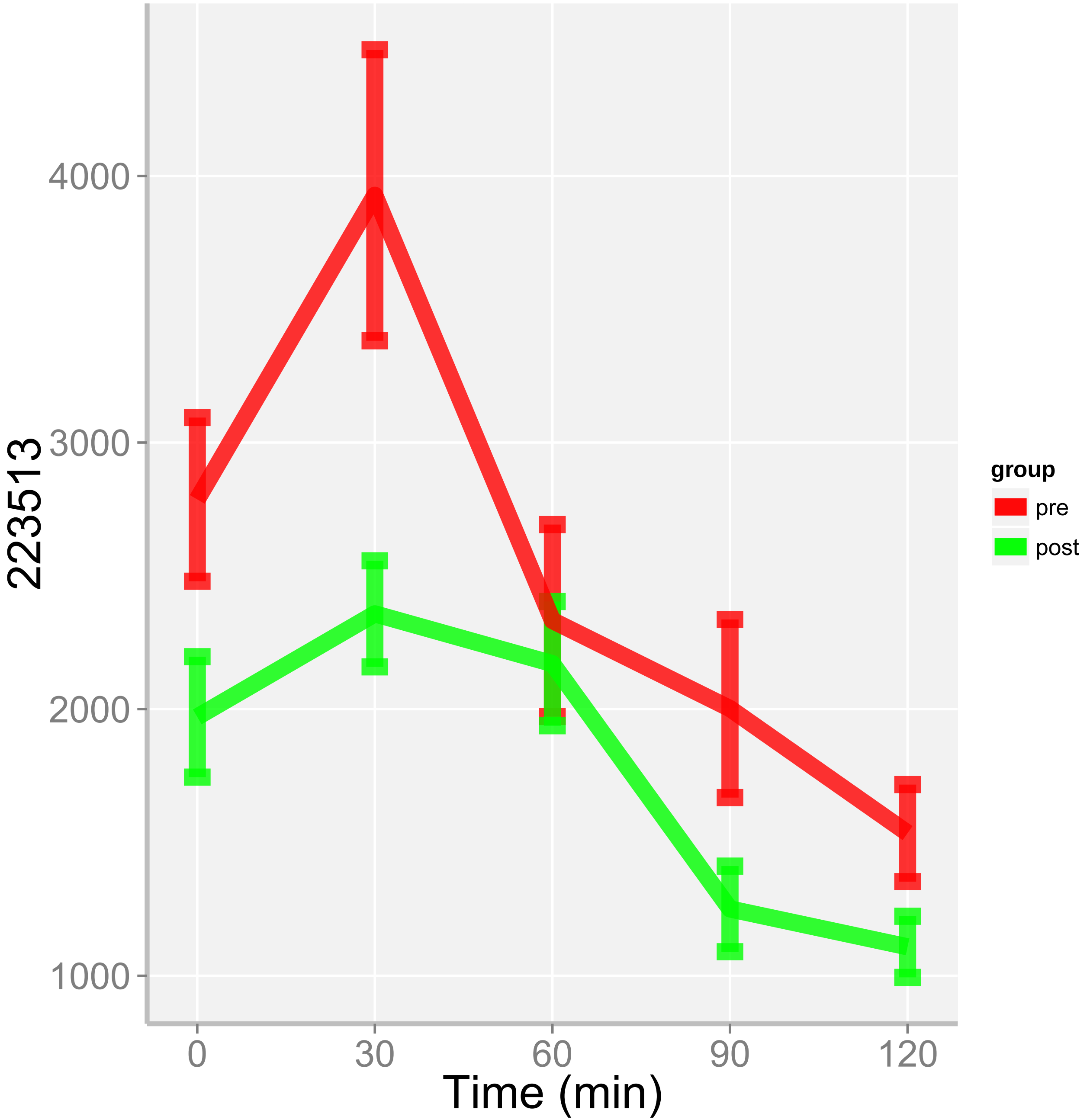

223513
4000
3000
2000
1000
0
30
60
90
120
Time (min)
group
pre
post

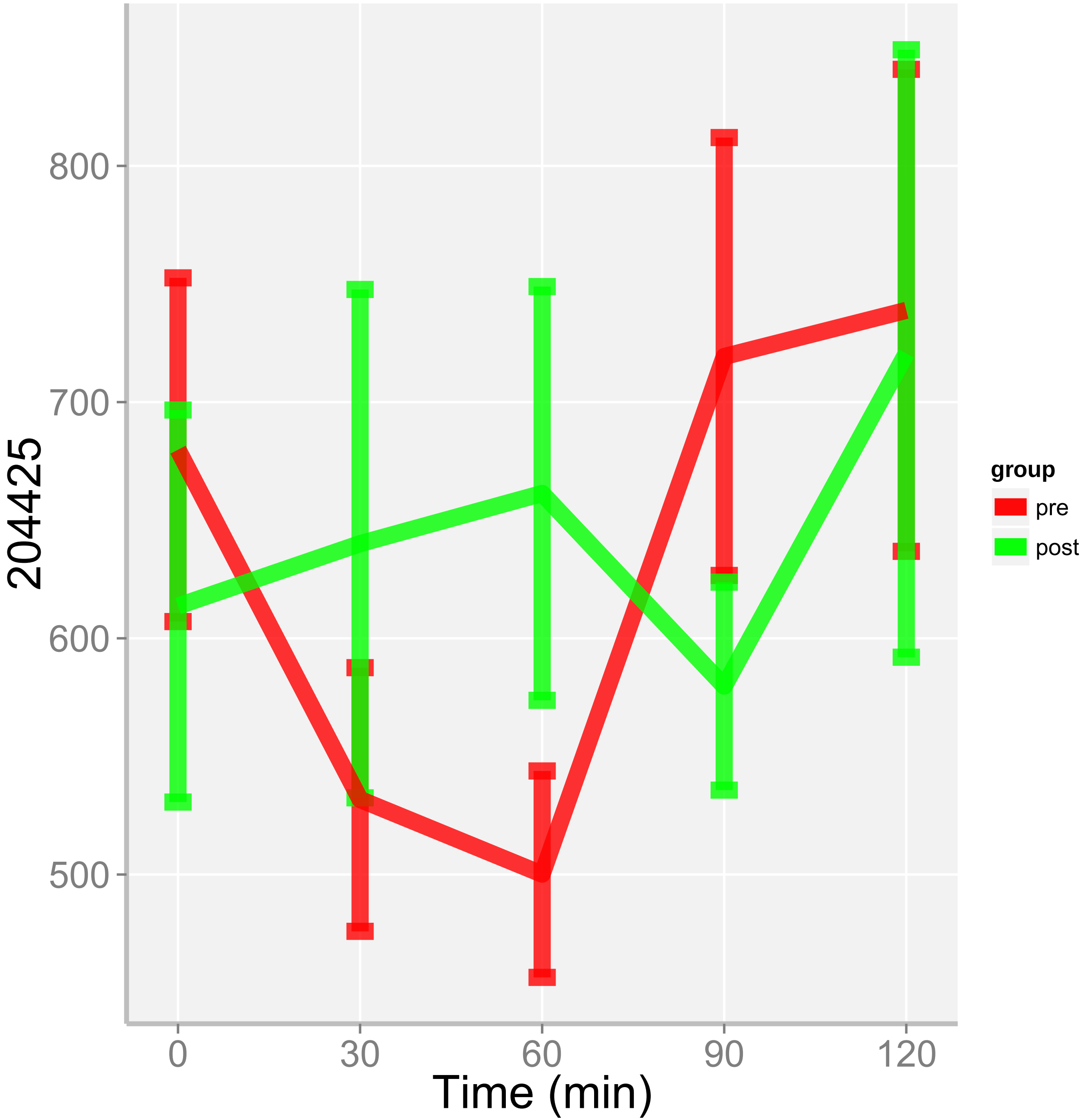

204425
800
700
600
500
0
30
60
90
120
Time (min)
group
pre
post

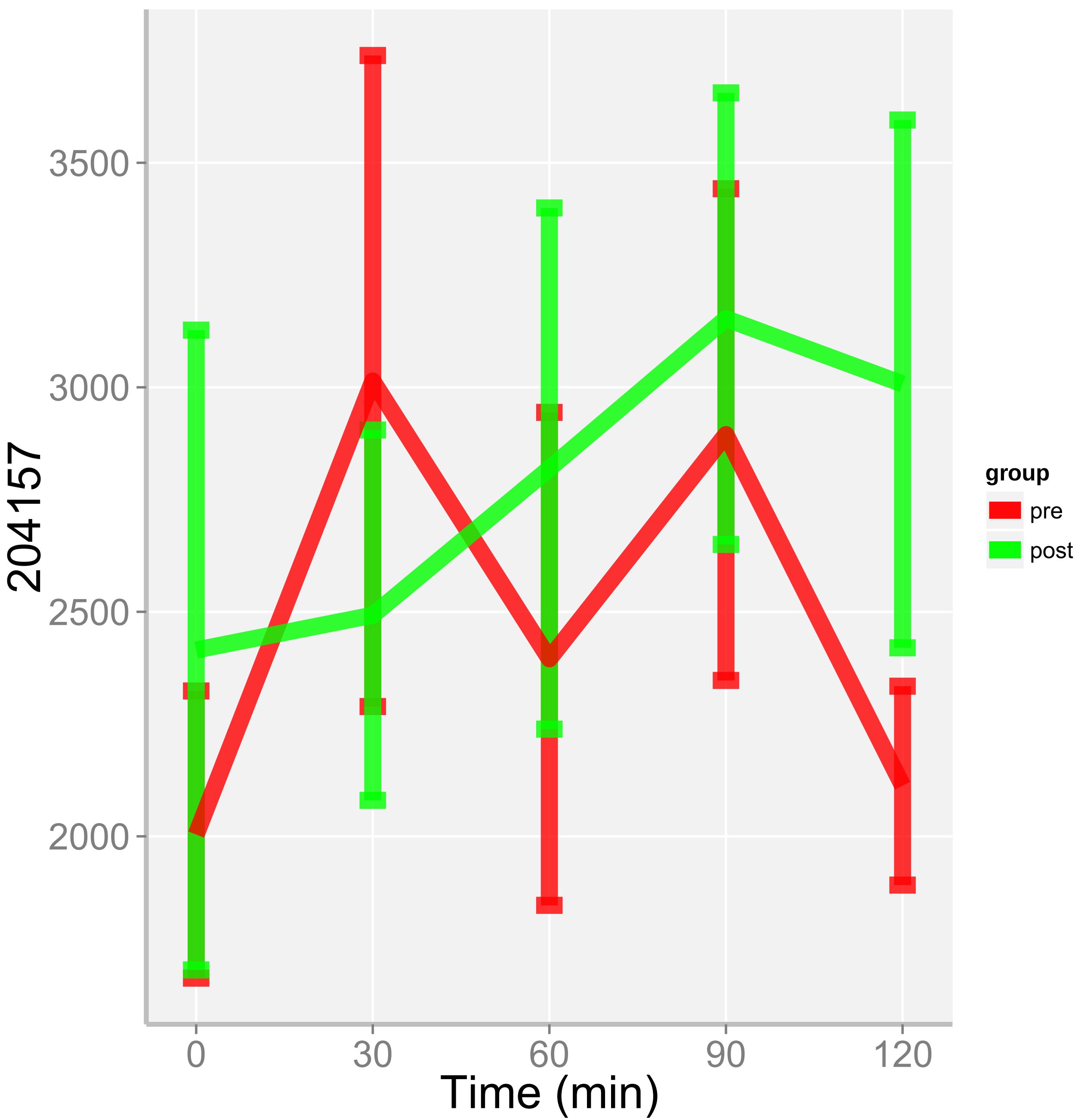

3500
3000
2500
2000
204157
0
30
60
90
120
Time (min)
group
pre
post

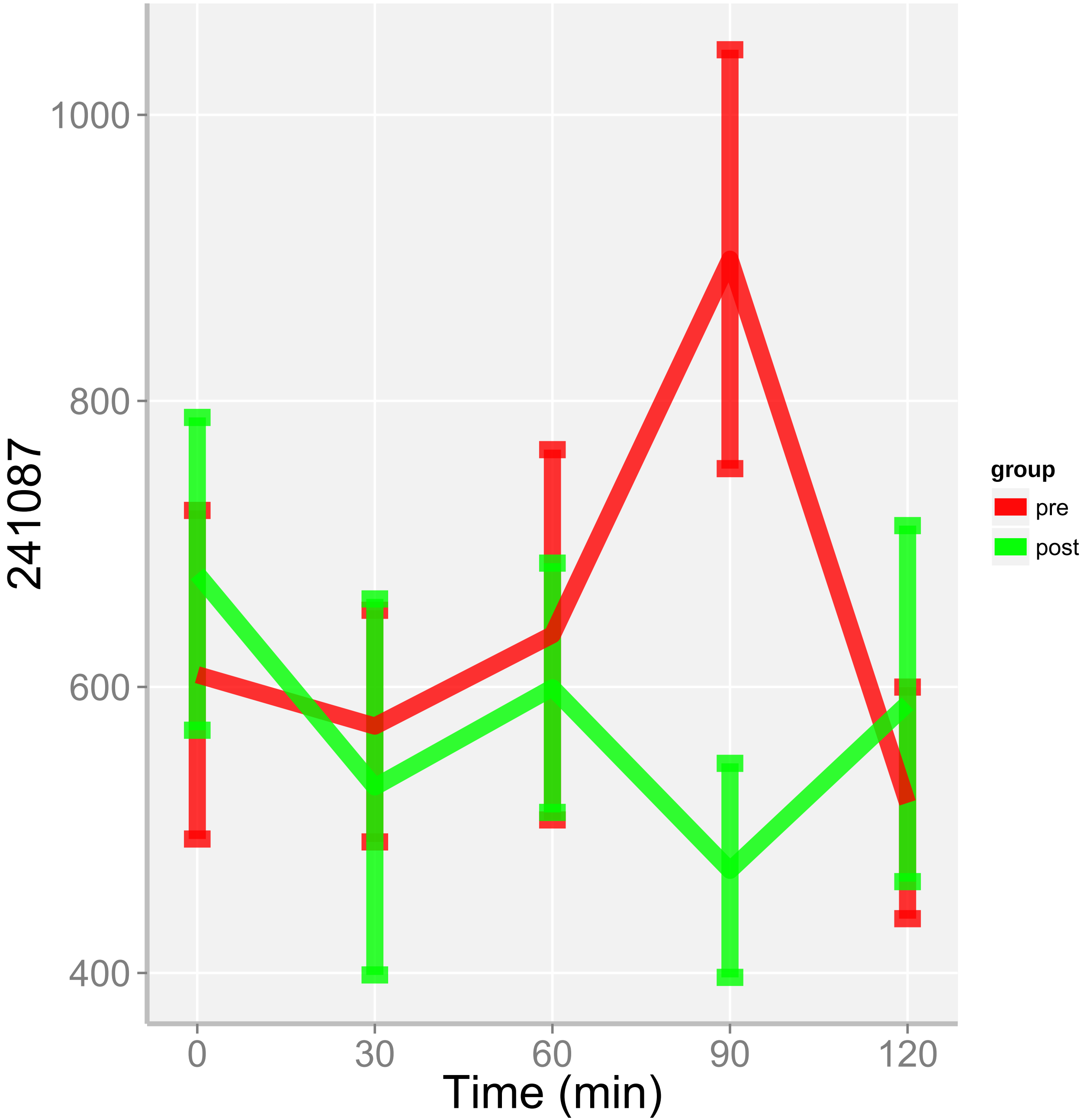

1000
800
600
400
241087
0
30
60
90
120
Time (min)
group
pre
post

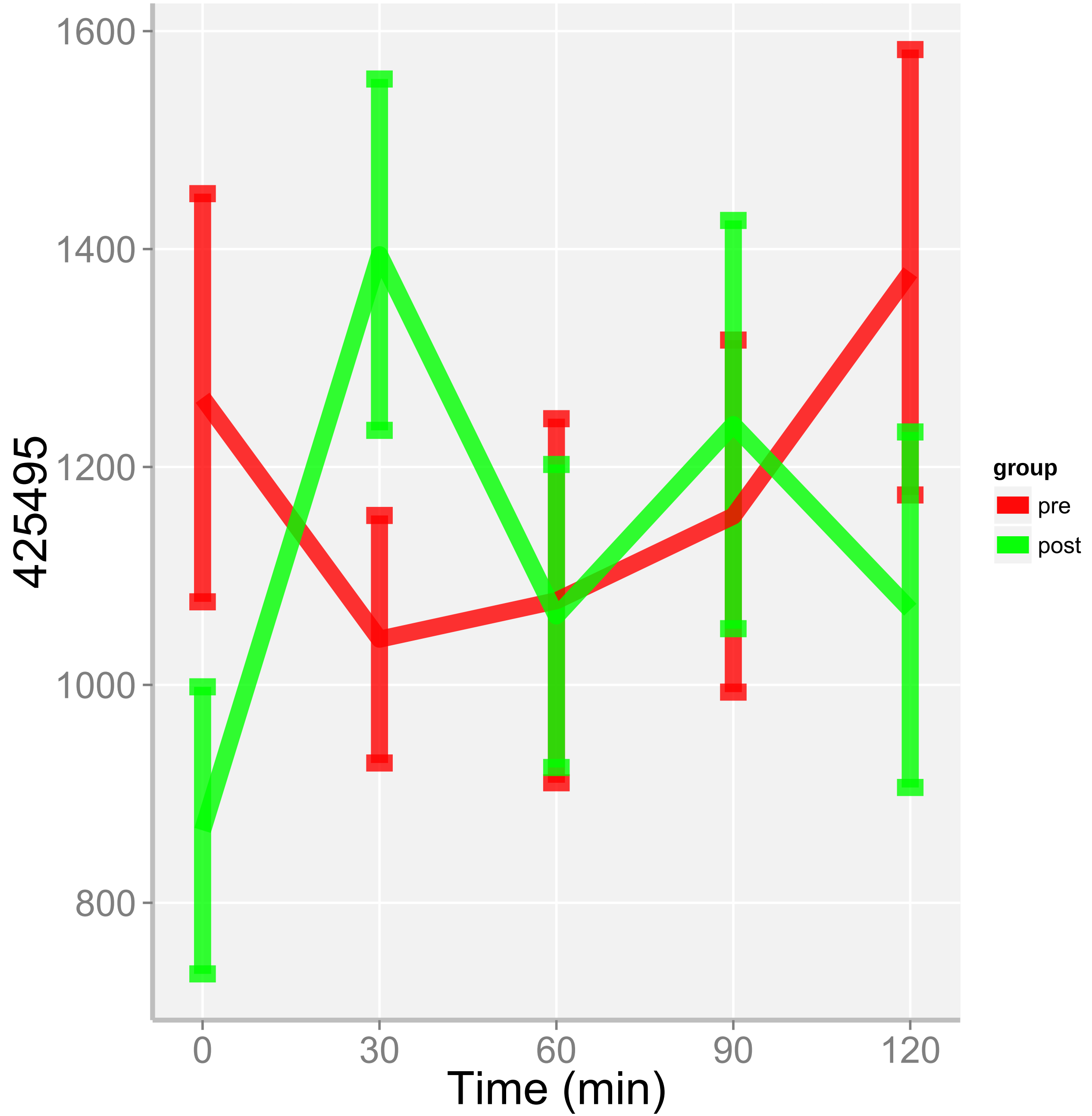

1600
1400
1200
1000
800
4254495
0
30
60
90
120
Time (min)
group
pre
post

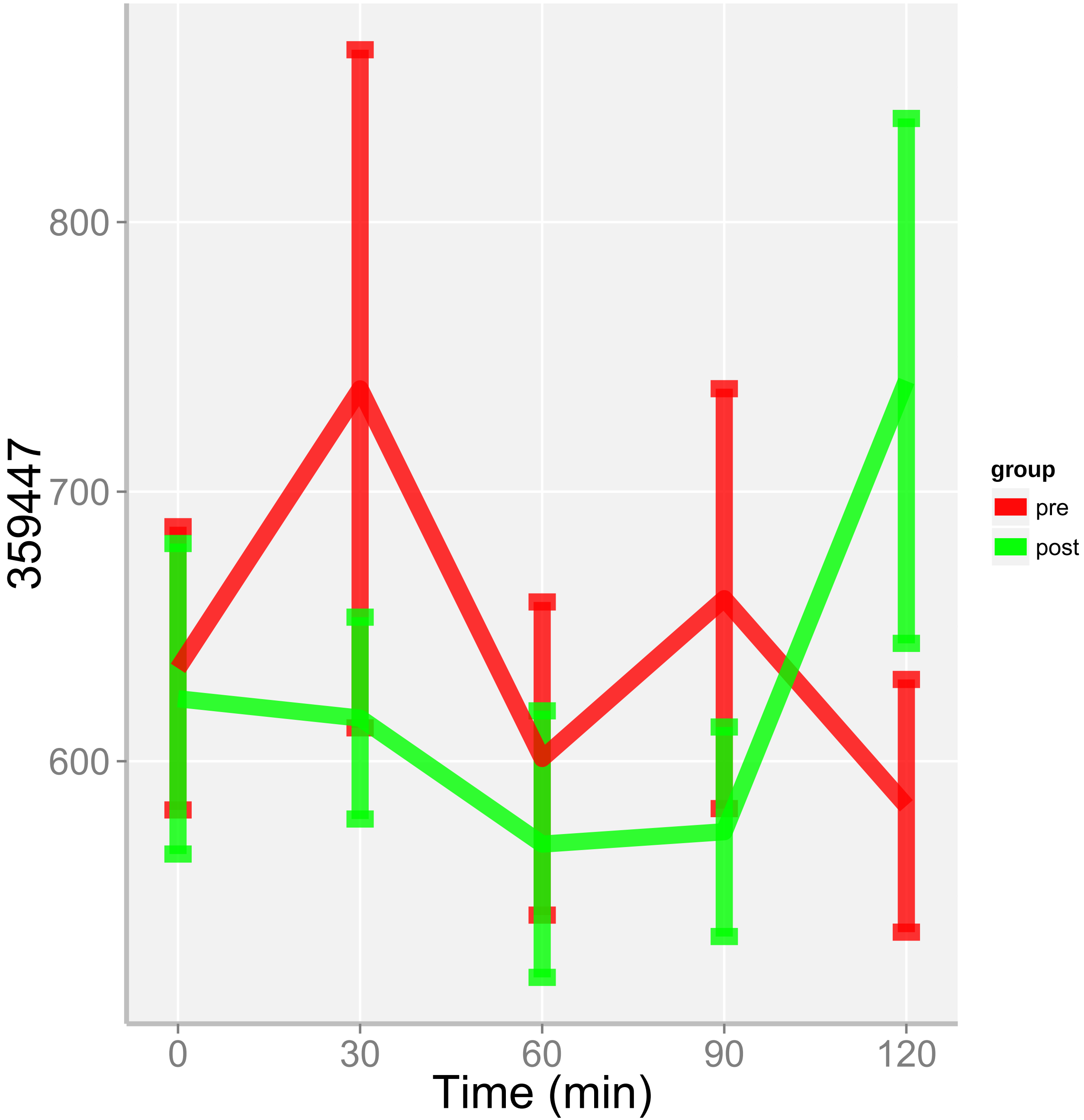

359447
800
700
600
0
30
60
90
120
Time (min)
group
pre
post

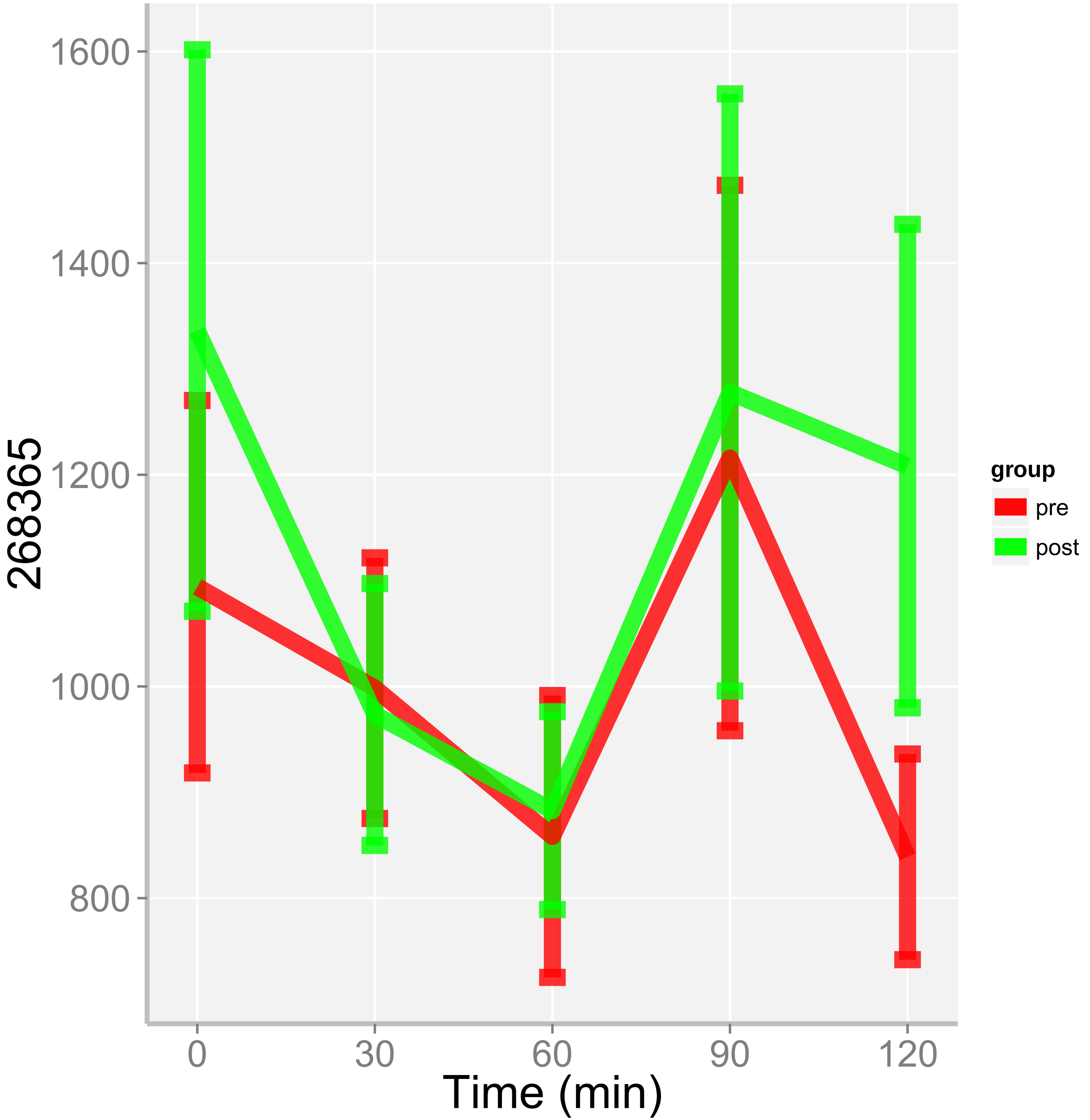

2683655
1600
1400
1200
1000
800
0
30
60
90
120
Time (min)
group
pre
post

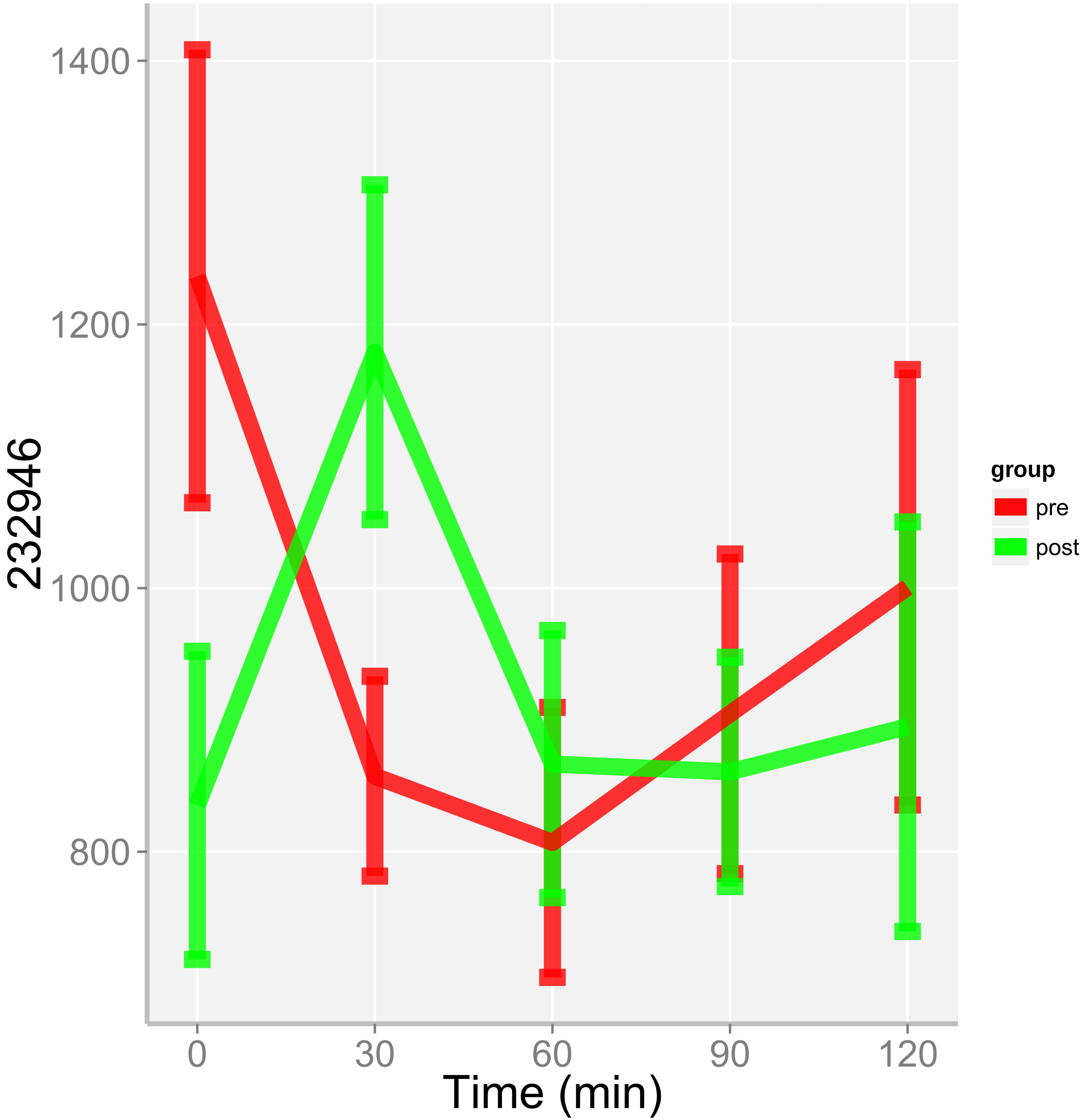

1400
1200
1000
800
232946
0
30
60
90
120
Time (min)
group
pre
post

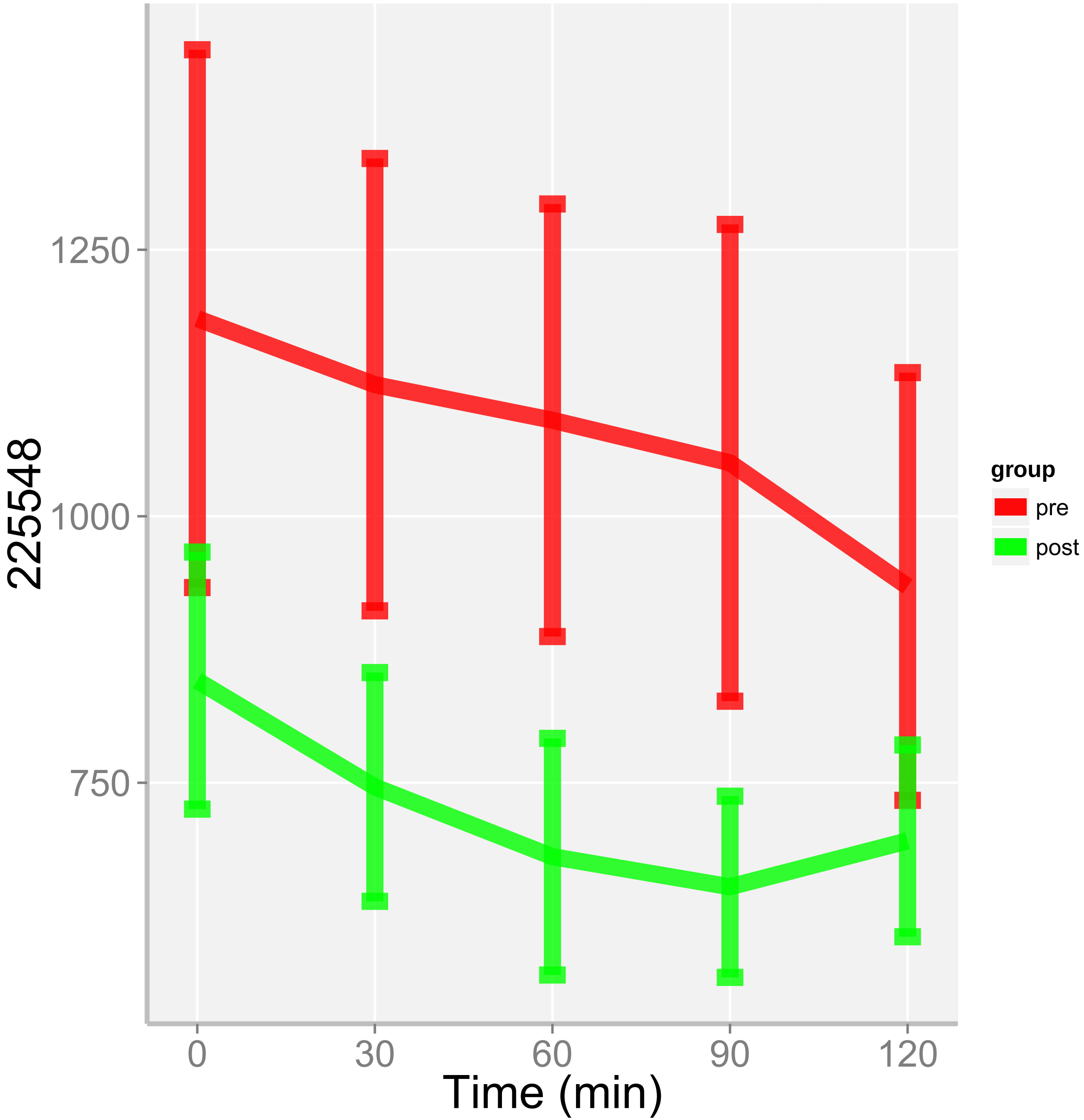

225548
1250
1000
750
0
30
60
90
120
Time (min)
group
pre
post

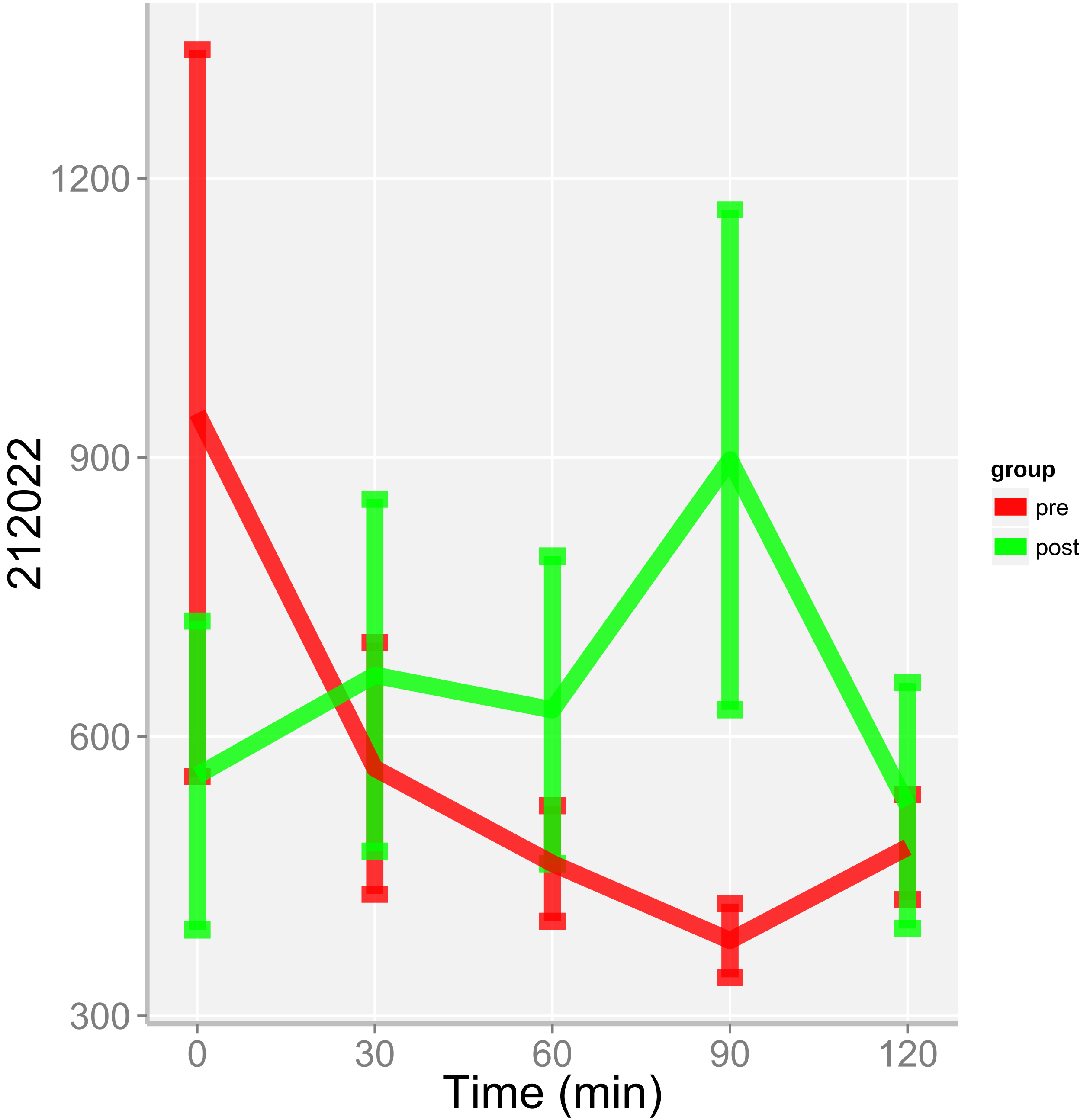
212022
1200
900
600
300
0
30
60
90
120
Time (min)
group
pre
post

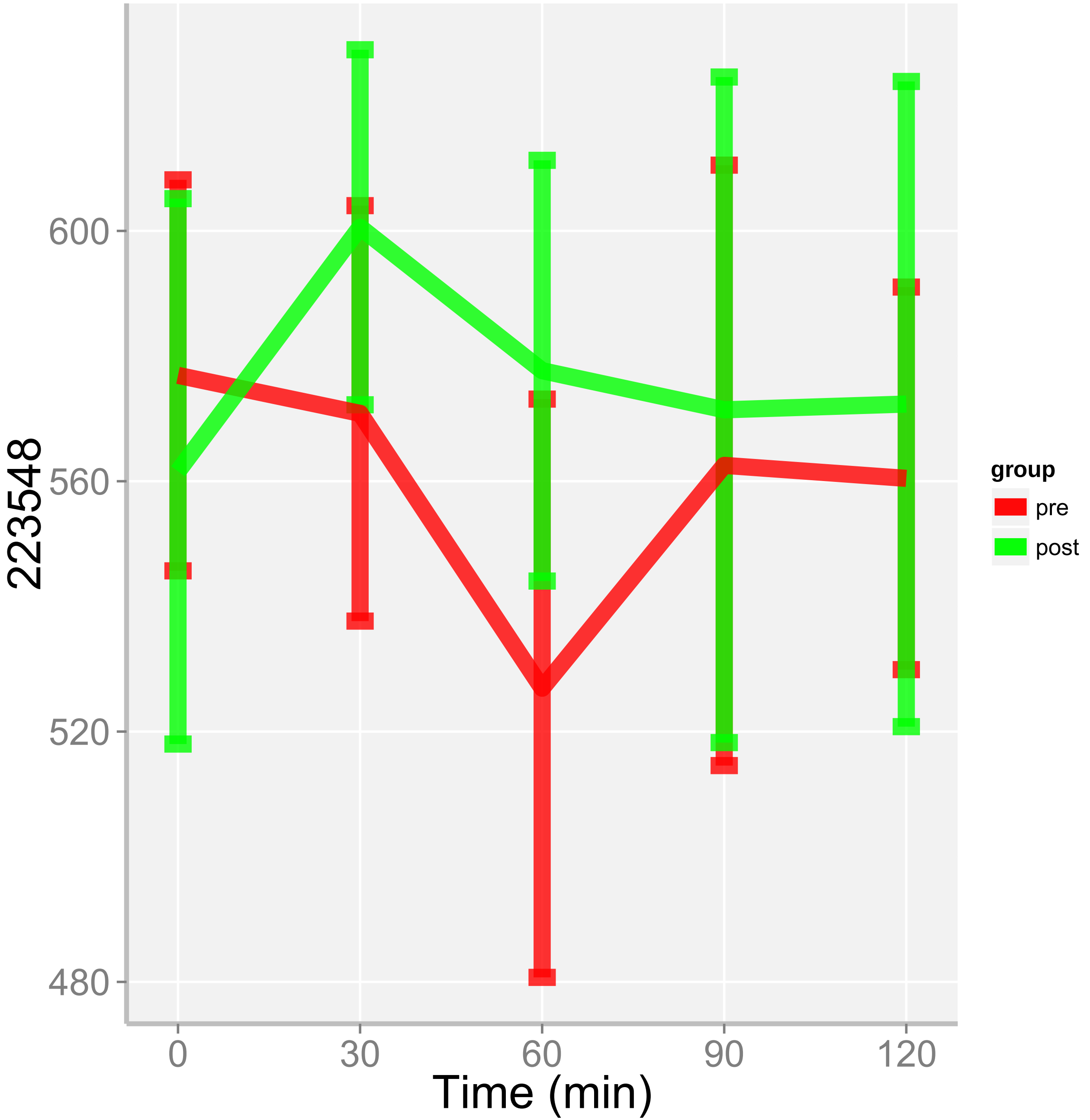

223548
600
560
520
480
0
30
60
90
120
Time (min)
group
pre
post

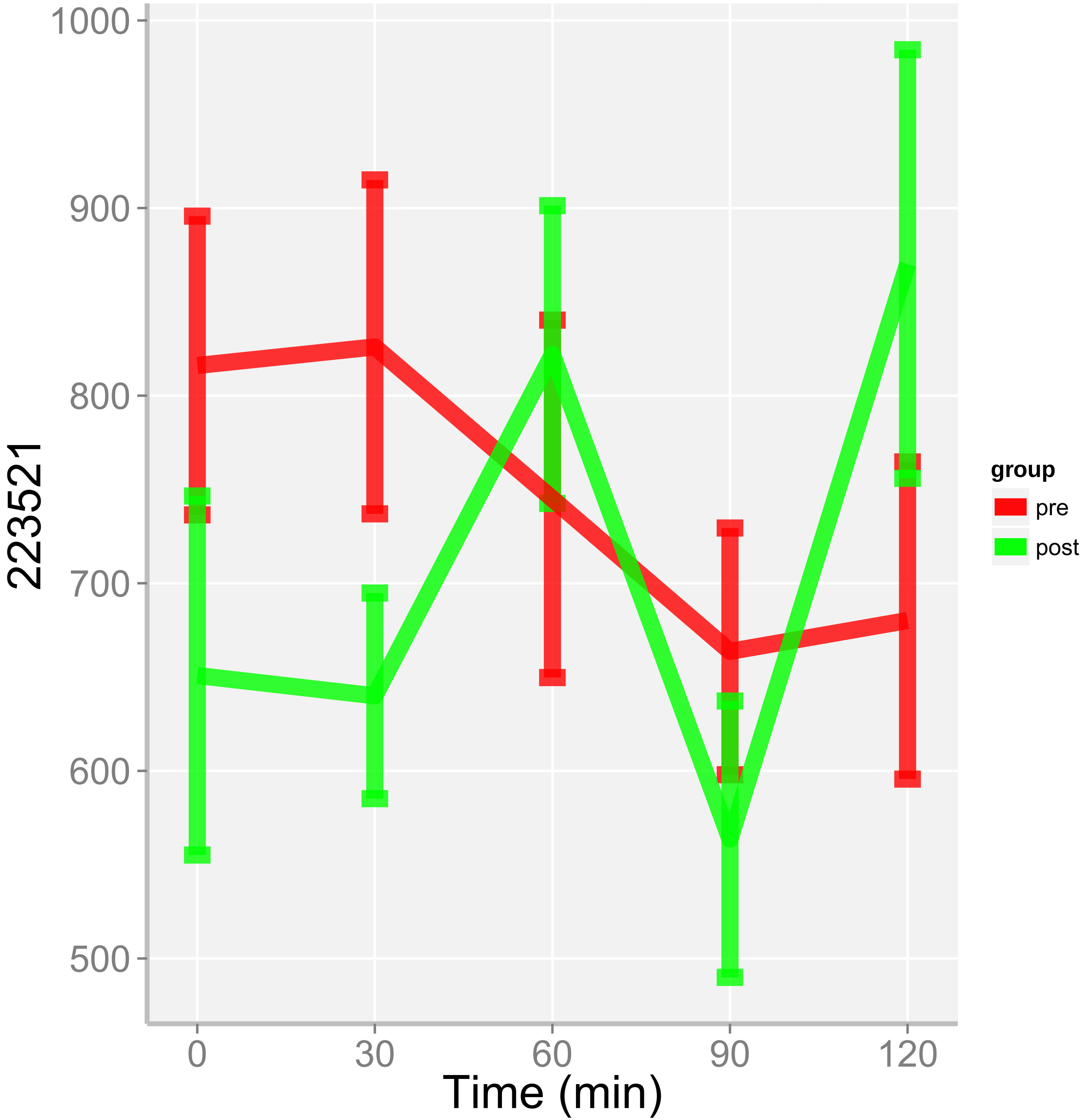

223521
1000
900
800
700
600
500
0
30
60
90
120
Time (min)
group
pre
post

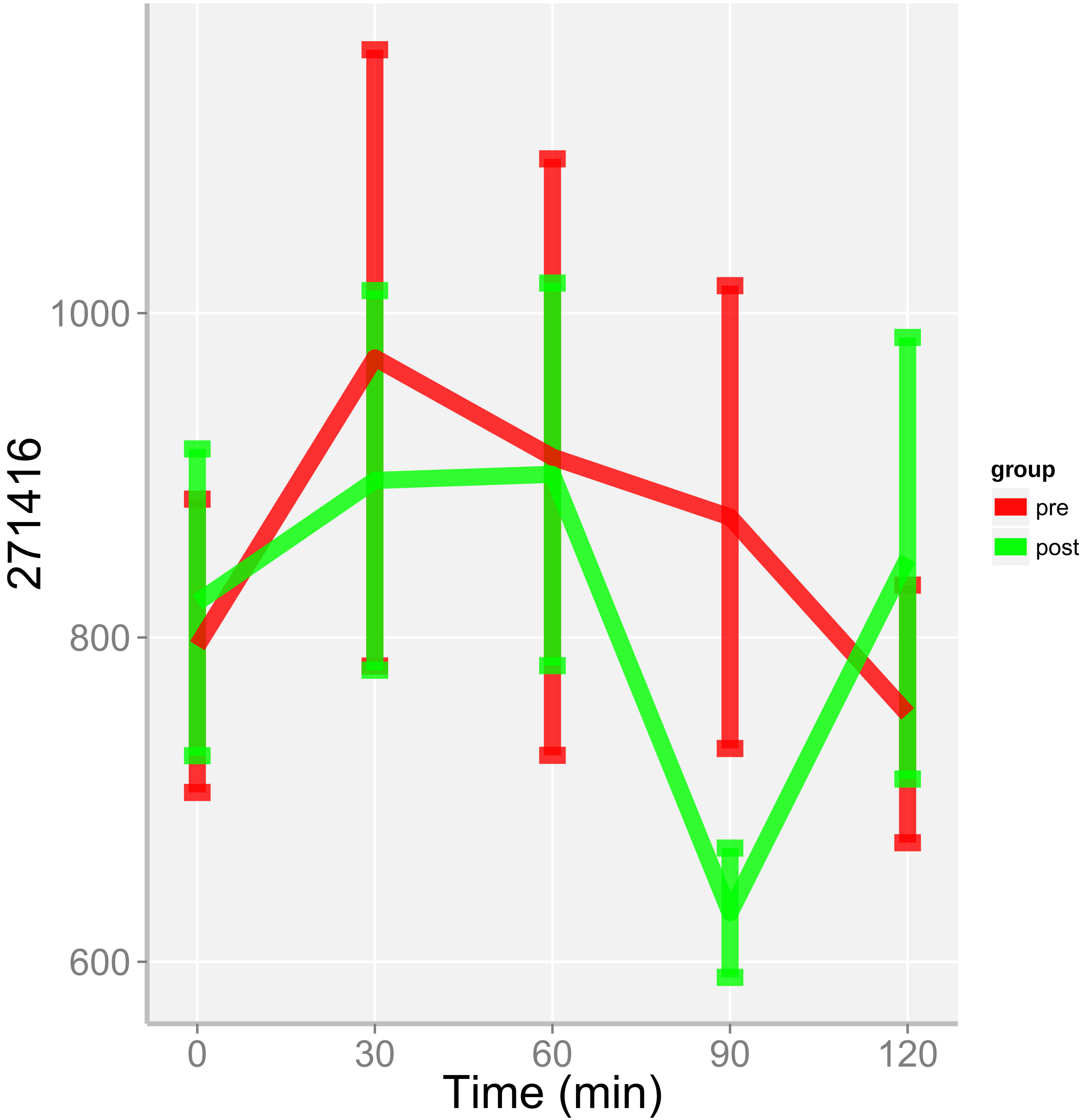

271416
1000
800
600
0
30
60
90
120
Time (min)
group
pre
post

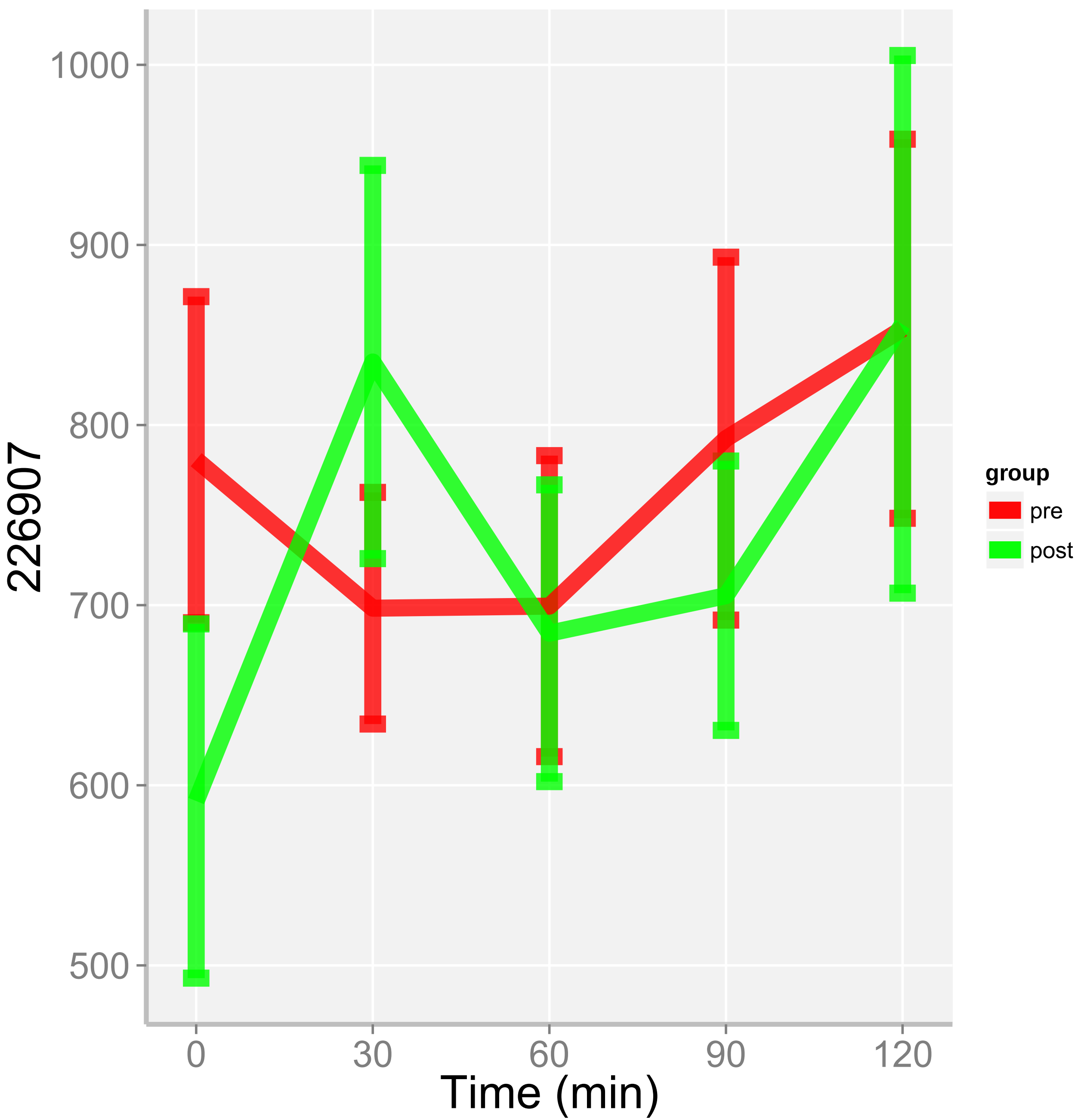

226907
1000
900
800
700
600
500
0
30
60
90
120
Time (min)
group
pre
post

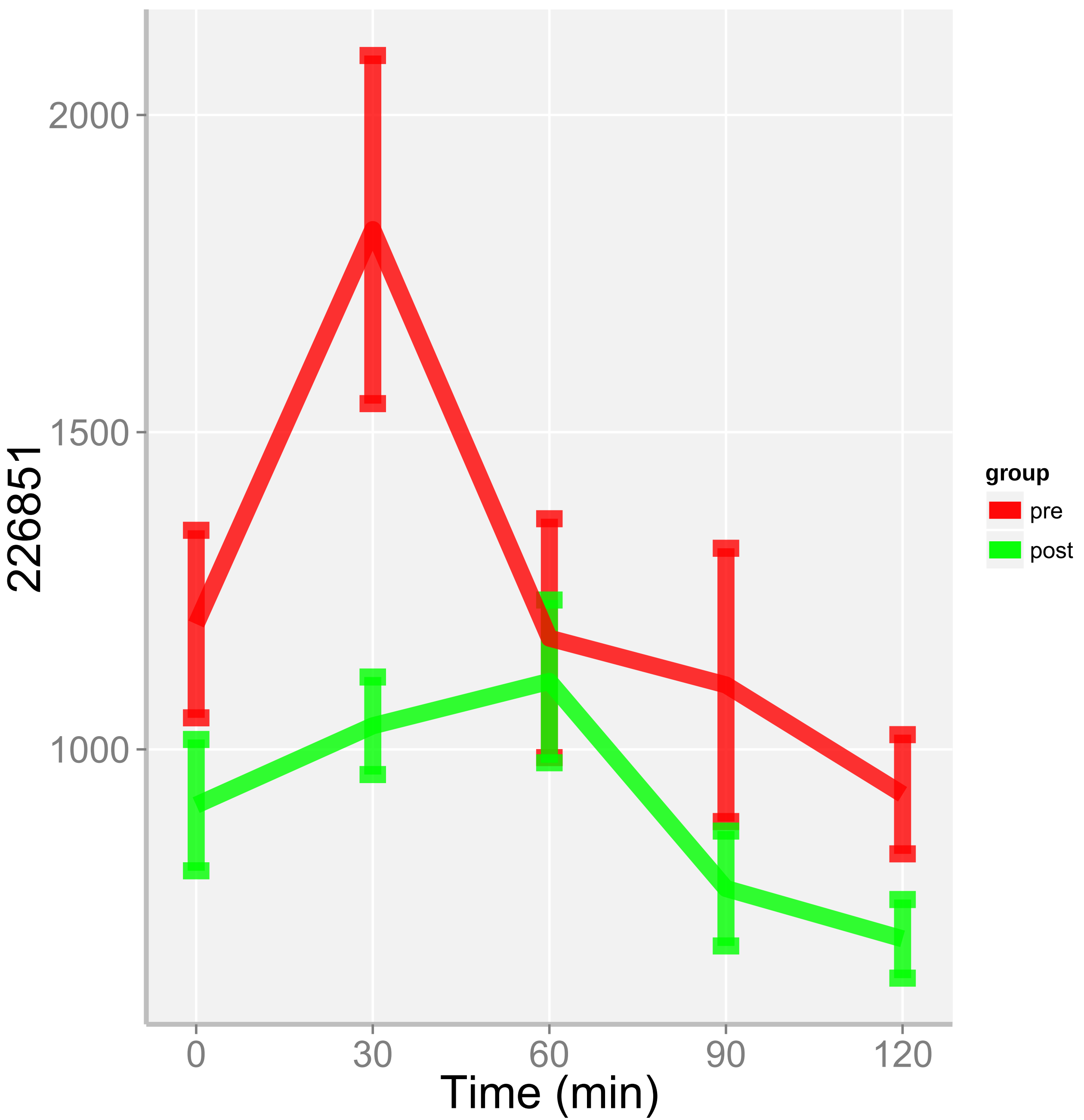

2000
1500
1000
226851
0
30
60
90
120
Time (min)
group
pre
post

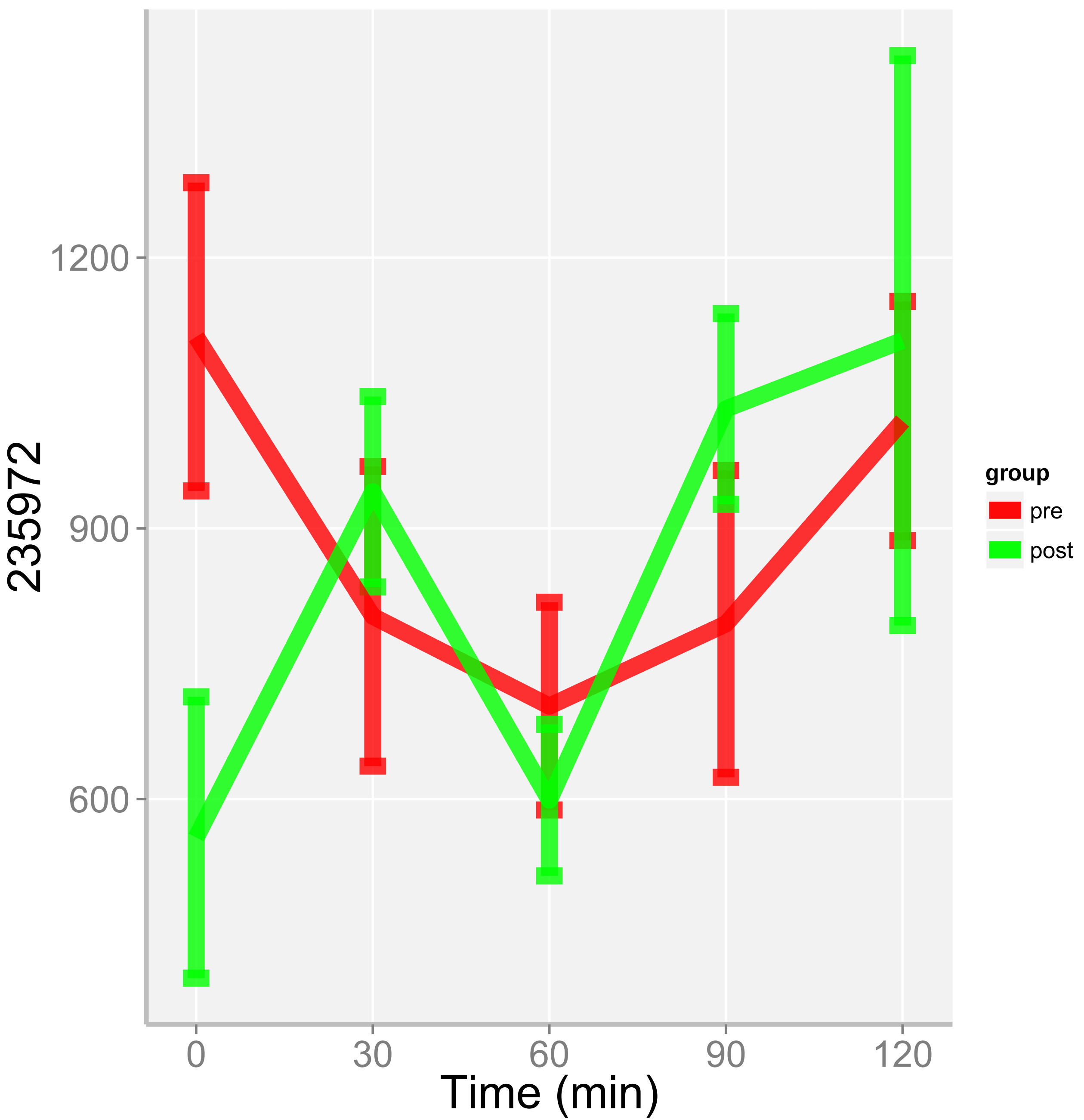

235972
1200
900
600
0
30
60
90
120
Time (min)
group
pre
post

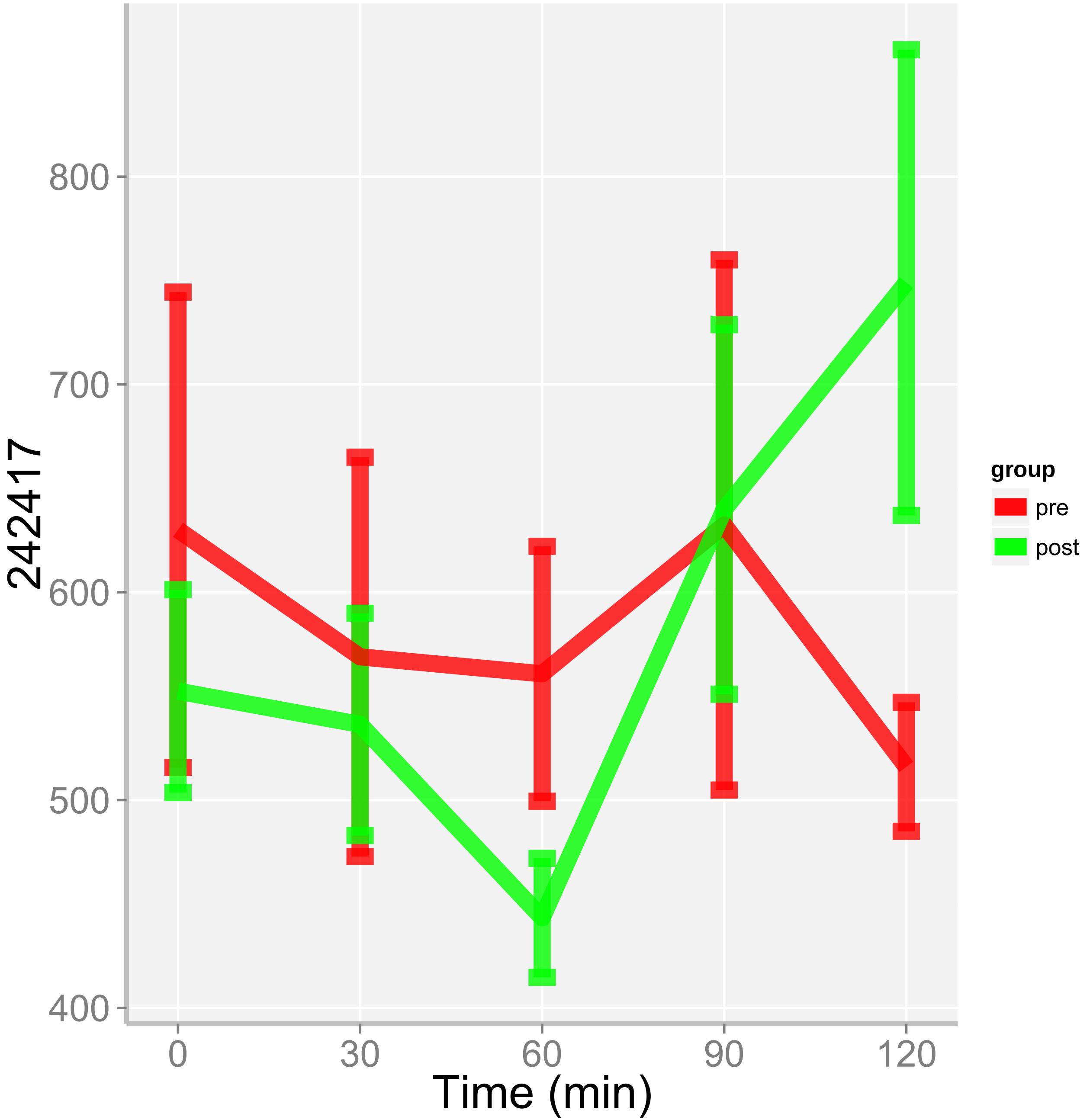

242417
800
700
600
500
400
0
30
60
90
120
Time (min)
group
pre
post

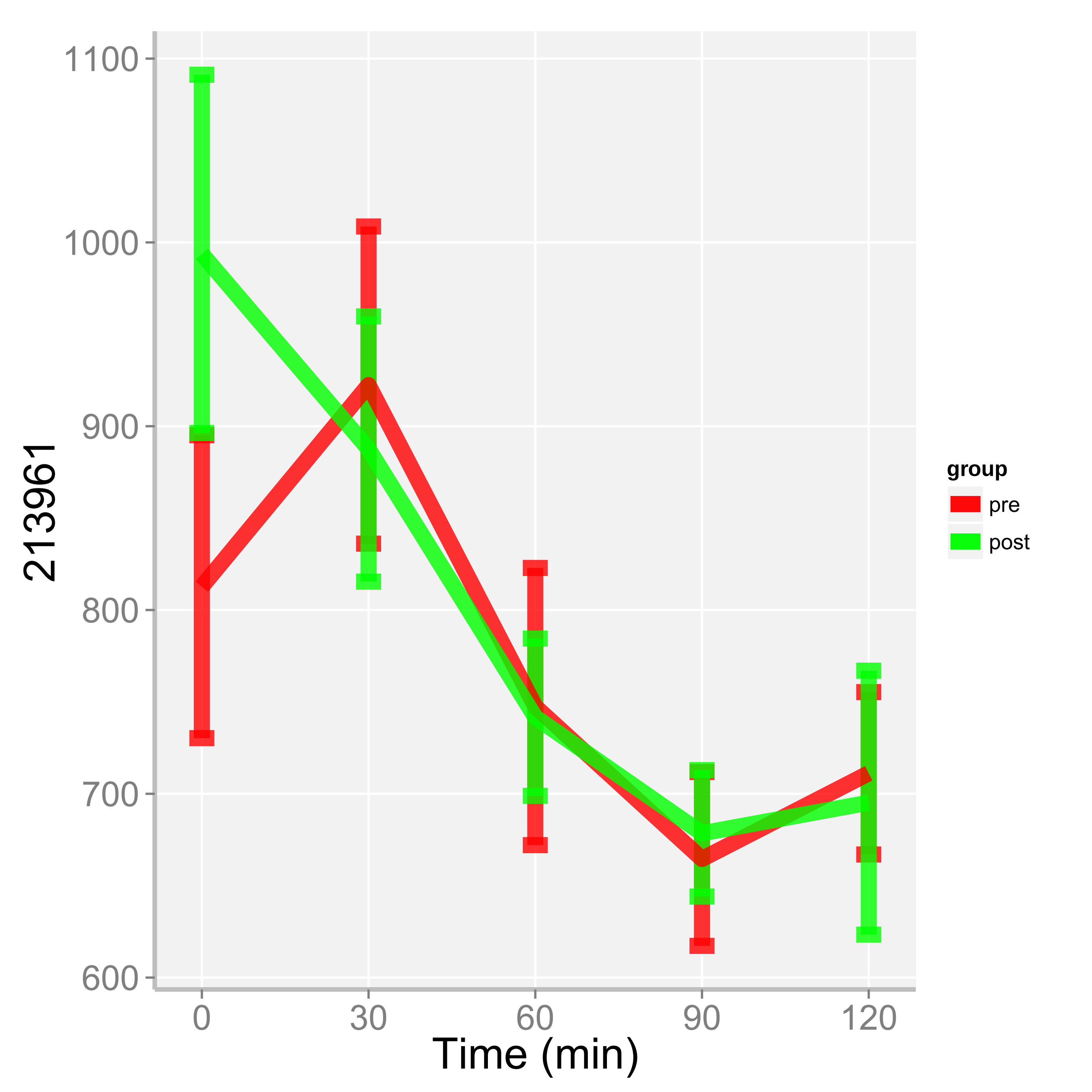

1100
1000
900
800
700
600
213961
0
30
60
90
120
Time (min)
group
pre
post

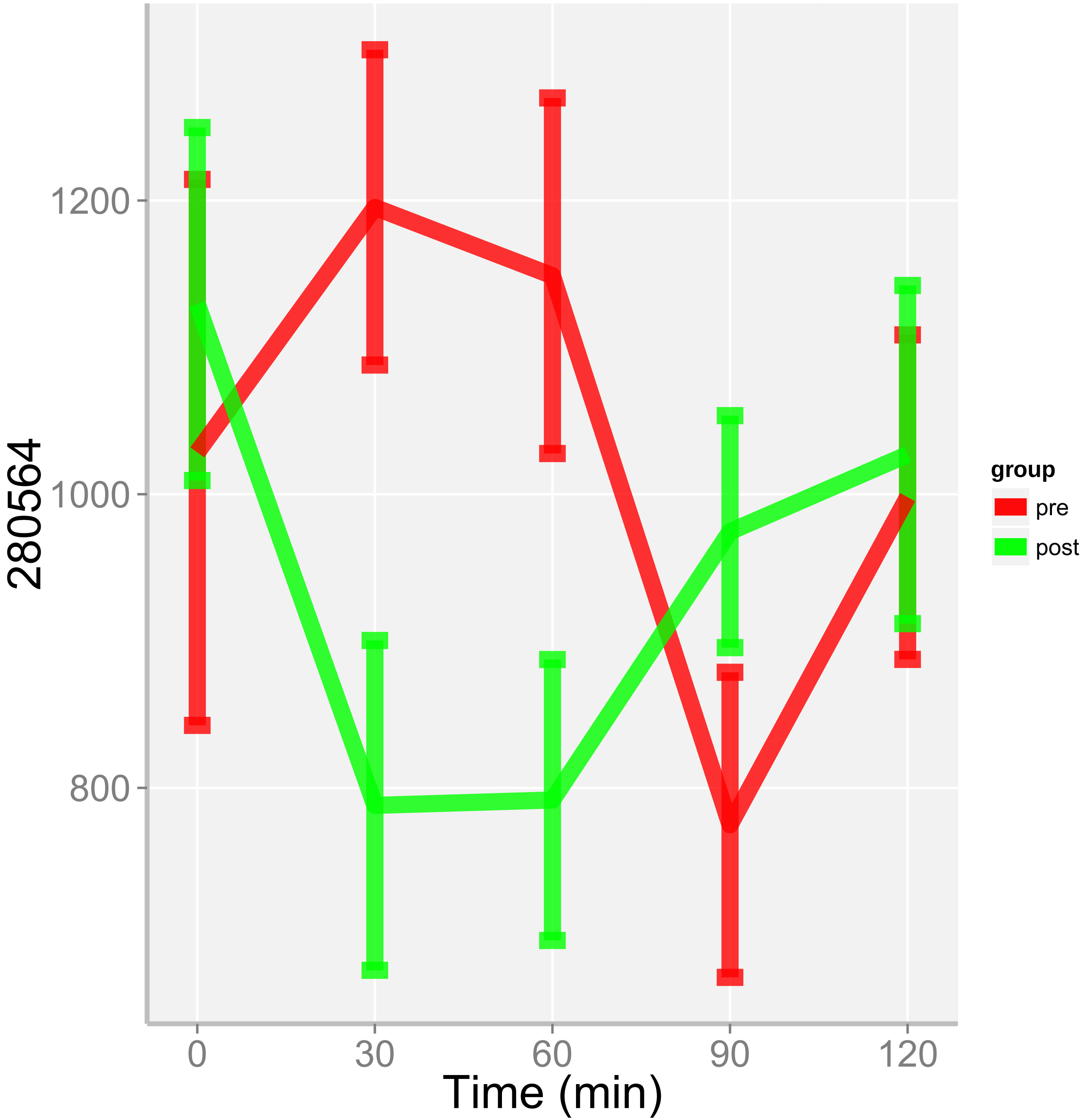

280564
1200
1000
800
0
30
60
90
120
Time (min)
group
pre
post

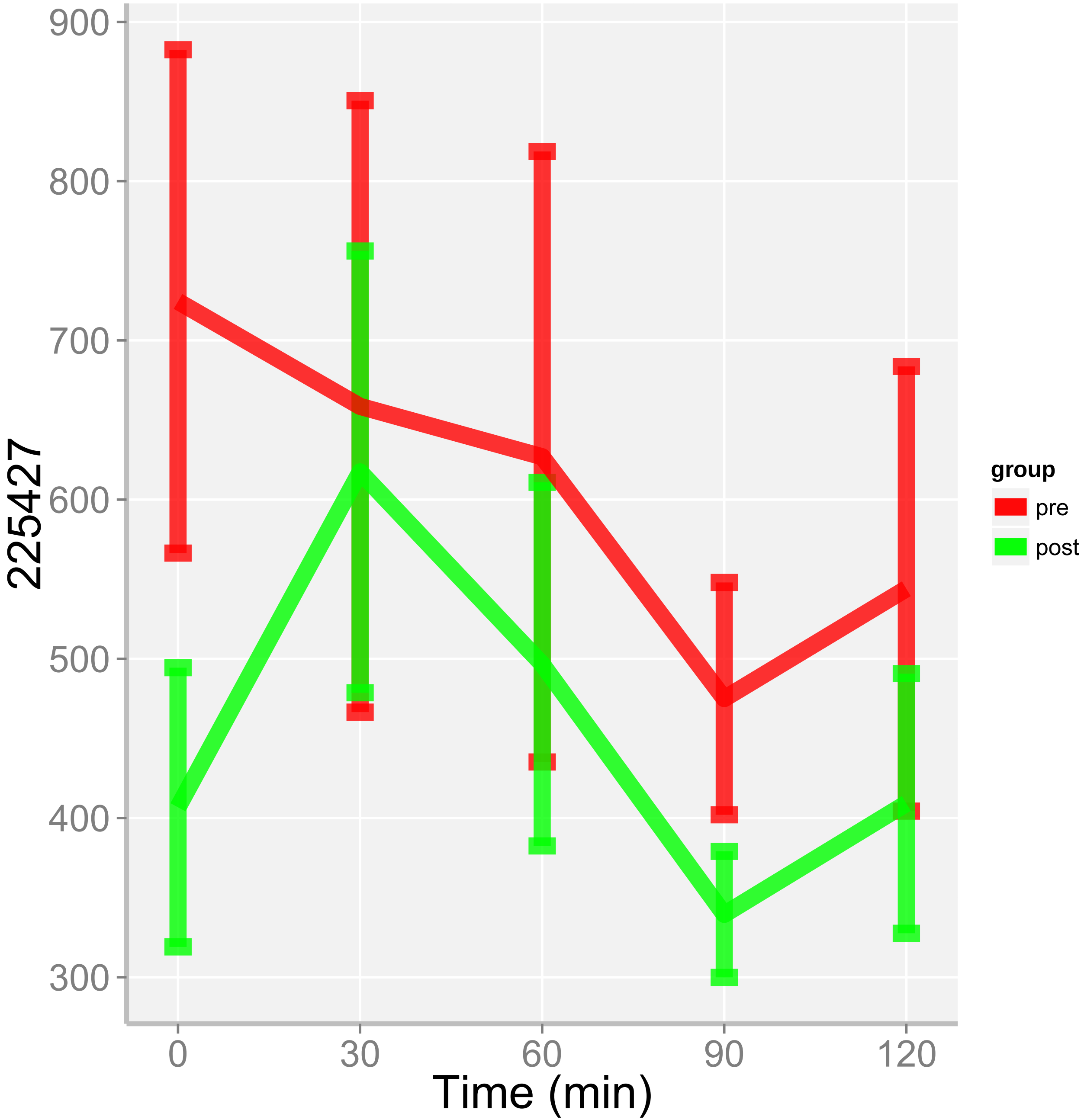

900
800
700
600
500
400
300
225427
0
30
60
90
120
Time (min)
group
pre
post

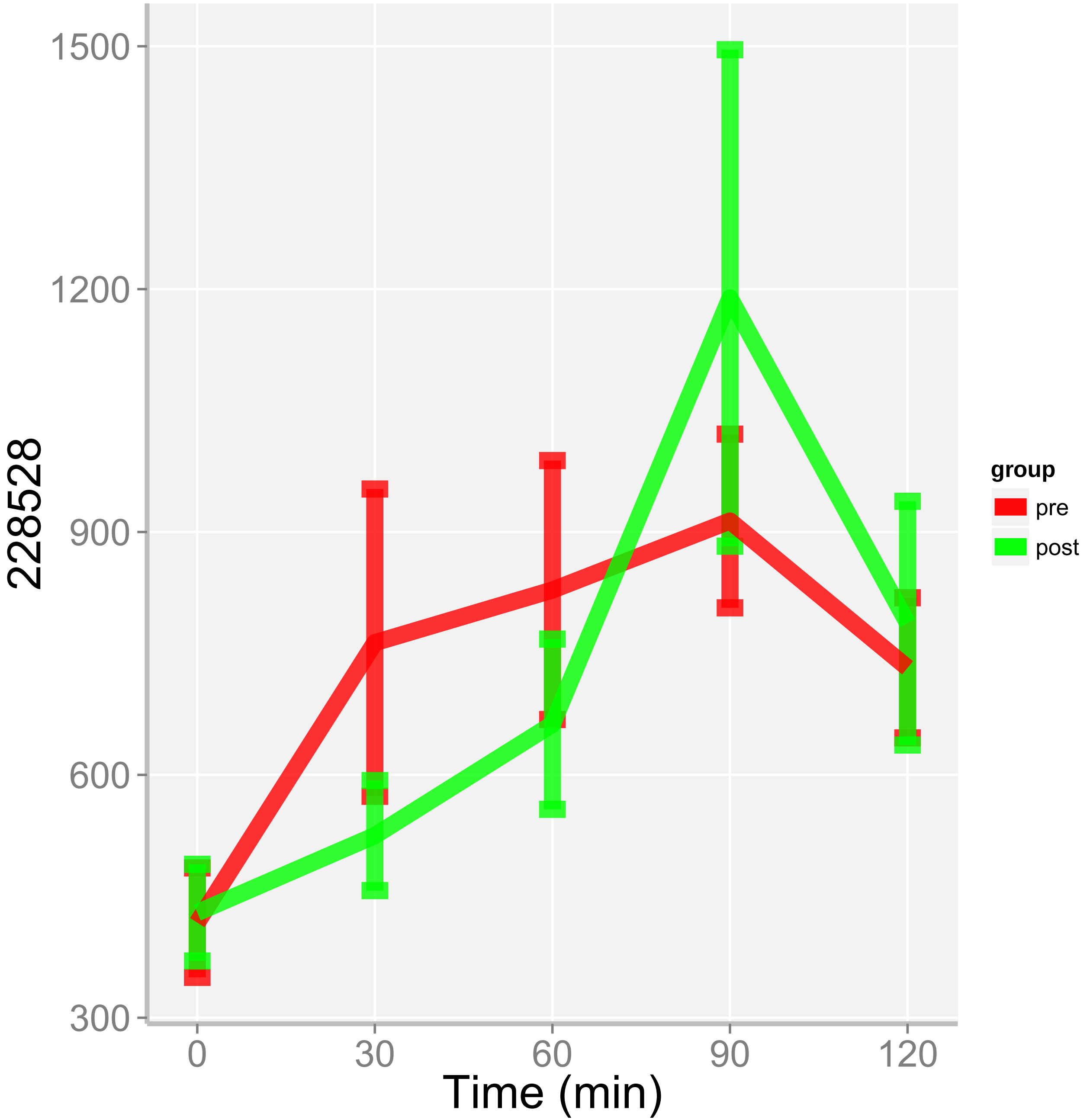

228528
1500
1200
900
600
300
0
30
60
90
120
Time (min)
group
pre
post

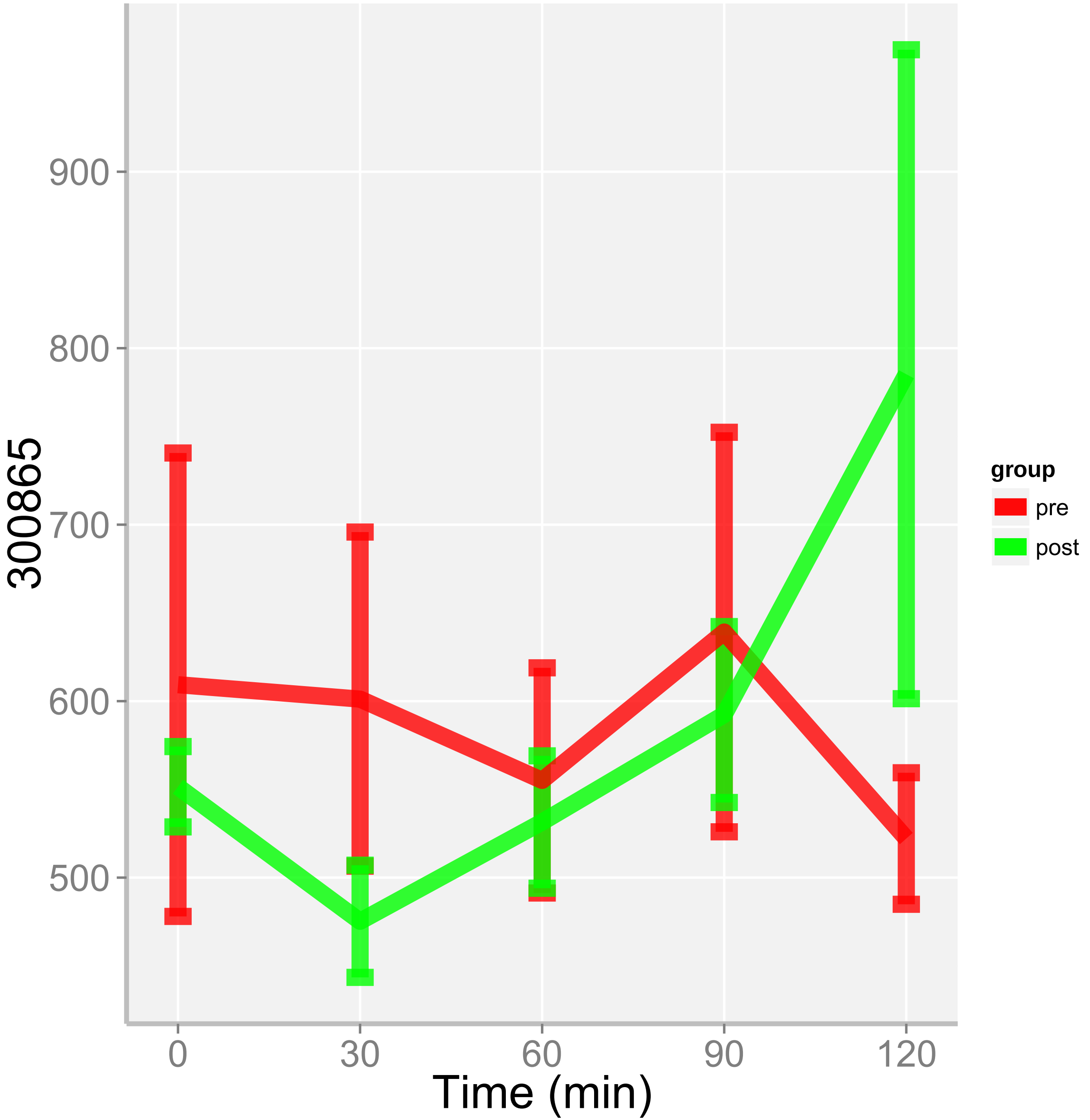
300865
900
800
700
600
500
0
30
60
90
120
Time (min)
group
pre
post

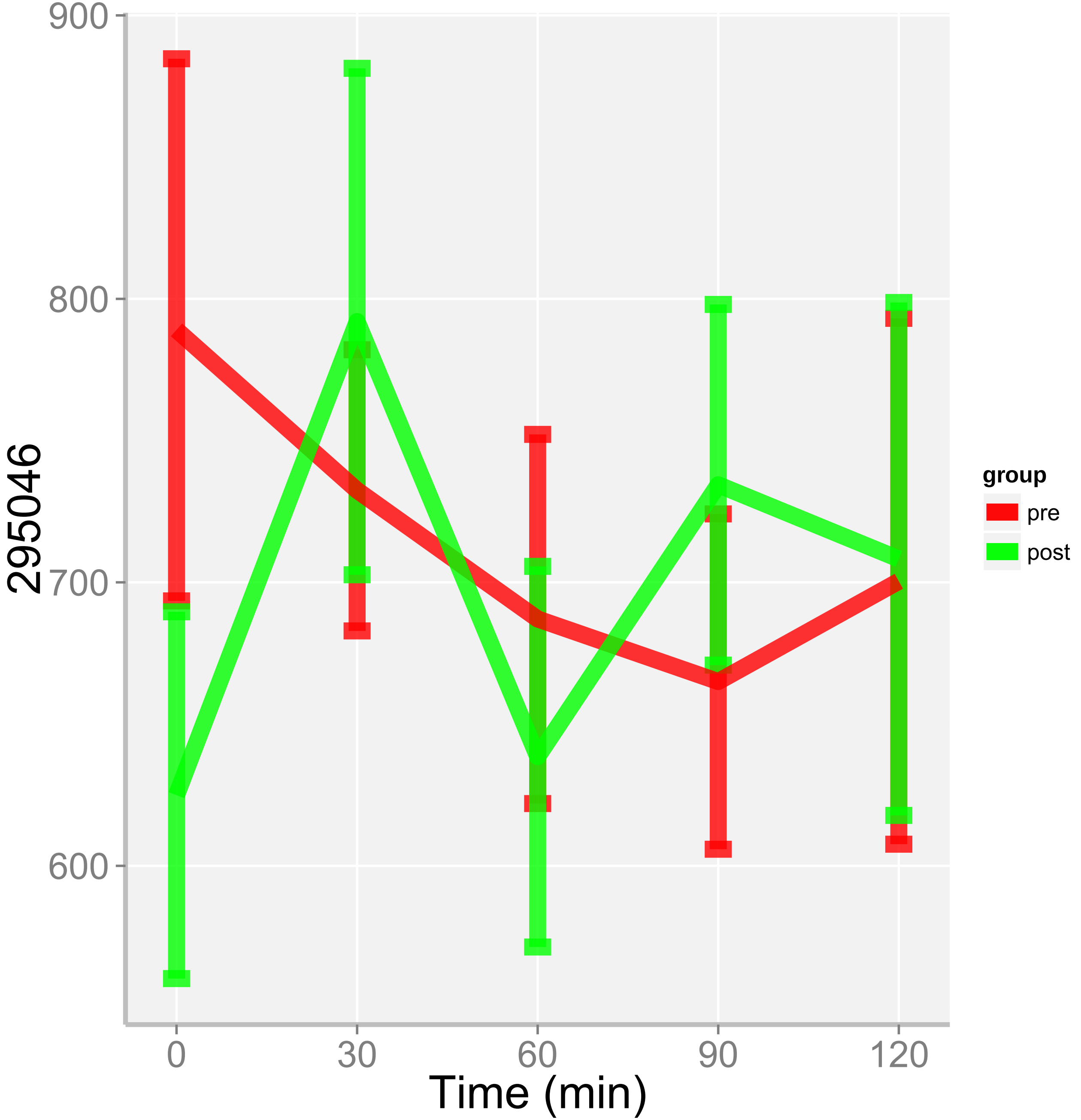
900
800
700
600
295046
0
30
60
90
120
Time (min)
group
pre
post

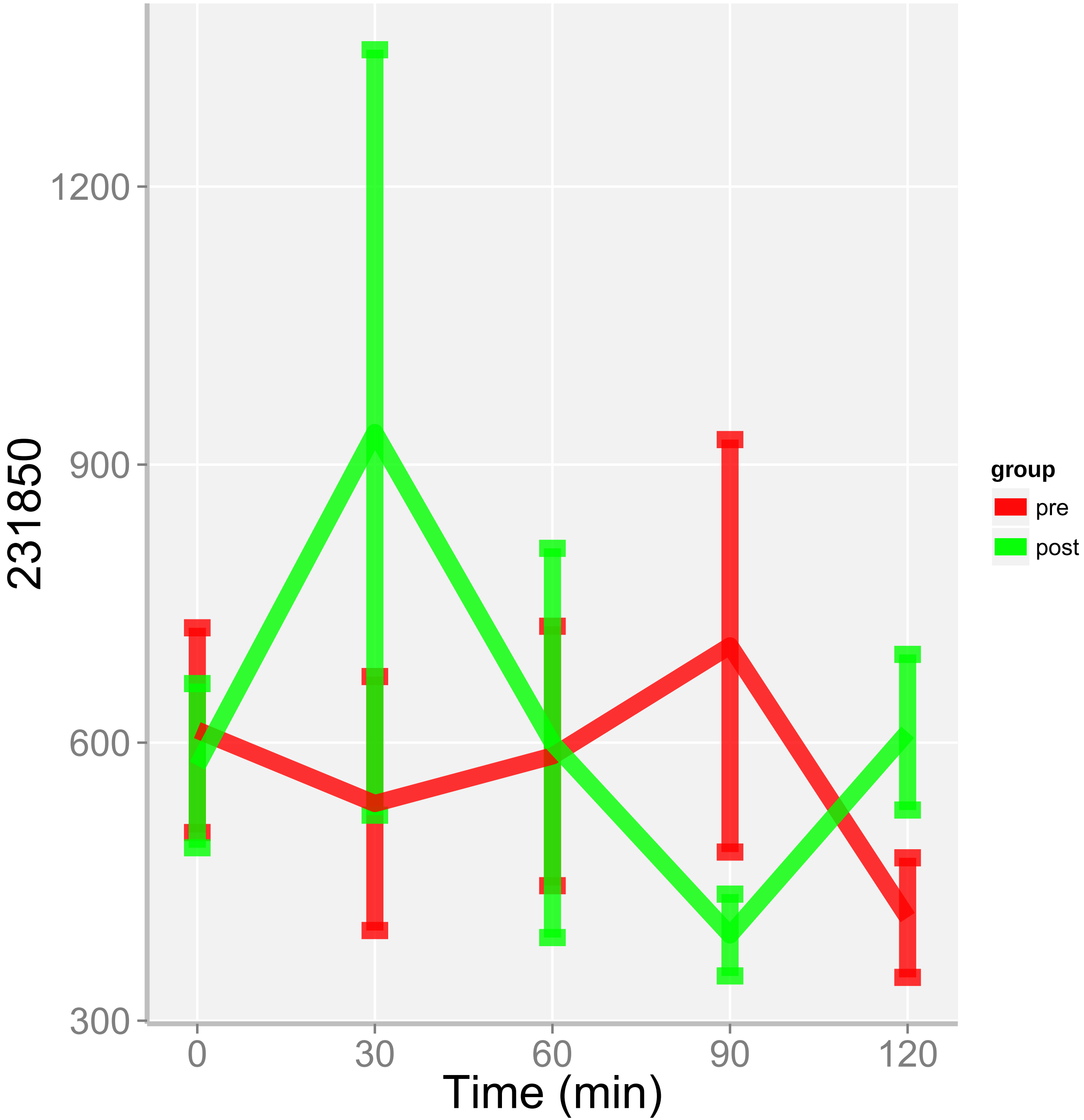

231850
1200
900
600
300
0
30
60
90
120
Time (min)
group
pre
post

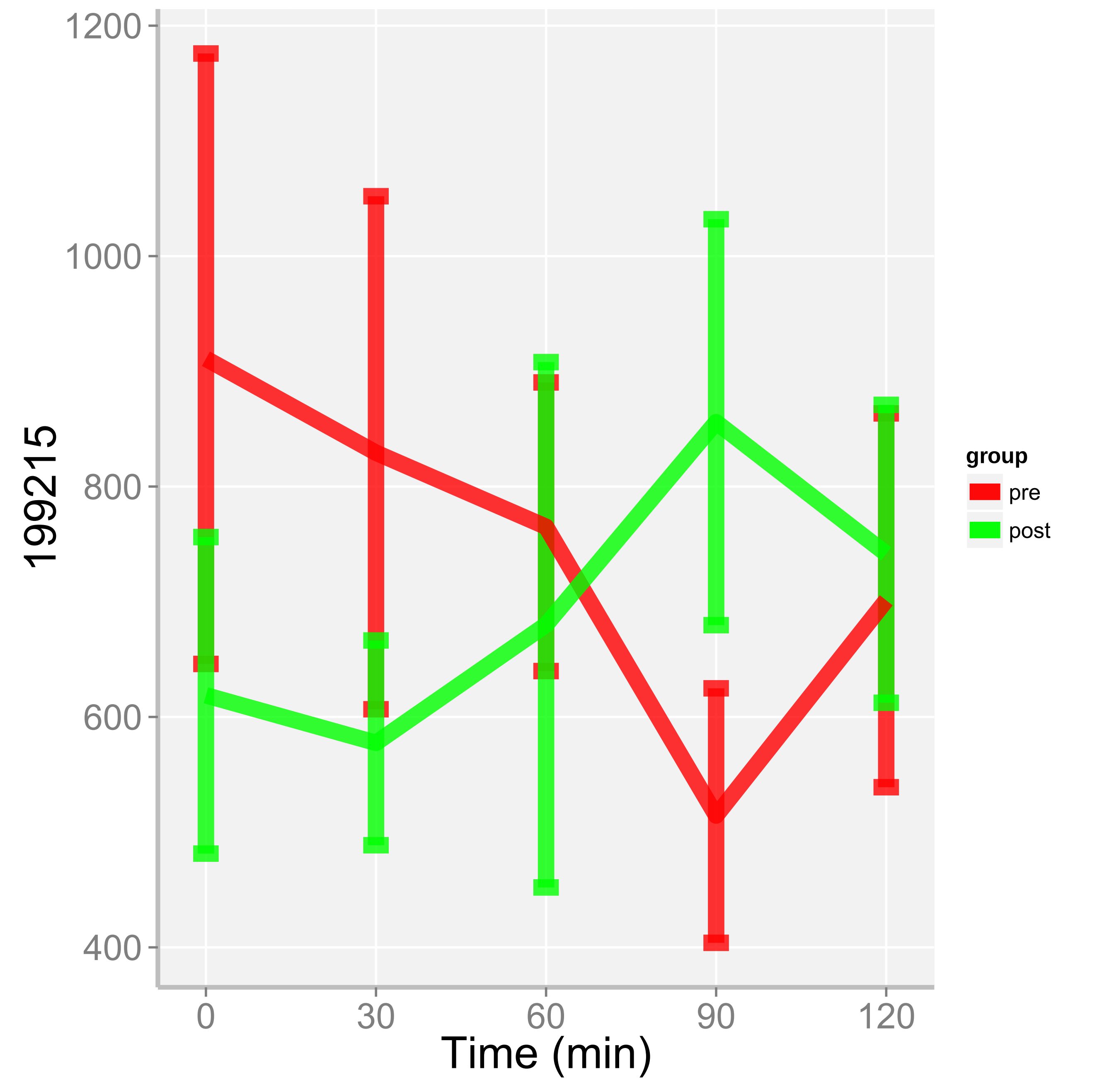

1200
1000
800
600
400
199215
0
30
60
90
120
Time (min)
group
pre
post

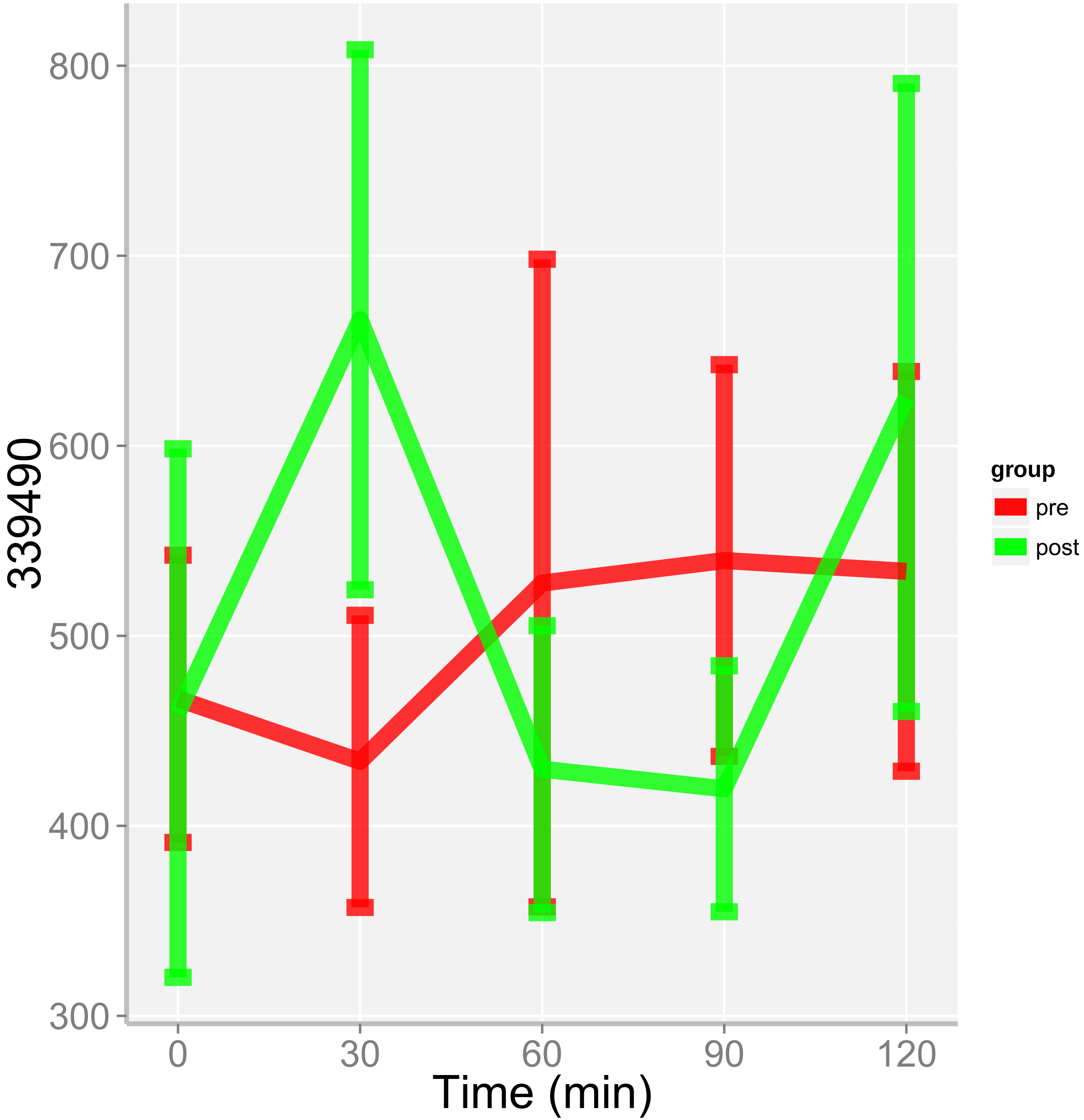

800
700
600
500
400
300
339490
0
30
60
90
120
Time (min)
group
pre
post

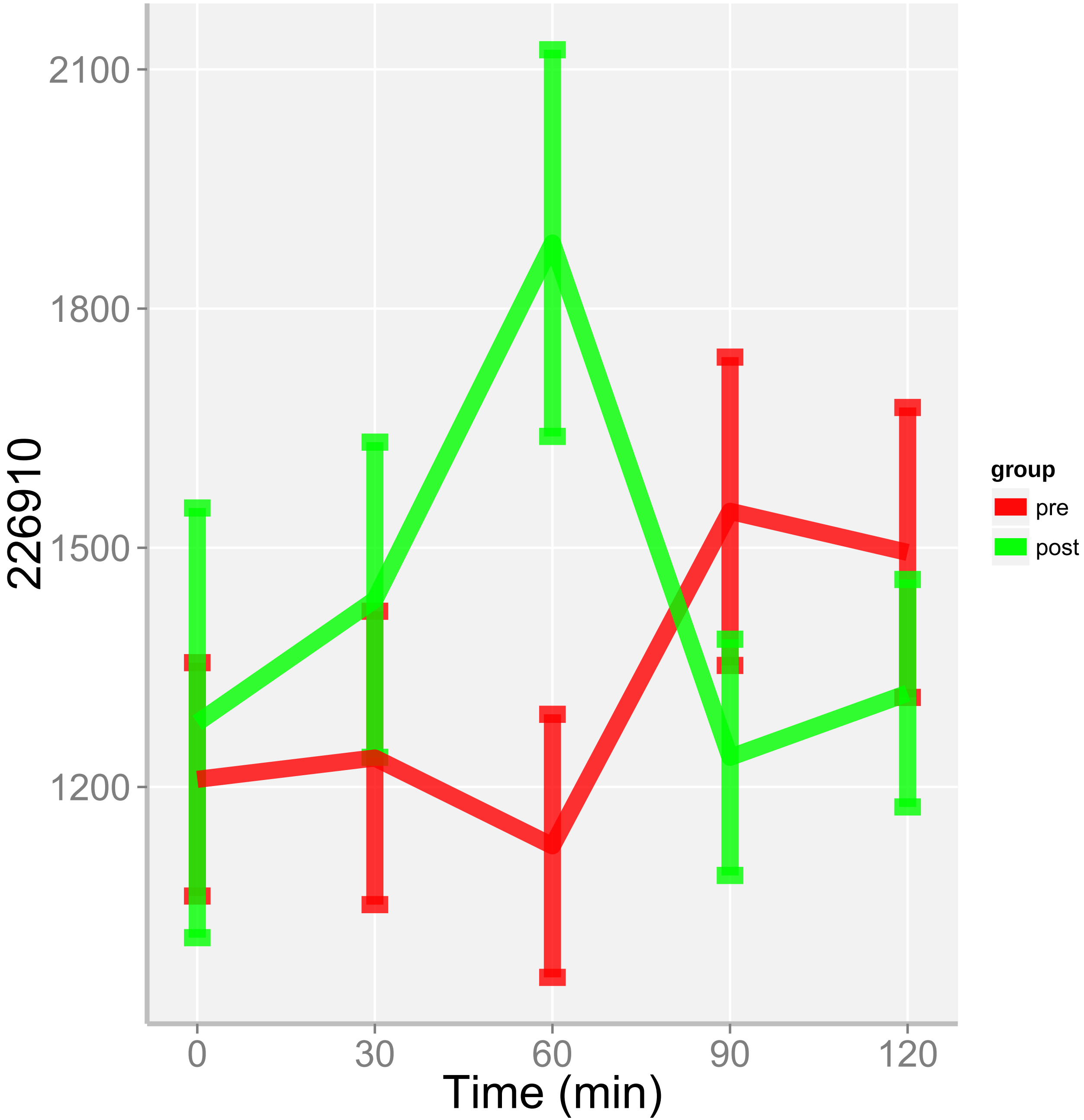
2100
1800
1500
1200
226910
0
30
60
90
120
Time (min)
group
pre
post

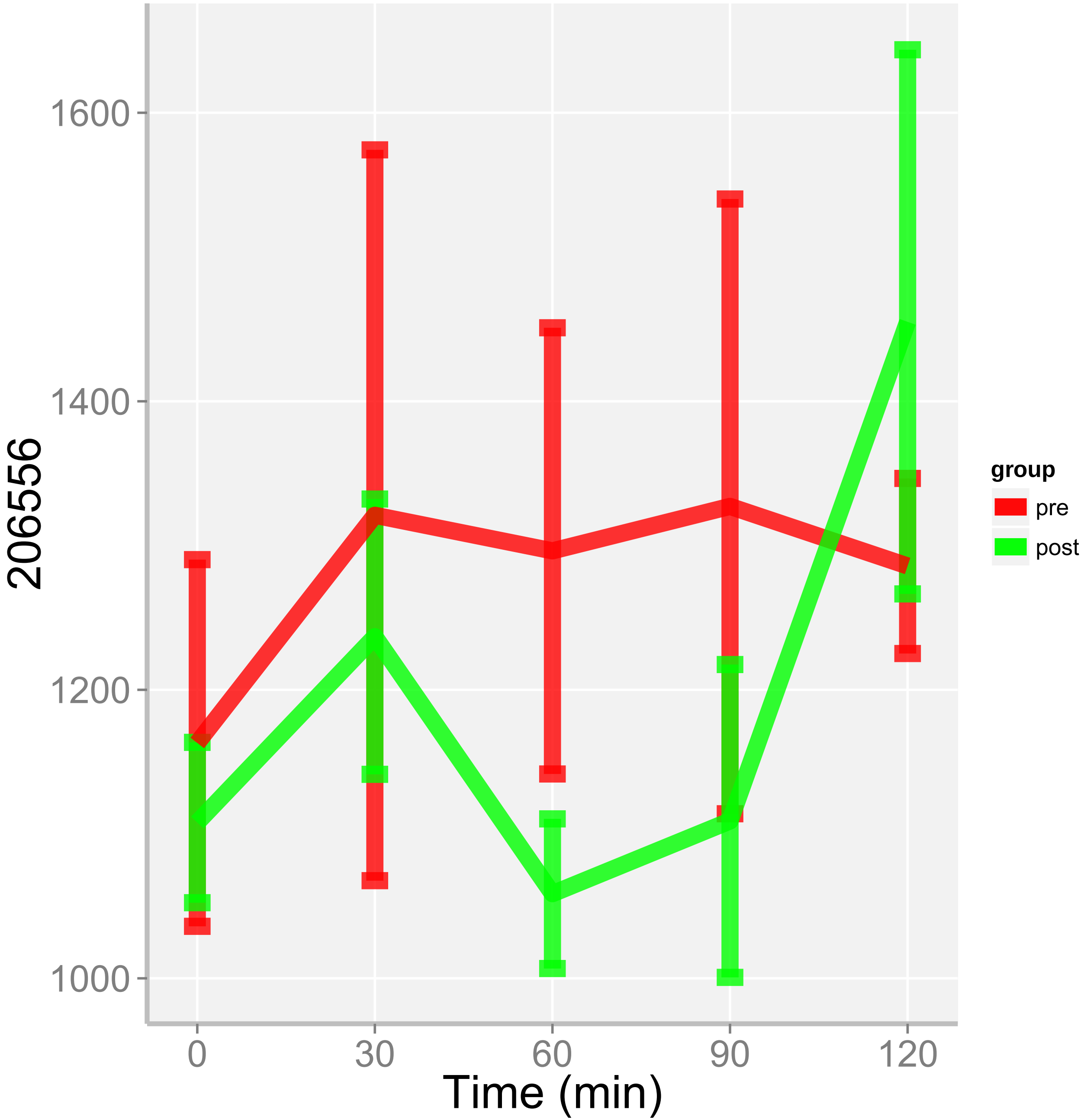

206556
1600
1400
1200
1000
0
30
60
90
120
Time (min)
group
pre
post

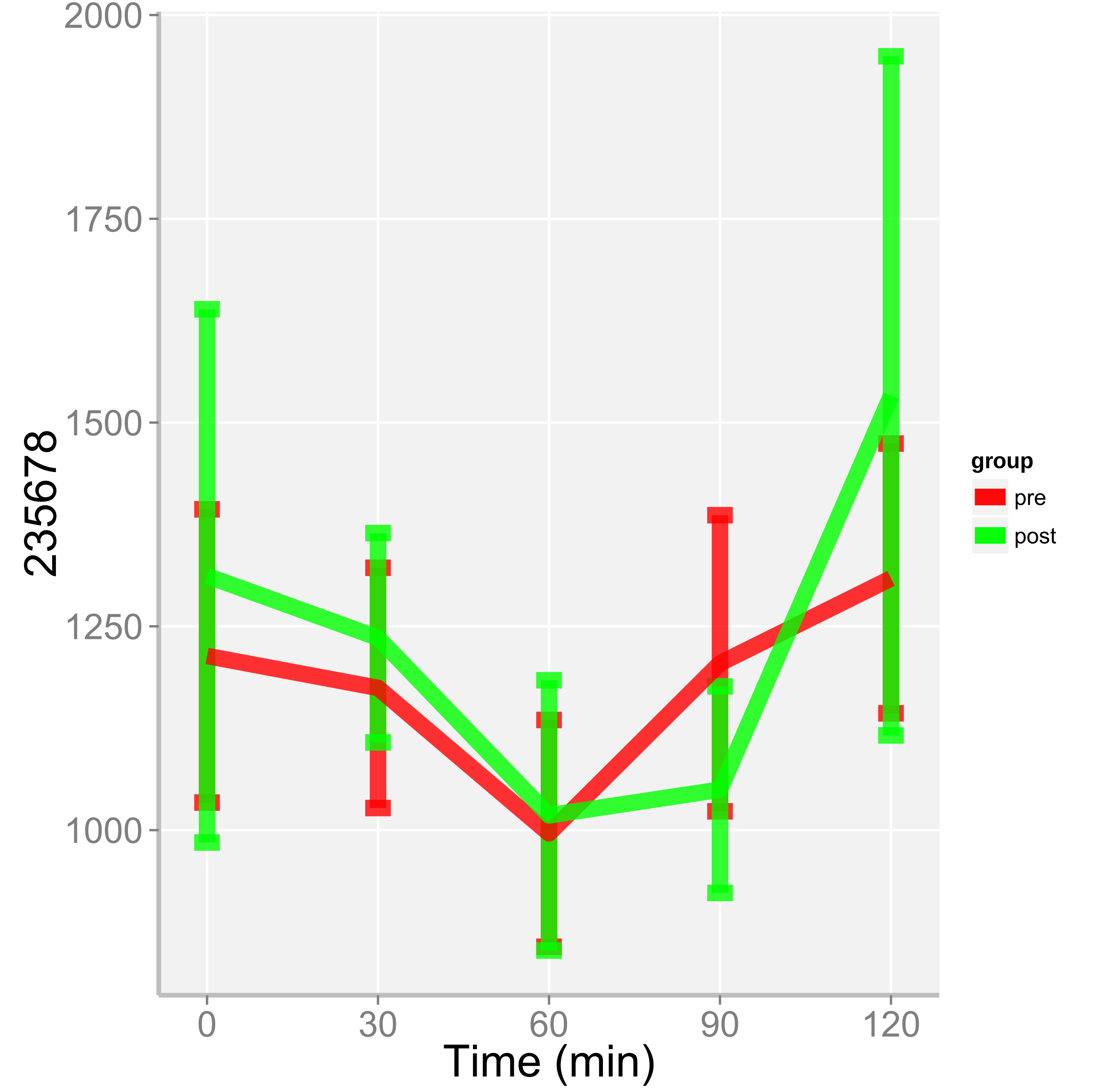

2000
1750
1500
1250
1000
235678
0
30
60
90
120
Time (min)
group
pre
post

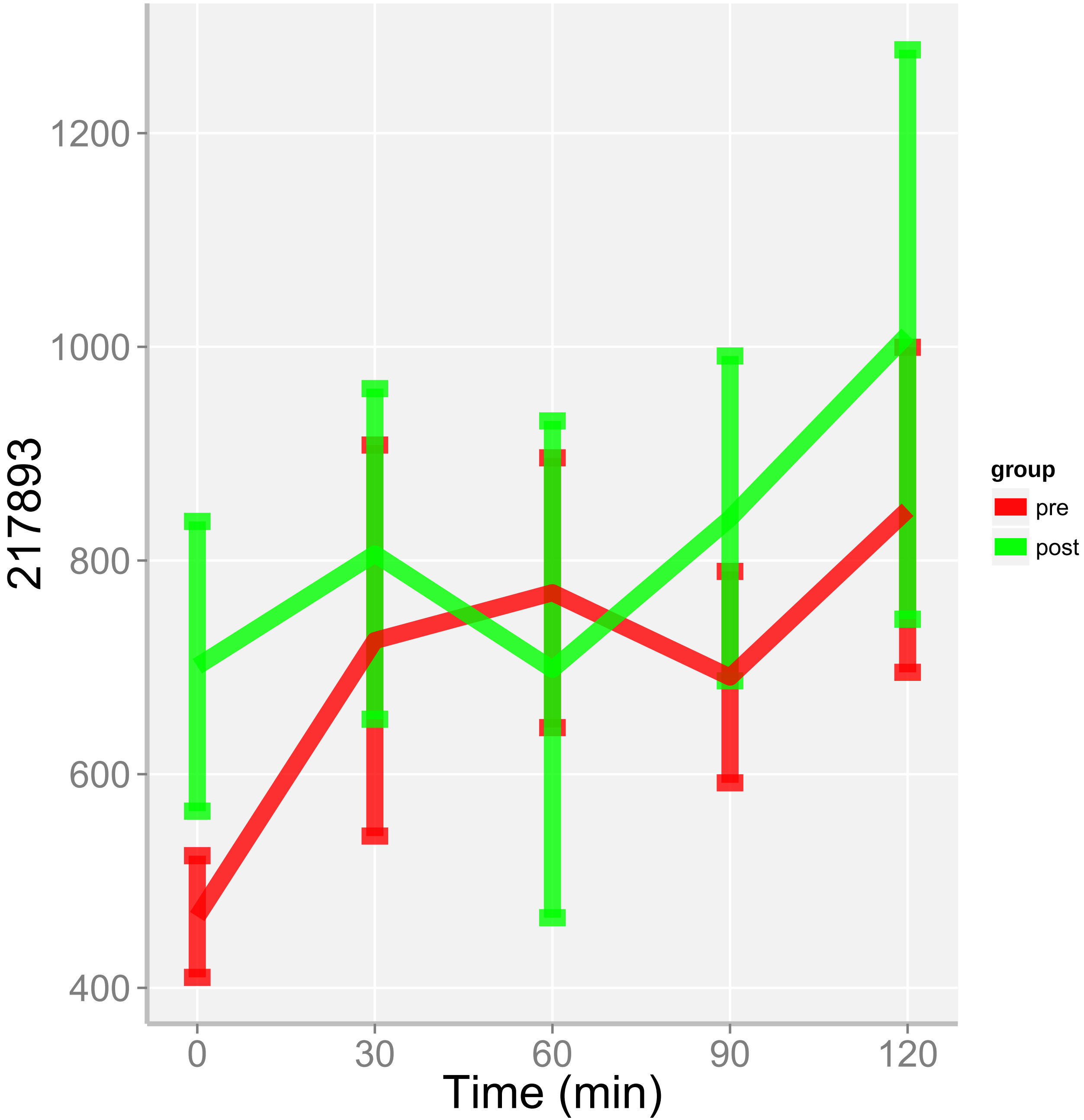
217893
1200
1000
800
600
400
0
30
60
90
120
Time (min)
group
pre
post

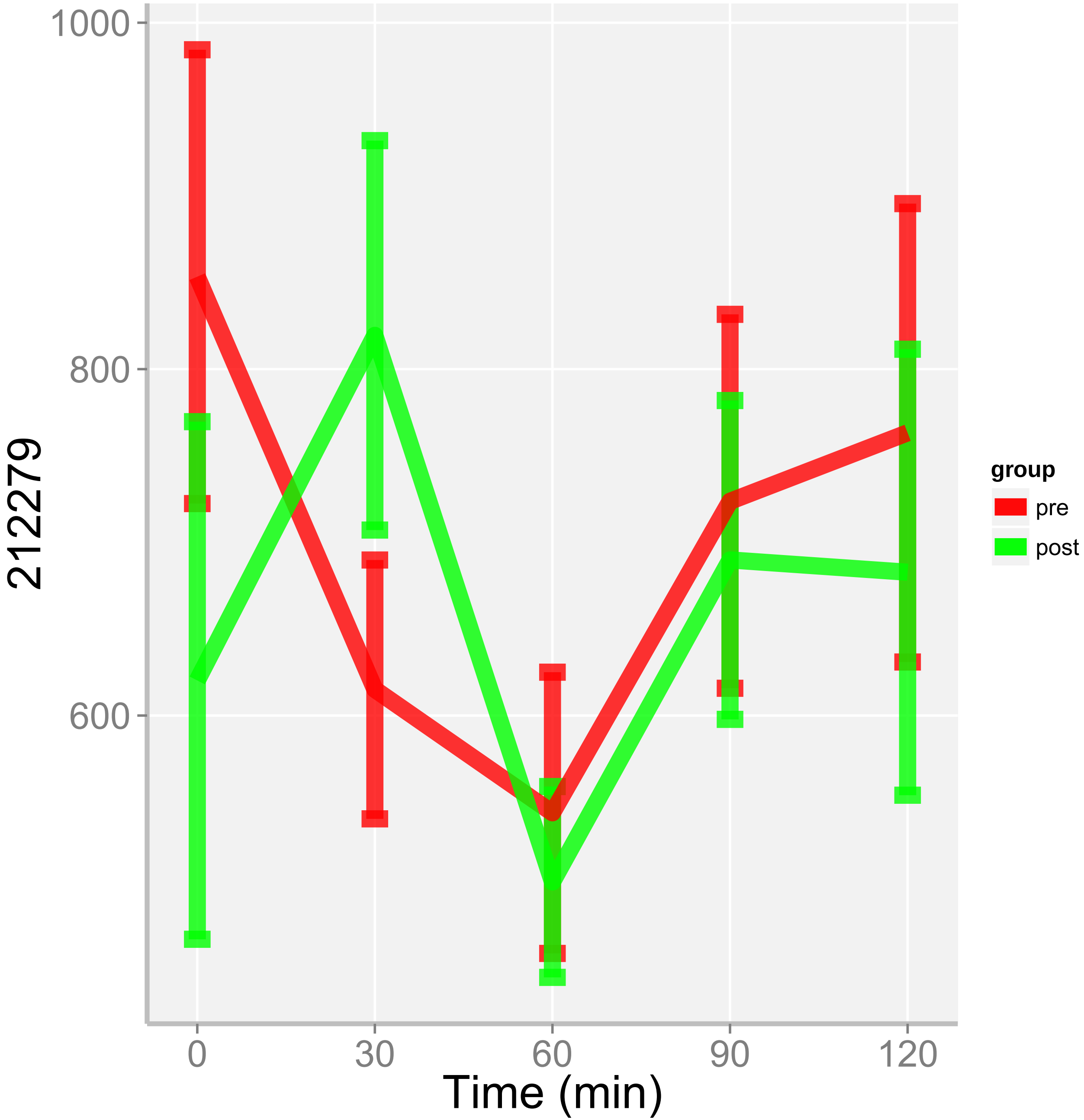

1000
800
600
212279
0
30
60
90
120
Time (min)
group
pre
post

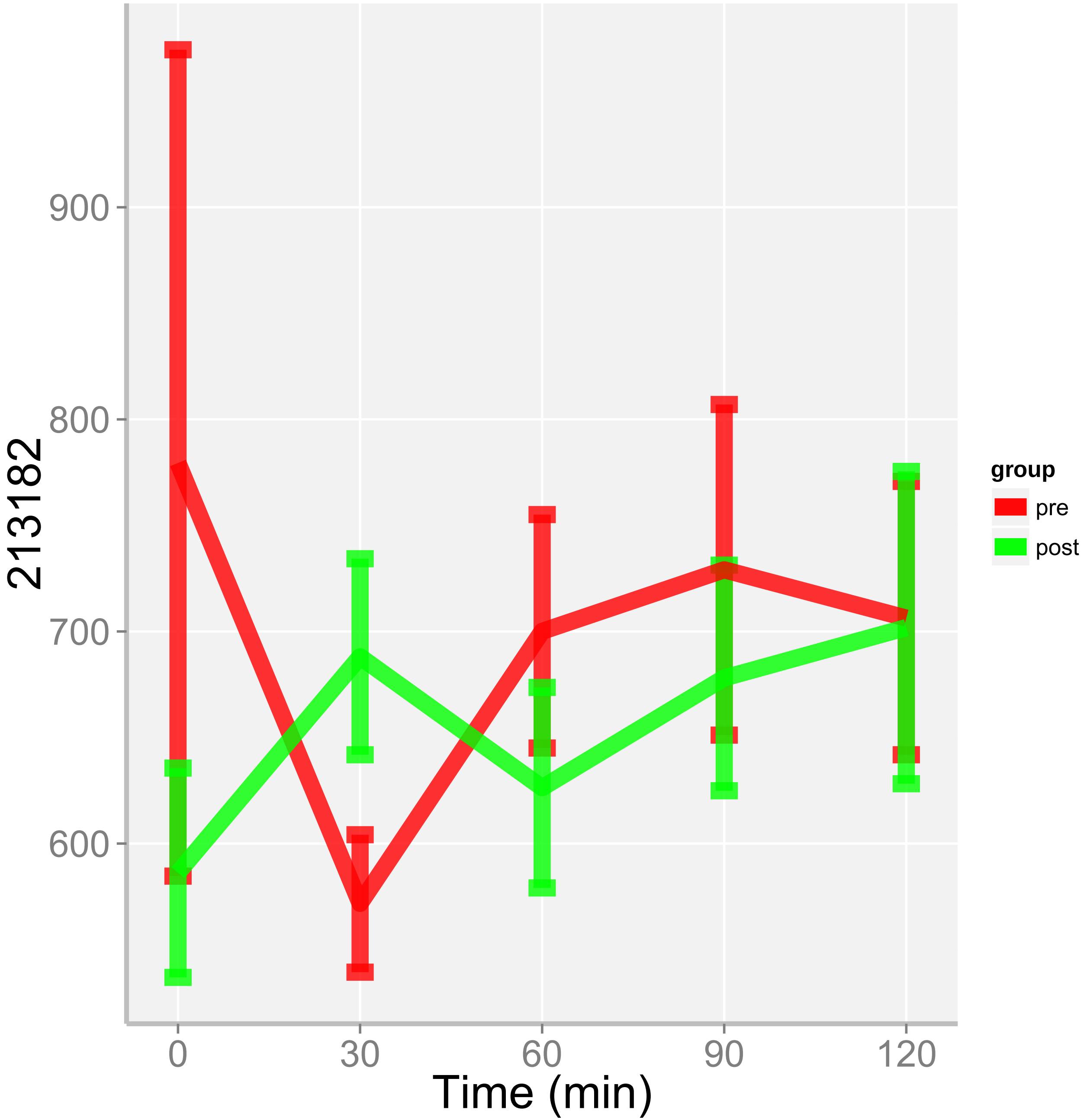

900
800
700
600
213182
0
30
60
90
120
Time (min)
group
pre
post

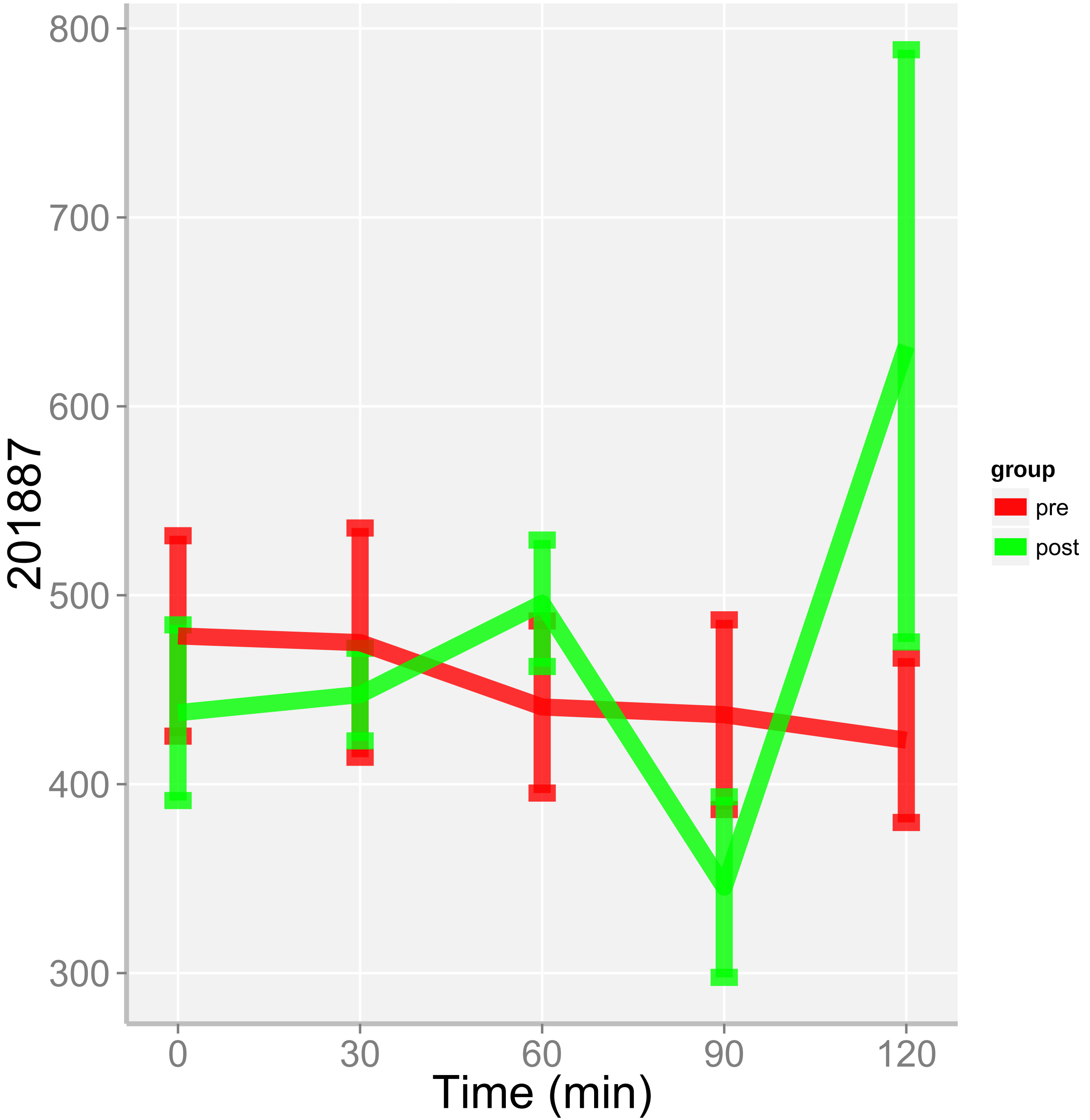

201887
800
700
600
500
400
300
0
30
60
90
120
Time (min)
group
pre
post

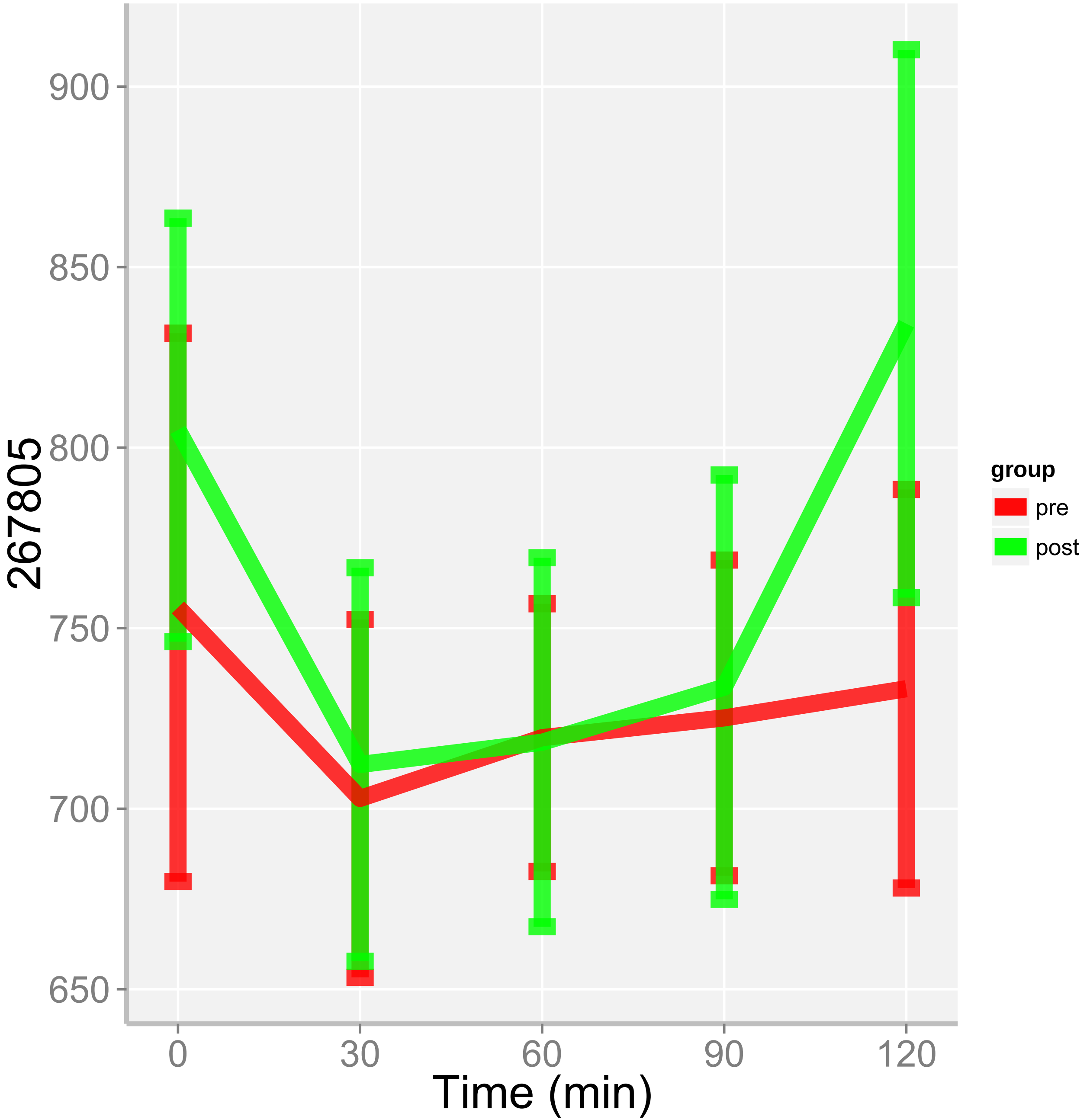
267805
900
850
800
750
700
650
0
30
60
90
120
Time (min)
group
pre
post

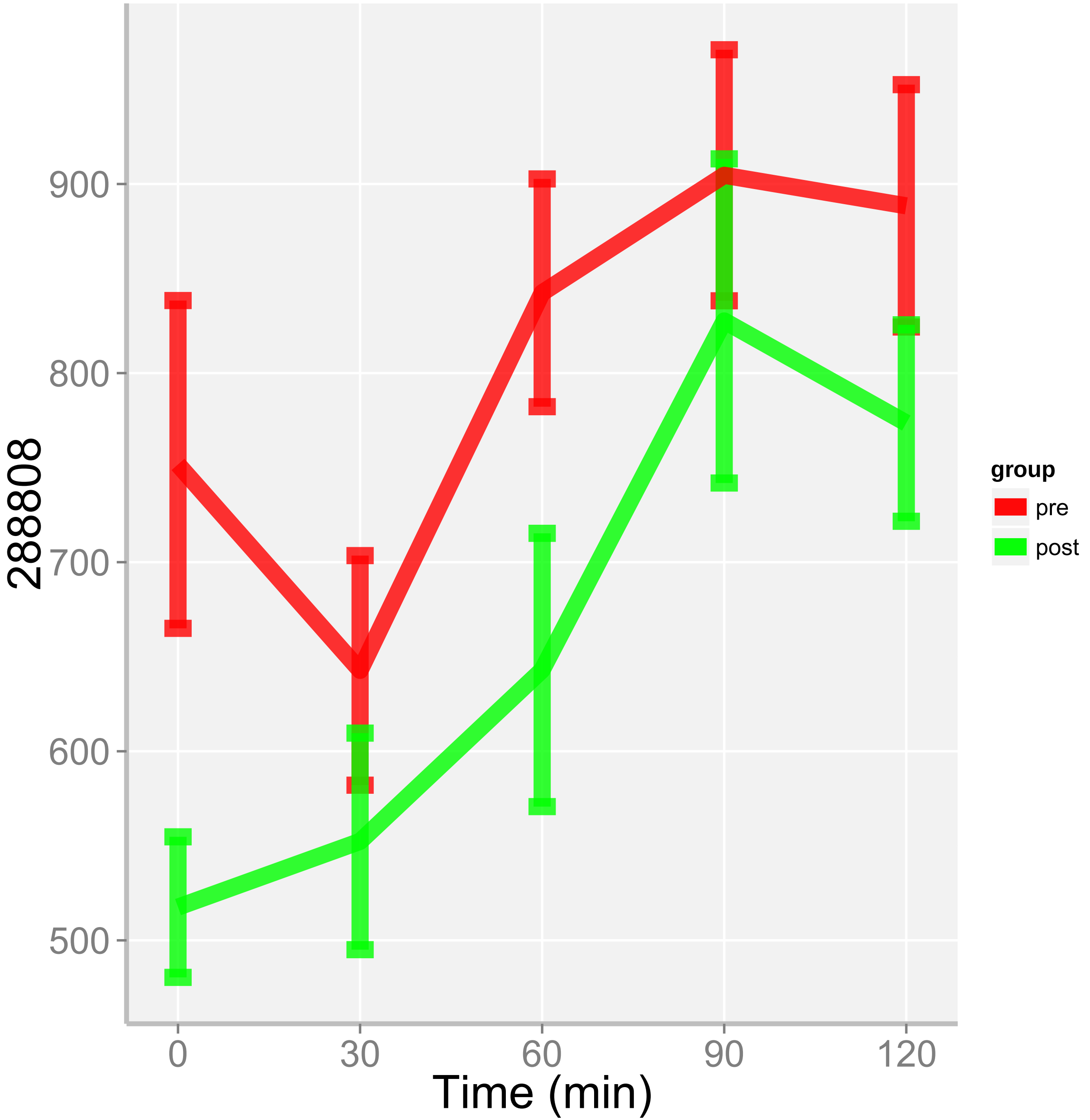

288808
900
800
700
600
500
0
30
60
90
120
Time (min)
group
pre
post

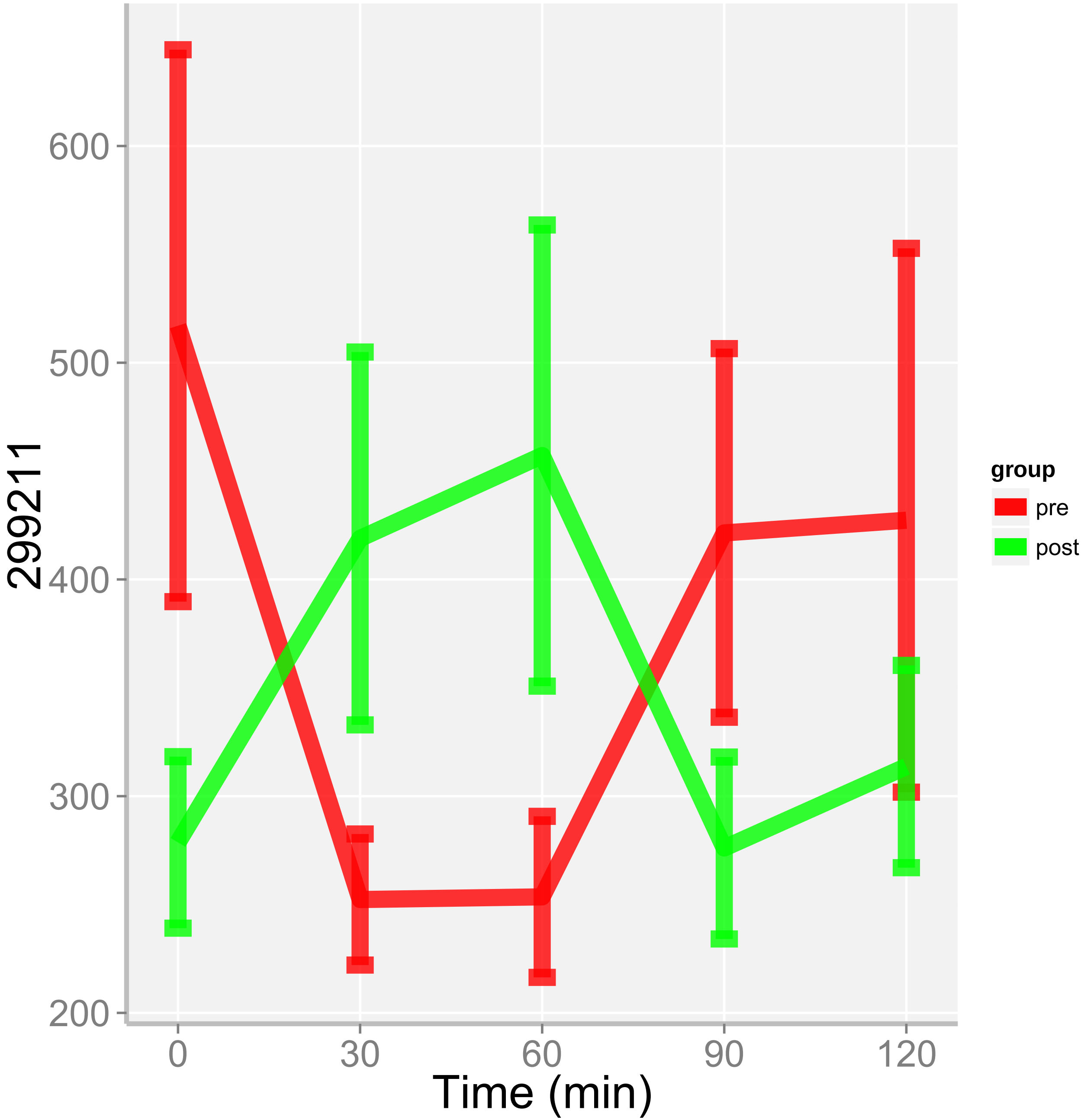

299211
600
500
400
300
200
0
30
60
90
120
Time (min)
group
pre
post

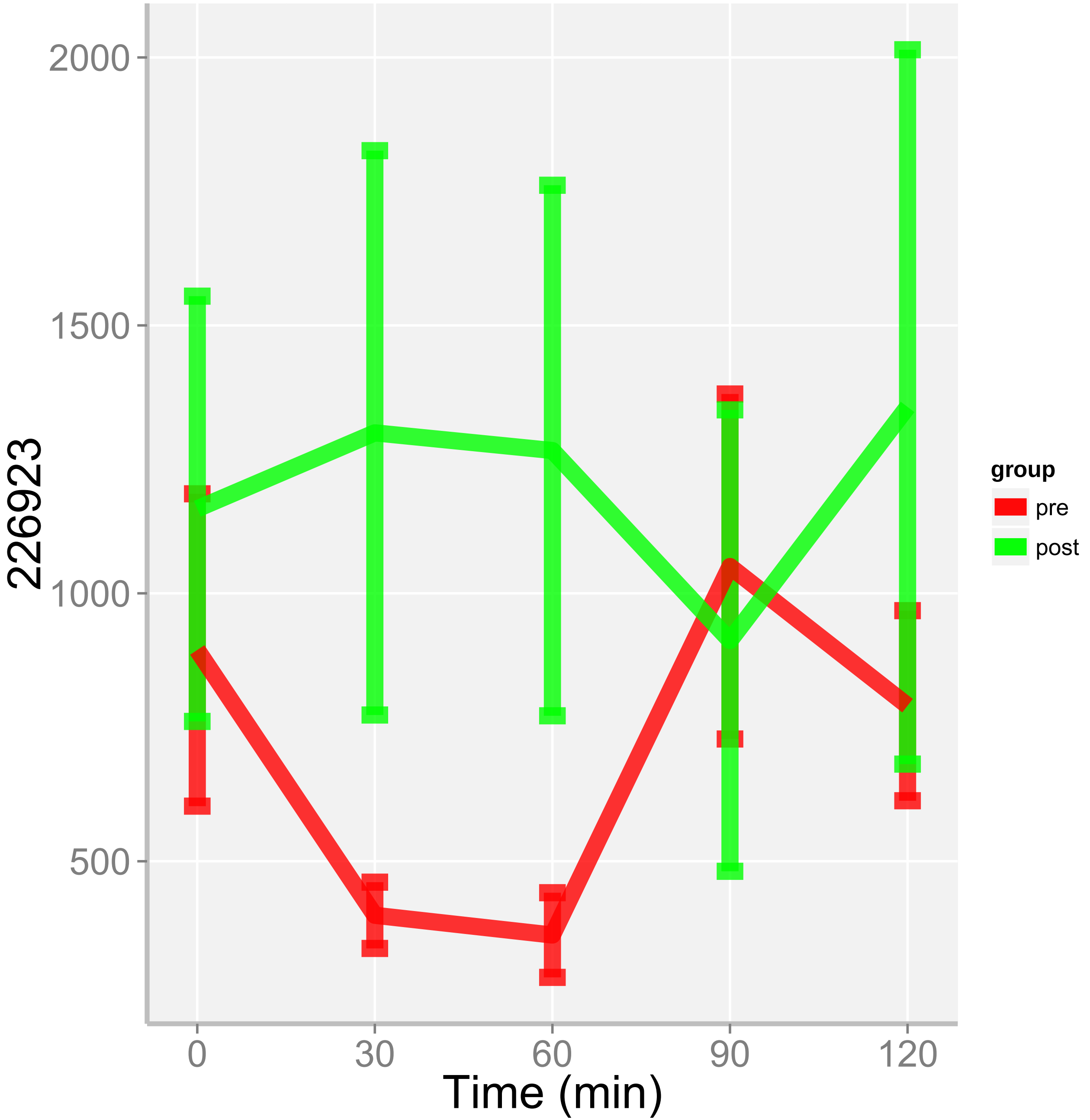
2000
1500
1000
500
226923
0
30
60
90
120
Time (min)
group
pre
post

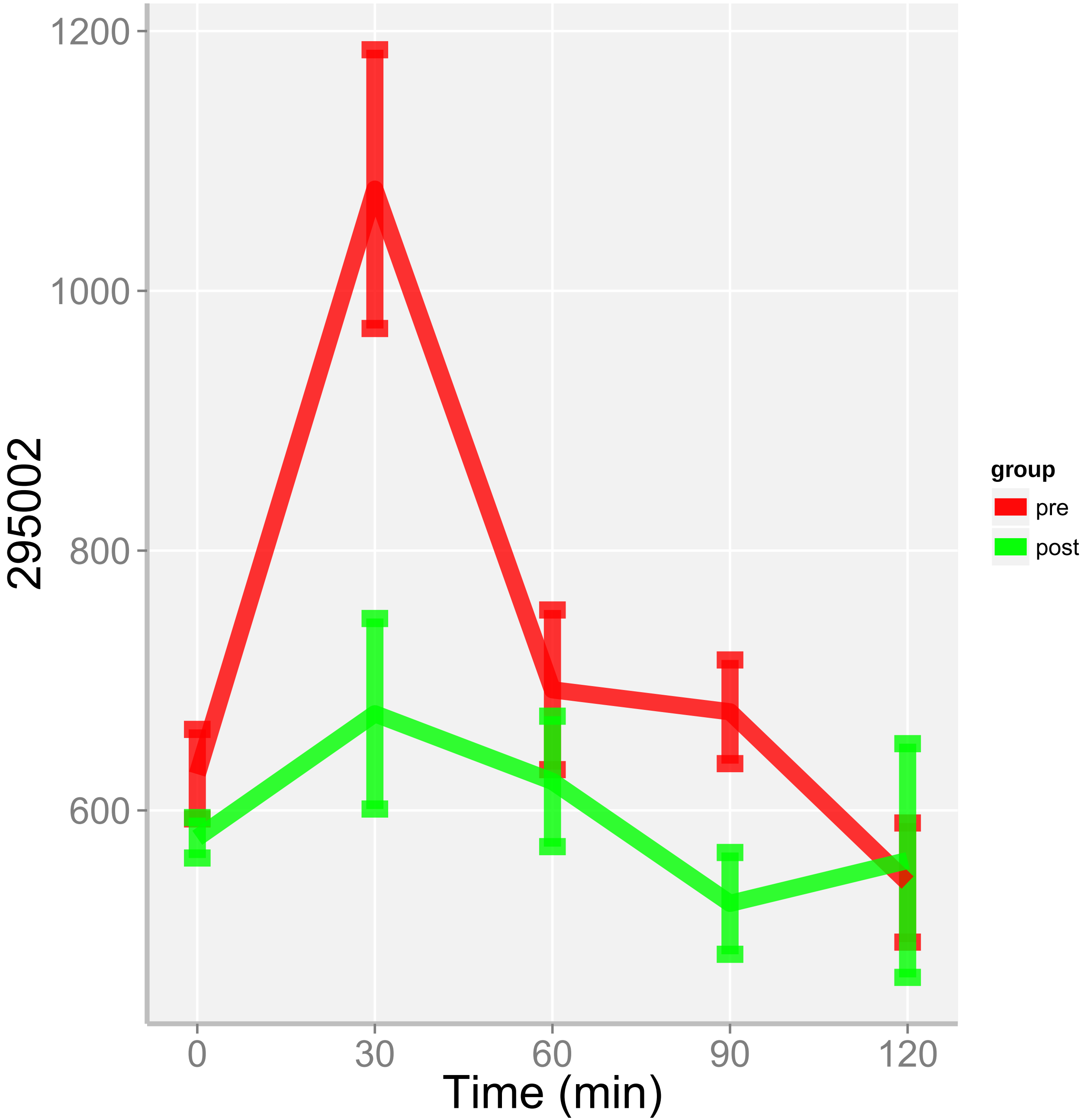
1200
1000
800
600
295002
0
30
60
90
120
Time (min)
group
pre
post

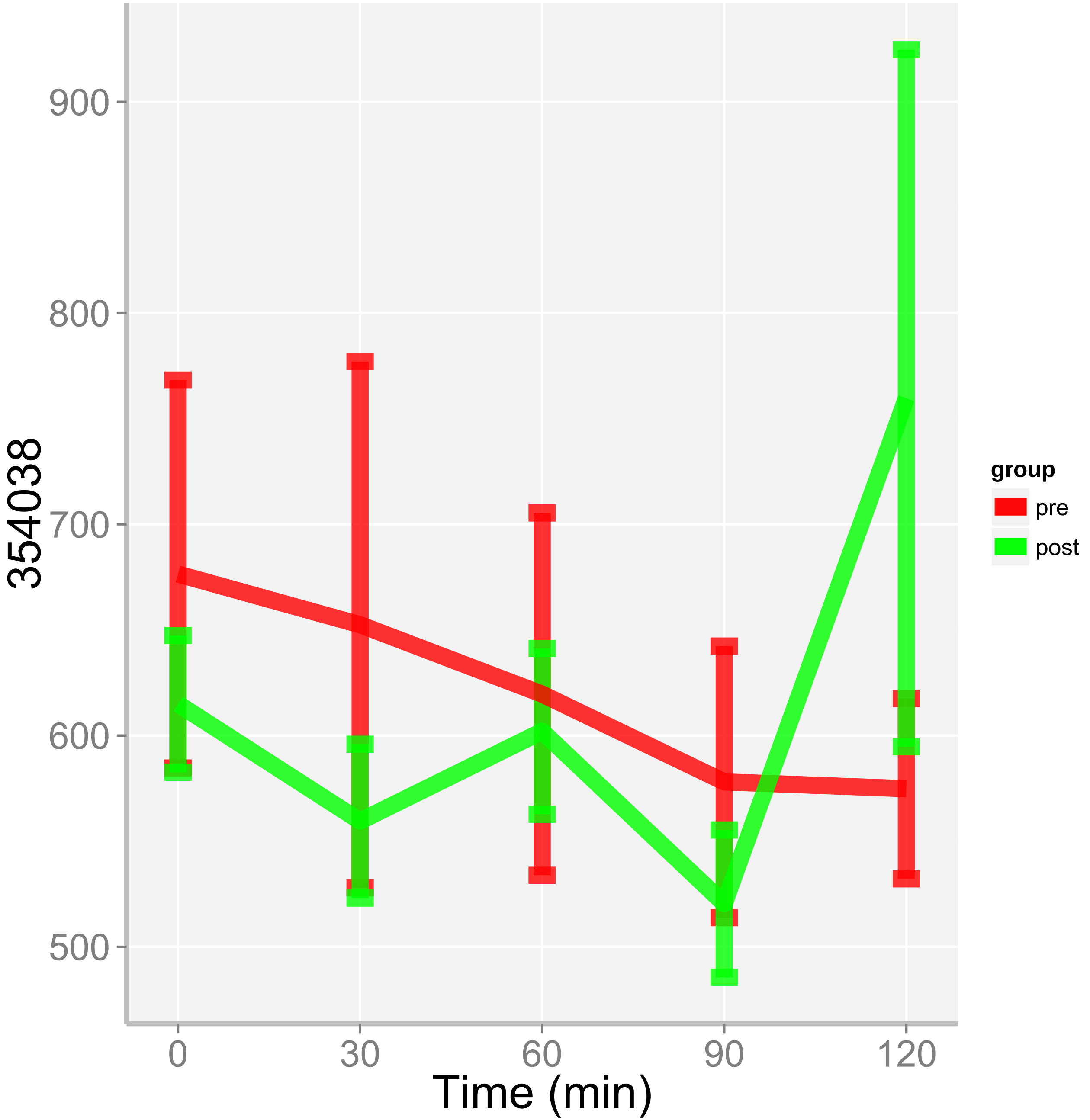

900
800
700
600
500
354038
0
30
60
90
120
Time (min)
group
pre
post

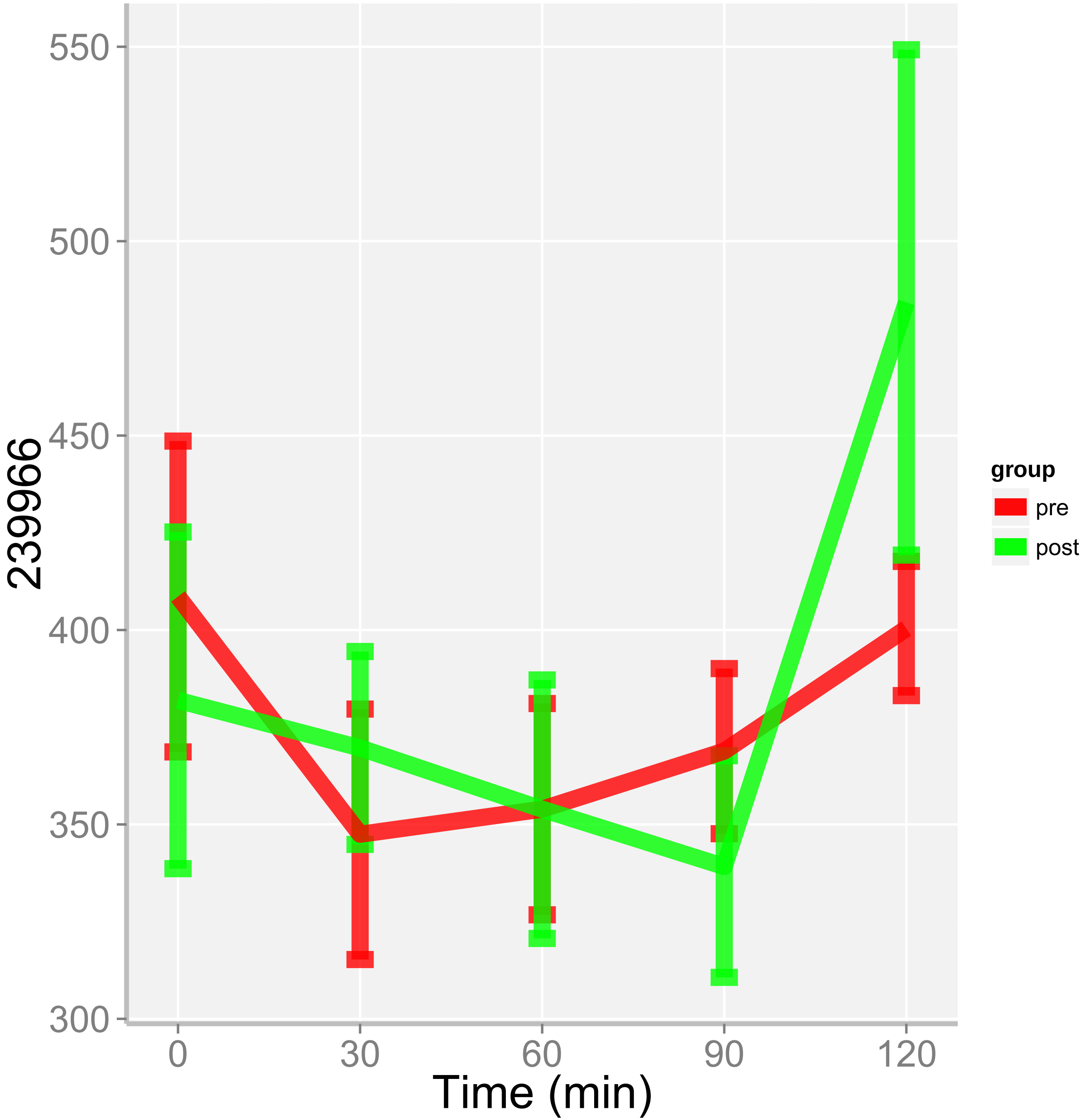

239966
550
500
450
400
350
300
0
30
60
90
120
Time (min)
group
pre
post

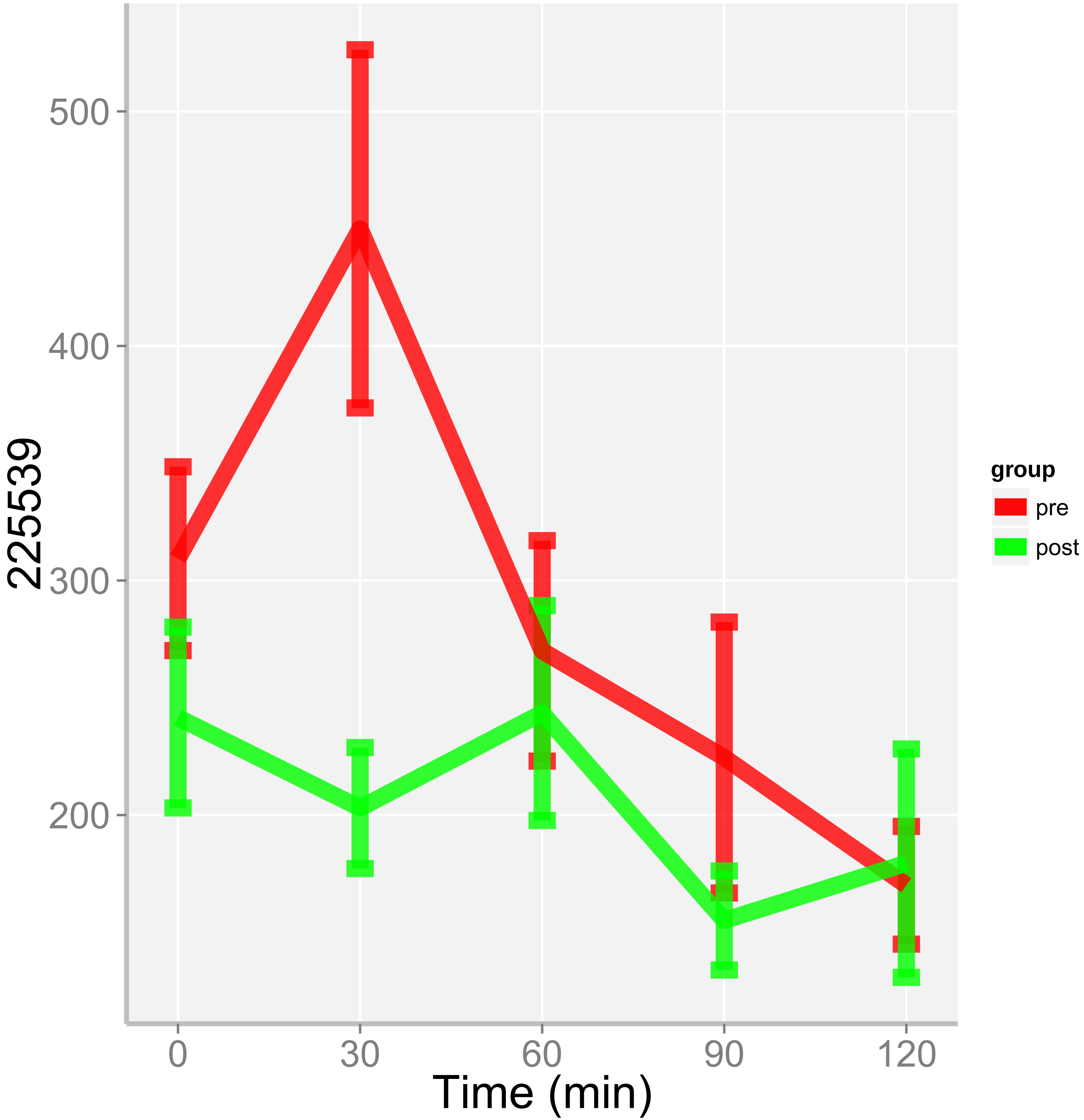

225539
500
400
300
200
0
30
60
90
120
Time (min)
group
pre
post

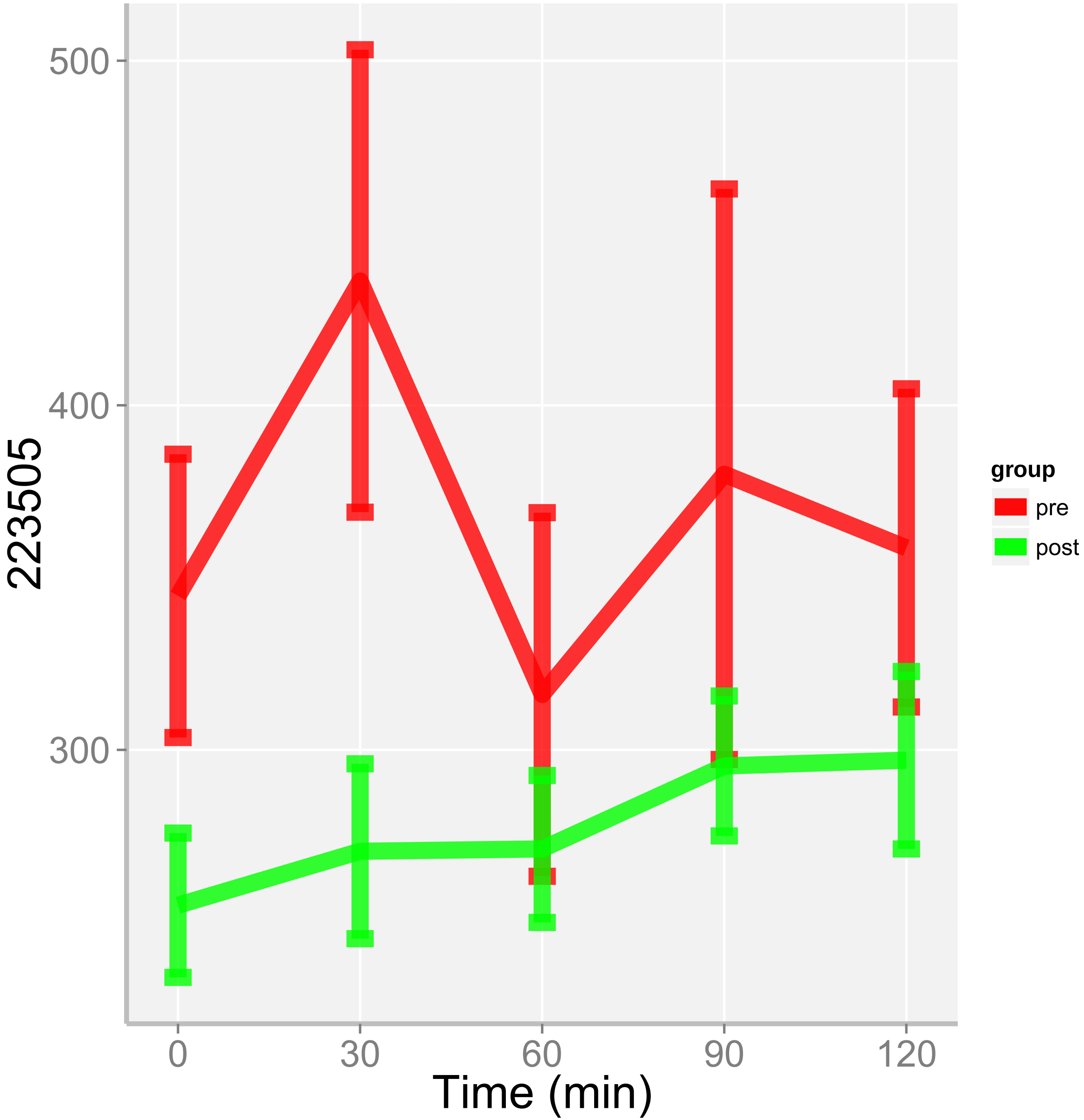

223505
500
400
300
0
30
60
90
120
Time (min)
group
pre
post

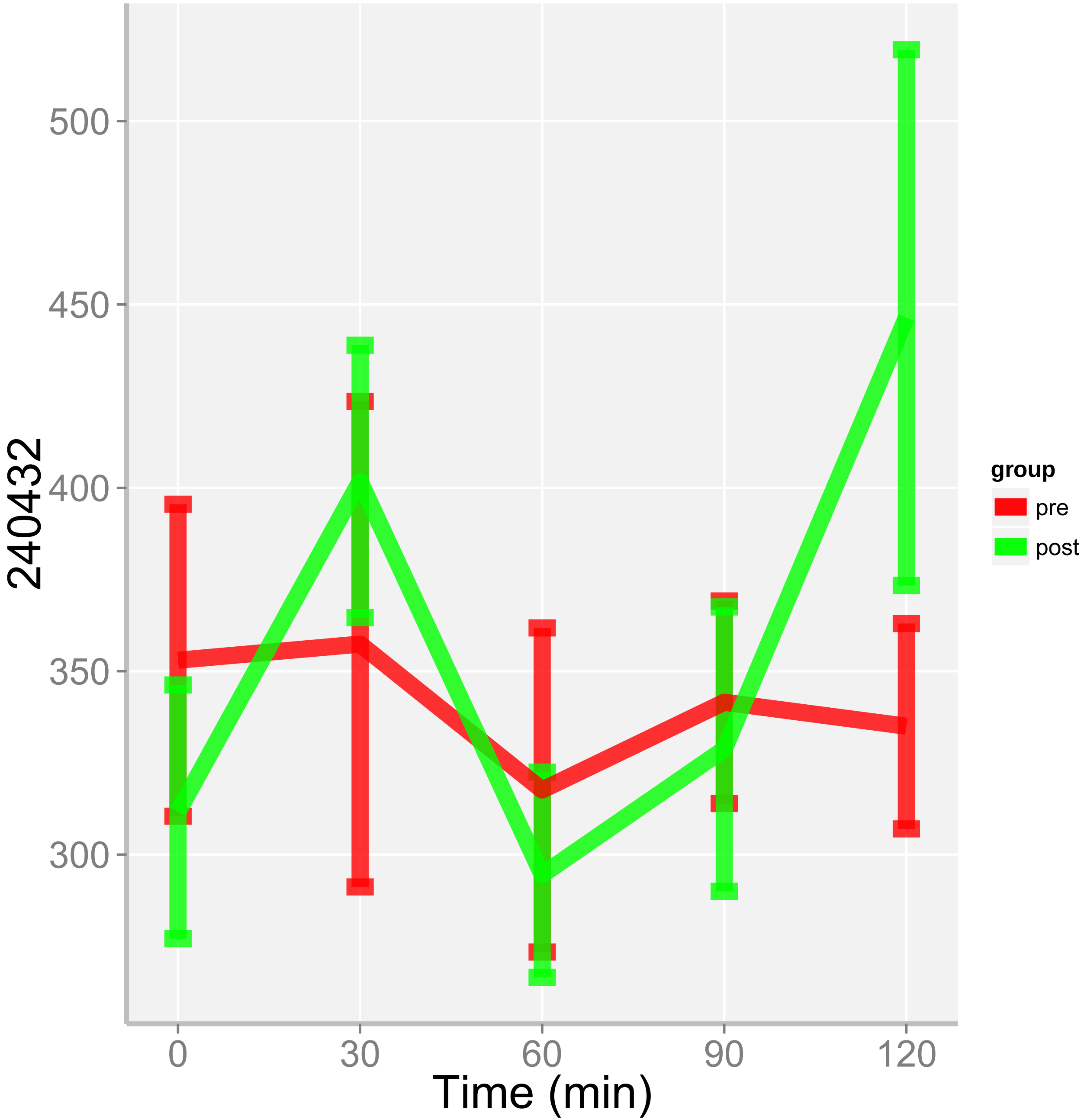

2404432
500
450
400
350
300
0
30
60
90
120
Time (min)
group
pre
post

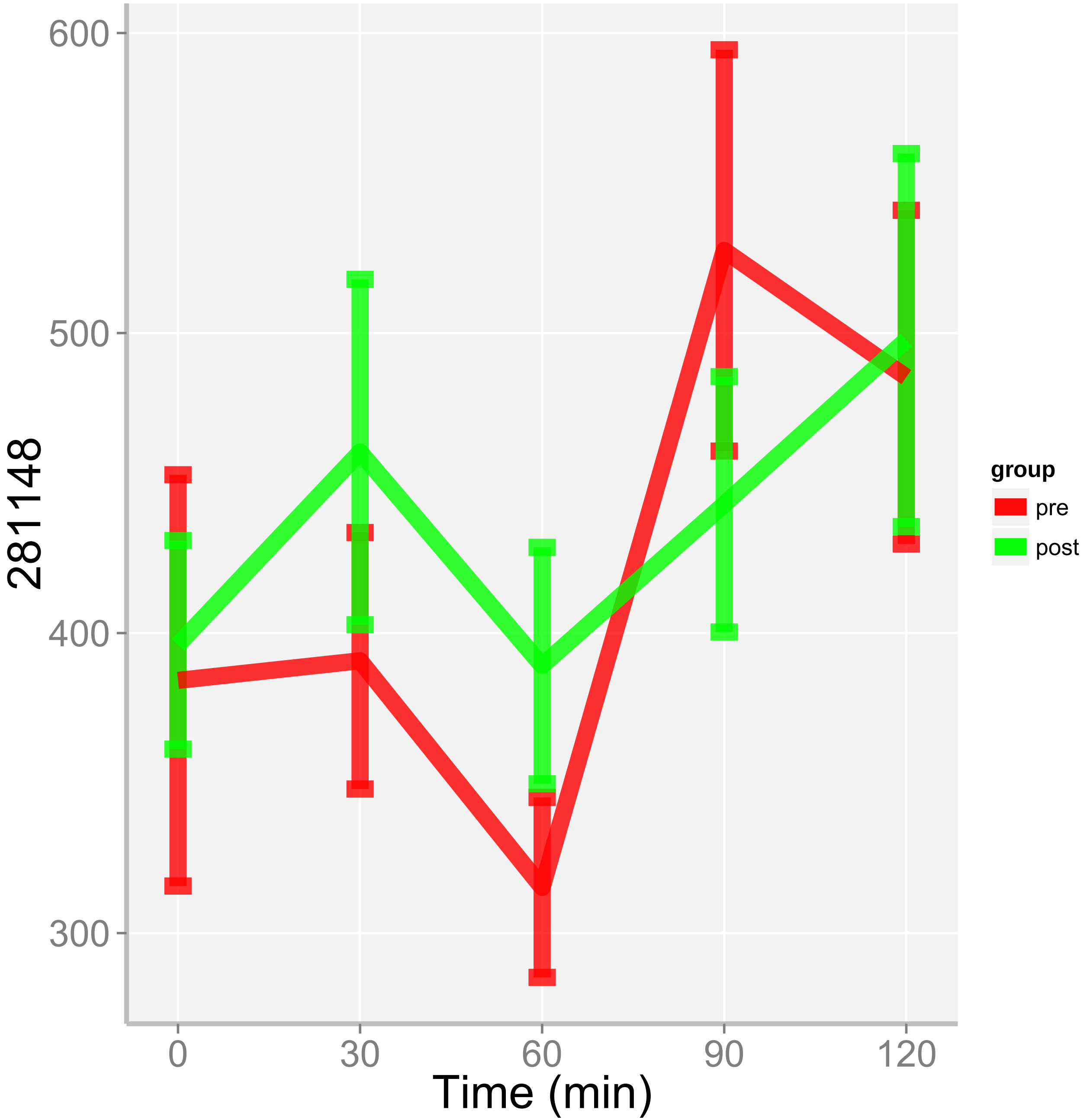

600
500
400
300
281148
0
30
60
90
120
Time (min)
group
pre
post

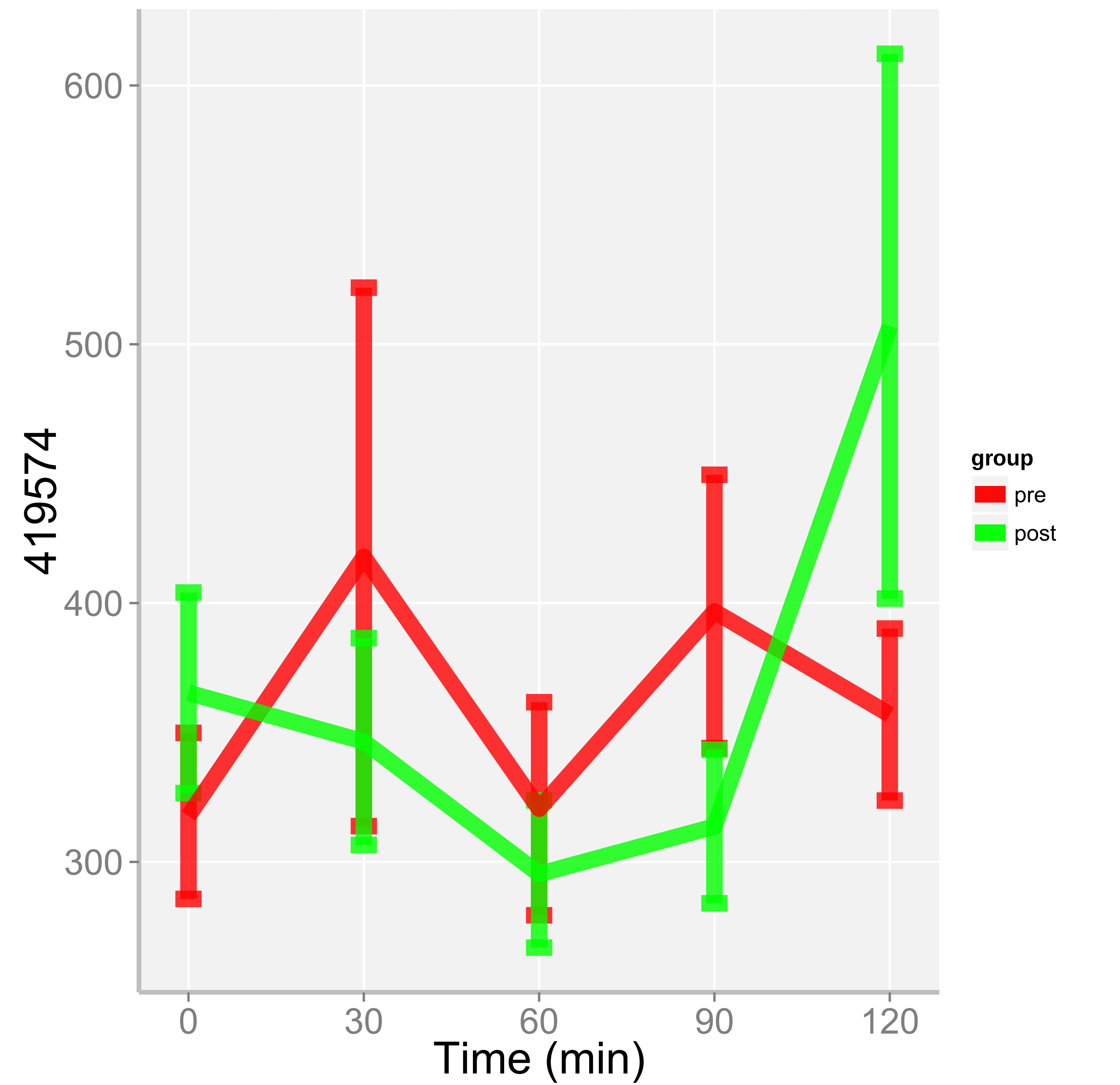

419574
600
500
400
300
0
30
60
90
120
Time (min)
group
pre
post

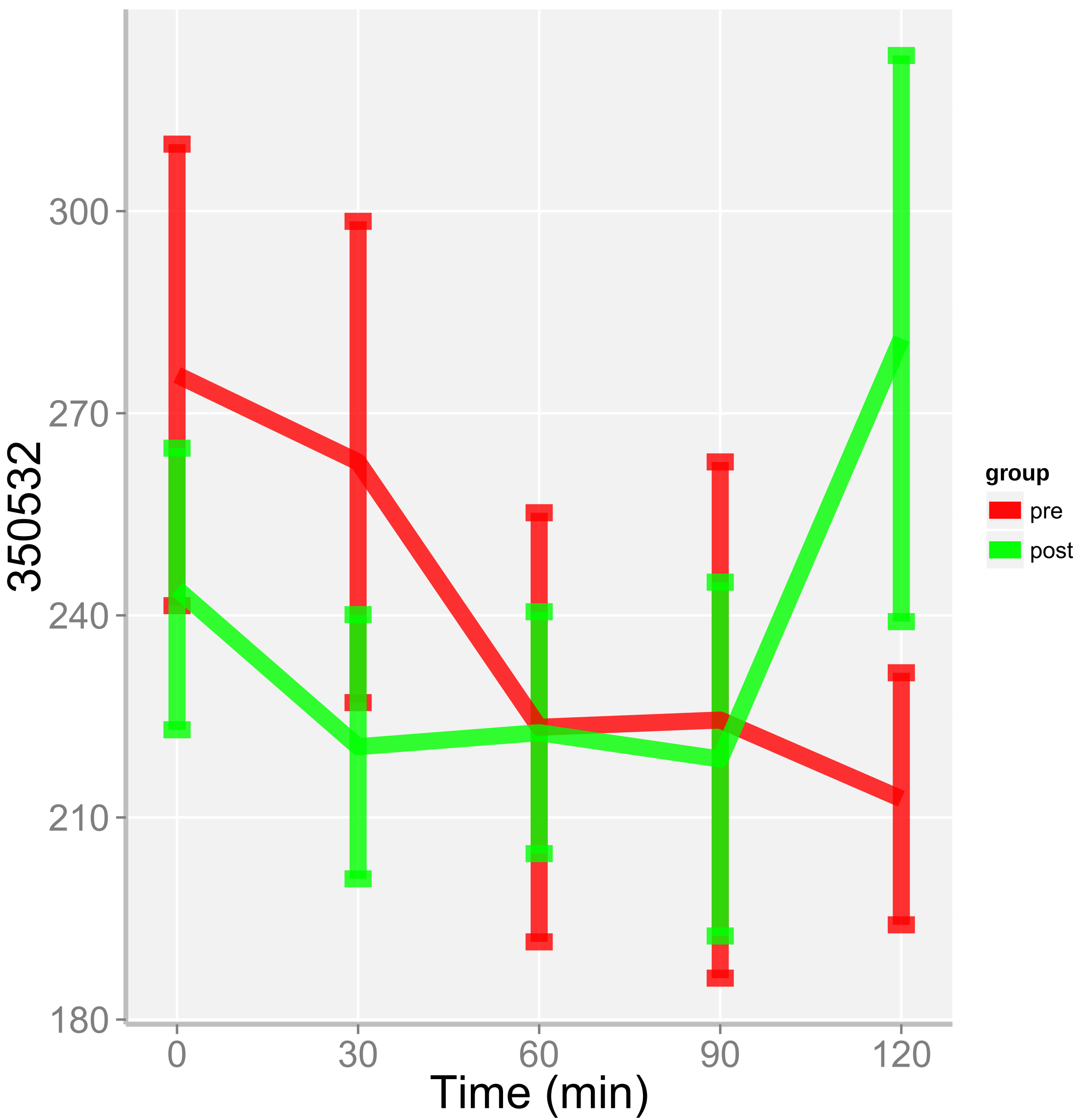

350532
300
270
240
210
180
0
30
60
90
120
Time (min)
group
pre
post
